# The Phylogenetic Relationships of *Tiaronthophagus* n.gen. (Coleoptera, Scarabaeidae, Onthophagini) Evaluated by Phenotypic Characters

**DOI:** 10.3390/insects10030064

**Published:** 2019-03-01

**Authors:** Angela Roggero, Philippe Moretto, Enrico Barbero, Claudia Palestrini

**Affiliations:** 1 Department of Life Sciences and Systems Biology, University of Torino, Via Accademia Albertina 13, I-10123 Torino, Italy; enrico.barbero@unito.it (E.B.); claudia.palestrini@unito.it (C.P.); 2 2 rue Marcel Sembat, F-83200 Toulon, France; naturafrique@gmail.com

**Keywords:** systematics, new species, new genus, geometric morphometrics, combined phylogenetic analysis, biogeography, necro-coprophagous habits, Afrotropical distribution

## Abstract

A necro-coprophagous new genus tha is widespread in the whole Sub-Saharan Africa was identified within the tribe Onthophagini and named *Tiaronthophagus* n.gen. The new genus, which is well characterized by an exclusive set of characters, comprises, at present, 26 species. Twenty species were formerly included in the genus *Onthophagus* and six were identified and here described as new species: *Tiaronthophagus angolensis* n.sp., *T. jossoi* n.sp., *T. katanganus* n.sp., *T. rolandoi* n.sp., *T. saadaniensis* n.sp., and *T. zambesianus* n.sp. A phylogenetic analysis that is based on a combined matrix, including discrete and landmark characters, was done. The landmark characters were tested using the geometric morphometrics techniques before their inclusion in the matrix. One single, fully resolved tree was obtained, with *Tiaronthophagus* constituting a distinct, monophyletic clade within Onthophagini, which was clearly separated from the other genera examined here. The biogeographical analysis identified the Central Africa as the ancestral area of the new genus and it mainly accounted for dispersal events leading to the present distribution. The generic rank that is assigned to the taxon is supported by the results of the morphological, phylogenetic, and biogeographical analyses, and by the comparison to the outgroups.

## 1. Introduction

Within the tribe Onthophagini Burmeister, 1846, the widespread genus *Onthophagus* Latreille, 1802 has now been found to exceed 2300 species worldwide, thence becoming one of the largest genera in the world [1]. The high biological diversification, as expressed by a great systematic complexity resulted into a troublesome taxonomic history, which makes the study of this genus extremely difficult. It was often emphasized how, in *Onthophagus,* different and surely not-related species often seem quite similar by an approximate survey of the morphological traits, thence the correctness of their taxonomic placement might be greatly affected. The majority of the Afrotropical *Onthophagus* species were assigned to groups of species by d’Orbigny [2], but the group-defining characters were often not unique and the classification remained ambiguous. The phylogenetic relationships within *Onthophagus* were recently examined [1,3,4,5,6,7,8], but the systematics of these species is far to be fully elucidated.

The already complicated systematics of the genus was exacerbated by the many new *Onthophagus* species that have been described along the years, to such an extent that nowadays the genus surpasses the 1000 species in the Afrotropical Region [9]. New taxonomic entities continue to be identified and included in *Onthophagus*, although some of the species-groups that were proposed by d’Orbigny [2] were already removed from *Onthophagus*, and new genera were thence established to accommodate part of those species [10,11,12,13,14,15,16,17,18,19]. Besides, the taxonomic rank of the majority of the Afrotropical species-groups are yet to be carefully evaluated in order to uphold their systematic position, and the taxa relationships remain basically unresolved due to the well known species richness of this megadiverse genus.

When we were examining the species that are included in the Afrontopical 28th *Onthophagus* group [2], we noted strong similarities with the part of the species belonging to the 16th and 24th groups (Table 1) according to the external and internal morphological features. All of these species shared many characters as the modified male vertex carina, with the evident frontal carina sometimes just discernible in major males, and few very large, scattered setigerous points on genae, pronotum lateral margins sinuate, and base were entirely rebordered. Furthermore, within material that was recently collected from various localities of sub-Saharan Africa, we identified Onthophagini specimens that are characterized by a marked likeness to the known species of these groups, although they could not be assigned to any of them.

The aim of the present research was to evaluate the taxonomic position of these species, which are closely related due to their likeness, testing also their relationships to other Onthophagini groups, while employing phenotypic characters of the external and internal morphology of adults. In the present research, it was also stressed whether the species should be regarded as a closely related cluster to which the generic rank should likely be assigned. Furthermore, the biogeographical diversity was tested by the evaluation of the present distribution data of these species.

## 2. Material and Methods

### 2.1. Material

The ingroup dataset comprises 26 Afrotropical species: 20 were formerly included in the speciose *Onthophagus* genus and six were identified here as new taxonomic entities to which specific rank was assigned (Table 1). The outgroup method was chosen to root the trees, selecting 10 representatives from Afrotropical and Palearctic Onthophagini (Table 1) to be included in the outgroup dataset. The choice of the outgroup taxa was done under the following considerations: (1) the well known, superficial resemblance within many “*Onthophagus*” species has greatly affected the taxonomic arrangements in the past; (2) the systematics of the majority of the Afrotropical Onthophagini species is far from being settled and their present knowledge has only been partially improved since the d’Orbigny Synopsys [2]; and, (3) no definition can be provided at present for *Onthophagus* due to the extreme heterogeneity of the species that are included in the genus. In this framework, the outgroup choice was thus based on the results that were gained from previous phylogenetic studies on Onthophagini [17,18], including *Onthophagus s.str.* species, the only ones, which, at present, can be regarded as true “*Onthophagus*”. The morphological features of these species were employed to define the discriminant criteria for the genus *Onthophagus* in our analysis. For the other *Onthophagus s.l.* species the effective taxonomic position cannot be defined with certainty for now, thus we chose to include the *Palaeonthophagus* species as a distinct taxon from *Onthophagus s.str*.

We examined more than 2000 specimens that were housed in the following institutions: MCST—Museo Civico di Storia Naturale, Trieste, Italy; MHNL—Musée des Confluences, Lyon, France; MMUE—Manchester University Museum, Manchester, United Kingdom; MNHN—Muséum National d’Histoire Naturelle, Paris, France; MSNG—Museo Civico di Storia Naturale Giacomo Doria, Genova, Italy; NHMW—Naturhistorisches Museum, Wien, Austria; SDEI—Senckenberg Deutsches Entomologisches Institut, Müncheberg, Germany; and, ZMHB—Museum für Naturkunde der Humboldt-Universität, Berlin, Germany.

We examined also material from private collections of E. Barbero (EBCT—Torino, Italy), M. Egger (MECI—Innsbruck, Austria), J.-F. Josso (JFJC—Treguen, Muzillac, France). M. Dierkens (MDCL—Lyon, France) and P. Moretto (PMOC—Toulon, France).

The typical material preserved in the following museums was compared to the non-typical material using the electronic facilities that were supported by the museums: BMNH—Natural History Museum, London, UK; MHNC—Musée d’Histoire Naturelle, La Chaux-de-Fonds, Switzerland; MNHN—Muséum National d’Histoire Naturelle, Paris, France; NHMB—Naturhistorisches Museum, Basel, Switzerland; RMNH—Nationaal Natuurhistorische Museum, Leiden, The Netherlands; SAMC—Iziko Museum of Capetown [formerly South African Museum], Cape Town, South Africa; ZSM—Zoologische Staatssammlung, Munich, Germany.

In addition to the aforementioned institutions and private collectors, other type specimens are preserved in: IRSNB—Institut Royal des Sciences Naturelles de Belgique, Bruxelles, Belgique; PPRC—P. Prévost, Lathus, France; RMIC—R. Minetti, La Ciotat, France; MFRC—M. François, Bar-sur-Aube, France; GWC—G. Werner, Peiting, Germany; CAS—A. Serrano, Lisboa, Portugal.

### 2.2. Characters

The morphological characters were studied while using a Leica^®^ DMC4500 (Leica Microsystems AG, Wetzler, Germany) digital camera that was connected to a stereoscopic dissecting scope (Leica^®^ Z16APO, Leica Microsystems AG, Wetzler, Germany). Images of the anatomical structures were acquired by the software Leica Application Suite (LAS, Leica^®^ Microsystems, Leica Microsystems AG, Wetzler, Germany), placing them in the same position in order to avoid error due to malpositioning.

**Figure 1:**
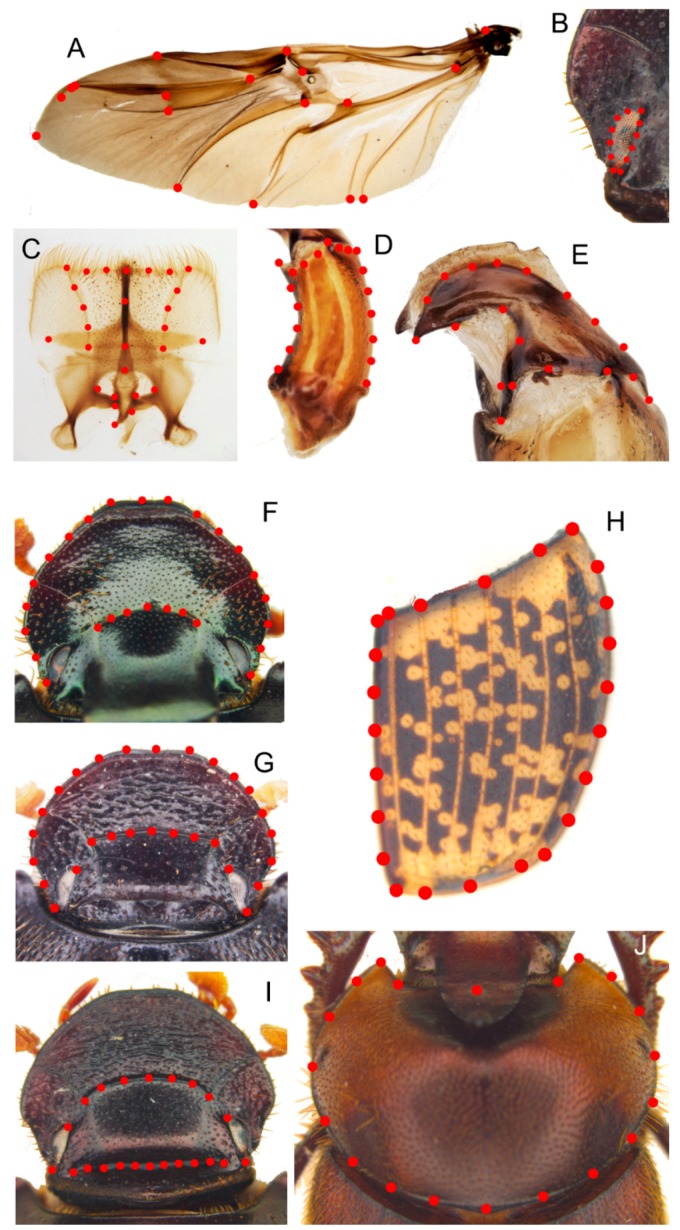
The landmark configurations employed in the analyses. (**A**): Hindwing, 18 landmarks. (**B**): Eye, 12 landmarks. (**C**): Epipharynx, 27 landmarks. (**D**): Phallobase, 19 landmarks. (**E**): Paramere, 19 landmarks. (**F**): Male head, 26 landmarks. (**G**): Female head, 28 landmarks. (**H**): Right elytron, 24 landmarks. (**I**): Female carinae, 22 landmarks. (**J**): Pronotum, 20 landmarks. For pronotum and eye, the same point configurations were applied in males and females, but the datasets were separately treated in the analyses.

Various external and internal anatomical traits were examined choosing those that were already usefully employed in the coleopteran taxa studies [9,20]. The characters were defined following the terminology that was used in other Onthophagini taxa [17,18]. The morphological dataset included: (1) the geometric morphometric partition, with 12 landmark configurations (Figure 1), chosen according to Palci and Lee [21]; and, (2) the discrete partition with 53 characters scored from external and internal anatomical traits of adult specimens (Supporting Information Data S1).

Since it was emphasized that the quality of phylogenetic analyses improves by increasing the number of landmark configurations that were used in the analysis [22,23], here we employed many different structures (head, eye, and pronotum of both sexes, female head carinae, hindwing, right elytron, epipharynx, phallobase, and right paramere) that were analysed applying the geometric morphometric approach, as already done in other Scarabaeidae taxa [24]. The softwares tpsDig v2.31 [25] and tpsUtil v1.76 [26] were employed to create the landmark datasets (Figure 1), while tpsSmall v1.34 [27] and tpsRelw v1.69 [28] were employed to evaluate the overall shape variation within the chosen datasets. The canonical variate analysis (CVA), as implemented in [29], tested the taxa attribution analysing the RW scores, setting the Mahalanobis distance stepwise method and leave-one-out classification. Additionally, the scatterplots of the RW scores were generated using SPSS software, defining the ingroup and outgroup taxa.

The matrix of landmark characters was built including the aligned values of each structure. In the phylogenetic analysis, each shape configuration was set as a single character, as recently implemented in TNT [30], being that it “allows running a combined analysis of traditional and phylo-morpho characters in an analogous way as two (or more) different gene sequences can be analysed together where each one contributes to the resolution of a phylogeny” [31].

The two partitions (21 binary and 32 multistate qualitative characters and 12 landmark characters) were combined into a single matrix (Supporting Information Data S2) to analyse the phylogenetic relationships within the taxa. The partitions were also separately examined to evaluate the phylogenetic signal that was provided by each structure, and the influence of each configuration in defining the relationships among the *Tiaronthophagus* species [32].

### 2.3. Phylogenetic Analysis

The software TNT v1.5 [30] (freely available at: http://www.lillo.org.ar/phylogeny/tnt/) analysed the discrete and continuous datasets first separately and then together, under equal weighting, and while applying different settings for each analysis. An initial parsimony analysis was performed on the discrete dataset (equal weights), with 10,000 random addition sequences to the TBR method, retaining 10 trees for each replication. Subsequently, a new technology search was done selecting sectorial search, ratchet, drift, and tree fusing options, as implemented in TNT. The outgroup method was chosen to root the resulting trees in all of the analyses. The ‘multiple taxa’ option = ON was chosen to test the hypothesis that the ingroup taxa were well separated from any of the outgroups. Thus, the *Tiaronthophagus* species were grouped together, while the outgroup species were not grouped thus the latter ones were not forced together.

The landmarks characters were separately examined by traditional search (Wagner parsimony), setting off the option ‘re-align data landmarks’, since the values were already aligned in tpsRelw (see above).

Finally, the full dataset merging discrete and landmark equal-weighted characters was analysed with the following initial settings: (1) implied weighting: OFF, to give the same weight to any character; (2) lmark confsample: ON, to include the landmark data; and, (3) force: ON, to allow for multiple outgroups in the search. The Hendrickx script [31] is available at http://phylo.wikidot.com/tntwiki. The landmark data were not re-aligned, since the aligned datasets from tpsRelw were included in the matrix [21]. Results and discussion were based on the combined analysis.

The combined matrix of discrete and landmark characters underwent the heuristic (traditional) search method, as implemented in TNT (Wagner starting trees) with 100,000 random addition sequences, followed by the tree bisection reconnection (TBR) branch-swapping algorithm (100 trees per replication save limit). Additionally, the ‘landcombsch.run’ [31] and ‘aquickie.run’ (TNT package) scripts were applied, with the same initial settings of the Wagner parsimony analysis.

The resulting trees of the analyses were thus compared to evaluate whether there were any differences when applying different methods to the combined matrix. Statistics (i.e., tree length, adjusted homoplasy, consistency index, and retention index) were evaluated for each tree. The TNT default options for resampling were used to determine branch support by the absolute bremer and relative bremer supports, as calculated using the TNT Bremer function (i.e., bremer.run). Moreover, the standard bootstrap, symmetric resampling, and jackknife values were calculated setting the traditional search method with nreps = 1000 and when considering the whole landmark configurations for resampling and relative support. The average group support values of each phylogenetic hypothesis were calculated with TNT. Discrete and landmark characters were separately mapped onto the tree, as implemented in the software, and then the synapomorphies were evaluated, retaining the list of the common synapomorphies.

The resulting trees were then drawn by TreeGraph2 v2.14.00-771beta [33].

### 2.4. Biogeographical Analysis

The distribution of *Tiaronthophagus* was subdivided into areas using the InfoMap online facility (http://bioregions.mapequation.org/), a powerful interactive web application [34,35] performing bipartite network clustering, whose aim is to identify taxon-specific bioregions from species distribution data. The dataset of the *Tiaronthophagus* species distribution was run at the InfoMap Bioregions web page (http://bioregions.mapequation.org/), setting max cell size = 4, min cell size = 1, max cell capacity = 100, and min cell capacity = 1. In the analysis, the phylogenetic tree was not time-calibrated. The analysis can also define the most common and indicative species for each area, allowing for evaluating ‘endemic species, unique or close to unique to a specific bioregion’ [35]. The resulting clusters defined the *Tiaronthophagus* bioregions, and the output data were summarized into a shape file, subsequently run in GIS-environment [36].

The software Vicariance Inference Program (VIP) analysed the spatial data and the *Tiaronthophagus* phylogenetic tree together [37], visualizing the results onto a map. The grid was generated applying the von Neumann neighborhood option, with grid size = 1 and maximum fill = 1. In the Heuristic search (100,000 iterations, holding 10 reconstructions per iteration), the following options were selected: Page’s heuristic, flip nodes, start with a sector (being sector size randomly set to 20), no annealing, 1000 generations. Subsequently, the consensus of the calculated reconstructions was examined onto the map. The potential geographic barriers that are associated with the disjunctions (calculated using the Voronoi tessellation) were shown in the maps.

The software QGis v3.0.1 [36] was employed to elaborate the maps.

### 2.5. Taxonomy

The collection material was carefully evaluated, and when compared to the type specimens preserved in various museums (see the full list above) to verify the specific attributions, the species were then re-arranged according to the present findings. On the basis of the external and internal morphological characters, we identified specimens that were different from any known species. Since these specimens could not be assigned to any known species, their taxonomic positions were thence evaluated.

Additionally, the actual synonymies and name availabilities of the species were carefully examined. For the species thatwere included in *Tiaronthophagus* n.gen., the lectotypes were designated when required, according to the rules of the ICZN (http://iczn.org/ articles 73 and 74) to fix the taxonomy and avoid any ambiguity.

## 3. Results

### 3.1. Discrete and Landmark Characters

The 53 discrete characters that were gained by the comparison of the *Tiaronthophagus* and the outgroups taxa were included in the matrix of characters (Supporting Information Data S2) to be employed in the subsequent phylogenetic analysis. Characters that could be regarded as duplicating the landmark characters (see below) were not included in the dataset.

Before using the twelve landmark characters to build the combined matrix, they were carefully examined to evaluate the differential patterns of shape variation of the ingroup and outgroup taxa for each structure. The evaluation of the configurations, as implemented by tpsSmall, gave a significant result for each of them (correlation ≈ 0.999), thus allowing the application in the following analyses.

**Figure 2:**
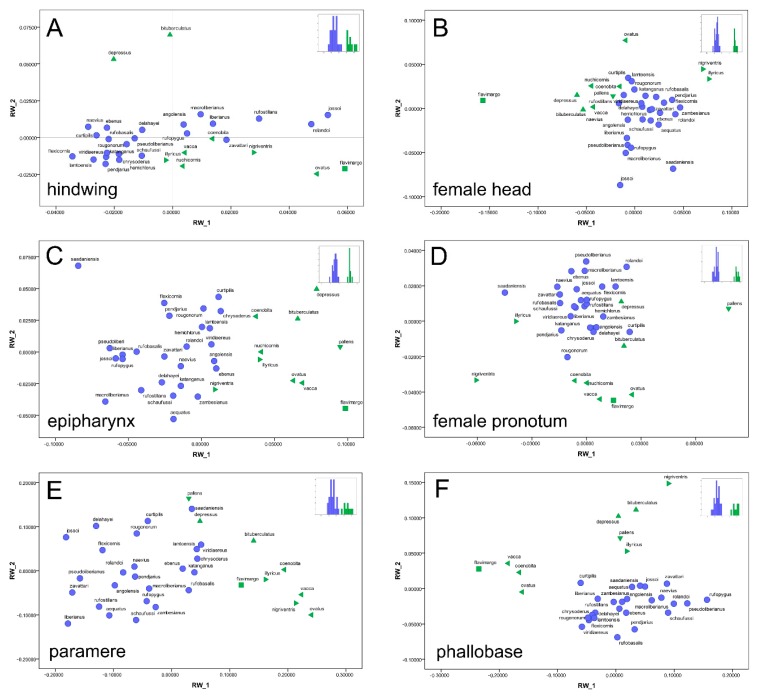
The scatterplots of the two first relative warps of (**A**): Hindwing. (**B**): Female head. (**C**): Epipharynx. (**D**): Female pronotum. (**E**): Paramere. (**F**): Phallobase. In the plots, blue points = ingroup *Tiaronthophagus* taxa, green points = outgroup taxa. The histogram summarizing the results of canonical variate analysis (CVA) on the whole relative warp (RW) scores is also given.

The relative warps analysis (i.e., PCA) of each configuration highlighted marked differences between the ingroup and outgroup taxa. The scatterplots of the first two relative warps (RWs) showed two well-separated clusters. Here, the plots of the hindwing, epipharynx, female head and pronotum, paramere, and phallobase were given (Figure 2). The statistics of each structure were examined, in order to assess the analogous developmental patterns.

The RW scores summarize 100% of the overall shape variation [38] were used for the canonical variate analysis (CVA). Congruent results were obtained for all of the examined structures, thus it was confirmed that the *Tiaronthophagus* genus was a well differentiated taxon from the other Onthophagini examined here (Figure 2). The evaluation of each dataset providing different patterns of shape variation is noteworthy here.

The hindwing plot of RWs 1 and 2 (Figure 2A) accounted for more than 45% of the overall shape variation, with the first six out of 31 RWs being significantly higher than 5%, and explaining together more than 76% of the overall shape variation. The CVA confirmed the differential pattern, being 96.9% of cross-validated grouped cases correctly classified.

In the plot of the female head (Figure 2B), two distinct clusters were present. The first two RWs accounted for about the 69% of the overall shape variation, and the first four (out of 35 RWs, having a score > 5%,) for about the 85%. In the plot, the ingroup and outgroup taxa were clearly separated, and the CVA labelled 100.00% of cross-validated grouped cases correctly classified.

Although the analysis of the male head gave similar results, being the overall shape variation more than 72% for RWs 1 and 2, and about 88% for the first four out of 33 RWs, in the plot of the first two RWs (not shown), the two groups are more closely related, also providing 91.2% of cross-validated grouped cases correctly classified.

The analysis of the female head carinae gave a high percent value of the explained overall shape variation, being more than 84% for the first two, and more than 92% for the first three (percent scores > 5%) out of 35 RWs. Besides, in the plot of RW 1 and 2 (not shown here) the ingroup and outgroup taxa were more closely related, and the CVA also confirmed the identified trend, with the 88.9% of cross-validated grouped cases being correctly classified.

The scatterplot of RWs 1 and 2 for the right elytron accounted for more than the 68% of the overall shape variation, with the first four out of 35 RWs explaining more than 88% of the overall shape variation. In the plot (not shown), the two proposed groups are quite close, as the CVA also confirmed it, with 94.4% of cross-validated grouped cases being correctly classified.

The plot of epipharynx highlighted more that 61% of the overall shape variation (Figure 2C), with the first four RWs (percent values > 5%) explaining more than 76% of overall shape variation. In the plot, the two groups are well-separated and the CVA confirmed the high discriminant power of the epipharynx, with 100.0% of cross-validated grouped cases being correctly classified.

The pronotum of both sexes evidenced two distinct groups in the plot of RWs 1 and 2, representing, respectively, more than 73% (males, not shown) and about 70% (female, Figure 2D) of the overall shape variation, and in both sexes, the first four RWs (percent values > 5%) accounted for about the 85% of the overall shape variation. Besides, while the CVA pronounced 100.0% of cross-validated grouped cases correctly classified for the female pronotum, the cross-validated grouped cases correctly classified were 94.1% for male pronotum.

For the eye in male and female (both plots not shown here), analogous patterns were obtained, being the well separated ingroup from the outgroup taxa, which instead (as expected) did not constitute a homogeneous group. In female, the plot of RWs 1 and 2 together accounted for about 77% of the overall shape variation, with four RWs (percent scores > 5%) out of 20 RWs explaining more than 92% of the overall shape variation. The cross-validated grouped cases correctly classified was 86.1%, with a partial superimposition in the plot. In males, the first two RWs plot explained more than 66% of the overall shape variation, and for the first four RWs (percent scores > 5%, out of 20 RWs), the number was 87%. The ingroup was also homogeneous here, while the outgroup taxa were differentiated. The CVA confirmed the result, with 88.2% of the cross-validated grouped cases being correctly classified.

The phallobase evidenced similar patterns, since, in the plot (accounting for more than 76% of the overall shape variation, Figure 2E), the ingroup taxa were clearly homogeneous but the outgroup taxa were separate, with marked differences for this structure. The first four RWs (scores more that >5% out of 33 RWs) together represented more than 88% of the explained shape variation. The CVA correctly classified 100.0% of the cross-validated grouped cases for the phallobase.

The paramere plot of RWs 1 and 2 (Figure 2F, more than 69% of the overall shape variation explained) also defined two distinct groups, with the overall shape variation accounting for the first four out of 33 RWs about 85%, and 97.2% of cross-validated grouped cases were correctly classified by CVA.

On the whole, the twelve landmark characters furnished useful information regarding the shape variation within *Tiaronthophagus* in comparison of the outgroups, thus all of them were included in the matrix for the phylogenetic analysis.

### 3.2. Phylogenetic Analysis

The parsimony analysis (both traditional search and new technology search) of the discrete characters gave 465 trees (tree length = 196, CI = 0.566, RI = 0.829); in the strict consensus (results not shown here), the *Tiaronthophagus* clade was distinct from the outgroup taxa, but the phylogenetic relationships among the species were not fully elucidated. The new technology search gave a similar consensus from six trees in which the *Tiaronthophagus* clade is defined, but the phylogenetic relationships within the genus also remained unsolved. Additionally, the resampling analyses confirmed that *Tiaronthophagus* constituted a distinct clade (100% branch support value at basal node of the clade for standard bootstrap, jackknife, and symmetric resampling).

The separate parsimony analyses of the landmark datasets (resulting trees not shown here) highlighted a common pattern for the ingroup clade, also evincing the relationships within the genus that constituted a well differentiated group of Onthophagini, as already seen in the geometric morphometric analysis. Each of the trees that were obtained from a single landmark character gave better resolved relationships among the species than the one from the analysis of the discrete dataset.

The landmark characters concur to define the phylogenetic relationships within *Tiaronthophagus*, thus, according to the former results, the twelve landmark characters were included in the combined matrix.

**Figure 3:**
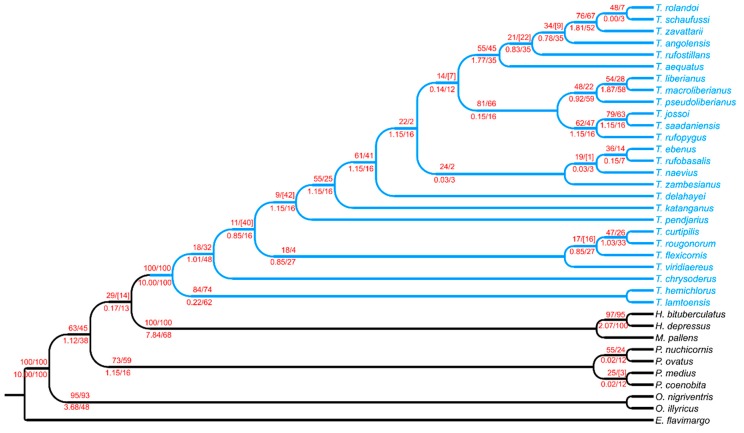
Tree from the phylogenetic analysis with the branch supports. Below branches: Bremer supports and relative Bremer supports. Above branches: symmetric resampling (P = 33), and the difference in support measured using frequency differences between the most frequent group and the most frequent contradictory group (GC values). The resulting values of jackknifing (P = 36), and standard bootstrap values were analogous to the symmetric resampling ones. Calculated tree statistics: tree length = 260.343, adjusted homoplasy = 15.108, consistency index = 0.462, retention index = 0.724, scores for 247 landmark points = 64.343.

Instead, the combined analysis gave a unique fully resolved tree from parsimony analysis (Figure 3, being the tree length = 260.343, adjusted homoplasy = 15.109, CI = 0.462, RI = 0.724, and scores for 247 landmark points = 64.343).

Within *Tiaronthophagus* some clades were defined (*T. rufopygus*/*T. liberianus*; *T. aequatus*/*T. schaufussi*; *T. zambesianus*/*T. ebenus*; and, *T. viridiaereus*/*T. curtipilis*). The common synapomorphies were calculated (Supporting Information Data S3), evidencing how the landmark characters greatly contribute to defining the phylogenetic relationships within the *Tiaronthophagus* species. The unique fully resolved tree that resulted from the analysis using the Hendrickx script was almost identical to the former one (Figure 3), with two differences at terminal nodes for *T. viridiaereus* and *T. flexicornis*, and *T. aequatus* and *T. angolensis*, which were here regarded as sister species. The unique tree from the aquickie run (not shown here) was also identical to the parsimony analysis. The branch support values (Figure 3) gave congruent results. Although *Tiaronthophagus* constituted a well differentiated clade from the other Onthophagini taxa, the phylogenetic relationships within the new genus were not fully elucidated, some with branches having low support values.

### 3.3. Biogeographical Analysis

**Figure 4:**
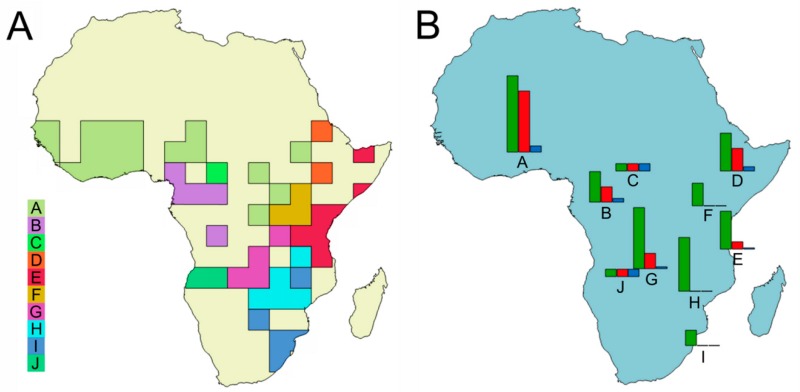
Results of the analysis by InfoMap. (**A**): Map of the 10 areas identified. (**B**): Map showing for each area (A–J) the total number of species (green bar), the number of endemic species (red bar), and the percent relative endemism (blue bar).

Ten macro-areas (A–J) were identified using InfoMap (Figure 4A), with A being the most extended, and also characterized by the major species abundance, with eight out of ten species being exclusively found in this area (Table 2). In the macroarea D five species are present, being three endemic of the area; two endemism were instead identified in both areas G (out of eight species) and B (out of four species). A single endemism was identified in C, E, and J, corresponding to one (*T. jossoi*) out of seven species in E, but to the totality of the *Tiaronthophagus* species in both C (*T. hemichlorus*) and J (*T. angolensis*). In F, H, and I, no endemism was detected, with H also being one of the most speciose areas, with seven species (Table 2). On the whole, the more widespread species are *T. rolandoi* (DEFGHI), *T. curtipilis* (ABGH), *T. rufobasalis* (DEFH), and *T. zambesianus* (EFGH), but about 70% of the *Tiaronthophagus* species are endemic to a single macroarea (Figure 4B).

**Figure 5:**
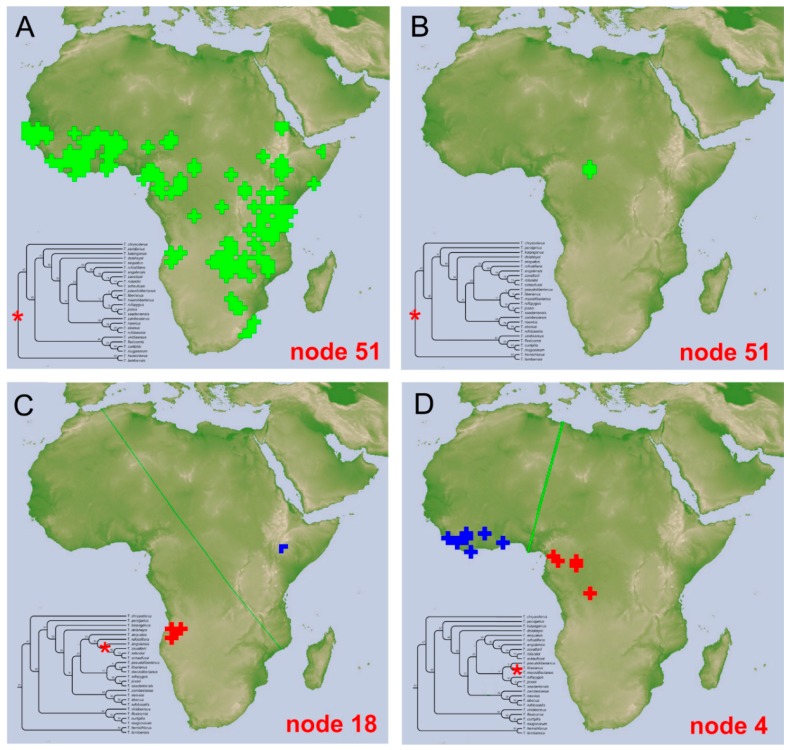
Vicariance Inference Program (VIP) reconstruction maps, with the corresponding node marked on the tree (red asterisk). (**A**): Map of OR (i.e., original or default) reconstruction at node 51. (**B**): Map of consensus reconstruction at node 51. (**C**): map of consensus reconstruction at node 18. (**D**): map of consensus reconstruction at node 4, with the Voronoi line in green. In the reconstructions, the allopatric distributions were defined by using different colours (red and blue) for each descendant, while overlaps were displayed in green.

Using the Page’s heuristics approach, from an initial OR (i.e., original or default) reconstruction, the search gave 12,511 reconstructions (not shown here), and then the consensus was calculated. Reconstruction statistics were given for the OR, heuristic, and consensus reconstructions. For the OR reconstruction, the cost was calculated to 17.000, the disjunct sister pairs were 8, and the nodes with removals 0, while for the consensus reconstruction, these values were 0.000, 2, and 13, respectively. The reconstruction buffer had instead cost = 13.000, disjunct sister pairs = 12, and nodes with removal = 4. The maps of the initial OR and consensus reconstructions (Supporting Information Data S4) were compared for each node, evidencing marked differences. The many vicariance events that were proposed by the OR reconstruction (Supporting Information Data S4) were reduced to two in the consensus reconstruction, at node 18 and 4, respectively (Figure 5C,D). Furthermore, at node 51, the OR reconstruction gave a large dispersal area (Figure 5A), while, in the consensus reconstruction, the dispersal event was set in a far more reduced area in Central Africa (Figure 5B).

### 3.4. Taxonomy

#### 3.4.1. *Tiaronthophagus* n.gen.

Zoobank Registration: http://zoobank.org/urn:lsid:zoobank.org:act:5548CA9E-A47D-4B1B-AFAC-3D037BFD3AB5.

**Type species**. *Onthophagus schaufussi* Harold, 1867.

**Etymology**. The genus is named after the characteristic features of the vertex carina of major males resembling a *tiara* i.e., an antique Central Asian headdress, usually cone-shaped with the tip bent forward. In ancient times, this high-peaked headpiece was also used as a crown by the Persian kings.

**Figure 6:**
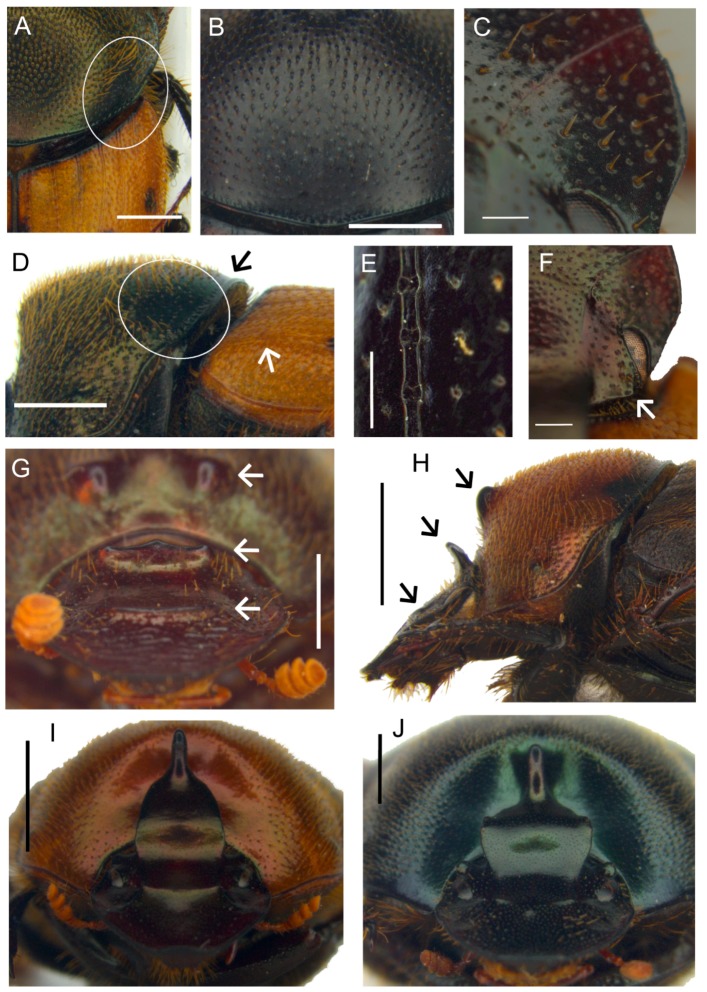

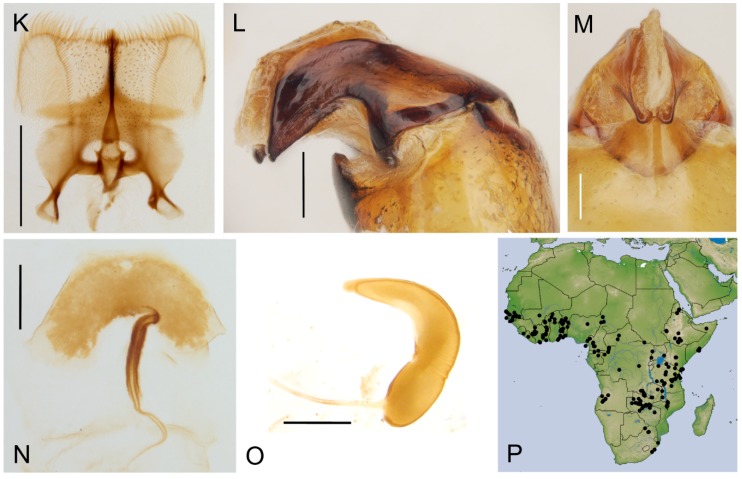
Unique combination of features characterizing *Tiaronthophagus* n.gen. (**A**): *T. naevius*, pronotum (from above): the thick pubescence on the surface and basal glabrous oval area are clearly evident, scalebar = 1 mm. (**B**): *T. aequatus*, pronotum puncture on disc, scalebar = 1 mm. (**C**): *T. ebenus*, head with few large setigerous points on the genae and near the genal sutura, scalebar = 0.1 mm. (**D**): *T. naevius*, pronotum side view, with the glabrous oval area, the corresponding concave area on elytra near the humeral callus (white arrow), and the row of points on the pronotum margin (black arrow), scalebar = 1.0 mm. (**E**): *T. chrysoderus*, elytral stria with the geminate points, scalebar = 0.2 mm. (**F**): *T. rolandoi*, eye with the basal triangular expansion, scalebar = 0.3 mm. (**G**): *T. rufostillans*, female head, vertex lamina, scalebar = 1.0 mm. (**H**): *T. pseudoliberianus*, female side view of the head and pronotum, highlighting the head carinae and the pronotum tubercles, scalebar = 2.0 mm. (**I**): *T. macroliberianus*, major male head, vertex lamina, scalebar = 2.0 mm. (**J**): *T. ebenus*, major male head, vertex lamina, scalebar = 1.0 mm. (**K**): *T. ebenus*, epipharynx, scalebar = 0.5 mm. (**L**): *T. zambesianus*, parameres side view, scalebar = 0.2 mm. (**M**): *T. aequatus*, apices of parameres, and phallobase distal margin, front view, scalebar = 0.2 mm. (**N**): *T. rufobasalis*, vagina infundibular tube, scalebar = 0.2 mm. (**O**): *T. zavattarii*, receptaculum seminis, scalebar = 0.2 mm. (**P**): Map of distribution of *Tiaronthophagus*.

**Figure 7:**
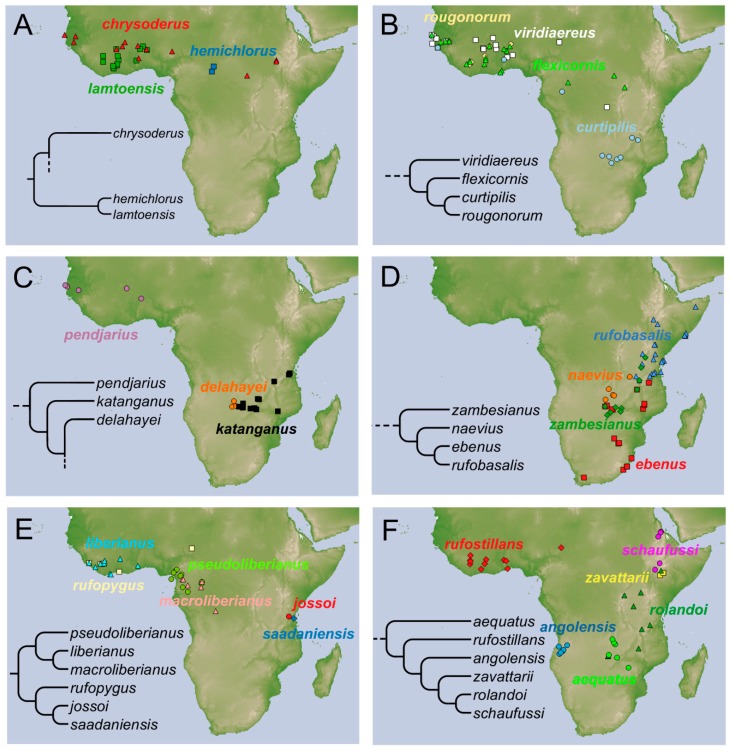
Distribution maps of *Tiaronthophagus* species, highlighting the phylogenetic relationships among them (see Figure 3 for complete tree). (**A**): *T. chrysoderus* (red), *T. hemichlorus* (blue) and *T. lamtoensis* (green). (**B**): *T. rougonorum* (yellow), *T. viridiaereus* (white), *T. flexicornis* (green) and *T. curtipilis* (blue). (**C**): *T. pendjarius* (violet), *T. delahayei* (orange) and *T. katanganus* (black). (**D**): *T. rufobasalis* (blue), *T. naevius* (orange), *T. zambesianus* (green) and *T. ebenus* (red). (**E**): *T. liberianus* (turquoise), *T. rufopygus* (yellow), *T. pseudoliberianus* (green), *T. macroliberianus* (rose), *T. jossoi* (red) and *T. saadaniensis* (blue). (**F**): *T. rufostillans* (red), *T. schaufussi* (violet), *T. zavattari* (yellow), *T. rolandoi* (dark green), *T. angolensis* (blue), *T. aequatus* (light green).

**Diagnosis.** The following synapomorphies combination allow for the recognition of the genus *Tiaronthophagus* among other Onthophagini genera (Figure 6): (1) oval area at base of pronotum on sides near the angles carrying, in the proximal area some long testaceous setae; large, thick, usually short, testaceous pubescence on the remaining surface of the pronotum; the bare area on pronotum corresponds to the large, flattened area on elytra near the humeral callous (Figure 6A,D); (2) pronotum with evident setigerous granulate points on the disc, while on the sides, punctures are larger, more tight, and almost embricate (Figure 6B,D); (3) pronotum at base carrying a row of large, more or less thick, points (Figure 6B,D); (4) elytral striae with large geminate points (Figure 6E), the interstriae with setigerous granulate points; (5) female pronotum usually carried anteriorly on disc, two symmetrical protuberances, triangular-shaped on side view (Figure 6G,H); (6) head genae and clypeus usually carrying few very large setigerous points, sometimes being more evident in major males (Figure 6C); (7) basally, the head carrying a large triangular expansion near the eyes, which are narrow on the whole length (Figure 6F, seen from above); (8) head with a well-developed frontal carina, although sometimes in major males, this can be extremely reduced (Figure 6G–J); (9) head vertex carina present, short in females, and characterized by a marked phenotypic plasticity in males (Figure 6G–J); (10) fore margin of clypeus that is largely upturned, the anterior part of the head being slightly concave (Figure 6G–J); (11) genae rounded and not developed (Figure 6C); (12) testaceous antennae (Figure 6G); (13) head sculpture showing sexual dimorphism, the clypeus of females is rough with a dense and evident sculpture, while in males, it is smoother, often with only some scattered large points (Figure 6G,I,J); (14) major males carrying a well developed, sinuate, and large vertex lamina, corresponding to a large concave area on anterior part of pronotum (Figure 6G,I,J), with the area being far less developed or absent in minor males; (15) epipharynx with a large anterior part, zygum with a tuft of short, thick setae, the crepis being rather underdeveloped (Figure 6K); (16) male parameres down-arched, well developed, the apex sharp and narrow (Figure 6L,M); (17) phallobase distally carrying an expanded sclerotized plate, whih largely extends above the margin (Figure 6L,M); (18) female genitalia, vagina with symmetrical, sclerotized infundibular wall, question-mark shaped, and well-sclerotized infundibular tube (Figure 6N,O), and posteriorly two symmetrical expansions; (19) receptaculum seminis, large at the base, narrowing to the sharp apex, often carrying a sclerotized nail-shaped process (Figure 6O); (20) exclusively Afrotropical distribution (Figure 6P).

At present, only males with a very short vertex carina are included in the typical series of *T. saadaniensis* n.sp. (see the description below), which is known only from the Saadani Park in Tanzania. This species is characterized by some peculiar features: the pronotum is fully covered by large and thick setigerous granules and the lateral areas are smaller than in the other species and covered by smaller granules, but the corresponding flat areas on elytra are nevertheless evident (character 1); the genae and clypeus of the known specimens carry many granulate setigeous points (character 6), while we do not know the features of the head sculpture (character 13) and vertex lamina (character 14) of the major male morph. A similar increasing development of the granulation and pubescence is also present in the sister species *T. jossoi* from Tanzania, although less marked than in *T. saadaniensis*. A differential pattern of morphological variation is thus evident in this clade, although the two species present evident differences in both internal and external morphology.

Noteworthy, the listed above characters can also help in the recognition within *Tiaronthophagus*, with clearly highlighted differences among the species (Supporting Information Data S5 and S6), e.g., in the density, shape, and size of the granulate points of the pronotal disc; position of the large points of the head; density and position of the points on the interstriae; shape of vertex carina (in females) and vertex lamina (in major males), features of the protuberances of pronotum in females (from above); shape of epipharynx, parameres, phallobase sclerotized plate, infundibular wall of vagina, infundibular tube, and posterior expansions of vagina.

#### 3.4.2. The *Tiaronthophagus* Species

At present 26 species are included in the genus *Tiaronthophagus*:

*T. aequatus* (Péringuey, 1901) n.comb.

*T. angolensis* n.sp.

*T. chrysoderus* (d’Orbigny, 1905a) n.comb.

*T. curtipilis* (d’Orbigny, 1905b) n.comb.

*T. delahayei* (Josso, 2011) n.comb.

*T. ebenus* (Péringuey, 1888) n.comb.

*T. flexicornis* (d’Orbigny, 1902) n.comb.

*T. hemichlorus* (d’Orbigny, 1915) n.comb.

*T. jossoi* n.sp.

*T. katanganus* n.sp.

*T. lamtoensis* (Cambefort, 1984) n.comb.

*T. liberianus* (Lansberge, 1883) n.comb.

*T. macroliberianus* (Moretto, 2010) n.comb.

*T. naevius* (d’Orbigny, 1913) n.comb.

*T. pendjarius* (Josso and Prévost, 2006) n.comb.

*T. pseudoliberianus* (Moretto, 2010) n.comb.

*T. rolandoi* n.sp.

*T. rougonorum* (Cambefort, 1984) n.comb.

*T. rufobasalis* (Fairmaire, 1887) n.comb.

*T. rufopygus* (Frey, 1957) n.comb.

*T. rufostillans* (d’Orbigny, 1907) n.comb.

*T. saadaniensis* n.sp.

*T. schaufussi* (Harold, 1867) n.comb.

*T. viridiaereus* (d’Orbigny, 1908) n.comb.

*T. zambesianus* n.sp.

*T. zavattarii* (Müller, 1939) n.comb.

- ***Tiaronthophagus aequatus* (Péringuey, 1901) n.comb.**

*Onthophagus aequatus* Péringuey, 1901: 205.

**Type material. Lectotype.** ♂ (here designated) ‘Salisbury Mashonaland Feb 1899 G. A. K. Marshall in carrion <partly handwritten>’ ‘224 <handwritten>’ ‘*Onthophagus aequatus* ♂ P. type <handwritten>’ ‘Type SAM/Ent 2752’ ‘Lectotype *Onthophagus aequatus* Peringuey, 1901 Roggero, Moretto, Barbero, Palestrini 2019’ ‘*Tiaronthophagus aequatus* (Peringuey, 1901) Roggero, Moretto, Barbero, Palestrini 2019’ (SAMC). **Paralectotypes.** Not yet traced.

**Other material.** BOTSWANA. 10 km NE Martins Drift (PMOC). MALAWI. Blantyre, Michiru Mt. Park (EBCT). TANZANIA. Chimala Escarpment (PMOC); Morogoro Reg., Mikesse Hills, 420 m (PMOC); Mts Uluguru (PMOC). ZAMBIA. Central Province, 25 km NE Lilemone 1250 m (PMOC); 60 km NE Serenje, Bwale (JFJC); Kafue NP, Kacheleko wildlife outpost (JFJC); Kafue NP, Tateyoyo (JFJC); Lower Zambesi NP, 3 km Mukamba gate (JFJC); Lusaka Province, Lower Zambesi NP (JFJC); Mfwanta 1355 m (JFJC); NW Province, 30 km N Lubungu, NE Kafue NP (JFJC); Lusaka (PMOC).

**Description.** Refer to the Supporting Information Data S5 and S6, original description [39], and Onthophagini Synopsis [2].

**Distribution.** The species is known from East and South Central Africa (Figure 7F), extending from Tanzania, Malawi, and Zambia, until the *locus typicus* in Zimbabwe.

**Notes**. In the SAMC a single specimen is housed, labelled as “type”. It is possible that other specimens had originally been included in the type series, since, in the original description, by Péringuey it is written “length 10–10 1/2 mm”, which likely means that the author had more than one specimen. Furthermore, in the last sentence of the description, there is written “In both this.... the punctures or the granules... are occasionally less numerous in the second interval”, meaning that there was at least another type specimen in the Péringuey collection. Thus, here we can account only for the lectotype, while any paralectotype has not yet been found.

- ***Tiaronthophagus angolensis* n.sp.**

Zoobank Registration: http://zoobank.org/urn:lsid:zoobank.org:act:CD48985C-28BA-4DEF-8C60-7EABF5FD51BC.

**Figure 8:**
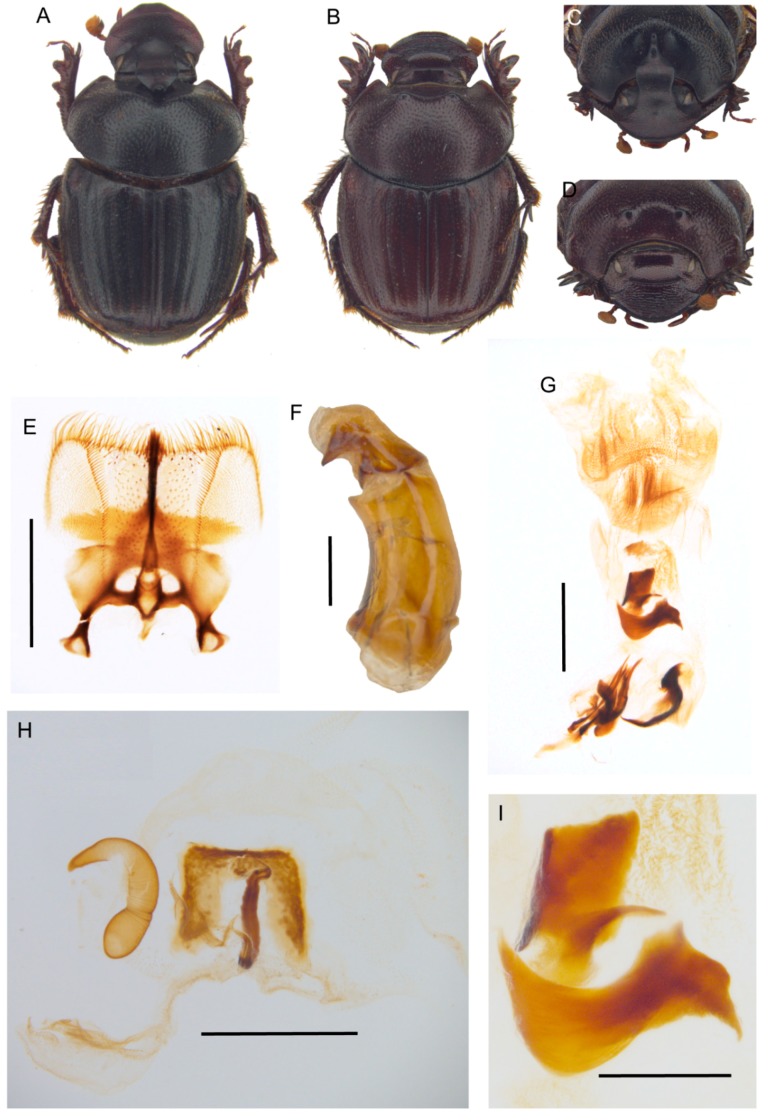
Features of *Tiaronthophagus angolensis* n.sp. (**A**): Male facies (above). (**B**): Female facies (above). (**C**): Male facies (frontal view). (**D**): Female facies (frontal view). (**E**): Epipharynx, scalebar = 0.5 mm. (**F**): Male genitalia, phallobase and parameres, scalebar = 0.5 mm. (**G**): Male genitalia, endophallus, scalebar = 0.5 mm. (**H**): Female genitalia, vagina and receptaculum seminis, scalebar = 0.5 mm. (**I**): male genitalia, lamella copulatrix, scalebar = 0.2 mm.

Figure 7 and Figure 8, Supporting Information Data S5 and S6.

**Type material. Holotype.** ♂ ‘ANGOLA, Huila, 3.5 km SW Negola, 14°08′53″ S, 14°28′16″ E 29.xi.2012 open forest, human shit P. Schüle leg. #10’ ‘*Tiaronthophagus angolensis* n.sp. holotype’ (BMNH). **Paratypes.** 7♂ 5♀ ‘ANGOLA, 8.xii.2012, Huila Prov. 3.5 km SW Negola, 14°28′16″ S, 14°08′53″ E, P. Schule leg. #22’ ‘*Tiaronthophagus angolensis* n.sp. paratype’ (PMOC); 1♂ 2♀ ‘ANGOLA, prov. Huila, 10 km SW Cacula, 4–6.xi.2011 P. Schule leg.’ ‘*Tiaronthophagus angolensis* n.sp. paratype’ (PMOC); 1♂ ‘5.xii.2012 env. Aldeia Cambala (S. Cacula) Huambo prov. Angola S. Rojkoff réc.’ ‘*Tiaronthophagus angolensis* n.sp. paratype’ (PMOC); 1♂ ‘6-xii-2012 PL 75 km N Cacula 13°26′192″ S, 15°22′755″ E, Huíla prov. Angola S. Rojkoff det.’ ‘Onthophagus cfr schaufussi Harold M. Dierkens det. 2013’ ‘*Tiaronthophagus angolensis* n.sp. paratype’ (PMOC); 1♂ 1♀ ‘6-xii-2012 PL 75 km N Cacula 13°26′192″ S, 15°22′755″ E, Huíla prov. Angola S. Rojkoff det.’ ‘*Tiaronthophagus angolensis* n.sp. paratype’ (MDCL); 2♂ 1♀ ‘29.xi.2012 PL 2 km S Negola 14°28′16″ E, 14°8′53″ S, 9.xii.2012 Huíla prov. Angola S. Rojkoff rec.’ ‘*Tiaronthophagus angolensis* n.sp. paratype’ (JFJC); 1♂ ‘ANGOLA, 19.xi.13, Malanje Prov. 20 km SE Cacandala, 1109 m 9°48′873″ S, 16°36′398″ E, P. Schule leg. #13’ ‘*Tiaronthophagus angolensis* n.sp. paratype’ (PMOC); 17♂ 7♀ ‘ANGOLA, Huila 3.5 km SW Negola 14°8′53″ S, 14°28′16″ E, 8.xii.2012, P. Schule leg. #22’ ‘*Tiaronthophagus angolensis* n.sp. paratype’ (PMOC); 1♀ ‘ANGOLA, Huila, 3.5 km SW Negola, 14°8′53″ S, 14°28′16″ E, 29.xi.2012 open forest, human shit P. Schüle leg. #10’ ‘*Tiaronthophagus angolensis* n.sp. paratype’ (PMOC); 1♀ ‘ANGOLA Huila, 75 km NE Caconda, 5-xii-2012, 13°26′19″ S, 15°22′55″ E, P. Schüle leg. #20/21’ ‘*Tiaronthophagus angolensis* n.sp. paratype’ (PMOC); 1♂ 1♀ ‘ANGOLA, 11–12.xi.2013, Huila Prov. 3.5 km SW Negola, 14°28′16″ S, 14°8′53″ E, P. Schüle leg. #4’ ‘*Tiaronthophagus angolensis* n.sp. paratype’ (PMOC); 1♂ ‘13.xi.13, Huíla Prov., near Nova Monção, 1489 m 13°30′292″ S 15°12′746″ E, P. Schule leg. WP 6 5 ‘*Tiaronthophagus angolensis* n.sp. paratype’ (PMOC); 1♂ 12.xii.2015, Huila prov, 3 km SW Nova Monção, 13°26.612 S, 5°21.881 E. G.Werner leg. ‘*Tiaronthophagus angolensis* n.sp. paratype’ (KWC): 1♂ 6♀ ‘Huila prov. 3.5 km SW Negola, 14°08′53″ S, 14°28′16″ E, 29.xi.2012 open forest, human shit P. Schule leg. #10’ ‘*Tiaronthophagus angolensis* n.sp. paratype’ (PMOC).

**Other material.** No other material is known at present.

**Etymology.** The species was named after the locality of the type material collection.

**Description.** Male (Figure 8A,C): length 10–13 mm. Body black, mat, surface covered by short and thick testaceous pubescence, longer on sides and legs. Head surface smooth, with many small points, and some larger ones near the genae suturae; frontal carina barely visible in major males, rounded, and evident in minor males; genae rounded, not much developed; vertex carina in major male lamina shaped, elongate, with a digitiform expansion apically, in minor male reduced to a short expansion truncated at apex. Antennae yellowish brown. Pronotum ovalar, enlarged, covered with granulate setigerous points, granules large and rounded, base with a row of large points, anteriorly a large, concave area in major males, and two triangular protuberances on disc in minor males. Elytra striae geminate, interstriae covered by setigerous points that are regularly spaced on the surface. Pygidium black, mat with dense, superficial puncture. Legs black with long, testaceous setae.

Female (Figure 8B,D): length 11–12 mm. Body black and mat as in male. Head surface wrinkled with some few large points near the genal suturae on clypeus, smooth with many small simple points on the remaining surface; frontal carina rounded, evident; genae rounded, not developed; vertex carina tricuspid, large and low, rectilinear from above view. Pronotum granulation as in male, with two evident conical tubercles on disc. Elytra and legs as in male. Pygidium is shorter than in male, but the same colour and puncture.

Epipharynx (Figure 8E) fore margin slightly concave, with evenly long setae, chaetopariae angular, with setae very short in basal half; anterior epitorma rod-shaped, thin, well sclerotized; the zygum is constituted by long setae, proplegmatium triangular, low, the sides thick and rounded; apotormae present, thin, rod-like; plegmatic area present, ovalar; pternotormae short, thick and rectilinear; laeotorma and dexiotorma symmetrical, short; crepis only slightly asymmetrical, being pointed at apex.

Male genitalia (Figure 8F,G,I): phallobase cylindrical, with a rectangular expansion ventrally; parameres symmetrical, tapering to apex, arched, the base diameter smaller than phallobase; endophallus with a large denticulate area, and an evident raspula being constituted by long, thin setae; lamella copulatrix present, constituted by two well-sclerotized parts (Figure 8I); accessory lamellae present, well-developed (Figure 8G).

Female genitalia (Figure 8H): vagina domed, membranous; infundibular wall reversed U-shaped, apically squared; well-sclerotized infundibulum question mark that is shaped toward the receptaculum seminis, rectilinear in central portion, and upward turned toward the ovarioles; receptaculum seminis not much expanded, lobate, tapering to apex carrying a small, rounded laminal claw, the desclerotized area small and near the apex.

**Distribution.** The species is known only from Angola (Figure 7F).

- ***Tiaronthophagus chrysoderus* (d’Orbigny, 1905) n.comb.**

*Onthophagus chrysoderus* d’Orbigny, 1905a: 490.

**Type material. Holotype.** ♀ (by monotypy), ‘Sénégal’ ‘chrysoderus n.sp. D’Orbigny’ ‘Holotype *Onthophagus chrysoderus* d’Orbigny, 1905 Roggero, Moretto, Barbero, Palestrini 2019’ ‘*Tiaronthophagus chrysoderus* (d’Orbigny, 1905) Roggero, Moretto, Barbero, Palestrini 2019’ (BMNH).

**Other material.** BENIN. North Eastern, E of Kandi, around Saa (PMOC). BURKINA FASO. Bobo Diulasso, Farako Ba (PMOC); Mogtedo, piste de Bomboré (PMOC); Passoré 8 km SW Yako 320 m (PMOC); Sanguié, Forêt de Sorobouli, 270 m (PMOC). DEMOCRATIC REPUBLIC OF CONGO. Haut Uele env., Doruma (NHMW). ETHIOPIA. Dembi (= Dembidolo), Illubabor (PMOC); Gambela (PMOC). GUINEA. Région Kindia, Mt Gangan, 500 m (BMNH). GUINEA BISSAU. Coli (Quebo), Tombali (CAS). NIGERIA. north Lama Burra Game Reserve 630 m. (PMOC). SENEGAL. Kolda, Mahon Forêt de Bakor (PMOC); Sebikotane (MNHN). TOGO. Sokodé (NHMW).

**Description.** Refer to the Supporting Information Data S5 and S6, original description [40], and Onthophagini Synopsis [2].

**Distribution.** The species is known from the Western and Central Africa (Figure 7A).

- ***Tiaronthophagus curtipilis* (d’Orbigny, 1905b: 440) n.comb.**

*Onthophagus curtipilis* d’Orbigny, 1905b: 440.

= *Onthophagus altidorsis* d’Orbigny, 1905b: 443. Josso and Moretto, 2018: 11.

**Type material. Holotype.** ♀ (by monotypy) ‘Guinea Portoghese Bolama vi–xii.1899. L.Fea’ ‘Typus’ ‘curtipilis n.sp. D’Orb. <handwritten by d’Orbigny>’ ‘curtipilis d’Orb. <handwritten>’ ‘Holotypus *Onthophagus curtipilis* d’Orbigny, 1905’ ‘Mus. Civ. Genova’ ‘*Tiaronthophagus curtipilis* (d’Orbigny, 1905) Roggero, Moretto, Barbero, Palestrini 2019’ (MSNG).

**Synonymy type material. *O. altidorsis.*** ♂ (holotype by monotypy) ‘Guinea Portoghese Bolama vi–xii.1899. L.Fea’ ‘Typus’ ‘altidorsis n.sp. D’Orb. <handwritten by d’Orbigny>’ ‘altidorsis d’Orb. <handwritten>’ ‘Holotypus Onthophagus altidorsis d’Orbigny, 1905’ ‘Mus. Civ. Genova’ ‘*Tiaronthophagus curtipilis* (d’Orbigny, 1905) Roggero, Moretto, Barbero, Palestrini 2019’ (MSNG).

**Other material.** BURKINA FASO. Sanguié, Boromo, Ft. de Sorobouli; Ouagadougou, la forêt; Bobodiolasso, Farako-Bâ (PMOC). CAMEROON. N.W. Province, Bankim (PMOC). CENTRAL AFRICAN REPUBLIC. Ombella-Mpoko Prov., 20 km N.W. Yaloke (PMOC). GHANA. Kyabobo N.P.; Great Accra Region, Shai Hills (PMOC). GUINEA BISSAU. Coli (Quebo), Tombali (CAS). IVORY COAST. Comoé, Zamou; P.N. du Mt. Sangbé; Korhogo, Koko; Touba, Biémasso; Odienné, Massif du Denguélé (PMOC). MALAWI. Northern, Vwaza Marsh Reserve, Rumphi district, 1150 m (JFJC). REPUBLIC OF THE CONGO. Odzala N. P. (JFJC). SENEGAL. Tambacounda, Niokolo-Koba N.P.; Kolda, Mahon, Forêt de Bakor; Kédougou, 5 km S. Ségou. (PMOC). TANZANIA. Mwanza region, Geita (JFJC); Morogoro Reg., Mbarika Mts. (Ikongwa) (PMOC). TOGO. Fazao (NHMW). ZAMBIA. 17 km N Choma I. Bruce-Miller farm (JFJC); Kafue N. P., Kacheleko wildlife outpost (JFJC); Kafue river east (JFJC); Lower Zambesi N. P., 3 km Mukamba gate (JFJC); Southern, Mwense Muchinga 3620Ft. (JFJC); Western, 14 km SW Kaoma, 1180 m (JFJC).

**Description.** Refer to the Supporting Information Data S5 and S6, original description [41], and Onthophagini Synopsis [2].

**Distribution.** The species is widespread from Guinea Bissau (the locus typicus) to Togo and Zambia (Figure 7B).

**Notes.** The species *T. curtipilis* was described by a single female specimen, while *O. altidorsis* was described by a single male specimen from the same locality [42].

- ***Tiaronthophagus delahayei* (Josso, 2011) n.comb.**

*Onthophagus delahayei* Josso, 2011: 4.

**Type material. Holotype.** ♂ ‘Zambie 18 km W Kaoma 14°52′0.3″ S, 24°38′29.4″ E, 9.xii-2008 JF Josso R Minetti leg’ ‘Exc. humains’ ‘holotype *Onthophagus delahayei* n.sp. J-F Josso det 2010’ ‘*Tiaronthophagus delahayei* (Josso, 2011) Roggero, Moretto, Barbero, Palestrini 2019’ (JFJC). **Paratypes**. 6♂ 6♀ ‘Zambie 18 km W Kaoma 14°52′0.3″ S, 24°38′29.4″ E, 9.xii-2008 JF Josso R Minetti leg’ ‘Exc. humains’ ‘paratype *Onthophagus delahayei* n.sp. J-F Josso det 2010’ ‘*Tiaronthophagus delahayei* (Josso, 2011) Roggero, Moretto, Barbero, Palestrini 2019’ (JFJC); 12♂ 9♀ ‘Zambie 18 km W Kaoma 14°52′0.3″ S, 24°38′29.4″ E, 14–15.xii-2009 Josso Juhel Minetti leg’ ‘Exc humains’ ‘paratype *Onthophagus delahayei* n. sp J-F Josso det 2010’ ‘*Tiaronthophagus delahayei* (Josso, 2011) Roggero, Moretto, Barbero, Palestrini 2019’ (JFJC) (PMOC) (PPRC) (IRSNB) (RMIC); 1♂ 1♀ ‘Zambie (E Mongu) piste de Ndanda 15°7′25.4″ S, 23°41′23.1″ E, 10-XII-2008 JF Josso R Minetti leg’ ‘Exc humains’ ‘*Tiaronthophagus delahayei* (Josso, 2011) Roggero, Moretto, Barbero, Palestrini 2019’ (JFJC); 4♂ 2♀ ‘Zambie 3 km NW Kandunda 1120 m 14°50′19″ S, 24°38′23″ E, 8-XII-2009 M. François leg.’ ‘Exc. humains’ ‘*Tiaronthophagus delahayei* (Josso, 2011) Roggero, Moretto, Barbero, Palestrini 2019’ (MFRC).

**Other material.** ZAMBIA. North Western province, 60 km N Kabompo (PMOC).

**Description.** Refer to the Supporting Information Data S5 and S6 and original description [43].

**Distribution.** At present the species is known only from Zambia (Figure 7C).

**Notes**. The species was originally included in 16th group, which was based on the features of the pronotum posterior margin that is not wholly re-bordered on sides. However, the author also stated that the species is different from any other one of the group.

- ***Tiaronthophagus ebenus* (Péringuey, 1888) n.comb.**

*Onthophagus ebenus* Péringuey, 1888: 97.

*= Onthophagus natalicus* d’Orbigny, 1902: 177. D’Orbigny, 1903: 296.

= *Onthophagus bituber* d’Orbigny, 1904: 289, **n.syn.**

**Type material. Lectotype.** ♂ (here designated) ‘Durban Natal <handwritten by Péringuey>‘ ‘Type SAM/Ent 2753b’ ‘*Onthophagus ebenus* Pe. type ♂ <handwritten by Péringuey>‘ ‘Lectotype *Onthophagus ebenus* Peringuey, 1888 Roggero, Moretto, Barbero, Palestrini 2019’ ‘*Tiaronthophagus ebenus* (Peringuey, 1888) Roggero, Moretto, Barbero, Palestrini 2019’ (SAMC). **Paralectotypes.** ♀ ‘*Onthophagus ebenus* Pe. type <handwritten by Péringuey>‘ ‘Kipibethe 1884 <handwritten by Péringuey>’ ‘Type SAM/Ent 6008’ ‘Imaged LAS 4.9 SAMC 2017’ ‘Paralectotype *Onthophagus ebenus* Peringuey, 1888 Roggero, Moretto, Barbero, Palestrini 2019’ ‘*Tiaronthophagus ebenus* (Peringuey, 1888) Roggero, Moretto, Barbero, Palestrini 2019’ (SAMC); ♀ ‘Durban Natal <handwritten by Péringuey>‘ ‘Type SAM/Ent 2753a’ ‘*Onthophagus ebenus* Pe. type ♀ <handwritten by Péringuey>‘ ‘Imaged LAS 4.9 SAMC 2017’ ‘Paralectotype *Onthophagus ebenus* Peringuey, 1888 Roggero, Moretto, Barbero, Palestrini 2019’ ‘*Tiaronthophagus ebenus* (Peringuey, 1888) Roggero, Moretto, Barbero, Palestrini 2019’ (SAMC).

**Synonymy type material. *O. natalicus.*** ♂ (lectotype) ‘Natal Dr Martin’ ‘*Tiaronthophagus ebenus* (Peringuey, 1888) Roggero, Moretto, Barbero, Palestrini 2019’ (MNHN); (2 paralectotypes) ‘Natal Dr. Martin’ ‘*Tiaronthophagus ebenus* (Peringuey, 1888) Roggero, Moretto, Barbero, Palestrini 2019’ (MNHN); (3 paralectotypes) ‘Durban, Natal’ ‘*Tiaronthophagus ebenus* (Peringuey, 1888) Roggero, Moretto, Barbero, Palestrini 2019’ (MNHN); (paralectotypes) Transvaal, Makapan near Pietersburg (MNHN). ***O. bituber***. ♀ (holotype by monotypy), “Botoka Feb. 98. <handwritten>” “bituber n.sp. d’Orb. <handwritten>” “d’Orbigny vidit 1904” “holotype” ‘Holotype *Onthophagus bituber* d’Orbigny, 1904 Roggero, Moretto, Barbero, Palestrini 2019’ ‘*Tiaronthophagus ebenus* (Peringuey, 1888) Roggero, Moretto, Barbero, Palestrini 2019’ (MNHN).

**Other material.** BOTSWANA. 10 km NE Martins Drift (PMOC). MALAWI. Liwonde Nat. Park (JFJC); Northern, Vwaza Marsh Reserve, Rumphi district, 1150 m (JFJC); Mzimba district, Vwanza Marsh Game res. 1150 m (JFJC); Blantyre Michiru Mt Park (PMOC); Southern, gorges de la Mpatamaga (JFJC). SOUTH AFRICA. KwaZulu Natal, 3 km S Nyamande Kwa Gudlumfula Mts. 350 m (JFJC) (MDCL); KwaZulu Natal, 10 km N Hluhluwe (PMOC); KwaZulu Natal, Durban (PMOC); KwaZulu-Natal, 4.5 km NW Gudlucingu Umzimkulu river (JFJC); Limpopo, Makopane 1395 m Shikwaru lodge (PMOC); Eastern Cape, 3 km N Qhaka, 138 m near Lusikisiki (JFJC). TANZANIA. Morogoro region, 360 m, Mbarika Mts, (Ikongwa) between Kichangari and Idunda (PMOC). ZAMBIA. Kafue NP, Chunga (JFJC); Kafue NP, Mayukuyuku Camp (JFJC); Magoye (PMOC); Kafue env. (PMOC); Kafue (MDCL); Southern province, Kafue, Chiron du Mts (MDCL); Southern province, 11 km E Mukuni, 960 m (MDCL); Southern province, Mosi Oa Tunya NP (JFJC).

**Description.** Refer to the Supporting Information Data S5 and S6, original description [44] and Onthophagini Synopsis [2].

**Distribution.***Onthophagus ebenus* was described by Vaal River (Mpumalanga), as quoted by Péringuey, while *Onthophagus natalicus* was described from Natal, Durban, Transvaal, and Makapan near Pietersburg. Besides, in the SAMC there are three specimens labelled as ‘*Onthophagus ebenus* type’. The species is widespread in Southeastern Africa (Figure 7D) from Tanzania (Morogoro region) to South Africa (KwaZulu Natal).

**Notes**. The synonymy of *T. ebenus* and *O. natalicus* was confirmed by the survey of the typical material of the latter species. The comparison of the type material of *O. ebenus* and *O. bituber*, shows that the specimens belong to a single species. Thus, we propose the new synonymy. The type locality of *T. ebenus* was defined according to the ICZN (articles 74E, 76.2).

- ***Tiaronthophagus flexicornis* (d’Orbigny, 1902) n.comb.**

*Onthophagus flexicornis* d’Orbigny, 1902: 178.

= *Onthophagus atroaereus* d’Orbigny, 1908: 164 **n.syn.**

**Type material.Lectotype.** ♂ (here designated) ‘Accra <written by hand> Ex. Musaeo H.W. Bates1892’ ‘flexicornis n.sp. D’Orb. <d’Orbigny handwritten> ‘d’Orbigny Ontho. Afr. 1902’ ‘Muséum Paris 1952 Coll. R.Oberthür’ ‘LECTOTYPE’ ‘Lectotype *Onthophagus flexicornis* d’Orbigny, 1902 Roggero, Moretto, Barbero, Palestrini 2019’ ‘*Tiaronthophagus flexicornis* (d’Orbigny, 1902) Roggero, Moretto, Barbero, Palestrini 2019’ (MNHN). **Paralectotypes.** 1♀’Accra W. Afr. <written by hand> Ex. Musaeo H.W.Bates 1892’ ‘d’Orbigny Ontho. Afr. 1902’ ‘Muséum Paris 1952 Coll. R.Oberthür’ ‘PARALECTOTYPE’ ‘Paralectotype *Onthophagus flexicornis* d’Orbigny, 1902 Roggero, Moretto, Barbero, Palestrini 2019’ ‘*Tiaronthophagus flexicornis* (d’Orbigny, 1902) Roggero, Moretto, Barbero, Palestrini 2019’ (MNHN); 1♀ ‘Accra <written by hand> Ex. Musaeo H.W.Bates 1892’ ‘Muséum Paris Coll. H. d’Orbigny 1915’ ‘PARALECTOTYPE’ ‘Paralectotype *Onthophagus flexicornis* d’Orbigny, 1902 Roggero, Moretto, Barbero, Palestrini 2019’ ‘*Tiaronthophagus flexicornis* (d’Orbigny, 1902) Roggero, Moretto, Barbero, Palestrini 2019’ (MNHN).

**Synonymy type material. *O. atroaereus*.** ♀ (holotype by monotypy) ‘Guinée Portug. Chime 1906 Rio Geba G.Favarel’ ‘atroaereus n.sp. D’Orb. <d’Orbigny handwritten>‘ ‘d’Orbigny Ontho. Afr. 1908’ ‘Muséum Paris 1952 Coll. R.Oberthür’ ‘HOLOTYPE’ ‘*Tiaronthophagus flexicornis* (d’Orbigny, 1902) Roggero, Moretto, Barbero, Palestrini 2019’ (MNHN).

**Other material.** BENIN. Northern, Parc Pendjari, Tanoungou (JFJC); Parc Pendjari, vers hotel Pendjari (JFJC). BURKINA FASO. Kompienga Pama, 230 m (PMOC); Sanguié Boromo forêt de Sorobouli 270 m (PMOC); Park W, 7km Koudou (vers Kondjo) (JFJC). CENTRAL AFRICAN REPUBLIC Bozo (PMOC); North, Parc de la Sangha (MHNL). DEMOCRATIC REPUBLIC OF CONGO. Haut-Uele, Moto (MNHN); Haut-Uele env. Doruma (NHMW). GHANA. Great Accra Region, Hills north end, 125 m (PMOC). GUINEA. Nimba (MNHN). IVORY COAST. Zamou (PMOC); Comoé, Zamou (PMOC). Korhogo Koko 347 m (PMOC); PN Comoé (MHNL); Touba (PMOC); Touba, Biémasso-Dolla (PMOC); Touba, Dolla (PMOC); Youho (MHNL). SENEGAL. Niokolo Koba NP, Niokolo (PMOC); Kolda Mahon, Forêt de Bakor (PMOC); Niokolo-Koba NP, poste de Niokolo 65m (PMOC); Niokolo-Koba NP poste de Siminti (PMOC); Sangalkam (MNHN); Sebikotane (MNHN); Tambacounda Botou, 52 m (PMOC).

**Description.** Refer to the Supporting Information Data S5 and S6, original description [45], and Onthophagini Synopsis [2].

**Distribution.** The species is widespread in Western Africa until the Democratic Republic of Congo (Figure 7B).

**Notes.** The comparison of the type material of the two species, in addition with series of recently collected material shows that all of the specimens belong to a single species. Thus, we propose the new synonymy.

- ***Tiaronthophagus hemichlorus* (d’Orbigny, 1915) n.comb.**

*Onthophagus hemichlorus* (d’Orbigny, 1915: 392.

**Type material. Holotype.** ♂ (by monotypy) ‘haut Chari Fort-Crampel coll. Felsche’ ‘*Tiaronthophagus hemichlorus* (d’Orbigny, 1915) Roggero, Moretto, Barbero, Palestrini 2019’ (MNHN, not yet traced).

**Other material.** CENTRAL AFRICAN REPUBLIC. Kemo prov., 45 km N Sibut, 550 m (JFJC).

**Description.** Refer to the Supporting Information Data S5 and S6 and original description [46].

**Distribution.** Described from Haut-Chari, Fort-Crampel (now, Kaga-Bandoro) in Central African Republic (Figure 7A).

**Notes.** The species was described from a single specimen [46] after the publication of the Synopsis [2] and included in the 24th species-group. The species is given, as strictly related to *T. schaufussi* and *T. ebenus*.

- ***Tiaronthophagus jossoi* n.sp.**

Zoobank Registration: http://zoobank.org/urn:lsid:zoobank.org:act:087184D9-01BA-484B-9252-411F6E4FDC5D.

**Figure 9:**
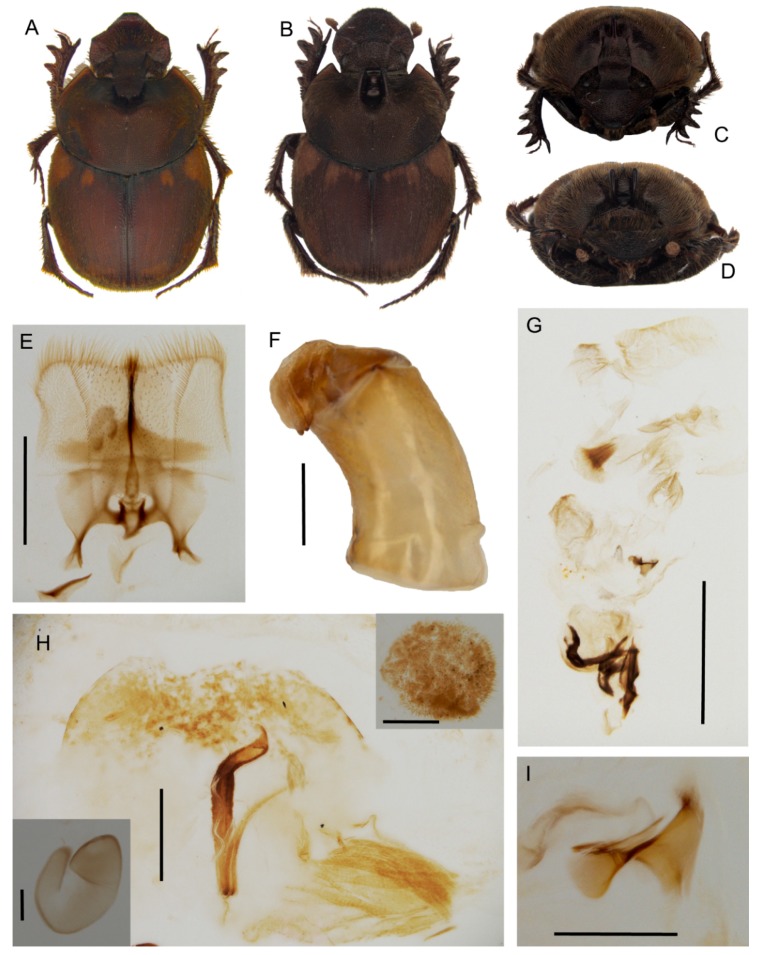
Features of *Tiaronthophagus jossoi* n.sp. (**A**): Male facies (above). (**B**): Female facies (above). (**C**): Male facies (frontal view). (**D**): Female facies (frontal view). (**E**): Epipharynx, scalebar = 0.5 mm. (**F**): Male genitalia, phallobase and parameres, scalebar = 0.5 mm. (**G**): Male genitalia, endophallus, scalebar = 1.0 mm. (**H**): Female genitalia, vagina, scalebar = 0.2 mm, with the detail of posterior expansion, scalebar = 0.1 mm, and receptaculum seminis, scalebar = 0.1 mm. (**I**): Male genitalia, lamella copulatrix, scalebar = 0.2 mm.

Figure 7 and Figure 9, Supporting Information Data S5 and S6.

**Type material. Holotype.** ♂ ‘Tanzanie, VII.2005 Nguu Mts., Tamota Fst. Local coll. > R. Minetti’ ‘*Tiaronthophagus jossoi* n.sp. holotype’ (BMNH). **Paratypes.** 3♂, 3♀ ‘Tanzanie, VII.2005 Nguu Mts., Tamota Fst. Local coll. > R. Minetti’ ‘*Tiaronthophagus jossoi* n.sp. paratype’ (PMOC); 5♂ ‘Morogoro Region Udzungwa National Park campsite 2, forêt, altitude 390 m., 7°50.743′ S, 36°52.584′ E, ‘*Tiaronthophagus jossoi* n.sp. paratype’ (PMOC).

**Other material.** No other material is known at present.

**Etymology.** The species was named after our colleague, the French entomologist J.F. Josso.

**Description.** Male (Figure 9A,C): length 11–14 mm. Body dark reddish brown with some symmetrical testaceous patches on elytra, relatively flat, mat, densely granulate, with a long testaceous pubescence on the whole surface. Head pentagonal, fore margin of the clypeus upward-turned, surface that is covered by many very thick large points, which are fewer and mixed to smaller ones in minor males, genae not much developed, squared; frontal carina being barely visible in major males, almost rectilinear; vertex carina in major male modified into a long, flat rectangular lamina with apical sides elongate and sharp, digitiform expansion that is not much developed, rectilinear, while in minor male is a small triangular expansion. Eyes slightly rounded and medium sized. Antennae yellowish brown. Pronotum with small dense granules, anterior angles with a testaceous longitudinal patch, base with a dense row of large points. Elytra rounded, wider than pronotum, with two symmetrical patches on the third and 5–6th interstriae at an anterior margin near the humeral callus and a larger one at posterior margin of elytra. Pygidium greenish brown, with a thick, long yellow pubescence, rounded small relatively dense granules on whole surface. Legs reddish brown with long, thick testaceous setae.

Female (Figure 9B,D): length 12–14 mm. Body reddish brown as in male. Head pentagonal, but more rounded, and less developed anteriorly than in males; the whole surface being covered by a rough granulation, with many large points; frontal carina evident, elevated, almost rectilinear; genae not much expanded, rounded; vertex carina small, short, triangular shaped. Pronotum with two symmetrically large expansions, triangular lamina shaped on side view, encircling a smooth, concave narrow area without pubescence. Elytra and legs as in male. Pygidium is shorter than in male, but the same colour and puncture.

Epipharynx (Figure 9E) fore margin rectangular shaped, acropariae with a thick long pubescence, zygum constituted by a thick tuft of setae; anterior epitorma thin but well-sclerotized, basal part enlarged, drop shaped, proplegmatium triangular-shaped, lowered, sides thick and rounded; apotormae present, triangular-shaped; plegmatic area ovalar, small; pternotormae thick, short, downturned; laeotorma and dexiotorma short, thin, symmetrical; crepis well developed, apex left-turned; chaetopariea rectilinear, the setae longer in the distal half, then very small; haptomerum with short thick setae that are mixed to very short, thinner ones.

Male genitalia (Figure 9F,G,I): phallobase short and large, with a rectangular expansion anteriorly; parameres with triangular apices; endophallus raspula that are constituted by a fringe of very long thin setae, lamella copulatrix present, saddle shaped (Figure 9I); accessory lamellae present, well-developed.

Female genitalia (Figure 9H): vagina dome-shaped, with two spherical expansions that are covered by thick and short setae on sides; infundibular wall with a semiovalar sclerotization; infundibulum question mark shaped, the part toward the ovarioles upward turned; receptaculum seminis lobate, tapering to the apex, the small sclerotized area near the apex.

**Distribution.** The species is known at present from Tanzania (Figure 7E).

- ***Tiaronthophagus katanganus* n.sp.**

Zoobank Registration: http://zoobank.org/urn:lsid:zoobank.org:act:222AB909-F9A6-4FE0-923A-8B08E8B2D1C6.

**Figure 10:**
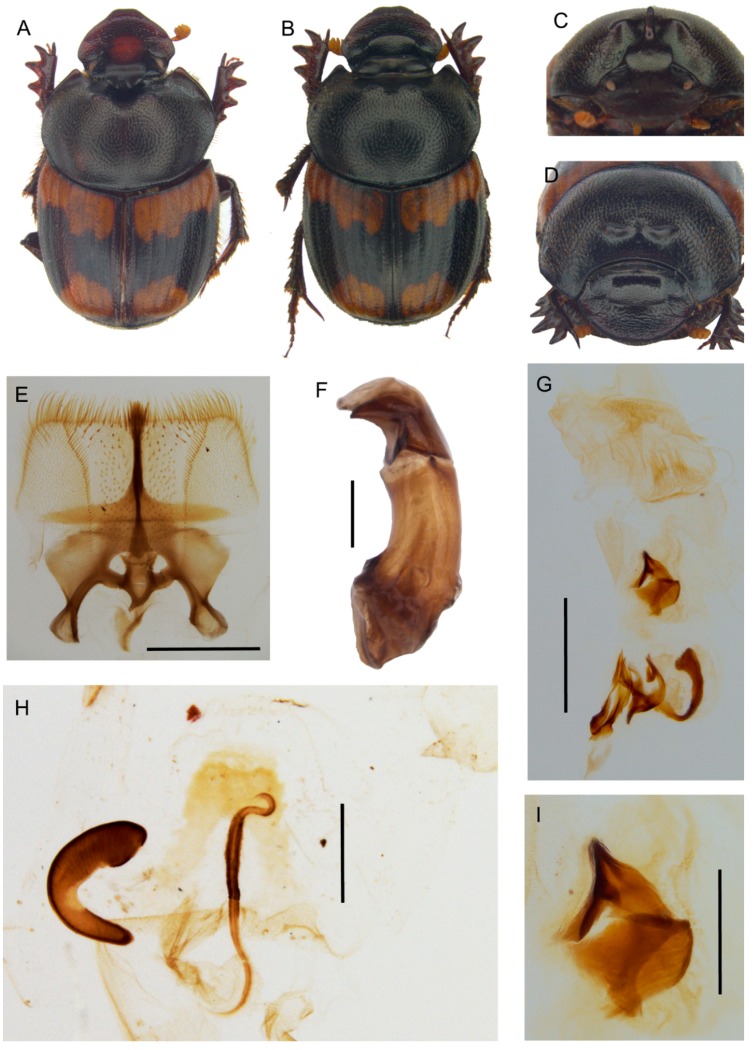
Features of *Tiaronthophagus katanganus* n.sp. (**A**): Male facies (above). (**B**): Female facies (above). (**C**): Male facies (frontal view). (**D**): Female facies (frontal view). (**E**): Epipharynx, scalebar = 0.5 mm. (**F**): Male genitalia, phallobase and parameres, scalebar = 0.5 mm. (**G**): Male genitalia, endophallus, scalebar = 0.5 mm. (**H**): Female genitalia, vagina and receptaculum seminis, scalebar = 0.5 mm. (**I**): Male genitalia, lamella copulatrix, scalebar = 0.2 mm.

Figure 7 and Figure 10, Supporting Information Data S5 and S6.

**Type material. Holotype.** ♂ ‘R. D. Congo, Katanga, Mwera i/ii.2001 coll. T. Bouyer’ ‘*Onthophagus aequatus* Peringuey J.L Nicolas det. 2001 <handwritten>’ ‘aequatus Per. <handwritten>’ ‘*Tiaronthophagus katanganus* n.sp. holotype’ (BMNH). **Paratypes.** 2♀ ‘R. D. Congo, Katanga, Mwera i/ii.2001 coll. T. Bouyer’ ‘*Tiaronthophagus katanganus* n.sp. paratype’ (PMOC); 1♂ ‘R. D. Congo Katanga Kasumbalesa 13.xii.01 coll. T. Bouyer’ ‘*Tiaronthophagus katanganus* n.sp. paratype’ (JFJC); 1♀ ‘R. D. Congo Katanga Lubumbashi 20-xi/13-xii-2002 coll. T. Bouyer’ ‘*Tiaronthophagus katanganus* n.sp. paratype’ (PMOC); 1♂ 2♀ ‘Zambie, 18.xii.2006 Lusaka C.diGennaro leg.’ ‘*Tiaronthophagus katanganus* n.sp. paratype’ (PMOC); 1♂ ‘Zambie, 14.xii.2006 Lusaka C. di Gennaro leg.’ ‘*Tiaronthophagus katanganus* n.sp. paratype’ (PMOC); Zambia 11.xi.2002 50–150 km S Kasempa Werner leg.’ ‘*Tiaronthophagus katanganus* n.sp. paratype’ (PMOC); 2♀ ‘Zambie NW Prov. 30km N Lubungo NE Kafue NP 10-11xii2012 14°11′ S, 26°29′ E, 3775Ft leg. Josso Juhel Minetti’ ‘lumiere’ ‘*Tiaronthophagus katanganus* n.sp. paratype’ (JFJC); 2♀ ‘22-xi-2003 Zambia N W prov.’ ‘Mufumbwe to Kasempa Werner et SMRZ’ ‘*Onthophagus rufobasalis* Fairmaire M. Dierkens det. 04’ ‘*Tiaronthophagus katanganus* n.sp. paratype’ (MDCL); 1♂ ‘Zambie Monze 7-10-xi-2010 J-F Josso leg’ ‘*Tiaronthophagus katanganus* n.sp. paratype’ (JFJC).

**Other material.** No other material is known at present.

**Etymology.** The species was named after the collection locality of the holotype.

**Description.** Male (Figure 10A,C): length 10–11 mm. Body blackish brown, which is covered by a short testaceous pubescence, with two large testaceous patches at base and apex of elytra, extending from elytral sutura to side margin. Head surface smooth covered by dense small points mixed to larger ones near the genal suturae; frontal carina sometimes almost inapparent in major males, slightly curved, well-developed in minor males; genae not much developed, rounded; vertex carina lamina-shaped, slightly rounded on sides with the digitiform apex relatively short and rectilinear in major male, short expansion with the superior edge rounded large one third of the width of the head base in minor male. Medium sized, slightly rounded eyes. Antennae yellowish brown. Pronotum that is covered by dense granulate setigerous points, the granules large, and rounded on disc, and larger, thicker, and ovalar on sides; in major males, a large, smooth concave area in anterior part; base with very large points very close. Elytra testaceous and blackish brown, the interstriae covered by regularly spaced setigerous points with small granules. Pygidium black, with large, thick points. Legs blackish brown with short, thick testaceous setae.

Female (Figure 10B,D): length 10–11 mm. Body as in male. Head rounded, clypeus surface rough, with some large points that are near the genal carinae; genae small, rounded, with many large points; frontal carina arched, well-developed, and bulging; vertex carina large, almost reaching the eyes, thick not much elevated, rectilinear; the area between the carinae smooth, concave, with only few small simple points. Pronotum with setigerous granulate points as in male, and two well-developed, very close, symmetrical, anteriorly rounded, and flat expansions on the disc. Elytra and legs as in male. Pygidium shorter than in male, but same colour and puncture.

Epipharynx (Figure 10E): fore margin rectilinear with evenly long, thick setae; zygum constituted by a tuft of long setae; chaetopariae shorter on the proximal half, longer near acropariae; anterior epitorma thin but well-sclerotized, enlarged at base; proplegmatium triangular shaped, low, tapering on sides; apotormae present, rod-shaped, thin; pternotormae short, thick, downturned; laeotorma and dexiotorma symmetrical, short; crepis asymmetrical, well-developed, left-turned; haptomerum covered by many short thin setae, with few being longer on chaetopedia.

Male genitalia (Figure 10F,G,I): phallobase cylindrical, with an anterior rectangular expansion; parameres symmetrical, arched, tapering to the sharp apex; endophallus with a evident raspula with long, thin setae, lamella copulatrix (Figure 10I) present, which is constituted by two distinct parts; accessory lamellae present, well-developed.

Female genitalia (Figure 10H): vagina membranaceous, dome-shaped with a squared, quite elongate infundibular wall; infundibulum question-mark shaped, rectilinear, thick, distal part to ovarioles upward turned; receptaculum seminis not much expanded, lobate, tapering to apex carrying a small, rounded laminal claw, the desclerotized area small and near the apex.

**Distribution.** At present, the species is only known from a circumscribed area between the Democratic Republic of Congo and Zambia (Figure 7C).

- ***Tiaronthophagus lamtoensis* (Cambefort, 1984) n.comb.**

*Onthophagus lamtoensis* Cambefort, 1984: 7.

**Type material. Holotype.** ♂ ‘P139 <Côte d’Ivoire, Lamto, 5-6.7.1980, Piste du Grand Sud, EH, Y.Cambefort leg.’ ‘holotype’ ‘*Tiaronthophagus lamtoensis* (Cambefort, 1984) Roggero, Moretto, Barbero, Palestrini 2019’ (MNHN). **Paratypes.** 550 specimens from Lamto, Abokouamékro, Ouango Fitini, vii.1979-vii.1981 ‘*Tiaronthophagus lamtoensis* (Cambefort, 1984) Roggero, Moretto, Barbero, Palestrini 2019’ (MNHN)

**Other material.** BENIN. North Eastern, E of Kandi, 4 km W of Bensekou (PMOC); Northern, Parc Pendjari vers Tanoungou (JFJC). BURKINA FASO. Bobo Diulasso, Farako Ba (PMOC); Park W, 7 km Koudou (JFJC). GHANA. Kyabobo NP, near Laboum outpost (PMOC). IVORY COAST. Zamou (PMOC) (MNHN); Haut Nzi (MNHL); Korhogo Koko 347 m (PMOC); Odienné (PMOC); Touba Biémasso-Dola (PMOC); Zamou (Comoé) (MNHN).

**Description.** Refer to the Supporting Information Data S5 and S6 and the original description [47].

**Distribution.** The species shows a Western African distribution (Figure 7A).

**Notes**. The species was included in the 24th group [47] and it was considered very close to *T. ebenus*.

- ***Tiaronthophagus liberianus* (Lansberge, 1883) n.comb.**

*Onthophagus liberianus* Lansberge, 1883: 15.

**Type material. Holotype.** ♀ (by monotypy) ‘Liberia Sala & Büttikofer leg.’ ‘Holotype *Onthophagus liberianus* Lansberge, 1883 Roggero, Moretto, Barbero, Palestrini 2019’ ‘*Tiaronthophagus liberianus* (Lansberge, 1883) Roggero, Moretto, Barbero, Palestrini 2019’ (RMNH).

**Other material.** BENIN. Lama (MDCL). GUINEA Nzérékore Ft. Classée de Diecké (PMOC). IVORY COAST. Sassandra, Pauly-Brousse (PMOC) (MDCL); W Sassandra (PMOC) (MDCL); Man, Mt. Tonkoui 1200 m (PMOC) (MDCL); San-Pédro, Monogaga (PMOC); Touba, Biémasso 441 m (PMOC); Comoé, Zamou (PMOC). SIERRA LEONE. 120 m Tiwai island, Moa river (PMOC). TOGO. 10 km N Kpalimé reg. de Missahohe, ft. de Demetui (EBCT).

**Description.** Refer to the Supporting Information Data S5 and S6, original description [48], and Onthophagini Synopsis [2].

**Distribution.** The species has a Western African distribution (Figure 7E).

- ***Tiaronthophagus macroliberianus* (Moretto, 2010) n.comb.**

Onthophagus macroliberianus Moretto, 2010: 465.

**Type material. Holotype.** ♂ ‘Centrafrique Bayanga 16.III–6.IV.96 <handwritten>’ ‘pieges excr. humains <handwritten>’ ‘P. Moretto leg. <handwritten>’ ‘Photo description Moretto 2010 <handwritten>’ ‘Holotype Onthophagus macroliberianus n.sp. P. Moretto det. 2009’ ‘*Tiaronthophagus macroliberianus* (Moretto, 2010) Roggero, Moretto, Barbero, Palestrini 2019’ (PMOC). **Paratypes**. 2♀ ‘Cameroun, Obout, ii.2007 local coll. >D. Moore’ ‘Paratype Onthophagus macroliberianus n.sp. P. Moretto det. 2009’ ‘*Tiaronthophagus macroliberianus* (Moretto, 2010) Roggero, Moretto, Barbero, Palestrini 2019’ (PMOC); 1♀ ‘Cameroun Centre, XI.1996 Mt. Kala M. Desfontaine leg.’ ‘Paratype Onthophagus macroliberianus n.sp. P. Moretto det. 2009’ ‘*Tiaronthophagus macroliberianus* (Moretto, 2010) Roggero, Moretto, Barbero, Palestrini 2019’ (PMOC); 1♀ ‘C Dewalsche Ipamu Kasai’ ‘Paratype Onthophagus macroliberianus n.sp. P. Moretto det. 2009’ ‘*Tiaronthophagus macroliberianus* (Moretto, 2010) Roggero, Moretto, Barbero, Palestrini 2019’ (PMOC); ‘forêt 40 km S/O2-3-83 <handwritten>’ ‘Allotype Onthophagus macroliberianus n.sp. P. Moretto det. 2009’ ‘*Tiaronthophagus macroliberianus* (Moretto, 2010) Roggero, Moretto, Barbero, Palestrini 2019’ (PMOC).

**Other material.** REPUBLIC OF CONGO. Mayoko, 28km NE of Pointe Noire, 663m (PMOC).

**Description.** Refer to the Supporting Information Data S5 and S6 and original description [49].

**Distribution.** The species is located in the Central Africa (Figure 7E), and is rare in collections.

- ***Tiaronthophagus naevius* (d’Orbigny, 1913) n.comb.**

*Onthophagus naevius* d’Orbigny, 1913: 403.

**Type material. Holotype.** ♀ (by monotypy) ‘150–200 miles W.of Kambove. 3500–4500 ft. 23.10.07’ ‘Neave Coll. 1907-230.’ ‘Type’ ‘naevius n.sp. d’Orb. <handwritten by d’Orbigny>‘ ‘Holotype *Onthophagus naevius* d’Orbigny, 1913 Roggero, Moretto, Barbero, Palestrini 2019’ ‘*Tiaronthophagus naevius* (d’Orbigny, 1913) Roggero, Moretto, Barbero, Palestrini 2019’ (BMNH).

**Other material.** TANZANIA. Rukwa Region, Mbizi Mts. Fst. Res., 2252 m (PMOC). ZAMBIA. 50–150 km S Kasempa (PMOC); NorthWestern prov. 80 km W Chingola (PMOC); North Eastern prov. Chimfunsi Wildlife reserve (PMOC).

**Description.** Refer to the Supporting Information Data S5 and S6 and original description in the Onthophagini Synopsis [2].

**Distribution.** The species is found in Central Africa (Figure 7D). It is extremely rare in collections.

- ***Tiaronthophagus pendjarius* (Josso and Prévost, 2006) n.comb.**

*Onthophagus pendjarius* Josso and Prévost, 2006: 4.

**Type material. Holotype.** ♂ ‘N BENIN Parc Pendjari vers hotel Pendjari 1-3-vi-2005 Josso Juhel Monfort leg.’ ‘lumiere’ ‘holotype *Onthophagus pendjarius* n.sp. Josso et Prévost det. 2006’ ‘*Tiaronthophagus pendjarius* (Josso and Prévost, 2006) Roggero, Moretto, Barbero, Palestrini 2019’ (JFJC). **Paratypes.** 1♀ ‘N BENIN Parc Pendjari vers hotel Pendjari 1-3-vi-2005 Josso Juhel Monfort leg.’ ‘lumiere’ ‘paratype *Onthophagus pendjarius* n.sp. Josso et Prévost det. 2006’ ‘*Tiaronthophagus pendjarius* (Josso and Prévost, 2006) Roggero, Moretto, Barbero, Palestrini 2019’ (JFJC); 2♂ ‘Burkina Faso, 8.viii.2005 Loroum, Toulfé, 330 m, 13°54′16″ N, 1°54′19″ W, O, piège excréments humains, P. Moretto leg.’ ‘paratype’ ‘*Onthophagus pendjarius* n.sp. Josso Prévost det. ix.2006’ ‘*Tiaronthophagus pendjarius* (Josso and Prévost, 2006) Roggero, Moretto, Barbero, Palestrini 2019’ (PMOC).

**Other material.** SENEGAL. Fatick Diouroup (PMOC); Gouloumbou (PMOC); Sebikotane (MNHN).

**Description.** Refer to the Supporting Information Data S5 and S6 and the original description [50].

**Distribution.** The species was collected from Western Africa (Figure 7C).

**Notes.** Although the species was formerly included in the 16th group [50], the similarities with the 24th and 28th groups were already highlighted in the original description.

- ***Tiaronthophagus pseudoliberianus* (Moretto, 2010) n.comb.**

*Onthophagus pseudoliberianus* Moretto, 2010: 466.

**Type material. Holotype.** ♂ ‘Congo (Gabon) Ogooué, Lambaréné, 1911–1912–1913 Ellenberger’ ‘*Tiaronthophagus pseudoliberianus* (Moretto, 2010) Roggero, Moretto, Barbero, Palestrini 2019’ (MNHN). **Paratypes.** 1♂ ‘Cameroun Bafia 600m i.1991 Ly? <handwritten>’ ‘Paratype *Onthophagus pseudoliberianus* n.sp. P. Moretto det. 2009’ ‘World Scarab Database WSD00257398’ ‘*Tiaronthophagus pseudoliberianus* (Moretto, 2010) Roggero, Moretto, Barbero, Palestrini 2019’ (PMOC); 1♀ ‘Gabon Woleu-Ntem Oyem ix.2000 >P. Oremans’ ‘Paratype *Onthophagus pseudoliberianus* n.sp. P. Moretto det. 2009’ ‘World Scarab Database WSD00257416’ ‘*Tiaronthophagus pseudoliberianus* (Moretto, 2010) Roggero, Moretto, Barbero, Palestrini 2019’ (PMOC); Congo (Gabon), Ogooué, Lambaréné, 1911–1912–1913 Ellenberger ‘*Tiaronthophagus pseudoliberianus* (Moretto, 2010) Roggero, Moretto, Barbero, Palestrini 2019’ (MNHN); 7♂ 2♀ ‘Centrafrique Bayanga 16.iii 6.iv.96 <handwritten>’ ‘pieges iules <handwritten>’ ‘P. Moretto leg. <handwritten>’ ‘Paratype *Onthophagus pseudoliberianus* n.sp. P. Moretto det. 2009’ ‘*Tiaronthophagus pseudoliberianus* (Moretto, 2010) Roggero, Moretto, Barbero, Palestrini 2019’ (PMOC); 5♂ 6♀ ‘Centrafrique Bayanga, 16.iii 6.iv.96 ‘pieges iules P. Moretto leg.’ ‘Paratype *Onthophagus pseudoliberianus* n.sp. P. Moretto det. 2009’ ‘*Tiaronthophagus pseudoliberianus* (Moretto, 2010) Roggero, Moretto, Barbero, Palestrini 2019’ (PMOC); 4♂ ‘Cameroun, N W Province Région de Bamenda, 1–3.vii.2008 Local. coll Voma eg.’ ‘Paratype *Onthophagus pseudoliberianus* n.sp. P. Moretto det. 2009’ ‘*Tiaronthophagus pseudoliberianus* (Moretto, 2010) Roggero, Moretto, Barbero, Palestrini 2019’ (PMOC); 3♂ 6♀ ‘Cameroun, N W Province Région de Bamenda, Bali, 5–16.vii.2006 Local. coll Jomia leg.’ ‘Paratype *Onthophagus pseudoliberianus* n.sp. P. Moretto det. 2009’ ‘*Tiaronthophagus pseudoliberianus* (Moretto, 2010) Roggero, Moretto, Barbero, Palestrini 2019’ (PMOC); 4♂ 2♀ ‘Cameroun N W Province Région de Bamenda, Bali-Nyonga 28.viii-1.ix.2006. Local. coll Jomia leg.’ ‘Paratype *Onthophagus pseudoliberianus* n.sp. P. Moretto det. 2009’ ‘*Tiaronthophagus pseudoliberianus* (Moretto, 2010) Roggero, Moretto, Barbero, Palestrini 2019’ (PMOC); 1♀ ‘O. liberianus… J.L. Nicolas det.1994 <handwritten>’ ‘Cameroun N Sanaga Ndom ii.93 J.C. Thibaud leg. <handwritten>’ ‘Paratype *Onthophagus pseudoliberianus* n.sp. P. Moretto det. 2009’ ‘*Tiaronthophagus pseudoliberianus* (Moretto, 2010) Roggero, Moretto, Barbero, Palestrini 2019’ (PMOC); 2♂ ‘Africa Cameroon SWP Bali 28-vii-05 #19 V.L. Voma Human Feces Cornell Voma Korup NP Exped’ ‘Paratype *Onthophagus pseudoliberianus* n.sp. P. Moretto det. 2009’ ‘Paratype *Onthophagus pseudoliberianus* n.sp. P. Moretto det. 2009’ ‘*Tiaronthophagus pseudoliberianus* (Moretto, 2010) Roggero, Moretto, Barbero, Palestrini 2019’ (PMOC); Gabon Woleu Ntem Oyem ix.2000 P. Oremans (PMOC); Cameroun, mount Cameroun, Bafia 600m i.1991 ‘*Tiaronthophagus pseudoliberianus* (Moretto, 2010) Roggero, Moretto, Barbero, Palestrini 2019’ (PMOC); Cameroun Bafia 600m i.91 ‘*Tiaronthophagus pseudoliberianus* (Moretto, 2010) Roggero, Moretto, Barbero, Palestrini 2019’ (PMOC).

**Other material.** CAMEROON. W region, Lebialem division, Wabane sub.div., Folepi village (PMOC); NW province, Region de Bamenda Bali (PMOC). CENTRAL AFRICAN REPUBLIC. N.P. Ndoki Lac (PMOC). EQUATORIAL GUINEA. Island Fernando Poo (now, Bioko), Musola. 500–800 m.s.m. (MSNG). GABON. E Ogoouè, Ivindo province, Makokou SW Ivindo N.P., Ipassa forest research station (PMOC).

**Description.** Refer to the Supporting Information Data S5 and S6 and the original description [49].

**Distribution.** The species is known from the Central Western Africa (Figure 7E).

- ***Tiaronthophagus rolandoi* n.sp.**

Zoobank Registration: http://zoobank.org/urn:lsid:zoobank.org:act:3A425D2F-DDAC-4989-B8B4-BD201D16F0AD.

**Figure 11:**
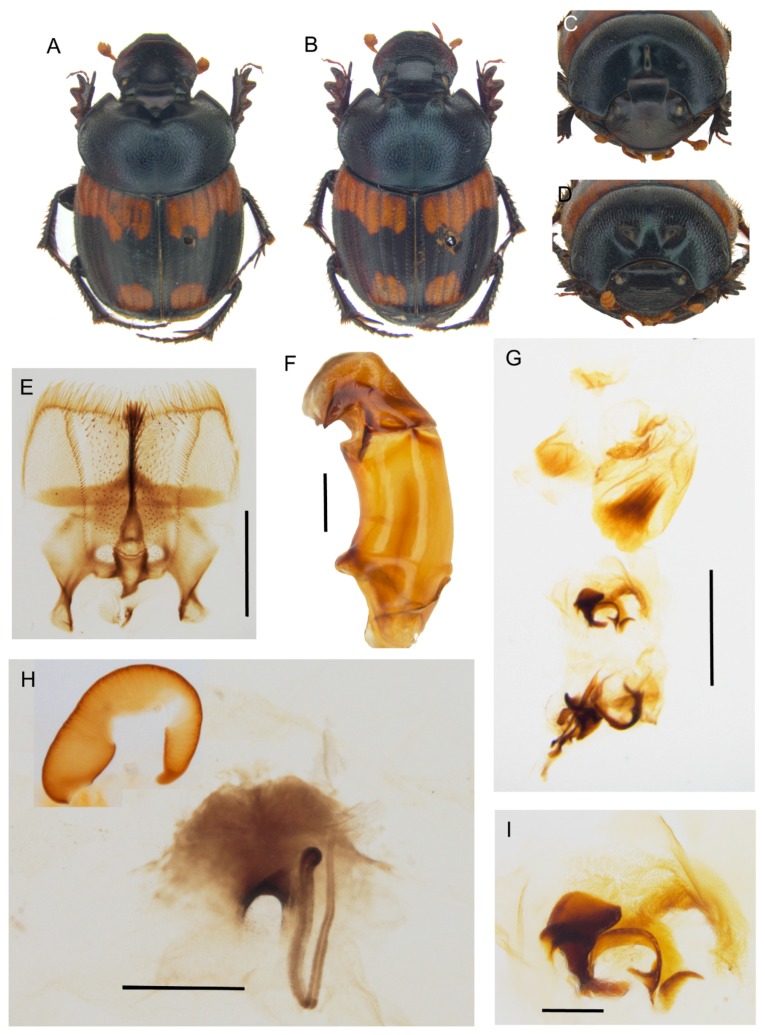
Features of *Tiaronthophagus rolandoi* n.sp. (**A**): Male facies (above). (**B**): Female facies (above). (**C**): Male facies (frontal view). (**D**): Female facies (frontal view). (**E**): Epipharynx, scalebar = 0.5 mm. (**F**): Male genitalia, phallobase and parameres, scalebar = 0.5 mm. (**G**): Male genitalia, endophallus, scalebar = 1.0 mm. (**H**): Female genitalia, vagina and receptaculum seminis, scalebar = 0.3 mm. (**I**): Male genitalia, lamella copulatrix, scalebar = 0.2 mm.

Figure 7 and Figure 11, Supporting Information Data S5 and S6.

**Type material. Holotype.** ♂ ‘Tanzania, Manyara, 1660 m, Ngorongoro N.W. 3°19′39 S, 35°36′19 E, 25.II–14.III.2008 T. & M. Ströhle leg.’ ‘*Tiaronthophagus rolandoi* n.sp. holotype’ (BMNH). **Paratypes** 1♂ 1♂ ‘Tanzanie, Mbeya Prov. Usangu Flats 1139m savane à épineux, 21.ii, 2004 R. Minetti leg.’ ‘*Tiaronthophagus rolandoi* n.sp. paratype’ (PMOC); 1♀ ‘Tanzanie Mwanza reg. Geita iv-2011. Minetti leg.’ ‘*Tiaronthophagus rolandoi* n.sp. paratype’ (JFJC); 2♀ ‘Tanzania, Manyara, 1660m, Ngorongoro N.W. 3°19′39 S, 35°36′19 E, 25.II–14.III.2008 T. & M. Ströhle leg. ‘*Tiaronthophagus rolandoi* n.sp. paratype’ (PMOC); 1♀ ‘Tanzanie 21.III-10.IV.2007. Manyara, Ngorongoro, NW. Karatu 1660m 3°19′39 S, 35°36′19″ E, Ströhle leg. ‘*Tiaronthophagus rolandoi* n.sp. paratype’ (PMOC); 1♂ 1♀ ‘N Malawi Chiwenga 1600 m 10–12.xii.2006 JossoJuhelMonfort leg.’ ‘poisson pourri’ ‘*Tiaronthophagus rolandoi* n.sp. paratype’ (JFJC); 1♂ ‘N Malawi Chiwenga 1600 m 10–12.xii.2006 JossoJuhelMonfort leg.’ ‘cadavre iule’ ‘*Tiaronthophagus rolandoi* n.sp. paratype’ (JFJC); 1♀ ‘Uganda Busia I.90 B. Wandora leg. <handwritten>’ ‘*Tiaronthophagus rolandoi* n.sp. paratype’ (PMOC); 1♂ ‘Uganda 5.v.2009 Queen Elizabeth NP P. Malec lgt.’ ‘*Tiaronthophagus rolandoi* n.sp. paratype’ (JFJC); 1♂ 1♀ ‘Uganda Queen Elizabeth NP 0°15’ S/30°00’ E ii.1996–iii.1997 leg. A. Hoffmann’ ‘*Tiaronthophagus rolandoi* n.sp. paratype’ (JFJC); 1♂ ‘acquis en iii-2003 Kenya’ ‘*Onthophagus rufobasalis* Fairmaire J.L. Nicolas det. 03’ ‘*Tiaronthophagus rolandoi* n.sp. paratype’ (MDCL); 1♂ 1♀ ‘4.XII.2012 PL. 1075m 6 km N Chunga 14°59′39″ S, 26°01′11″ E, Zambie Central Prov. S. Rojkoff réc.’ ‘*Tiaronthophagus rolandoi* n.sp. paratype’ (PMOC); 1♂ 8-xii-205 40 km E Kapiri Mposhi Central Province Zambia S. Rojkoff & K. Werner rec.’ ‘Onthophagus cf rufobasalis Fairmaire M. Dierkens det. 2014’ ‘*Tiaronthophagus rolandoi* n.sp. paratype’ (MDCL); 1♂ ‘Zambie Kafue NP Chunga 15°02,362’ S, 25°59,437’ E, 11–12-xii-2009 Josso Juhel Minetti leg.’ ‘exc. humains’ ‘*Tiaronthophagus rolandoi* n.sp. paratype’ (JFJC); 3♂ ‘Zambie Kafue NP Mayukuyuku Camp 13-xii-2009 Josso Juhel Minetti leg.’ ‘lumiere’ ‘*Tiaronthophagus rolandoi* n.sp. paratype’ (JFJC); 1♂ 2♀ ‘Zambie NW Prov. 30km N Lubungu NE Kafue NP. 10-11xii2012 14°11’ S, 26°29’ E, 3775Ft. leg. Josso Juhel Minetti’ ‘lumiere’ ‘*Tiaronthophagus rolandoi* n.sp. paratype’ (JFJC).

**Other material.** ETHIOPIA. Oromia 11.5 km S of Kibre Mengist 2200 m (MECI).

**Etymology.** The species was named after our colleague, the Italian ornithologist Antonio Rolando.

**Description.** Male (Figure 11A,C): length 10–12 mm. Body greenish/bluish black, being covered by a short testaceous pubescence, with two large testaceous patches at the base and apex of elytra, basal one extending from elytral sutura to side margin, the apical one rounded and then placed on interstriae 1–4. Head surface smooth and covered by small points mixed to few larger ones; frontal carina largely triangular, sometimes being almost inapparent in major males, curved, more evident in minor males; genae not developed, slightly rounded; vertex carina lamina-shaped, rectangular with the digitiform apex relatively long and arched in major male, and shaped as a short, narrow protuberance with the superior edge that is rounded in minor male. Medium sized, slightly rounded eyes. Antennae yellowish brown. Pronotum covered by dense granulate setigerous points, large, ovalar granules becoming larger and thicker (almost embricate) on the sides; in major males a large, smooth, concave area in anterior part; pronotum base with a tight row of very large points. Elytra testaceous and black with greenish/bluish hue, the first two interstriae without granules, almost smooth, the others covered by sparse, regularly spaced setigerous points with small granules. Pygidium black, covered by deep, dense puncture, with large, simple points mixed to smaller ones. Legs blackish brown with short, thick testaceous setae.

Female (Figure 11B,D): length 11–12 mm. Body as in male. Head surface rough on clypeus with some large points near the genal suturae, smooth with small sparse points on the remaining parts; genae not developed, slightly rounded, covered by many large points; frontal carina rounded, elevated, thick; vertex carina thick, rectilinear, large, reaching the eyes, area between the carinae smooth with relatively dense, small points being simple. Pronotum granulation as in male, two well-developed, conical protuberances slightly diverging, flat, and rounded from dorsal view. Elytra and legs as in male. Pygidium shorter than in male, but the same colour and puncture.

Epipharynx (Figure 11E) anterior margin slightly concave, without central notch, acropariae that is constituted by very long, thin setae; zygum constituted by a tuft of long thick setae; proplegmatium triangular shaped, lowered; anterior epitorma thin, rod-shaped, but enlarged in the proximal third; plegmatic area ovalar lowered; chaetopariae rectilinear, proximal half with very short setae, longer on distal half; apotormae present, thin, rod-shaped, short; pternotormae short and thick; crepis asymmetrical, left-turned, sharp at apex; laeotorma and dexiotorma symmetrical, short, and thick; haptomerum with very short and thick setae.

Male genitalia (Figure 11F,G,I): phallobase cylindrical, short, with an anterior rectangular expansion; parameres symmetrical, arched, tapering to the sharp apex; endophallus with a evident raspula constituted by very long, thin setae, lamella copulatrix (Figure 11I) present, well-sclerotized, constituted by two distinct parts; accessory lamellae present, well-developed.

Female genitalia (Figure 11H): vagina membranaceous, dome-shaped with a symmetrical, squared, short infundibular wall, with a rounded notch at base, and a well-sclerotized expansion; infundibulum question-mark shaped, rectilinear, thick, the distal part to ovarioles upward turned; receptaculum seminis not much expanded, lobate, tapering to the apex carrying a rounded laminal claw, the large desclerotized area that is placed in the central third.

**Distribution.** The species was collected from Eastern Africa (Figure 7F).

- ***Tiaronthophagus rougonorum* (Cambefort, 1984) n.comb.**

*Onthophagus rougonorum* Cambefort, 1984: 8.

**Type material.Holotype.** ♂ ‘Ouango Fitini, iii.1981’ ‘*Tiaronthophagus rougonorum* (Cambefort, 1984) Roggero, Moretto, Barbero, Palestrini 2019’ (MNHN). **Paratypes.** 55 specimens ‘Ouango Fitini, iii.1981’ ‘*Tiaronthophagus rougonorum* (Cambefort, 1984) Roggero, Moretto, Barbero, Palestrini 2019’ (MNHN); 58 specimens ‘Ouango Fitini, v.1981’ ‘*Tiaronthophagus rougonorum* (Cambefort, 1984) Roggero, Moretto, Barbero, Palestrini 2019’ (MNHN); six specimens ‘Ouango Fitini, vii.1981’ ‘*Tiaronthophagus rougonorum* (Cambefort, 1984) Roggero, Moretto, Barbero, Palestrini 2019’ (MNHN); four specimens ‘Niger Tapoa, vii–viii.1976 D. Rougon’ ‘*Tiaronthophagus rougonorum* (Cambefort, 1984) Roggero, Moretto, Barbero, Palestrini 2019’ (MNHN).

**Other material.** BENIN. Tanguieta (Atakore) (PMOC). BURKINA FASO. Sanguié, Boromo, Forêt de Sorobouli, 270 m (PMOC); Sanguié, Forêt de Sorobouli, 270 m (PMOC). IVORY COAST. Korhogo, Koko (PMOC); Korhogo (PMOC); Man, Mt. Tonkoui (PMOC); PN du Mt. Sangbé (PMOC). SENEGAL. Kolda, Mahon Forêt de Bakor (PMOC); Niokolo Koba NP, Niokolo (PMOC); Niokolo Koba NP, poste de Niokolo, 65 m (PMOC); Niokolo Koba NP, Siminti (PMOC); PN Niokolo Koba, lisière Ft. galerie, Mt. Assirik, 144 m (PMOC); PN Niokolo Koba, savane arborée, Mt. Assirik, 144 m (PMOC). TOGO. Fazao, PN du Fazao (PMOC).

**Description.** Refer to the Supporting Information Data S5 and S6 and original description [47].

**Distribution.** The species was collected from Western Africa (Figure 7B).

**Notes.** The species was included in 28th group [47], and related to *T. flexicornis*.

- ***Tiaronthophagus rufobasalis* (Fairmaire, 1887) n.comb.**

*Onthophagus rufobasalis* Fairmaire, 1887: 113.

= *Onthophagus heynei* Lansberge, 1887: 108. D’Orbigny, 1902: 176.

**Type material. Lectotype.** ♂ (here designated) ‘Guelidi <handwritten>‘ ‘*Onthophagus rufobasalis* Fm. guelidi <handwritten>‘ rufobasalis Fairm. <handwritten>‘ ‘Museum Paris coll. L. Fairmaire 1906’ ‘Syntype’ ‘Syntype *Onthophagus rufobasalis* Fairmaire, 1887’ ‘MNHN EC8641’ ‘Lectotype *Onthophagus rufobasalis* Fairmaire, 1887 Roggero, Moretto, Barbero, Palestrini 2019’ ‘*Tiaronthophagus rufobasalis* (Fairmaire, 1887) Roggero, Moretto, Barbero, Palestrini 2019’ (MNHN). **Paralectotype.** ♀ Guelidi Revoil <handwritten>‘ ‘ex Typis’ ‘Museum Paris coll. L. Fairmaire 1906’ ‘Syntype’ ‘Syntype *Onthophagus rufobasalis* Fairmaire, 1887’ ‘MNHN EC8642’ ‘Paralectotype *Onthophagus rufobasalis* Fairmaire, 1887 Roggero, Moretto, Barbero, Palestrini 2019’ ‘*Tiaronthophagus rufobasalis* (Fairmaire, 1887) Roggero, Moretto, Barbero, Palestrini 2019’ (MNHN).

**Synonymy type material. *O. heynei*** ♂ (lectotype) ‘E. Heyne Somalis Africa <handwritten by Lansberge>’ ‘Onthophagus Heynei Lansbge <handwritten by d’Orbigny>’ ‘RMNH.INS 1104278’ ‘*Tiaronthophagus rufobasalis* (Fairmaire, 1887) Roggero, Moretto, Barbero, Palestrini 2019’ (RMNH). 1♀ (paralectotype) ‘E. Heyne Somalis Africa <handwritten by Lansberge>’ ‘Onthophagus Heynei Lansberge ♀’ ‘RMNH.INS 1104277 <handwritten by Lansberge>’ ‘*Tiaronthophagus rufobasalis* (Fairmaire, 1887) Roggero, Moretto, Barbero, Palestrini 2019’ (RMNH); 1♂ (paralectotype) ‘Ex-Musaeo Van Lansberge’ ‘Heynei. Lansbe. Type Somali’ ‘Muséum Paris ex. Coll. R. Oberthür’ ‘Syntype’ ‘Syntype *Onthophagus heynei* Lansberge, 1887’ ‘MNHN EC8643’ ‘*Tiaronthophagus rufobasalis* (Fairmaire, 1887) Roggero, Moretto, Barbero, Palestrini 2019’ (MNHN).

**Other material.** ETHIOPIA. 2 km SE Key Afer, Gamu Gofa (PMOC); Southern, Turmi 920 m Mango-Lodge (PMOC); Hamer or., Turmi near 950 m (MDCL). KENYA. Mombasa Diani (EBCT); Meru District, Gatunga (PMOC); Meru district, Materi (Mitunguu) mt. 800 (PMOC); Mombasa Diani beach (PMOC); 30 km S Mombasa (JFJC); 5 km N Malindi (EBCT); km 65 S Mombasa (EBCT); Mombasa (JFJC); Afr. Or. Shimo la Tewa (NHMW); Kibwezi (NHMW); Hola (MDL) (PMOC). SOMALIA. Benadir Afgoi (Mogadiscio) (NHMW); ‘Somalia italiana’ Salambà (MSNG); Benadir Mogadiscio (MSNG); ‘Somalia It.’ O. Giuba Balet Amin (MSNG); ‘Somalia italiana mer.’ Villaggio Duca degli Abruzzi (now, Giohar) (MSNG); Balad (PMOC) (EBCT); Mogadiscio (MCST); umg. Mogadiscio (PMOC). TANZANIA. Trockenvald b. Mtotohovu, ‘DOA’ (in Tanga district) (ZMHB); 35 km E Singida (JFJC); ‘Afr.-Tanzanien’ Ngorongoro res. Serengeti (JFJC); Dar es Salaam campus université (JFJC); Dar es Salaam (PMOC); Iringa prov. Chimala 1000 m (PMOC); Morogoro region, Mikumi (PMOC); Iringa Province, Mkimbizi Mts. (PMOC); Morogoro region, Mazimbu area (PMOC); Morogoro region, Mikesse Hills, 420 m (PMOC); Uluguru Mts. (PMOC); Morogoro region, 360 m, Mbarika Mts. (Ikongwa) between Kichangari and Idunda (PMOC); Morogoro region, Udzungwa N.P. forêt, 390 m (PMOC); Taga region, savane de Kisangiro, 750 m (PMOC); Rufiji river, 15 km W Ikwiriri (MDCL): Mt. Kilimanjaro (PMOC).

**Description.** Refer to the Supporting Information Data S5 and S6, original description [48,51] and Onthophagini Synopsis [2].

**Distribution.** The species is known from Eastern Africa (Figure 7D).

**Notes.** The synonymy of *T. rufobasalis* and *O. heynei* was confirmed by the survey of the typical material of these species.

- ***Tiaronthophagus rufopygus* (Frey, 1957) n.comb.**

*Onthophagus rufopygus* Frey, 1957: 688.

**Type material. Holotype.** ♂ ‘Kumba 20.xi.1955’ ‘♂’ ‘H. Typus <handwritten> Onthophagus <print> rufo—♂ <handwritten> pygus m <handwritten> det. G. Frey, 1957 <print>’ ‘Exped. Mus. G. Frey Nigeria-Kamerun Bechyne 1955-56’ ‘holotype’ ‘*Tiaronthophagus rufopygus* (Frey, 1957) Roggero, Moretto, Barbero, Palestrini 2019’ (NHMB). **Paratypes**. 2♂ ‘Kumba 20.xi.1955’ ‘♂’ ‘P. Typus *Onthophagus rufopygus* ♂ <handwritten> det. G. Frey, 1957 <print>’ ‘Exped. Mus. G. Frey Nigeria-Kamerun Bechyne 1955-56’ ‘paratype’ ‘*Tiaronthophagus rufopygus* (Frey, 1957) Roggero, Moretto, Barbero, Palestrini 2019’ (NHMB). **Allotypes**. 1♂ and 1♀ ‘Kumba 20.xi.1955’ ‘A. Typus *Onthophagus rufopygus* <handwritten> det. G. Frey, 1957 <print>’ ‘Exped. Mus. G. Frey Nigeria-Kamerun Bechyne 1955–56’ ‘allotype’ ‘*Tiaronthophagus rufopygus* (Frey, 1957) Roggero, Moretto, Barbero, Palestrini 2019’ (NHMB).

**Other material.** IVORY COAST. Abidjan PN du Banco (PMOC); Man Mt. Tonkoui 1200 m (PMOC); Danané (PMOC); Man Mt. Tonkoui (PMOC). GUINEA. Nzerekore Forêt, Classée de Diecké (PMOC). SIERRA LEONE. 120 m Tiwai Island, Moa river (BMNH).

**Description.** Refer to the Supporting Information Data S5 and S6 and original description [52].

**Distribution.** Except the type material, all the recently collected material come from the Western Africa (Figure 7E). The type material was collected by Bechyné during his 1956 trip in Nigeria and British Cameroon. The collection area of *O. rufopygus* was classified as ‘rainforest’.

**Notes.** The species was included in 24th group [52] although the lamina of vertex of *T. rufopygus* is very different from that of other species of the same group, as *T. schaufussi*.

- ***Tiaronthophagus rufostillans* (d’Orbigny, 1907) n.comb.**

*Onthophagus rufostillans* d’Orbigny, 1907: 173.

**Type material. Holotype.** ♀ ‘Togo Conradt’ ‘holotypus’ ‘rufostillans n.sp. d’Orb. <handwritten by d’Orbigny>’ ‘*Tiaronthophagus rufostillans* (d’Orbigny, 1907) Roggero, Moretto, Barbero, Palestrini 2019’ (SDEI).

**Other material.** CAMEROON. Extreme north, province Logone and Chari, division Wasa (PMOC). GHANA. Kyabobo National Park near Laboum Outpost (PMOC). IVORY COAST. Korhogo, Koko 347 m (PMOC); Touba (PMOC); Comoé, Zamou (PMOC); Odienne, Sud Massif du Denguélé (PMOC); Touba, Biémasso, 441 m (PMOC); Touba, Biémasso-Dolla (PMOC); Lamto (PMOC); Man, Mt. Tonkui (PMOC).

**Description.** Refer to the Supporting Information Data S5 and S6, original description [53] and Onthophagini Synopsis [2].

**Distribution.** The species has a Western Africa distribution (Figure 7F).

- ***Tiaronthophagus saadaniensis* n.sp.**

Zoobank Registration: http://zoobank.org/urn:lsid:zoobank.org:act:E5024024-C278-438F-969C-CC0FF7BE5B0F.

**Figure 12:**
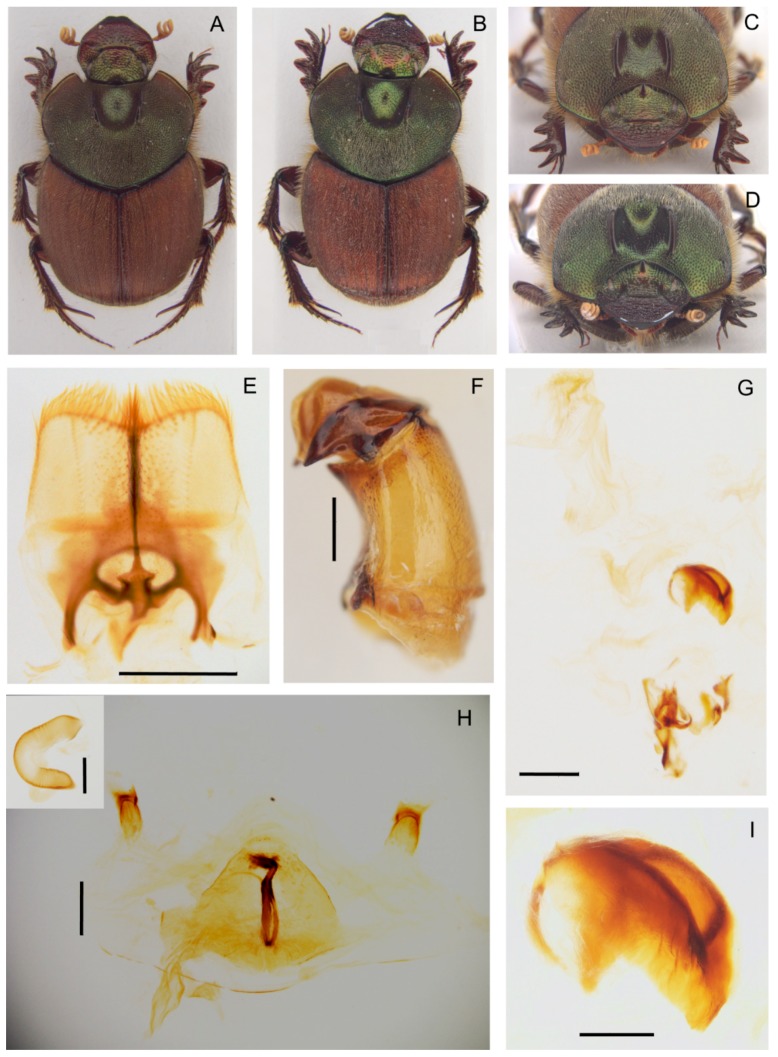
Features of *Tiaronthophagus saadaniensis* n.sp. (**A**): Male facies (above). (**B**): Female facies (above). (**C**): Male facies (frontal view). (**D**): Female facies (frontal view). (**E**): Epipharynx scalebar = 1.0 mm. (**F**): Male genitalia, phallobase and parameres, scalebar = 0.5 mm. (**G**): Male genitalia, endophallus, scalebar = 0.5 mm. (**H**): Female genitalia, vagina, scalebar = 0.3 mm, and receptaculum seminis, scalebar = 0.2 mm. (**I**): Male genitalia, lamella copulatrix, scalebar = 0.2 mm.

Figure 7 and Figure 12, Supporting Information Data S5 and S6.

**Type material. Holotype**. ♂ ‘Onthophagus Tanz. sp. 46 S. Pokorný det 2014’ ‘Tanzania 200m Zaraninge Coastal Forest Saadani N.P., S. Pools x–xi.94 Pitfall Trap UDSM coll.’ ‘BMNH(E) 2013-71 1310485’ ‘BMNH <handwritten>’ ‘*Tiaronthophagus saadaniensis* n.sp. holotype’ (BMNH). **Paratypes.** 11♂ and 2♀ ‘Tanzania 200m Zaraninge Coastal Forest Saadani N.P., S. Pools x–xi.94 Pitfall Trap UDSM coll.’ ‘BMNH(E) 2013-71 1310492’ ‘BMNH <handwritten>’ ‘Onthophagus Tanz. sp. 46 S. Pokorný det 2014’ ‘*Tiaronthophagus saadaniensis* n.sp. paratype’ (BMNH).

**Other material.** No other material is known at present.

**Etymology.** The species was named after the collection locality in Tanzania.

**Description.** Male (Figure 12A,C): length 11–13.5 mm. Body wholly covered by a long, dense and thin pubescence light yellow; head cupreous, pronotum green, frequently with cupreous reflects; elytra brown, mat. Head surface rough, with some large points on clypeus; genae not expanded, slightly rounded, with large points; frontal carina slightly rounded, evident; vertex carina conical-shaped, sharp, very small. Antennae lamellae light yellow, the scape brown. Pronotum entirely covered by very thick, large granulate points, granules very large, ovalar, except for the fore central smooth area with two lateral elevated triangular expansions. Elytral interstriae evenly covered by very thick granulate points, the granules large, ovalar. Pygidium testaceous with a light greenish lustre, with rounded, dense points and thick, long yellow pubescence on the whole surface. Legs brown with long thick setae.

Female (Figure 12B,D): length 11.5–14 mm. Similar to male. Pygidium shorter and darker.

Epipharynx (Figure 12E) anterior margin largely triangular notched, acropariae evenly long; zygum reduced to few long setae; anterior epitorma very thin, rod-shaped; proplegmatium triangular, very lowered, sides rectangular, apotormae barely visible, rod-shaped; pternotormae narrow, downturned; plegmatic area ovalar, large, enlarged, crepis narrow, asymmetrical, left turned; laeotorma and dexiotorma symmetrical, short, relatively thin; haptomerum with very short, thick setae.

Male genitalia (Figure 12F,G,I): phallobase cylindrical, short, with an anterior rectangular expansion; parameres symmetrical, almost rectilinear, tapering to the sharp apex, not vertically expanded; endophallus with a evident raspula constituted by very long, thin setae, lamella copulatrix (Figure 12I) present, well-sclerotized; accessory lamellae present, well-developed.

Female genitalia (Figure 12H): vagina membranaceous, dome-shaped with a symmetrical, large, triangular infundibular wall, and two sacciform, sclerotized protuberances on sides; infundibulum question-mark shaped, rectilinear, thick, distal part to ovarioles upward turned; receptaculum seminis not much expanded, tubular, apex rounded with a small laminal claw, desclerotized area that is placed in the central third.

**Distribution.** The species at present is known only from the type locality in Tanzania (Figure 7E).

- ***Tiaronthophagus schaufussi* (Harold, 1867) n.comb.**

*Onthophagus schaufussi* Harold, 1867: 43.

= *Onthophagus nutans* (nec Fabricius) var. *maxima* Roth, 1851 (MNHN, see notes below). Harold, 1867: 44.

**Type material. Lectotype.** ♂ (here designated) ‘Tigré. <handwritten>‘ ‘Schaufussi T. Harold <handwritten>‘ ‘Ex-Musaeo Harold’ ‘Muséum Paris ex Coll. R. Oberthür’ ‘Syntype’ ‘Syntype *Onthophagus schaufussi* Harold, 1867’ ‘MNHN EC8636’ (MNHN) ‘Lectotype *Onthophagus schaufussi* Harold, 1851 Roggero, Moretto, Barbero, Palestrini 2019’ ‘Paralectotype *Onthophagus nutans* var. *maxima* Roth, 1851 Roggero, Moretto, Barbero, Palestrini 2019’ ‘*Tiaronthophagus schaufussi* (Harold, 1851) Roggero, Moretto, Barbero, Palestrini 2019’. **Paralectotypes.** ♂ ‘Tigré. O. schaufussi Harold. ‘<handwritten>‘ ‘Ex-Musaeo Harold’ ‘Muséum Paris ex Coll. R. Oberthür’ ‘Syntype’ ‘Syntype *Onthophagus schaufussi* Harold, 1867’ ‘MNHN EC8637’ ‘Paralectotype *Onthophagus nutans* var. *maxima* Roth, 1851 Roggero, Moretto, Barbero, Palestrini 2019’ ‘Paralectotype *Onthophagus schaufussi* Harold, 1851 Roggero, Moretto, Barbero, Palestrini 2019’ ‘*Tiaronthophagus schaufussi* (Harold, 1851) Roggero, Moretto, Barbero, Palestrini 2019’ (MNHN); ♀ ‘Tigré <handwritten>‘ ‘Ex-Musaeo Harold’ ‘Muséum Paris ex Coll. R. Oberthür’ ‘Syntype’ ‘Syntype *Onthophagus schaufussi* Harold, 1867’ ‘MNHN EC8638’ ‘Paralectotype *Onthophagus nutans* var. *maxima* Roth, 1851 Roggero, Moretto, Barbero, Palestrini 2019’ ‘Paralectotype *Onthophagus schaufussi* Harold, 1851 Roggero, Moretto, Barbero, Palestrini 2019’ ‘*Tiaronthophagus schaufussi* (Harold, 1851) Roggero, Moretto, Barbero, Palestrini 2019’ (MNHN); ♂ ‘Tigré. <handwritten>‘ ‘Ex-Musaeo Harold’ ‘Muséum Paris ex Coll. R. Oberthür’ ‘Syntype’ ‘Syntype *Onthophagus schaufussi* Harold, 1867’ ‘MNHN EC8639’ ‘Paralectotype *Onthophagus nutans* var. *maxima* Roth, 1851 Roggero, Moretto, Barbero, Palestrini 2019’ ‘Paralectotype *Onthophagus schaufussi* Harold, 1851 Roggero, Moretto, Barbero, Palestrini 2019’ ‘*Tiaronthophagus schaufussi* (Harold, 1851) Roggero, Moretto, Barbero, Palestrini 2019’ (MNHN); ♂ ‘Tigré. Schimper. <handwritten>‘ ‘Ex-Musaeo Harold’ ‘Muséum Paris ex Coll. R. Oberthür’ ‘Syntype’ ‘Syntype *Onthophagus schaufussi* Harold, 1867’ ‘MNHN EC8640’ ‘Paralectotype *Onthophagus nutans* var. *maxima* Roth, 1851 Roggero, Moretto, Barbero, Palestrini 2019 ‘Paralectotype *Onthophagus schaufussi* Harold, 1851 Roggero, Moretto, Barbero, Palestrini 2019’ ‘*Tiaronthophagus schaufussi* (Harold, 1851) Roggero, Moretto, Barbero, Palestrini 2019’ (MNHN).

**Synonymy type material. *O. nutans* var. *maxima*. Lectotype** ♂ (here designated), ‘Abyssinia O schaufussi Typi: Har: <handwritten>’ ‘round blue chip’ ‘something <red>’ ‘Lectotype *Onthophagus nutans* var. *maxima* Roth, 1851 Roggero, Moretto, Barbero, Palestrini 2019‘ ‘Paralectotype *Onthophagus schaufussi* Harold, 1851 Roggero, Moretto, Barbero, Palestrini 2019’ ‘*Tiaronthophagus schaufussi* (Harold, 1851) Roggero, Moretto, Barbero, Palestrini 2019’ (ZSM). **Paralectotypes. *O. nutans* var. *maxima*** nine ex. ‘round blue chip’ ‘Paralectotype *Onthophagus nutans* var. *maxima* Roth, 1851 Roggero, Moretto, Barbero, Palestrini2019’ ‘Paralectotype *Onthophagus schaufussi* Harold, 1851 Roggero, Moretto, Barbero, Palestrini 2019’ ‘*Tiaronthophagus schaufussi* (Harold, 1851) Roggero, Moretto, Barbero, Palestrini 2019’ (ZSM).

**Other material.** ERITREA. Serayè Dubarwa (EBCT) (PMOC); env. Asmara (PMOC). ETHIOPIA. Gemu-Gofa prov. near Arba Minch (PMOC) (MDCL); Oromia reg Arsi Negele Woreda 1600 m Lake Langano near Bishangari (PMOC); Asosa, Benishangui (MDCL); Turmi, 920 m (PMOC).

**Description.** Refer to the Supporting Information Data S5 and S6, original description [54] and Onthophagini Synopsis [2].

**Distribution.** The species is distributed in Eastern Africa (Figure 7F).

**Notes.** Following the ICZN code (45.6.4), the subspecific rank can be assigned to *O. nutans* var. *maxima* Roth, and the taxon can be considered to be valid. Harold (1867: 45) stated that the type material of *O. schaufussi* come from the Roth collection. The author described the species on some specimens which were formerly described [55] as *O. nutans* Fabricius var. *maxima* from material collected by Schimper in Abyssinia. Ten specimens of the same material collected by Schimper and coming from Roth collection are preserved in ZSM as type material of *O. schaufussi* Harold. Together, the five syntypes of O. schaufussi Harold held in Paris and the 10 syntypes held in München are the type series of *O. nutans* var. *maxima* Roth and of *O. schaufussi* Harold, which are synonyms. At present, *O. nutans* Fabricius, 1787 is regarded as a synonym of *Palaeonthophagus verticicornis* (Laichtarting, 1781), thus the proposed variety is surely due to misidentification. Although the var. *maxima* was described prior *O. schaufussi*, it was only mentioned as a synonym of the latter name, which must be maintained following the stability criterion that is recommended by the ICZN.

- ***Tiaronthophagus viridiaereus* (d’Orbigny, 1908) n.comb.**

*Onthophagus viridiaereus* d’Orbigny, 1908: 163.

**Type material. Holotype.** ♂ (by monotypy) ‘Guinée Port. Chime 1906 Rio Geba G. Favarel d’Orbigny 1908 coll. Oberthur’ ‘Holotype *Onthophagus viridiaereus* d’Orbigny, 1908 Roggero, Moretto, Barbero, Palestrini 2019’ ‘*Tiaronthophagus viridiaereus* (d’Orbigny, 1908) Roggero, Moretto, Barbero, Palestrini 2019’ (MNHN).

**Other material.** BENIN. Ndali, 3 km W of Sontou (PMOC); Penessoulou forêt de Penelan (PMOC). BURKINA FASO. Bama, site de Samandeni, 330 m (PMOC); Bobo Diulasso (PMOC); Bobo Diulasso, Farako Ba Station (PMOC); Loroum Toulfé 330 m (PMOC); Nahouri Tiakané 340 m (PMOC); NE Oubritenga, Zithenga Tanghen (=Zitenga-Tanghin) (PMOC). CAMEROON. Extreme north (PMOC). DEMOCRATIC REPUBLIC OF CONGO. ‘Congo Belge’ Central Province of Maniema, Kindu (MNHN). GUINEA BISSAU. Binar, Oio; Coli (Quebo), Tombali (CAS). MALI. Segou (MHNL). SENEGAL. Fatick Diouroup (PMOC); Kaolak, Nioro du Rip (PMOC); Sebikotane Dakar (MHNL); Sebikotane (MNHN); south, 10 km SW Medina Gounass (PMOC); Zinguinchor 18m Boukithingo (PMOC).

**Description.** Refer to the Supporting Information Data S5 and S6, original description [56] and Onthophagini Synopsis [2].

**Distribution.** The species is known from Western Africa (Figure 7B).

- ***Tiaronthophagus zambesianus* n.sp.**

Zoobank Registration: http://zoobank.org/urn:lsid:zoobank.org:act:C7753587-2A13-4911-8564-A516BEFFFAF2

**Figure 13:**
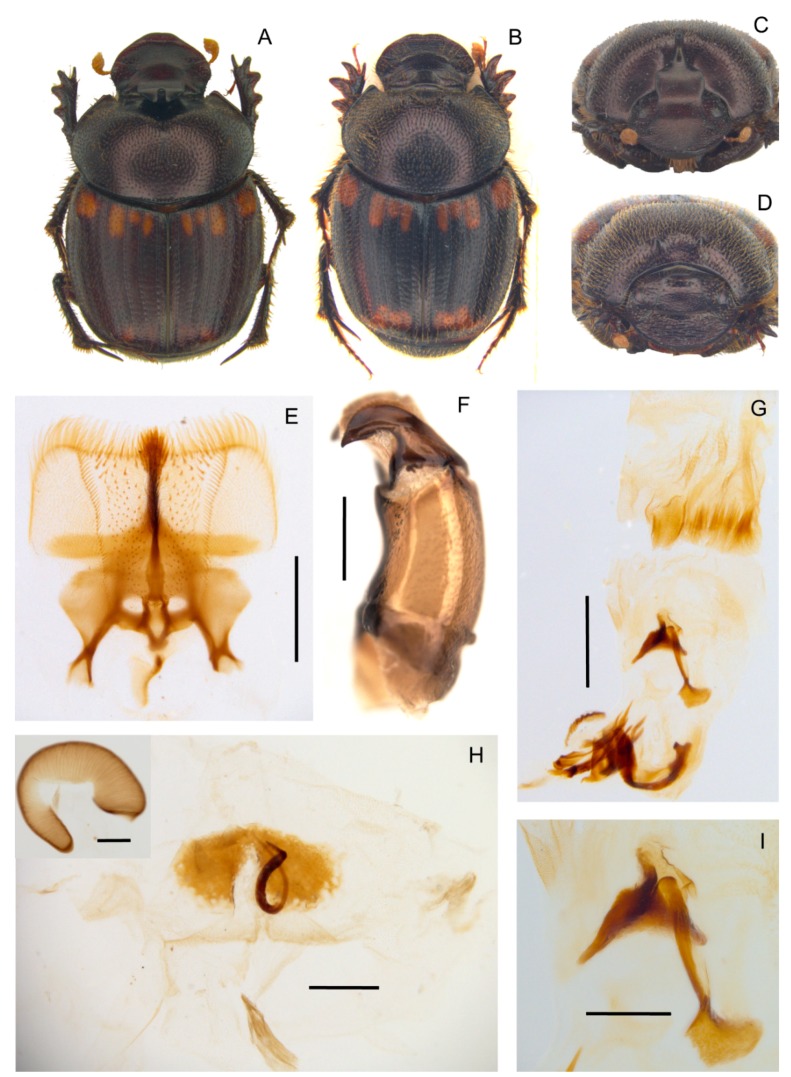
Features of *Tiaronthophagus zambesianus* n.sp. (**A**): Male facies (above). (**B**): Female facies (above). (**C**): Male facies (frontal view). (**D**): Female facies (frontal view). (**E**): Epipharynx, scalebar = 0.4 mm. (**F**): Male genitalia, phallobase and parameres, scalebar = 0.5 mm. (**G**): Male genitalia, endophallus, scalebar = 0.5 mm. (**H**): Female genitalia, vagina, scalebar = 0.3 mm, and receptaculum seminis, scalebar = 0.1 mm. (**I**): Male genitalia, lamella copulatrix, scalebar = 0.2 mm.

Figure 7 and Figure 13, Supporting Information Data S5 and S6

**Type material. Holotype.** ♂ ‘21.xi.2010 1280 m 9 km S Lusaka 15°30′11″ S, 28°15′52″ E, Zambie (Lusaka S.Rojkoff réc.’ ‘*Tiaronthophagus zambesianus* n.sp. holotype’ (BMNH). **Paratypes.** 1♂ ‘21.xi.2010 1280 m 9 km S Lusaka 15°30′11″ S, 28°15′52″ E, Zambie (Lusaka S.Rojkoff réc.’ ‘*Tiaronthophagus zambesianus* n.sp. paratype’ (JFJC); 1♂ ‘Zambie Lusaka Prov. Lower Zambesi N.P. 14-16-xii-2012 15°22’ S, 29°18’ E, leg. Josso Juhel Minetti’ ‘*Tiaronthophagus zambesianus* n.sp. paratype’ (JFJC); 1♀ ‘Zambia Southern province 10km E Zimba 24.-26.12.2002 A. Kudrna jr. lgt.’ ‘*Tiaronthophagus zambesianus* n.sp. paratype’ (PMOC); 1♂ ‘Zambie i.98 Kafue Co. R. Minetti leg. <handwritten>’ ‘*Tiaronthophagus zambesianus* n.sp. paratype’ (PMOC); 1♂ ‘Zambie, Kafue Co. i.98 R. Minetti leg.’ ‘*Tiaronthophagus zambesianus* n.sp. paratype’ (PMOC); 3♂ 6♀ ‘Zambie 17 km N Choma I. Bruce–Miller farm 16°38.22’ S, 27°01.51’ E, 26xi-7xii-2013 1170 m leg. Josso Juhel Minetti’ ‘exc. humains’ ‘*Tiaronthophagus zambesianus* n.sp. paratype’ (JFJC); 3♂ 2♀ ‘N Malawi Vwaza Marsh Reserv Rumphi dist. 1150 m 17-18-i1201 J-F Josso rec.’ ‘poisson pourri’ ‘*Tiaronthophagus zambesianus* n.sp. paratype’ (JFJC); 1♂ 1♀ ‘Karoi Vuti env. Zimbabwe 18-xii-1998’ ‘*Tiaronthophagus zambesianus* n.sp. paratype’ (JFJC); 2♀ ‘Zimbabwe bor. Karoi Vuti env. 18.12.98 lgt. Smrž’ ‘*Tiaronthophagus zambesianus* n.sp. paratype’ (JFJC); 1♂ 1♀ ‘Tanzania, 4.xii.1999 Dodoma prov. near Itigi K. Werner & R. Lizler leg.’ ‘*Tiaronthophagus zambesianus* n.sp. paratype’ (PMOC); 1♂ 2♀ ‘Tanzanie, Iringa prov. Chimala savane 1674 m 20.ii.2004 8°52.510’ S, 33°59.453’ E, Ph.Darge leg.’ ‘*Tiaronthophagus zambesianus* n.sp. paratype’ (PMOC); 5♂ 4♀ Tanzanie, Iringa prov. Chimala, 1000 m 22.ii.2004 R. Minetti leg.’ ‘*Tiaronthophagus zambesianus* n.sp. paratype’ (PMOC).

**Other material.** No other material was examined.

**Etymology.** The species was named after the collection area.

**Description.** Male (Figure 13A,C): length 9–11 mm. Body brownish black, covered by a short, thick, testaceous pubescence, with some small testaceous patches at base of elytra (on interstriae 1–3, and 5–6), and at apex of elytra (barely visible, on interstriae 1–3). Head surface smooth and covered by small points mixed to few larger ones; frontal carina slightly curved. sometimes almost inapparent in major males, more evident in minor males; genae not developed, slightly rounded; vertex carina lamina-shaped, squared, with sides lightly curved, digitiform apex relatively long and arched in major male, while the carina is shaped as a short, narrow rectangular protuberance with the superior edge truncated in minor male. Medium sized, slightly rounded eyes. Antennae yellowish brown. Pronotum covered by dense granulate setigerous points, with large, ovalar; in major males a smooth, concave area in anterior part; pronotum base with a tight row of evident points. Elytra testaceous and black, first two interstriae without granules, almost smooth, the others being covered by sparse, regularly spaced setigerous points with small granules. Pygidium black, with dense, large, and deep puncture. Legs blackish brown with short, thick testaceous setae.

Female (Figure 13B,D): length 9–11 mm. Body as in male. Head surface is wrinkled on clypeus with some large points, less rough with small mixed to large points on the remaining parts; genae not developed, slightly rounded; frontal carina rounded, elevated, thick; vertex carina thick, rectangular, elevated, narrow; area between the carinae smooth with relatively dense, small points simple. Pronotum granulation as in male, two small, conical protuberances that slightly diverge. Elytra and legs as in male. Pygidium shorter than in male, but same colour and puncture.

Epipharynx (Figure 13E): anterior margin slightly concave, without central notch, acropariae constituted by very long, thin setae, slightly tapering toward the zygum; evident zygum that is constituted by a tuft of long thick setae; proplegmatium triangular shaped, sides rectangular; anterior epitorma thin, rectilinear in distal half, slightly expanded, bottle-shaped in proximal half; plegmatic area ovalar, small, very lowered; chaetopariae only slightly sinuate, proximal half with very short setae, longer on distal half; apotormae barely visible, thin, rod-shaped, short; pternotormae short and thick; crepis asymmetrical, small, left-turned, apex blunt; laeotorma and dexiotorma symmetrical, short, and thick; haptomerum with many short and thick setae.

Male genitalia (Figure 13F,G,I): phallobase cylindrical, short, with an anterior rectangular expansion; parameres symmetrical, arched, tapering to the sharp apex; endophallus with a greatly developed raspula constituted by very long, thin setae, lamella copulatrix (Figure 13I) present, well-sclerotized, constituted by two distinct parts; accessory lamellae present and well-developed.

Female genitalia (Figure 13H): vagina membranaceous, triangular-shaped with a symmetrical, bilobed, short infundibular wall, with a large, rounded notch at base; infundibulum question-mark shaped, rectilinear, thick, distal part to ovarioles upward turned; receptaculum seminis not much expanded, tapering to the apex carrying a very small expansion, large desclerotized area placed in the central third.

**Distribution.** The species distribution covers the Southern East Africa (Figure 7D).

- ***Tiaronthophagus zavattarii* (Müller, 1939) n.comb.**

*Onthophagus zavattarii* Müller, 1939: 266.

**Type material**. **Lectotype.** ♂ (here designated) ‘Aresc <sic> aprile 1937’ ‘Missione Zavattari nel Borana ROI’ ‘*Onthophagus rufobasalis* Fairmaire <handwritten>’ ‘Onthophagus zavattari n.sp. <handwritten>’ ‘Lectotype *Onthophagus zavattarii* Müller, 1939 Roggero, Moretto, Barbero, Palestrini 2019’ ‘*Tiaronthophagus zavattarii* (Müller, 1939) Roggero, Moretto, Barbero, Palestrini 2019’ (MCST). **Paralectotypes.** 2♂, 2 ♀, ‘Neghelli 22–25.x.37’ ‘Sped. Brunelli Somalia Mer. leg. Vatova’ ‘Paralectotype *Onthophagus zavattarii* Müller, 1939 Roggero, Moretto, Barbero, Palestrini 2019’ ‘*Tiaronthophagus zavattarii* (Müller, 1939) Roggero, Moretto, Barbero, Palestrini 2019’ (2♂, 2 ♀, MCST); 1 ♂, ‘Neghelli 31.x.37’ ‘Sped. Brunelli Somalia Mer. leg. Vatova’ ‘Paralectotype *Onthophagus zavattarii* Müller, 1939 Roggero, Moretto, Barbero, Palestrini 2019’ ‘*Tiaronthophagus zavattarii* (Müller, 1939) Roggero, Moretto, Barbero, Palestrini 2019’ (MCST).

**Other material.** ETHIOPIA. Sidamo, 25 km E Negele 7–10.v.2016 S. Prepsl leg. (JFJC).

**Description.** Refer to the Supporting Information Data S5 and S6 and original description [57].

**Distribution.** The species is distributed in Ethiopia (Figure 7F).

**Notes.** A marked similarity of this species with *T. rufobasalis* [57] was highlighted, although the two species can be well differentiated by some characters, such as the features of the vertex carina in the females, and the pronotum punctures (Supporting Information Data S5).

## 4. Discussion

The results of the present analyses for *Tiaronthophagus* were congruent to those that were previously obtained for other Onthophagini [15,16,17,18,19], in which it was already suggested that the genus *Onthophagus s. l.* (according to current understanding and specific composition) cannot be considered as a monophyletic taxon [7,20]. According to those finding, it seems obvious that the systematic position of the Afrotropical *Onthophagus* members should be examined in detail for all of the species-groups proposed in the past [2] to evaluate their phylogenetic relationships. In this framework, the analyses should perhaps also be extended to the whole genus, although being unlikely to happen in the near future. Due to the large number of species that were currently included in the worldwide *Onthophagus* genus, and the extremely diversified features of these taxa, it would be difficult to study them all together at once.

Some interesting results were already evinced here within the outgroup dataset, as regarding the relationships between *Onthophagus s. str.* and *Palaeonthophagus*, which constitute two distinct clades in the phylogenetic tree (Figure 3). The present findings could also suggest that *Palaeonthophagus* and *Onthophagus s.str.* must be regarded as distinct genera, as the other Onthophagini taxa included in our analyses. The hypothesis is challenging and it deserves a more careful evaluation in the future, but it is far beyond the purpose of our present analysis, thus we mainly focused on *Tiaronthophagus*, which is the new genus identified here.

Noteworthy, both *Onthophagus s.str.* and *Palaeonthophagus* are well-separated from the *Tiaronthophagus* on the tree. In our analysis, *Hamonthophagus/Morettius* constitutes the sister clade of *Tiaronthophagus*. This is rather interesting, since species now belonging to *Hamonthophagus* were examined in the past using molecular data [4], and then it was already suggested that they are not at all related to *Onthophagus*. This hypothesis was later confirmed by other (both morphological and molecular) phylogenetic analyses [17,18].

### 4.1. Combination of Characters

A unique combination of 20 diagnostic characters of internal and external morphology defines *Tiaronthophaus* (Figure 6, Supporting Information Data S5 and S6). The glabrous ovalar area on the pronotum basal sides, matched to a similar depressed area on elytra near the humeral callous (Figure 6A,D), is a rather good character for species attribution to *Tiaronthophagus*. Although a bare area would be present in some other Onthophagini taxa, as a rule, it is differently shaped and located on the pronotum surface, and often there is not a corresponding concave area on the elytral surface.

Another fair example of diagnostic character is the head horn of major males, which is well-developed and it shows a marked uniformity in these species, carrying a vertex carina that is modified into an elongate, flat, more or less sinuate lamina. The presence of such horns, coupled with other characters, such as the presence of some few very large points on the clypeus and genae, can be very useful in the identification of the *Tiaronthophagus* species. The head weaponry displays a uniform model (the laminar, sinuate horn) within the genus, but nevertheless it is quite differentiated at the species level, thus being very useful for species identification (Supporting Information Data S5).

The female clypeus, which is far rougher than the other parts of the head, and the short and thick vertex carina, which is also well differentiated at the species level (Supporting Information Data S5), can be usefully employed for the identification of *Tiaronthophagus* taxa.

The epipharynx features are, as usual, well-characterized (Figure 6K), thus it should be employed to define the taxonomic attribution to the new genus, even though this anatomical trait is currently not so widely employed. Additionally, the genitalia of both sexes carry useful diagnostic characters for identification at the genus and species level (Supporting Information Data S6), with the most noteworthy of them being the parameres, the upper margin of the phallobase, and the lamella copulatrix in males (Figure 6L,M, Supporting Information Data S6), and the infundibular wall and receptaculum seminis features in females (Figure 6N,O, Supporting Information Data S6). Furthermore, on the posterior surface of the vagina, there are two well-developed, symmetrical expansions (Figure 6O) that characterize the genus.

The features of the *Tiaronthophagus* species maintain a common pattern of morphological differentiation within the genus, as all of these species were nevertheless well distinguished. The detected morphological differences lead to the identification of sets of species thatare more closely allied within the genus, with the structures here examined, thus also providing useful indications regarding the taxon attribution at a supra-specific level. The definition power of these anatomical traits, which have proven highly discriminant, was quantified using the geometric morphometrics approach.

### 4.2. Landmark Characters Survey

The twelve structures that were included in the phylogenetic analysis as landmark characters were chosen among those usually employed in Scarabaeidae systematics [20,58] for they can be accurately described using the geometric morphometrics approach. A detailed survey allowed for us to evaluate the overall shape variation and the differentiation patterns for each structure. According to the present outcomes, some structures were more differentiated than others, thus providing a more detailed discrimination at a generic and specific level, but both the PCA and CVA results showed an evident separation of the two defined groups (i.e., ingroup and outgroup taxa being well differentiated) for each anatomical trait.

In detail, the hindwing, epipharynx, female head and pronotum, and phallobase (100% cross-validated cases correctly classified by CVA) were confirmed to be useful characters for taxa identification at various taxonomic levels, followed by the parameres (97%), elytra (95%), major male pronotum (94%), and head (92%).

For the male genitalia, the arched, pointed parameres with a large, rounded ventral notch, and the phallobase with a plate-shaped ventral expansion allowed for optimal separation of the ingroup and outgroup taxa. Noteworthy, this well-developed, characteristic ventral projection of phallobase (Figure 6L,M) could suggest its involvement in the coupling mechanism, which is a complex phenomenon only marginally studied till now [59,60].

The usefulness of the epipharynx in taxa identification is well known, as it has already been assessed many times in different Scarabaeoidea taxa [38,61,62]. Here again, the ingroup and outgroup taxa showed impressive shape variations for this anatomical trait, and in the scatterplot, the relative relationships among the taxa were fully highlighted, also distinguishing different patterns within the outgroups (i.e., the different genera), therefore the structure again confirmed its high-discriminant power.

The shape variation patterns defined for the hindwing allowed a clear separation between the ingroup and outgroup taxa, also confirming differences within the outgroups. The usefulness of wing features in taxonomy and phylogeny has long been recognized in Scarabaeidae, but investigations regarding wing shape evolution in this family using geometric morphometric approach were carried out only recently [24,63,64]. The results of the analysis confirmed that the hindwing could be a good predictor of the phylogenetic relationships, as formerly evaluated at various taxonomic ranks [58,63,64].

For the elytra, a lower discriminant effectiveness at both species and genus level was obtained, as the groups were rather overlapping, with similar shape variation patterns. The forewings can contribute to the taxa discrimination at a lesser rate than the hindwings, although the whole dataset accounted for 95% of the correctly classified cases (meaning that a large number of RWs accounted for the overall shape variation). It should be worth to remember that, as a rule, in taxa that are phylogenetically close, the elytra features commonly used for the identification are mainly related to the variation in colour, puncture, and pubescence than in form. Thus, the elytra shape variation deserves a careful evaluation to gain a better definition of the form variant patterns of this structure.

The shape variation patterns of the head are similar in both sexes, also with a common pattern in *Tiaronthophagus* for this anatomical trait. In the plots, different relationships among taxa were defined for the two sexes, showing that female heads have a more marked shape differentiation than males, both between and within the proposed groups. Ostensibly, other factors (such as the development of sexual secondary traits) could have influenced the differentiated head shape in males due to the presence of exaggerated weaponry [65,66,67]. The same patterns that were already highlighted for the head were also observed for the pronotum, and likely the same factors could contribute to define the variation in shape between the ingroup and outgroup taxa, as well as the difference in the discrimination power for the sexes in both groups.

Among the examined anatomical traits, only the shape of the eyes was slightly less effective in defining the groups for both sexes (86% in females and 88% in males). Perhaps the minor discrimination of this structure could be ascribed to the sensitivity to different factors that intervene at various levels [68]. The eyes are useful in taxa discrimination, and a detailed examination of the growth mechanism for these structures within *Tiaronthophagus*, as well as a comparison between the shape (and size) variation of the head lamina and eyes in males [67,68,69], could furnish useful information.

### 4.3. The Phylogenetic Analysis

All of the chosen landmark characters were included together in the matrix for the phylogenetic analysis. The combined phylogenetic analysis allowed for us to define a single fully resolved tree subdivided into distinct clades, in which congruent patterns were highlighted, although some of the phylogenetic relationships among the species were not completely resolved. Besides, the support values (Figure 3) confirmed that *Tiaronthophagus* constitutes a distinct, monophyletic clade within Onthophagini, which is clearly separated from the other genera examined here.

In the phylogenetic tree, each clade is rather homogeneous in external and internal features, as the epipharynx, genitalia (mainly, the male lamella copulatrix, and female infundibular wall), female vertex carina, pronotal, and elytral interstriae puncture. Besides, some species surely belonging to the genus *Tiaronthophagus* were not close to any other species, but rather constituted isolated branches. The latter results could perhaps suggest that some *Tiaronthophagus* species remain unknown. An increasing knowledge of the Afrotropical Onthophagini taxa thus would surely contribute to a better definition of the phylogenetic relationships within the *Tiaronthophagus* species.

### 4.4. Weaponry Diversity

Sexual selection has generated spectacular male weaponry diversity within the tunnelling Onthophagini species, which show a guard behaviour of the female that led to the development of weapons (secondary sexual traits), such as long horns. In the male-male reproductive competition, weapons are used to keep out the rival males from the burrows by blocking their access.

**Figure 14:**
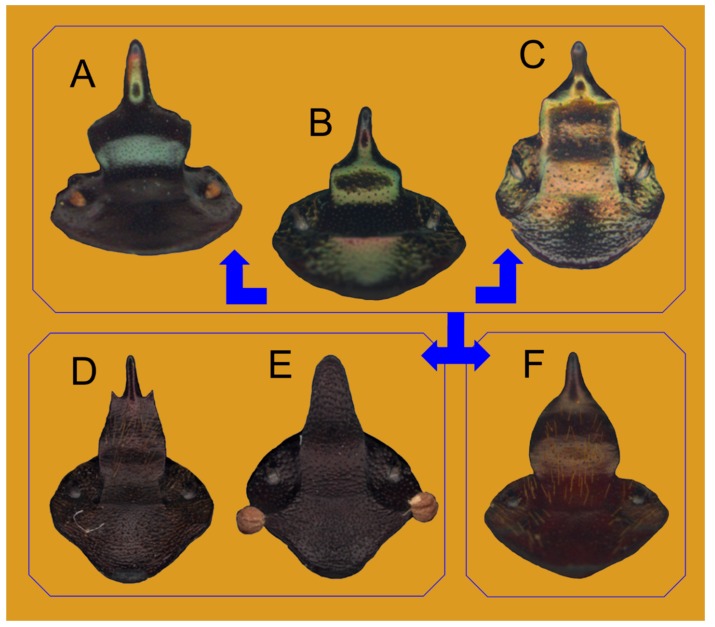
Some examples of the variation pattern of the horn models in *Tiaronthophagus* major males. (**A**): *T. rufobasalis*. (**B**): *T. chrysoderus.* (**C**): *T. naevius*. (**D**): *T. jossoi*. (**E**): *T. rufopygus*. (**F**): *T. liberianus*—Three major model variation were grouped by boxes, and the arrows suggest pot vvential variation patterns. The B model of lamina is relatively short, and it widens into the A model and then elongates into C model. More obvious changes in shape can be seen in D–E and F models.

In these taxa, a single horn originating from the base of the head, as the one of *Tiaronthophagus* males is commonly considered to be the ancestral form [70,71], which splits into a number of main derived, extremely differentiated patterns, even in closely related species. The horns may markedly differ in the shape, location, and numbers, giving rise to an intense and impressive radiation of the weapon morphology [65,67,70]. According to what was suggested by previous research on horn evolution in the genus *Onthophagus* [70,71], one would have expected more variegate examples of horns in *Tiaronthophagus*, yet the genus is characterized by a uniform horn model (Figure 14).

The unique ancestral horn pattern (i.e., a large, flat and sinuate lamina) radiated into some little-diversified morphotypes in *Tiaronthophagus* (Figure 14). The most common one, as shared by the majority of the species, with some slight variations (Figure 14A–C), may be considered to be ancestral, while the other (derivate) morphotypes are present in some species where the major male vertex carina becoming (e.g.,) narrower and straightened (as in *T. jossoi* and *T. rufopygus*, Figure 14D,E), or shortened and ovalar (as in *T. liberianus*, *T. macroliberianus,* and *T. pseudoliberianus*, Figure 14F). The intense directional sexual selection leading to the different weaponry displayed by many Onthophagini taxa is seemingly not very relevant in *Tiaronthophagus,* which contradicts this common scheme.

A suggestive hypothesis affirming that weapon shapes reflect structural adaptation to different fighting styles was recently tested [66], highlighting how horns would be stronger and stiffer in response to species-typical fighting. A link between weapon form and function was thus suggested [66]. In this framework, it could be hypothesized that the evenness of horn model that was displayed by *Tiaronthophagus* would also mean that the male-male competition follows a unique, very homogeneous model of reproductive behaviour in this genus.

### 4.5. Biogeographical Analysis and Ecological Considerations

**Figure 15:**
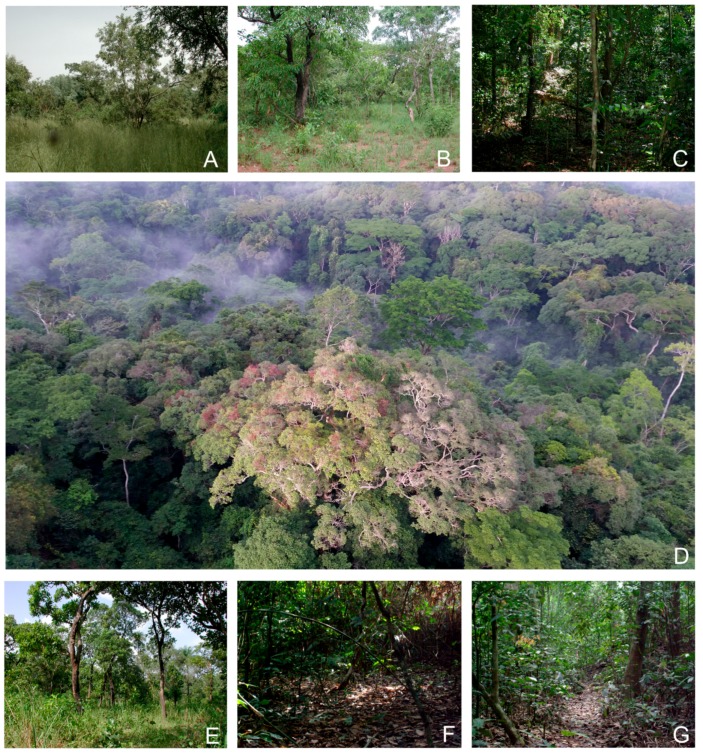
Photos of the habitats of some *Tiaronthophagus* species (by Philippe Moretto). The species collected in each locality are reported in brackets. (**A**): Sénégal, Kolda, Forêt de Bakor (*T. chrysoderus*, *T. viridiaereus*, *T. rougonorum,* and *T. flexicornis*). (**B**): Côte d’Ivoire, Dabakalla, Ouanderama (*T. lamtoensis, T. curtipilis* and *T. flexicornis*). (**C**): Côte d’Ivoire, Taï N.P. (*T. liberianus* and *T. rufopygus*). (**D**): Côte d’Ivoire, Man, Mt. Tonkoui (*T. liberianus* and *T. rufopygus*). (**E**): Côte d’Ivoire, Touba, Biémasso (*T. lamtoensis, T. liberianus, T. flexicornis* and *T. rufostillans*). (**F**,**G**): Ndoki, Centrafrique (*T. pseudoliberianus* and *T. macroliberianus*). A,B: Soudanian wooded savannahs and forests. E: Savana-forest Guinean mosaic. C,F,G: Primary wet forest. D: Primary Guinean montane forest.

The genus is widely spread on the whole Afrotropical region, with some species having a large distribution (as *T. rufobasalis*), while others are characterized by a far more reduced one (as *T. naevius* or *T. angolensis*). According to the phylogenetic results and the known distributions, each of the ten areas that were identified by InfoMap showed different species diversity, with a variant combination of common and indicator species (Table 2). The macroarea A, coarsely corresponding to the whole W Africa, comprehends the majority of the species, but also the East Africa Rift is rather rich in species. For some species, habitat photos are provided (Figure 15), evidencing how the species from W Africa live in forest habitat.

The various clades that are defined by the phylogenetic analysis are characterized by a diversified overall distribution (Figure 7). The four species that were included in the clade *T. zambesianus*/*T. rufobasalis* extend in the whole E Afrotropical area from the Somali-Masai region to the Cape area. In contrast, the clade *T. pseudoliberianus*/*T. saadaniensis*, consisting of six species, are present from W Africa to E Africa till the Zambesian region southward. The clade *T. aequatus*/*T. schaufussi* instead shows a disjoint distribution, widely extending southward in E Africa from Ethiopia to Zambia (similar to the former clade) and to Angola westward, with only *T. rufostillans* in NW Africa. Thus, these latter clades have the widest distribution, almost covering the known geographic area of the whole genus.

The biogeographical analysis highlighted a defined pattern mainly related to dispersal events in both Western and Southern Afrotropical region, and only few vicariance events. This is a relatively common case in the Afrotropical Scarabaeidae, as already evidenced in the past in other taxa [18,58], The VIP analysis (consensus reconstruction) suggested that the ancestral area of the genus should be in Central Africa, and extending into the whole sub-Saharan Africa by dispersal events. According to the results, a recent expansion can thus be hypothesized for those species [18,24,72]. Although various vicariance events were proposed in the OR reconstructions by VIP, they were not confirmed by the consensus reconstruction, except for the case of the nodes 18 and 4 of the phylogenetic tree (Supporting Information Data S4). The first vicariance event (node 18) refers to *T. angolensis* (endemic to SW Africa) vs the clade *T. zavattari*/*T. schaufussi* (E Africa). The second case (node 4) covering refers to a vicariance between *T. liberianus* vs *T. macroliberianus*, in W Africa vs CW Africa. The vicariance events thatare involved only some species in the Western part of the Afrotropical region, with the present distribution of *Tiaronthophagus* mainly depending on dispersal events.

The species that were currently included in *Tiaronthophagus* also share some peculiar similarities in their feeding habits, as they were often collected from small carrion, including dead diplopods during the second stage when quinones are totally evaporated and the diplopods becomes palatable decaying carcasses. For many of these species, a necro-coprophagous behaviour was thus suggested, as achieved by the collection data on the labels and the field observations that were corroborated by numerous trappings using various baits (e.g., *T. aequatus*, *T. rufopygus*, *T. rufostillans*, *T. liberianus*, *T. pseudoliberianus*, *T. flexicornis*, *T. ebenus*, *T.curtipilis,* or *T. rufobasalis*) (J.-F. Josso pers. comm., P. Moretto pers. obs.). Other species, as *T. angolensis*, are instead likely to show true coprophagous feeding habits. Even if less documented for African fauna than for American fauna [73,74], maybe because the involved African species use discreet small carrion, a necrophagous or copro-necrophagous diet is more widespread on the continent than usually believed, especially between Onthophagini, whose several species groups display strong copro-necrophagous feeding behaviour. At this point, it is necessary to be very careful in order to avoid confusion [7] between the guild of millipedes-dependant species, attracted by quinones that are emitted by living or decaying diplopods, and using fresh content of these animals, then being more likely carnivorous and even predators, and the guild of species attracted to small carcasses (lizards, snakes, toads, millipedes, and so on) and using decaying flesh, which is the case of the necrophagous and copro-necrophagous *Tiaronthophagus*.

There is an ecological succession in the use of diplopods: necrophagous species are attracted to the carcass after the millipede-dependant species, never the contrary. Even if necrophagous feeding habits are not the proof that decaying meat is used to feed the larvae, there is a high probability that some species be necrophagous during both the adult and larval stages, as we can infer from their behaviour. For example, the common *T. lamtoensis* will be attracted to a trap that is baited with diplopods carcasses only, even if this trap is close (i.e., few meters) to a trap that is baited with human excrement, while opportunist copro-necrophagous species will be attracted to both traps. Notheworthy, the species of *Tiaronthophagus,* which are strongly suspected to be true necrophagous, are in the most basal position in the tree (*T. hemichlorus*, *T. lamtoensis*, *T. chrysoderus*, *T. viridiaereus*, *T. flexicornis*, *T. curtipilis*, and *T. rougonorum*). A part of these species shows two more or less developed teeth in the middle of the clypeus while this character is absent in all of the other *Tiaronthophagus* species. This character is shared by many necrophagous or copro-necrophagous species in several genera of Scarabaeinae (*Catharsius* and Onthophagini in Africa). In the most part of African millipedes-dependant Onthophagini, the clypeus is instead never bidentate, and the head is totally unarmed, or carries at least a short triangular lamina on the vertex.

The coprophagous habits, matched to a relatively simple tunnelling behaviour, as found in Onthophagini, were considered to be ancestral, while the necrophagy is commonly regarded as a more recently evolved behaviour [7]. Likely, the definition of necrophagy does not entirely cover its biological complexity, and thus some peculiar feeding habits have not yet been fully understood. The many examples of taxa that are associated with feeding on millipede carrion mainly in the Afrotropical region may signify a more ancient origin [7]. The two different necrophagous behaviours that were detected in these Afrotropical taxa corroborated the hypothesis that the necrophagous feeding originated more than one time independently. Surely, the behavioural aspects of the *Tiaronthophagus* feeding and nesting would deserve a thorough survey in order to define the mechanism behind these necro-coprophagous habits in the genus.

## 5. Conclusions

In brief, *Tiaronthophagus* constitutes a well-defined taxon within the Onthophagini, which is surely separated from the genus *Onthophagus*. The new genus is easily identifiable by a set of exclusive diagnostic characters. The generic rank that is assigned to the taxon is supported by the results of the morphological, phylogenetic, and biogeographical analyses. However, according to the present results, the whole Onthophagini should be carefully examined, since the classification of this speciose tribe remains contentous and poorly supported. Noteworthy, the recent improvements of the knowledge about Onthophagini made it even more obvious that thorough evaluation of the systematics and phylogenetic relationships of these taxa is in great need.

## Figures and Tables

**Table 1:** List of the ingroup and outgroup species included in the analysis. The species were classified as Palearctic (PA) or Afrotropical (AF), according to their distribution (D). Part of the ingroup species were formerly included in three different d’Orbigny *Onthophagus* groups [2]: 14 in the 24th, four in the 28th, and two in the 16th group, respectively.

	D	Outgroup	Intgroup		
			24th Group	28th Group	16th Group
*Euonthophagus flavimargo* (d’Orbigny, 1902)	AF	●			
*Morettius pallens* (d’Orbigny, 1908)	AF	●			
*Hamonthophagus bituberculatus* (Olivier, 1789)	AF	●			
*Hamonthophagus depressus* (Harold, 1871)	AF	●			
*Onthophagus nigriventris* d’Orbigny, 1902	AF	●			
*Onthophagus illyricus* (Scopoli, 1763)	PA	●			
*Palaeonthophagus coenobita* (Herbst, 1783)	PA	●			
*Palaeonthophagus medius* (Kugelann, 1792)	PA	●			
*Palaeonthophagus nuchicornis* (Linnaeus, 1758)	PA	●			
*Palaeonthophagus ovatus* (Linnaeus, 1767)	PA	●			
*Tiaronthophagus aequatus* (Peringuey, 1900)	AF		●		
*Tiaronthophagus angolensis* n.sp.	AF				
*Tiaronthophagus chrysoderus* (d’Orbigny, 1905)	AF			●	
*Tiaronthophagus curtipilis* (d’Orbigny, 1905)	AF		●		
*Tiaronthophagus delahayei* (Josso, 2011)	AF				●
*Tiaronthophagus ebenus* (Peringuey, 1888)	AF		●		
*Tiaronthophagus flexicornis* (d’Orbigny, 1902)	AF			●	
*Tiaronthophagus hemichlorus* (d’Orbigny, 1915)	AF		●		
*Tiaronthophagus jossoi* n.sp.	AF				
*Tiaronthophagus katanganus* n.sp.	AF				
*Tiaronthophagus lamtoensis* (Cambefort, 1984)	AF		●		
*Tiaronthophagus liberianus* (Lansberge, 1883)	AF		●		
*Tiaronthophagus macroliberianus* (Moretto, 2010)	AF		●		
*Tiaronthophagus naevius* (d’Orbigny, 1913)	AF		●		
*Tiaronthophagus pendjarius* (Josso & Prevost, 2006)	AF				●
*Tiaronthophagus pseudoliberianus* (Moretto, 2010)	AF		●		
*Tiaronthophagus rolandoi* n.sp.	AF				
*Tiaronthophagus rougonorum* (Cambefort, 1984)	AF			●	
*Tiaronthophagus rufobasalis* (Fermaire, 1887)	AF		●		
*Tiaronthophagus rufopygus* (Frey, 1957)	AF		●		
*Tiaronthophagus rufostillans* (d’Orbigny, 1907)	AF		●		
*Tiaronthophagus saadaniensis* n.sp.	AF				
*Tiaronthophagus schaufussi* (Harold, 1867)	AF		●		
*Tiaronthophagus viridiaereus* (d’Orbigny, 1908)	AF			●	
*Tiaronthophagus zambesianus* n.sp.	AF				
*Tiaronthophagus zavattarii* (Muller, 1939)	AF	●	●	●	●

**Table 2:** Results of the InfoMap analysis, showing for each macroarea (A–J) the classification defined the common and indicator species. The lists of the species are arranged in decreasing order.

Common Species	Common Species Count	Indicator Species	Indicator Species Ccores	Macroarea
*flexicornis*	20	*viridiaereus*	1.30	A
*viridiaereus*	18	*lamtoensis*	1.30	A
*lamtoensis*	14	*liberianus*	1.30	A
*rougonorum*	13	*rougonorum*	1.30	A
*chrysoderus*	13	*chrysoderus*	1.30	A
*rufostillans*	10	*rufostillans*	1.30	A
*liberianus*	9	*rufopygus*	1.30	A
*rufopygus*	6	*pendjarius*	1.30	A
*pendjarius*	5	*flexicornis*	1.24	A
*curtipilis*	2	*curtipilis*	0.26	A
*pseudoliberianus*	10	*pseudoliberianus*	2.60	B
*macroliberianus*	6	*macroliberianus*	2.60	B
*flexicornis*	1	*curtipilis*	0.26	B
*curtipilis*	1	*flexicornis*	0.12	B
*hemichlorus*	2	*hemichlorus*	13.00	C
*schaufussi*	5	*saadaniensis*	5.20	D
*zavattari*	3	*schaufussi*	5.20	D
*rufobasalis*	2	*zavattari*	5.20	D
*saadaniensis*	1	*rolandoi*	0.58	D
*rolandoi*	1	*rufobasalis*	0.40	D
*rufobasalis*	22	*jossoi*	1.18	E
*aequatus*	2	*rufobasalis*	1.00	E
*zambesianus*	1	*aequatus*	0.18	E
*jossoi*	1	*rolandoi*	0.13	E
*rolandoi*	1	*zambesianus*	0.12	E
*rolandoi*	3	*rolandoi*	2.89	F
*zambesianus*	1	*zambesianus*	0.87	F
*rufobasalis*	1	*rufobasalis*	0.33	F
*naevius*	5	*delahayei*	5.20	G
*aequatus*	4	*naevius*	5.20	G
*katanganus*	4	*katanganus*	4.16	G
*ebenus*	3	*aequatus*	1.60	G
*delahayei*	3	*rolandoi*	1.16	G
*rolandoi*	2	*curtipilis*	1.04	G
*curtipilis*	2	*ebenus*	0.97	G
*zambesianus*	1	*zambesianus*	0.52	G
*zambesianus*	7	*zambesianus*	2.60	H
*aequatus*	7	*aequatus*	2.00	H
*curtipilis*	5	*curtipilis*	1.86	H
*ebenus*	2	*katanganus*	0.74	H
*katanganus*	1	*ebenus*	0.46	H
*rufobasalis*	1	*rolandoi*	0.41	H
*rolandoi*	1	*rufobasalis*	0.14	H
*ebenus*	11	*ebenus*	1.63	I
*rolandoi*	1	*rolandoi*	0.26	I
*angolensis*	7	*angolensis*	3.71	J

## Supplementary Materials

The following materials are available online at https://www.mdpi.com/2075-4450/10/3/64/s1: Data S1. List of the 53 discrete characters used in the phylogenetic analysis, with images of the character states; Data S2. Combined matrix of discrete and 2D landmark characters, formatted according to TNT software requirements; Data S3. List of the synapomorphies common to the phylogenetic tree (Figure 2); Data S4. VIP analysis, the phylogenetic tree (with numbered nodes), and the OR and consensus reconstructions are showed. Please note that the file should be viewed using the option “bookmark ON” in Adobe Acrobat; Data S5. List of the main characters to identify the *Tiaronthophagus* species; Data S6. Plates of the genitalia of both sexes of the *Tiaronthophagus* species.
