# Supplementary material for: The Phylogenetic Relationships of Tiaronthophagus n.gen. (Coleoptera, Scarabaeidae, Onthophagini) Evaluated by Phenotypic Characters
Source: Insects. 2019 Mar 1;10(3):64. doi: 10.3390/insects10030064 (PMC6468411; doi:10.3390/insects10030064)
Supplement: Supplementary file 1 [file insects-10-00064-s001.zip › Roggero Data S1 Discrete Characters List.pdf]

Tiaronthophagus - List of the discrete characters

1. **Epipharynx, the anterior part on the whole:** (0) rounded; (1); squared, almost rectangular; (2) trapezoidal.
2. **Epipharynx, setae of the acropariae:** (0) same length; (1) longer on sides and shorter in the middle.
3. **Epipharynx, the fore margin:** (0) rectilinear, or only slightly rounded in the middle; (1) V-notched; (2) largely triangular notched.
4. **Epipharynx, chaetopedia:** (0) evident, well-developed, constituted by many thick setae; (1) not much evident, reduced, carrying few and shorter setae.
5. **Epipharynx, zygum:** (0) constituted by few long setae; (1) constituted by long and thick setae forming a tuft.
6. **Epipharynx, proplegmatium:** (0) placed in the half of the epipharynx; (1) placed in the hind third.
7. **Epipharynx proplegmatium sclerotization:** (0) trangular shaped, with narrow sides; (1) almost rectangular, being thus thick, enlarged, subequal also on sides; (2) thick, but clearly forming a triangle on ther central third, the sides tapering to vertex.
8. **Epipharynx, apotormae:** (0) present, variously shaped; (1) reduced or absent.
9. **Epipharynx, chaetopariae setae:** (0) almost equal on the whole length; (1) far longer near the fore margin, then narrowing to base.
10. **Epipharynx, anterior epitorma:** (0) narrow on the whole length, rod-shaped; (1) enlarged, triangular-shaped about the basal half; (2) largely elongate, ovalar at basal third; (3) basally rounded, or largely ovalar expanded on sides.

Tiaronthophagus - List of the discrete characters

11. Epipharynx, laeotorma and dexiotorma: (0) more or less asymmetrical; (1) symmetrical, almost alike.

12. Epipharynx, laeo- and dexiotormae: (0) short and stout; (1) elongate and more slender.

13. Epipharynx, laeo- and dexiotormae joining part to mandibles: (0) very large, ovalar-rounded; (1) smaller, triangular-shaped.

14. Female genitalia, receptaculum seminis [RS]: (0) equal diameter on the whole length; (1) uneven diameter.

15. Female genitalia, RS overall shape: (0) not lobate nor expanded at base; (1) plurilobate and expanded at base; (2) plurilobate, more expanded in the middle; (3) enlarged at base, but not plurilobate; (4) plurilobate, but not expanded at base.

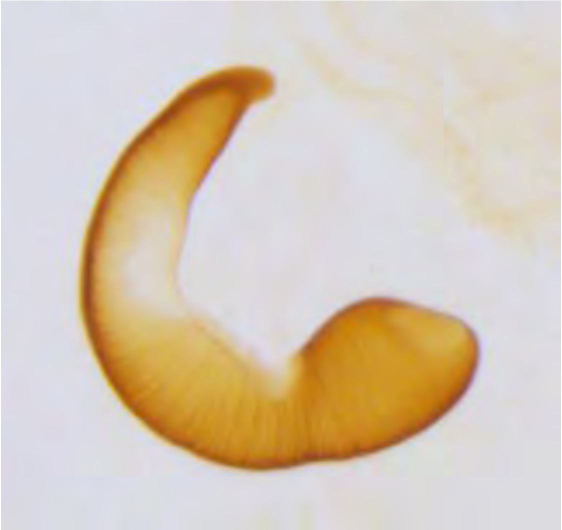

(15.1)

T. aequatus

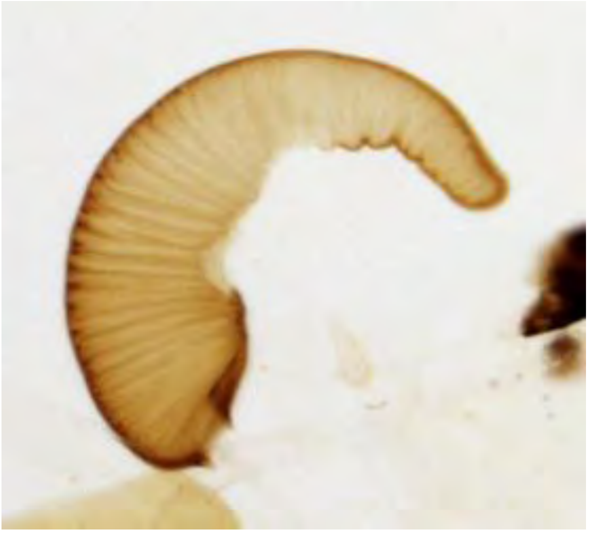

(15.2)

T. curtipilis

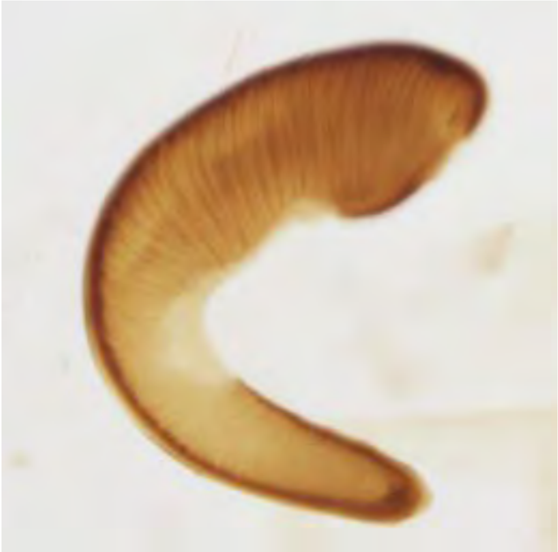

(15.3)

T. katanganus

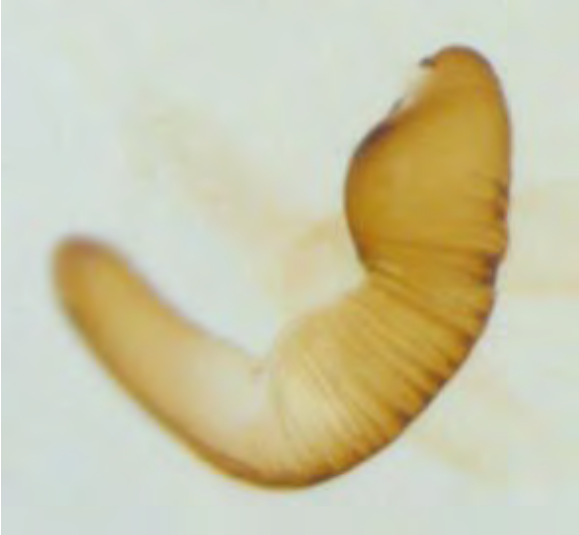

(15.4)

T. liberianus

Tiaronthophagus - List of the discrete characters

16. Female genitalia, RS apex: (0) blunt; (1) sharp.
17. Female genitalia, RS connection to infundibular tube with tube insertion: (0) in the middle, not lateral, sometimes with initial sclerotized joining part; (1) lateral, with basal portion usually expanded, the joining part membranaceous.
18. Female genitalia, shape of the vagina on the whole: (0) high or very high, and rounded; (1) short and flat, sometimes with apical expansion; (2) short and triangolar-shaped; (3) short and rounded.
19. Female genitalia, two symmetrical lateral protrusions: (0) absent; (1) present, triangular-shaped; (2) present, rounded and large.
20. Female genitalia, if two protrusion present, then: (0) membranaeous, without setae; (1) carrying thick small setae, and sclerotized; (2) rounded and membranaceous, but carrying two well-sclerotized, narrow diverticula.

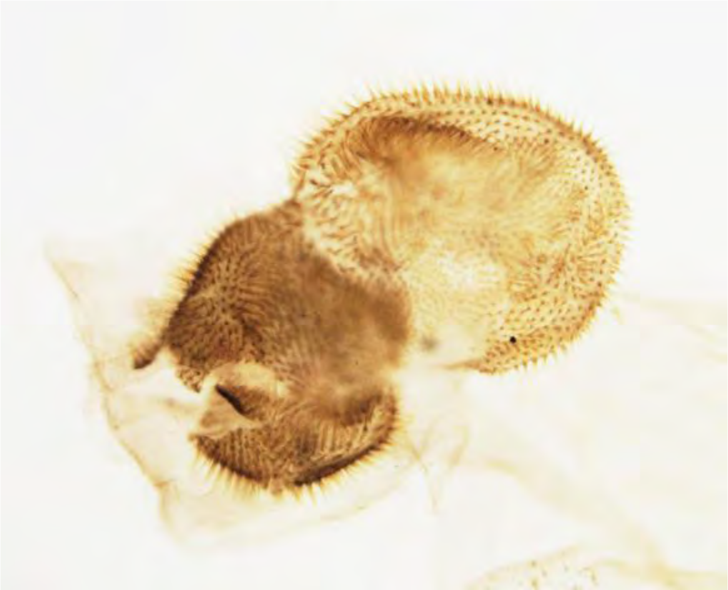
(20.1)

T. pseudoliberianus

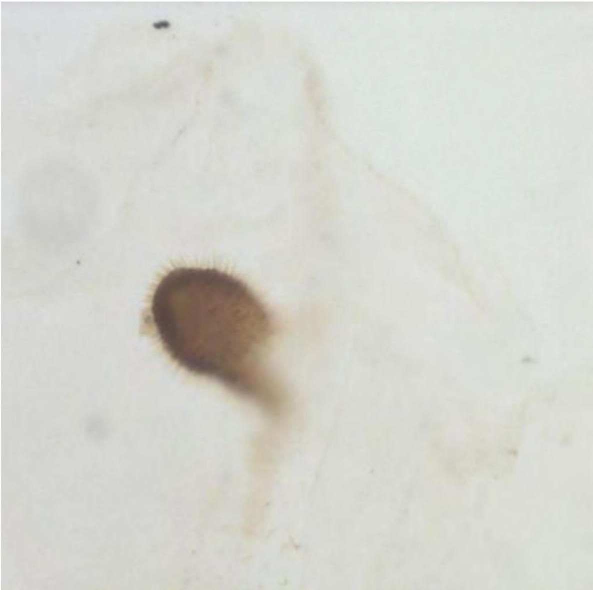
(20.2)

T. delahayei

Tiaronthophagus - List of the discrete characters

21. Female genitalia, infundibular tube: (0) tubuliform, scarcely sclerotized, not rigidly folded; (1) question mark shaped with a small loop, sclerotized, rigidly folded; (2) question mark shaped with a large loop, sclerotized, rigidly folded.

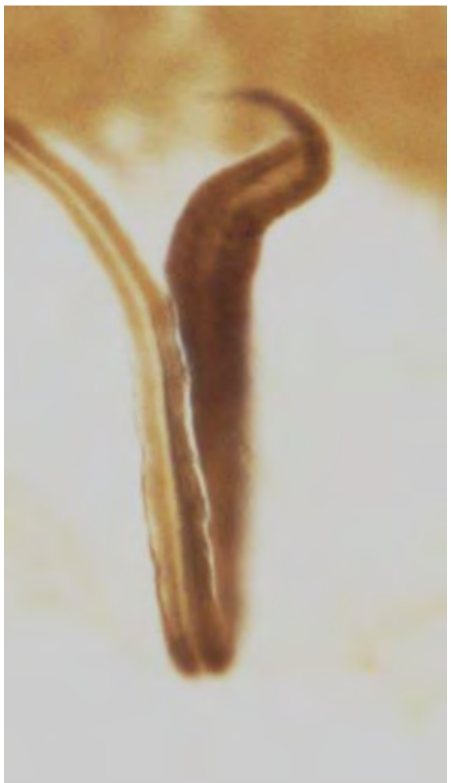

(21.1)

T. ebenus

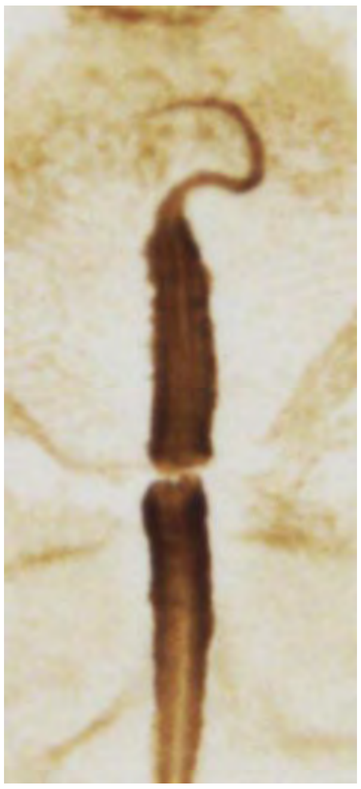

(21.2)

T. rougonorum

22. Female genitalia, infundibular tube inferior part: (0) largely arched; (1) strongly upturned with a narrow folding.

23. Female genitalia, infundibular tube medial part: (0) not sclerotized, variously folding, different size and shape; (1) well-sclerotized, rectilinear, thick and relatively large.

24. Female genitalia, infundibular wall sclerotization: (0) present, evident and well-defined sclerotization in central area; (1) present, but thin sclerotization not defined, and extending on the vagina surface; (2) absent, vagina entirely membranaceous; (3) absent, but then well-sclerotized supporting structure internally.

Tiaronthophagus - List of the discrete characters

25. Female genitalia, infundibular wall sclerotization: (0) short; (1) elongate.

26. Female genitalia, infundibular wall sclerotization: (0) H-shaped; (1) U reverse shaped, narrow, not expanded, the upper part often squared; (2) arched, thick, the sides sometimes slightly enlarged; (3) triangular-shaped, sometimes the apex enlarged on sides (mushroom-shaped); (4) bilobed; (5) paddle-shaped.

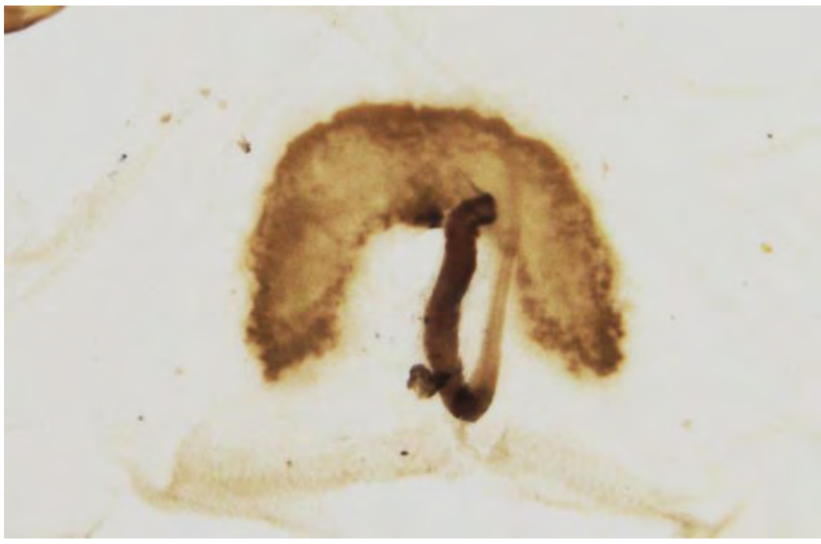

(26.2)

T. rufostillans

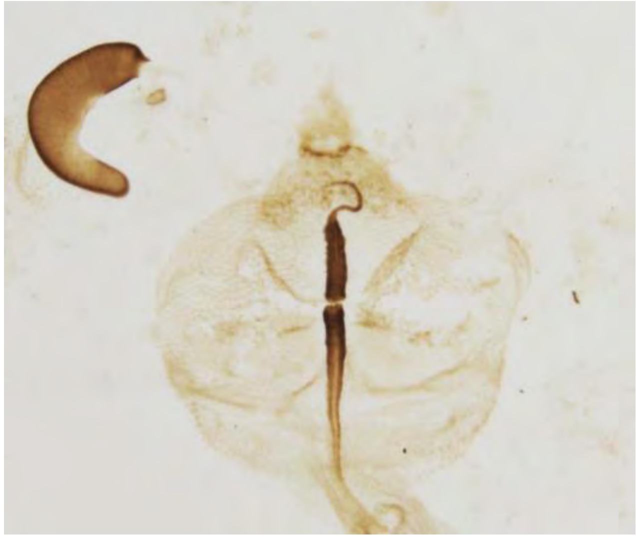

(26.3)

T. rougonorum

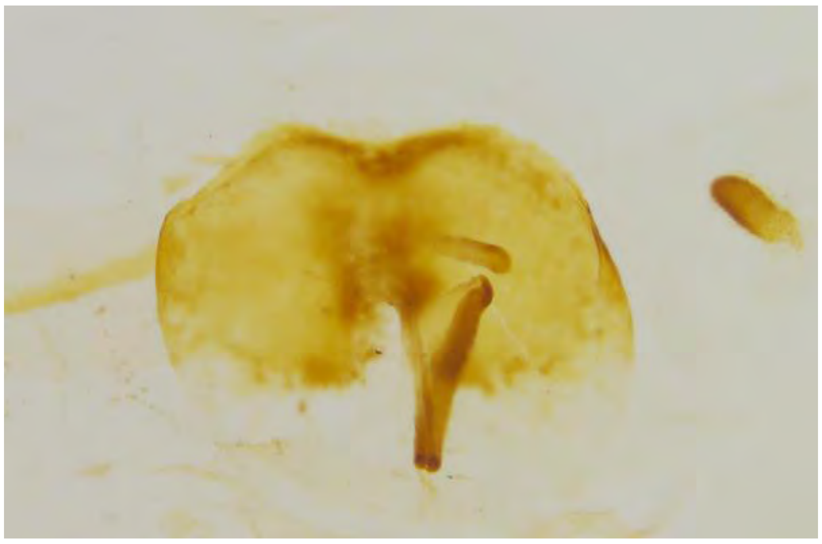

(26.4)

T. delahayei

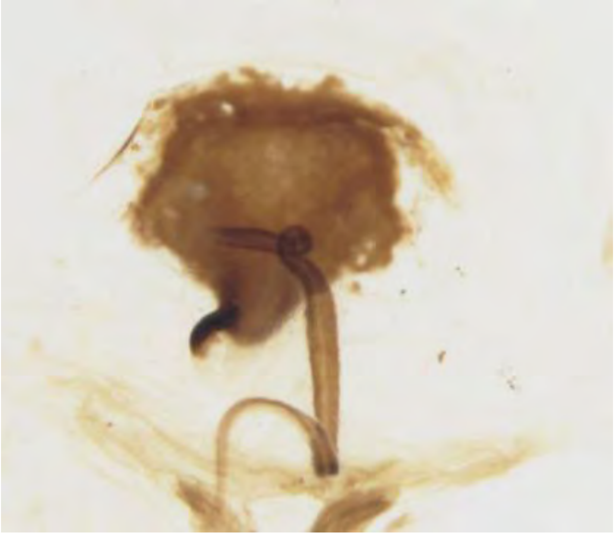

(26.5)

T. macroliberianus

Tiaronthophagus - List of the discrete characters

27. Male genitalia, paramere apices (ventral view): (0) parallel; (1) diverging; (2) converging.

28. Male genitalia, endophallus raspula: (0) present; (1) absent.

29. Male genitalia, if raspula present, then: (0) ovalar-shaped, constituted by a dense tuft of short, thick setae; (1) diffused, not differentiated, scattered setae; (2) constituted by a thick, large fringe of very long setae (Figs. 8-13).

30. Male genitalia, lamella copulatrix: (0) present; (1) absent; (2) extremely reduced (Fig. 9G,H,I).

31. Male genitalia, if LC present: (0) a single piece; (1) two pieces at least.

32. Male genitalia, if LC constituted by a single part then: (0) comma-shaped with expanded apical part; (1) plate-shaped; (2) variously folded; (3) dome-shaped.

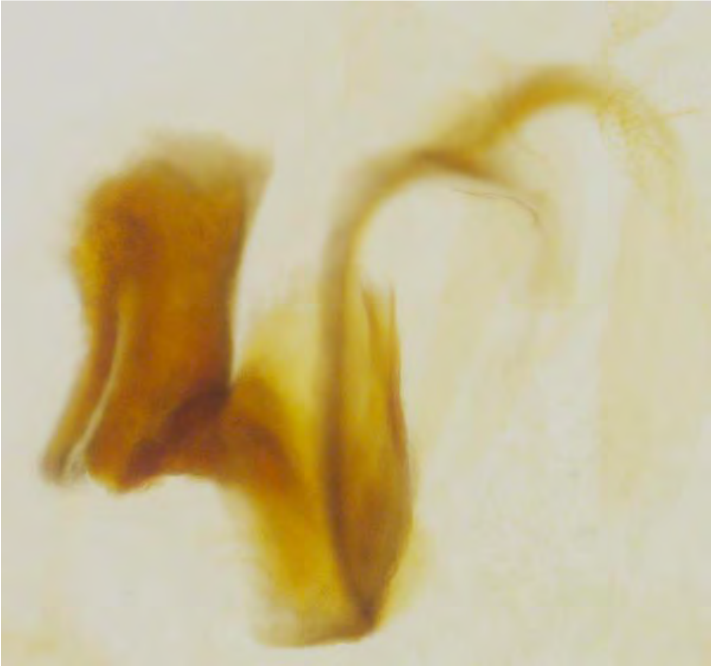

(32.2)

T. pseudoliberianus

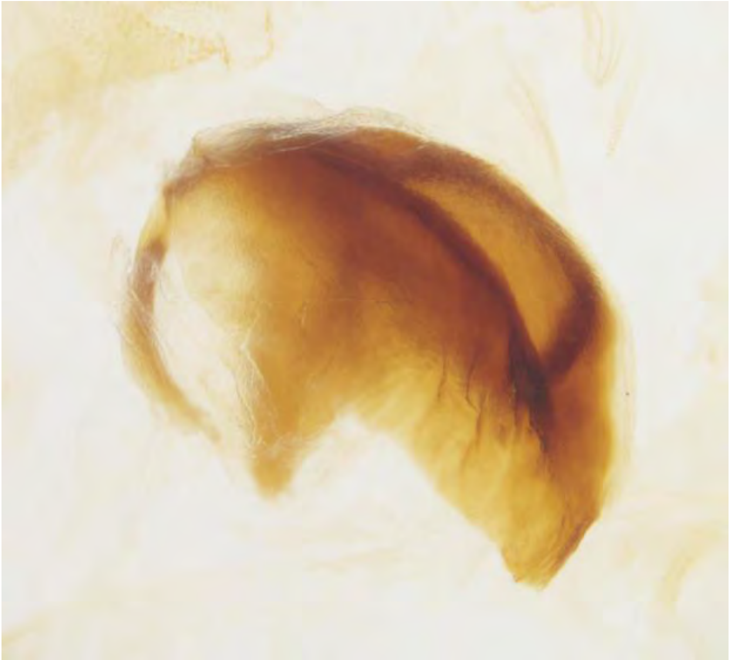

(32.3)

T. saadaniensis

Tiaronthophagus - List of the discrete characters

33. Male genitalia, if lamella copulatrix constituted by two separate parts, then: (0) a flat, rectangular lamina, and a short, folded one; (1) two short, narrow, arched laminae, with the apice pointed; (2) a long, narrow lamina, with the apex expanded and spatulate, and a folded, smaller lamina; (3) a flat lamina angulate, and a smaller, thicker one trangular-shaped; (4) a nastriform, curved lamina, and a tricuspid with a large, flat expansion one; (5) a part large, rounded lamina-shaped, and a small, elongate rod-shaped part.

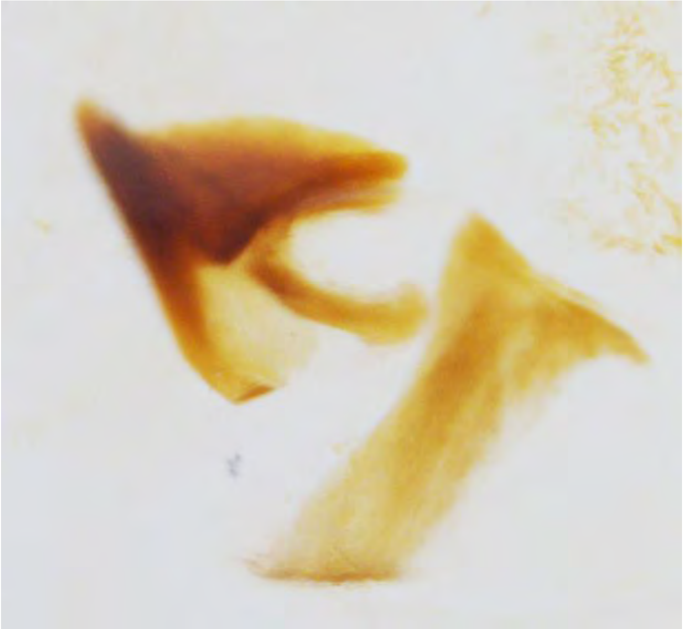

(33.0)

T. aequatus

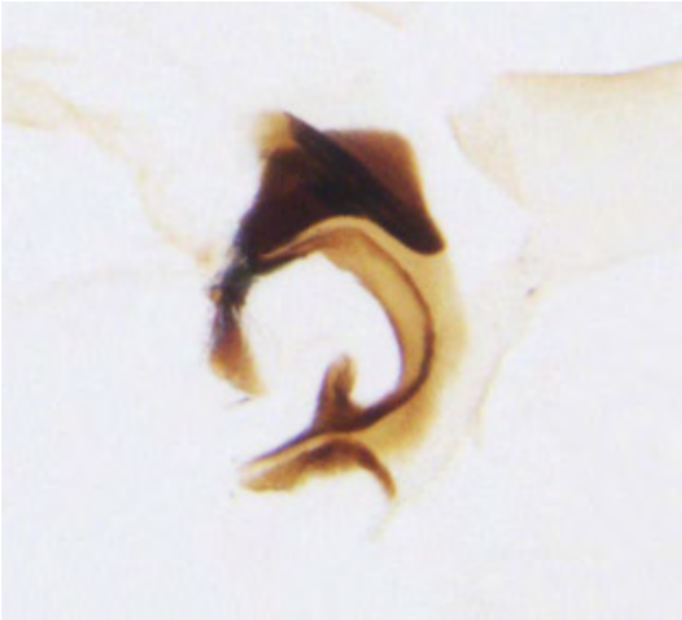

(33.1)

T. schaufussi

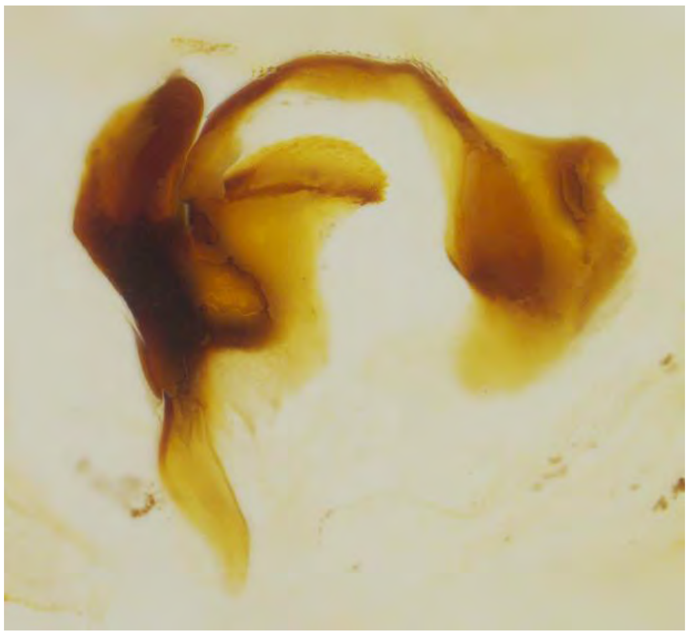

(33.2)

T. delahayei

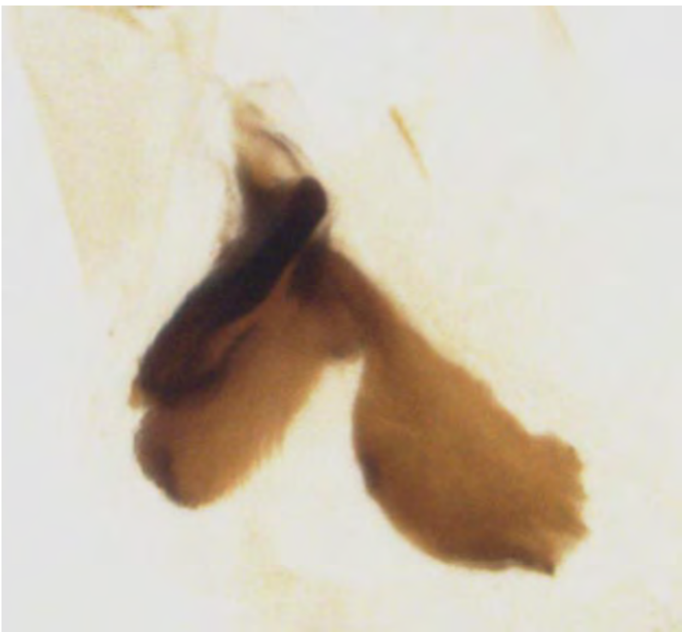

(33.3)

T. ebenus

Tiaronthophagus - List of the discrete characters

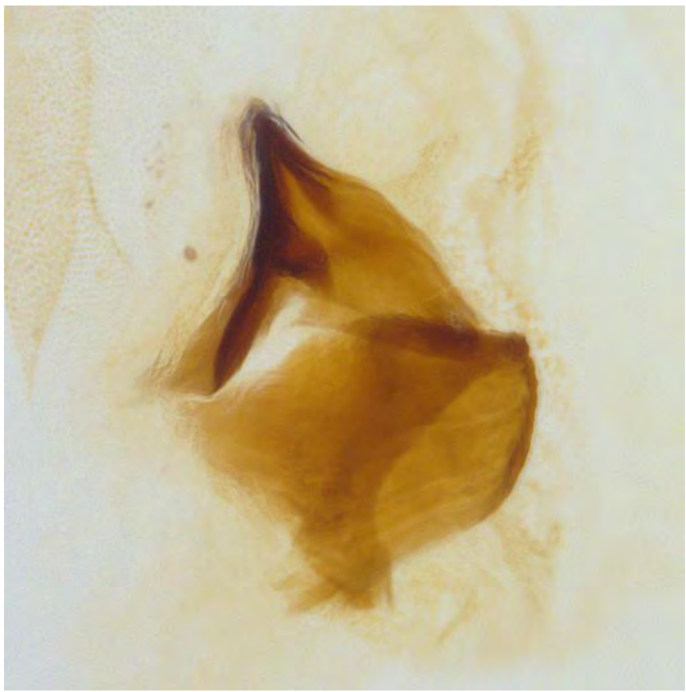

(33.4)

T. katanganus

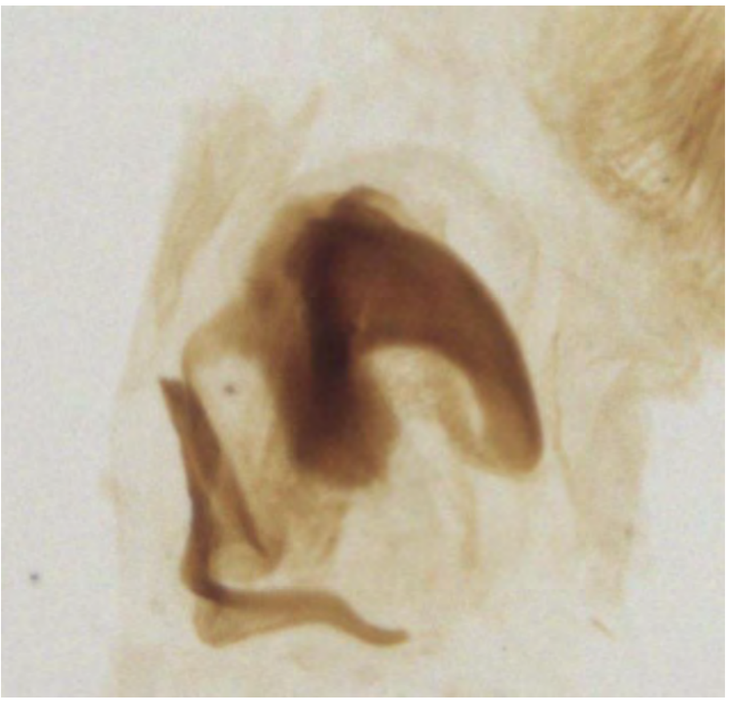

(33.5)

T. rougonorum

34. Male genitalia, secondary lamellae: (0) well developed, constituted by different parts; (1) greatly reduced to a single lamina thick and well-sclerotized.

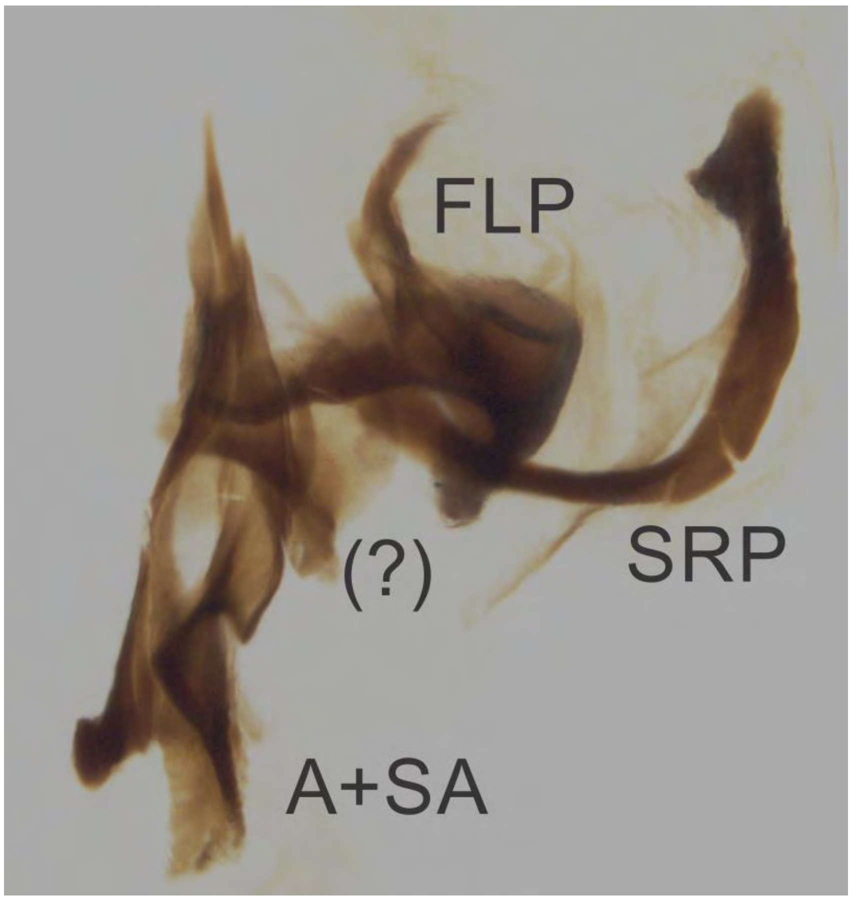

(34.0)

T. ebenus

The following accessory sclerites were identified in Tiaronthophagus: FLP (= fronto-lateral peripheral), SRP (= superior right peripheral), A (= axial), and SA (= subaxial) sclerites, plus an undetermined (?) small sclerite, which could be regarded as BSc (= basal semicircular) sclerite. See Tarasov and Solodovnikov (2011) for further details.

Tiaronthophagus - List of the discrete characters

35. Head, vertex carina in major males (Figs. 6I,J; 8-13)**:** (0) not modified, unarmed head; (1) modified into a long, narrow and curved horn; (2) modified into a squared, large lamina, carrying a pointed, curved petione-shaped extension apically; (3) modified into an extremely long, flat and large lamina; (4) modified into two symmetrical, narrow and arched horns.

36. Head frontal carina in major males: (0) well-defined; (1) more or less evident, sometimes reduced in major males to a thin line barely visible (Fig. 6I,J); (2) inapparent/absent.

37. Head frontal carina in females: (0) absent; (1) small, elevated only in the central part; (2) evident, and raised (Fig. 6G).

38. Head vertex carina in females: (0) large, bisinuate; (1) rectangular lamina quite elevated; (2) narrow, interrupted in the middle, divided in two parts; (3) relatively narrow, rounded flap low-arched, or tricuspid; (4) very large, thick, subtrapezoidal (view from above); (5) narrow, more or less elevated; (6) trapezoidal inverted (frontal view).

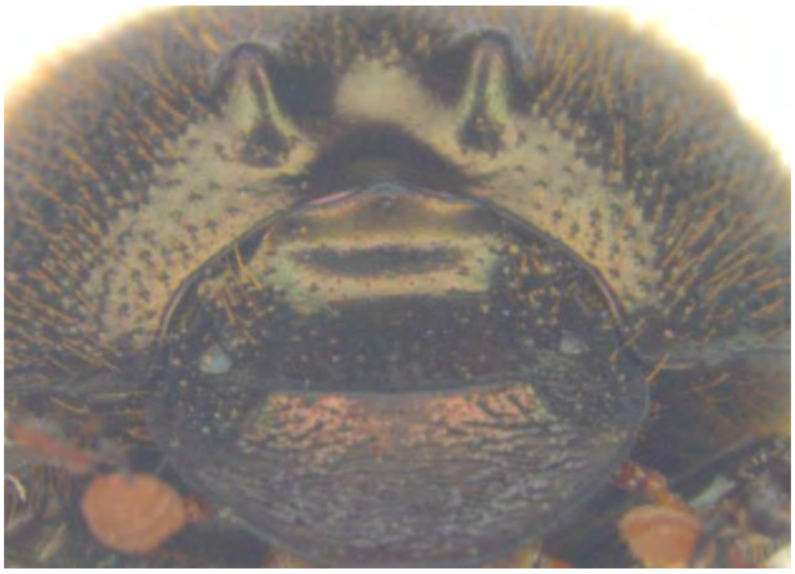

(38.3)

T. naevius

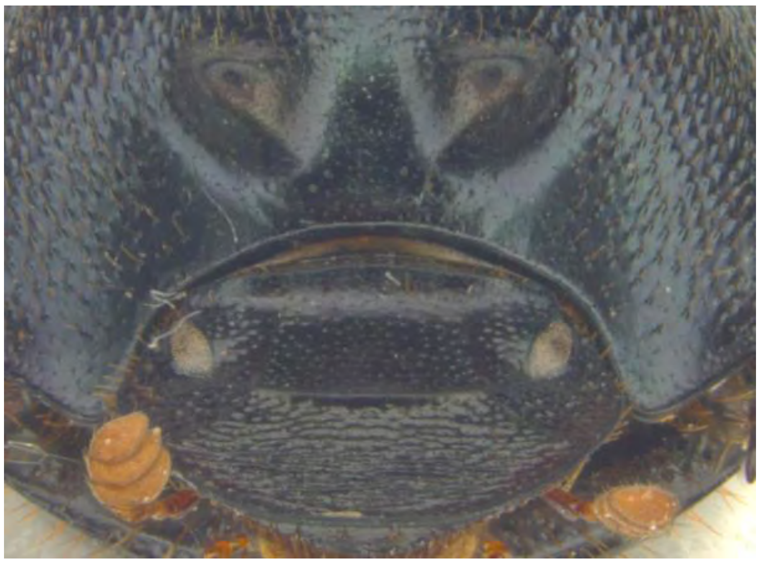

(38.4)

T. rolandoi

Tiaronthophagus - List of the discrete characters

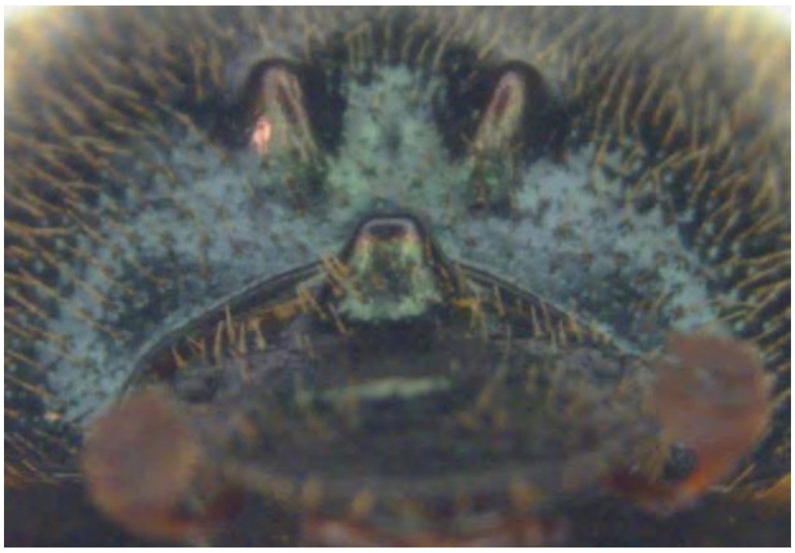

(38.5)

T. lamtoensis

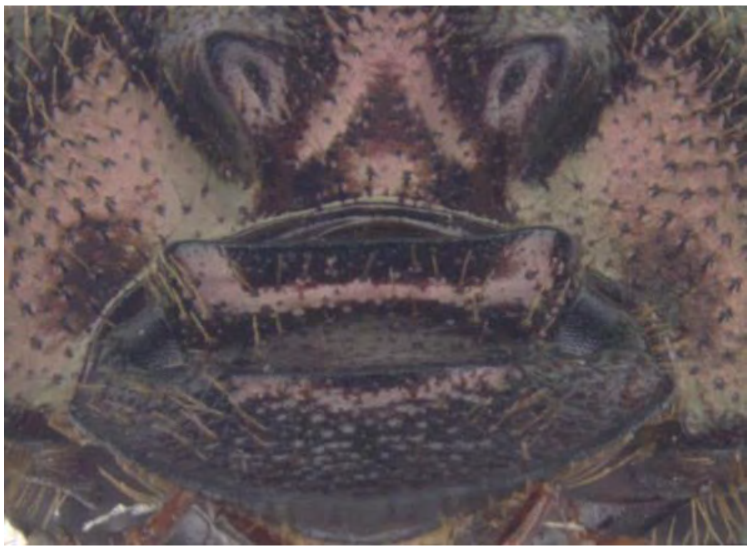

(38.6)

T. pendjarius

39. Head, some large points on the clypeus and genae (Fig. 6C): (0) present, but only on the edge of the head, not on the whole surface; (1) absent; (2) present, scattered on the genae and clypeus surface.

40. Head clypeus: (0) very similar in both sexes, never wholly smooth in males; (1) in males only the anterior part is wrinkled and smooth the remaining 2/3 of the surface, while females clypeus is entirely wrinkled; (2) entirely smooth in males, and wrinkled (with a vermiform microsculture) in females.

41. Pronotum, on sides two symmetrical ovalar areas, glabrous, but carrying a fringe of long yellow setae in superior margin, and smooth (the microsculpture is different from the pronotum other areas). Two corresponding hollow concavities on the elytra near the humeral callous: (0) absent; (1) present (Fig. 6A,D).

42. Pronotum in females, and often in minor males, carrying: (0) no protuberances; (1) a single well-developed tubercle in the middle; (2) two tubercles, variously shaped; (3) two large laminar expansions.

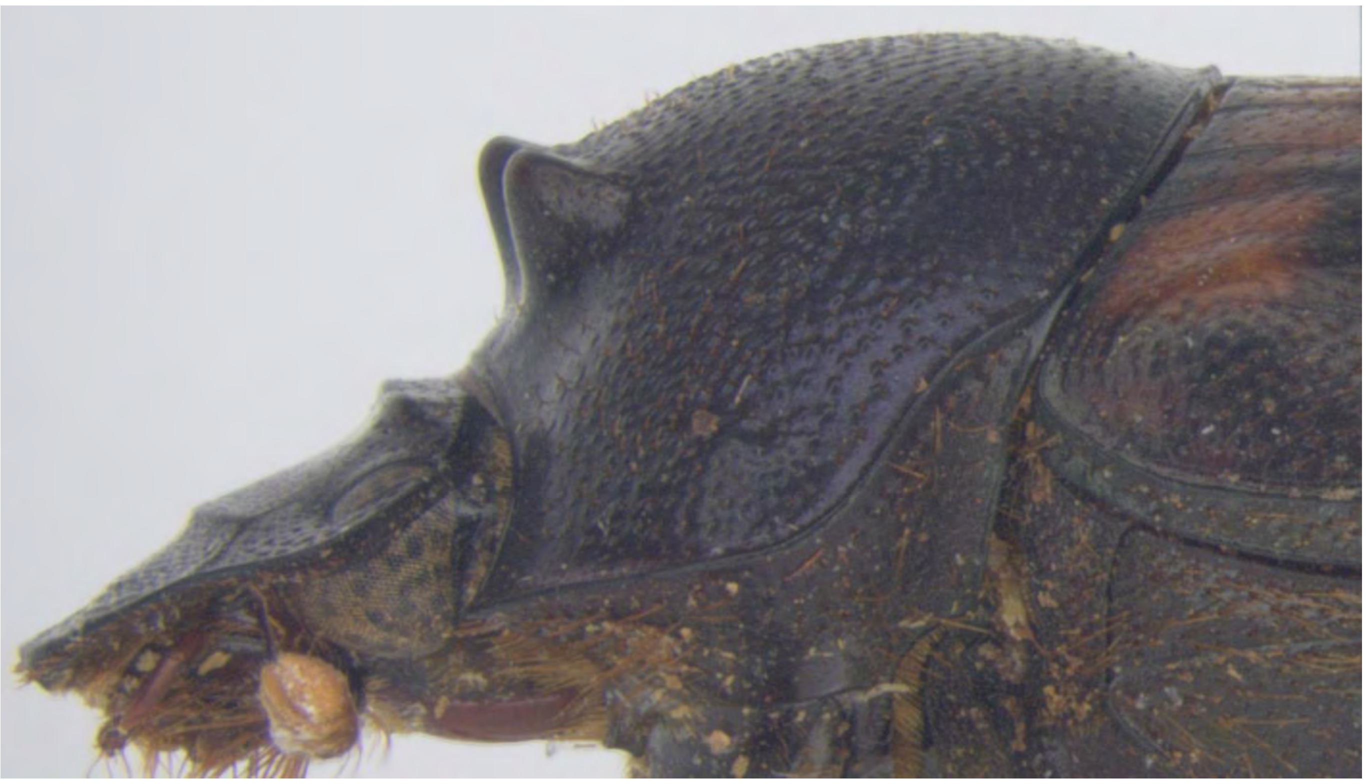

(42.2)

T. zavattarii

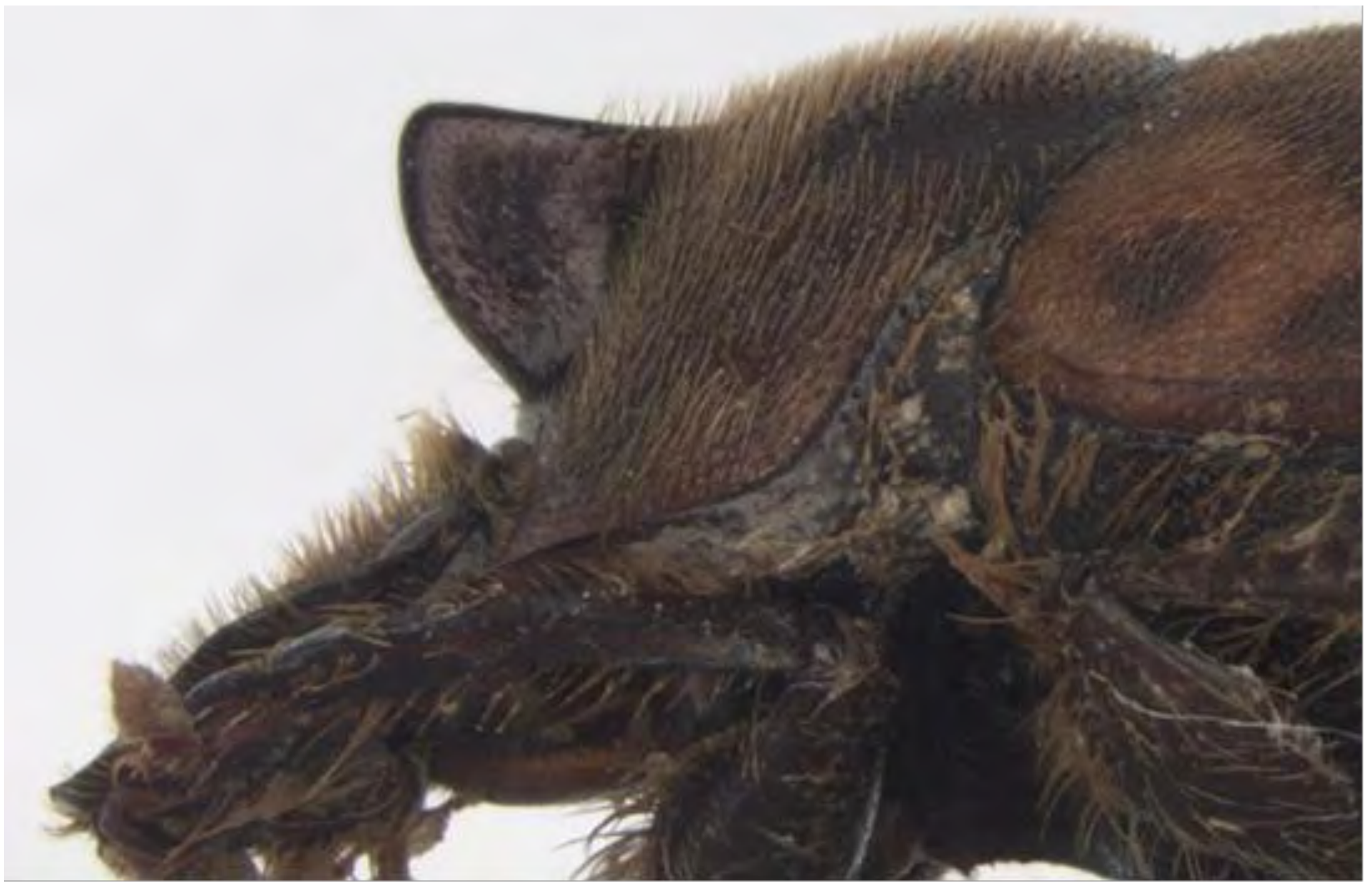

(42.3)

T. jossoi

Tiaronthophagus - List of the discrete characters

43. Female pronotum, tubercles: (0); pointed, parallel apices (1) rounded, parallel apices; (2) pointed, diverging apices; (3) flat-rounded, parallel apices; (4) rounded, converging (almost fuse) apices.

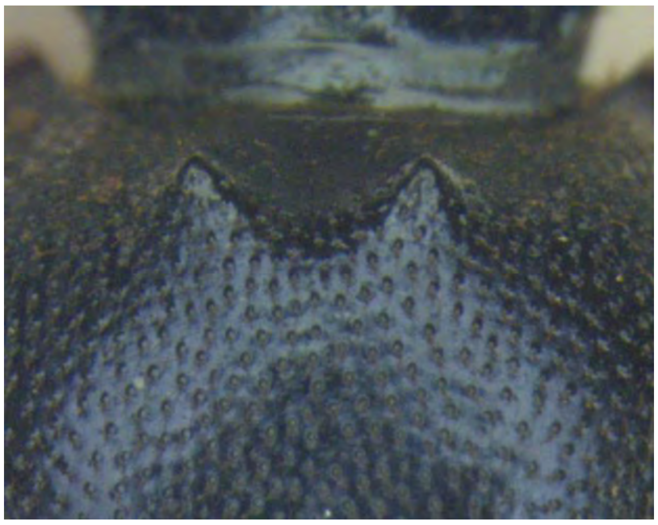

(43.0)

T. aequatus

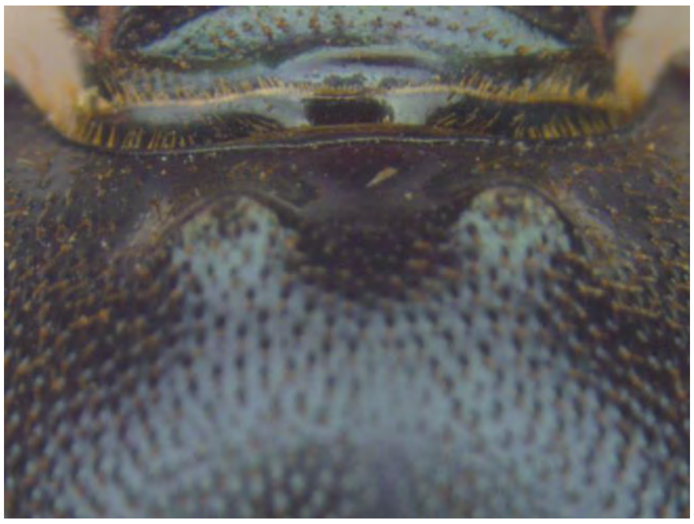

(43.1)

T. rolandoi

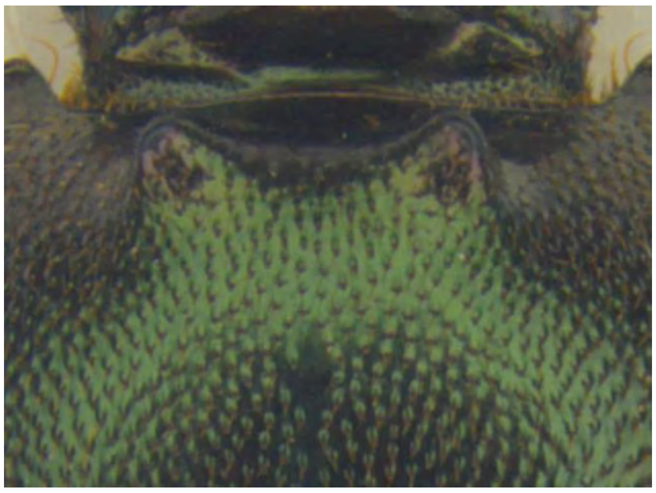

(43.2)

T. ebenus

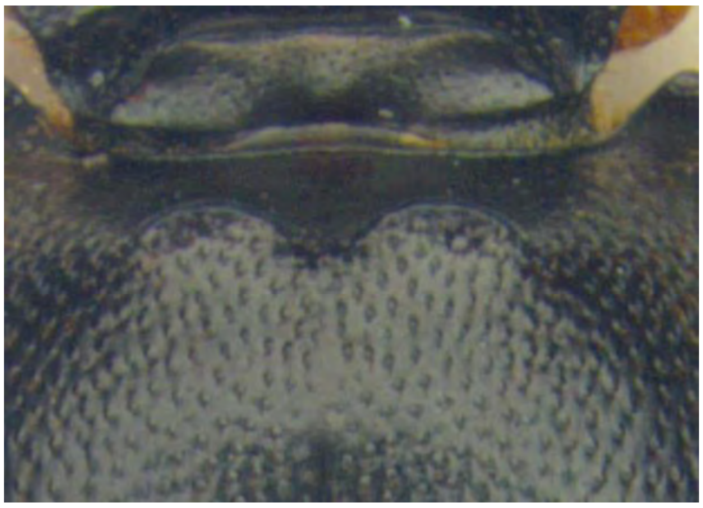

(43.3)

T. katanganus

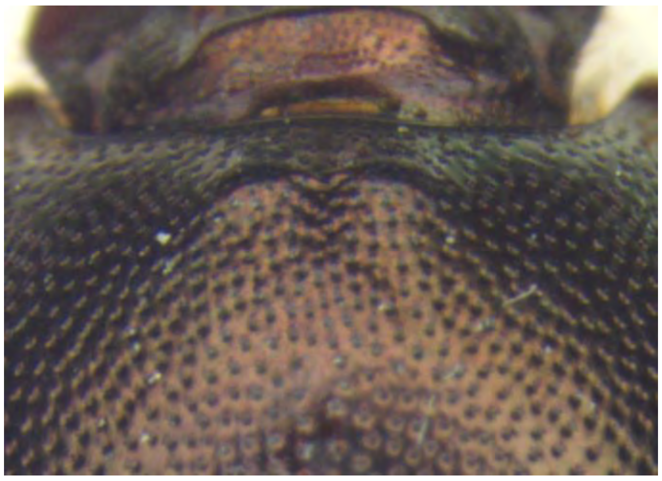

(43.4)

T. rougonorum

Tiaronthophagus - List of the discrete characters

44. Pronotum microsculpture costituted by: (0) simple, evident points, never granulate, sometimes differently sized; (1) large granules, without points and pubescence; (2) granulate points carrying a pubescence.

45. Pronotum pubescence: (0) very reduced; (1) present, thick

46. Pronotum microsculpture: (0) even on the whole surface; (1) smaller and/or scattered in the middle, and tighter on sides, often the granulate points larger and very close.

47. Pronotum points shaped as: (0) simple; (1) ocellate, and granulate; (2) simple (or at least with a little rebord), and granulate.

48. Pronotum, granulate points: (0) quite large, with oval granules beak-shaped; (1) very large with small granules; (2) rounded, with small, rounded granules; (3) ovalar, with ovalar granules.

49. Pronotum, anterior angles: (0) no flat area anteriorly; (1) area quite flattened anteriorly; (2) very small flattened antero-lateral area; (3) small flattened anterior area.

50. Elytra, striae: (0) more or less narrow, not rebordered, simple points; (1) narrow, geminate points, not rebordered; (2) large, geminate points, slightly rebordered.

51. Elytra, interstriae: (0) flat; (1) convex; (2) slightly convex.

Tiaronthophagus - List of the discrete characters

52. Elytra, interstriae: (0) large and deep rough points, arranged on two rows, pubescence constituted by short, dark and (thick) straight setae; (1) simple points, pubescence varying from relatively dense to sparse; (2) large, well-developed and deep granulate points, dense pubescence, with long yellow setae; (3) thick granulate points, the granules relatively small, dense pubescence with yellow recumbent setae; (4) rather deep granulate points, the granules variously developed, arranged on three rows, pubescence rather developed, with yellow long and thin recumbent setae; (5) very thick granules, very dense pubescence, the setae yellow and long; (6) large and dense ocellate points with small granules, relatively dense pubescence with yellow clavate setae.

53. Elytra: (0) with the same microsculpture on all the interstriae; (1) the first two interstriae almost smooth.

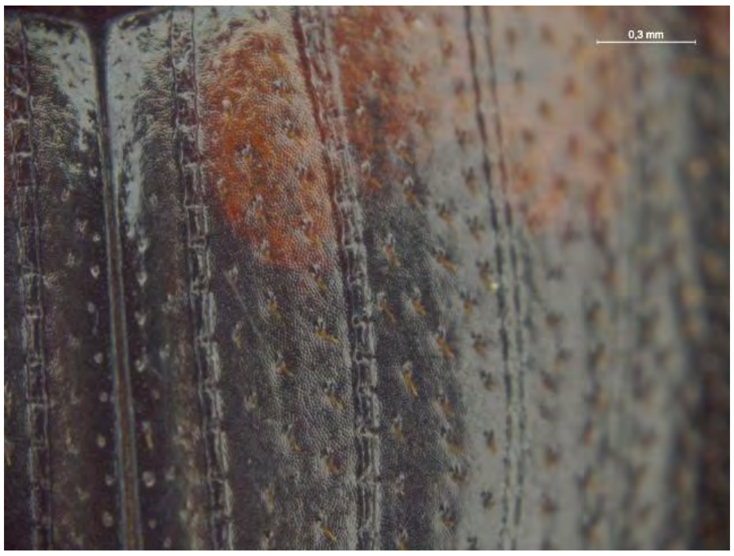

(53.0)

T. rufobasalis

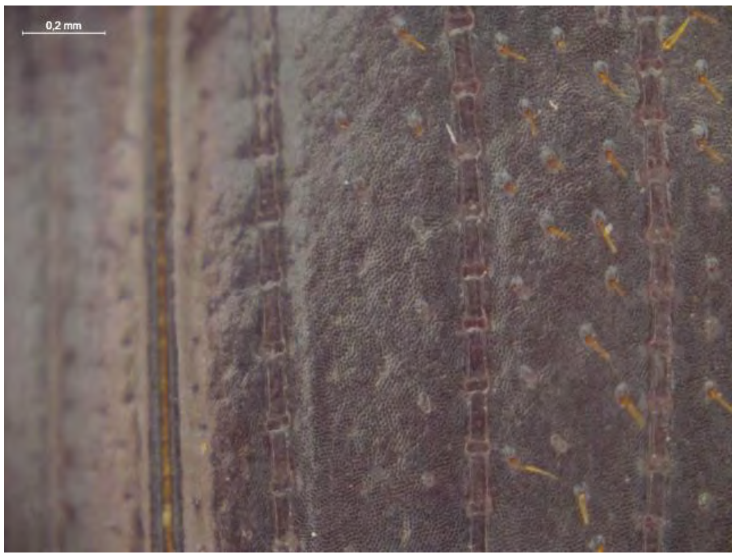

(53.1)

T. rufostillans
