# Supplementary material for: The Phylogenetic Relationships of Tiaronthophagus n.gen. (Coleoptera, Scarabaeidae, Onthophagini) Evaluated by Phenotypic Characters
Source: Insects. 2019 Mar 1;10(3):64. doi: 10.3390/insects10030064 (PMC6468411; doi:10.3390/insects10030064)
Supplement: Supplementary file 1 [file insects-10-00064-s001.zip › Roggero Data S2 Matrix.pdf]

xread
'Matrix of Thiaronthophagus
each point configuration is set as a character
epipharynx - head_F - head_M - right elytron - pronotum_F - pronotum_M - phallobase - paramere - hindwing - eye_F - eye_M - carinae_F'

65 36

&[landmark 2D]
E_flavimargo 0.00426,0.19967 0.22118,0.14365 0.24524,-0.05470 -0.03343,-0.25577 -0.00012,-0.13031 0.11648,-0.05985 -0.00084,-0.06665 0.00575,0.09485 [illegible]
M_pallens -0.00089,0.15244 0.21780,0.15038 0.26029,-0.04198 -0.01748,-0.26787 -0.00019,-0.12158 0.12288,-0.04476 -0.00281,-0.04697 -0.00424,0.06521 [illegible]
H_bituberculatus 0.00408,0.16038 0.19533,0.17633 0.25992,-0.02738 -0.03121,-0.25639 0.00018,-0.12153 0.11921,-0.04889 0.00204,-0.05000 0.00463,0.05121 [illegible]
H_depressus 0.00498,0.15973 0.20464,0.18011 0.26019,-0.04207 -0.04104,-0.26350 0.00094,-0.11701 0.13340,-0.05770 0.00038,-0.05798 0.00273,0.04201 [illegible]
P_nuchicornis 0.00345,0.18696 0.19453,0.17377 0.25167,-0.05746 -0.02592,-0.25297 0.00313,-0.11065 0.12910,-0.04982 0.00168,-0.05585 0.00004,0.06662 [illegible]
O_nigriventris 0.01343,0.18272 0.20020,0.19084 0.23314,-0.03823 -0.03887,-0.26273 0.00011,-0.13972 0.10388,-0.05587 0.00244,-0.05435 -0.00400,0.07096 [illegible]
P_coenobita 0.00263,0.19684 0.19983,0.18107 0.24257,-0.06289 -0.03645,-0.27948 -0.00644,-0.12400 0.12562,-0.06472 -0.00172,-0.06362 -0.00108,0.04354 [illegible]
P_ovatus 0.00438,0.17621 0.20163,0.16649 0.24130,-0.05402 -0.02970,-0.26127 -0.00428,-0.11400 0.13131,-0.05585 0.00012,-0.04877 0.00647,0.08929 [illegible]
P_medius -0.00460,0.18987 0.23284,0.15987 0.23652,-0.05006 -0.03578,-0.26247 0.00116,-0.12617 0.12115,-0.05526 0.00004,-0.05722 0.00083,0.05893 [illegible]
O_illyricus -0.00173,0.16810 0.20096,0.18269 0.26729,-0.05047 -0.04138,-0.27123 0.00256,-0.11292 0.11676,-0.06744 -0.00081,-0.06888 -0.00191,0.08281 [illegible]
T_aequatus 0.00219,0.18200 0.18924,0.18831 0.21906,-0.05241 -0.01785,-0.26826 -0.00210,-0.13667 0.11562,-0.06766 0.00050,-0.06588 -0.00077,0.11567 [illegible]
T_angolensis 0.00130,0.17022 0.19120,0.19477 0.24739,-0.03826 -0.01999,-0.25854 -0.00155,-0.12493 0.12172,-0.05773 0.00015,-0.05360 0.00263,0.06788 [illegible]
T_chrysoderus [illegible]
T_curtipilis [illegible]
T_delahayei [illegible]
T_ebenus [illegible]
T_flexicornis [illegible]
T_hemichlorus [illegible]
T_jossoi [illegible]
T_katangensis [illegible]
T_lamtoensis [illegible]
T_liberianus [illegible]
T_macroliberianus [illegible]
T_naevius [illegible]
T_pendjarius [illegible]
T_pseudoliberianus [illegible]
T_rolandoi [illegible]
T_rougeonum [illegible]
T_rufobasalis [illegible]
T_rufopygus [illegible]
T_rufostillans [illegible]
T_saadaniensis [illegible]
T_schaufussi [illegible]
T_viridiaereus [illegible]
T_zambesianus [illegible]
T_zavattarii [illegible]

&[landmark 2D]
[illegible]

&[landmark 2D]
[illegible]

&[landmark 2D]
[illegible]

T_delahayei -0.11093,0.15343 -0.05387,0.16656 0.03672,0.19658 0.09202,0.23222 0.12792,0.26559 0.15061,0.20852 0.15528,0.15606 0.15738,0.10222 0.15505,0.04821 0.14421,-0.00614 0.12818,-0.05904 0.10852,-0.11087 0.08122,-0.15991 0.03882,-0.20337 -0.02806,-0.23122 -0.08676,-0.24628 -0.14358,-0.24996 -0.14036,-0.19949 -0.13976,-0.14770 -0.13877,-0.09445 -0.13833,-0.03992 -0.13624,0.01626 -0.13239,0.07250 -0.12688,0.13021
T_ebenus -0.12294,0.13907 -0.06860,0.14927 0.02457,0.18182 0.10029,0.23494 0.13167,0.27035 0.14913,0.20856 0.16035,0.15268 0.16393,0.09653 0.15938,0.04118 0.15103,-0.01402 0.13419,-0.06583 0.11148,-0.11534 0.08468,-0.16299 0.04070,-0.20070 -0.01642,-0.22970 -0.08694,-0.24659 -0.13410,-0.24609 -0.13852,-0.19258 -0.13989,-0.13726 -0.14199,-0.08121 -0.13993,-0.02517 -0.14102,0.03051 -0.13977,0.08297 -0.14127,0.12962
T_flexicornis -0.12913,0.15433 -0.06001,0.16468 0.03294,0.19631 0.09637,0.23892 0.12234,0.27177 0.14242,0.20704 0.15262,0.14930 0.15703,0.09111 0.15312,0.03379 0.14672,-0.02293 0.12770,-0.07496 0.11006,-0.12627 0.08363,-0.17132 0.04098,-0.20412 -0.01212,-0.22419 -0.08242,-0.24382 -0.12858,-0.24730 -0.13033,-0.19388 -0.13598,-0.13957 -0.13764,-0.08420 -0.13744,-0.02792 -0.13771,0.02933 -0.13760,0.08522 -0.13698,0.13870
T_hemichlorus -0.10978,0.14737 -0.05189,0.16069 0.03835,0.19322 0.10923,0.24335 0.13400,0.27354 0.15368,0.21481 0.16117,0.16016 0.16392,0.10514 0.15762,0.05103 0.14614,-0.00260 0.12446,-0.05269 0.10026,-0.10210 0.07242,-0.15049 0.02881,-0.19352 -0.02869,-0.23124 -0.09180,-0.24128 -0.14399,-0.24459 -0.14562,-0.19884 -0.14410,-0.15219 -0.14103,-0.10359 -0.13912,-0.05287 -0.13589,0.00088 -0.13257,0.05710 -0.12557,0.11871
T_jossoi -0.11463,0.15814 -0.06081,0.16875 0.02811,0.19319 0.09583,0.23328 0.12724,0.26621 0.15110,0.20525 0.15861,0.15040 0.16075,0.09530 0.15767,0.04118 0.15184,-0.01214 0.13147,-0.06108 0.11079,-0.11037 0.08435,-0.15737 0.04873,-0.20079 -0.01063,-0.22652 -0.09003,-0.23887 -0.14591,-0.24117 -0.14564,-0.19325 -0.14552,-0.14461 -0.14124,-0.09469 -0.14158,-0.04275 -0.13988,0.01206 -0.13779,0.06903 -0.13282,0.13081
T_katanganus -0.11607,0.15945 -0.05513,0.16625 0.02589,0.19738 0.08866,0.24046 0.11555,0.27486 0.13950,0.20983 0.14919,0.15082 0.15142,0.09062 0.15130,0.03149 0.14060,-0.02671 0.12654,-0.08208 0.10657,-0.13369 0.07668,-0.17822 0.03279,-0.21098 -0.01412,-0.23423 -0.07266,-0.24744 -0.11886,-0.25218 -0.12562,-0.19611 -0.12938,-0.13870 -0.13256,-0.08034 -0.13346,-0.02126 -0.13508,0.03819 -0.13659,0.09516 -0.13515,0.14745
T_lamtoensis -0.12137,0.13897 -0.05377,0.15878 0.03690,0.19648 0.10031,0.23809 0.13220,0.26599 0.14227,0.20951 0.15081,0.15734 0.15502,0.10300 0.15252,0.04803 0.14170,-0.00768 0.12664,-0.06237 0.11063,-0.11680 0.08223,-0.16613 0.03717,-0.20951 -0.02786,-0.24002 -0.08117,-0.25016 -0.14297,-0.24925 -0.13617,-0.19629 -0.13668,-0.14125 -0.13444,-0.08479 -0.13492,-0.02801 -0.13459,0.02820 -0.13276,0.08066 -0.13169,0.12720
T_liberianus -0.11799,0.14214 -0.06372,0.15570 0.02958,0.18665 0.10198,0.23211 0.12497,0.26069 0.14665,0.20507 0.15653,0.15360 0.15864,0.10087 0.15231,0.04788 0.14097,-0.00635 0.12637,-0.05996 0.10699,-0.11284 0.08275,-0.16461 0.04530,-0.21176 -0.01754,-0.24302 -0.07963,-0.25081 -0.13815,-0.25683 -0.13888,-0.19922 -0.13971,-0.14034 -0.13776,-0.08044 -0.13792,-0.02082 -0.13586,0.03729 -0.13363,0.09027 -0.13223,0.13473
T_macroliberianus -0.12009,0.14918 -0.06828,0.15966 0.02682,0.18940 0.09890,0.23282 0.12226,0.26335 0.14554,0.20369 0.15509,0.14939 0.16090,0.09472 0.15833,0.04065 0.14929,-0.01341 0.13236,-0.06489 0.11186,-0.11529 0.08526,-0.16303 0.04449,-0.20375 -0.01395,-0.23120 -0.08065,-0.25085 -0.14116,-0.24968 -0.14045,-0.19553 -0.13847,-0.13949 -0.14046,-0.08265 -0.13882,-0.02568 -0.13778,0.03123 -0.13580,0.08552 -0.13519,0.13585
T_naevius -0.12193,0.13651 -0.06079,0.15194 0.03918,0.19114 0.10619,0.24563 0.12645,0.27148 0.14345,0.21498 0.15053,0.15872 0.15503,0.10056 0.14876,0.04253 0.14119,-0.01626 0.12802,-0.07190 0.10566,-0.12366 0.07953,-0.17228 0.03857,-0.20920 -0.01552,-0.22983 -0.08154,-0.24545 -0.13616,-0.25104 -0.13551,-0.19558 -0.13566,-0.13886 -0.13692,-0.08146 -0.13516,-0.02406 -0.13550,0.03255 -0.13666,0.08471 -0.13122,0.12882
T_pendjarius -0.12150,0.13732 -0.05750,0.15385 0.03355,0.18383 0.11083,0.23524 0.14158,0.28775 0.16300,0.21768 0.16648,0.15842 0.16742,0.10153 0.15928,0.04660 0.14438,-0.00734 0.12668,-0.05940 0.10597,-0.10902 0.07615,-0.15353 0.03416,-0.19163 -0.01815,-0.21854 -0.08643,-0.23527 -0.14369,-0.23608 -0.14653,-0.19125 -0.14672,-0.14553 -0.14552,-0.09833 -0.14427,-0.04923 -0.14192,0.00275 -0.14051,0.05660 -0.13675,0.11359
T_pseudoliberianus -0.11928,0.15609 -0.06488,0.16891 0.02756,0.19766 0.09345,0.23824 0.11335,0.26953 0.14171,0.20824 0.15164,0.15197 0.15630,0.09475 0.15337,0.03789 0.14517,-0.01905 0.12906,-0.07292 0.10944,-0.12499 0.07926,-0.17175 0.03857,-0.21288 -0.02019,-0.23533 -0.07798,-0.24485 -0.13472,-0.24893 -0.13614,-0.19517 -0.13293,-0.14060 -0.13393,-0.08467 -0.13135,-0.02780 -0.13065,0.02999 -0.12932,0.08610 -0.12750,0.13958
T_rolandoi -0.11264,0.14713 -0.06952,0.15576 0.02689,0.19334 0.09301,0.23864 0.12066,0.27558 0.14163,0.21120 0.15302,0.15387 0.15576,0.09538 0.15005,0.03708 0.13934,-0.02135 0.12607,-0.07721 0.10604,-0.12946 0.07705,-0.17560 0.03550,-0.21040 -0.00882,-0.23322 -0.07615,-0.24942 -0.13078,-0.25523 -0.13105,-0.19733 -0.13317,-0.13817 -0.13277,-0.07835 -0.13326,-0.01858 -0.13352,0.04037 -0.13375,0.09489 -0.12958,0.14109
T_rougonorum -0.11202,0.14982 -0.05236,0.16346 0.03706,0.19917 0.10063,0.24361 0.12376,0.27766 0.13895,0.21420 0.14548,0.15691 0.14878,0.09889 0.14799,0.04153 0.13836,-0.01533 0.12452,-0.06970 0.10111,-0.12020 0.07594,-0.16909 0.03437,-0.20697 -0.01661,-0.23618 -0.08678,-0.25195 -0.13759,-0.25413 -0.13651,-0.20020 -0.13373,-0.14484 -0.13260,-0.08833 -0.12965,-0.03128 -0.12793,0.02634 -0.12713,0.08205 -0.12404,0.13455
T_rufobasalis -0.12522,0.14818 -0.06243,0.16079 0.03184,0.18660 0.10083,0.22889 0.13034,0.25834 0.14676,0.20088 0.15718,0.15002 0.15957,0.09835 0.15733,0.04689 0.14691,-0.00488 0.13422,-0.05616 0.11308,-0.10571 0.08793,-0.15595 0.05024,-0.20456 -0.02229,-0.23578 -0.08879,-0.24691 -0.14593,-0.25199 -0.14074,-0.19847 -0.14091,-0.14331 -0.14020,-0.08678 -0.13967,-0.02982 -0.13867,0.02715 -0.13592,0.08159 -0.13547,0.13266
T_rufopygus -0.11790,0.15515 -0.06563,0.16999 0.02551,0.19395 0.09412,0.22985 0.12800,0.26495 0.14591,0.20174 0.15493,0.14781 0.16079,0.09452 0.15636,0.04181 0.14831,-0.01135 0.13079,-0.06160 0.11277,-0.11203 0.08744,-0.15960 0.05010,-0.20228 -0.00862,-0.22979 -0.08753,-0.24712 -0.14824,-0.24638 -0.14642,-0.19537 -0.14248,-0.14288 -0.14015,-0.08912 -0.13907,-0.03441 -0.13614,0.02155 -0.13183,0.07713 -0.13101,0.13351
T_rufostillans -0.13377,0.15079 -0.07302,0.17096 0.02516,0.20232 0.08861,0.23824 0.12171,0.26128 0.14375,0.20287 0.15571,0.14756 0.16317,0.08968 0.16096,0.03139 0.15123,-0.02728 0.13449,-0.08261 0.11233,-0.13453 0.08101,-0.17921 0.03689,-0.21093 -0.01243,-0.22570 -0.07003,-0.23652 -0.12104,-0.24086 -0.12562,-0.18717 -0.13248,-0.13293 -0.13467,-0.07742 -0.14064,-0.02142 -0.14302,0.03513 -0.14507,0.08883 -0.14322,0.13755
T_saadaniensis -0.11241,0.15210 -0.06010,0.16552 0.02740,0.19711 0.09563,0.23114 0.13048,0.26339 0.15676,0.20695 0.16410,0.15555 0.16705,0.10293 0.15864,0.05006 0.14614,-0.00403 0.12948,-0.05656 0.10596,-0.10646 0.07867,-0.15494 0.04079,-0.19808 -0.01801,-0.22335 -0.08602,-0.24457 -0.15054,-0.24539 -0.14826,-0.19752 -0.14741,-0.14817 -0.14389,-0.09761 -0.14100,-0.04555 -0.13427,0.00856 -0.12925,0.06424 -0.12992,0.12469
T_schaufussi -0.11505,0.14540 -0.06740,0.15999 0.02373,0.19395 0.08915,0.23980 0.11868,0.26961 0.13951,0.20975 0.15067,0.15351 0.15642,0.09540 0.15533,0.03738 0.14951,-0.02055 0.13131,-0.07446 0.11080,-0.12720 0.08252,-0.17468 0.03824,-0.21005 -0.01678,-0.23228 -0.08777,-0.25078 -0.13284,-0.25152 -0.13224,-0.19511 -0.13349,-0.13698 -0.13179,-0.07826 -0.13370,-0.01970 -0.13360,0.03804 -0.13268,0.09149 -0.12853,0.13724
T_viridiaereus -0.12611,0.14812 -0.07541,0.16270 0.01971,0.19740 0.09695,0.24277 0.11957,0.26673 0.14312,0.20685 0.15559,0.14997 0.16340,0.09130 0.16000,0.03293 0.14953,-0.02520 0.12930,-0.07922 0.10856,-0.13168 0.07803,-0.17653 0.03423,-0.20894 -0.01339,-0.22790 -0.07316,-0.24169 -0.12357,-0.24613 -0.12686,-0.19109 -0.13234,-0.13513 -0.13576,-0.07825 -0.13810,-0.02096 -0.13815,0.03624 -0.13909,0.09004 -0.13604,0.13770
T_zambesianus -0.10744,0.16305 -0.06211,0.17230 0.02829,0.19878 0.08904,0.23477 0.11106,0.26931 0.13797,0.20585 0.14735,0.14948 0.14860,0.09194 0.14707,0.03465 0.13844,-0.02265 0.12393,-0.07719 0.10400,-0.12926 0.07788,-0.17689 0.03795,-0.21344 -0.01088,-0.23576 -0.06836,-0.25307 -0.12679,-0.26117 -0.13133,-0.20230 -0.13095,-0.14192 -0.13206,-0.08074 -0.13165,-0.01925 -0.13057,0.04203 -0.13060,0.09987 -0.12885,0.15161
T_zavattarii -0.11889,0.15480 -0.06479,0.16636 0.03487,0.19670 0.09760,0.23276 0.12577,0.26446 0.14873,0.20452 0.15691,0.15134 0.15762,0.09712 0.15312,0.04295 0.14777,-0.01161 0.13394,-0.06318 0.11558,-0.11318 0.09050,-0.16065 0.04786,-0.20186 -0.01975,-0.23080 -0.09097,-0.24032 -0.14234,-0.24171 -0.14468,-0.19291 -0.14286,-0.14315 -0.14187,-0.09174 -0.13811,-0.03894 -0.13710,0.01613 -0.13507,0.07210 -0.13384,0.13083

&[landmark 2D]
E_flavimargo 0.00201,0.12639 0.11623,0.14038 0.16104,0.18811 0.21447,0.13958 0.24135,0.07673 0.24258,0.00531 0.22117,-0.06331 0.18609,-0.11995 0.13591,-0.15778 0.07514,-0.18047 0.00649,-0.19727 -0.06484,-0.18447 -0.12893,-0.16008 -0.18761,-0.12241 -0.22951,-0.06291 -0.24844,0.00978 -0.24444,0.08530 -0.21603,0.14808 -0.16357,0.18881 -0.11912,0.14017
M_pallens -0.00242,0.11833 0.13833,0.12745 0.16885,0.17916 0.21140,0.12605 0.24817,0.06587 0.25650,-0.00136 0.24234,-0.06328 0.20503,-0.10934 0.14749,-0.13787 0.08059,-0.16208 0.00460,-0.17788 -0.07293,-0.16197 -0.14281,-0.14252 -0.20369,-0.10809 -0.24819,-0.05973 -0.25985,0.00484 -0.24833,0.07416 -0.20997,0.13068 -0.17413,0.17324 -0.14099,0.12434
H_bituberculatus 0.00282,0.12029 0.12421,0.13670 0.15685,0.18959 0.19999,0.13861 0.24003,0.07861 0.24748,0.00607 0.23414,-0.06398 0.19905,-0.11845 0.14338,-0.15274 0.07671,-0.17548 0.00056,-0.18934 -0.07539,-0.17962 -0.14073,-0.15479 -0.19973,-0.11953 -0.24089,-0.06649 -0.25185,0.00247 -0.23711,0.07774 -0.19799,0.14265 -0.15488,0.19148 -0.12665,0.13622
H_depressus 0.00717,0.12644 0.11765,0.13090 0.15446,0.17632 0.19199,0.13285 0.23463,0.07855 0.24617,0.00723 0.24043,-0.06285 0.20939,-0.11636 0.15262,-0.14813 0.08593,-0.17561 0.00598,-0.18616 -0.07627,-0.17793 -0.14709,-0.15424 -0.20984,-0.12094 -0.24769,-0.06347 -0.25690,0.00809 -0.23978,0.08251 -0.19556,0.14111 -0.15200,0.18164 -0.12129,0.14005
P_nuchicornis 0.00163,0.13395 0.11150,0.14202 0.15694,0.19379 0.20130,0.14151 0.24030,0.07799 0.25235,0.00574 0.23342,-0.06213 0.18879,-0.11526 0.13331,-0.15766 0.06767,-0.18328 -0.00268,-0.20380 -0.07121,-0.18638 -0.13276,-0.16009 -0.18868,-0.12019 -0.23180,-0.06329 -0.25102,0.00502 -0.23905,0.07817 -0.20184,0.14267 -0.15428,0.19123 -0.11391,0.13999
O_nigriventris 0.00612,0.15113 0.10714,0.14741 0.14430,0.18786 0.18966,0.15237 0.22417,0.09323 0.23589,0.01665 0.22234,-0.06109 0.19095,-0.12670 0.13896,-0.17058 0.07333,-0.19734 -0.00130,-0.21747 -0.07477,-0.20563 -0.13640,-0.17270 -0.19490,-0.13362 -0.22480,-0.06577 -0.23459,0.01474 -0.22917,0.09439 -0.19011,0.15283 -0.14449,0.18998 -0.10233,0.15032
P_coenobita 0.01056,0.13165 0.12060,0.14302 0.16170,0.19026 0.20209,0.14488 0.23626,0.08308 0.25053,0.01182 0.22970,-0.05637 0.18618,-0.11308 0.12994,-0.15670 0.06478,-0.18647 -0.00616,-0.20753 -0.07392,-0.18959 -0.13486,-0.16222 -0.19129,-0.12385 -0.23129,-0.06542 -0.24754,0.00552 -0.23664,0.07992 -0.19971,0.14230 -0.15981,0.18875 -0.11111,0.14004
P_ovatus 0.00449,0.13038 0.11060,0.14213 0.15999,0.18696 0.21415,0.13402 0.24234,0.07003 0.24449,0.00031 0.22493,-0.06501 0.18761,-0.11721 0.13875,-0.15505 0.07886,-0.17655 0.00927,-0.19120 -0.06451,-0.18223 -0.13001,-0.15761 -0.19092,-0.12083 -0.23503,-0.06333 -0.25556,0.00559 -0.24161,0.07976 -0.21150,0.14613 -0.16559,0.19039 -0.12077,0.14332
P_medius 0.00194,0.13211 0.10607,0.14151 0.16751,0.18953 0.20341,0.13959 0.23640,0.07740 0.24271,0.00370 0.22960,-0.06675 0.18933,-0.11950 0.13641,-0.15972 0.07317,-0.18315 0.00446,-0.20225 -0.06468,-0.18393 -0.12835,-0.15855 -0.18693,-0.11994 -0.23035,-0.06136 -0.25195,0.00768 -0.23975,0.08081 -0.20675,0.14446 -0.17156,0.19144 -0.11068,0.14692
O_illyricus -0.00142,0.14439 0.11403,0.14460 0.14177,0.17794 0.18683,0.14574 0.22233,0.08673 0.24340,0.01296 0.23796,-0.05954 0.20122,-0.11807 0.14436,-0.16078 0.07606,-0.19179 0.00202,-0.21804 -0.07285,-0.19521 -0.14156,-0.16676 -0.20096,-0.12413 -0.23883,-0.05972 -0.24727,0.01548 -0.22583,0.09123 -0.18561,0.14968 -0.14025,0.18036 -0.11540,0.14494
T_aequatus 0.00072,0.11583 0.12987,0.13292 0.15335,0.17035 0.19524,0.14390 0.23317,0.09311 0.24676,0.02099 0.23609,-0.05454 0.20313,-0.11813 0.14774,-0.16072 0.07733,-0.18540 -0.00295,-0.19906 -0.07994,-0.18333 -0.14569,-0.16094 -0.19907,-0.11840 -0.23635,-0.05681 -0.25074,0.01369 -0.23664,0.08531 -0.20056,0.14348 -0.14770,0.17774 -0.12377,0.14000
T_angolensis 0.00265,0.11931 0.11481,0.13197 0.15156,0.18048 0.20103,0.14113 0.24241,0.08557 0.25397,0.01507 0.23560,-0.05619 0.19758,-0.11636 0.14156,-0.15828 0.07516,-0.18590 -0.00144,-0.19793 -0.07604,-0.18613 -0.13874,-0.15749 -0.19501,-0.11575 -0.23483,-0.05524 -0.25242,0.01436 -0.24126,0.08430 -0.20145,0.13995 -0.15533,0.17871 -0.11979,0.13843
T_chrysoderus 0.00235,0.11470 0.13149,0.13589 0.15611,0.17668 0.19816,0.14231 0.23469,0.08813 0.24715,0.01757 0.23122,-0.05469 0.19544,-0.11638 0.14195,-0.16037 0.07731,-0.18894 0.00041,-0.19856 -0.07520,-0.18756 -0.13872,-0.16114 -0.19349,-0.11721 -0.23430,-0.05581 -0.25106,0.01537 -0.23996,0.08678 -0.20108,0.14308 -0.15707,0.18153 -0.12540,0.13864
T_curtipilis -0.00021,0.12992 0.13640,0.13666 0.16139,0.17110 0.20714,0.13505 0.23957,0.08005 0.24969,0.01250 0.23142,-0.05512 0.19436,-0.11318 0.14059,-0.15476 0.07567,-0.18249 0.00144,-0.19828 -0.07244,-0.18015 -0.13768,-0.15605 -0.19293,-0.11537 -0.23223,-0.05534 -0.25171,0.01233 -0.24188,0.08147 -0.21066,0.13893 -0.16095,0.17422 -0.13699,0.13852
T_delahayei -0.00384,0.12667 0.11408,0.13607 0.15825,0.17964 0.20057,0.13920 0.23857,0.08205 0.25230,0.01189 0.23538,-0.05760 0.19555,-0.11549 0.14338,-0.15962 0.07842,-0.18426 0.00310,-0.19764 -0.07381,-0.18570 -0.13987,-0.15936 -0.19709,-0.11706 -0.23668,-0.05500 -0.25325,0.01484 -0.23428,0.08522 -0.19594,0.14163 -0.15903,0.17669 -0.12580,0.13784
T_ebenus -0.00153,0.13186 0.12181,0.13533 0.14945,0.16442 0.19452,0.14118 0.23666,0.09175 0.25652,0.02215 0.24520,-0.05077 0.20743,-0.11266 0.14749,-0.15598 0.07404,-0.18440 -0.00873,-0.20111 -0.08665,-0.18496 -0.15213,-0.15916 -0.20631,-0.11763 -0.24110,-0.05726 -0.24894,0.01312 -0.23296,0.08339 -0.19034,0.13592 -0.14530,0.16940 -0.11912,0.13541
T_flexicornis -0.00497,0.12973 0.13435,0.13334 0.16200,0.16764 0.19871,0.13757 0.23270,0.08481 0.24961,0.01676 0.23873,-0.05214 0.20265,-0.10989 0.14667,-0.15129 0.07776,-0.17953 -0.00075,-0.19974 -0.07984,-0.18627 -0.14791,-0.16203 -0.20381,-0.11825 -0.24230,-0.05646 -0.25141,0.01487 -0.22908,0.08662 -0.19508,0.14266 -0.15536,0.16776 -0.13268,0.13384
T_hemichlorus -0.00057,0.13391 0.11637,0.13406 0.16168,0.17240 0.18879,0.13414 0.23477,0.08606 0.25347,0.01799 0.24007,-0.05359 0.20201,-0.11433 0.14561,-0.15828 0.07687,-0.18570 -0.00100,-0.19985 -0.07723,-0.17988 -0.14535,-0.15983 -0.20119,-0.11983 -0.23856,-0.05778 -0.25462,0.01306 -0.23825,0.08383 -0.19576,0.13909 -0.15585,0.17742 -0.11128,0.13711
T_jossoi -0.00417,0.11788 0.12103,0.13378 0.15156,0.17198 0.19137,0.13911 0.23432,0.08870 0.25074,0.01904 0.23810,-0.05383 0.20301,-0.11611 0.14895,-0.16006 0.07928,-0.18462 0.00373,-0.20763 -0.07267,-0.18491 -0.14301,-0.15846 -0.20308,-0.11627 -0.24190,-0.05232 -0.25459,0.02046 -0.23509,0.09146 -0.19288,0.14356 -0.15193,0.17196 -0.12277,0.13630
T_katanganus 0.00236,0.11928 0.12173,0.13423 0.15616,0.17847 0.19378,0.14650 0.23541,0.09448 0.25044,0.02210 0.23281,-0.05404 0.19817,-0.12022 0.14117,-0.16353 0.07167,-0.19012 -0.00699,-0.20172 -0.08024,-0.18679 -0.14222,-0.16414 -0.19292,-0.12181 -0.22771,-0.05751 -0.24758,0.01456 -0.23896,0.08662 -0.20091,0.14437 -0.15251,0.18430 -0.11366,0.13497
T_lamtoensis 0.00170,0.12853 0.12487,0.13129 0.15760,0.16760 0.19733,0.13377 0.23850,0.08261 0.25511,0.01510 0.24340,-0.05333 0.20436,-0.10933 0.14618,-0.15046 0.07740,-0.18087 -0.00094,-0.19877 -0.07826,-0.18128 -0.14582,-0.15593 -0.20280,-0.11479 -0.24024,-0.05290 -0.25665,0.01512 -0.23642,0.08264 -0.19790,0.13673 -0.15893,0.16840 -0.12851,0.13587
T_liberianus -0.00147,0.13157 0.11390,0.13523 0.15545,0.17460 0.19854,0.14354 0.23336,0.08687 0.25192,0.01626 0.23440,-0.05528 0.19998,-0.11814 0.14544,-0.16046 0.07757,-0.18585 -0.00094,-0.19949 -0.07885,-0.18801 -0.14457,-0.16086 -0.20171,-0.12224 -0.23426,-0.05765 -0.24925,0.01447 -0.23010,0.08597 -0.19404,0.14303 -0.15496,0.17514 -0.12039,0.14130
T_macroliberianus 0.00112,0.12259 0.11266,0.13393 0.14592,0.17077 0.19132,0.13840 0.23337,0.08414 0.25508,0.01510 0.24396,-0.05522 0.20911,-0.11484 0.15308,-0.15648 0.08452,-0.18417 0.00306,-0.19477 -0.07920,-0.18218 -0.14976,-0.15939 -0.20781,-0.11694 -0.24445,-0.05177 -0.25927,0.01948 -0.23568,0.08848 -0.19178,0.14068 -0.14912,0.16811 -0.11613,0.13408
T_naevius 0.00309,0.13350 0.11288,0.13678 0.15057,0.17045 0.18424,0.14079 0.22696,0.08844 0.25222,0.01763 0.24399,-0.05560 0.20604,-0.11739 0.15116,-0.16389 0.07984,-0.18810 -0.00026,-0.20497 -0.07773,-0.18470 -0.14625,-0.16112 -0.20211,-0.11663 -0.24047,-0.05243 -0.25480,0.01884 -0.23281,0.08837 -0.19013,0.14086 -0.15462,0.17233 -0.11182,0.13682
T_pendjarius -0.00302,0.12458 0.12122,0.13351 0.14922,0.18193 0.19789,0.13939 0.23905,0.08268 0.24975,0.01189 0.23245,-0.05898 0.19562,-0.11843 0.14440,-0.16151 0.07994,-0.18513 0.00466,-0.19687 -0.07196,-0.18573 -0.13724,-0.16007 -0.19307,-0.11567 -0.23668,-0.05584 -0.25290,0.01520 -0.24328,0.08683 -0.20083,0.14272 -0.14897,0.18187 -0.12624,0.13763
T_pseudoliberianus -0.00468,0.13186 0.12781,0.13598 0.15163,0.16459 0.19108,0.14015 0.22768,0.08587 0.25494,0.01795 0.24504,-0.05260 0.20985,-0.11410 0.15256,-0.15768 0.08123,-0.18615 -0.00217,-0.19818 -0.08282,-0.18351 -0.15004,-0.15536 -0.20666,-0.11235 -0.24426,-0.05160 -0.25725,0.01456 -0.23015,0.08130 -0.19229,0.13730 -0.14983,0.16510 -0.12168,0.13687
T_rolandoi 0.00287,0.12908 0.12221,0.12797 0.15158,0.16419 0.19010,0.13268 0.23122,0.07939 0.25557,0.01164 0.24737,-0.05684 0.20997,-0.11430 0.15645,-0.15735 0.08671,-0.18073 0.00572,-0.19280 -0.07490,-0.17420 -0.14541,-0.14978 -0.20472,-0.10929 -0.24441,-0.04893 -0.26245,0.01685 -0.24315,0.08202 -0.19960,0.13463 -0.15530,0.17024 -0.12983,0.13552
T_rougonorum -0.00391,0.13567 0.12930,0.14286 0.15466,0.18472 0.19937,0.14616 0.23388,0.08752 0.24393,0.01342 0.22923,-0.06074 0.19319,-0.12190 0.13808,-0.16363 0.07256,-0.19114 -0.00346,-0.20194 -0.07686,-0.18839 -0.13799,-0.16133 -0.19171,-0.12236 -0.22663,-0.06170 -0.24225,0.00903 -0.23357,0.08202 -0.19970,0.14298 -0.15024,0.18478 -0.12791,0.14397
T_rufobasalis -0.00063,0.13783 0.11749,0.13521 0.14623,0.17529 0.19264,0.14194 0.23366,0.08665 0.25321,0.01707 0.23574,-0.05466 0.20116,-0.11786 0.14674,-0.16120 0.07757,-0.18643 0.00001,-0.20494 -0.07714,-0.18651 -0.14497,-0.16102 -0.20204,-0.11979 -0.23850,-0.05638 -0.25350,0.01448 -0.23278,0.08536 -0.19246,0.14289 -0.14225,0.17442 -0.12019,0.13765
T_rufopygus -0.00255,0.11811 0.13139,0.13964 0.16201,0.16954 0.19722,0.14284 0.23197,0.09050 0.25008,0.02040 0.23647,-0.05276 0.20189,-0.11576 0.14440,-0.15792 0.07290,-0.18453 -0.00568,-0.20446 -0.07987,-0.18727 -0.14334,-0.15940 -0.19726,-0.11638 -0.23290,-0.05252 -0.25256,0.01569 -0.23493,0.08282 -0.19541,0.13771 -0.15784,0.17629 -0.12598,0.13745
T_rufostillans 0.00276,0.12032 0.11667,0.13561 0.15298,0.17237 0.19737,0.14004 0.23548,0.08542 0.25341,0.01685 0.23595,-0.05340 0.20129,-0.11523 0.14680,-0.15783 0.07961,-0.18523 0.00236,-0.20148 -0.07546,-0.18663 -0.14262,-0.15976 -0.19938,-0.11466 -0.23979,-0.05174 -0.25508,0.01930 -0.23920,0.08916 -0.19885,0.14183 -0.15107,0.17068 -0.12322,0.13434
T_saadaniensis -0.00211,0.12008 0.11018,0.13750 0.14193,0.17703 0.18627,0.14846 0.23121,0.09658 0.25298,0.02660 0.23218,-0.04913 0.20295,-0.11870 0.14407,-0.16216 0.07591,-0.19734 -0.00086,-0.21934 -0.07744,-0.19805 -0.14500,-0.16521 -0.20503,-0.12251 -0.23548,-0.05202 -0.25193,0.02389 -0.22927,0.09541 -0.18353,0.14796 -0.13735,0.17514 -0.10970,0.13581
T_schaufussi 0.00166,0.12199 0.11960,0.14004 0.15202,0.17496 0.19146,0.13943 0.23172,0.08641 0.24595,0.01537 0.23510,-0.05734 0.19968,-0.11810 0.14906,-0.16280 0.08248,-0.18667 0.00591,-0.20388 -0.07212,-0.18902 -0.13972,-0.15887 -0.19978,-0.11495 -0.24141,-0.05263 -0.25417,0.01949 -0.23652,0.09046 -0.19320,0.14263 -0.15457,0.17570 -0.12313,0.13779
T_viridiaereus 0.00283,0.11145 0.12629,0.13593 0.15511,0.17465 0.19224,0.13875 0.23585,0.09006 0.24946,0.02266 0.23036,-0.04979 0.19776,-0.11559 0.14506,-0.16119 0.07854,-0.18971 0.00186,-0.20692 -0.07437,-0.19231 -0.13940,-0.16110 -0.19687,-0.11526 -0.23696,-0.05058 -0.25250,0.02247 -0.24096,0.09127 -0.19406,0.13997 -0.15460,0.17755 -0.12564,0.13769
T_zambesianus -0.00075,0.12872 0.12019,0.13003 0.15655,0.17254 0.19501,0.13332 0.23618,0.08100 0.24951,0.01291 0.23574,-0.05606 0.19857,-0.11348 0.14771,-0.15701 0.08215,-0.18041 0.00587,-0.19540 -0.07266,-0.18383 -0.14087,-0.15862 -0.20089,-0.12040 -0.23881,-0.05799 -0.25549,0.01414 -0.24364,0.08594 -0.19974,0.14142 -0.15227,0.18132 -0.12237,0.14184
T_zavattarii 0.00127,0.12754 0.11590,0.13429 0.14719,0.17175 0.19486,0.14307 0.23760,0.09208 0.25365,0.02130 0.24087,-0.05245 0.20272,-0.11478 0.14465,-0.15944 0.07402,-0.18861 -0.00523,-0.20629 -0.08088,-0.18812 -0.14580,-0.15979 -0.20126,-0.11657 -0.23921,-0.05549 -0.25006,0.01528 -0.23345,0.08613 -0.19255,0.14009 -0.14758,0.17237 -0.11670,0.13763

&[landmark 2D]
E_flavimargo 0.00072,0.13358 0.10676,0.14261 0.15557,0.19061 0.21420,0.14195 0.23973,0.07794 0.24653,0.00735 0.22684,-0.05967 0.19087,-0.11576 0.13887,-0.15411 0.07643,-0.17993 0.00526,-0.19544 -0.06892,-0.17993 -0.13452,-0.15843 -0.19398,-0.13123 -0.22835,-0.07160 -0.24876,0.00151 -0.24127,0.07749 -0.21302,0.14286 -0.16320,0.18762 -0.10975,0.14256
M_pallens 0.00331,0.12356 0.12400,0.12968 0.16507,0.17802 0.19563,0.12588 0.23657,0.07094 0.24847,0.00296 0.24191,-0.06284 0.20755,-0.11360 0.15605,-0.15108 0.09253,-0.17554 0.01602,-0.17904 -0.06549,-0.16805 -0.13505,-0.14347 -0.19770,-0.10858 -0.24339,-0.05646 -0.26122,0.00908 -0.24799,0.07625 -0.21072,0.13229 -0.18370,0.17869 -0.14186,0.13130
H_bituberculatus -0.00102,0.11402 0.12224,0.13261 0.14889,0.18781 0.18539,0.14092 0.23028,0.08653 0.24611,0.01381 0.24307,-0.05784 0.21110,-0.11461 0.15507,-0.15240 0.08436,-0.17808 0.00295,-0.19295 -0.07811,-0.18224 -0.14629,-0.15908 -0.20505,-0.12123 -0.24501,-0.06387 -0.25378,0.00902 -0.23635,0.08241 -0.19024,0.13867 -0.15575,0.18456 -0.11785,0.13193
H_depressus -0.00503,0.12043 0.11838,0.13680 0.16143,0.18079 0.19571,0.13715 0.23778,0.08388 0.25107,0.01384 0.24194,-0.05702 0.20703,-0.11369 0.15046,-0.15117 0.07993,-0.17410 -0.00017,-0.18608 -0.07988,-0.17820 -0.14521,-0.15583 -0.20112,-0.11861 -0.24268,-0.06867 -0.24930,-0.00002 -0.23766,0.07404 -0.20061,0.13736 -0.15444,0.18632 -0.12763,0.13277
P_nuchicornis ?
O_nigriventris -0.00186,0.14750 0.08738,0.15422 0.13102,0.19384 0.19054,0.15518 0.22291,0.09263 0.23465,0.01556 0.22447,-0.06128 0.19345,-0.12563 0.14084,-0.16922 0.07833,-0.20168 0.00626,-0.22023 -0.06915,-0.20280 -0.13459,-0.17961 -0.18844,-0.13566 -0.22587,-0.06827 -0.24120,0.01160 -0.22918,0.09178 -0.19680,0.15738 -0.12904,0.19290 -0.09371,0.15177
P_coenobita -0.00247,0.13435 0.10678,0.14406 0.15139,0.18283 0.19058,0.14236 0.22911,0.08571 0.24677,0.01513 0.23474,-0.05623 0.20093,-0.11674 0.14486,-0.15700 0.07723,-0.18687 0.00493,-0.21160 -0.06945,-0.18804 -0.13688,-0.16184 -0.19663,-0.12477 -0.23535,-0.06337 -0.25181,0.00859 -0.23309,0.08138 -0.19721,0.14337 -0.15144,0.18363 -0.11299,0.14505
P_ovatus -0.00079,0.13438 0.11136,0.14190 0.15810,0.19074 0.21219,0.13960 0.24499,0.07870 0.24747,0.00803 0.23350,-0.05974 0.19010,-0.11037 0.13391,-0.15050 0.06790,-0.17807 -0.00281,-0.19871 -0.07152,-0.18108 -0.13299,-0.16008 -0.18823,-0.12566 -0.23063,-0.07478 -0.24251,-0.00512 -0.23972,0.07096 -0.22117,0.14035 -0.16525,0.19304 -0.10388,0.14641
P_medius -0.00480,0.12233 0.10975,0.13633 0.13772,0.18942 0.19556,0.14945 0.23177,0.08832 0.24806,0.01530 0.23607,-0.05652 0.20091,-0.11617 0.14425,-0.15687 0.07734,-0.18871 0.00403,-0.21141 -0.07042,-0.19084 -0.13612,-0.16144 -0.19597,-0.12252 -0.23575,-0.06085 -0.25306,0.01144 -0.23418,0.08456 -0.19852,0.14688 -0.14568,0.18352 -0.11097,0.13777
O_illyricus -0.00724,0.15342 0.10041,0.14782 0.12838,0.20280 0.18763,0.14914 0.22475,0.08067 0.24089,0.00619 0.22424,-0.06448 0.19024,-0.12373 0.14146,-0.16542 0.08140,-0.19337 0.01069,-0.21011 -0.06566,-0.19908 -0.13150,-0.17459 -0.19029,-0.13507 -0.23212,-0.07337 -0.24613,0.00426 -0.23023,0.08563 -0.18985,0.15571 -0.13198,0.20487 -0.10508,0.14871
T_aequatus -0.00058,0.13121 0.12385,0.13304 0.14744,0.16783 0.19123,0.13596 0.22976,0.08382 0.24942,0.01675 0.24104,-0.05178 0.20803,-0.11073 0.15687,-0.15409 0.08753,-0.17876 0.00716,-0.19669 -0.07493,-0.18456 -0.14370,-0.15766 -0.20355,-0.11829 -0.24192,-0.05611 -0.25935,0.01466 -0.24315,0.08312 -0.20099,0.13686 -0.15099,0.17114 -0.12316,0.13430
T_angolensis 0.00397,0.13047 0.11460,0.13696 0.14022,0.17124 0.18380,0.13885 0.22913,0.08971 0.25029,0.02197 0.24233,-0.04936 0.20819,-0.11143 0.15442,-0.15805 0.08555,-0.18975 0.00492,-0.20613 -0.07620,-0.18818 -0.14500,-0.16098 -0.20318,-0.11831 -0.24158,-0.05440 -0.25751,0.01741 -0.23800,0.08583 -0.19299,0.13823 -0.14613,0.17213 -0.11680,0.13380
T_chrysoderus -0.00069,0.11839 0.11786,0.13770 0.14903,0.17808 0.19430,0.14093 0.23422,0.08758 0.25102,0.02033 0.23458,-0.04927 0.19997,-0.11185 0.14753,-0.15734 0.07987,-0.18581 0.00457,-0.20770 -0.07206,-0.19076 -0.13788,-0.16176 -0.19568,-0.11888 -0.23512,-0.05446 -0.25584,0.01645 -0.23792,0.08536 -0.19943,0.14139 -0.15517,0.17654 -0.12316,0.13507
T_curtipilis -0.00072,0.12089 0.12912,0.14134 0.15026,0.18410 0.19997,0.14578 0.23110,0.08795 0.24183,0.01698 0.22535,-0.05471 0.19184,-0.11731 0.14471,-0.16319 0.08091,-0.18830 0.00699,-0.20391 -0.06864,-0.18808 -0.13387,-0.16523 -0.18815,-0.12223 -0.22872,-0.05898 -0.25050,0.01358 -0.24252,0.08530 -0.20807,0.14398 -0.15486,0.18337 -0.12603,0.13868
T_delahayei -0.00148,0.12615 0.11894,0.13100 0.15332,0.16677 0.19129,0.13317 0.23518,0.08616 0.25563,0.02244 0.24451,-0.04525 0.21095,-0.10514 0.15516,-0.14801 0.08506,-0.17897 0.00250,-0.19606 -0.08153,-0.18627 -0.15065,-0.15869 -0.21025,-0.11787 -0.24854,-0.05731 -0.25707,0.01415 -0.23567,0.08283 -0.19166,0.13282 -0.15382,0.16354 -0.12186,0.13455
T_ebenus -0.00080,0.12647 0.11815,0.13320 0.14243,0.16676 0.18164,0.13864 0.22690,0.09097 0.25307,0.02447 0.24738,-0.04689 0.21644,-0.10945 0.15897,-0.15281 0.08681,-0.18559 0.00166,-0.20040 -0.08349,-0.18475 -0.15431,-0.16235 -0.21052,-0.11825 -0.24932,-0.05749 -0.25655,0.01440 -0.23409,0.08395 -0.18647,0.13521 -0.13958,0.16737 -0.11832,0.13654
T_flexicornis -0.00829,0.13371 0.12080,0.13279 0.14915,0.16513 0.18906,0.13736 0.23313,0.08936 0.25756,0.02403 0.24582,-0.04598 0.21160,-0.10926 0.15477,-0.15431 0.08262,-0.18480 -0.00044,-0.20006 -0.08203,-0.18404 -0.14875,-0.15524 -0.20604,-0.11484 -0.24426,-0.05671 -0.25410,0.01296 -0.23781,0.08099 -0.19463,0.13249 -0.14731,0.16668 -0.12085,0.12975
T_hemichlorus ?
T_jossoi -0.00159,0.10659 0.11929,0.13626 0.14323,0.18271 0.17980,0.14960 0.22382,0.09903 0.24776,0.02959 0.23626,-0.04559 0.20294,-0.11312 0.14868,-0.16275 0.08043,-0.19769 0.00104,-0.21582 -0.07803,-0.19957 -0.14383,-0.17032 -0.19890,-0.12367 -0.23540,-0.05583 -0.24791,0.02155 -0.22916,0.09413 -0.18376,0.14630 -0.14495,0.18150 -0.11973,0.13711
T_katanganus 0.00366,0.12384 0.10709,0.13961 0.14881,0.18137 0.19317,0.13784 0.23241,0.08178 0.24752,0.01430 0.23056,-0.05413 0.19954,-0.11465 0.15054,-0.15684 0.08707,-0.18341 0.01174,-0.19915 -0.06955,-0.18873 -0.13912,-0.16773 -0.19540,-0.12237 -0.23998,-0.05903 -0.25625,0.01552 -0.24001,0.08818 -0.19684,0.14362 -0.15560,0.18239 -0.11936,0.13759
T_lamtoensis -0.00323,0.12991 0.11940,0.13135 0.15318,0.16671 0.19415,0.13638 0.23693,0.08855 0.25473,0.02194 0.24155,-0.04933 0.21199,-0.11265 0.15516,-0.15145 0.08321,-0.17895 0.00209,-0.19780 -0.07888,-0.17985 -0.14786,-0.15521 -0.20572,-0.11619 -0.24221,-0.05472 -0.25770,0.01454 -0.23866,0.08102 -0.19845,0.13253 -0.15706,0.16349 -0.12262,0.12974
T_liberianus -0.00911,0.12300 0.11150,0.13423 0.14297,0.16917 0.19154,0.14103 0.23208,0.08843 0.25489,0.02017 0.25025,-0.04876 0.21191,-0.10691 0.15616,-0.15387 0.08440,-0.18461 0.00126,-0.20157 -0.08212,-0.18707 -0.15112,-0.16248 -0.20654,-0.11833 -0.24260,-0.05390 -0.25380,0.01813 -0.22816,0.08631 -0.18708,0.13824 -0.15079,0.16613 -0.12565,0.13267
T_macroliberianus -0.00071,0.12440 0.12048,0.13694 0.14701,0.17776 0.18364,0.14179 0.22859,0.09093 0.25158,0.02170 0.24374,-0.05049 0.20848,-0.11234 0.15302,-0.15720 0.08063,-0.18363 -0.00214,-0.19872 -0.08197,-0.18800 -0.14711,-0.16286 -0.20186,-0.12052 -0.24050,-0.05926 -0.25678,0.01089 -0.23815,0.07989 -0.19195,0.13616 -0.14318,0.17922 -0.11281,0.13333
T_naevius -0.00729,0.13638 0.11161,0.13679 0.14690,0.17540 0.18070,0.13894 0.22526,0.08599 0.25406,0.01881 0.24610,-0.05115 0.21190,-0.11194 0.15403,-0.15452 0.08243,-0.18593 0.00232,-0.20647 -0.07813,-0.18412 -0.14898,-0.16458 -0.20553,-0.12337 -0.24203,-0.05925 -0.25329,0.01484 -0.23013,0.08529 -0.18417,0.13729 -0.15181,0.17364 -0.11395,0.13796
T_pendjarius -0.01129,0.12889 0.11603,0.13115 0.14389,0.17497 0.19013,0.13811 0.23469,0.08773 0.25066,0.01894 0.24280,-0.05147 0.20820,-0.11015 0.15163,-0.15213 0.08322,-0.18467 0.00400,-0.20243 -0.07698,-0.18726 -0.14511,-0.16071 -0.20357,-0.11989 -0.24363,-0.06092 -0.25170,0.01196 -0.23701,0.08409 -0.19081,0.13777 -0.14594,0.17850 -0.11921,0.13753
T_pseudoliberianus 0.00004,0.12480 0.11812,0.13637 0.14664,0.17009 0.19202,0.13885 0.23492,0.08981 0.25214,0.02204 0.24232,-0.04924 0.20819,-0.11106 0.15489,-0.15607 0.08328,-0.18102 0.00180,-0.20017 -0.07988,-0.18770 -0.14673,-0.15755 -0.20582,-0.11716 -0.24308,-0.05626 -0.25528,0.01379 -0.23369,0.08154 -0.19252,0.13526 -0.14930,0.16812 -0.12805,0.13554
T_rolandoi 0.00075,0.12150 0.10947,0.13337 0.14174,0.17993 0.18923,0.14027 0.23405,0.08541 0.25675,0.01831 0.24264,-0.05054 0.20852,-0.11144 0.15241,-0.15359 0.08314,-0.18388 0.00401,-0.20160 -0.07645,-0.18354 -0.14634,-0.16116 -0.20400,-0.12023 -0.24112,-0.05572 -0.25812,0.01685 -0.23896,0.08553 -0.19240,0.13692 -0.14994,0.17271 -0.11538,0.13088
T_rougonorum -0.00427,0.12593 0.12129,0.14206 0.14660,0.17609 0.18429,0.14798 0.22662,0.09822 0.24939,0.02898 0.23632,-0.04619 0.20425,-0.11512 0.14948,-0.16362 0.07773,-0.19441 -0.00292,-0.21430 -0.08101,-0.19503 -0.14600,-0.16633 -0.19958,-0.12054 -0.23710,-0.05803 -0.24515,0.01538 -0.22481,0.08710 -0.18831,0.14236 -0.14705,0.17210 -0.11979,0.13736
T_rufobasalis -0.00558,0.12229 0.11664,0.13664 0.14145,0.17471 0.18728,0.14609 0.22618,0.09338 0.24681,0.02328 0.23962,-0.04942 0.20588,-0.11234 0.15322,-0.15955 0.08382,-0.18965 0.00427,-0.20946 -0.07623,-0.19157 -0.14530,-0.16474 -0.20363,-0.12176 -0.24143,-0.05675 -0.25499,0.01727 -0.23112,0.08830 -0.19153,0.14486 -0.14545,0.17390 -0.10991,0.13454
T_rufopygus 0.00332,0.11274 0.12115,0.13369 0.15068,0.16859 0.18709,0.14398 0.22635,0.09455 0.25086,0.02668 0.24401,-0.04521 0.20777,-0.10781 0.15031,-0.15538 0.08126,-0.19239 0.00060,-0.20824 -0.07859,-0.18789 -0.14607,-0.16266 -0.20052,-0.11705 -0.23957,-0.05333 -0.25556,0.01770 -0.23548,0.08592 -0.19452,0.13944 -0.15286,0.17321 -0.12023,0.13346
T_rufostillans -0.00258,0.12623 0.11542,0.13803 0.14198,0.17750 0.19927,0.13835 0.23857,0.08275 0.25281,0.01539 0.23864,-0.05231 0.20360,-0.11070 0.14936,-0.15253 0.08203,-0.18213 0.00444,-0.19972 -0.07499,-0.18410 -0.14306,-0.16122 -0.19965,-0.11910 -0.24100,-0.06002 -0.25202,0.01243 -0.23929,0.08389 -0.19848,0.13774 -0.15177,0.17260 -0.12328,0.13691
T_saadaniensis -0.00678,0.11752 0.10564,0.14213 0.13356,0.18308 0.18904,0.14877 0.23604,0.09959 0.24742,0.02925 0.23012,-0.04767 0.20258,-0.11716 0.14379,-0.16100 0.07733,-0.20074 0.00119,-0.22245 -0.07515,-0.20165 -0.13977,-0.16889 -0.19643,-0.12420 -0.22983,-0.05415 -0.24738,0.02270 -0.23357,0.09400 -0.18998,0.14578 -0.13723,0.17622 -0.11059,0.13885
T_schaufussi -0.00543,0.12837 0.12239,0.13064 0.15440,0.16546 0.19528,0.13511 0.23568,0.08496 0.25699,0.01921 0.24823,-0.04794 0.21036,-0.10460 0.15500,-0.14861 0.08179,-0.17258 0.00172,-0.19711 -0.07987,-0.18082 -0.15010,-0.15662 -0.20851,-0.11467 -0.25050,-0.05807 -0.25742,0.01101 -0.23512,0.07981 -0.19398,0.13230 -0.15684,0.16354 -0.12406,0.13062
T_viridiaereus -0.00101,0.11225 0.12007,0.13421 0.14633,0.17505 0.19252,0.14221 0.23648,0.09311 0.25390,0.02570 0.24116,-0.04590 0.20616,-0.10878 0.14764,-0.15278 0.07739,-0.18758 -0.00106,-0.20929 -0.07829,-0.18846 -0.14525,-0.16301 -0.19998,-0.11801 -0.23839,-0.05402 -0.25297,0.01864 -0.23717,0.08677 -0.19295,0.13608 -0.15337,0.17018 -0.12121,0.13363
T_zambesianus -0.00007,0.11797 0.11373,0.13511 0.14525,0.18426 0.19120,0.14227 0.23421,0.08784 0.24966,0.01817 0.23693,-0.05288 0.20282,-0.11322 0.14778,-0.15510 0.08137,-0.18623 0.00375,-0.20055 -0.07511,-0.18777 -0.14172,-0.16529 -0.19704,-0.12275 -0.23742,-0.06039 -0.25367,0.01352 -0.24075,0.08580 -0.19519,0.14132 -0.14802,0.18477 -0.11770,0.13316
T_zavattarii -0.00061,0.11719 0.12029,0.13247 0.14693,0.16689 0.17845,0.14026 0.22353,0.09608 0.24883,0.02961 0.24218,-0.04381 0.20945,-0.10989 0.15812,-0.16023 0.08616,-0.18884 0.00505,-0.21220 -0.07728,-0.19418 -0.14734,-0.16493 -0.20588,-0.11964 -0.24108,-0.05013 -0.25649,0.02534 -0.23448,0.09384 -0.18782,0.14119 -0.14816,0.16799 -0.11989,0.13297

&[landmark 2D]
E_flavimargo -0.10598,0.09266 -0.08047,0.12896 -0.05000,0.17448 -0.01593,0.20881 0.01264,0.25084 0.03570,0.23362 0.06380,0.22505 0.08899,0.20770 0.11053,0.12876 0.12764,0.03537 0.13962,-0.07189 0.14133,-0.18508 0.13319,-0.30008 0.10930,-0.40971 -0.20596,-0.33654 -0.16281,-0.23931 -0.12662,-0.13837 -0.10715,-0.04013 -0.10782,0.03488
M_pallens -0.16828,0.11062 -0.11177,0.10453 -0.05217,0.12996 -0.00502,0.15752 -0.00734,0.22347 0.04458,0.19608 0.09833,0.18323 0.14882,0.17959 0.16073,0.09741 0.17503,0.01605 0.17926,-0.06949 0.17932,-0.15595 0.17525,-0.24264 0.16032,-0.32711 -0.23789,-0.27733 -0.20488,-0.20242 -0.18683,-0.12134 -0.17668,-0.03818 -0.17077,0.03602
H_bituberculatus -0.16265,0.08812 -0.11402,0.10654 -0.05887,0.12758 -0.01167,0.15174 0.01768,0.19824 0.05682,0.18284 0.09868,0.17566 0.14114,0.17080 0.15960,0.10472 0.17227,0.02934 0.17907,-0.05706 0.18801,-0.14844 0.19123,-0.24259 0.18487,-0.33436 -0.24353,-0.27742 -0.22519,-0.19099 -0.20660,-0.10370 -0.18983,-0.02153 -0.17700,0.04050
H_depressus -0.16502,0.08234 -0.11240,0.10512 -0.05463,0.13139 -0.00300,0.15689 0.02690,0.20666 0.06081,0.19395 0.09543,0.18712 0.13036,0.17970 0.14352,0.10869 0.16227,0.03002 0.17820,-0.05895 0.18463,-0.15334 0.18438,-0.24991 0.17233,-0.34297 -0.24586,-0.27757 -0.21335,-0.19623 -0.19484,-0.10972 -0.17915,-0.02757 -0.17056,0.03438
P_nuchicornis ? ? ? ? ? ? ? ? ? ? ? ? ? ? ? ? ? ? ?
O_nigriventris -0.16437,0.09432 -0.11568,0.11052 -0.06547,0.13958 -0.01774,0.16160 0.02365,0.18487 0.06537,0.17999 0.10375,0.15924 0.14147,0.13436 0.15705,0.07594 0.17770,0.01266 0.19455,-0.05876 0.20884,-0.13306 0.21137,-0.21040 0.21181,-0.28760 -0.29060,-0.28283 -0.25200,-0.19664 -0.21701,-0.10630 -0.19309,-0.02077 -0.17960,0.04329
P_coenobita -0.12328,0.09677 -0.08990,0.12720 -0.05864,0.16693 -0.02731,0.19745 -0.01282,0.23849 0.02817,0.23051 0.06579,0.21094 0.10008,0.18639 0.12809,0.10790 0.15199,0.02005 0.16446,-0.07919 0.16374,-0.18293 0.14928,-0.28801 0.12479,-0.38921 -0.21572,-0.31201 -0.17557,-0.21845 -0.13540,-0.12405 -0.11731,-0.03048 -0.12047,0.04170
P_ovatus -0.12108,0.10401 -0.09144,0.13931 -0.06684,0.18170 -0.04710,0.21462 -0.02205,0.24467 0.01523,0.21889 0.05571,0.19246 0.09660,0.16738 0.13281,0.09603 0.15680,0.01074 0.16611,-0.08832 0.16960,-0.19172 0.15899,-0.29599 0.13828,-0.39563 -0.20039,-0.29910 -0.16377,-0.20917 -0.13034,-0.11683 -0.12247,-0.02278 -0.12465,0.04975
P_medius -0.12233,0.09693 -0.08922,0.12825 -0.04289,0.16026 -0.00692,0.19483 0.01549,0.24205 0.03996,0.22826 0.06302,0.21332 0.08716,0.19827 0.11398,0.12147 0.13919,0.03248 0.15477,-0.07002 0.15464,-0.17904 0.14333,-0.29050 0.11864,-0.39758 -0.20763,-0.32776 -0.17049,-0.23025 -0.13780,-0.13043 -0.12420,-0.03284 -0.12869,0.04231
O_illyricus -0.17506,0.07693 -0.12967,0.11947 -0.08411,0.16871 -0.03292,0.20131 0.01630,0.22955 0.06015,0.20161 0.10627,0.17280 0.14802,0.14010 0.16223,0.07252 0.16869,-0.00021 0.17549,-0.07929 0.17534,-0.16018 0.18031,-0.24093 0.17653,-0.31862 -0.22229,-0.27123 -0.19181,-0.19696 -0.18015,-0.11424 -0.17669,-0.03162 -0.17665,0.03028
T_aequatus -0.22439,0.15390 -0.12328,0.10893 -0.06323,0.12723 -0.01494,0.15816 0.02489,0.20329 0.05958,0.18008 0.09460,0.17582 0.12993,0.17417 0.15003,0.09781 0.16536,0.01375 0.17962,-0.07448 0.18458,-0.16274 0.17736,-0.25076 0.16349,-0.33723 -0.21739,-0.27742 -0.18513,-0.19912 -0.16223,-0.11641 -0.15632,-0.03025 -0.18251,0.05526
T_angolensis -0.20948,0.14390 -0.14360,0.10957 -0.08281,0.12835 -0.02015,0.15476 0.01529,0.21621 0.05569,0.19360 0.09379,0.18199 0.13106,0.17400 0.15274,0.09148 0.16571,0.00548 0.18136,-0.08236 0.18454,-0.17001 0.17675,-0.25705 0.15382,-0.34135 -0.21329,-0.27669 -0.17688,-0.19653 -0.15023,-0.11238 -0.14780,-0.02381 -0.16649,0.06083
T_chrysoderus -0.17965,0.15674 -0.13809,0.12462 -0.09089,0.14750 -0.03612,0.17465 0.01312,0.21536 0.05089,0.19662 0.08656,0.18081 0.12311,0.16868 0.14074,0.09039 0.15177,0.00347 0.17129,-0.08685 0.17668,-0.17792 0.17587,-0.26784 0.15899,-0.35399 -0.20811,-0.28399 -0.16158,-0.20805 -0.13959,-0.11999 -0.13975,-0.02514 -0.15525,0.06494
T_curtipilis -0.15441,0.13564 -0.10335,0.10551 -0.06781,0.14354 -0.03258,0.18117 0.00702,0.22113 0.04328,0.20561 0.08008,0.19321 0.11773,0.18353 0.13861,0.10443 0.16251,0.01666 0.17255,-0.07966 0.17717,-0.17690 0.16832,-0.27319 0.14670,-0.36516 -0.19776,-0.28109 -0.19052,-0.19817 -0.15908,-0.11979 -0.14731,-0.03778 -0.16119,0.04133
T_delahayei -0.20770,0.15888 -0.13591,0.11967 -0.07040,0.12762 -0.01509,0.15948 0.01903,0.22267 0.05967,0.19816 0.09451,0.17919 0.13062,0.17334 0.14331,0.08760 0.15567,0.00092 0.17026,-0.08647 0.17744,-0.17210 0.17134,-0.25697 0.16371,-0.34109 -0.19462,-0.27985 -0.16845,-0.20066 -0.14714,-0.11979 -0.16129,-0.03159 -0.18495,0.06098
T_ebenus -0.17027,0.15684 -0.15002,0.10825 -0.10484,0.14224 -0.04773,0.18226 0.00844,0.23294 0.05084,0.20170 0.08988,0.17044 0.13510,0.15305 0.15983,0.07149 0.17427,-0.01223 0.18660,-0.09525 0.18789,-0.17479 0.18194,-0.25180 0.16349,-0.32634 -0.20773,-0.25996 -0.17589,-0.19141 -0.15428,-0.11820 -0.15531,-0.03739 -0.17221,0.04815
T_flexicornis -0.17909,0.16154 -0.12622,0.11009 -0.08521,0.14118 -0.04095,0.18137 0.00347,0.23361 0.04447,0.20596 0.08519,0.18669 0.12653,0.17541 0.14322,0.08863 0.16363,0.00044 0.16858,-0.09110 0.17175,-0.18081 0.16518,-0.26901 0.15032,-0.35519 -0.19550,-0.28205 -0.16980,-0.20166 -0.14076,-0.12131 -0.13220,-0.03593 -0.15264,0.05213
T_hemichlorus ? ? ? ? ? ? ? ? ? ? ? ? ? ? ? ? ? ? ?
T_jossoi -0.22121,0.11925 -0.13319,0.10871 -0.07079,0.14106 -0.01738,0.17247 0.02682,0.20997 0.06367,0.18928 0.09962,0.17632 0.13418,0.16175 0.15178,0.08716 0.16936,0.00534 0.18676,-0.08103 0.19023,-0.16800 0.18206,-0.25421 0.16716,-0.33665 -0.19922,-0.24910 -0.17768,-0.18097 -0.16975,-0.10866 -0.17779,-0.03220 -0.20464,0.03950
T_katanganus -0.19071,0.14990 -0.13678,0.10744 -0.09033,0.13694 -0.03622,0.17239 0.01185,0.22281 0.05098,0.20085 0.08874,0.18753 0.12739,0.18164 0.14903,0.10042 0.16518,0.01163 0.17348,-0.08250 0.17500,-0.17602 0.16231,-0.26814 0.13909,-0.35739 -0.21632,-0.28117 -0.16638,-0.20498 -0.13138,-0.12158 -0.12391,-0.03289 -0.15104,0.05313
T_lamtoensis -0.17966,0.16784 -0.13778,0.10982 -0.08948,0.13559 -0.03257,0.16914 0.02343,0.21345 0.05544,0.19594 0.08535,0.18464 0.11761,0.18089 0.13116,0.10025 0.15619,0.01249 0.16843,-0.08332 0.17482,-0.17976 0.17344,-0.27525 0.15600,-0.36697 -0.19117,-0.28258 -0.16676,-0.19989 -0.14607,-0.11486 -0.13822,-0.02748 -0.16017,0.06006
T_liberianus -0.16936,0.13317 -0.12647,0.12247 -0.07843,0.14634 -0.03437,0.17511 -0.00095,0.22243 0.04146,0.19646 0.08139,0.17318 0.12555,0.16983 0.14664,0.09296 0.16955,0.00900 0.18371,-0.08298 0.18458,-0.17742 0.17254,-0.27204 0.14880,-0.36285 -0.18645,-0.27658 -0.16887,-0.19410 -0.16084,-0.10827 -0.15579,-0.02210 -0.17271,0.05539
T_macroliberianus -0.20637,0.15643 -0.15428,0.11078 -0.09061,0.12138 -0.02687,0.14014 0.00836,0.20004 0.05129,0.17879 0.09241,0.16823 0.13374,0.16587 0.15931,0.08641 0.18101,0.00375 0.19499,-0.08245 0.19841,-0.16848 0.19443,-0.25376 0.17553,-0.33605 -0.22296,-0.25366 -0.18843,-0.17800 -0.15701,-0.10185 -0.15596,-0.02006 -0.18697,0.06250
T_naevius -0.23565,0.11920 -0.16018,0.09090 -0.09073,0.11700 -0.02176,0.15913 0.03084,0.22200 0.07166,0.19760 0.10898,0.17914 0.14506,0.16266 0.16257,0.07999 0.18250,-0.00039 0.18710,-0.08322 0.19058,-0.16443 0.18024,-0.24445 0.16428,-0.32315 -0.21185,-0.24944 -0.18552,-0.17675 -0.16422,-0.10264 -0.16149,-0.02699 -0.19241,0.04382
T_pendjarius -0.20422,0.15282 -0.14851,0.09690 -0.10190,0.12859 -0.05145,0.17387 -0.00318,0.23163 0.04578,0.20157 0.09302,0.17683 0.14054,0.16055 0.16253,0.07288 0.18053,-0.01345 0.18862,-0.09913 0.18863,-0.18123 0.18267,-0.26090 0.16468,-0.33756 -0.20033,-0.24630 -0.17512,-0.17504 -0.15131,-0.10265 -0.14006,-0.02811 -0.17093,0.04875
T_pseudoliberianus -0.23930,0.13082 -0.16002,0.08856 -0.10152,0.11912 -0.03855,0.15517 0.01820,0.19826 0.06591,0.18149 0.11096,0.17011 0.15333,0.15535 0.16805,0.07774 0.18339,-0.00409 0.19449,-0.08848 0.19755,-0.17125 0.20258,-0.25124 0.19538,-0.32662 -0.20170,-0.21784 -0.19134,-0.15449 -0.18051,-0.08973 -0.17804,-0.02110 -0.19887,0.04823
T_rolandoi -0.24113,0.15418 -0.15705,0.09479 -0.08621,0.12035 -0.01605,0.15574 0.03753,0.21606 0.07023,0.18447 0.10735,0.17452 0.14276,0.16029 0.16070,0.07513 0.17558,-0.00776 0.18486,-0.08856 0.19212,-0.16416 0.19008,-0.23694 0.17754,-0.30804 -0.21471,-0.24603 -0.18459,-0.18130 -0.16515,-0.11257 -0.16997,-0.03658 -0.20389,0.04642
T_rougonorum -0.19271,0.15531 -0.11827,0.11539 -0.07651,0.14341 -0.03878,0.17876 -0.00257,0.22487 0.04288,0.20229 0.08538,0.18511 0.12668,0.17621 0.13832,0.09197 0.16090,0.00466 0.16975,-0.08846 0.17336,-0.18145 0.16966,-0.27343 0.14997,-0.36191 -0.19664,-0.28354 -0.17055,-0.19899 -0.13903,-0.11534 -0.12663,-0.02960 -0.15519,0.05476
T_rufobasalis -0.18921,0.14592 -0.13860,0.09012 -0.10995,0.13669 -0.06628,0.19060 -0.01137,0.24863 0.04087,0.21412 0.09306,0.18441 0.14593,0.16539 0.16039,0.07221 0.17937,-0.01763 0.18907,-0.10502 0.18354,-0.18750 0.17122,-0.26677 0.15440,-0.34290 -0.19497,-0.23656 -0.16811,-0.17579 -0.14810,-0.11175 -0.13714,-0.04105 -0.15410,0.03688
T_rufopygus -0.24424,0.14662 -0.17101,0.05985 -0.11445,0.09956 -0.04388,0.14965 0.03039,0.20374 0.07990,0.17951 0.12832,0.16755 0.17696,0.16728 0.18745,0.08654 0.19810,0.00378 0.19971,-0.07826 0.19658,-0.15620 0.18887,-0.23126 0.17215,-0.30328 -0.21711,-0.21797 -0.20459,-0.15941 -0.18374,-0.10377 -0.17610,-0.04340 -0.20331,0.02947
T_rufostillans -0.22740,0.11331 -0.13222,0.12138 -0.07029,0.14376 -0.02284,0.17912 0.01278,0.22513 0.04859,0.19636 0.08418,0.17669 0.11742,0.15697 0.14457,0.08073 0.17066,0.00114 0.18649,-0.08490 0.18764,-0.17389 0.17864,-0.26437 0.15578,-0.35211 -0.21095,-0.27693 -0.17565,-0.18744 -0.14423,-0.09649 -0.12968,-0.01231 -0.17349,0.05384
T_saadaniensis -0.26110,0.11853 -0.11412,0.10094 -0.04086,0.14140 0.01276,0.17809 0.04424,0.22612 0.07564,0.20390 0.09649,0.18546 0.11651,0.17010 0.13851,0.08886 0.15796,0.00338 0.17215,-0.08428 0.17593,-0.16994 0.17779,-0.25280 0.17103,-0.33087 -0.20609,-0.24557 -0.17843,-0.18545 -0.16135,-0.12165 -0.16990,-0.05060 -0.20717,0.02438
T_schaufussi -0.22373,0.16829 -0.16202,0.09865 -0.09153,0.11283 -0.01962,0.14444 0.03454,0.20226 0.06923,0.18580 0.09859,0.17364 0.12848,0.16210 0.15383,0.08080 0.16892,-0.00446 0.18687,-0.08903 0.19625,-0.17096 0.19547,-0.25125 0.18884,-0.32928 -0.19739,-0.24411 -0.18147,-0.17412 -0.16906,-0.10182 -0.17318,-0.02392 -0.20303,0.06013
T_viridiaereus -0.19028,0.15551 -0.12292,0.12499 -0.07082,0.15623 -0.02413,0.19715 0.01950,0.24773 0.04892,0.21339 0.07744,0.18585 0.10505,0.16157 0.11890,0.07266 0.14234,-0.01434 0.16678,-0.10078 0.17399,-0.18700 0.17360,-0.27195 0.15866,-0.35464 -0.17642,-0.28690 -0.16088,-0.20383 -0.13748,-0.12049 -0.13930,-0.03158 -0.16293,0.05644
T_zambesianus -0.20873,0.14279 -0.12127,0.08714 -0.06475,0.12796 -0.02131,0.18660 0.03184,0.24547 0.06238,0.21227 0.09462,0.18980 0.12816,0.17220 0.14903,0.08235 0.16546,-0.00668 0.17645,-0.09292 0.17828,-0.17357 0.17488,-0.24987 0.15308,-0.32182 -0.19958,-0.25525 -0.18457,-0.19069 -0.16382,-0.12674 -0.16333,-0.05528 -0.18681,0.02624
T_zavattarii -0.22270,0.13250 -0.12882,0.11235 -0.07850,0.15042 -0.03072,0.16988 0.00376,0.20276 0.05460,0.17836 0.10526,0.16358 0.15177,0.14680 0.16763,0.07311 0.18048,-0.00479 0.19074,-0.08558 0.19689,-0.16459 0.19424,-0.24178 0.18289,-0.31576 -0.21031,-0.24127 -0.18815,-0.17684 -0.17712,-0.10785 -0.18421,-0.03300 -0.20773,0.04168

&[landmark 2D]
E_flavimargo -0.32378,-0.01681 -0.28322,0.06984 -0.26370,0.12343 -0.03621,0.18571 0.07429,0.14586 0.17634,0.08639 0.26285,0.02801 0.33331,-0.03460 0.29092,-0.04767 0.23090,-0.06288 0.13567,-0.11572 0.05247,-0.16994 0.06250,-0.24949 -0.00615,-0.16436 -0.03816,-0.07841 -0.12799,-0.01746 -0.21823,0.01831 -0.20073,0.13627 -0.12109,0.16354
M_pallens -0.34792,-0.07358 -0.25368,0.07086 -0.20031,0.18194 0.03176,0.18647 0.10341,0.15903 0.18669,0.11923 0.26743,0.07327 0.35436,-0.02018 0.29404,0.00796 0.22456,-0.02926 0.07995,-0.07283 -0.01166,-0.16542 0.00706,-0.28033 -0.06335,-0.18550 -0.08638,-0.10274 -0.15714,-0.07074 -0.23857,-0.05178 -0.14146,0.12855 -0.04878,0.12504
H_bituberculatus -0.37691,-0.04897 -0.32984,0.04134 -0.23046,0.09804 0.02236,0.17813 0.11508,0.15296 0.18507,0.09812 0.24182,0.04635 0.31345,-0.05642 0.27507,-0.01215 0.23735,-0.04310 0.14649,-0.09677 0.05280,-0.14754 0.02048,-0.23390 -0.00619,-0.14651 -0.04412,-0.07239 -0.14398,-0.02098 -0.26339,-0.03519 -0.15046,0.13425 -0.06462,0.16470
H_depressus -0.35999,-0.08662 -0.31572,0.02419 -0.22895,0.10150 0.02564,0.18508 0.12178,0.15619 0.20815,0.11985 0.26630,0.07052 0.32044,-0.03426 0.27322,-0.00944 0.22027,-0.06338 0.10627,-0.10303 0.00783,-0.15039 -0.01765,-0.24027 -0.04068,-0.15449 -0.04985,-0.07600 -0.10252,-0.01244 -0.23602,-0.04631 -0.14213,0.14293 -0.05640,0.17639
P_nuchicornis ? ? ? ? ? ? ? ? ? ? ? ? ? ? ? ? ? ? ?
O_nigriventris -0.40995,-0.07321 -0.35000,0.03268 -0.26117,0.11556 -0.01164,0.14313 0.08883,0.09891 0.17880,0.05026 0.25186,0.00439 0.32235,-0.07459 0.27923,-0.04550 0.23428,-0.06095 0.16092,-0.06850 0.10922,-0.12095 0.09070,-0.16199 0.03634,-0.11641 -0.03374,-0.05657 -0.13635,0.02625 -0.26993,-0.01223 -0.18289,0.15756 -0.09685,0.16216
P_coenobita -0.39101,-0.04228 -0.34020,0.05157 -0.25815,0.12913 0.00484,0.15138 0.10038,0.11911 0.17607,0.07137 0.24981,0.03023 0.32248,-0.05505 0.26989,-0.02997 0.21106,-0.04601 0.13114,-0.07387 0.08576,-0.14233 0.07128,-0.22404 0.02710,-0.14770 -0.02072,-0.07390 -0.12558,-0.02292 -0.25433,-0.02831 -0.17597,0.16427 -0.08388,0.16933
P_ovatus -0.34231,-0.00613 -0.32722,0.08737 -0.29404,0.14596 -0.02903,0.13679 0.07343,0.09299 0.15923,0.05043 0.23510,0.01112 0.33460,-0.05312 0.27386,-0.03598 0.22389,-0.06507 0.15364,-0.09831 0.11942,-0.16108 0.13237,-0.21042 0.04733,-0.16631 -0.03012,-0.09179 -0.14936,-0.01264 -0.24188,0.02442 -0.21815,0.17494 -0.12076,0.17684
P_medius -0.39702,-0.03917 -0.32258,0.04521 -0.24895,0.12545 -0.02287,0.16643 0.08372,0.13535 0.17216,0.07627 0.24787,0.02033 0.30927,-0.07213 0.26680,-0.05633 0.22043,-0.06943 0.15706,-0.08908 0.11098,-0.13869 0.09992,-0.21819 0.04357,-0.13357 -0.02119,-0.05720 -0.14707,-0.01552 -0.25935,-0.00658 -0.18512,0.15854 -0.10761,0.16832
O_illyricus -0.39176,-0.10133 -0.33582,0.02045 -0.25244,0.12541 0.01628,0.14172 0.10912,0.10085 0.18442,0.05540 0.25884,0.02148 0.33485,-0.06929 0.27726,-0.03220 0.21092,-0.04662 0.14107,-0.07379 0.08017,-0.11276 0.06790,-0.18837 0.01492,-0.11179 -0.03782,-0.03668 -0.15863,0.00088 -0.27491,-0.03381 -0.16970,0.16627 -0.07468,0.17417
T_aequatus -0.23140,-0.15416 -0.26399,0.01238 -0.26986,0.14193 -0.04771,0.18306 0.06612,0.11754 0.19129,0.06515 0.29854,0.01664 0.37137,-0.09186 0.32595,-0.04908 0.25717,-0.05501 0.10323,-0.07790 -0.01779,-0.10969 -0.02745,-0.20763 -0.06189,-0.12014 -0.06266,-0.03849 -0.10307,0.02908 -0.18465,-0.04659 -0.21168,0.18734 -0.13152,0.19742
T_angolensis -0.28553,-0.15486 -0.27826,0.00091 -0.25271,0.11567 -0.01544,0.18765 0.07451,0.13456 0.18573,0.08338 0.29162,0.02330 0.39557,-0.07325 0.32126,-0.04462 0.22945,-0.05845 0.07991,-0.07606 -0.02302,-0.11252 -0.01954,-0.22311 -0.06197,-0.11842 -0.07778,-0.01907 -0.10593,0.05348 -0.19384,-0.04494 -0.17377,0.14990 -0.09025,0.17643
T_chrysoderus -0.33867,-0.08837 -0.32092,0.02667 -0.25258,0.10321 -0.00157,0.16573 0.10743,0.12787 0.20224,0.07784 0.28622,0.03178 0.34004,-0.00992 0.29455,-0.03855 0.20975,-0.05727 0.10637,-0.08935 0.01493,-0.14202 -0.00145,-0.22935 -0.04194,-0.14396 -0.01877,-0.04845 -0.09414,0.01406 -0.22942,-0.02461 -0.17444,0.15111 -0.08762,0.17359
T_curtipilis -0.31813,-0.11678 -0.28501,0.01321 -0.21494,0.11371 0.02120,0.17018 0.11622,0.14633 0.21287,0.11708 0.29827,0.06686 0.36142,-0.01811 0.29660,-0.01963 0.19596,-0.04728 0.07138,-0.10761 -0.02071,-0.15010 -0.02166,-0.22880 -0.06709,-0.15171 -0.09429,-0.07642 -0.12492,0.00052 -0.22779,-0.04993 -0.14025,0.15998 -0.05914,0.17849
T_delahayei -0.24239,-0.05553 -0.25609,0.03048 -0.23580,0.08979 0.00147,0.15758 0.10796,0.14240 0.22232,0.11194 0.32754,0.06127 0.40708,-0.00109 0.33674,-0.01241 0.22510,-0.03264 0.05729,-0.09002 -0.05695,-0.16015 -0.07482,-0.24220 -0.10557,-0.16217 -0.11651,-0.07428 -0.13503,-0.00286 -0.20750,-0.02526 -0.16914,0.11962 -0.08570,0.14553
T_ebenus -0.31745,-0.10592 -0.30674,0.02159 -0.24774,0.11649 0.00229,0.17860 0.09527,0.14455 0.18159,0.10177 0.26128,0.04429 0.35234,-0.08309 0.28905,-0.02478 0.20993,-0.04435 0.11011,-0.08658 0.02663,-0.15840 0.00871,-0.22684 -0.04205,-0.16117 -0.04223,-0.05251 -0.10726,0.03480 -0.22188,-0.03734 -0.17030,0.16209 -0.08155,0.17679
T_flexicornis -0.28882,-0.14635 -0.27221,-0.01127 -0.22131,0.10832 0.00623,0.20432 0.09429,0.16470 0.19126,0.11594 0.29142,0.04864 0.39060,-0.05670 0.29866,-0.04218 0.17817,-0.05012 0.05121,-0.09267 -0.02856,-0.15147 -0.02977,-0.24808 -0.06869,-0.15684 -0.08426,-0.04384 -0.10307,0.04735 -0.17653,-0.07266 -0.15516,0.17806 -0.07346,0.20485
T_hemichlorus ? ? ? ? ? ? ? ? ? ? ? ? ? ? ? ? ? ? ?
T_jossoi -0.26424,-0.16033 -0.26043,-0.00970 -0.23795,0.11505 0.03442,0.18589 0.10233,0.14701 0.18522,0.10697 0.30280,0.06318 0.42593,-0.07985 0.31949,-0.01243 0.17335,-0.02013 0.01273,-0.05785 -0.06906,-0.13831 -0.03383,-0.26966 -0.09695,-0.15501 -0.10532,-0.03839 -0.10064,0.03351 -0.17682,-0.07082 -0.15617,0.17222 -0.05486,0.18864
T_katanganus -0.36541,-0.12403 -0.32655,0.00983 -0.25405,0.11493 0.00577,0.17780 0.09420,0.12399 0.18484,0.07387 0.27396,0.02673 0.36347,-0.06331 0.29366,-0.04054 0.21136,-0.05439 0.11046,-0.07407 0.01614,-0.10612 0.00752,-0.20831 -0.03590,-0.11267 -0.00789,-0.02055 -0.09832,0.01452 -0.23066,-0.05347 -0.16691,0.14826 -0.07569,0.16752
T_lamtoensis -0.35867,-0.11240 -0.32588,0.00065 -0.27362,0.09119 0.04175,0.16834 0.13816,0.13444 0.21172,0.08112 0.27333,0.02961 0.32598,-0.00814 0.27633,-0.03357 0.19702,-0.06108 0.10517,-0.08491 0.01832,-0.14483 0.00757,-0.24392 -0.04878,-0.14536 -0.02791,-0.01403 -0.10187,0.07383 -0.22521,-0.03681 -0.17263,0.13715 -0.06079,0.16872
T_liberianus -0.18284,-0.12754 -0.24141,0.00343 -0.25888,0.11552 -0.05406,0.17914 0.06514,0.15012 0.19817,0.10456 0.33295,0.04366 0.41622,-0.15121 0.34096,-0.05071 0.20654,-0.04259 0.06362,-0.10100 -0.02113,-0.14491 -0.00794,-0.18721 -0.06885,-0.13900 -0.11032,-0.03655 -0.17243,0.06496 -0.14545,-0.02698 -0.21592,0.16288 -0.14435,0.18342
T_macroliberianus -0.27885,-0.12115 -0.27767,0.01647 -0.25008,0.12623 -0.03147,0.19352 0.07681,0.14517 0.18914,0.09096 0.28436,0.03545 0.35292,-0.05844 0.29893,-0.04751 0.22250,-0.07326 0.10278,-0.10905 0.01038,-0.15312 -0.00427,-0.22476 -0.04258,-0.14991 -0.05920,-0.04771 -0.10649,0.04807 -0.19273,-0.03826 -0.18522,0.17303 -0.10926,0.19427
T_naevius -0.29140,-0.10502 -0.28885,0.02216 -0.24190,0.11252 -0.01861,0.16184 0.08447,0.13685 0.19415,0.09829 0.29891,0.04596 0.38217,-0.08948 0.32470,-0.02237 0.23530,-0.02349 0.09110,-0.06790 -0.01590,-0.13399 -0.02959,-0.21032 -0.07167,-0.14184 -0.07999,-0.05939 -0.09913,0.00986 -0.21088,-0.03805 -0.17016,0.14644 -0.09273,0.15792
T_pendjarius -0.32399,-0.13341 -0.28655,-0.00714 -0.22661,0.09765 -0.01464,0.17967 0.08213,0.13911 0.18028,0.09161 0.27583,0.03264 0.38713,-0.05541 0.30102,-0.04031 0.20179,-0.05997 0.08412,-0.09584 -0.01369,-0.14162 -0.00059,-0.24934 -0.06436,-0.14306 -0.04566,-0.01275 -0.09399,0.08162 -0.18519,-0.05167 -0.16551,0.17077 -0.09155,0.19744
T_pseudoliberianus -0.25917,-0.14759 -0.25572,-0.00548 -0.22367,0.11298 -0.03526,0.19152 0.06734,0.14511 0.19006,0.08739 0.31195,0.03048 0.41215,-0.08186 0.33763,-0.04022 0.23565,-0.04197 0.07430,-0.07106 -0.03684,-0.12602 -0.05688,-0.19215 -0.08979,-0.13273 -0.10218,-0.04583 -0.11692,0.03101 -0.18617,-0.06157 -0.16508,0.16118 -0.10139,0.18680
T_rolandoi -0.29927,-0.11128 -0.28074,0.01183 -0.24316,0.09769 -0.02801,0.17274 0.07969,0.13816 0.19681,0.09567 0.29872,0.03850 0.39129,-0.05086 0.32218,-0.03199 0.23281,-0.05488 0.08449,-0.09538 -0.02624,-0.12253 -0.06005,-0.24219 -0.05407,-0.12705 -0.05338,-0.02762 -0.09038,0.04225 -0.19529,-0.02839 -0.17573,0.13839 -0.09966,0.15697
T_rougonorum -0.32054,-0.12249 -0.27518,0.00595 -0.20980,0.10760 -0.00307,0.19034 0.10196,0.15722 0.20754,0.10738 0.30049,0.05983 0.35221,-0.00781 0.29949,-0.02627 0.20295,-0.07174 0.07756,-0.10236 -0.01543,-0.16385 -0.04915,-0.23423 -0.07429,-0.16366 -0.06039,-0.06015 -0.10212,0.02666 -0.20973,-0.05073 -0.14669,0.16241 -0.07582,0.18592
T_rufobasalis -0.33237,-0.09552 -0.31289,0.03922 -0.24375,0.12238 -0.03764,0.17217 0.05852,0.11792 0.16777,0.07446 0.27259,0.03171 0.37693,-0.02652 0.30209,-0.04172 0.20945,-0.08011 0.11061,-0.10598 0.02435,-0.14694 0.01198,-0.25453 -0.03179,-0.14148 -0.00033,-0.01759 -0.09125,0.05839 -0.21023,-0.01868 -0.17172,0.14845 -0.10232,0.16437
T_rufopygus -0.26889,-0.08882 -0.26305,0.04932 -0.22416,0.14609 -0.08338,0.16880 0.04323,0.11576 0.18491,0.07847 0.30248,0.04748 0.36965,-0.07289 0.32054,-0.03053 0.24688,-0.04032 0.12314,-0.10307 0.01333,-0.15859 -0.04602,-0.23241 -0.04228,-0.15488 -0.03316,-0.05833 -0.11834,0.01709 -0.19751,-0.02646 -0.18523,0.16736 -0.14212,0.17592
T_rufostillans -0.24778,-0.15645 -0.26249,0.00074 -0.24771,0.11811 -0.03866,0.18577 0.06328,0.13689 0.17582,0.08638 0.28376,0.02176 0.39503,-0.10316 0.32088,-0.04651 0.23023,-0.03991 0.09517,-0.07713 -0.01572,-0.13601 -0.02803,-0.24519 -0.07116,-0.13915 -0.05880,-0.01186 -0.11170,0.07768 -0.18015,-0.03738 -0.18727,0.17027 -0.11469,0.19515
T_saadaniensis -0.37229,-0.06515 -0.30849,0.01834 -0.22544,0.09437 0.03093,0.16035 0.12655,0.12250 0.21998,0.09038 0.29261,0.05294 0.34180,-0.01579 0.30229,0.00056 0.24372,-0.02133 0.10255,-0.06967 -0.00785,-0.13358 -0.03228,-0.18487 -0.06762,-0.14356 -0.09125,-0.11012 -0.11407,-0.05276 -0.24561,-0.05425 -0.14178,0.14200 -0.05374,0.16963
T_schaufussi -0.27501,-0.10479 -0.27375,0.01438 -0.26089,0.11081 -0.06520,0.18049 0.05613,0.13476 0.18229,0.08196 0.28935,0.02010 0.38577,-0.08425 0.31951,-0.05699 0.24054,-0.06786 0.11635,-0.10805 0.00518,-0.13086 -0.02164,-0.21538 -0.04653,-0.12288 -0.03280,-0.01894 -0.09619,0.06185 -0.17886,-0.02844 -0.20566,0.15534 -0.13860,0.17875
T_viridiaereus -0.33735,-0.08956 -0.30865,0.03826 -0.24243,0.11815 0.00466,0.19043 0.10189,0.15680 0.18313,0.09794 0.25863,0.03782 0.32782,-0.01925 0.27595,-0.03151 0.20375,-0.04540 0.09826,-0.08233 0.02473,-0.17166 -0.00754,-0.28160 -0.02246,-0.18040 -0.02408,-0.07210 -0.07926,0.00563 -0.21857,-0.02249 -0.16252,0.16459 -0.07594,0.18667
T_zambesianus -0.29691,-0.09492 -0.29646,0.01967 -0.26217,0.10056 -0.04619,0.17112 0.06663,0.13679 0.17627,0.08046 0.27865,0.01984 0.37416,-0.09742 0.31548,-0.04543 0.24052,-0.04712 0.11086,-0.06617 0.00661,-0.12617 -0.01353,-0.25222 -0.03728,-0.13101 -0.02004,-0.01494 -0.08392,0.05174 -0.19477,-0.02339 -0.19586,0.14590 -0.12206,0.17270
T_zavattarii -0.25406,-0.16161 -0.26062,-0.00595 -0.23555,0.12066 -0.03916,0.17173 0.06667,0.13292 0.17898,0.07795 0.30781,0.02389 0.44271,-0.09834 0.34247,-0.03672 0.21417,-0.02864 0.06507,-0.06515 -0.03845,-0.11538 -0.06753,-0.19349 -0.08285,-0.12398 -0.06906,-0.03763 -0.09586,0.03233 -0.18307,-0.05617 -0.18028,0.17559 -0.11138,0.18799

&[landmark 2D]
E_flavimargo 0.09609,0.10439 0.44923,0.03931 0.36242,0.00789 0.16535,-0.00183 0.13387,0.01265 0.12181,0.06417 0.16613,-0.14825 0.14041,-0.14986 -0.00844,-0.15625 -0.11391,-0.12245 -0.35361,-0.02107 -0.31821,0.01919 -0.28444,0.04972 -0.26113,0.06660 -0.10348,0.04085 -0.08983,0.00708 0.03576,0.07460 -0.13801,0.11325
M_pallens ? ? ? ? ? ? ? ? ? ? ? ? ? ? ? ? ? ?
H_bituberculatus 0.09773,0.08909 0.43722,0.06444 0.37146,0.01434 0.18001,-0.00765 0.12403,-0.00071 0.11050,0.04863 0.18892,-0.15128 0.17522,-0.15471 -0.03059,-0.14268 -0.11092,-0.11882 -0.34332,-0.01258 -0.30145,0.04408 -0.27316,0.05248 -0.25383,0.06350 -0.15983,0.04359 -0.14394,0.01113 0.03839,0.05251 -0.10645,0.10463
H_depressus 0.09791,0.09249 0.39564,0.05751 0.36482,0.01568 0.18163,-0.01644 0.13825,0.00947 0.11142,0.05772 0.20830,-0.16919 0.18559,-0.16995 -0.02221,-0.15257 -0.11471,-0.11016 -0.33824,-0.00770 -0.30051,0.03874 -0.28840,0.05218 -0.27023,0.06268 -0.14548,0.04411 -0.13460,0.01492 0.04070,0.06308 -0.10990,0.11741
P_nuchicornis 0.09668,0.10529 0.42389,0.05945 0.36926,0.01406 0.17996,-0.02391 0.11819,-0.00601 0.10180,0.06019 0.17930,-0.16373 0.16267,-0.16586 0.01164,-0.15588 -0.11598,-0.10962 -0.33612,-0.02157 -0.30754,0.03483 -0.29289,0.05594 -0.26860,0.06781 -0.10944,0.04948 -0.10669,0.01710 0.02665,0.06477 -0.13278,0.11768
O_nigriventris 0.09777,0.09433 0.42624,0.05376 0.37778,0.01950 0.17752,-0.01751 0.12232,0.00975 0.11439,0.05195 0.17422,-0.15770 0.16049,-0.16039 0.00561,-0.14476 -0.13227,-0.10329 -0.33106,-0.00549 -0.31758,0.02446 -0.29626,0.04456 -0.26241,0.07006 -0.10608,0.04293 -0.10788,0.01187 0.04328,0.05734 -0.14608,0.10862
P_coenobita 0.09912,0.09721 0.42930,0.05599 0.37031,0.02065 0.16563,-0.00046 0.12891,-0.00185 0.11200,0.05484 0.17181,-0.16326 0.16191,-0.16238 0.00681,-0.15662 -0.12236,-0.10595 -0.34338,-0.01852 -0.30974,0.03834 -0.28202,0.05850 -0.26615,0.06507 -0.11742,0.03992 -0.11345,0.00965 0.04386,0.05888 -0.13515,0.10999
P_ovatus 0.09595,0.11164 0.44601,0.05366 0.38125,0.00756 0.18348,-0.01015 0.11406,0.00920 0.11254,0.06074 0.16059,-0.16098 0.14299,-0.16147 -0.00824,-0.14732 -0.11866,-0.11736 -0.33147,-0.01891 -0.30567,0.02515 -0.28474,0.04431 -0.26905,0.05867 -0.10523,0.04644 -0.09126,0.02197 0.02494,0.06582 -0.14750,0.11105
P_medius 0.09246,0.09953 0.43494,0.06201 0.36757,0.01618 0.17956,-0.02060 0.10588,0.00426 0.11002,0.05703 0.17534,-0.16570 0.16688,-0.16831 0.01495,-0.14938 -0.12044,-0.10252 -0.34399,-0.01718 -0.31115,0.03416 -0.28134,0.05537 -0.26167,0.07028 -0.11820,0.04874 -0.10880,0.00968 0.02770,0.06038 -0.12971,0.10608
O_illyricus 0.08935,0.09455 0.43730,0.05679 0.36103,0.02388 0.15864,-0.01737 0.12221,-0.00290 0.09736,0.05663 0.18966,-0.17252 0.17281,-0.17137 0.01259,-0.14217 -0.11910,-0.11838 -0.33161,-0.00732 -0.30837,0.03344 -0.28166,0.06334 -0.26943,0.06675 -0.11225,0.04477 -0.10896,0.01000 0.03580,0.06433 -0.14535,0.11755
T_aequatus ? ? ? ? ? ? ? ? ? ? ? ? ? ? ? ? ? ?
T_angolensis 0.09418,0.09587 0.42211,0.05872 0.37102,0.01562 0.18871,-0.01288 0.11900,0.00104 0.11601,0.05853 0.18147,-0.16065 0.16005,-0.15511 0.00922,-0.14679 -0.12563,-0.10750 -0.35390,-0.00820 -0.30885,0.04116 -0.27979,0.05906 -0.26029,0.05970 -0.12286,0.03447 -0.12025,0.01083 0.04266,0.05688 -0.13288,0.09927
T_chrysoderus 0.08900,0.10604 0.41526,0.06761 0.35670,0.01528 0.19264,-0.02010 0.11643,-0.00063 0.11719,0.06168 0.18364,-0.16402 0.15812,-0.17131 0.01822,-0.16310 -0.11796,-0.12083 -0.34793,-0.01764 -0.30301,0.04793 -0.28249,0.06543 -0.26641,0.07224 -0.12282,0.04081 -0.11194,0.01348 0.03598,0.06196 -0.13063,0.10516
T_curtipilis 0.08685,0.09391 0.41825,0.07081 0.36979,0.02222 0.18737,-0.01575 0.10883,-0.00179 0.10437,0.05680 0.19516,-0.17187 0.16006,-0.17817 0.01585,-0.15916 -0.12385,-0.11504 -0.33200,-0.01257 -0.30818,0.04530 -0.28012,0.06111 -0.26211,0.07040 -0.12585,0.04702 -0.12123,0.01828 0.02915,0.06014 -0.12235,0.10838
T_delahayei 0.09488,0.08825 0.42856,0.06194 0.37147,0.01617 0.19388,-0.01643 0.11643,-0.00077 0.11022,0.05825 0.16990,-0.15441 0.15987,-0.15811 0.01932,-0.14850 -0.11176,-0.12299 -0.35196,-0.01248 -0.31092,0.04144 -0.27497,0.06064 -0.25267,0.06557 -0.13191,0.04352 -0.12900,0.01205 0.02643,0.05252 -0.12776,0.11333
T_ebenus 0.08739,0.08248 0.42622,0.07985 0.36889,0.02953 0.18961,-0.00589 0.12034,-0.00253 0.10827,0.05575 0.17355,-0.17216 0.15371,-0.17328 0.01753,-0.16064 -0.12266,-0.11810 -0.33976,0.00163 -0.30351,0.05052 -0.27451,0.06206 -0.25469,0.06737 -0.12886,0.03802 -0.13110,0.01174 0.04974,0.05358 -0.14016,0.10006
T_flexicornis 0.08757,0.09471 0.41445,0.07470 0.36231,0.02369 0.18236,-0.02343 0.11657,-0.00739 0.11067,0.05391 0.18415,-0.16422 0.16544,-0.16848 0.02490,-0.16433 -0.11058,-0.12716 -0.34881,0.00560 -0.30813,0.04829 -0.27426,0.06383 -0.26314,0.07025 -0.12249,0.03975 -0.11787,0.01179 0.03949,0.05535 -0.14260,0.11314
T_hemichlorus 0.08892,0.09517 0.42660,0.06345 0.36462,0.01901 0.19111,-0.02003 0.11677,-0.01005 0.10916,0.05615 0.18012,-0.15473 0.16031,-0.15793 0.02646,-0.15437 -0.11325,-0.12077 -0.34804,-0.01122 -0.31014,0.04980 -0.28524,0.06552 -0.25737,0.07420 -0.11759,0.03741 -0.11850,0.01007 0.02374,0.05138 -0.13768,0.10694
T_jossoi 0.08882,0.10186 0.44218,0.06220 0.37704,0.01721 0.19023,-0.01046 0.11170,0.00546 0.11664,0.06098 0.16329,-0.15553 0.13909,-0.15133 -0.01295,-0.14222 -0.14449,-0.10613 -0.34573,-0.01405 -0.31092,0.03340 -0.28172,0.04487 -0.26865,0.05643 -0.11438,0.03278 -0.10472,0.00566 0.07587,0.06142 -0.12129,0.09747
T_katanganus 0.09476,0.10198 0.41481,0.06287 0.36016,0.02162 0.19366,-0.02038 0.11957,-0.00978 0.10778,0.07115 0.18030,-0.16722 0.16383,-0.17230 0.01850,-0.16212 -0.11755,-0.12208 -0.35392,-0.00239 -0.30996,0.03758 -0.26865,0.05600 -0.25101,0.06395 -0.11944,0.04398 -0.12341,0.01605 0.02969,0.06366 -0.13911,0.11742
T_lamtoensis 0.08704,0.09467 0.41457,0.06187 0.35127,0.01685 0.18907,-0.02122 0.11945,-0.00768 0.11452,0.04814 0.18962,-0.15386 0.16752,-0.15449 0.03226,-0.15852 -0.11120,-0.12480 -0.35264,-0.00314 -0.31641,0.05189 -0.29042,0.06544 -0.26774,0.07262 -0.12108,0.03727 -0.10915,0.01130 0.03629,0.05364 -0.13296,0.11000
T_liberianus 0.09958,0.09918 0.44117,0.06923 0.37586,0.01440 0.18755,-0.01374 0.12182,-0.00967 0.11796,0.06828 0.16708,-0.15628 0.14664,-0.15895 0.00519,-0.14623 -0.13572,-0.11178 -0.33714,-0.01284 -0.29353,0.04287 -0.27697,0.05342 -0.26283,0.05636 -0.13309,0.03989 -0.12205,0.00090 0.03024,0.05712 -0.13175,0.10784
T_macroliberianus 0.09047,0.07623 0.43676,0.06658 0.37435,0.02199 0.18438,-0.00528 0.11719,-0.00172 0.10893,0.05058 0.17323,-0.15241 0.15505,-0.15380 0.01173,-0.13674 -0.12086,-0.11317 -0.36145,-0.00808 -0.32563,0.03664 -0.27921,0.06282 -0.26152,0.07309 -0.12215,0.03562 -0.10850,0.01096 0.03131,0.04361 -0.10408,0.09305
T_naevius 0.08981,0.08791 0.42410,0.08307 0.36762,0.02604 0.18108,-0.01028 0.11037,-0.00271 0.11210,0.05071 0.18861,-0.17372 0.16677,-0.17118 0.00437,-0.16085 -0.11837,-0.11885 -0.34375,-0.00501 -0.29464,0.05427 -0.27603,0.06836 -0.26948,0.07106 -0.11992,0.03665 -0.11915,0.01062 0.02366,0.04860 -0.12713,0.10529
T_pendjarius 0.09329,0.10034 0.42365,0.07348 0.36151,0.01734 0.18245,-0.02375 0.11107,-0.00855 0.10494,0.06105 0.18275,-0.16956 0.16641,-0.16621 0.01905,-0.16837 -0.12237,-0.12385 -0.33301,-0.00886 -0.30416,0.04441 -0.28143,0.06072 -0.26632,0.06850 -0.11919,0.04458 -0.11166,0.01688 0.02552,0.06233 -0.13251,0.11951
T_pseudoliberianus 0.08655,0.09613 0.43955,0.07162 0.35639,0.02126 0.18259,-0.01862 0.10945,-0.00952 0.10263,0.06678 0.18243,-0.16096 0.16174,-0.16464 0.01337,-0.16217 -0.12332,-0.11794 -0.34597,-0.00099 -0.29766,0.04955 -0.28118,0.06222 -0.26544,0.06427 -0.12740,0.03563 -0.11150,0.00513 0.04359,0.05966 -0.12582,0.10257
T_rolandoi 0.09670,0.10100 0.43537,0.06016 0.37280,0.01158 0.19214,-0.01663 0.13037,0.00182 0.12410,0.05748 0.15864,-0.15418 0.14714,-0.15386 -0.01629,-0.13774 -0.12494,-0.10577 -0.34796,-0.02149 -0.30820,0.03023 -0.28528,0.04261 -0.25842,0.05944 -0.12151,0.03796 -0.10723,0.01389 0.05492,0.06345 -0.14235,0.11005
T_rougonorum 0.09283,0.09811 0.42624,0.06587 0.36270,0.02054 0.18240,-0.01933 0.11515,-0.00282 0.11337,0.05924 0.18245,-0.16630 0.16191,-0.16691 0.01681,-0.16007 -0.12238,-0.11930 -0.33946,-0.01305 -0.30341,0.04734 -0.28064,0.05764 -0.26021,0.06826 -0.12727,0.04496 -0.11920,0.01250 0.02977,0.06365 -0.13107,0.10967
T_rufobasalis 0.09126,0.08828 0.41965,0.06917 0.36428,0.01947 0.18879,-0.01801 0.11397,-0.00788 0.11411,0.05067 0.18268,-0.15863 0.16643,-0.15879 0.01912,-0.16127 -0.11937,-0.12004 -0.34559,-0.00531 -0.29987,0.04977 -0.27955,0.06065 -0.26922,0.06608 -0.12842,0.04440 -0.11910,0.01804 0.04182,0.05531 -0.14098,0.10809
T_rufopygus 0.08680,0.10046 0.43766,0.07135 0.36932,0.02079 0.19215,-0.01941 0.11165,-0.01121 0.10452,0.06508 0.17396,-0.16069 0.15441,-0.16489 0.00139,-0.15555 -0.13504,-0.12086 -0.33422,-0.00234 -0.30221,0.04061 -0.27539,0.05460 -0.26437,0.05583 -0.12510,0.04026 -0.11259,0.01003 0.04434,0.06709 -0.12727,0.10885
T_rufostillans 0.08937,0.08872 0.42864,0.04973 0.37829,0.00994 0.18784,-0.01547 0.11761,0.00726 0.11374,0.05345 0.18275,-0.14568 0.15391,-0.14890 -0.00321,-0.13709 -0.11868,-0.11178 -0.33773,-0.01116 -0.31208,0.03567 -0.28964,0.05107 -0.28737,0.06133 -0.11041,0.03542 -0.11776,0.01841 0.05376,0.05856 -0.12902,0.10051
T_saadaniensis ? ? ? ? ? ? ? ? ? ? ? ? ? ? ? ? ? ?
T_schaufussi 0.09424,0.09880 0.41488,0.05633 0.36369,0.01461 0.18838,-0.02096 0.12125,-0.01138 0.11582,0.07040 0.17874,-0.15869 0.16786,-0.15693 0.01616,-0.16069 -0.11703,-0.12303 -0.33779,-0.00852 -0.31505,0.04038 -0.28066,0.05928 -0.26670,0.06861 -0.12247,0.04482 -0.11579,0.01713 0.03641,0.06373 -0.14194,0.10614
T_viridiaereus 0.09395,0.10474 0.40789,0.05755 0.36768,0.01205 0.19640,-0.01827 0.11168,0.00142 0.11622,0.06306 0.18457,-0.16410 0.15675,-0.16649 0.02851,-0.15417 -0.11169,-0.12316 -0.34470,-0.01502 -0.30759,0.04837 -0.28019,0.06163 -0.26003,0.06856 -0.12952,0.04190 -0.12536,0.01164 0.03812,0.05997 -0.14268,0.11032
T_zambesianus ? ? ? ? ? ? ? ? ? ? ? ? ? ? ? ? ? ?
T_zavattarii 0.09218,0.10841 0.43856,0.05869 0.35867,0.00496 0.19386,-0.01007 0.12519,-0.00630 0.10467,0.06374 0.17375,-0.16002 0.15682,-0.16159 0.00109,-0.13919 -0.11794,-0.11842 -0.35020,-0.02751 -0.30524,0.04132 -0.29140,0.05272 -0.26397,0.06141 -0.11827,0.04700 -0.10286,0.02109 0.02155,0.05696 -0.11646,0.10681

&[landmark 2D]
E_flavimargo 0.00252,-0.39568 0.00312,-0.22480 0.04921,-0.06014 0.10875,0.10058 0.18096,0.24372 0.24839,0.35522 0.05263,0.34556 -0.08805,0.25189 -0.14735,0.09234 -0.17212,-0.07528 -0.13543,-0.23971 -0.10263,-0.39370
M_pallens 0.07031,-0.47163 0.07332,-0.26929 0.11520,-0.10986 0.16637,0.03439 0.20263,0.18811 0.18764,0.35986 0.00129,0.31560 -0.12389,0.24467 -0.17676,0.14642 -0.17608,0.02116 -0.17249,-0.13334 -0.16754,-0.32609
H_bituberculatus 0.01489,-0.37932 0.08579,-0.23897 0.14989,-0.09827 0.21001,0.04900 0.24051,0.20862 0.21183,0.36772 -0.00225,0.33579 -0.13275,0.21861 -0.20123,0.08643 -0.21601,-0.04994 -0.20389,-0.18418 -0.15679,-0.31549
H_depressus 0.02052,-0.38315 0.06653,-0.24978 0.11893,-0.11161 0.15739,0.03955 0.20047,0.18966 0.22674,0.33003 0.01769,0.36591 -0.12633,0.26786 -0.20617,0.12918 -0.20271,-0.03507 -0.16925,-0.20010 -0.10382,-0.34250
P_nuchicornis -0.01383,-0.45610 0.01456,-0.26995 0.05650,-0.10691 0.08999,0.06070 0.14575,0.22998 0.17553,0.40282 0.05713,0.35255 -0.04698,0.25092 -0.11771,0.12071 -0.13470,-0.02963 -0.12488,-0.19113 -0.10137,-0.36396
O_nigriventris -0.00980,-0.45142 -0.00267,-0.26595 0.04718,-0.10399 0.10284,0.06160 0.15803,0.23024 0.16614,0.40384 0.04942,0.35291 -0.05082,0.25812 -0.09753,0.11720 -0.12940,-0.03715 -0.13347,-0.19812 -0.09993,-0.36728
P_coenobita 0.01716,-0.42505 0.04622,-0.25777 0.08298,-0.10300 0.12127,0.06056 0.19146,0.21743 0.20633,0.37874 0.03431,0.34426 -0.08848,0.24823 -0.14469,0.10531 -0.18486,-0.04039 -0.17282,-0.18858 -0.10887,-0.33973
P_ovatus -0.01230,-0.45275 0.03685,-0.28264 0.04319,-0.11842 0.08398,0.04892 0.14068,0.22056 0.16458,0.39822 0.05271,0.36502 -0.04321,0.26887 -0.10384,0.13090 -0.12777,-0.02760 -0.13569,-0.19292 -0.09918,-0.35817
P_medius -0.00969,-0.43627 0.00496,-0.25216 0.04303,-0.08835 0.09570,0.07525 0.16484,0.23754 0.20254,0.39783 0.05825,0.34930 -0.05737,0.24234 -0.12582,0.10141 -0.15374,-0.04943 -0.12720,-0.20618 -0.09551,-0.37128
O_illyricus -0.01898,-0.42402 0.01515,-0.25193 0.07219,-0.09568 0.12934,0.06238 0.18139,0.22167 0.18442,0.38143 0.03264,0.35767 -0.07368,0.25318 -0.13748,0.11376 -0.14442,-0.04397 -0.13328,-0.20682 -0.10729,-0.36769
T_aequatus 0.00002,-0.44715 0.04678,-0.27985 0.10042,-0.13391 0.13093,0.02681 0.16463,0.20059 0.16149,0.38458 0.02418,0.35950 -0.07518,0.26672 -0.13492,0.13594 -0.16215,-0.01026 -0.14533,-0.16658 -0.11087,-0.33639
T_angolensis -0.01418,-0.43735 0.04323,-0.27693 0.08508,-0.13040 0.13503,0.02249 0.16814,0.19331 0.18971,0.37094 0.02464,0.36380 -0.09672,0.27244 -0.16954,0.14351 -0.16514,-0.00910 -0.11943,-0.17161 -0.08082,-0.34110
T_chrysoderus -0.01524,-0.44346 0.02433,-0.26436 0.06149,-0.10148 0.11513,0.05757 0.16441,0.21956 0.17701,0.38457 0.03657,0.35359 -0.06976,0.26330 -0.11719,0.12451 -0.14266,-0.03144 -0.12516,-0.19566 -0.10894,-0.36670
T_curtipilis -0.00850,-0.43968 0.03436,-0.26426 0.06724,-0.09874 0.11769,0.06023 0.16404,0.21796 0.19214,0.37403 0.03098,0.34446 -0.08759,0.26733 -0.12487,0.13181 -0.13533,-0.02845 -0.13663,-0.19776 -0.11354,-0.36694
T_delahayei -0.01398,-0.41182 0.03207,-0.25331 0.09006,-0.10421 0.14238,0.05616 0.18856,0.22223 0.19307,0.38750 0.02909,0.35081 -0.08637,0.24677 -0.14425,0.10423 -0.16411,-0.04937 -0.15670,-0.20145 -0.10983,-0.34755
T_ebenus -0.02646,-0.41045 0.02468,-0.24309 0.09592,-0.09530 0.16057,0.05849 0.20499,0.21802 0.19023,0.37716 0.01842,0.34767 -0.09960,0.24909 -0.15271,0.10653 -0.16698,-0.04780 -0.13854,-0.20244 -0.11053,-0.35788
T_flexicornis 0.01361,-0.42827 0.01162,-0.25117 0.04288,-0.09061 0.11632,0.05920 0.17680,0.21733 0.21290,0.37202 0.04256,0.35220 -0.08293,0.25917 -0.14733,0.12045 -0.16069,-0.03683 -0.13510,-0.20233 -0.09063,-0.37117
T_hemichlorus -0.01971,-0.42139 0.02216,-0.25691 0.07050,-0.09698 0.10710,0.06824 0.16211,0.22364 0.19972,0.37086 0.03522,0.35815 -0.08048,0.26993 -0.12249,0.12153 -0.13459,-0.04715 -0.13420,-0.21634 -0.10535,-0.37357
T_jossoi -0.03196,-0.38223 0.03283,-0.23070 0.10970,-0.09008 0.17570,0.06167 0.20435,0.22731 0.21077,0.38442 0.01958,0.34772 -0.10652,0.23654 -0.16008,0.08377 -0.18052,-0.07025 -0.15311,-0.21587 -0.12075,-0.35231
T_katanganus -0.05331,-0.40085 0.01487,-0.24161 0.08156,-0.08860 0.15058,0.06648 0.19351,0.22751 0.18764,0.38407 0.02288,0.35850 -0.08823,0.25328 -0.13415,0.09999 -0.14428,-0.06414 -0.12252,-0.22370 -0.10856,-0.37092
T_lamtoensis -0.01877,-0.41427 0.03306,-0.24909 0.07642,-0.09140 0.13721,0.06186 0.18897,0.21860 0.20950,0.37246 0.02954,0.34656 -0.09958,0.25327 -0.15749,0.11391 -0.16106,-0.04358 -0.13400,-0.20545 -0.10380,-0.36289
T_liberianus -0.02194,-0.41160 0.00453,-0.24048 0.07202,-0.08769 0.12616,0.07286 0.19160,0.22847 0.20046,0.38308 0.03814,0.35705 -0.07595,0.24001 -0.15225,0.10253 -0.14878,-0.05536 -0.13239,-0.21683 -0.10160,-0.37204
T_macroliberianus -0.03474,-0.41022 0.02474,-0.25477 0.07508,-0.10016 0.12528,0.06383 0.17776,0.23038 0.19408,0.39369 0.03799,0.35644 -0.07542,0.24783 -0.13609,0.10201 -0.14800,-0.05719 -0.13878,-0.21394 -0.10190,-0.35791
T_naevius -0.03608,-0.40364 0.02306,-0.24957 0.08264,-0.10061 0.13824,0.06023 0.19103,0.22470 0.20303,0.38653 0.03147,0.35098 -0.09424,0.25144 -0.14965,0.10409 -0.16113,-0.05626 -0.13536,-0.21233 -0.09303,-0.35555
T_pendjarius -0.02990,-0.40443 0.00570,-0.23854 0.06410,-0.08442 0.13144,0.06899 0.18972,0.22514 0.20862,0.37621 0.03606,0.35943 -0.08595,0.25063 -0.15473,0.10528 -0.14921,-0.05809 -0.12247,-0.22308 -0.09338,-0.37712
T_pseudoliberianus -0.03629,-0.44269 0.02894,-0.27340 0.08086,-0.11622 0.13714,0.04334 0.17458,0.21315 0.16038,0.39058 0.02646,0.35231 -0.07959,0.26222 -0.13305,0.13129 -0.13541,-0.02216 -0.12411,-0.18567 -0.09993,-0.35274
T_rolandoi -0.04229,-0.39820 0.01863,-0.24696 0.09915,-0.10892 0.14431,0.05300 0.18422,0.22143 0.19504,0.38464 0.02550,0.36265 -0.09180,0.25538 -0.14934,0.10607 -0.15049,-0.05749 -0.13357,-0.21459 -0.09936,-0.35700
T_rougonorum -0.03509,-0.43734 0.01547,-0.26456 0.05916,-0.09912 0.10523,0.06511 0.15765,0.22448 0.17449,0.38379 0.03397,0.36133 -0.06782,0.27081 -0.10403,0.12584 -0.11147,-0.04073 -0.11703,-0.21198 -0.11052,-0.37763
T_rufobasalis -0.01736,-0.41007 0.05707,-0.28777 0.11233,-0.15768 0.14301,0.00906 0.16741,0.19951 0.18104,0.39637 0.02198,0.36974 -0.09250,0.27013 -0.15059,0.12424 -0.17932,-0.03043 -0.15636,-0.17717 -0.08672,-0.30593
T_rufopygus -0.02244,-0.40926 0.01508,-0.23616 0.08659,-0.08303 0.15191,0.06978 0.19787,0.22505 0.19272,0.37686 0.02366,0.35268 -0.08811,0.24379 -0.14786,0.10151 -0.14816,-0.05547 -0.13695,-0.21492 -0.12431,-0.37083
T_rufostillans -0.07089,-0.39808 0.03237,-0.26199 0.09384,-0.10935 0.13239,0.06276 0.17070,0.23946 0.16823,0.41044 0.02941,0.36512 -0.06525,0.24829 -0.10805,0.09191 -0.13055,-0.06960 -0.12563,-0.22260 -0.12657,-0.35636
T_saadaniensis -0.04499,-0.39327 0.01037,-0.24031 0.06175,-0.08529 0.12001,0.07492 0.18216,0.23072 0.21740,0.37502 0.04006,0.36080 -0.08828,0.26046 -0.14340,0.10192 -0.15144,-0.07062 -0.11810,-0.23569 -0.08554,-0.37867
T_schaufussi -0.02354,-0.40413 0.04002,-0.25239 0.11477,-0.11313 0.16189,0.04639 0.19906,0.21592 0.19850,0.38593 0.01819,0.34370 -0.10978,0.24619 -0.16413,0.10736 -0.17135,-0.04475 -0.15484,-0.19429 -0.10880,-0.33680
T_viridiaereus -0.05132,-0.41145 0.00770,-0.23659 0.06157,-0.06874 0.12723,0.09683 0.17197,0.26729 0.17761,0.42810 0.05123,0.34747 -0.04034,0.21520 -0.09008,0.06050 -0.12769,-0.08992 -0.14417,-0.23464 -0.14371,-0.37404
T_zambesianus -0.03931,-0.40609 0.00647,-0.24216 0.05378,-0.08378 0.12532,0.06985 0.17269,0.23429 0.21561,0.38523 0.04503,0.35670 -0.08096,0.24820 -0.14877,0.10272 -0.14425,-0.06150 -0.11887,-0.22638 -0.08675,-0.37707
T_zavattarii -0.05088,-0.38928 0.01550,-0.23438 0.08206,-0.08506 0.14455,0.07563 0.18961,0.24548 0.20706,0.40484 0.03868,0.35158 -0.07908,0.22852 -0.13977,0.07403 -0.16033,-0.08175 -0.13744,-0.22801 -0.10998,-0.36158

&[landmark 2D]
E_flavimargo 0.00399,-0.39043 0.02625,-0.24038 0.06545,-0.08264 0.12489,0.06877 0.16794,0.22717 0.17279,0.38895 0.01283,0.37489 -0.09910,0.25481 -0.15295,0.09566 -0.14845,-0.07267 -0.11380,-0.23498 -0.05983,-0.38914
M_pallens 0.00965,-0.36101 0.06096,-0.22816 0.12258,-0.06973 0.16566,0.09697 0.20587,0.25427 0.16854,0.40115 -0.02838,0.35889 -0.13351,0.22749 -0.11308,0.04386 -0.10158,-0.12915 -0.21344,-0.23620 -0.14327,-0.35839
H_bituberculatus 0.04583,-0.36769 0.07396,-0.22714 0.10587,-0.07649 0.14872,0.06937 0.18964,0.21291 0.22069,0.34732 -0.00253,0.36148 -0.15193,0.25321 -0.20983,0.09656 -0.19886,-0.06938 -0.14890,-0.22704 -0.07265,-0.37310
H_depressus 0.02911,-0.37379 0.05304,-0.24025 0.09070,-0.08779 0.13537,0.06622 0.18069,0.21808 0.18775,0.36953 0.00044,0.37397 -0.12169,0.26075 -0.16865,0.09611 -0.17212,-0.07620 -0.14666,-0.23530 -0.06800,-0.37134
P_nuchicornis ? ? ? ? ? ? ? ? ? ? ? ?
O_nigriventris 0.02944,-0.42915 0.06289,-0.29034 0.08082,-0.13335 0.09912,0.03222 0.13183,0.21462 0.12899,0.41484 0.02658,0.37862 -0.07027,0.26674 -0.14198,0.12459 -0.15113,-0.03344 -0.12317,-0.19096 -0.07312,-0.35438
P_coenobita 0.00397,-0.39414 0.03896,-0.24518 0.05887,-0.08170 0.09585,0.07822 0.14817,0.23665 0.18839,0.39138 0.02717,0.37253 -0.09226,0.25278 -0.14832,0.09244 -0.14760,-0.07618 -0.10895,-0.23730 -0.06424,-0.38950
P_ovatus -0.02283,-0.36731 0.06226,-0.22920 0.13779,-0.08061 0.16405,0.08252 0.18027,0.25711 0.14645,0.42918 0.00172,0.35764 -0.08423,0.20021 -0.15425,0.04924 -0.15112,-0.09867 -0.14228,-0.23519 -0.13783,-0.36493
P_medius 0.03472,-0.41790 0.03515,-0.26378 0.05954,-0.10102 0.09392,0.05994 0.13900,0.22519 0.16891,0.39598 0.02492,0.37056 -0.08654,0.26339 -0.13808,0.11329 -0.14603,-0.05056 -0.12127,-0.21425 -0.06424,-0.38083
O_illyricus -0.02297,-0.39966 0.01007,-0.23822 0.06486,-0.06772 0.13304,0.09355 0.18582,0.24785 0.13516,0.39931 0.00993,0.36487 -0.07426,0.24034 -0.12560,0.08435 -0.12840,-0.08123 -0.11290,-0.24401 -0.07475,-0.39942
T_aequatus -0.00596,-0.40269 0.03340,-0.24858 0.06821,-0.08337 0.11138,0.07725 0.15300,0.24045 0.16268,0.40703 0.02036,0.36278 -0.08995,0.24813 -0.12920,0.09216 -0.13251,-0.07237 -0.10983,-0.23211 -0.08156,-0.38868
T_angolensis -0.00029,-0.37890 0.05409,-0.24025 0.10911,-0.08994 0.15040,0.06528 0.17261,0.22836 0.17790,0.39239 -0.00226,0.36204 -0.12396,0.24646 -0.16179,0.08738 -0.15971,-0.07530 -0.12756,-0.22657 -0.08852,-0.37094
T_chrysoderus 0.01348,-0.40121 0.04725,-0.24212 0.07400,-0.07507 0.12053,0.07887 0.16429,0.23354 0.17451,0.39146 0.01241,0.35802 -0.10379,0.24457 -0.15017,0.09575 -0.13872,-0.06527 -0.12302,-0.22864 -0.09077,-0.38990
T_curtipilis -0.00051,-0.38731 0.05009,-0.23496 0.10152,-0.07639 0.14313,0.08056 0.17591,0.23995 0.16683,0.40034 0.00368,0.35517 -0.10743,0.23306 -0.14855,0.07878 -0.15100,-0.07863 -0.12880,-0.23004 -0.10487,-0.38052
T_delahayei -0.00657,-0.38168 0.04580,-0.24127 0.09711,-0.08566 0.13806,0.07532 0.18104,0.23811 0.18096,0.40292 0.00655,0.35125 -0.12272,0.23870 -0.15928,0.08196 -0.15660,-0.07954 -0.12271,-0.22917 -0.08163,-0.37094
T_ebenus -0.01196,-0.38579 0.01892,-0.23020 0.06369,-0.06976 0.12752,0.08166 0.15535,0.24803 0.18342,0.40973 0.02412,0.36493 -0.09162,0.23166 -0.14972,0.07358 -0.13748,-0.08724 -0.10935,-0.24329 -0.07290,-0.39333
T_flexicornis -0.00845,-0.39754 0.03514,-0.23755 0.06552,-0.06801 0.11812,0.08832 0.15832,0.24681 0.16756,0.40575 0.01898,0.35765 -0.09152,0.23895 -0.12918,0.08313 -0.12772,-0.08011 -0.11166,-0.24066 -0.09509,-0.39673
T_hemichlorus ? ? ? ? ? ? ? ? ? ? ? ?
T_jossoi -0.00727,-0.37034 0.03099,-0.23970 0.09055,-0.09360 0.15212,0.05870 0.17227,0.22738 0.17224,0.39816 0.00295,0.37856 -0.10900,0.24923 -0.16097,0.08394 -0.15469,-0.08437 -0.12534,-0.23728 -0.06384,-0.37068
T_katanganus -0.01366,-0.38057 0.03797,-0.22828 0.09917,-0.06987 0.15097,0.08772 0.18944,0.25007 0.17688,0.41336 0.01019,0.34210 -0.11605,0.21544 -0.16521,0.06737 -0.14951,-0.08553 -0.12525,-0.23364 -0.09494,-0.37817
T_lamtoensis 0.00081,-0.40355 0.03116,-0.23716 0.07329,-0.06861 0.09966,0.09247 0.14818,0.24311 0.16291,0.39364 0.01714,0.36645 -0.08165,0.25017 -0.11520,0.09067 -0.12104,-0.07781 -0.11368,-0.24346 -0.10159,-0.40592

T_liberianus -0.00503,-0.40451 0.02241,-0.23947 0.06208,-0.07393 0.10609,0.08154 0.15160,0.23405 0.16968,0.38760 0.01581,0.36990 -0.09374,0.25959 -0.13132,0.10079 -0.12323,-0.07017 -0.09567,-0.23880 -0.07869,-0.40659
T_macroliberianus -0.00792,-0.40645 0.01994,-0.24630 0.05692,-0.07880 0.10561,0.07944 0.14870,0.23955 0.16442,0.40241 0.02158,0.36601 -0.08821,0.25366 -0.12569,0.09755 -0.11778,-0.07075 -0.10383,-0.23729 -0.07373,-0.39901
T_naevius 0.00373,-0.39260 0.03125,-0.23622 0.06503,-0.06981 0.10987,0.08823 0.15161,0.24388 0.18119,0.39384 0.02052,0.36360 -0.09335,0.24730 -0.13142,0.08627 -0.13499,-0.08236 -0.12218,-0.24487 -0.08127,-0.39726
T_pendjarius 0.00733,-0.38442 0.04264,-0.22728 0.09143,-0.06766 0.15452,0.07925 0.18590,0.23393 0.18163,0.39041 0.00127,0.35037 -0.12285,0.23453 -0.16502,0.08289 -0.15745,-0.07527 -0.13011,-0.23120 -0.08929,-0.38553
T_pseudoliberianus -0.00682,-0.39612 0.03107,-0.24285 0.06802,-0.07840 0.11584,0.07906 0.15990,0.23554 0.17234,0.39335 0.01319,0.36360 -0.10280,0.25610 -0.13264,0.09624 -0.13500,-0.07435 -0.10940,-0.23822 -0.07369,-0.39396
T_rolandoi -0.01263,-0.37557 0.02573,-0.24029 0.07933,-0.08791 0.13282,0.07341 0.16501,0.24601 0.17603,0.41723 0.01724,0.36597 -0.09926,0.23729 -0.13893,0.07000 -0.15238,-0.09375 -0.12368,-0.24030 -0.06929,-0.37209
T_rougonorum 0.02013,-0.39431 0.03949,-0.23629 0.06719,-0.06877 0.10596,0.09197 0.15421,0.24872 0.18241,0.40119 0.02446,0.35858 -0.08755,0.23552 -0.13126,0.07721 -0.14458,-0.08379 -0.13650,-0.23962 -0.09395,-0.39040
T_rufobasalis -0.02368,-0.39223 0.03057,-0.24159 0.08233,-0.08171 0.13035,0.07794 0.17157,0.24009 0.15367,0.40478 0.00916,0.36748 -0.09306,0.24304 -0.13911,0.08461 -0.13361,-0.07946 -0.10267,-0.23540 -0.08553,-0.38755
T_rufopygus 0.01355,-0.38603 0.03190,-0.24065 0.08203,-0.08970 0.13015,0.06604 0.17135,0.22788 0.18548,0.39124 0.01027,0.36595 -0.11457,0.25094 -0.16177,0.09166 -0.16295,-0.07316 -0.12373,-0.22732 -0.06172,-0.37686
T_rufostillans -0.01301,-0.39655 0.03845,-0.24602 0.08494,-0.08614 0.11611,0.07400 0.14278,0.23795 0.16604,0.40020 0.01484,0.37139 -0.09201,0.25109 -0.13554,0.09237 -0.12640,-0.07431 -0.10674,-0.23461 -0.08946,-0.38936
T_saadaniensis -0.00024,-0.39247 0.03420,-0.24250 0.07351,-0.08412 0.11520,0.07274 0.15624,0.23017 0.17134,0.38770 0.01321,0.37525 -0.09603,0.25971 -0.13932,0.09560 -0.14570,-0.07484 -0.11076,-0.23593 -0.07164,-0.39133
T_schaufussi -0.01089,-0.37697 0.03557,-0.23634 0.08874,-0.08762 0.13796,0.06855 0.17119,0.23679 0.19530,0.40312 0.01356,0.36139 -0.12156,0.23776 -0.17446,0.07823 -0.16220,-0.08310 -0.10753,-0.22904 -0.06567,-0.37276
T_viridiaereus 0.00928,-0.38365 0.02189,-0.22582 0.06168,-0.06064 0.12135,0.09634 0.15855,0.25914 0.18250,0.41472 0.02693,0.35780 -0.08215,0.22254 -0.12655,0.05948 -0.14402,-0.09924 -0.13891,-0.24916 -0.09056,-0.39152
T_zambesianus -0.01144,-0.40551 0.03086,-0.24329 0.07688,-0.07825 0.11629,0.08024 0.15584,0.23773 0.16099,0.39840 0.01225,0.36160 -0.09815,0.25154 -0.12928,0.09704 -0.12220,-0.06928 -0.10052,-0.23316 -0.09151,-0.39707
T_zavattarii -0.00081,-0.39973 0.03582,-0.23562 0.08051,-0.07349 0.12138,0.08041 0.16562,0.23318 0.17771,0.38810 0.01049,0.36056 -0.10909,0.24674 -0.15500,0.09467 -0.13597,-0.06795 -0.10259,-0.23037 -0.08810,-0.39648

&[landmark 2D]
E_flavimargo -0.20022,0.07958 -0.13406,0.11170 -0.06262,0.10718 0.00795,0.08285 0.07681,0.10083 0.14055,0.10261 0.18676,0.08603 0.39453,-0.04070 -0.42216,0.01490 -0.29975,0.09558 0.26601,0.07337 -0.26603,-0.04467 -0.21572,-0.06905 -0.16175,-0.07399 -0.10544,-0.07222 -0.05474,-0.07761 0.00581,-0.07745 0.06564,-0.07774 0.11841,-0.07940 0.16629,-0.08597 0.22370,-0.08664 0.27004,-0.06920
M_pallens -0.19929,0.08695 -0.13168,0.12744 -0.05675,0.15206 0.02129,0.15653 0.09285,0.14444 0.15196,0.11361 0.19876,0.09291 0.37923,-0.07421 -0.43401,-0.03749 -0.28801,0.04423 0.24061,0.04414 -0.26117,-0.08716 -0.15967,-0.04143 -0.12722,-0.06686 -0.08640,-0.07894 -0.04444,-0.08655 0.00396,-0.08445 0.04954,-0.08750 0.09551,-0.08511 0.13397,-0.06955 0.16635,-0.05119 0.25461,-0.11184
H_bituberculatus -0.21012,0.11368 -0.14867,0.14847 -0.07334,0.17140 0.00808,0.18164 0.08635,0.16344 0.15747,0.14254 0.21013,0.12227 0.33664,-0.09074 -0.34312,-0.03136 -0.27442,0.08063 0.25433,0.06847 -0.21219,-0.09576 -0.17273,-0.09378 -0.13030,-0.09285 -0.08778,-0.09117 -0.04471,-0.09185 0.00231,-0.09133 0.04506,-0.09580 0.08926,-0.10117 0.12674,-0.10442 0.16967,-0.10663 0.21134,-0.10568
H_depressus -0.20793,0.11339 -0.14124,0.14022 -0.06426,0.15187 0.01706,0.15508 0.09322,0.14656 0.15902,0.12825 0.20966,0.12050 0.34222,-0.06677 -0.37648,-0.01642 -0.28389,0.07863 0.24047,0.07553 -0.22915,-0.06761 -0.18071,-0.08039 -0.13636,-0.09024 -0.09187,-0.09798 -0.04526,-0.10589 0.00388,-0.10607 0.05011,-0.10749 0.09586,-0.10321 0.13934,-0.10170 0.18178,-0.09164 0.22452,-0.07460
P_nuchicornis -0.20782,0.12296 -0.15458,0.16484 -0.08077,0.19187 0.00272,0.20455 0.08450,0.18746 0.15965,0.16183 0.21642,0.11960 0.31678,-0.04476 -0.30967,-0.02173 -0.25829,0.09615 0.29101,0.07669 -0.18927,-0.11159 -0.15518,-0.10803 -0.12035,-0.10708 -0.08136,-0.10718 -0.04408,-0.10807 -0.00599,-0.10983 0.03453,-0.11333 0.07420,-0.11681 0.11048,-0.12098 0.14336,-0.12586 0.17373,-0.13070
O_nigriventris -0.21567,0.09055 -0.15166,0.13874 -0.07298,0.15516 0.01186,0.16132 0.09446,0.15032 0.17309,0.12103 0.24500,0.07154 0.32946,-0.11518 -0.37003,-0.10017 -0.28066,-0.02007 0.27121,-0.02690 -0.26246,-0.09479 -0.22172,-0.05269 -0.16554,-0.04155 -0.10781,-0.03568 -0.05397,-0.05338 -0.00087,-0.06691 0.05121,-0.04806 0.10077,-0.03989 0.15964,-0.04283 0.21813,-0.05511 0.24854,-0.09545
P_coenobita -0.23868,0.11790 -0.19105,0.17889 -0.10256,0.20689 0.00585,0.21593 0.11216,0.20816 0.20020,0.17189 0.25588,0.10523 0.29773,-0.06066 -0.29273,-0.06433 -0.26959,0.07167 0.32343,0.04323 -0.15993,-0.12348 -0.12927,-0.11269 -0.10008,-0.10124 -0.06798,-0.09775 -0.03676,-0.09591 -0.00554,-0.09636 0.02558,-0.09904 0.05339,-0.10473 0.07960,-0.11118 0.10723,-0.12012 0.13312,-0.13231
P_ovatus -0.23237,0.08488 -0.17167,0.13672 -0.08762,0.17449 0.01002,0.18531 0.10315,0.17013 0.17661,0.12725 0.21976,0.06774 0.35163,-0.09282 -0.34983,-0.07833 -0.29145,0.05617 0.30458,0.02608 -0.20532,-0.09324 -0.16868,-0.07314 -0.12725,-0.06645 -0.08505,-0.06745 -0.04717,-0.06721 -0.00615,-0.07037 0.04041,-0.07158 0.07601,-0.07334 0.12360,-0.07464 0.16627,-0.08664 0.20054,-0.11356
P_medius -0.19365,0.11490 -0.14097,0.15292 -0.07189,0.17534 0.00712,0.18195 0.08488,0.17189 0.15309,0.14693 0.20305,0.10533 0.35128,-0.04050 -0.32206,-0.02335 -0.25522,0.09221 0.28562,0.07269 -0.21104,-0.10717 -0.17902,-0.10238 -0.14148,-0.10058 -0.10079,-0.09950 -0.05692,-0.10145 -0.01235,-0.10028 0.03231,-0.10219 0.07619,-0.10489 0.12087,-0.10836 0.16392,-0.10957 0.20704,-0.11393
O_illyricus -0.20665,0.12663 -0.14321,0.15992 -0.07011,0.17090 0.00922,0.17035 0.08771,0.16691 0.16345,0.15361 0.23107,0.10659 0.31582,-0.10334 -0.34059,-0.08846 -0.26251,0.00498 0.24814,-0.00159 -0.24094,-0.10175 -0.20210,-0.07474 -0.15503,-0.06250 -0.10559,-0.06150 -0.05504,-0.07107 -0.00289,-0.07581 0.04884,-0.07741 0.09668,-0.07262 0.14808,-0.06932 0.19700,-0.08432 0.23865,-0.11550
T_aequatus -0.19197,0.13567 -0.13303,0.17377 -0.06461,0.19391 0.00956,0.19695 0.08093,0.18920 0.14482,0.16423 0.19773,0.12313 0.31088,-0.08372 -0.33636,-0.06337 -0.25603,0.06885 0.24581,0.04747 -0.21415,-0.11050 -0.17028,-0.09915 -0.12906,-0.09247 -0.08486,-0.09674 -0.04471,-0.09840 -0.00462,-0.10125 0.04512,-0.10283 0.08617,-0.09914 0.12865,-0.10212 0.17015,-0.11164 0.20985,-0.13186
T_angolensis -0.18733,0.12268 -0.12778,0.16272 -0.05821,0.17659 0.01566,0.17896 0.08591,0.17142 0.14795,0.15034 0.19536,0.10882 0.33848,-0.08538 -0.35667,-0.06698 -0.25793,0.04399 0.24785,0.03810 -0.23887,-0.09390 -0.19405,-0.08481 -0.14394,-0.08795 -0.09332,-0.08440 -0.04716,-0.08785 -0.00167,-0.09293 0.04148,-0.09162 0.08737,-0.09109 0.13585,-0.09358 0.18457,-0.09272 0.22645,-0.10039
T_chrysoderus -0.22147,0.13633 -0.15645,0.16732 -0.07959,0.18968 0.00379,0.20070 0.08702,0.19050 0.16924,0.16789 0.24677,0.12791 0.31625,-0.12122 -0.32084,-0.10110 -0.26975,0.05205 0.29289,0.03741 -0.18457,-0.10984 -0.15054,-0.09930 -0.11660,-0.09070 -0.07708,-0.08430 -0.03861,-0.07977 -0.00314,-0.07883 0.03583,-0.08277 0.06904,-0.08830 0.09923,-0.09745 0.13295,-0.11059 0.16562,-0.12562
T_curtipilis -0.22426,0.13242 -0.16075,0.16462 -0.08198,0.18146 0.00489,0.18966 0.09061,0.18467 0.17055,0.15715 0.24261,0.11399 0.34478,-0.12266 -0.32520,-0.10286 -0.27384,0.05323 0.30272,0.03581 -0.17999,-0.08674 -0.15040,-0.08765 -0.11749,-0.08659 -0.08354,-0.08773 -0.04534,-0.09015 -0.00707,-0.09147 0.02912,-0.09053 0.06636,-0.08964 0.09598,-0.09066 0.13312,-0.09196 0.16913,-0.09438
T_delahayei -0.19264,0.12601 -0.13054,0.14298 -0.06473,0.15346 0.00442,0.15793 0.07311,0.14962 0.14281,0.13521 0.21313,0.10960 0.33534,-0.10155 -0.35523,-0.07688 -0.25968,0.03328 0.25305,0.02925 -0.25414,-0.09360 -0.21345,-0.06978 -0.16152,-0.06430 -0.11289,-0.06716 -0.06104,-0.06823 -0.00211,-0.07135 0.05494,-0.07178 0.10745,-0.07215 0.16189,-0.07130 0.21147,-0.08712 0.25037,-0.12216
T_ebenus -0.19583,0.15586 -0.13678,0.19214 -0.06572,0.20765 0.01051,0.21401 0.08441,0.20519 0.15188,0.18121 0.20675,0.13815 0.31592,-0.09508 -0.31306,-0.07281 -0.24877,0.06901 0.25601,0.05850 -0.18557,-0.10063 -0.15122,-0.11102 -0.11301,-0.11376 -0.07623,-0.11718 -0.04146,-0.11700 -0.00528,-0.11757 0.02879,-0.11732 0.06569,-0.11718 0.10114,-0.11772 0.13669,-0.11472 0.17515,-0.10974
T_flexicornis -0.19704,0.11065 -0.13938,0.16162 -0.06617,0.18587 0.01483,0.19170 0.09267,0.17916 0.16015,0.14824 0.20876,0.09935 0.33752,-0.12341 -0.33924,-0.09742 -0.25935,0.02600 0.25404,0.01761 -0.22938,-0.10967 -0.19317,-0.08988 -0.15196,-0.07688 -0.10278,-0.06923 -0.05602,-0.07036 -0.00332,-0.07015 0.04277,-0.06975 0.08810,-0.06934 0.13475,-0.07485 0.18120,-0.08845 0.22301,-0.11082
T_hemichlorus -0.24342,0.16389 -0.18283,0.21009 -0.09746,0.23972 0.00150,0.24916 0.09932,0.23812 0.18604,0.19988 0.25552,0.14170 0.27927,-0.11746 -0.25494,-0.09604 -0.26080,0.07200 0.31093,0.05256 -0.12107,-0.12830 -0.10036,-0.12402 -0.07717,-0.12115 -0.05456,-0.11837 -0.02966,-0.11685 -0.00549,-0.11679 0.01694,-0.11802 0.03828,-0.12085 0.05871,-0.12454 0.07984,-0.12850 0.10140,-0.13622
T_jossoi -0.18691,0.17718 -0.13216,0.20114 -0.07093,0.21850 -0.00519,0.22676 0.06154,0.21822 0.13006,0.19771 0.19899,0.15019 0.30900,-0.08742 -0.29250,-0.06539 -0.23985,0.11893 0.27960,0.07639 -0.16096,-0.11578 -0.13373,-0.12867 -0.09990,-0.12971 -0.06716,-0.12637 -0.03476,-0.12826 -0.00235,-0.13157 0.02944,-0.13255 0.06232,-0.13873 0.09251,-0.14042 0.11853,-0.13759 0.14444,-0.12257
T_katanganus -0.19483,0.11713 -0.13030,0.14769 -0.06258,0.16693 0.00902,0.16776 0.07870,0.15721 0.14468,0.13986 0.20144,0.09978 0.33470,-0.08416 -0.35581,-0.05733 -0.26396,0.03150 0.24756,0.02087 -0.25443,-0.09385 -0.21321,-0.06878 -0.15902,-0.06513 -0.10477,-0.07128 -0.05091,-0.08453 0.00235,-0.08959 0.05661,-0.08597 0.10825,-0.08004 0.16021,-0.07796 0.20199,-0.08297 0.24434,-0.10712
T_lamtoensis -0.22792,0.15395 -0.16987,0.19437 -0.09140,0.22158 -0.00049,0.22933 0.09061,0.22007 0.17491,0.19351 0.24484,0.14805 0.28729,-0.10958 -0.27011,-0.06869 -0.25627,0.08211 0.29535,0.06265 -0.14801,-0.14104 -0.11978,-0.12677 -0.09595,-0.11693 -0.06422,-0.10826 -0.03360,-0.10665 -0.00637,-0.10576 0.02507,-0.10834 0.05549,-0.11352 0.07798,-0.12199 0.10212,-0.13150 0.13035,-0.14658
T_liberianus -0.18216,0.14063 -0.12860,0.17562 -0.06486,0.18551 0.00435,0.18890 0.07264,0.18029 0.13820,0.16255 0.19651,0.12708 0.32965,-0.05956 -0.33585,-0.03869 -0.24633,0.06960 0.24609,0.05333 -0.21627,-0.08004 -0.17417,-0.09314 -0.13481,-0.10238 -0.09346,-0.10995 -0.05319,-0.11563 -0.00363,-0.12020 0.04196,-0.12076 0.08389,-0.12103 0.12812,-0.11538 0.17554,-0.10816 0.21639,-0.09861
T_macroliberianus -0.18758,0.12801 -0.13108,0.16033 -0.06923,0.17934 -0.00309,0.18479 0.06258,0.17385 0.12776,0.15002 0.19254,0.10918 0.32637,-0.07991 -0.33691,-0.04000 -0.25182,0.05966 0.26011,0.03838 -0.23350,-0.09996 -0.19210,-0.08676 -0.14959,-0.08304 -0.09854,-0.08859 -0.04787,-0.09118 0.00087,-0.09318 0.05102,-0.09671 0.10384,-0.09491 0.15401,-0.09747 0.19552,-0.10611 0.22668,-0.12575
T_naevius -0.19219,0.15186 -0.13162,0.17208 -0.06765,0.19272 0.00127,0.19883 0.06999,0.18812 0.13805,0.16913 0.20167,0.13143 0.32218,-0.05397 -0.31371,-0.02050 -0.24718,0.07057 0.25258,0.05430 -0.20607,-0.08665 -0.17348,-0.10708 -0.13284,-0.11202 -0.09031,-0.11767 -0.04833,-0.12017 -0.00115,-0.11840 0.04467,-0.11972 0.08419,-0.12023 0.12233,-0.12450 0.16439,-0.12386 0.20320,-0.10425
T_pendjarius -0.18985,0.11229 -0.12378,0.13987 -0.05656,0.15855 0.01363,0.16121 0.08158,0.15134 0.14556,0.13423 0.19997,0.09698 0.33607,-0.11511 -0.36148,-0.09637 -0.26263,0.02004 0.23676,0.01337 -0.26187,-0.10080 -0.22318,-0.07601 -0.17677,-0.05891 -0.12323,-0.04867 -0.06683,-0.05113 -0.00846,-0.05687 0.05869,-0.05358 0.11894,-0.05863 0.17058,-0.06545 0.22763,-0.08699 0.26524,-0.11935
T_pseudoliberianus -0.16981,0.13492 -0.11654,0.17117 -0.05715,0.18604 0.00555,0.19083 0.06747,0.18192 0.12800,0.16130 0.18487,0.12017 0.34237,-0.06597 -0.33780,-0.05446 -0.24015,0.06683 0.26070,0.05859 -0.22821,-0.09881 -0.18729,-0.09466 -0.13923,-0.09843 -0.09371,-0.09867 -0.05039,-0.10155 -0.01054,-0.10562 0.03638,-0.10524 0.08524,-0.10701 0.12931,-0.10855 0.17324,-0.11284 0.21769,-0.11994
T_rolandoi -0.18353,0.14385 -0.12082,0.16819 -0.05534,0.18182 0.01174,0.18562 0.07560,0.17975 0.13491,0.16069 0.18735,0.12361 0.31716,-0.09353 -0.34617,-0.06134 -0.24858,0.04675 0.22874,0.04334 -0.24104,-0.10925 -0.19154,-0.09746 -0.14544,-0.08885 -0.09512,-0.08481 -0.04461,-0.08955 0.00160,-0.09301 0.04746,-0.09152 0.09877,-0.08970 0.14701,-0.09652 0.18956,-0.10860 0.23230,-0.12950
T_rougonorum -0.22398,0.12755 -0.15845,0.16537 -0.07487,0.18322 0.01472,0.19671 0.10068,0.18621 0.17644,0.16111 0.23062,0.11497 0.32796,-0.10095 -0.33437,-0.09113 -0.27822,0.05756 0.28274,0.04241 -0.18583,-0.08728 -0.15137,-0.08860 -0.11489,-0.08911 -0.07845,-0.09062 -0.04216,-0.09410 -0.00417,-0.10160 0.03456,-0.09836 0.06904,-0.09974 0.10261,-0.10008 0.13630,-0.09849 0.17109,-0.09506
T_rufobasalis -0.18268,0.14801 -0.12842,0.18471 -0.06374,0.20183 0.00624,0.20957 0.07454,0.20053 0.13779,0.17919 0.18988,0.13897 0.31629,-0.06591 -0.31159,-0.03456 -0.23967,0.07401 0.23899,0.06421 -0.19716,-0.07898 -0.16565,-0.10770 -0.13381,-0.12293 -0.09458,-0.12254 -0.04796,-0.12187 -0.00385,-0.12374 0.03821,-0.12875 0.07969,-0.13371 0.12576,-0.13805 0.16327,-0.12168 0.19843,-0.10061
T_rufopygus -0.18444,0.15638 -0.13052,0.19294 -0.06922,0.21836 0.00085,0.21770 0.07080,0.21408 0.13325,0.18262 0.19291,0.13580 0.30970,-0.05665 -0.30185,-0.03179 -0.23901,0.09927 0.26435,0.07193 -0.18276,-0.09526 -0.15115,-0.11341 -0.11558,-0.12619 -0.07677,-0.13419 -0.04148,-0.14063 -0.00158,-0.14301 0.03557,-0.14300 0.06847,-0.14131 0.10661,-0.13567 0.14089,-0.12197 0.17096,-0.10602
T_rufostillans -0.19182,0.12770 -0.13266,0.16629 -0.06484,0.18709 0.00988,0.18616 0.08322,0.17902 0.14957,0.15853 0.20207,0.11297 0.34120,-0.06356 -0.34222,-0.04273 -0.25665,0.05969 0.26120,0.04415 -0.22051,-0.09276 -0.17726,-0.09243 -0.13473,-0.09405 -0.08511,-0.09856 -0.04297,-0.11064 -0.00261,-0.11806 0.03895,-0.11116 0.07669,-0.10149 0.12068,-0.09926 0.15995,-0.10077 0.20795,-0.09610
T_saadaniensis -0.20955,0.21412 -0.15738,0.23460 -0.08973,0.24852 -0.01153,0.25152 0.07058,0.24683 0.15575,0.23142 0.24114,0.18410 0.26683,-0.11967 -0.23571,-0.09495 -0.22764,0.10374 0.28492,0.07178 -0.11596,-0.13451 -0.09683,-0.13708 -0.07699,-0.14089 -0.05502,-0.14277 -0.03261,-0.14510 -0.00594,-0.14635 0.01733,-0.14710 0.03817,-0.14614 0.05896,-0.14479 0.08014,-0.14377 0.10108,-0.14349
T_schaufussi -0.18289,0.12227 -0.12675,0.15879 -0.06217,0.17188 0.00746,0.17419 0.07547,0.16906 0.13972,0.15315 0.19651,0.11614 0.31800,-0.08252 -0.34338,-0.05440 -0.25180,0.04929 0.23437,0.03122 -0.24998,-0.11461 -0.20881,-0.08991 -0.15833,-0.07647 -0.10460,-0.07327 -0.05304,-0.07777 0.00069,-0.08351 0.05146,-0.08345 0.10666,-0.08313 0.16146,-0.08892 0.20481,-0.10791 0.24514,-0.13013
T_viridiaereus -0.21276,0.15681 -0.15215,0.19370 -0.07759,0.22394 0.00586,0.23604 0.08789,0.22528 0.16310,0.19183 0.23123,0.14962 0.28749,-0.08671 -0.28915,-0.06898 -0.25637,0.10097 0.29254,0.08050 -0.14834,-0.11618 -0.12010,-0.12347 -0.09031,-0.12624 -0.06038,-0.12910 -0.03278,-0.12956 -0.00258,-0.12890 0.02746,-0.13053 0.05385,-0.13076 0.07423,-0.12949 0.09690,-0.12969 0.12196,-0.12910
T_zambesianus -0.22194,0.14957 -0.16377,0.18931 -0.08699,0.20582 -0.00111,0.21457 0.08554,0.20362 0.17005,0.17924 0.24519,0.13661 0.32256,-0.16104 -0.30288,-0.13165 -0.26430,0.06070 0.28955,0.04036 -0.15255,-0.10842 -0.12723,-0.10191 -0.09825,-0.09507 -0.07224,-0.09096 -0.04502,-0.08990 -0.01054,-0.08857 0.01960,-0.08759 0.05131,-0.09462 0.08890,-0.09921 0.12317,-0.11011 0.15095,-0.12076
T_zavattarii -0.19277,0.14200 -0.13137,0.15863 -0.06574,0.17124 0.00393,0.17433 0.07322,0.16884 0.14231,0.15423 0.21001,0.12315 0.32791,-0.09348 -0.33867,-0.06657 -0.25468,0.05514 0.25098,0.03947 -0.23059,-0.10504 -0.19004,-0.09027 -0.14596,-0.07990 -0.09596,-0.08039 -0.05572,-0.08631 -0.00067,-0.09285 0.04740,-0.08901 0.09417,-0.09043 0.14051,-0.08971 0.18520,-0.10270 0.22652,-0.12036

&[numeric]
E_flavimargo 0 0 0 0 1 0 2 0 1 0 0 0 0 0 0 0 0 0 0 - 0 1 1 1 - - 0 0 0 0 0 0 - 0 0 0 0 0 0 0 0 0 - 0 0 0 0 - 0 1 0 0 0
M_pallens 0 1 1 0 1 0 0 0 0 0 0 0 0 1 0 1 0 1 0 - 0 0 0 2 - - 0 1 - 1 0 - - 1 0 0 1 3 1 0 0 0 - 1 0 0 - - 0 0 1 0 0
H_bituberculatus 0 0 1 0 1 0 0 0 0 0 0 1 0 1 0 1 0 1 0 - 0 0 0 3 - - 0 1 - 1 0 - - 1 0 0 2 2 1 0 0 0 - 2 1 0 1 1 0 0 1 6 0
H_depressus 0 0 1 0 1 0 0 0 0 0 0 1 0 1 0 1 0 1 0 - 0 0 0 3 - - 0 1 - 1 0 - - 1 0 0 2 2 1 0 0 0 - 2 1 0 1 1 0 0 1 6 0
P_nuchicornis 0 0 0 0 0 0 0 0 0 0 1 0 0 0 0 0 0 0 0 - 0 0 1 0 0 1 ? ? ? ? ? ? ? ? ? ? 2 1 1 ? 0 1 - 2 1 0 2 0 0 0 0 0 0
O_nigriventris 0 0 0 0 0 1 0 0 1 1 0 0 0 0 0 0 0 0 0 - 0 0 1 0 0 0 0 0 1 0 0 0 - 0 1 2 1 0 1 1 0 0 - 0 0 0 0 - 0 0 2 1 0
P_coenobita 0 0 0 0 0 0 0 0 0 0 1 0 0 0 0 0 0 0 0 - 0 0 1 0 0 1 1 0 1 0 0 1 - 0 2 0 2 1 1 0 0 1 - 2 1 0 2 0 0 0 0 0 0
P_ovatus 0 0 0 0 0 0 0 0 0 0 1 0 0 0 0 0 0 0 0 - 0 0 1 0 0 1 1 0 1 0 0 1 - 0 0 0 2 1 1 0 0 1 - 2 1 0 2 0 0 0 0 0 0
P_medius 0 0 0 0 0 0 0 0 0 0 1 0 0 0 0 0 0 0 0 - 0 0 1 0 0 1 1 0 1 0 0 1 - 0 2 0 2 1 1 0 0 1 - 2 1 0 2 0 0 0 0 0 0
O_illyricus 0 0 1 0 0 1 0 0 1 1 0 0 0 0 0 0 0 0 0 - 0 0 1 0 0 0 0 0 1 0 0 0 - 0 4 2 2 4 1 0 0 1 - 0 0 0 0 - 0 0 2 1 0
T_aequatus 1 1 0 0 1 1 2 1 1 1 1 0 1 1 1 1 1 3 2 0 1 1 1 0 0 2 0 0 2 0 1 - 0 0 2 1 2 3 2 2 1 2 0 2 1 1 2 2 1 2 0 4 1
T_angolensis 1 0 0 0 1 1 2 1 1 1 1 0 1 1 1 1 1 2 2 0 1 1 1 0 0 2 0 0 2 0 1 - 0 0 2 1 2 3 2 2 1 2 0 2 1 1 2 2 2 2 0 4 1
T_chrysoderus 2 0 0 1 1 1 1 1 1 0 1 0 1 1 2 0 1 0 2 0 2 1 1 0 1 3 0 0 2 0 1 - 4 0 2 1 2 5 2 2 1 2 0 2 1 0 2 3 2 2 0 4 0
T_curtipilis 2 0 0 1 1 1 1 1 1 1 1 0 1 1 2 0 1 0 1 0 2 1 1 0 0 4 2 0 2 0 1 - 5 0 2 1 2 5 2 2 1 2 3 2 1 0 2 3 2 2 0 2 0
T_delahayei 1 0 0 0 1 1 2 1 1 1 1 0 1 1 1 0 1 3 2 2 1 1 1 0 0 4 1 0 2 0 1 - 2 0 ? ? 2 4 2 2 1 2 0 2 1 1 2 3 3 2 2 4 0
T_ebenus 1 1 0 0 1 0 2 1 1 1 1 0 1 1 1 0 1 3 2 0 1 1 1 0 0 2 2 0 2 0 1 - 3 0 2 1 2 3 2 2 1 2 2 2 1 1 2 3 2 2 2 3 0
T_flexicornis 2 0 0 1 1 1 1 1 1 1 1 0 1 1 2 0 1 0 1 0 0 1 1 0 0 2 1 0 2 0 1 - 4 0 2 1 2 4 2 2 1 2 4 2 1 0 2 2 2 2 0 2 0
T_hemichlorus 2 0 0 1 1 0 1 1 1 0 1 0 1 1 2 0 1 0 1 0 0 1 1 0 1 3 ? ? ? ? ? - ? ? ? ? 2 5 2 ? 1 2 0 2 1 1 2 3 2 2 0 2 1
T_jossoi 1 1 2 1 0 1 2 1 1 2 1 0 1 1 4 0 1 3 2 1 1 1 1 0 0 5 1 0 2 2 0 - - 0 2 1 2 5 2 0 1 3 - 2 1 0 2 2 2 2 0 5 0
T_katanganus 2 1 0 0 1 1 2 1 1 1 1 0 1 1 3 0 1 0 1 0 1 1 1 0 1 3 0 0 2 0 1 - 4 0 2 1 2 4 2 2 1 2 3 2 1 0 2 3 3 2 2 4 1
T_lamtoensis 2 0 0 1 1 0 1 1 1 0 1 0 1 1 2 0 1 0 1 0 0 1 1 0 1 3 0 0 2 0 1 - 4 0 2 1 2 5 2 2 1 2 0 2 1 0 2 3 2 2 0 2 0
T_liberianus 1 1 2 1 0 1 2 1 1 2 1 0 1 1 4 0 1 3 2 1 1 1 1 0 0 5 2 0 2 0 0 2 - 0 2 1 2 3 2 2 1 2 0 2 1 1 2 2 1 2 2 3 0
T_macroliberianus 1 1 2 1 1 1 2 1 1 3 1 0 1 1 4 0 1 3 2 1 1 1 1 1 0 0 5 2 0 2 0 0 2 - 0 2 1 2 3 2 2 1 2 0 2 1 1 2 2 1 2 2 3 0
T_naevius 1 1 0 0 1 0 2 1 1 1 1 0 1 1 1 0 1 3 1 0 1 1 1 0 0 2 1 0 2 0 1 - 2 0 2 1 2 3 2 2 1 2 0 2 1 1 2 3 3 2 2 4 0
T_pendjarius 2 0 0 1 1 0 1 1 1 1 1 0 1 1 2 0 1 0 1 0 0 1 1 0 1 3 1 0 2 0 1 - 3 0 2 1 2 6 2 2 1 2 0 2 1 0 2 3 3 2 0 2 0
T_pseudoliberianus 1 1 2 1 0 1 2 1 1 2 1 0 1 1 4 0 1 3 2 1 1 1 1 1 0 0 4 0 0 2 0 0 2 - 0 2 1 2 3 2 2 1 2 0 2 1 1 2 2 1 2 2 3 0
T_rolandoi 1 0 0 0 1 1 2 1 1 1 1 0 1 1 1 1 1 2 1 0 1 1 1 0 0 4 0 0 2 0 1 - 1 0 2 1 2 4 2 2 1 2 1 2 1 1 2 2 2 2 0 4 1
T_rougonorum 2 0 0 1 1 1 1 1 1 1 1 0 1 1 2 0 1 0 1 0 2 1 1 0 0 3 0 0 2 0 1 - 5 0 2 1 2 4 2 2 1 2 4 2 1 0 2 2 3 2 0 2 0
T_rufobasalis 1 1 0 0 1 0 2 1 1 1 1 0 1 1 3 0 1 3 1 0 1 1 1 0 0 2 0 0 2 0 1 - 3 0 2 1 2 3 2 2 1 2 2 2 1 1 2 2 2 2 0 4 0
T_rufopygus 1 1 2 1 0 1 2 1 1 2 1 0 1 1 4 0 1 3 2 1 1 1 1 0 0 4 0 0 2 0 0 2 - 0 3 1 2 3 2 0 1 2 4 2 1 0 2 2 2 2 2 3 0
T_rufostillans 1 0 0 0 1 1 2 1 1 1 1 0 1 1 1 1 1 3 2 0 1 1 1 0 0 2 1 0 2 0 1 - 0 0 2 1 2 3 2 2 1 2 0 2 1 1 2 2 2 2 0 4 1
T_saadaniensis 2 0 2 1 0 1 2 1 1 3 1 0 1 1 3 1 0 2 2 1 1 1 1 0 0 5 1 0 2 0 0 3 - 0 ? 1 2 5 2 0 1 3 - 2 1 0 2 3 2 2 0 5 0
T_schaufussi 1 0 0 0 1 0 2 1 1 1 1 0 1 1 1 1 1 2 1 0 1 1 1 0 0 4 1 0 2 0 1 - 1 0 2 1 2 4 2 2 1 2 1 2 1 1 2 2 2 2 0 4 1
T_viridiaereus 2 0 2 1 1 1 1 1 1 1 1 0 1 1 2 0 1 0 1 0 0 1 1 0 1 3 1 0 2 0 1 - 4 0 2 1 2 ? 2 2 1 2 3 2 1 0 2 2 2 2 0 2 0
T_zambesianus 1 1 0 0 1 0 2 1 1 1 1 0 1 1 1 0 1 2 1 0 1 1 1 0 0 4 1 0 2 0 1 - 2 0 2 1 2 3 2 2 1 2 0 2 1 1 2 3 2 2 2 4 0
T_zavattarii 1 0 0 0 1 1 2 1 1 1 1 0 1 1 3 1 1 2 1 0 1 1 1 0 0 4 0 0 2 0 1 - 1 0 2 1 2 4 2 2 1 2 1 2 1 1 2 2 2 2 0 4 1
