# Supplementary material for: The Phylogenetic Relationships of Tiaronthophagus n.gen. (Coleoptera, Scarabaeidae, Onthophagini) Evaluated by Phenotypic Characters
Source: Insects. 2019 Mar 1;10(3):64. doi: 10.3390/insects10030064 (PMC6468411; doi:10.3390/insects10030064)
Supplement: Supplementary file 1 [file insects-10-00064-s001.zip › Roggero Data S3 Common Synapomorphies.pdf]

APPENDIX S3
List of the synapomorphies common to the tree

```
E. flavimargo:
      No autapomorphies

M. pallens:
      Char. 0 - landmark
      Char. 1 - landmark
      Char. 2 - landmark
      Char. 3 - landmark
      Char. 4 - landmark
      Char. 5 - landmark
      Char. 6 - landmark
      Char. 7 - landmark
      Char. 9 - landmark
      Char. 10 - landmark
      Char. 11 - landmark
      Char. 13: 0 --> 1
      Char. 48: 2 --> 1
      Char. 55: 2 --> 1
      Char. 56: 1 --> 0

H. bituberculatus:
      Char. 0 - landmark
      Char. 1 - landmark
      Char. 2 - landmark
      Char. 3 - landmark
      Char. 4 - landmark
      Char. 5 - landmark
      Char. 6 - landmark
      Char. 7 - landmark
      Char. 8 - landmark
      Char. 9 - landmark
      Char. 10 - landmark
      Char. 11 - landmark

H. depressus:
      Char. 0 - landmark
      Char. 1 - landmark
      Char. 2 - landmark
      Char. 3 - landmark
      Char. 4 - landmark
      Char. 5 - landmark
      Char. 6 - landmark
      Char. 7 - landmark
      Char. 8 - landmark
      Char. 9 - landmark
      Char. 10 - landmark
      Char. 11 - landmark

P. nuchicornis:
      Char. 0 - landmark
      Char. 1 - landmark
      Char. 3 - landmark
      Char. 4 - landmark
      Char. 8 - landmark
      Char. 9 - landmark
      Char. 11 - landmark

O. nigriventris:
      Char. 0 - landmark
      Char. 1 - landmark
      Char. 2 - landmark
      Char. 3 - landmark
      Char. 4 - landmark
      Char. 5 - landmark
      Char. 6 - landmark
      Char. 7 - landmark
      Char. 8 - landmark
      Char. 9 - landmark
      Char. 10 - landmark
      Char. 11 - landmark
      Char. 48: 2 --> 1
      Char. 51: 0 --> 1

P. coenobita:
      Char. 0 - landmark
      Char. 1 - landmark
      Char. 2 - landmark
      Char. 3 - landmark
      Char. 4 - landmark
      Char. 5 - landmark
      Char. 6 - landmark
      Char. 7 - landmark
      Char. 8 - landmark
      Char. 9 - landmark
      Char. 10 - landmark
      Char. 11 - landmark

P. ovatus:
      Char. 0 - landmark
      Char. 1 - landmark
      Char. 2 - landmark
      Char. 3 - landmark
      Char. 4 - landmark
      Char. 5 - landmark
      Char. 6 - landmark
      Char. 7 - landmark
      Char. 8 - landmark
      Char. 9 - landmark
      Char. 10 - landmark
      Char. 11 - landmark

P. medius:
      Char. 0 - landmark
      Char. 1 - landmark
      Char. 2 - landmark
      Char. 3 - landmark
      Char. 4 - landmark
      Char. 5 - landmark
      Char. 6 - landmark
      Char. 7 - landmark
      Char. 8 - landmark
      Char. 9 - landmark
      Char. 10 - landmark
      Char. 11 - landmark
```

O. illyricus:
Char. 0 - landmark
Char. 1 - landmark
Char. 2 - landmark
Char. 3 - landmark
Char. 4 - landmark
Char. 5 - landmark
Char. 6 - landmark
Char. 7 - landmark
Char. 8 - landmark
Char. 9 - landmark
Char. 10 - landmark
Char. 11 - landmark
Char. 14: 0 --> 1
Char. 49: 0 --> 4
Char. 53: 0 --> 1

T. aequatus:
Char. 0 - landmark
Char. 1 - landmark
Char. 2 - landmark
Char. 3 - landmark
Char. 4 - landmark
Char. 5 - landmark
Char. 6 - landmark
Char. 7 - landmark
Char. 9 - landmark
Char. 10 - landmark
Char. 11 - landmark
Char. 60: 2 --> 1

T. angolensis:
Char. 0 - landmark
Char. 1 - landmark
Char. 2 - landmark
Char. 3 - landmark
Char. 4 - landmark
Char. 5 - landmark
Char. 6 - landmark
Char. 7 - landmark
Char. 8 - landmark
Char. 9 - landmark
Char. 10 - landmark
Char. 11 - landmark

T. chrysoderus:
Char. 0 - landmark
Char. 1 - landmark
Char. 2 - landmark
Char. 3 - landmark
Char. 4 - landmark
Char. 5 - landmark
Char. 6 - landmark
Char. 7 - landmark
Char. 8 - landmark
Char. 9 - landmark
Char. 10 - landmark
Char. 11 - landmark
Char. 30: 1 --> 2
Char. 32: 0 --> 2
Char. 63: 2 --> 4

T. curtipilis:
Char. 0 - landmark
Char. 1 - landmark
Char. 2 - landmark
Char. 3 - landmark
Char. 4 - landmark
Char. 5 - landmark
Char. 6 - landmark
Char. 7 - landmark
Char. 8 - landmark
Char. 9 - landmark
Char. 10 - landmark
Char. 11 - landmark
Char. 37: 3 --> 4
Char. 49: 4 --> 5
Char. 59: 2 --> 3

T. delahayei:
Char. 0 - landmark
Char. 1 - landmark
Char. 2 - landmark
Char. 3 - landmark
Char. 4 - landmark
Char. 5 - landmark
Char. 6 - landmark
Char. 7 - landmark
Char. 8 - landmark
Char. 9 - landmark
Char. 10 - landmark
Char. 11 - landmark
Char. 31: 0 --> 2

T. ebenus:
Char. 0 - landmark
Char. 1 - landmark
Char. 2 - landmark
Char. 3 - landmark
Char. 4 - landmark
Char. 5 - landmark
Char. 6 - landmark
Char. 7 - landmark
Char. 8 - landmark
Char. 9 - landmark
Char. 10 - landmark
Char. 11 - landmark
Char. 30: 1 --> 2
Char. 63: 4 --> 3

T. flexicornis:
Char. 0 - landmark
Char. 1 - landmark
Char. 2 - landmark
Char. 3 - landmark
Char. 4 - landmark
Char. 5 - landmark
Char. 6 - landmark
Char. 7 - landmark
Char. 8 - landmark
Char. 9 - landmark
Char. 10 - landmark
Char. 11 - landmark
Char. 37: 3 --> 2

T. hemichlorus:
Char. 0 - landmark
Char. 1 - landmark
Char. 3 - landmark
Char. 4 - landmark
Char. 8 - landmark
Char. 9 - landmark
Char. 11 - landmark
Char. 57: 0 --> 1
Char. 64: 0 --> 1

T. katanganus:
Char. 0 - landmark
Char. 1 - landmark
Char. 2 - landmark
Char. 3 - landmark
Char. 4 - landmark
Char. 5 - landmark
Char. 6 - landmark
Char. 7 - landmark
Char. 8 - landmark
Char. 9 - landmark
Char. 10 - landmark
Char. 11 - landmark
Char. 38: 1 --> 0
Char. 54: 0 --> 3
Char. 64: 0 --> 1

T. lamtoensis:
Char. 0 - landmark
Char. 1 - landmark
Char. 2 - landmark
Char. 3 - landmark
Char. 4 - landmark
Char. 5 - landmark
Char. 6 - landmark
Char. 7 - landmark
Char. 8 - landmark
Char. 9 - landmark
Char. 10 - landmark
Char. 11 - landmark

T. liberianus:
Char. 0 - landmark
Char. 1 - landmark
Char. 2 - landmark
Char. 3 - landmark
Char. 4 - landmark
Char. 5 - landmark
Char. 6 - landmark
Char. 7 - landmark
Char. 8 - landmark
Char. 9 - landmark
Char. 10 - landmark
Char. 11 - landmark

T. macroliberianus:
Char. 0 - landmark
Char. 1 - landmark
Char. 2 - landmark
Char. 3 - landmark
Char. 4 - landmark
Char. 5 - landmark
Char. 6 - landmark
Char. 7 - landmark
Char. 8 - landmark
Char. 9 - landmark
Char. 10 - landmark
Char. 11 - landmark
Char. 16: 0 --> 1
Char. 21: 2 --> 3

T. naevius:
Char. 0 - landmark
Char. 1 - landmark
Char. 2 - landmark
Char. 3 - landmark
Char. 4 - landmark
Char. 5 - landmark
Char. 6 - landmark
Char. 7 - landmark
Char. 8 - landmark
Char. 9 - landmark
Char. 10 - landmark
Char. 11 - landmark
Char. 60: 2 --> 3

T. pendjarius:
Char. 0 - landmark
Char. 1 - landmark
Char. 2 - landmark
Char. 3 - landmark
Char. 4 - landmark
Char. 5 - landmark
Char. 6 - landmark
Char. 7 - landmark
Char. 8 - landmark
Char. 9 - landmark
Char. 10 - landmark
Char. 11 - landmark
Char. 17: 1 --> 0
Char. 44: 4 --> 3
Char. 49: 4 --> 6

T. pseudoliberianus:
Char. 0 - landmark
Char. 1 - landmark
Char. 2 - landmark
Char. 3 - landmark
Char. 4 - landmark
Char. 5 - landmark
Char. 6 - landmark
Char. 7 - landmark
Char. 8 - landmark
Char. 9 - landmark
Char. 10 - landmark
Char. 11 - landmark

T. rougonorum:
Char. 0 - landmark
Char. 1 - landmark
Char. 2 - landmark
Char. 3 - landmark
Char. 4 - landmark
Char. 5 - landmark
Char. 6 - landmark

Char. 7 - landmark
Char. 8 - landmark
Char. 9 - landmark
Char. 10 - landmark
Char. 11 - landmark
Char. 60: 2 --> 3

T. rufobasalis:
Char. 0 - landmark
Char. 1 - landmark
Char. 2 - landmark
Char. 3 - landmark
Char. 4 - landmark
Char. 5 - landmark
Char. 6 - landmark
Char. 7 - landmark
Char. 8 - landmark
Char. 9 - landmark
Char. 10 - landmark
Char. 11 - landmark
Char. 26: 1 --> 3
Char. 59: 3 --> 2
Char. 62: 2 --> 0

T. rufopygus:
Char. 0 - landmark
Char. 1 - landmark
Char. 2 - landmark
Char. 3 - landmark
Char. 4 - landmark
Char. 5 - landmark
Char. 6 - landmark
Char. 7 - landmark
Char. 8 - landmark
Char. 9 - landmark
Char. 10 - landmark
Char. 11 - landmark
Char. 46: 2 --> 3

T. rufostillans:
Char. 0 - landmark
Char. 1 - landmark
Char. 2 - landmark
Char. 3 - landmark
Char. 4 - landmark
Char. 5 - landmark
Char. 6 - landmark
Char. 7 - landmark
Char. 8 - landmark
Char. 9 - landmark
Char. 10 - landmark
Char. 11 - landmark
Char. 38: 0 --> 1

T. schaufussi:
Char. 0 - landmark
Char. 1 - landmark
Char. 2 - landmark
Char. 3 - landmark
Char. 4 - landmark
Char. 5 - landmark
Char. 6 - landmark
Char. 7 - landmark
Char. 8 - landmark
Char. 9 - landmark
Char. 10 - landmark
Char. 11 - landmark
Char. 17: 1 --> 0
Char. 38: 0 --> 1

T. viridiaereus:
Char. 0 - landmark
Char. 1 - landmark
Char. 2 - landmark
Char. 3 - landmark
Char. 4 - landmark
Char. 5 - landmark
Char. 6 - landmark
Char. 7 - landmark
Char. 8 - landmark
Char. 9 - landmark
Char. 10 - landmark
Char. 11 - landmark
Char. 14: 0 --> 2

T. zambesianus:
Char. 0 - landmark
Char. 1 - landmark
Char. 2 - landmark
Char. 3 - landmark
Char. 4 - landmark
Char. 5 - landmark
Char. 6 - landmark
Char. 7 - landmark
Char. 9 - landmark
Char. 10 - landmark
Char. 11 - landmark
Char. 29: 3 --> 2

T. zavattarii:
Char. 0 - landmark
Char. 1 - landmark
Char. 2 - landmark
Char. 3 - landmark
Char. 4 - landmark
Char. 5 - landmark
Char. 6 - landmark
Char. 7 - landmark
Char. 8 - landmark
Char. 9 - landmark
Char. 10 - landmark
Char. 11 - landmark
Char. 26: 1 --> 3

T. rolandoi:
Char. 0 - landmark
Char. 1 - landmark
Char. 2 - landmark
Char. 3 - landmark
Char. 4 - landmark
Char. 5 - landmark
Char. 6 - landmark
Char. 7 - landmark
Char. 8 - landmark
Char. 9 - landmark
Char. 10 - landmark

```
        Char. 11 - landmark

T. jossoi:
        Char. 0 - landmark
        Char. 1 - landmark
        Char. 2 - landmark
        Char. 3 - landmark
        Char. 4 - landmark
        Char. 5 - landmark
        Char. 6 - landmark
        Char. 7 - landmark
        Char. 8 - landmark
        Char. 9 - landmark
        Char. 10 - landmark
        Char. 11 - landmark
        Char. 41: 0 --> 2

T. saadaniensis:
        Char. 0 - landmark
        Char. 1 - landmark
        Char. 2 - landmark
        Char. 3 - landmark
        Char. 4 - landmark
        Char. 5 - landmark
        Char. 6 - landmark
        Char. 7 - landmark
        Char. 9 - landmark
        Char. 10 - landmark
        Char. 11 - landmark
        Char. 12: 1 --> 2
        Char. 13: 1 --> 0
        Char. 21: 2 --> 3
        Char. 26: 4 --> 3
        Char. 27: 0 --> 1
        Char. 28: 1 --> 0
        Char. 29: 3 --> 2
        Char. 59: 2 --> 3

   Node 37:
        Char. 0 - landmark
        Char. 1 - landmark
        Char. 2 - landmark
        Char. 3 - landmark
        Char. 4 - landmark
        Char. 5 - landmark
        Char. 6 - landmark
        Char. 7 - landmark
        Char. 8 - landmark
        Char. 9 - landmark
        Char. 10 - landmark
        Char. 11 - landmark
        Char. 14: 0 --> 1
        Char. 27: 0 --> 1
        Char. 29: 0 --> 1
        Char. 34: 1 --> 0
        Char. 39: 0 --> 1
        Char. 41: 0 --> 1
        Char. 45: 0 --> 1
        Char. 62: 0 --> 1

   Node 38:
        Char. 0 - landmark
        Char. 1 - landmark
        Char. 2 - landmark
        Char. 3 - landmark
        Char. 4 - landmark
        Char. 5 - landmark
        Char. 6 - landmark
        Char. 7 - landmark
        Char. 8 - landmark
        Char. 9 - landmark
        Char. 10 - landmark
        Char. 25: 0 --> 1

    Node 39:
        Char. 0 - landmark
        Char. 1 - landmark
        Char. 2 - landmark
        Char. 3 - landmark
        Char. 4 - landmark
        Char. 5 - landmark
        Char. 6 - landmark
        Char. 7 - landmark
        Char. 8 - landmark
        Char. 9 - landmark
        Char. 10 - landmark
        Char. 11 - landmark
        Char. 55: 0 --> 2
        Char. 56: 0 --> 1
        Char. 58: 0 --> 2

    Node 40:
        Char. 0 - landmark
        Char. 1 - landmark
        Char. 2 - landmark
        Char. 3 - landmark
        Char. 4 - landmark
        Char. 5 - landmark
        Char. 6 - landmark
        Char. 7 - landmark
        Char. 8 - landmark
        Char. 9 - landmark
        Char. 10 - landmark
        Char. 11 - landmark

    Node 41:
        Char. 0 - landmark
        Char. 1 - landmark
        Char. 2 - landmark
        Char. 3 - landmark
        Char. 4 - landmark
        Char. 5 - landmark
        Char. 6 - landmark
        Char. 7 - landmark
        Char. 8 - landmark
        Char. 9 - landmark
        Char. 10 - landmark
        Char. 11 - landmark
        Char. 23: 0 --> 1
        Char. 63: 0 --> 6

    Node 42:
        Char. 0 - landmark
        Char. 1 - landmark
        Char. 2 - landmark
        Char. 3 - landmark
```

```
    Char. 4 - landmark
    Char. 5 - landmark
    Char. 6 - landmark
    Char. 7 - landmark
    Char. 8 - landmark
    Char. 9 - landmark
    Char. 10 - landmark
    Char. 11 - landmark

Node 43:
    Char. 0 - landmark
    Char. 1 - landmark
    Char. 2 - landmark
    Char. 3 - landmark
    Char. 4 - landmark
    Char. 5 - landmark
    Char. 6 - landmark
    Char. 7 - landmark
    Char. 8 - landmark
    Char. 9 - landmark
    Char. 11 - landmark
    Char. 38: 0 --> 1
    Char. 53: 0 --> 1

Node 44:
    Char. 0 - landmark
    Char. 1 - landmark
    Char. 2 - landmark
    Char. 3 - landmark
    Char. 4 - landmark
    Char. 5 - landmark
    Char. 6 - landmark
    Char. 7 - landmark
    Char. 8 - landmark
    Char. 9 - landmark
    Char. 10 - landmark
    Char. 11 - landmark
    Char. 17: 0 --> 1
    Char. 21: 0 --> 1
    Char. 47: 0 --> 2
    Char. 62: 0 --> 2
    Char. 63: 0 --> 1

Node 45:
    Char. 0 - landmark
    Char. 1 - landmark
    Char. 2 - landmark
    Char. 3 - landmark
    Char. 4 - landmark
    Char. 5 - landmark
    Char. 6 - landmark
    Char. 7 - landmark
    Char. 8 - landmark
    Char. 9 - landmark
    Char. 10 - landmark
    Char. 11 - landmark
    Char. 46: 0 --> 2

Node 46:
    Char. 0 - landmark
    Char. 1 - landmark
    Char. 2 - landmark
    Char. 3 - landmark
    Char. 4 - landmark
    Char. 5 - landmark
    Char. 6 - landmark
    Char. 7 - landmark
    Char. 8 - landmark
    Char. 9 - landmark
    Char. 10 - landmark
    Char. 11 - landmark
    Char. 27: 0 --> 1
    Char. 37: 4 --> 2
    Char. 62: 2 --> 0
    Char. 64: 0 --> 1

Node 47:
    Char. 0 - landmark
    Char. 1 - landmark
    Char. 2 - landmark
    Char. 3 - landmark
    Char. 4 - landmark
    Char. 5 - landmark
    Char. 6 - landmark
    Char. 7 - landmark
    Char. 8 - landmark
    Char. 9 - landmark
    Char. 10 - landmark
    Char. 38: 1 --> 0
    Char. 59: 3 --> 2

Node 48:
    Char. 0 - landmark
    Char. 1 - landmark
    Char. 2 - landmark
    Char. 3 - landmark
    Char. 4 - landmark
    Char. 5 - landmark
    Char. 6 - landmark
    Char. 7 - landmark
    Char. 8 - landmark
    Char. 10 - landmark
    Char. 11 - landmark
    Char. 49: 4 --> 3
    Char. 60: 3 --> 2

Node 49:
    Char. 0 - landmark
    Char. 1 - landmark
    Char. 2 - landmark
    Char. 3 - landmark
    Char. 4 - landmark
    Char. 5 - landmark
    Char. 6 - landmark
    Char. 7 - landmark
    Char. 8 - landmark
    Char. 9 - landmark
    Char. 10 - landmark
    Char. 11 - landmark
    Char. 12: 2 --> 1
    Char. 29: 0 --> 3
    Char. 36: 1 --> 0
    Char. 37: 3 --> 4
    Char. 44: 4 --> 2
    Char. 57: 0 --> 1
```

```
Node 50:
    Char. 0 - landmark
    Char. 1 - landmark
    Char. 2 - landmark
    Char. 3 - landmark
    Char. 4 - landmark
    Char. 5 - landmark
    Char. 6 - landmark
    Char. 7 - landmark
    Char. 8 - landmark
    Char. 9 - landmark
    Char. 10 - landmark
    Char. 11 - landmark
    Char. 15: 1 --> 0
    Char. 18: 1 --> 2
    Char. 32: 0 --> 1
    Char. 62: 0 --> 2
    Char. 63: 2 --> 4

Node 51:
    Char. 0 - landmark
    Char. 1 - landmark
    Char. 2 - landmark
    Char. 3 - landmark
    Char. 4 - landmark
    Char. 5 - landmark
    Char. 6 - landmark
    Char. 7 - landmark
    Char. 8 - landmark
    Char. 9 - landmark
    Char. 10 - landmark
    Char. 11 - landmark
    Char. 60: 2 --> 3

Node 52:
    Char. 0 - landmark
    Char. 1 - landmark
    Char. 2 - landmark
    Char. 3 - landmark
    Char. 4 - landmark
    Char. 5 - landmark
    Char. 6 - landmark
    Char. 7 - landmark
    Char. 8 - landmark
    Char. 9 - landmark
    Char. 10 - landmark
    Char. 11 - landmark
    Char. 21: 0 --> 1
    Char. 38: 0 --> 1
    Char. 49: 5 --> 4

Node 53:
    Char. 0 - landmark
    Char. 1 - landmark
    Char. 2 - landmark
    Char. 3 - landmark
    Char. 4 - landmark
    Char. 5 - landmark
    Char. 6 - landmark
    Char. 7 - landmark
    Char. 8 - landmark
    Char. 9 - landmark
    Char. 10 - landmark
    Char. 11 - landmark
    Char. 17: 0 --> 1

Node 54:
    Char. 0 - landmark
    Char. 1 - landmark
    Char. 2 - landmark
    Char. 3 - landmark
    Char. 4 - landmark
    Char. 5 - landmark
    Char. 6 - landmark
    Char. 7 - landmark
    Char. 8 - landmark
    Char. 9 - landmark
    Char. 10 - landmark
    Char. 11 - landmark
    Char. 12: 0 --> 2
    Char. 15: 0 --> 1
    Char. 18: 0 --> 1
    Char. 19: 0 --> 1
    Char. 24: 0 --> 1
    Char. 26: 0 --> 2
    Char. 28: 0 --> 1
    Char. 30: 0 --> 1
    Char. 33: 0 --> 1
    Char. 42: 0 --> 1
    Char. 46: 0 --> 2
    Char. 47: 0 --> 1
    Char. 50: 1 --> 2
    Char. 51: 0 --> 2
    Char. 52: 0 --> 1
    Char. 53: 0 --> 2
    Char. 60: 0 --> 2
    Char. 61: 0 --> 2
    Char. 63: 0 --> 2

Node 55:
    Char. 0 - landmark
    Char. 1 - landmark
    Char. 2 - landmark
    Char. 3 - landmark
    Char. 4 - landmark
    Char. 5 - landmark
    Char. 6 - landmark
    Char. 7 - landmark
    Char. 8 - landmark
    Char. 9 - landmark
    Char. 10 - landmark
    Char. 11 - landmark
    Char. 29: 3 --> 2

Node 56:
    Char. 0 - landmark
    Char. 1 - landmark
    Char. 2 - landmark
    Char. 3 - landmark
    Char. 4 - landmark
    Char. 5 - landmark
    Char. 6 - landmark
    Char. 7 - landmark
    Char. 8 - landmark
    Char. 9 - landmark
    Char. 10 - landmark
```

```
    Char. 11 - landmark
    Char. 13: 1 --> 0

Node 57:
    Char. 0 - landmark
    Char. 1 - landmark
    Char. 2 - landmark
    Char. 3 - landmark
    Char. 4 - landmark
    Char. 5 - landmark
    Char. 6 - landmark
    Char. 7 - landmark
    Char. 8 - landmark
    Char. 9 - landmark
    Char. 10 - landmark
    Char. 11 - landmark
    Char. 32: 0 --> 2
    Char. 44: 4 --> 5

Node 58:
    Char. 0 - landmark
    Char. 1 - landmark
    Char. 2 - landmark
    Char. 3 - landmark
    Char. 4 - landmark
    Char. 5 - landmark
    Char. 6 - landmark
    Char. 7 - landmark
    Char. 8 - landmark
    Char. 9 - landmark
    Char. 10 - landmark
    Char. 11 - landmark
    Char. 36: 1 --> 0

Node 59:
    Char. 0 - landmark
    Char. 1 - landmark
    Char. 2 - landmark
    Char. 3 - landmark
    Char. 4 - landmark
    Char. 5 - landmark
    Char. 6 - landmark
    Char. 7 - landmark
    Char. 8 - landmark
    Char. 10 - landmark
    Char. 11 - landmark
    Char. 59: 3 --> 2

Node 60:
    Char. 0 - landmark
    Char. 1 - landmark
    Char. 2 - landmark
    Char. 3 - landmark
    Char. 4 - landmark
    Char. 5 - landmark
    Char. 6 - landmark
    Char. 7 - landmark
    Char. 8 - landmark
    Char. 9 - landmark
    Char. 10 - landmark
    Char. 11 - landmark
    Char. 44: 2 --> 3
    Char. 54: 0 --> 2

Node 61:
    Char. 0 - landmark
    Char. 1 - landmark
    Char. 2 - landmark
    Char. 3 - landmark
    Char. 4 - landmark
    Char. 5 - landmark
    Char. 6 - landmark
    Char. 7 - landmark
    Char. 8 - landmark
    Char. 9 - landmark
    Char. 10 - landmark
    Char. 11 - landmark
    Char. 37: 4 --> 2

Node 62:
    Char. 0 - landmark
    Char. 1 - landmark
    Char. 2 - landmark
    Char. 3 - landmark
    Char. 4 - landmark
    Char. 5 - landmark
    Char. 6 - landmark
    Char. 7 - landmark
    Char. 8 - landmark
    Char. 9 - landmark
    Char. 10 - landmark
    Char. 11 - landmark
    Char. 17: 1 --> 0

Node 63:
    Char. 0 - landmark
    Char. 1 - landmark
    Char. 2 - landmark
    Char. 3 - landmark
    Char. 4 - landmark
    Char. 5 - landmark
    Char. 6 - landmark
    Char. 7 - landmark
    Char. 8 - landmark
    Char. 9 - landmark
    Char. 10 - landmark
    Char. 11 - landmark

Node 64:
    Char. 0 - landmark
    Char. 1 - landmark
    Char. 2 - landmark
    Char. 3 - landmark
    Char. 4 - landmark
    Char. 5 - landmark
    Char. 6 - landmark
    Char. 7 - landmark
    Char. 8 - landmark
    Char. 9 - landmark
    Char. 10 - landmark
    Char. 11 - landmark
    Char. 37: 4 --> 5
    Char. 38: 0 --> 2

Node 65:
    Char. 0 - landmark
```

```
    Char. 1 - landmark
    Char. 2 - landmark
    Char. 3 - landmark
    Char. 4 - landmark
    Char. 5 - landmark
    Char. 6 - landmark
    Char. 7 - landmark
    Char. 8 - landmark
    Char. 9 - landmark
    Char. 10 - landmark
    Char. 11 - landmark
    Char. 60: 2 --> 1

Node 66:
    Char. 0 - landmark
    Char. 1 - landmark
    Char. 2 - landmark
    Char. 3 - landmark
    Char. 4 - landmark
    Char. 5 - landmark
    Char. 6 - landmark
    Char. 7 - landmark
    Char. 8 - landmark
    Char. 9 - landmark
    Char. 10 - landmark
    Char. 11 - landmark
    Char. 14: 0 --> 2
    Char. 15: 0 --> 1
    Char. 16: 1 --> 0
    Char. 21: 1 --> 2
    Char. 26: 1 --> 4
    Char. 31: 0 --> 1
    Char. 42: 1 --> 0
    Char. 63: 4 --> 3

Node 67:
    Char. 0 - landmark
    Char. 1 - landmark
    Char. 2 - landmark
    Char. 3 - landmark
    Char. 4 - landmark
    Char. 5 - landmark
    Char. 6 - landmark
    Char. 7 - landmark
    Char. 8 - landmark
    Char. 9 - landmark
    Char. 10 - landmark
    Char. 11 - landmark
    Char. 51: 2 --> 0
    Char. 57: 1 --> 0

Node 68:
    Char. 0 - landmark
    Char. 1 - landmark
    Char. 2 - landmark
    Char. 3 - landmark
    Char. 4 - landmark
    Char. 5 - landmark
    Char. 6 - landmark
    Char. 7 - landmark
    Char. 8 - landmark
    Char. 9 - landmark
    Char. 10 - landmark
    Char. 11 - landmark

Node 69:
    Char. 0 - landmark
    Char. 1 - landmark
    Char. 2 - landmark
    Char. 3 - landmark
    Char. 4 - landmark
    Char. 5 - landmark
    Char. 6 - landmark
    Char. 7 - landmark
    Char. 8 - landmark
    Char. 9 - landmark
    Char. 10 - landmark
    Char. 11 - landmark
    Char. 30: 2 --> 1
    Char. 37: 2 --> 4
    Char. 44: 0 --> 1
    Char. 49: 3 --> 4
    Char. 54: 0 --> 1
```
