# Supplementary material for: The Phylogenetic Relationships of Tiaronthophagus n.gen. (Coleoptera, Scarabaeidae, Onthophagini) Evaluated by Phenotypic Characters
Source: Insects. 2019 Mar 1;10(3):64. doi: 10.3390/insects10030064 (PMC6468411; doi:10.3390/insects10030064)

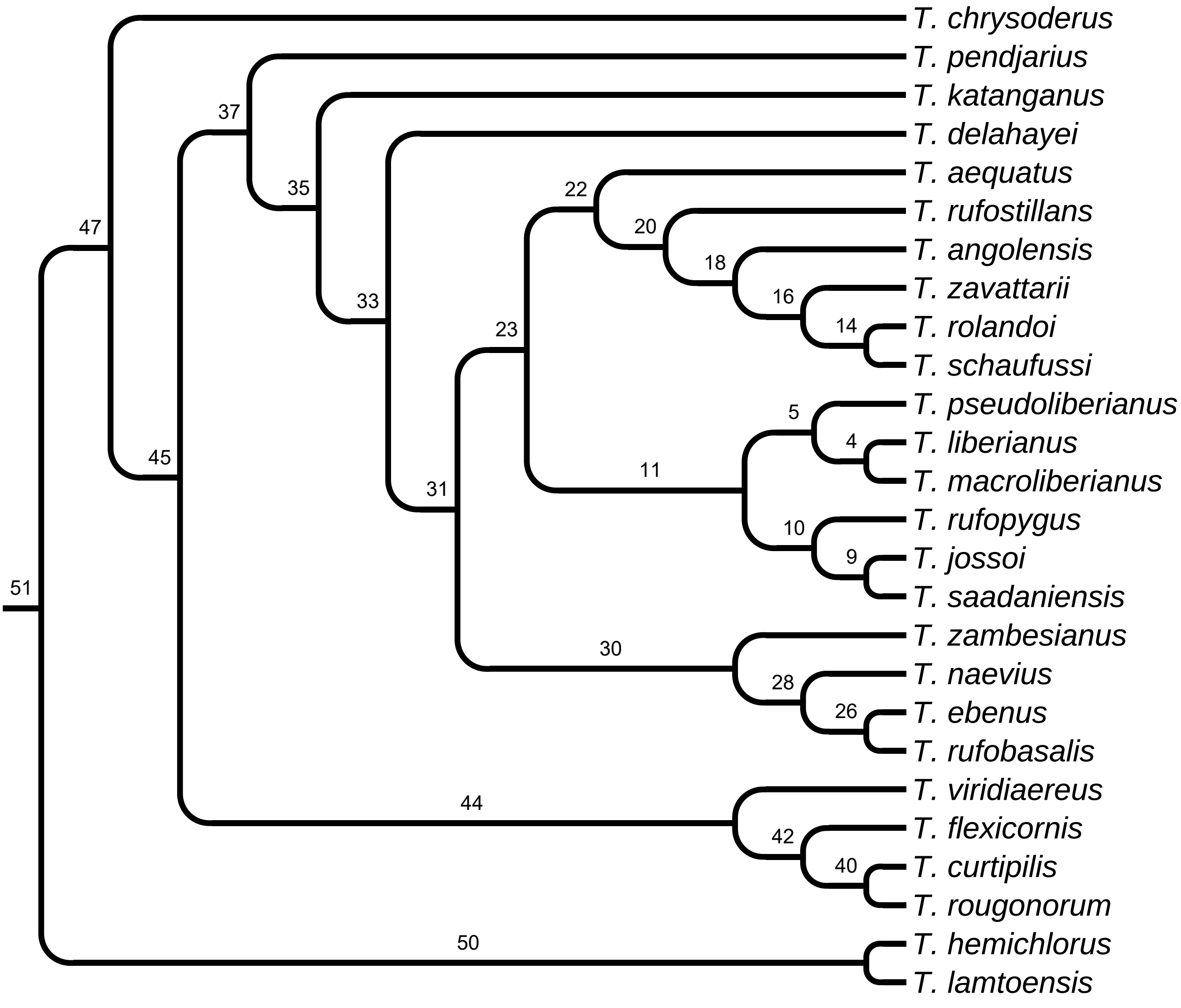
T. chrysoderus
T. pendjarius
T. katanganus
T. delahayei
T. aequatus
T. rufostillans
T. angolensis
T. zavattarii
T. rolandoi
T. schaufussi
T. pseudoliberianus
T. liberianus
T. macroliberianus
T. rufopygus
T. jossoi
T. saadaniensis
T. zambesianus
T. naevius
T. ebenus
T. rufobasalis
T. viridiaereus
T. flexicornis
T. curtipilis
T. rougonorum
T. hemichlorus
T. lamtoensis
51
47
45
37
35
33
31
23
22
20
18
16
14
11
5
4
10
9
30
28
26
44
42
40
50

OR reconstruction

node_1 [pseudoliberianus]
node_2 [liberianus]
node_3 [macroliberianus]
node_4
node_5
node_6 [rufopygus]
node_7 [jossoi]
node_8 [saadaniensis]
node_9
node_10
node_11
node_12 [schaufussi]
node_13 [rolandoi]
node_14
node_15 [zavattarii]
node_16
node_17 [angolensis]
node_18
node_19 [rufostillans]
node_20
node_21 [aequatus]
node_22
node_23
node_24 [rufobasalis]
node_25 [ebenus]
node_26
node_27 [naevius]
node_28
node_29 [zambesianus]
node_30
node_31
node_32 [delahayei]
node_33
node_34 [katanganus]
node_35
node_36 [pendjarius]
node_37
node_38 [rougonorum]
node_39 [curtipilis]
node_40
node_41 [flexicornis]
node_42
node_43 [viridiaereus]
node_44
node_45
node_46 [chrysoderus]
node_47
node_48 [lamtoensis]
node_49 [hemichlorus]
node_50
node_51

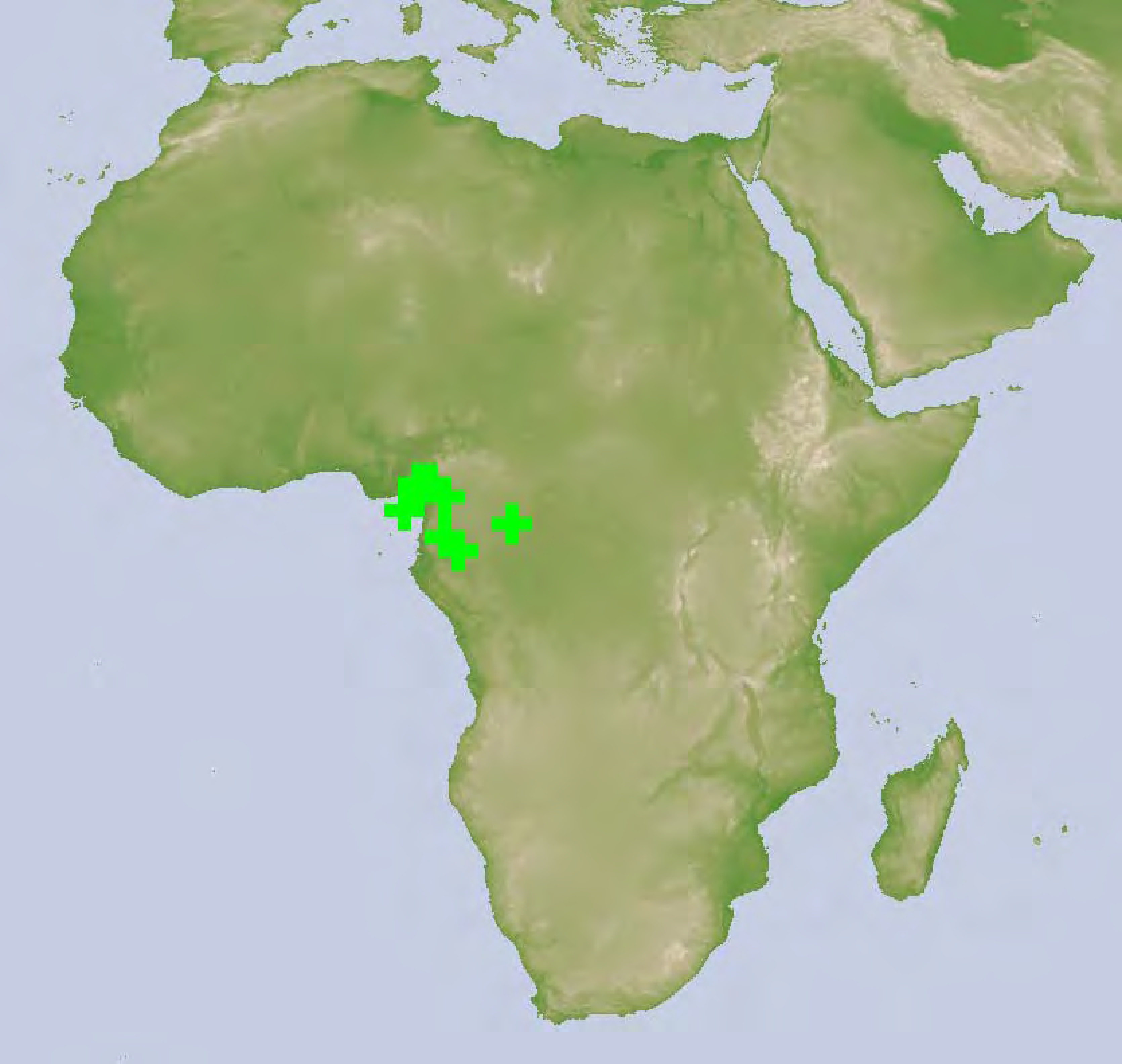

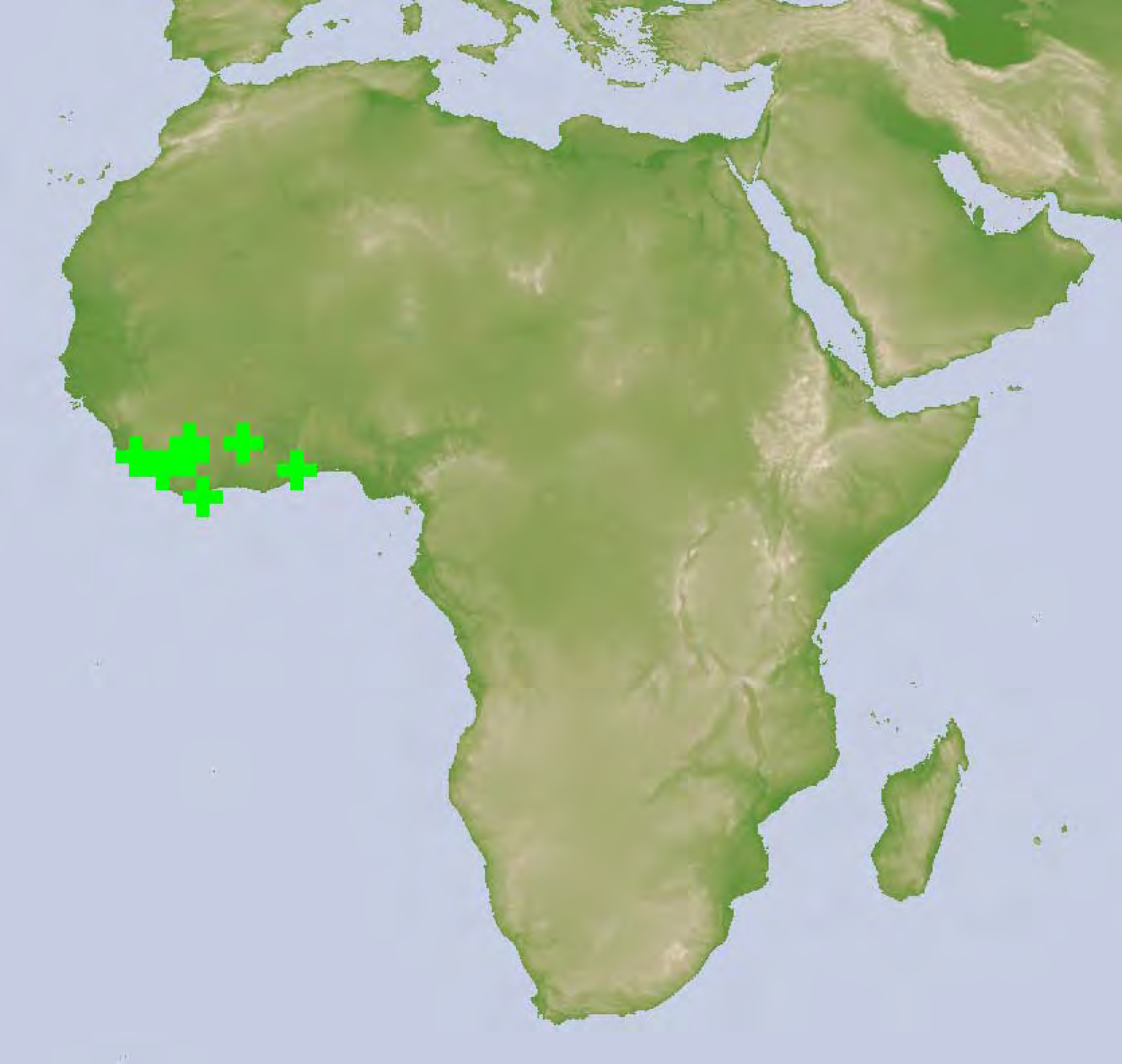

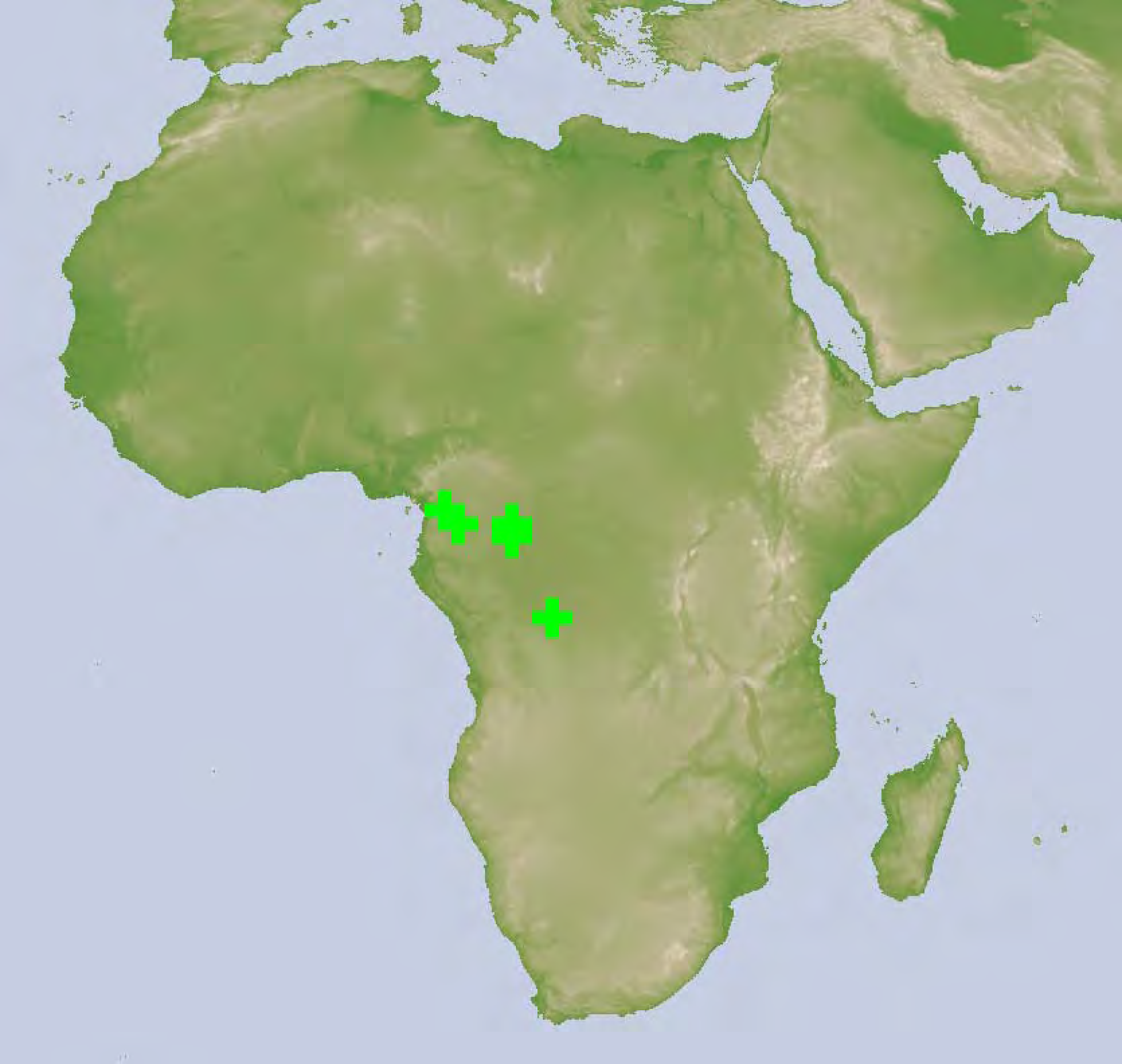

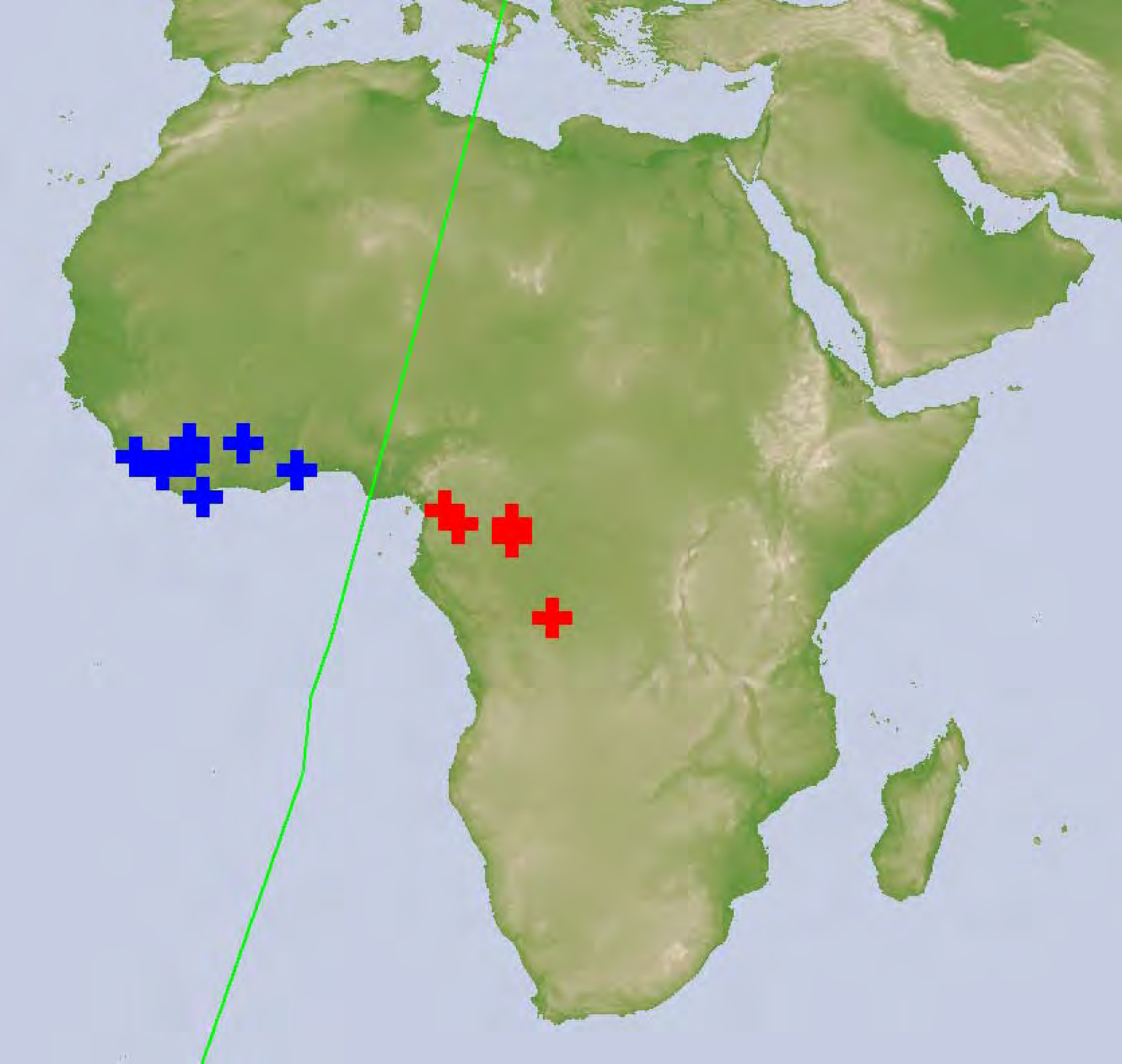

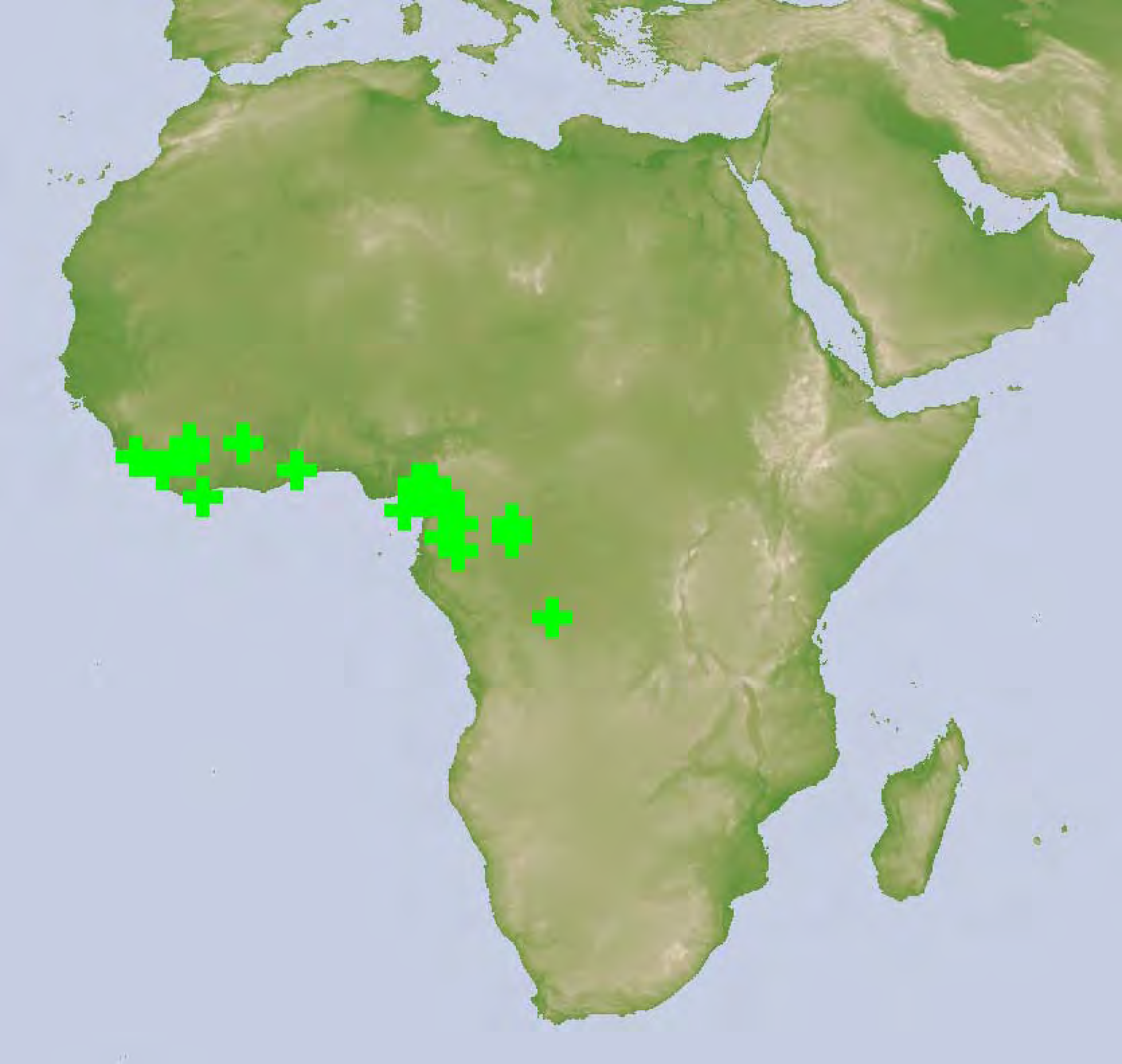

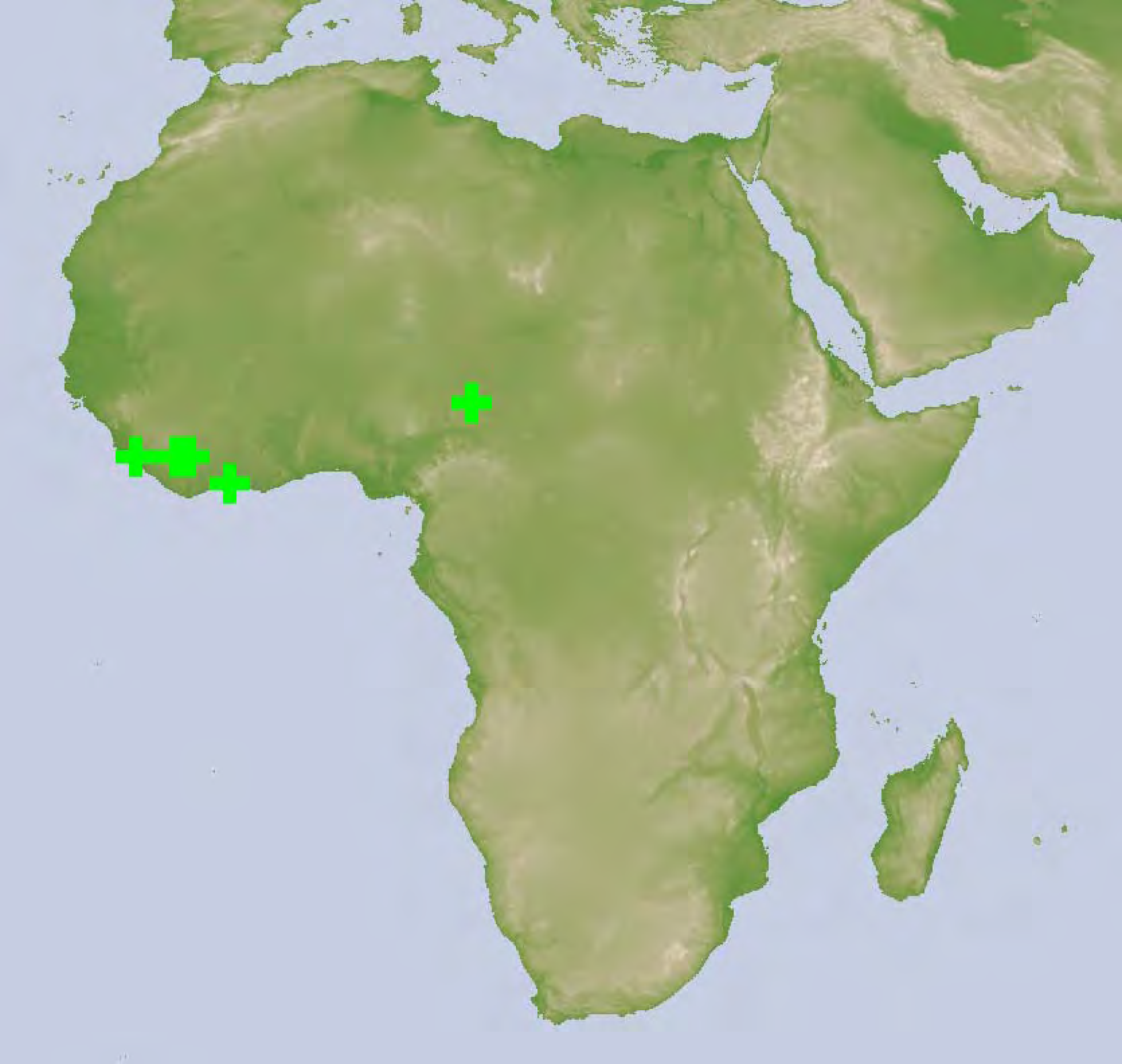

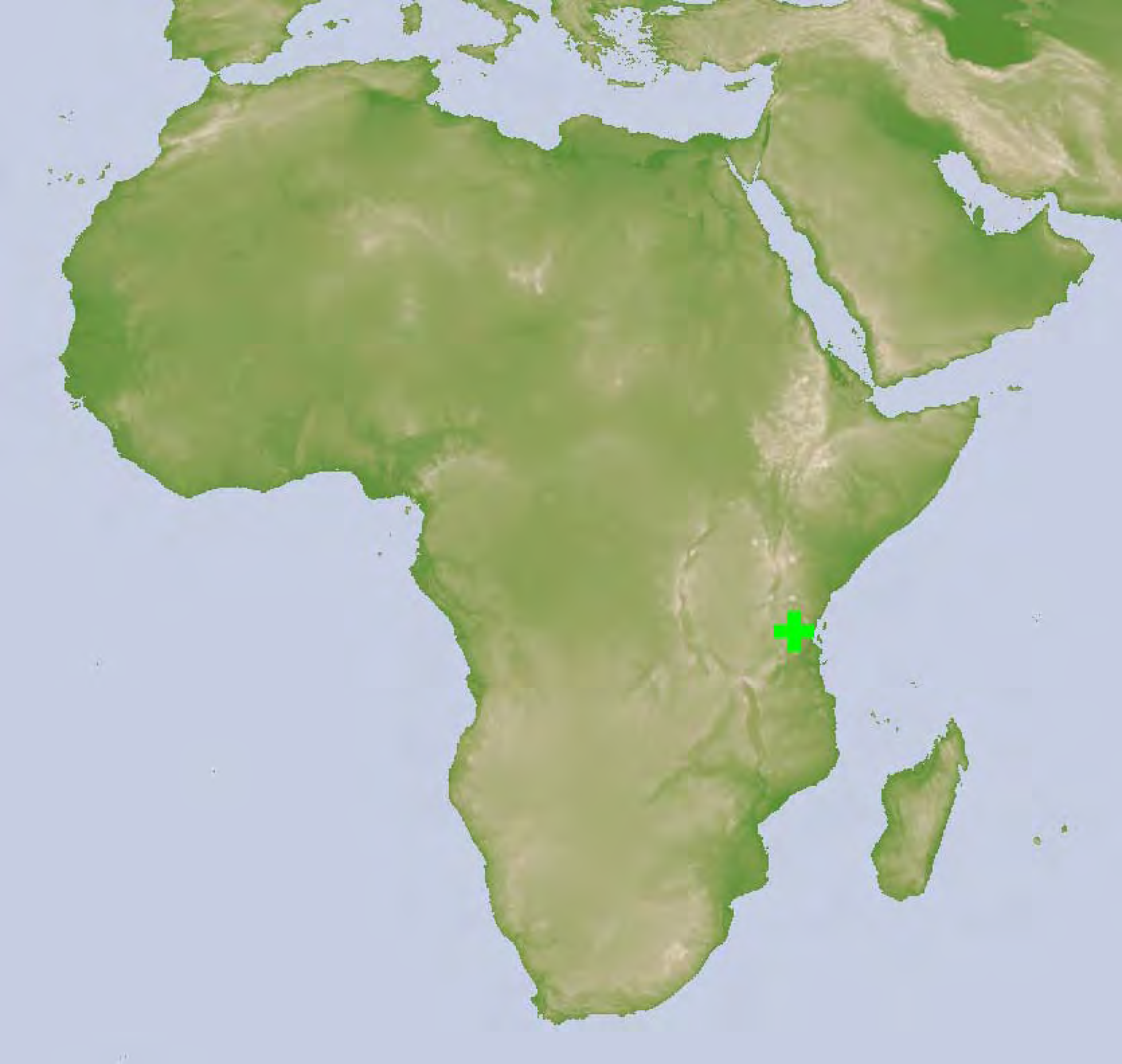

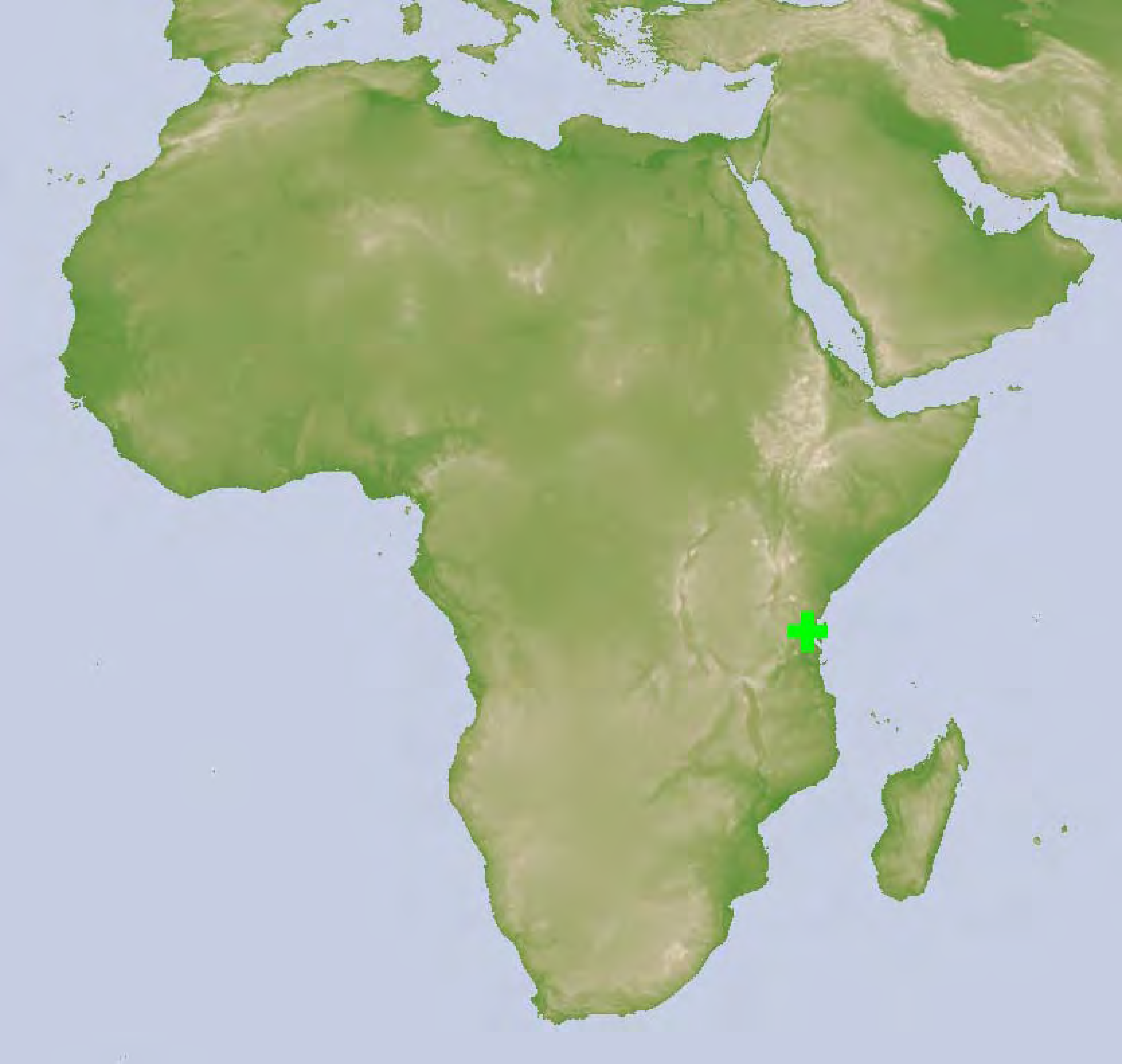

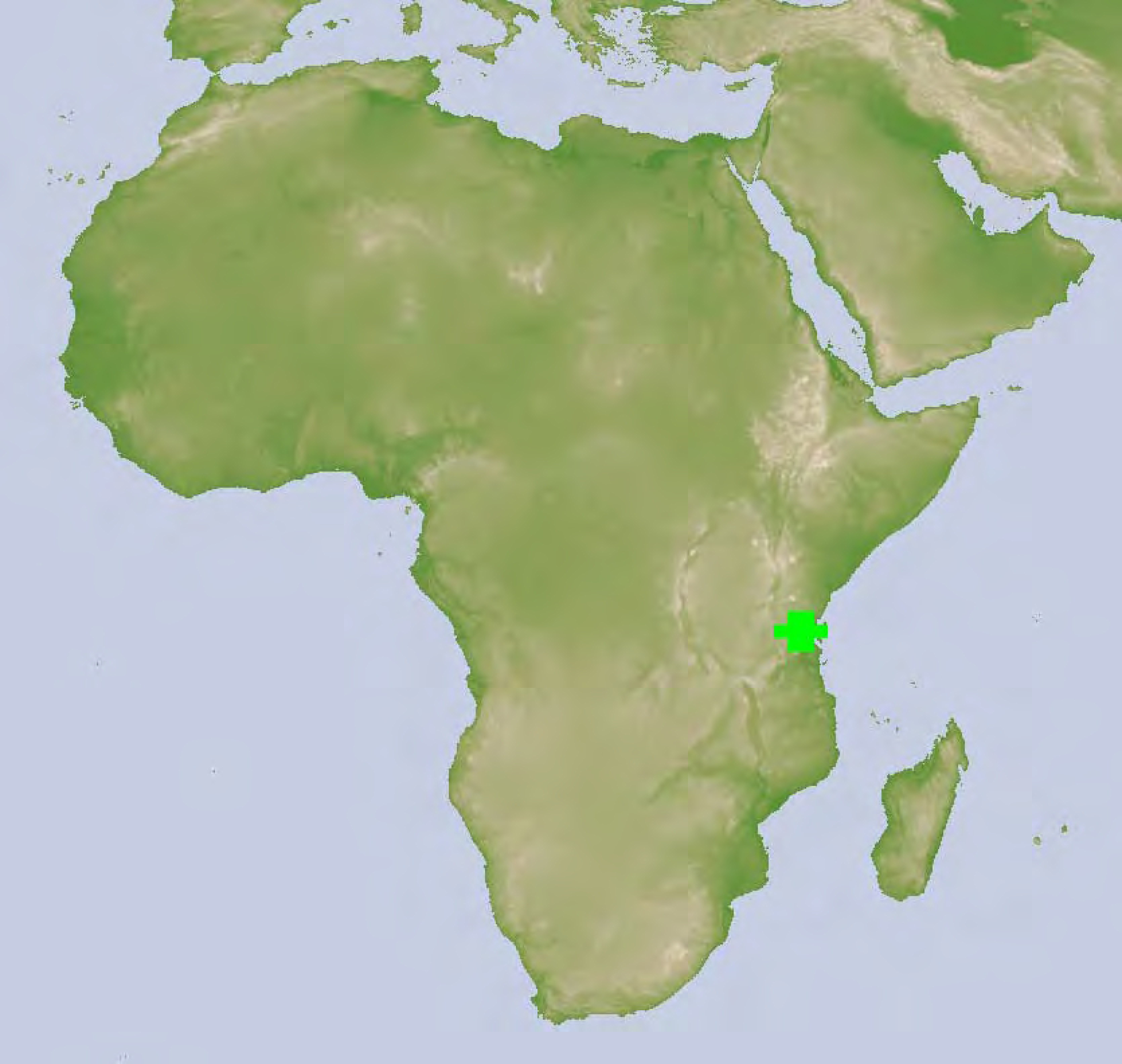

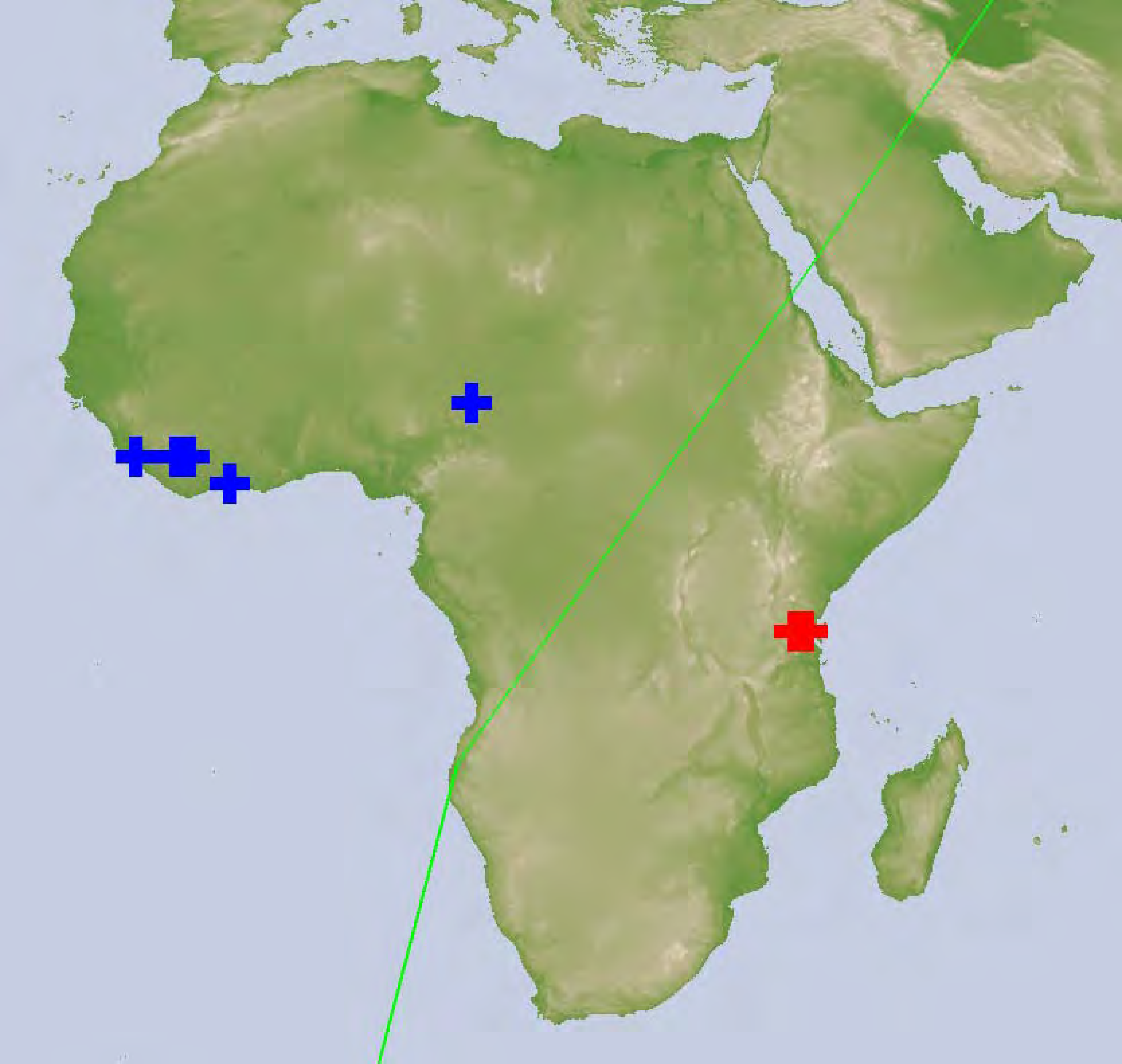

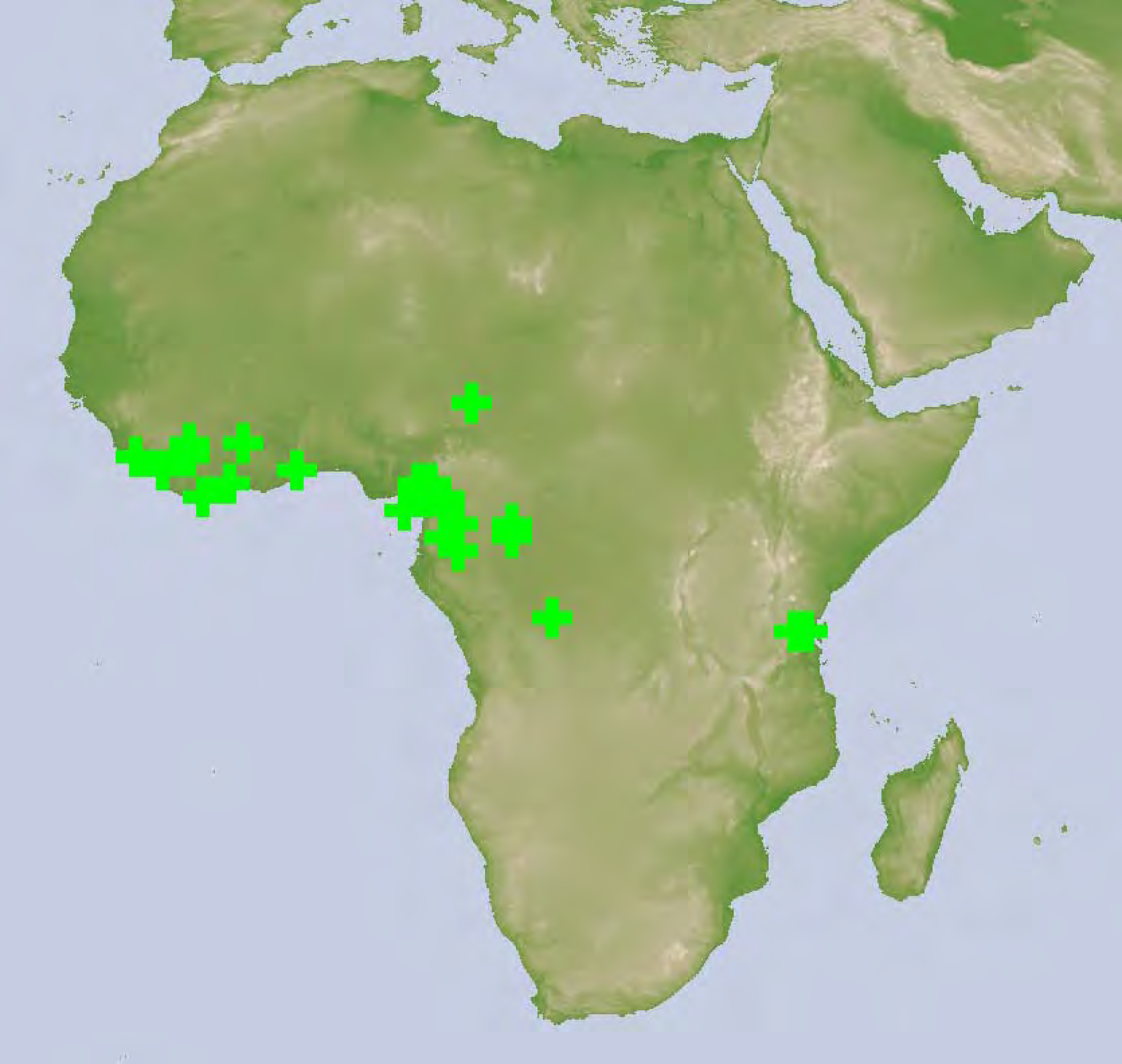

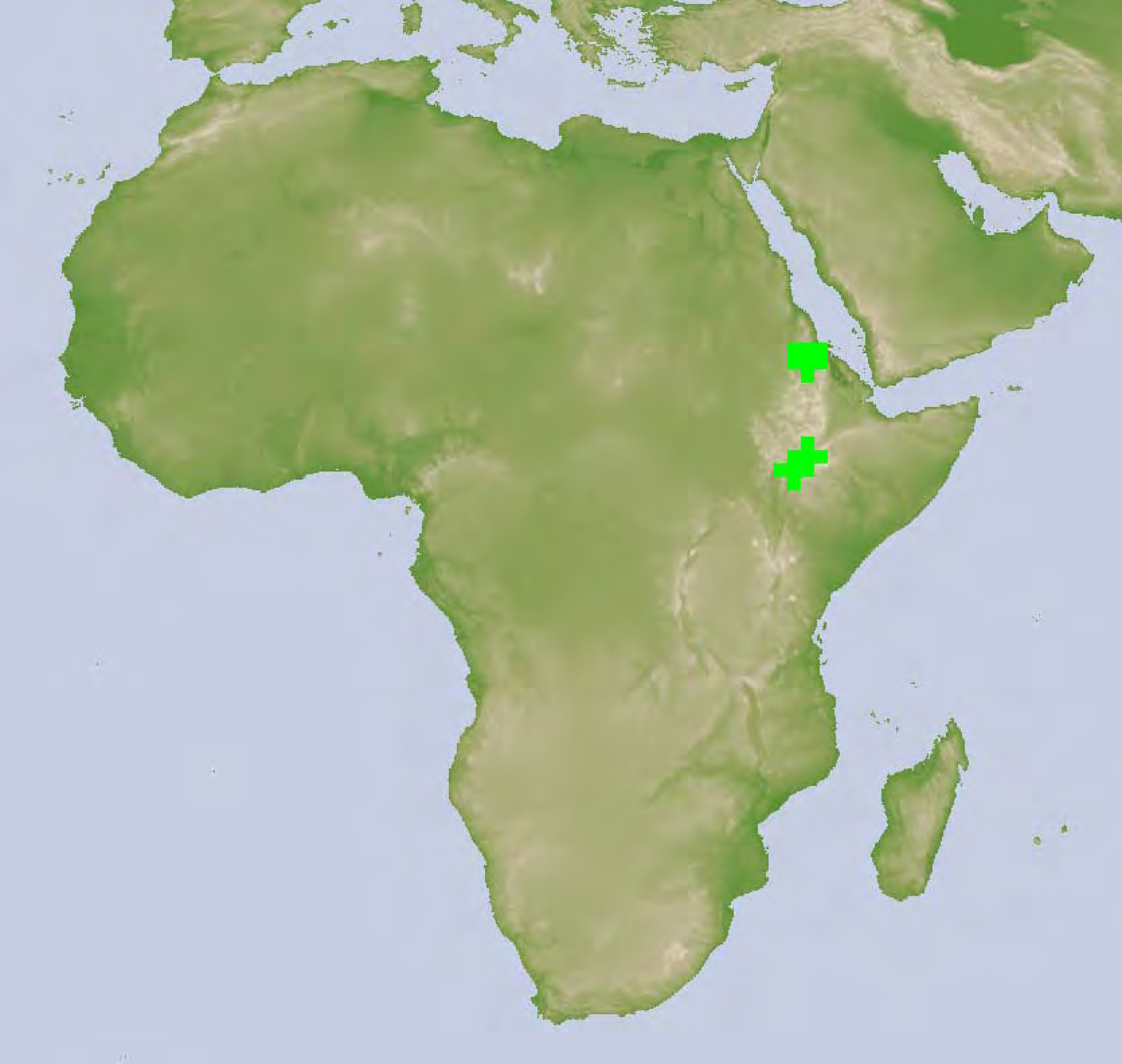

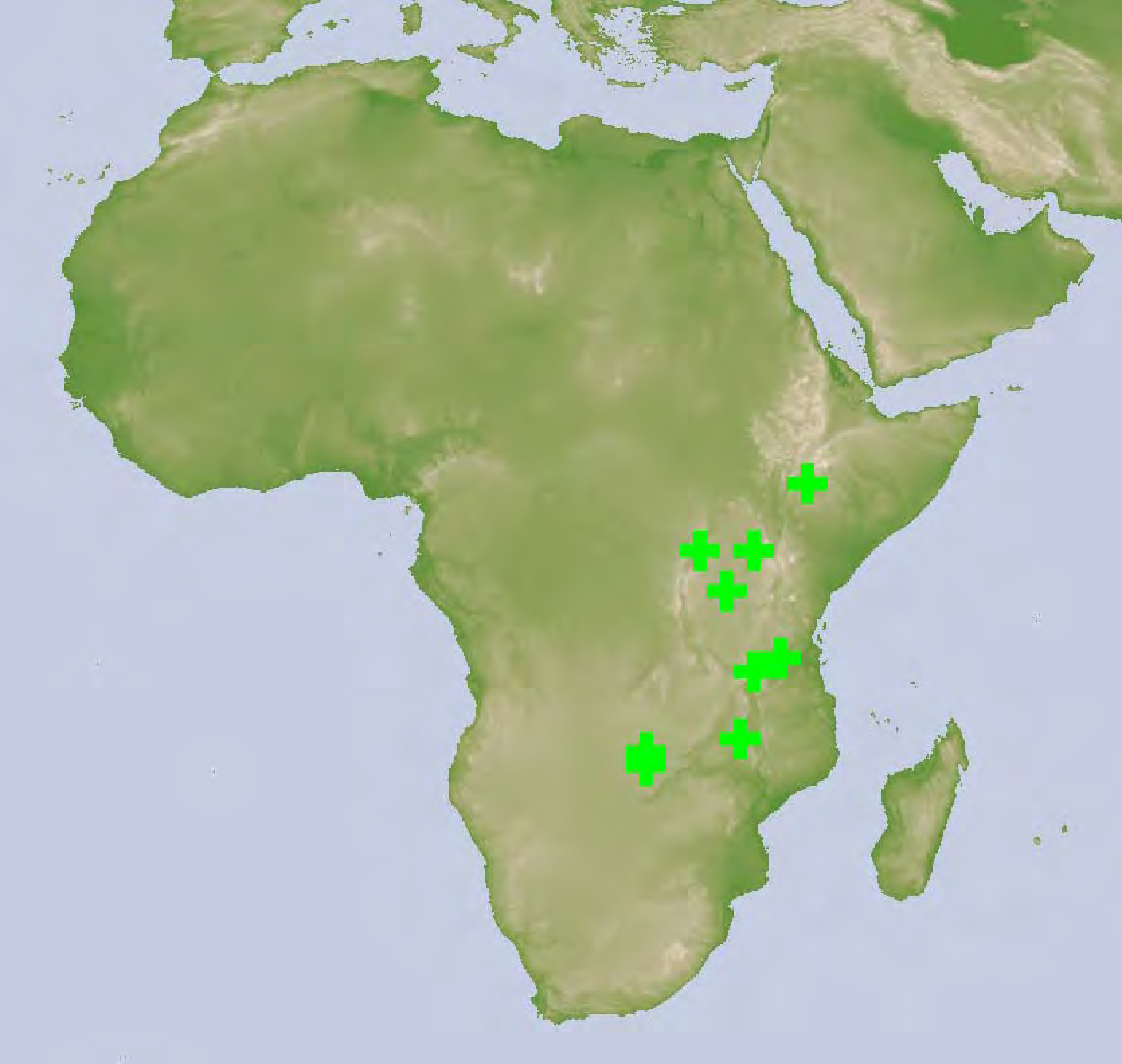

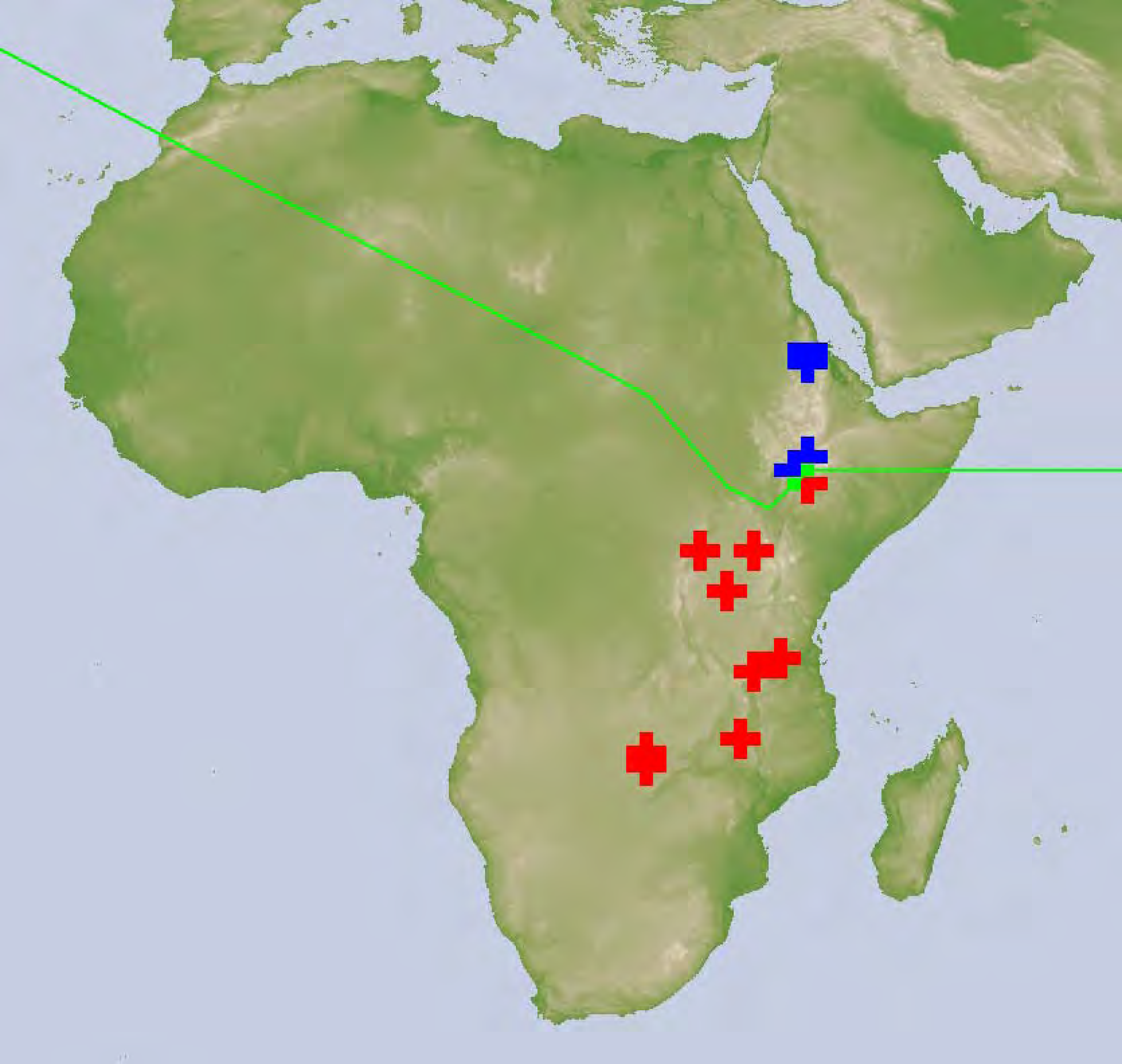

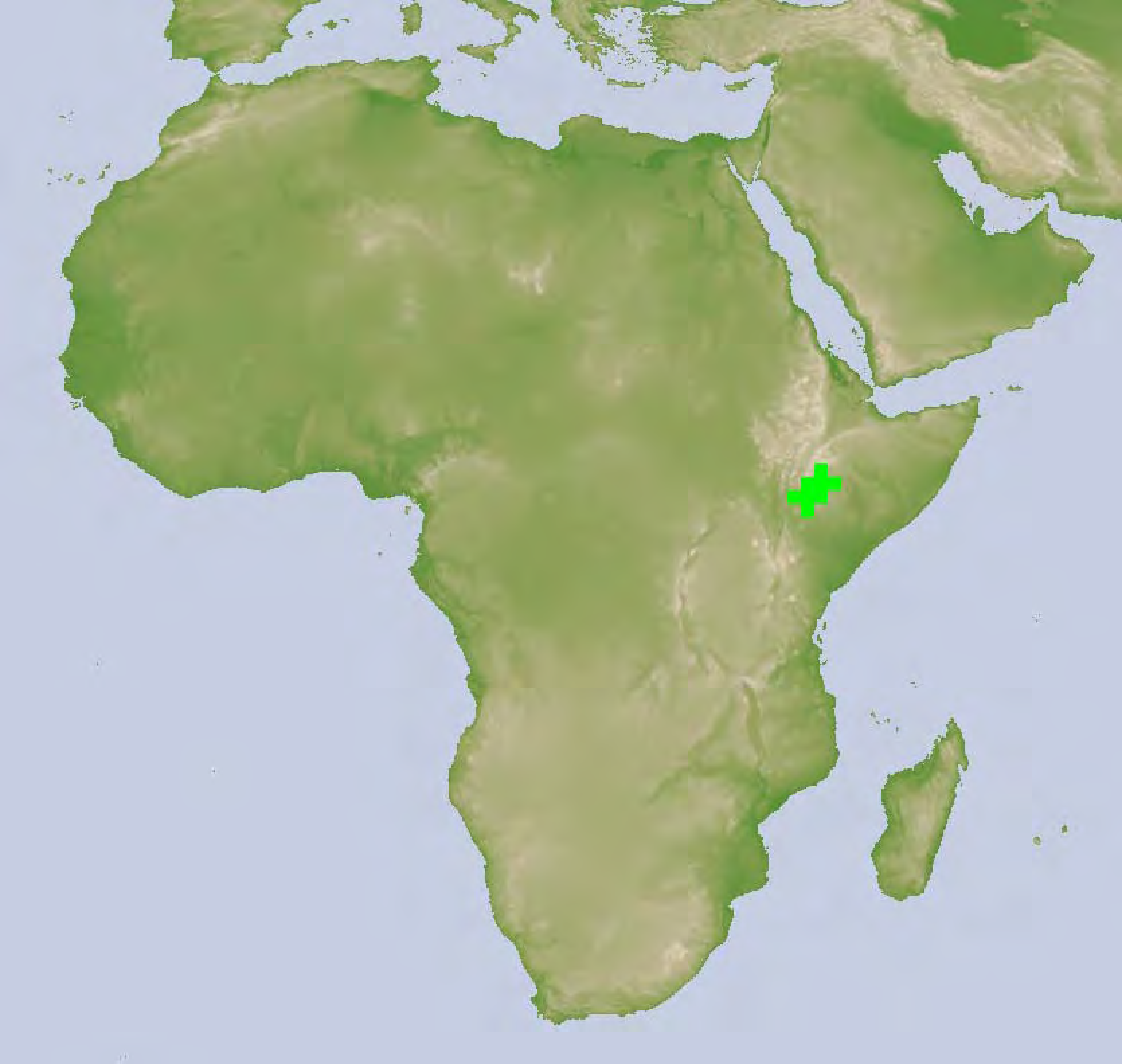

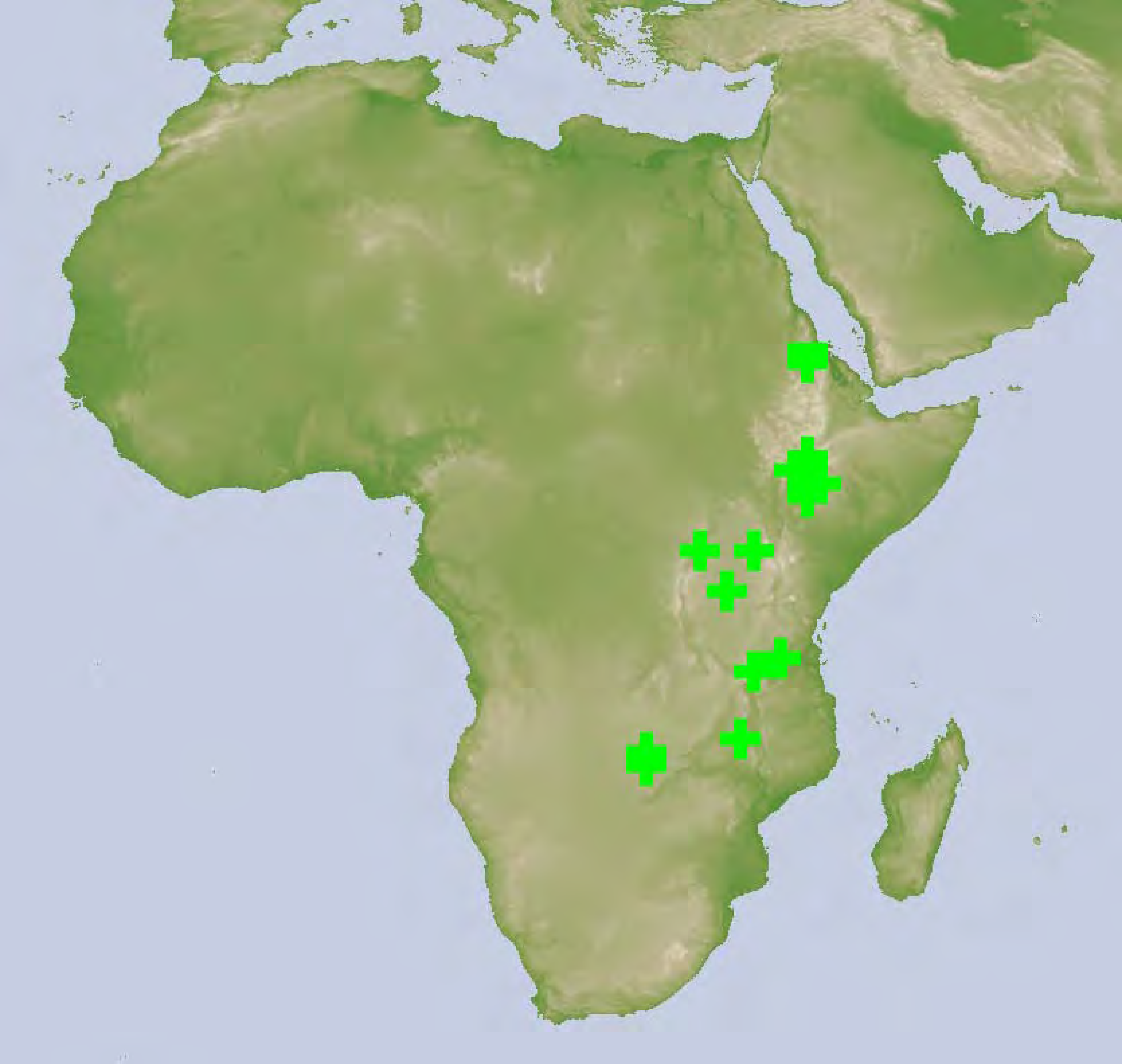

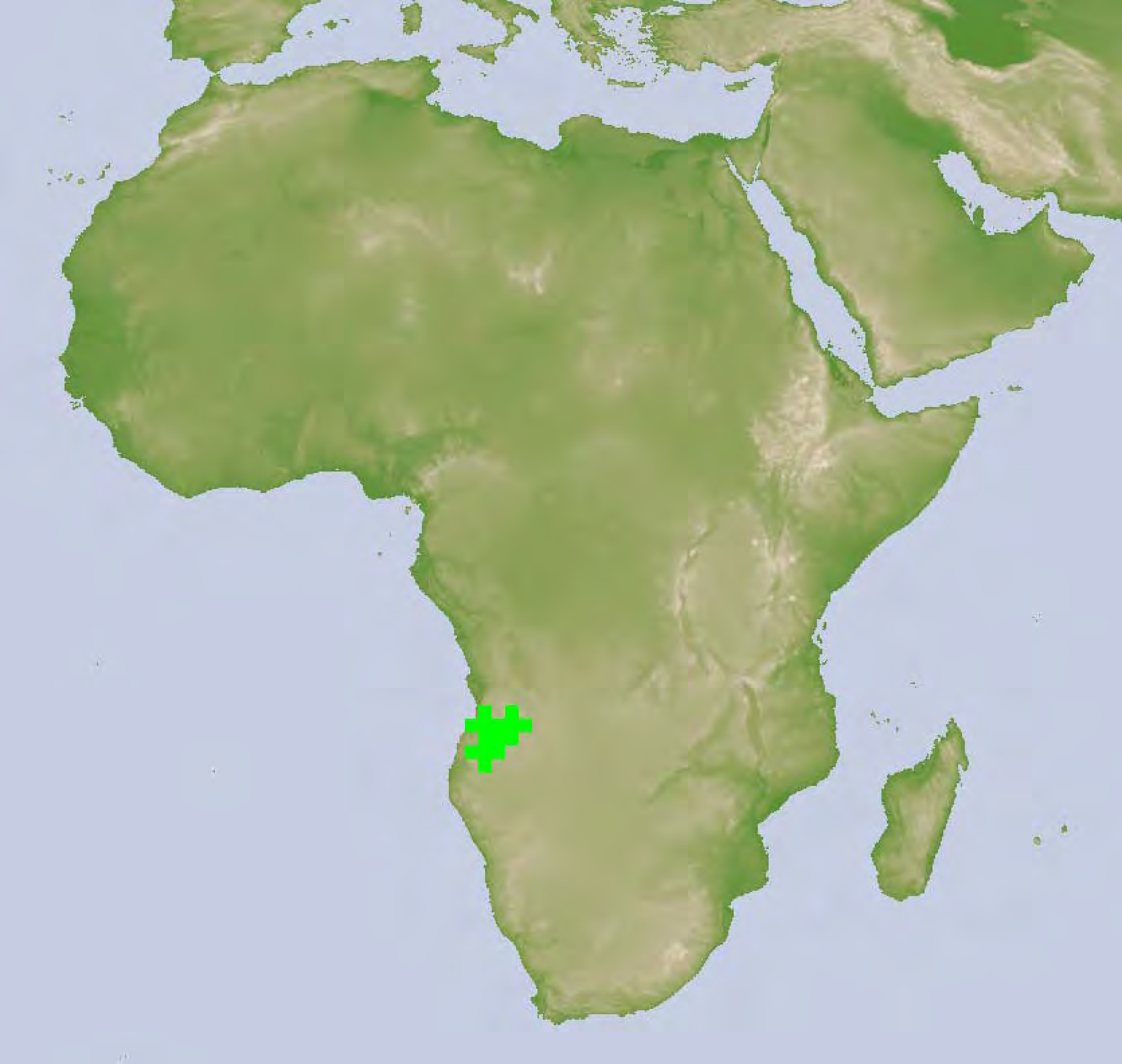

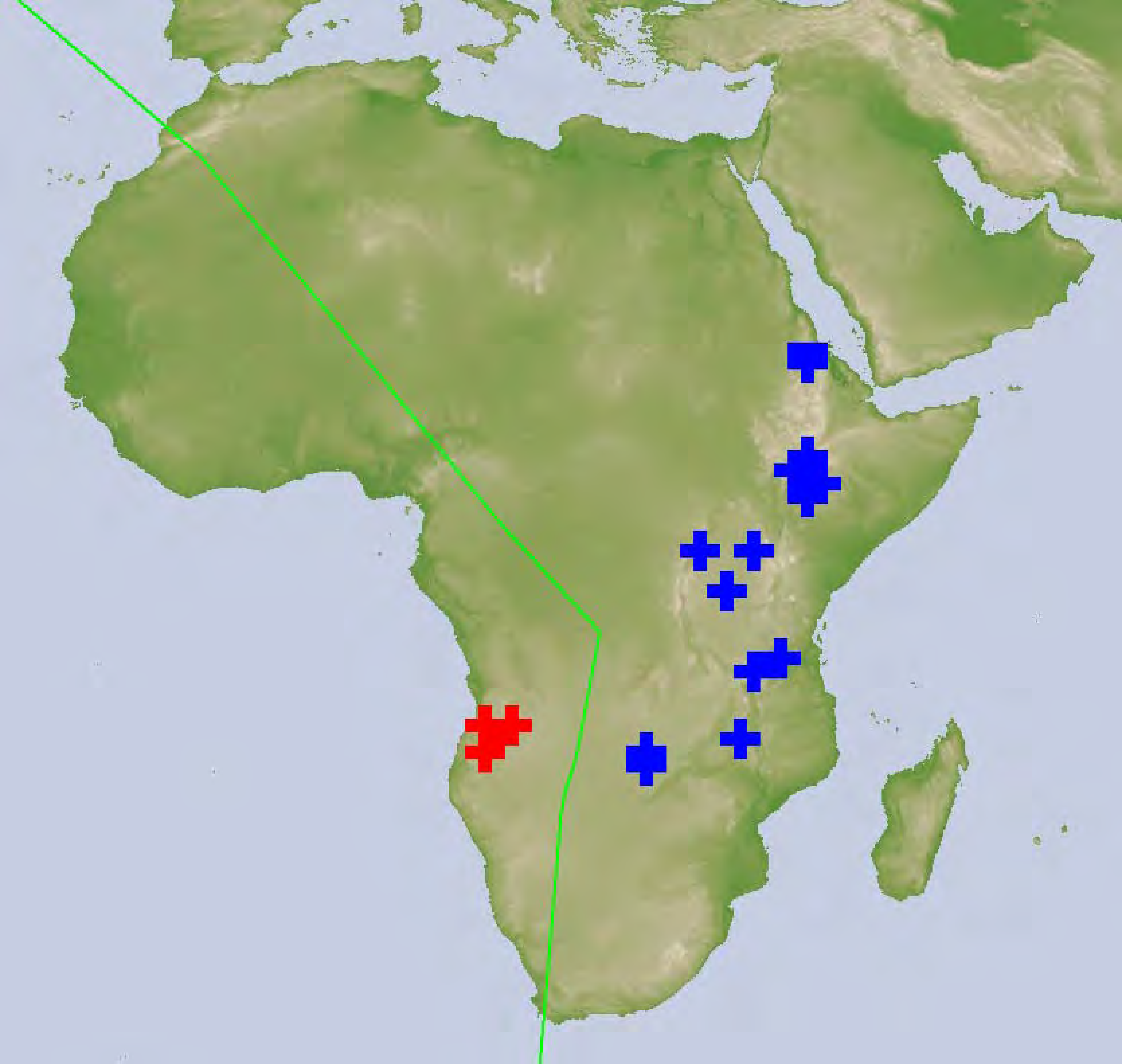

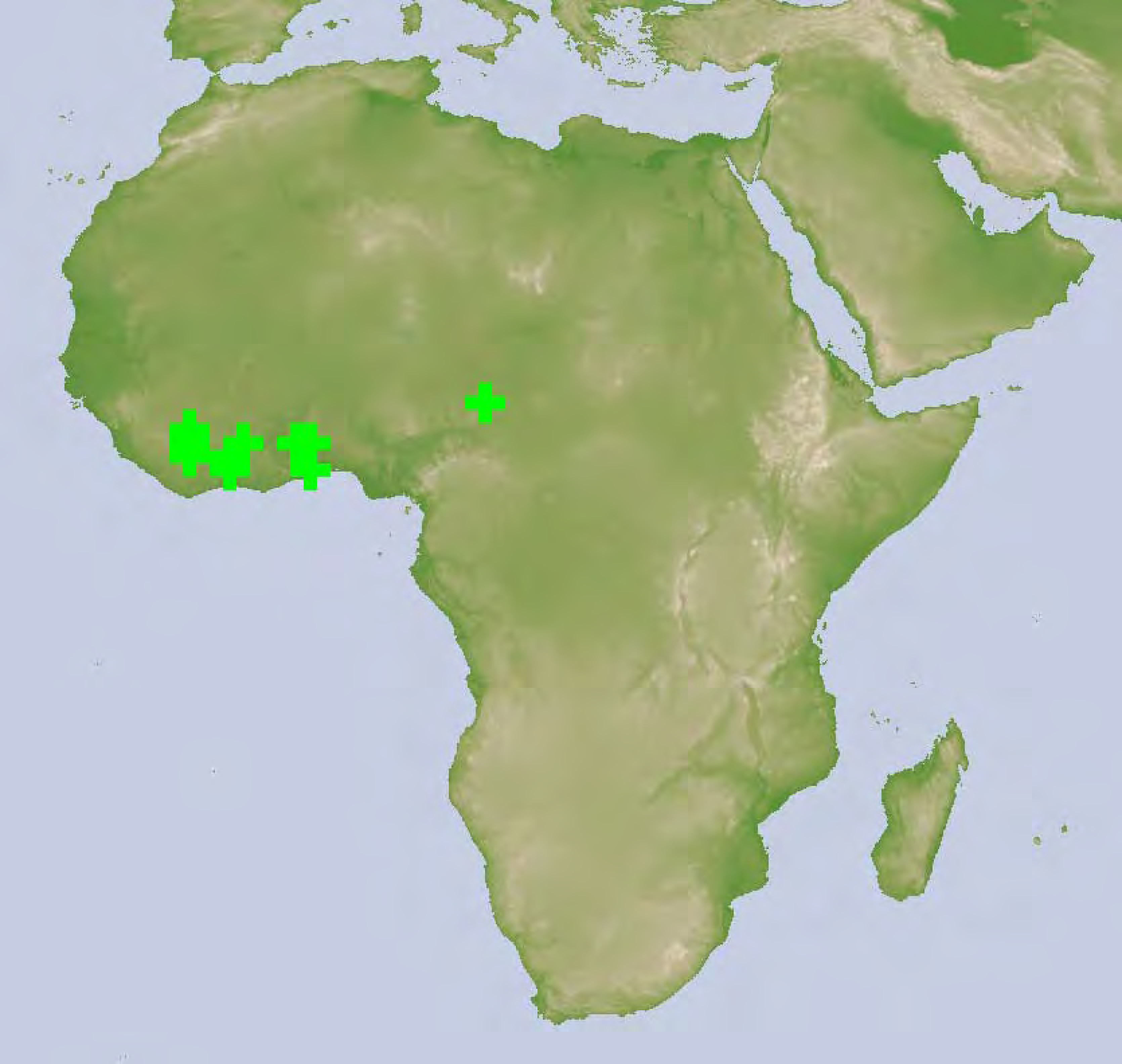

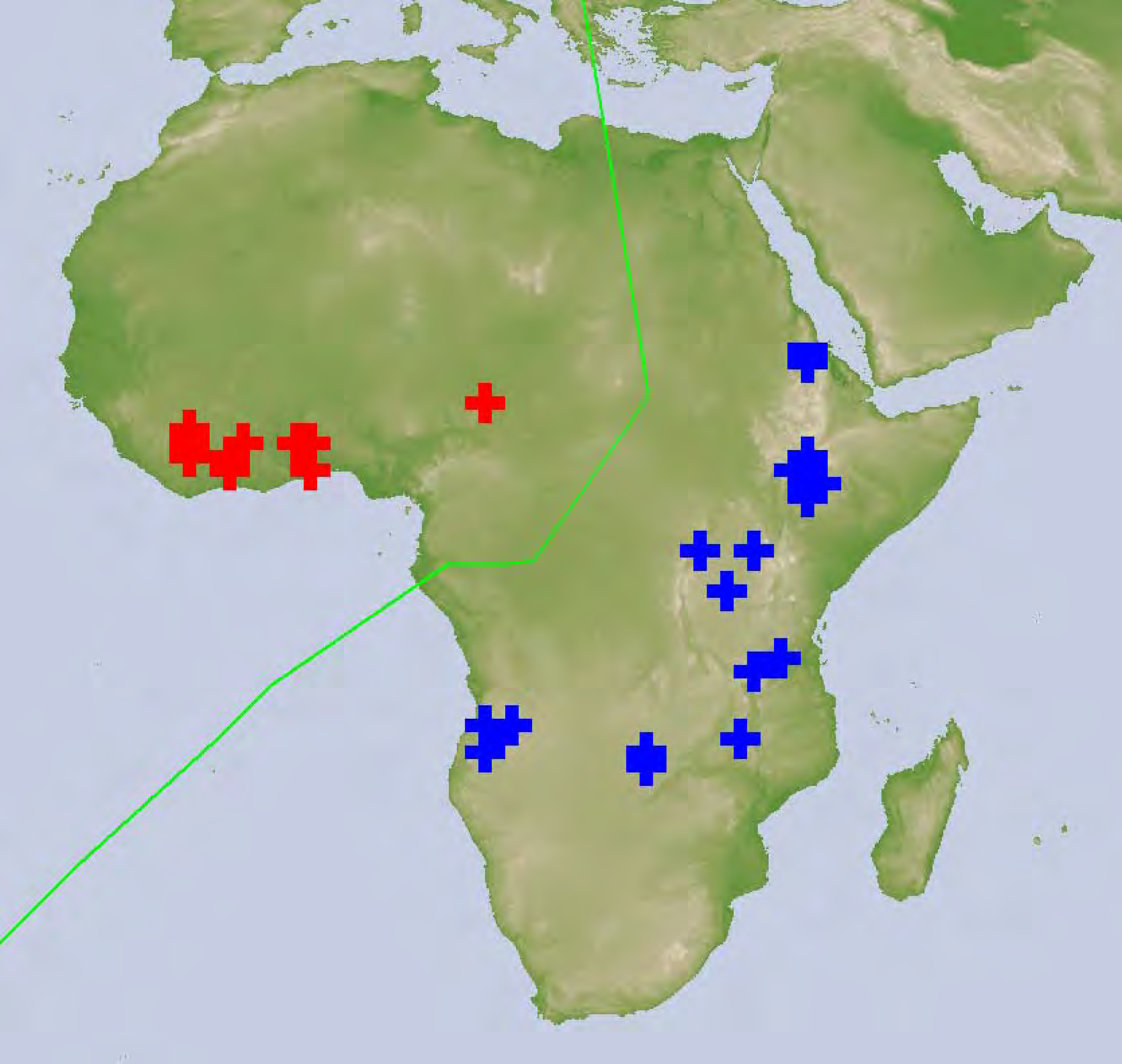

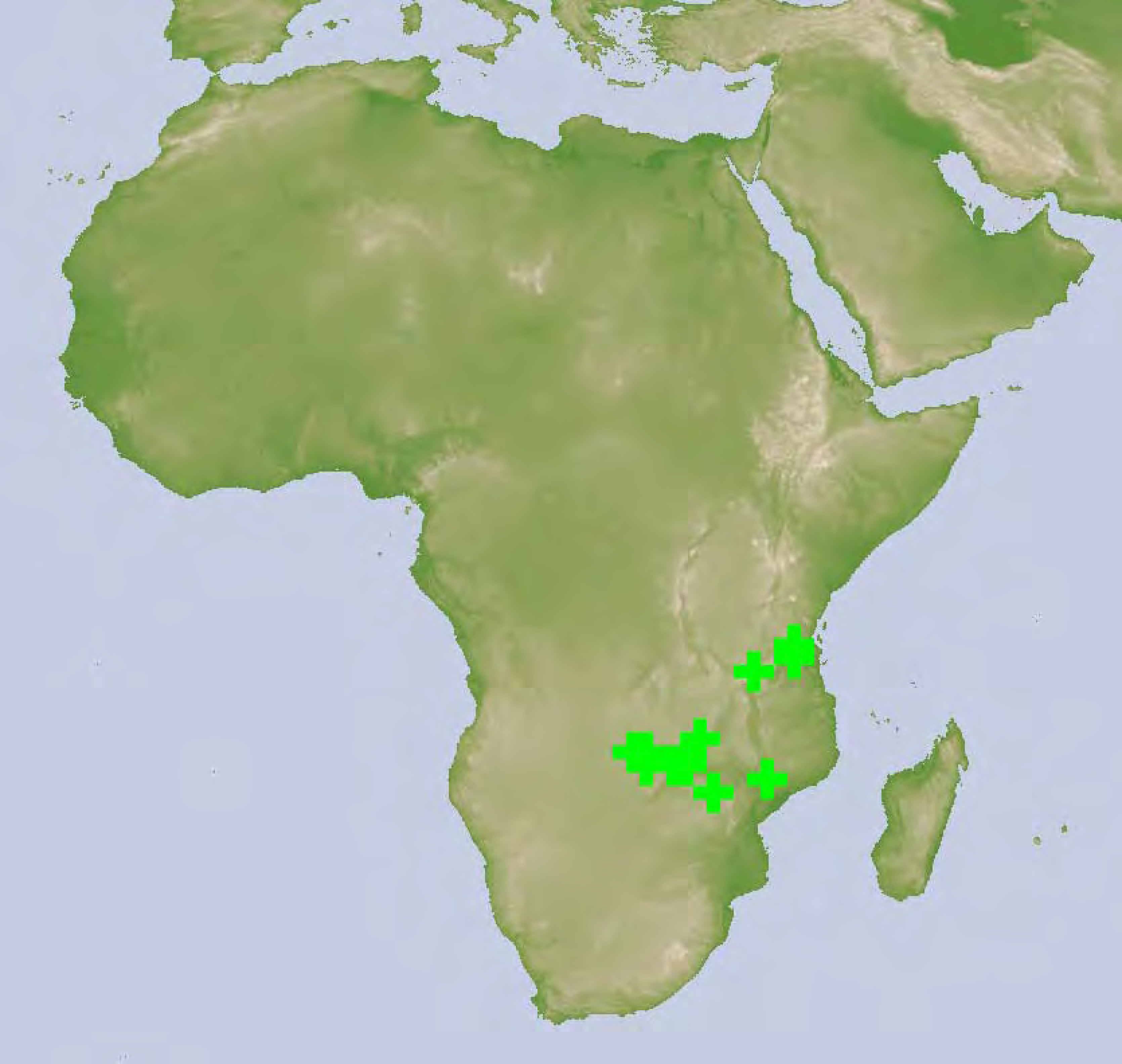

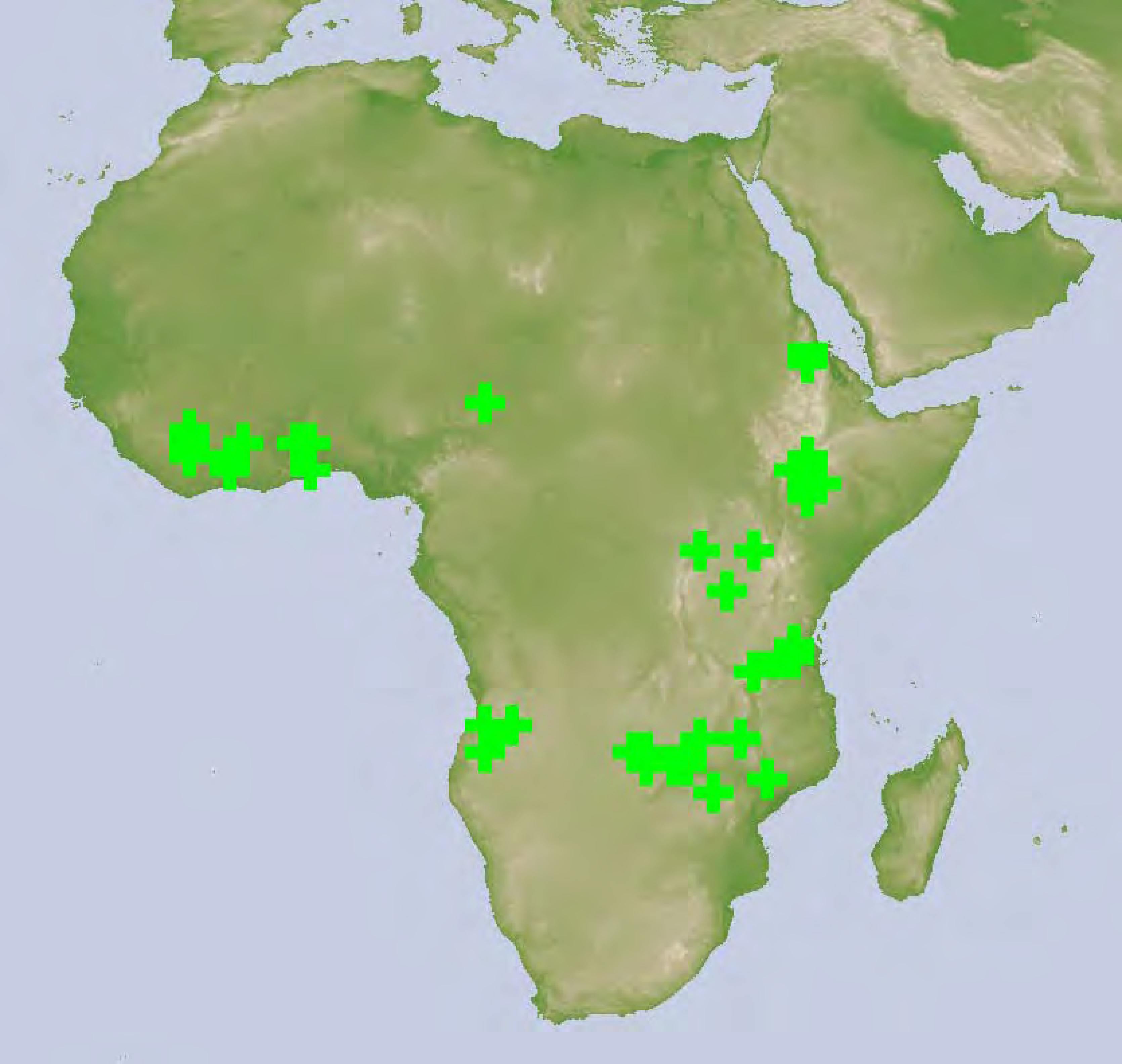

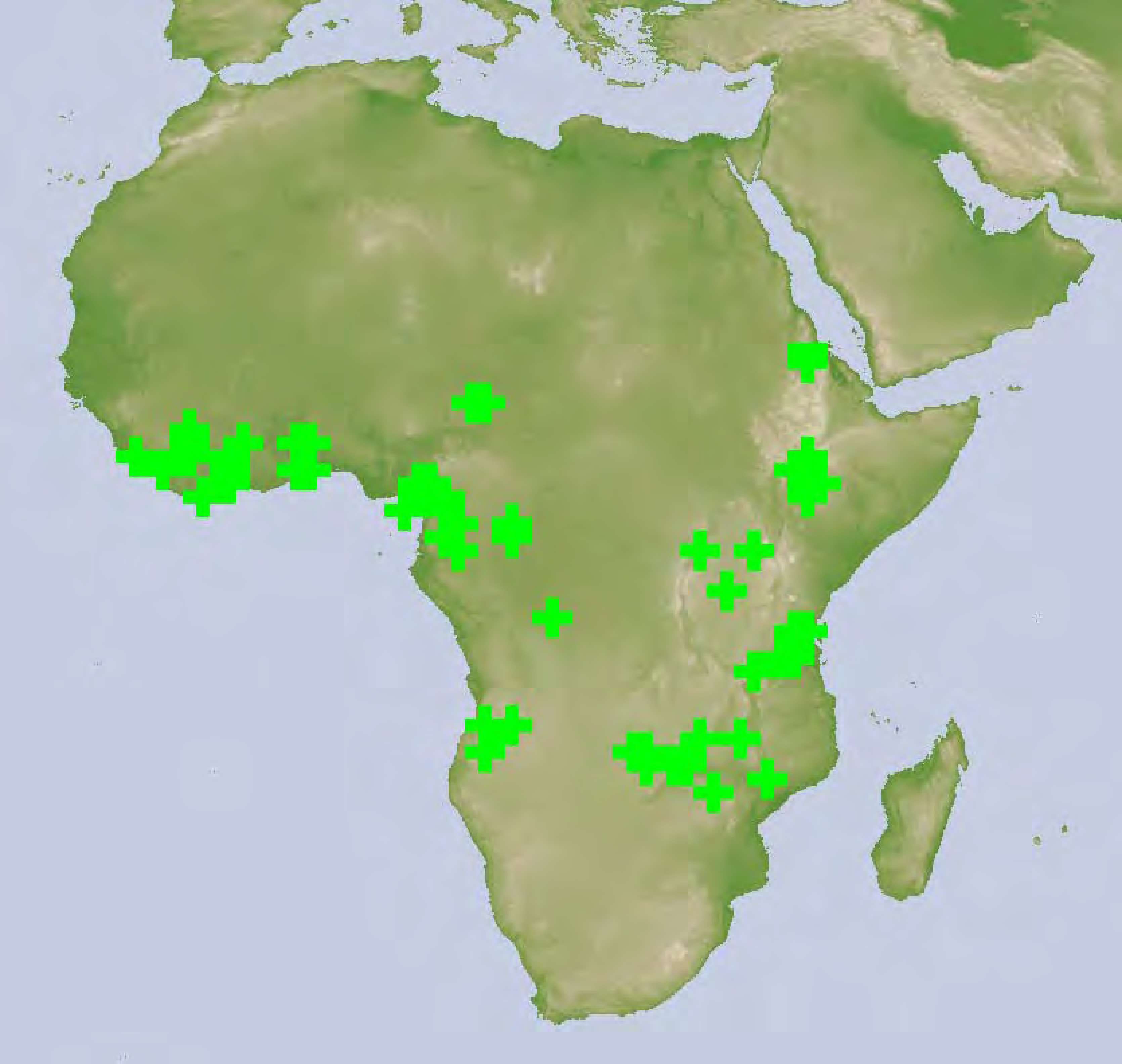

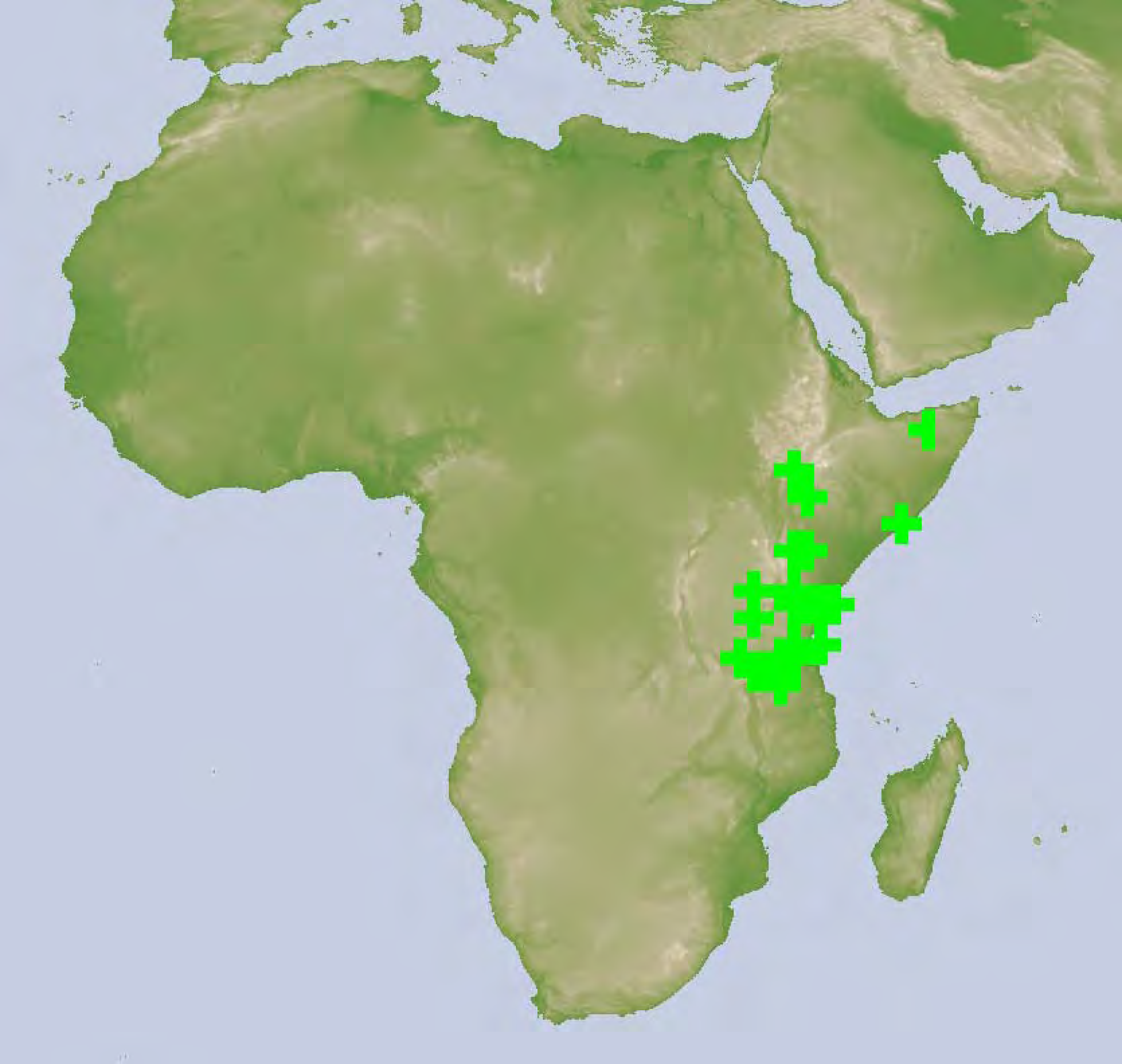

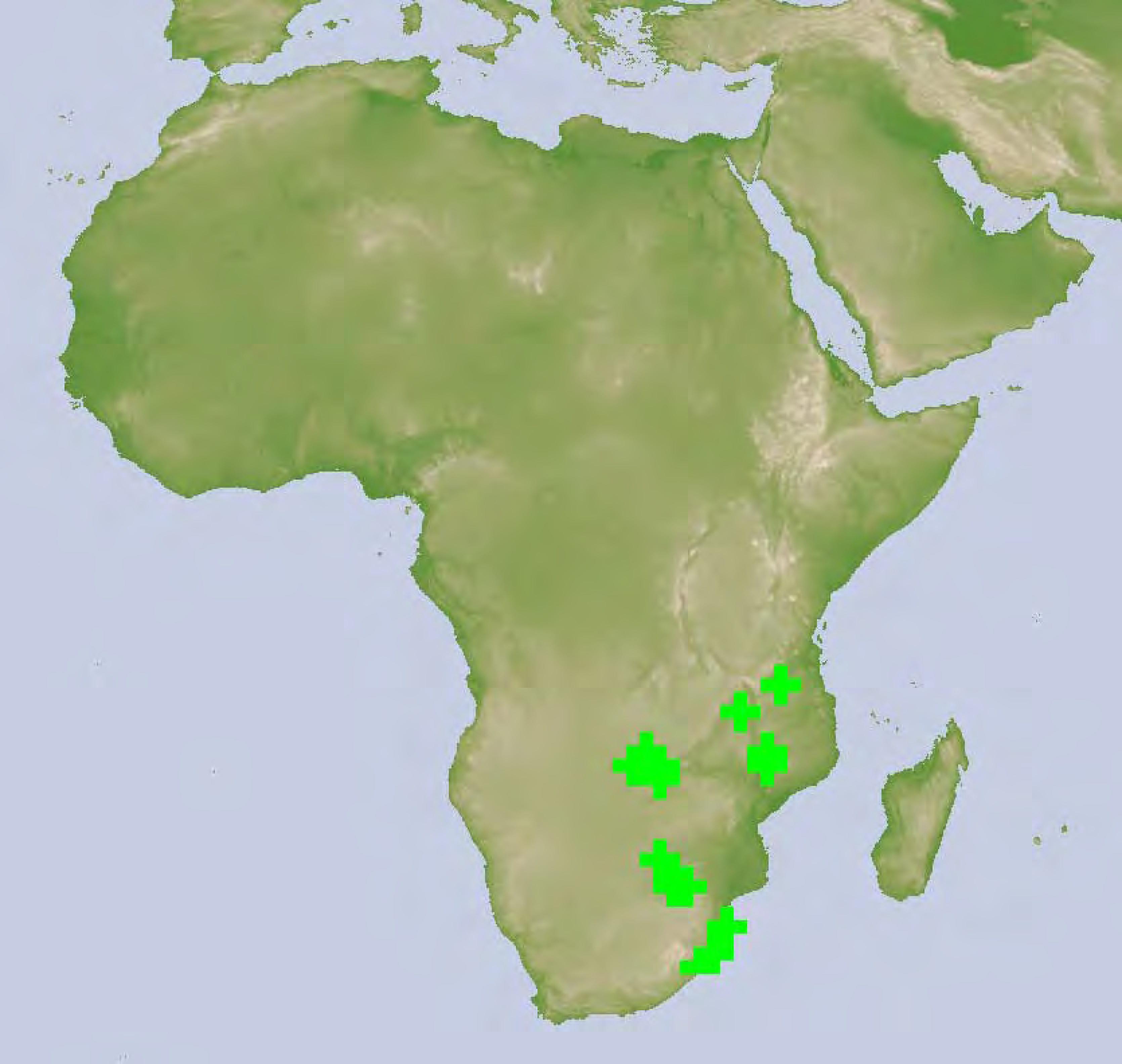

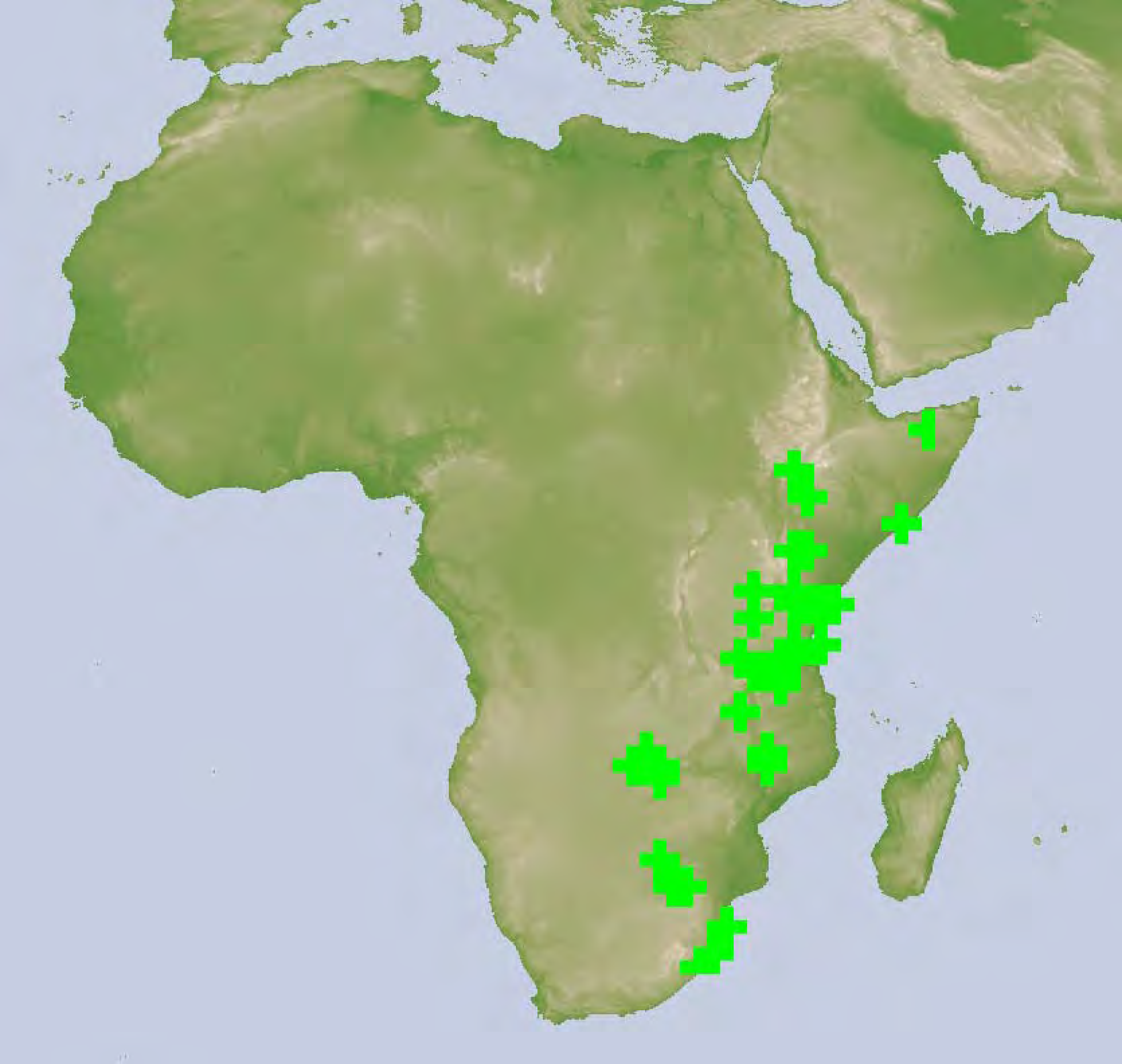

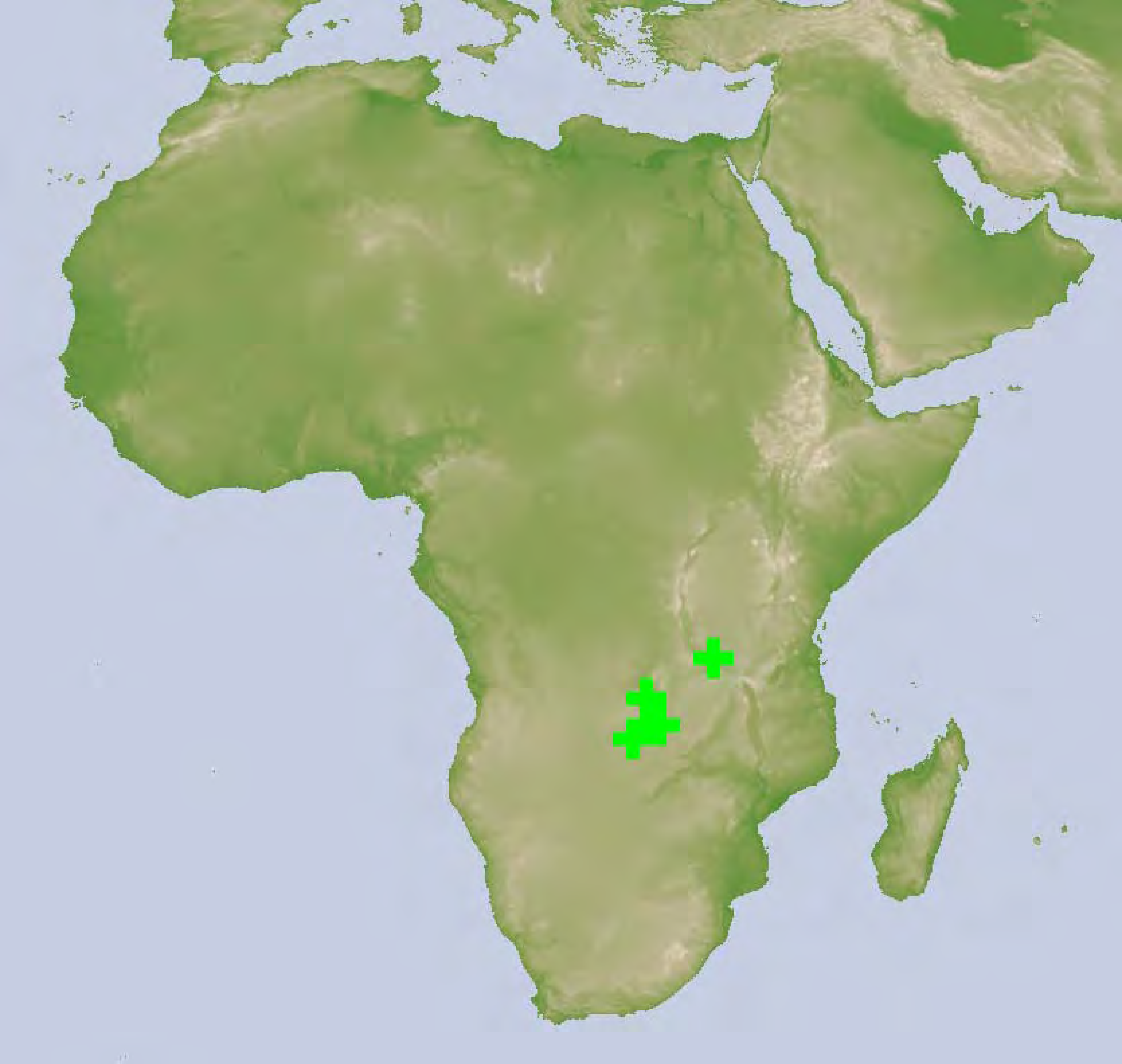

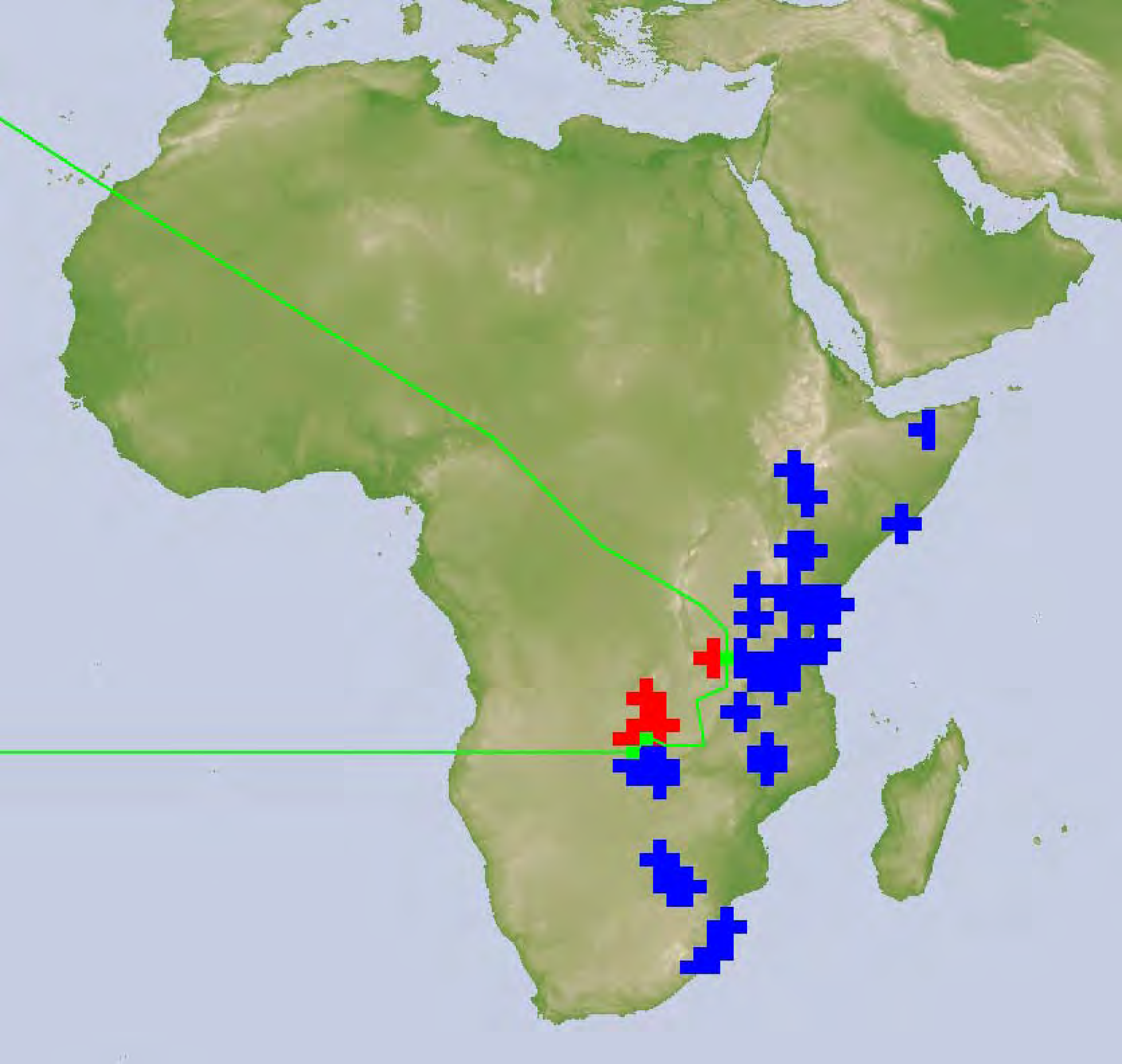

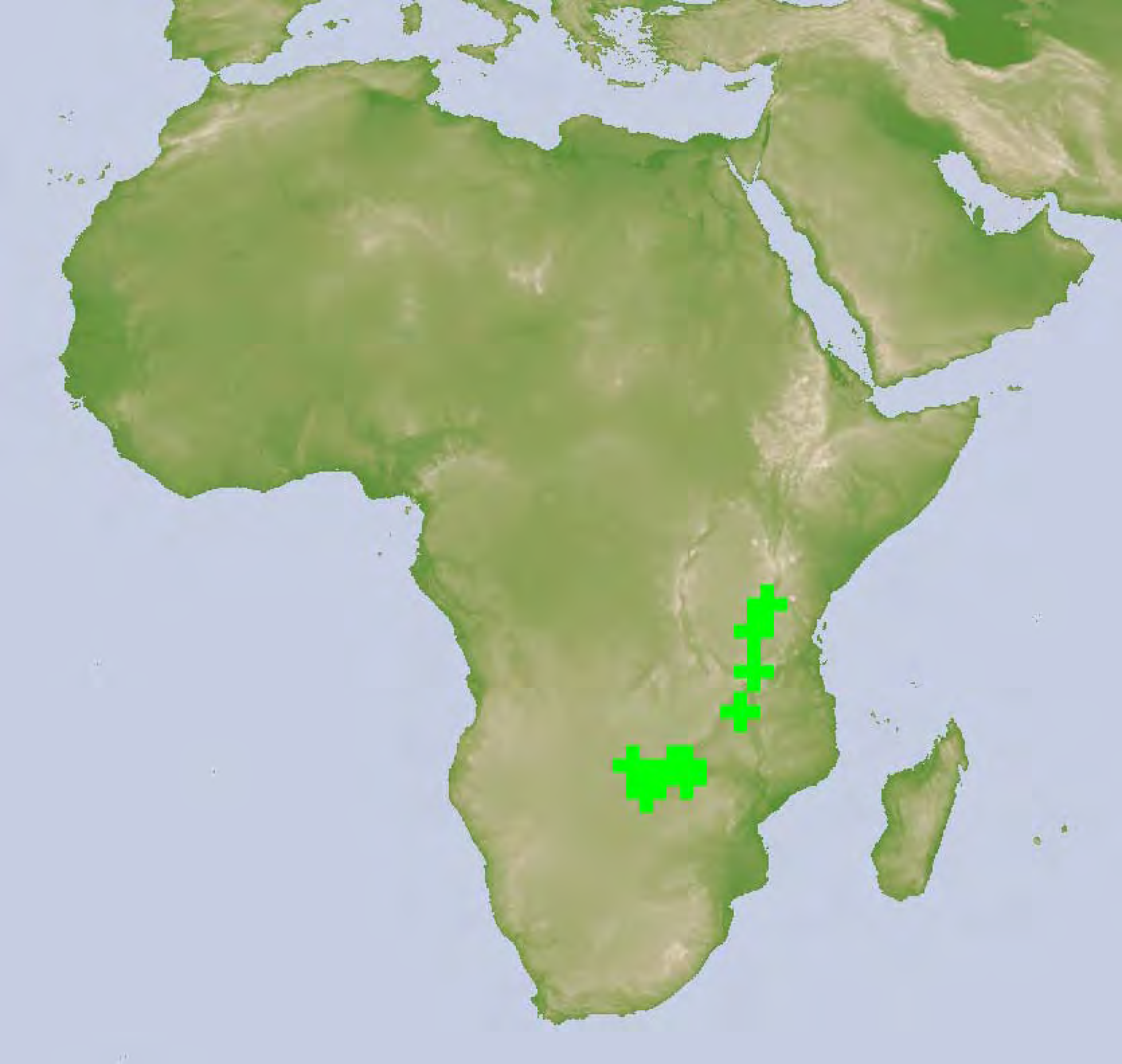

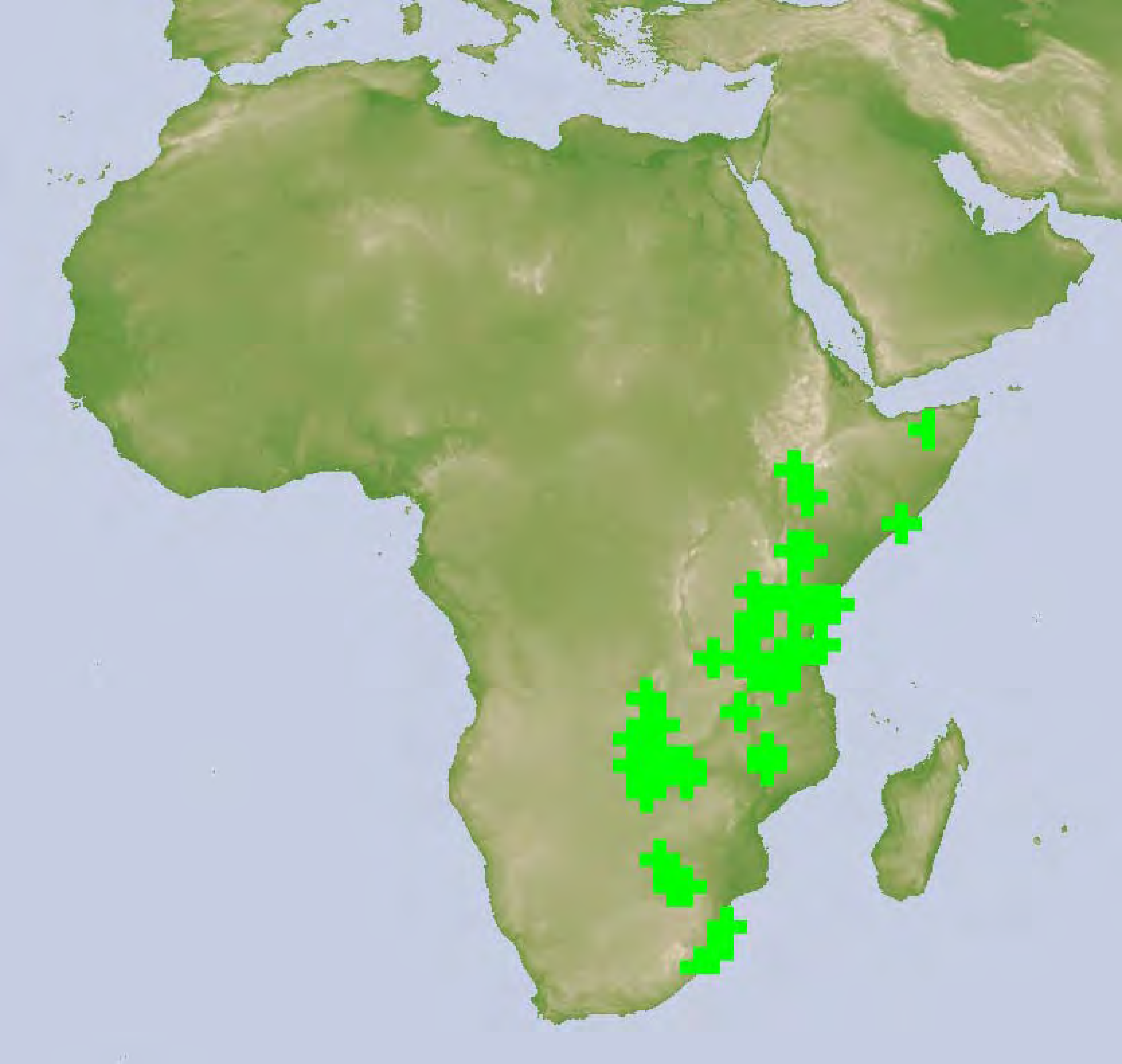

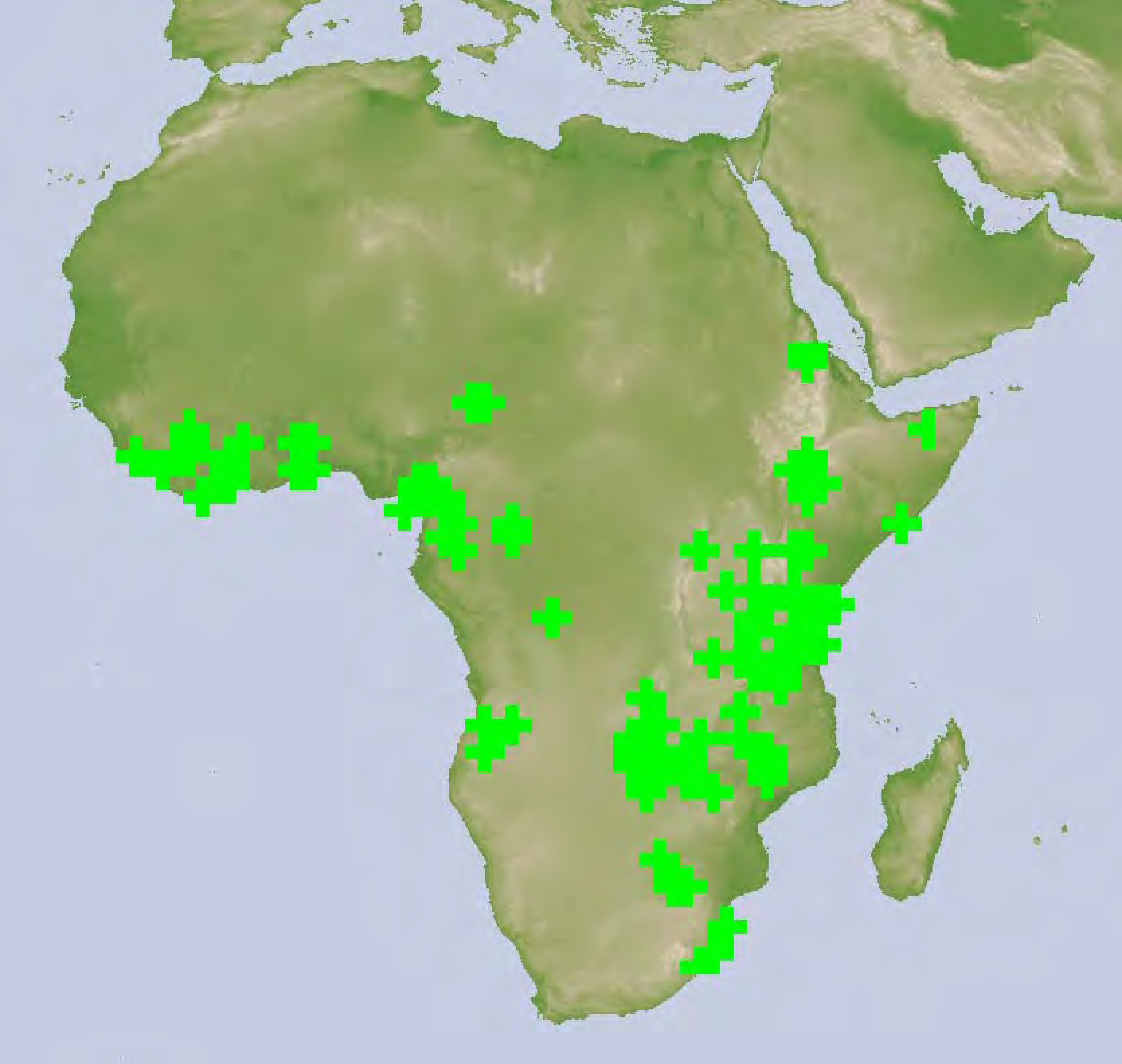

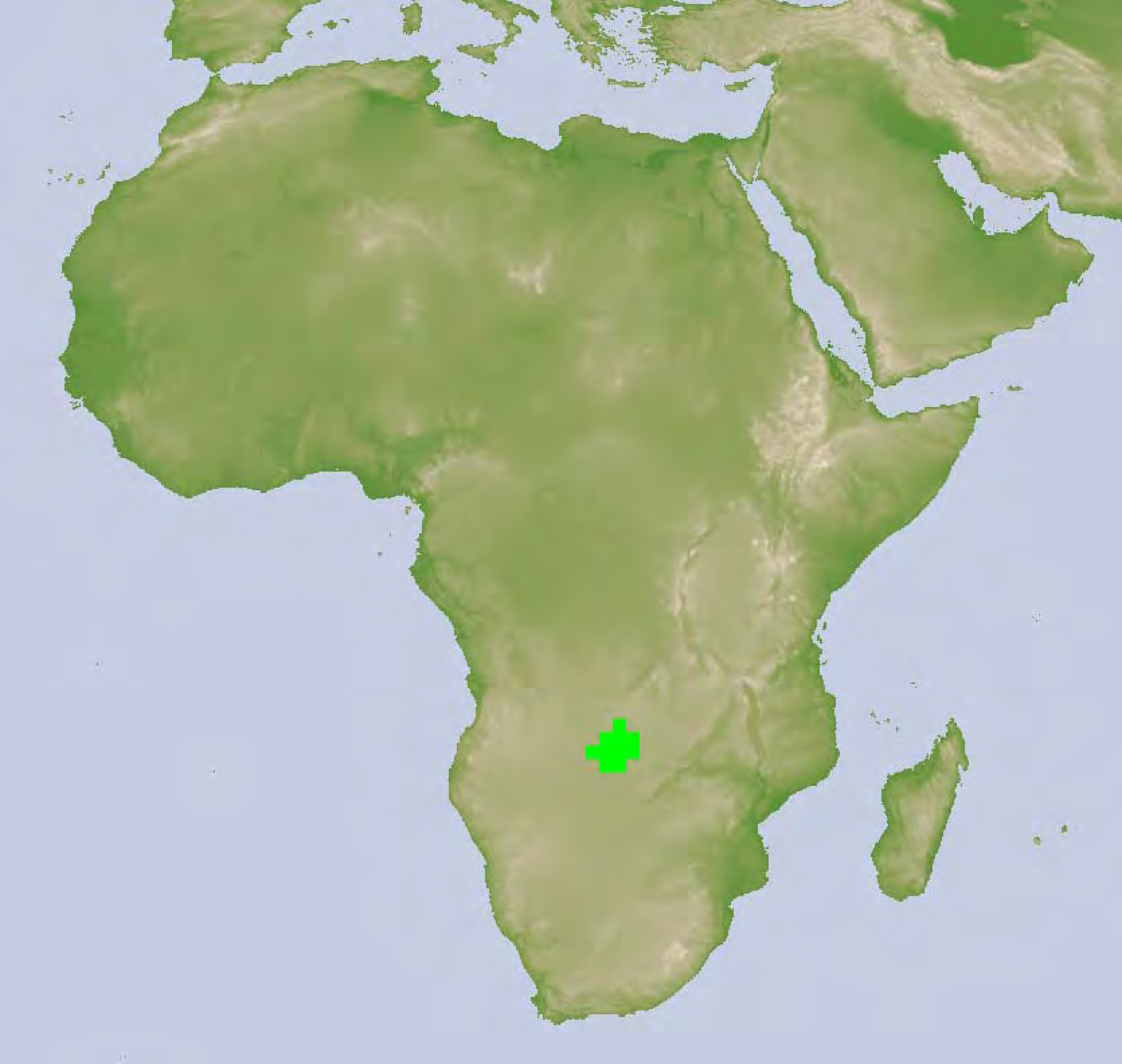

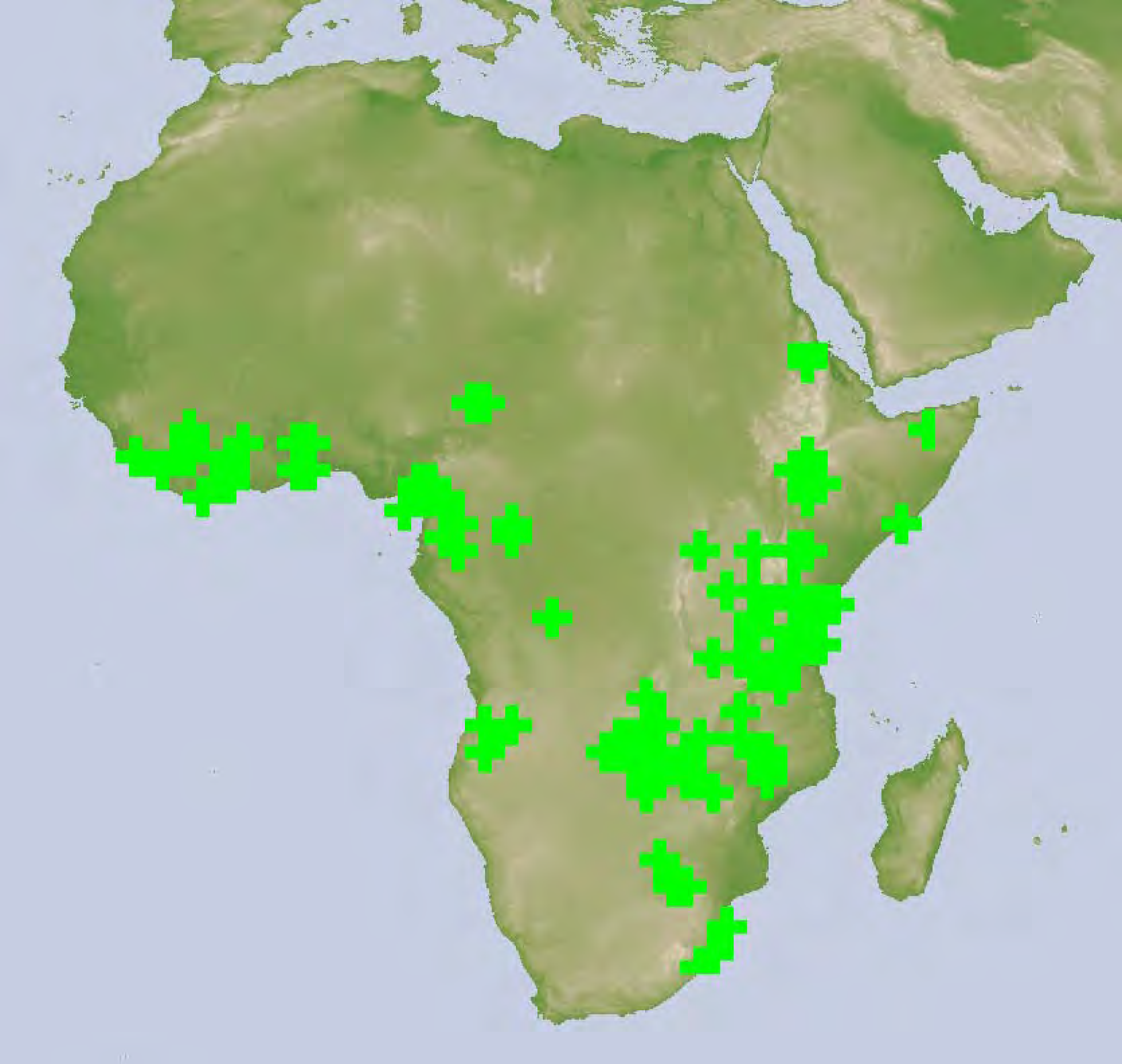

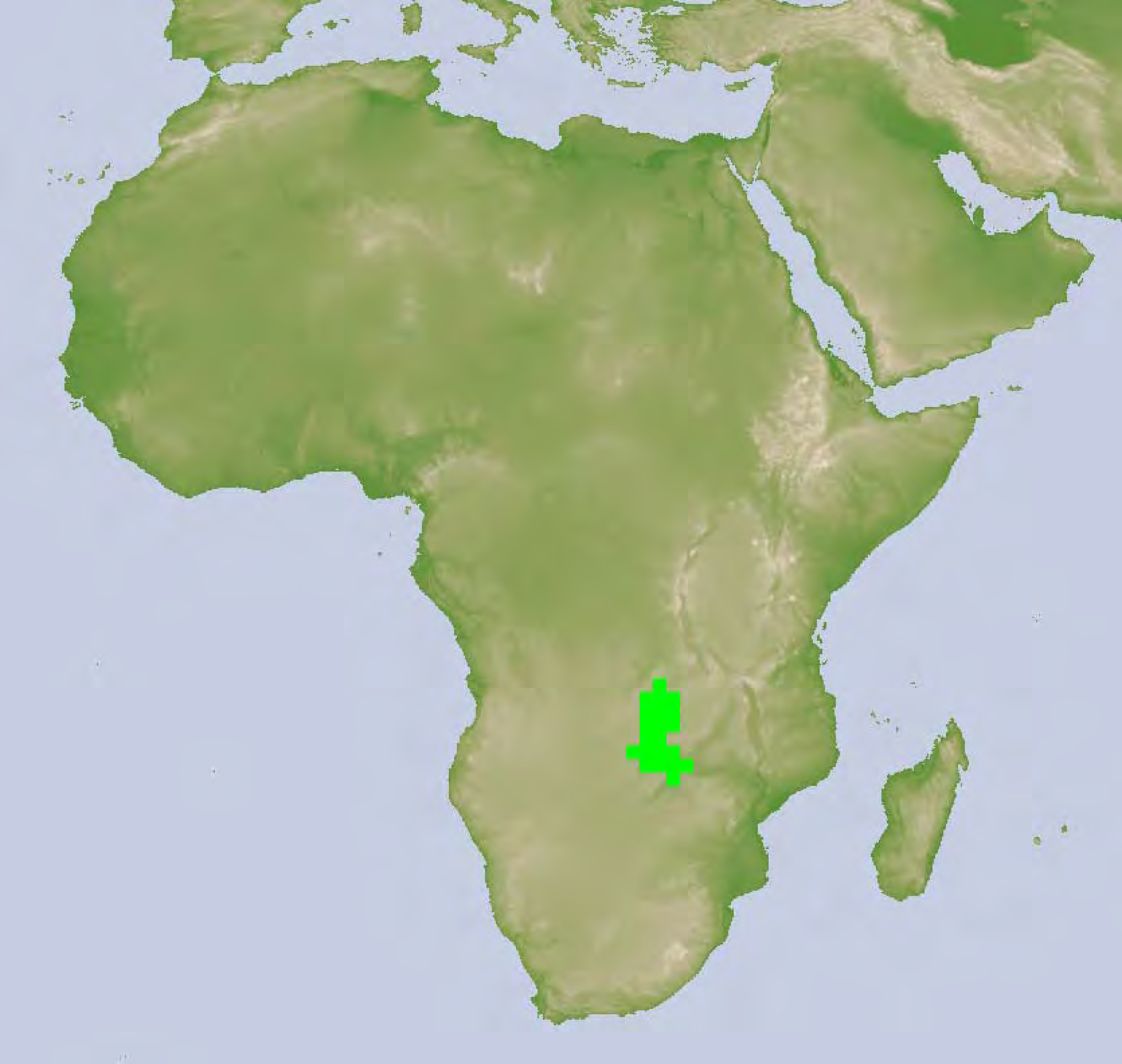

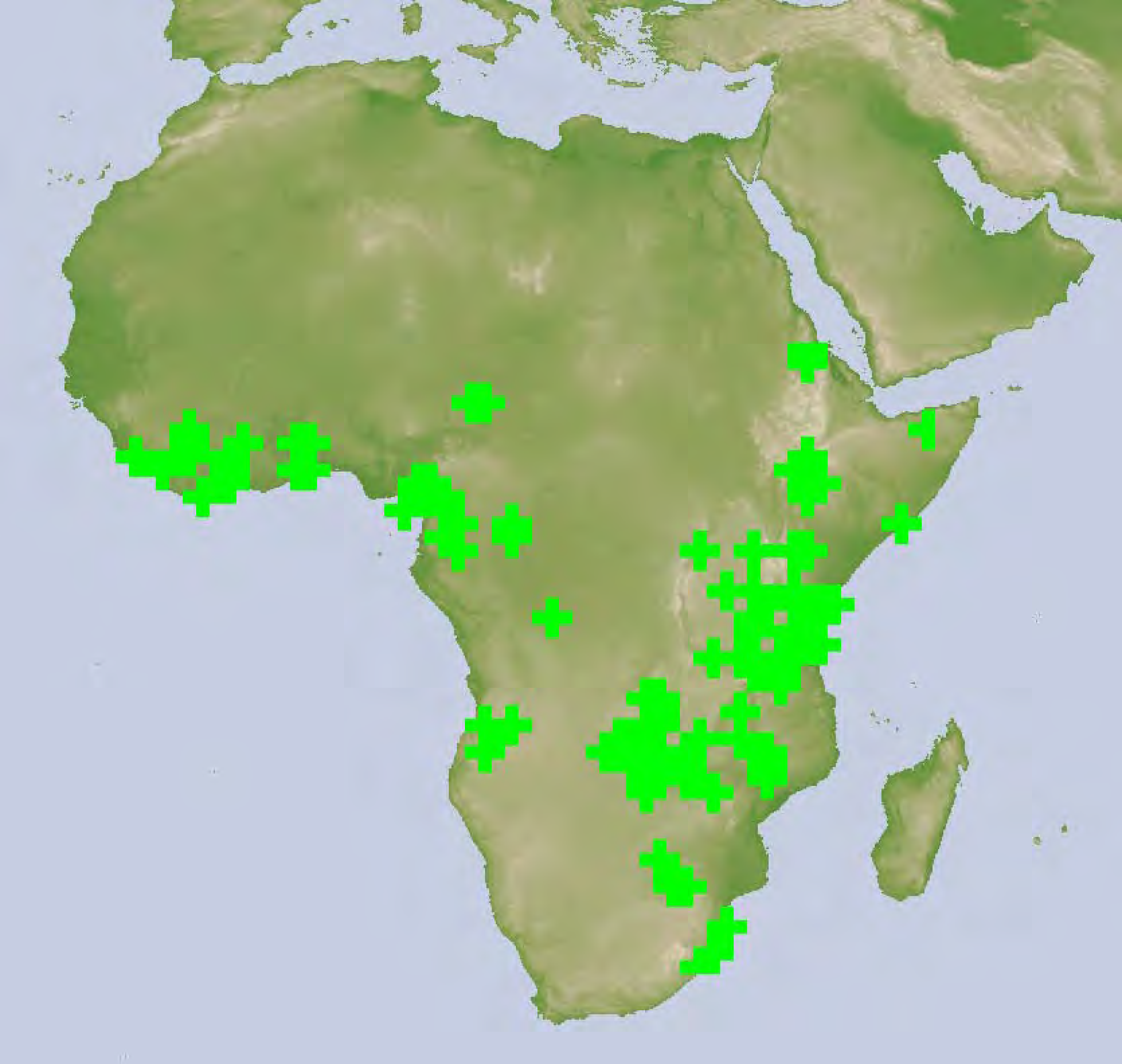

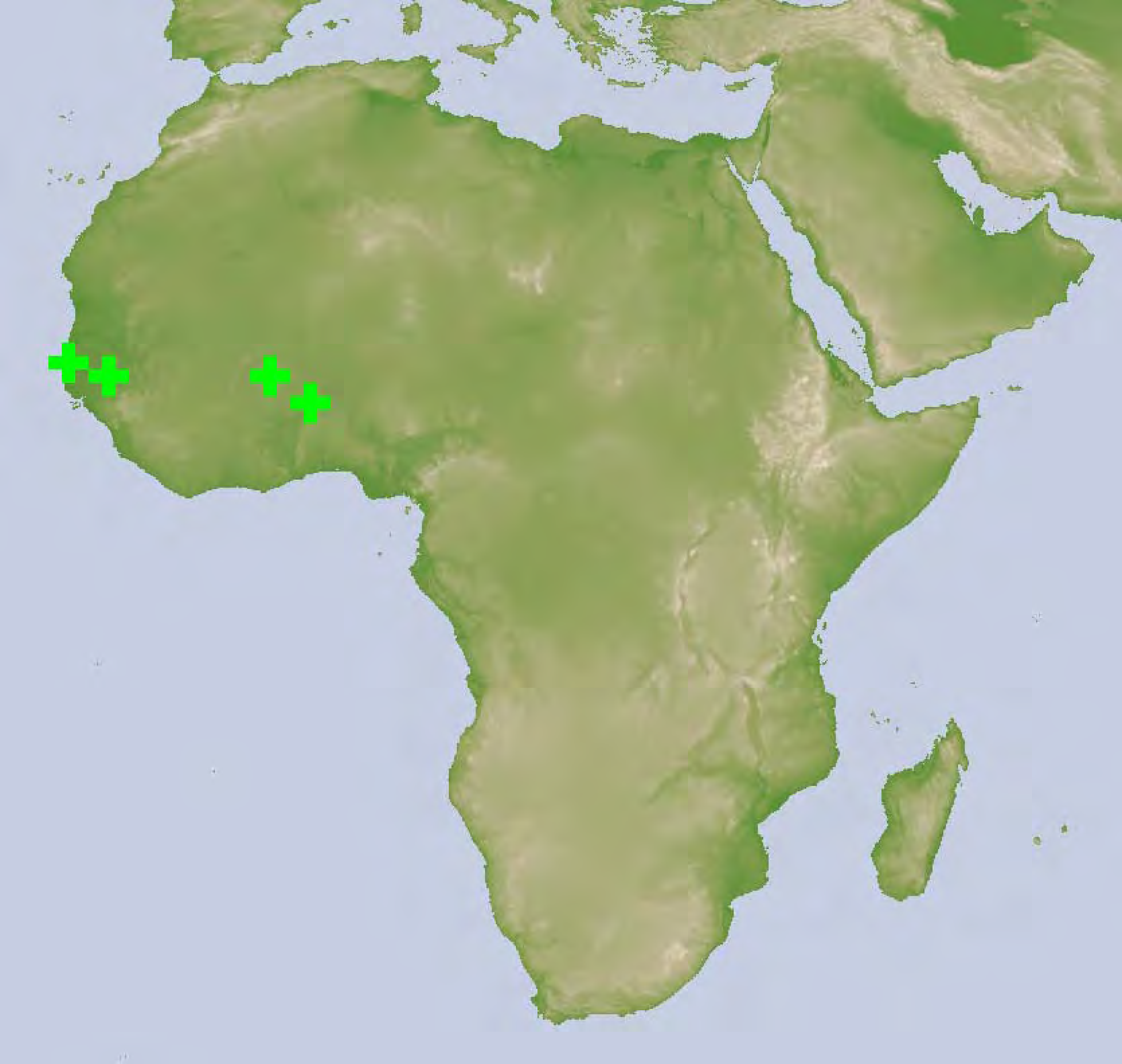

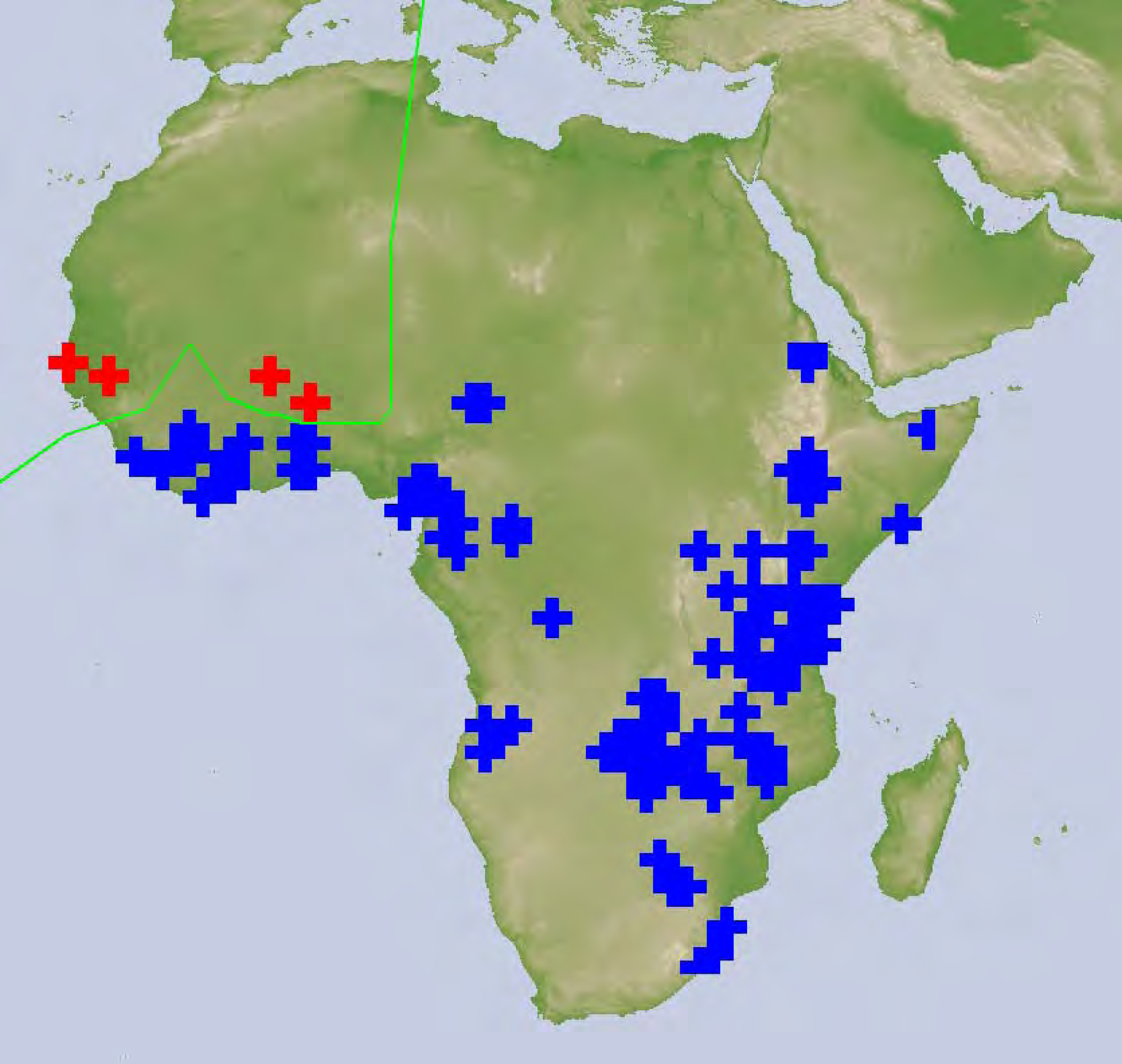

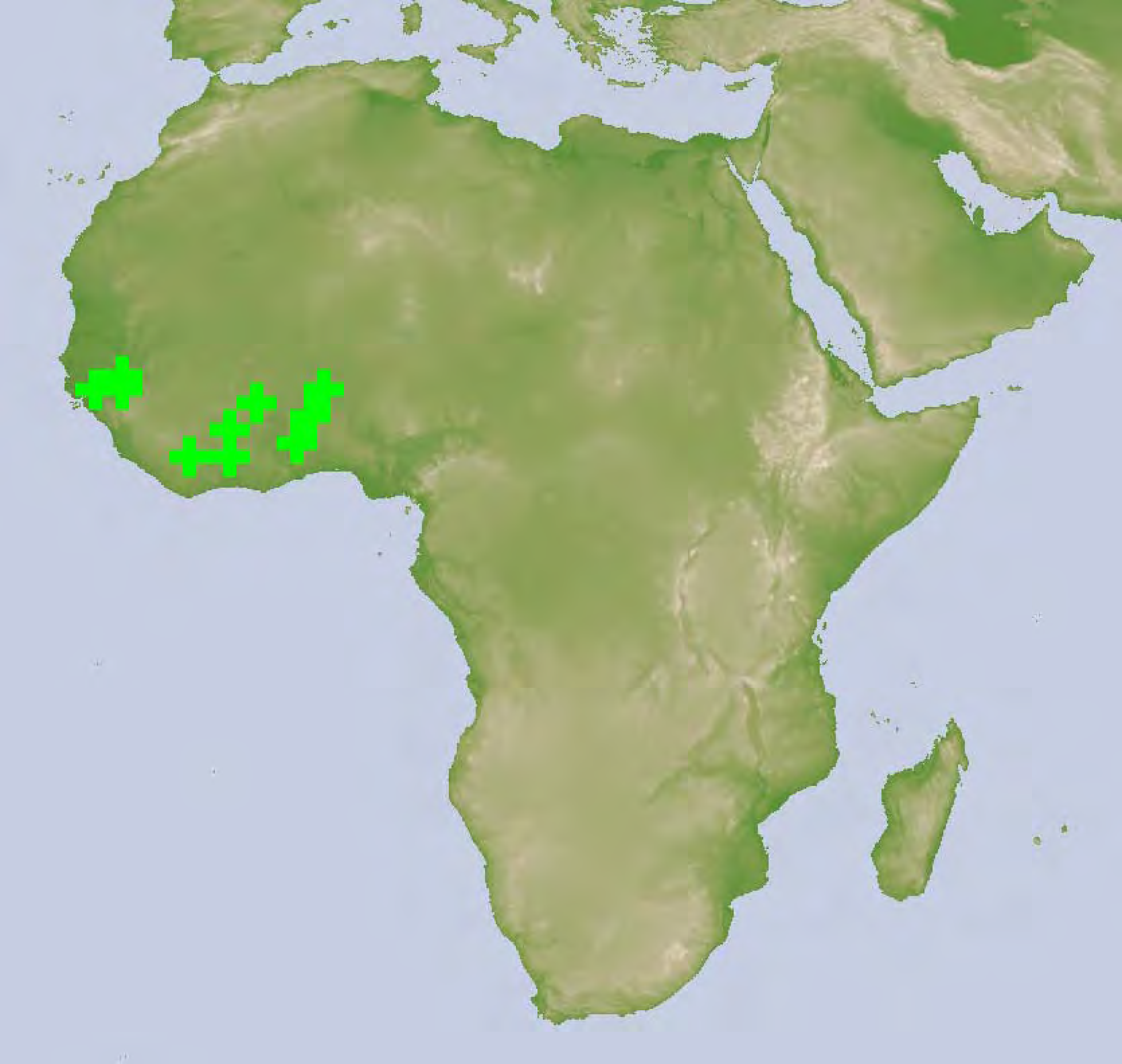

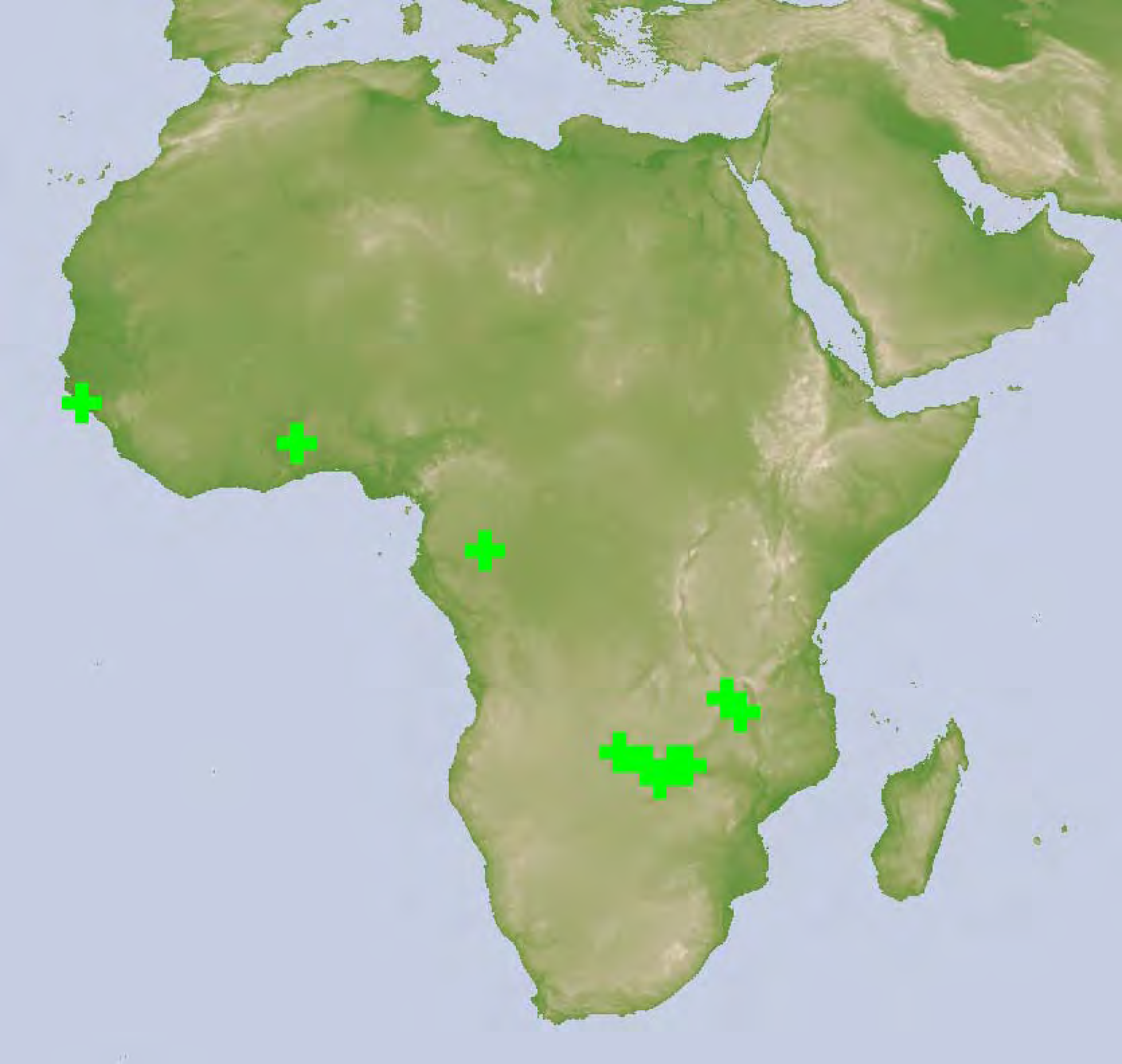

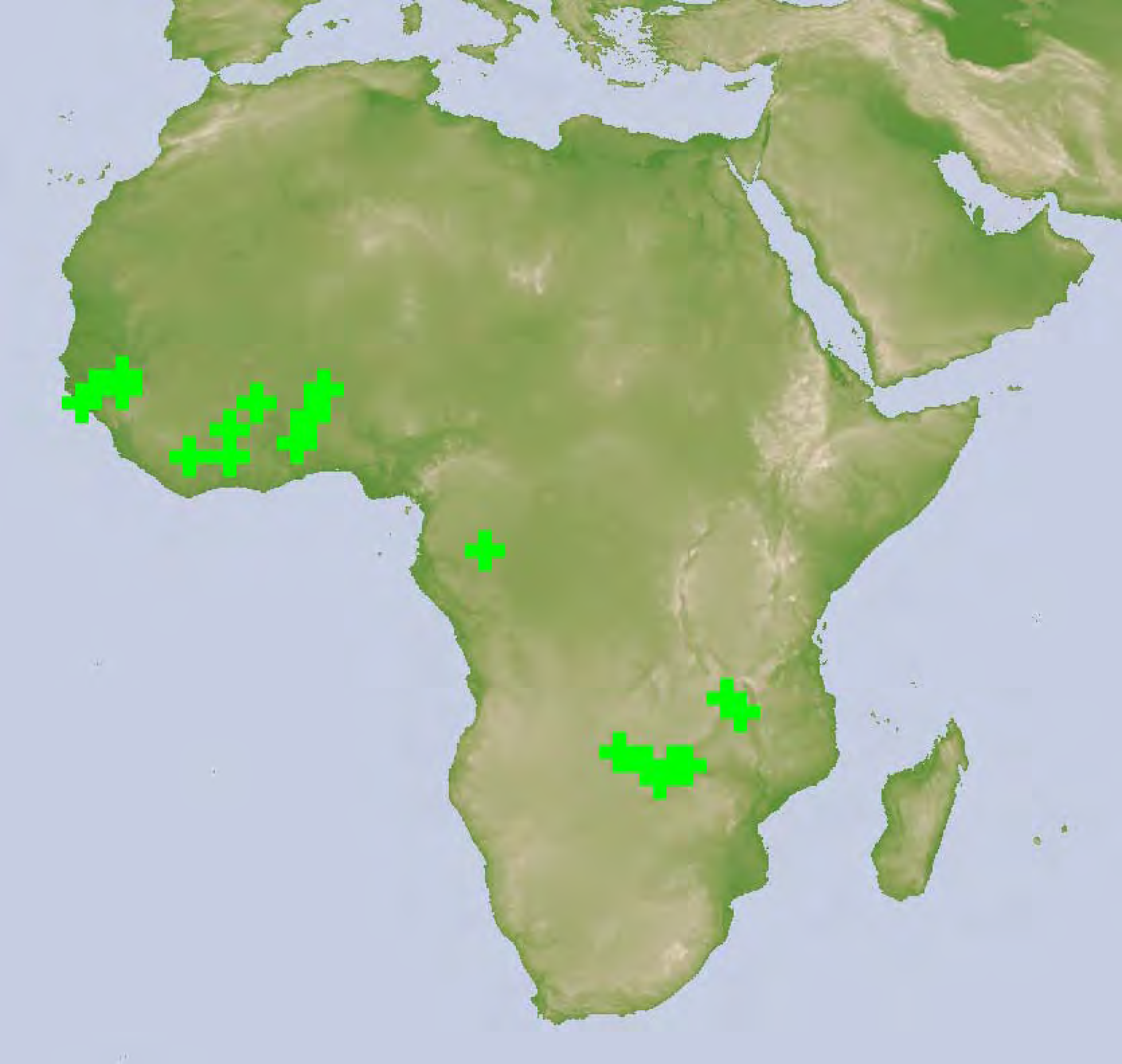

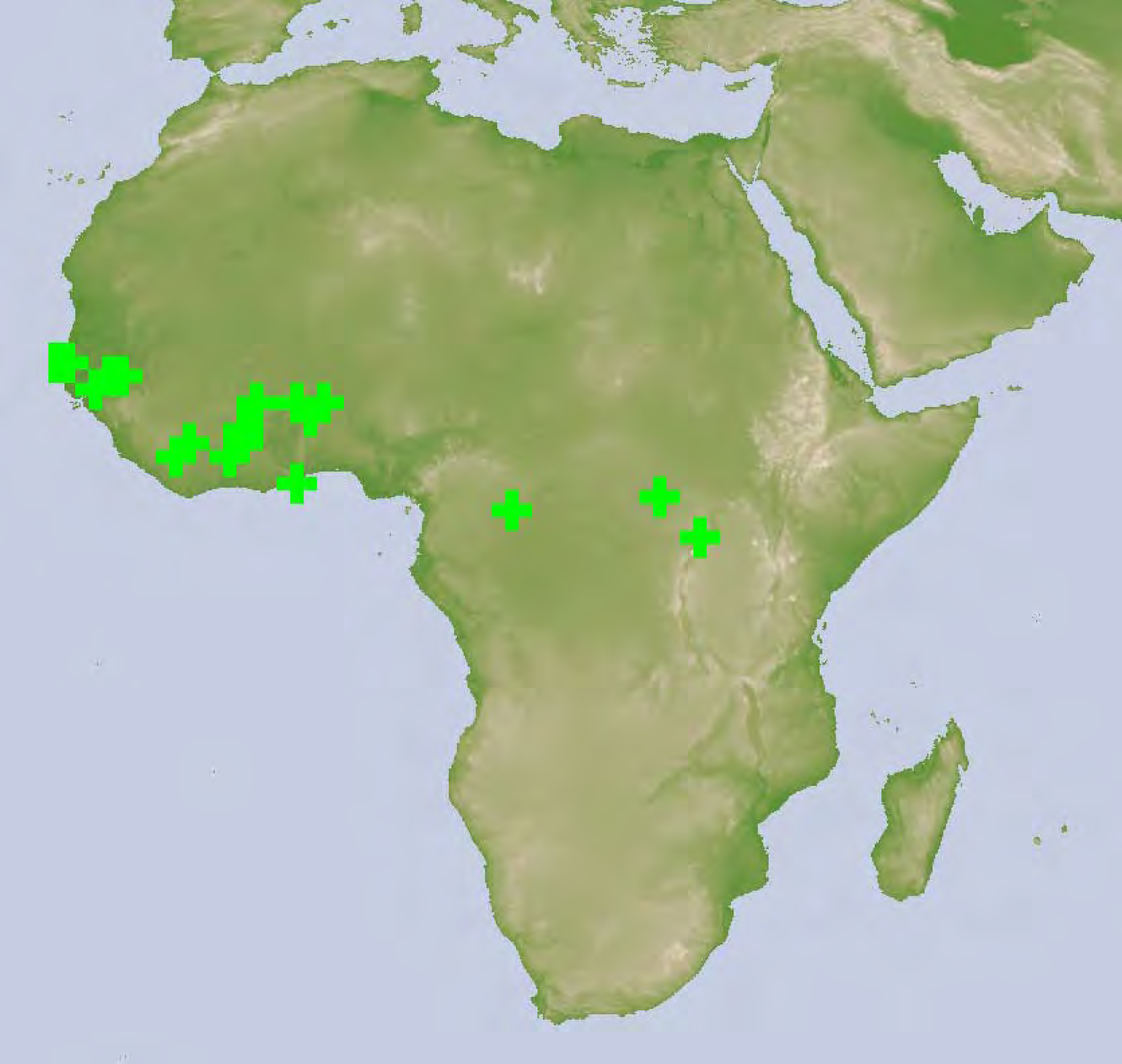

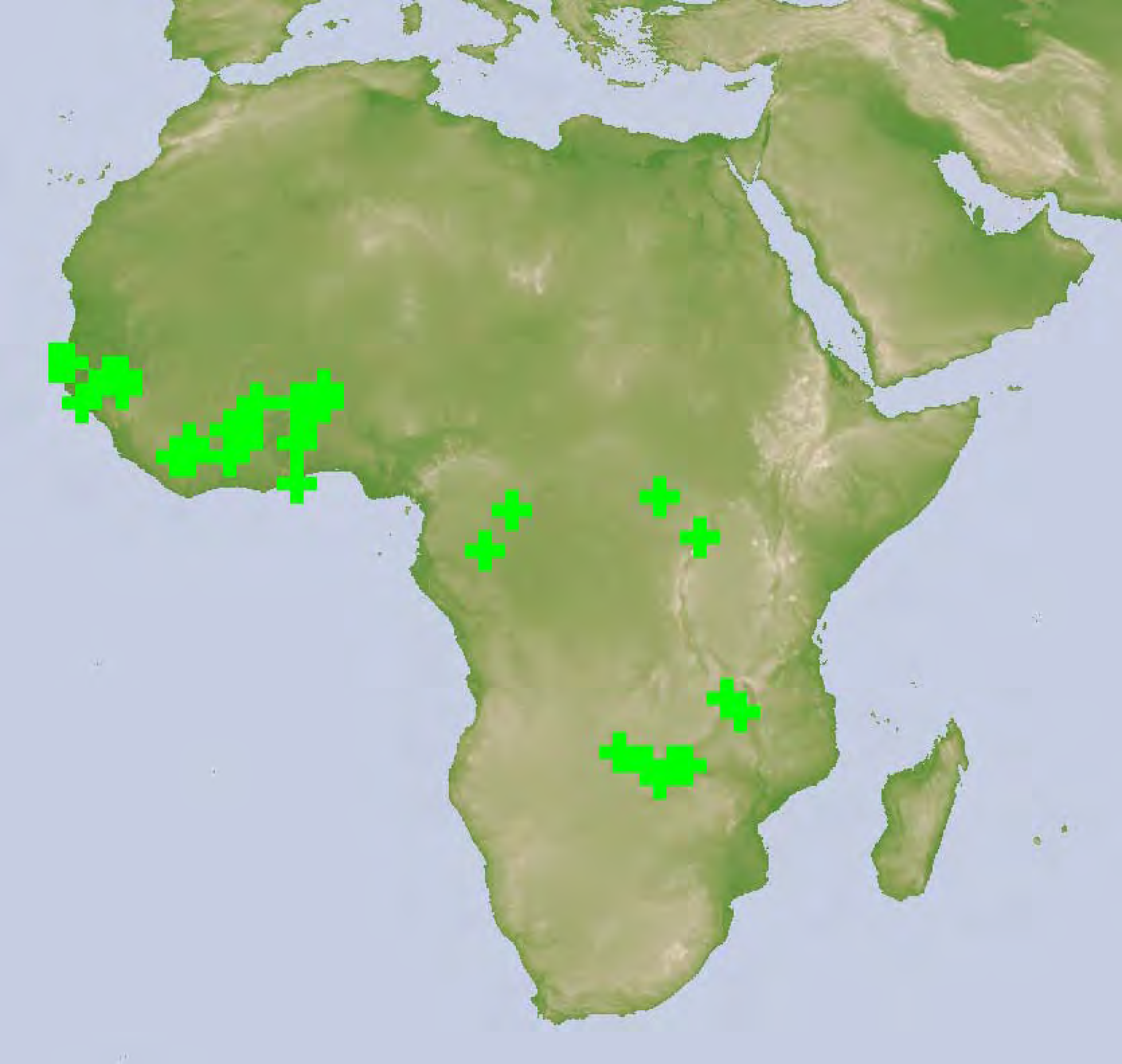

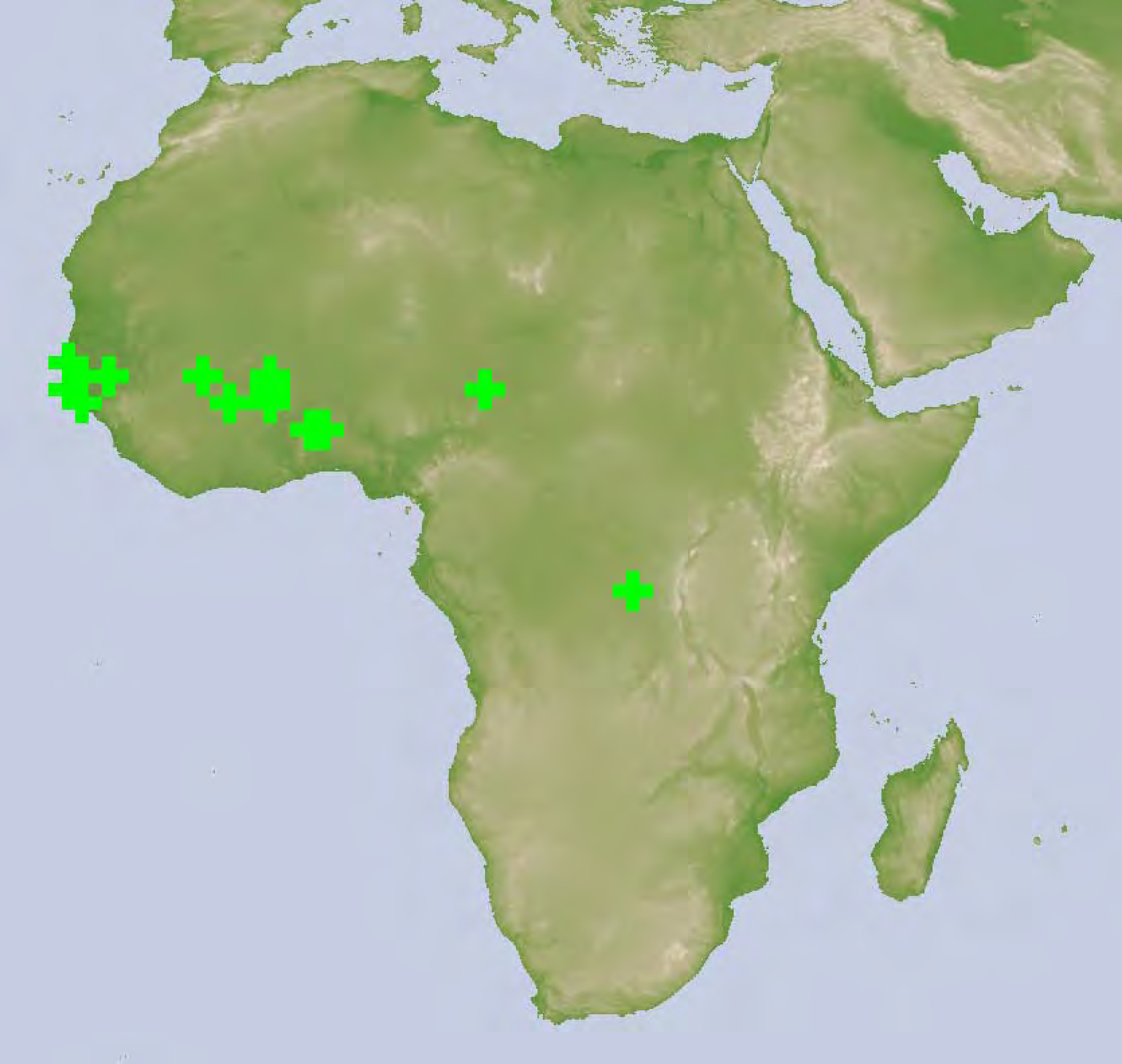

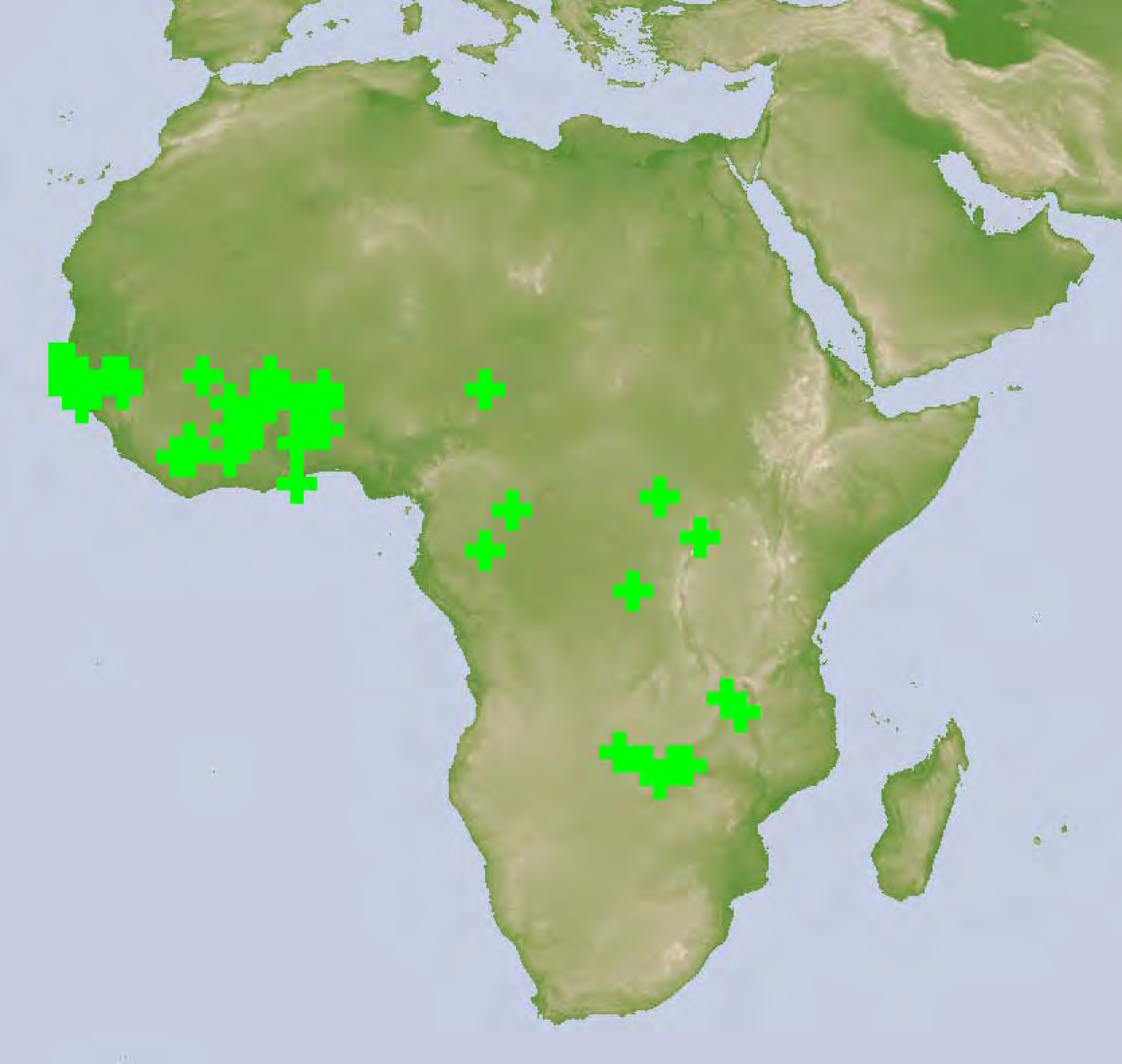

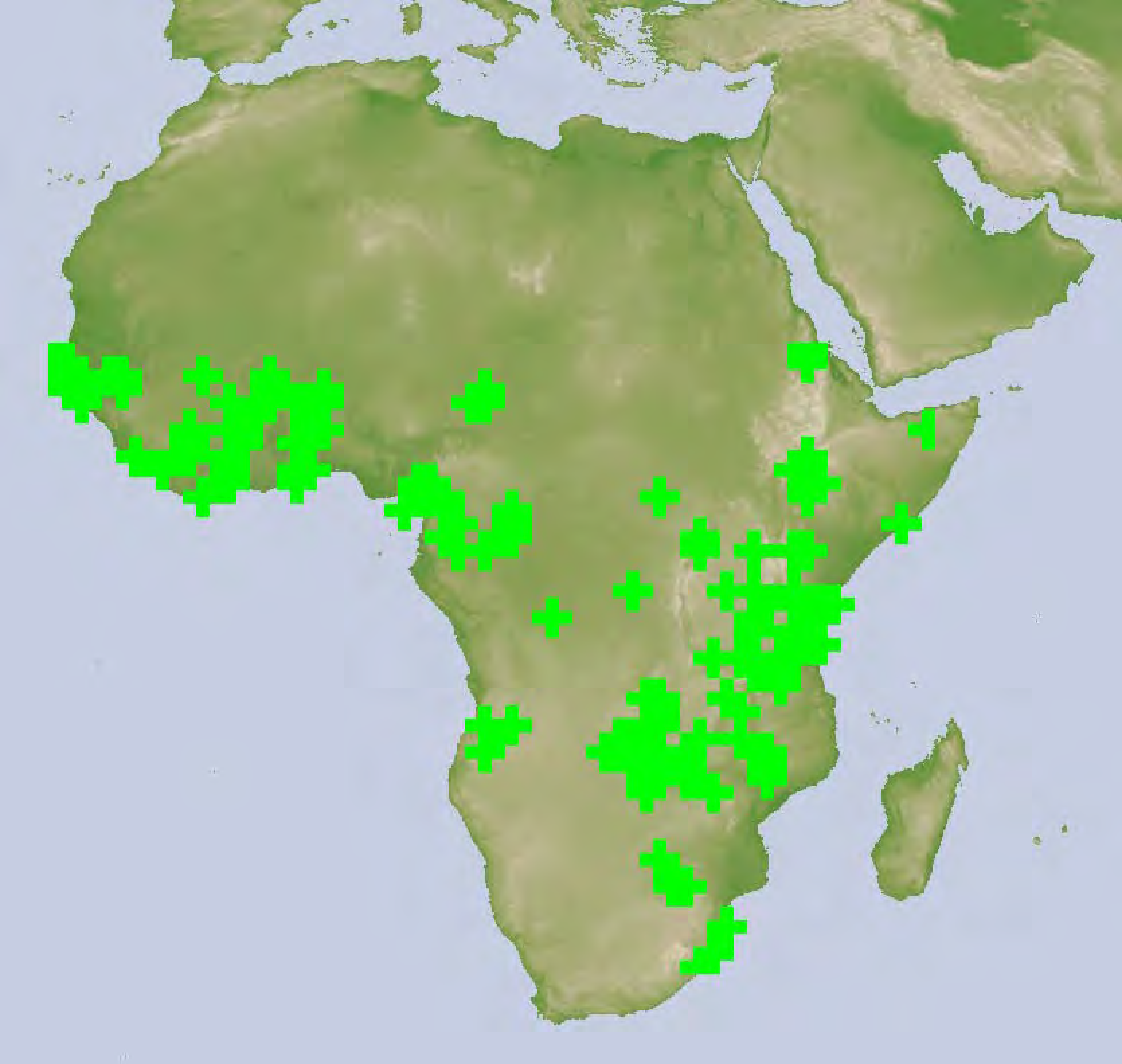

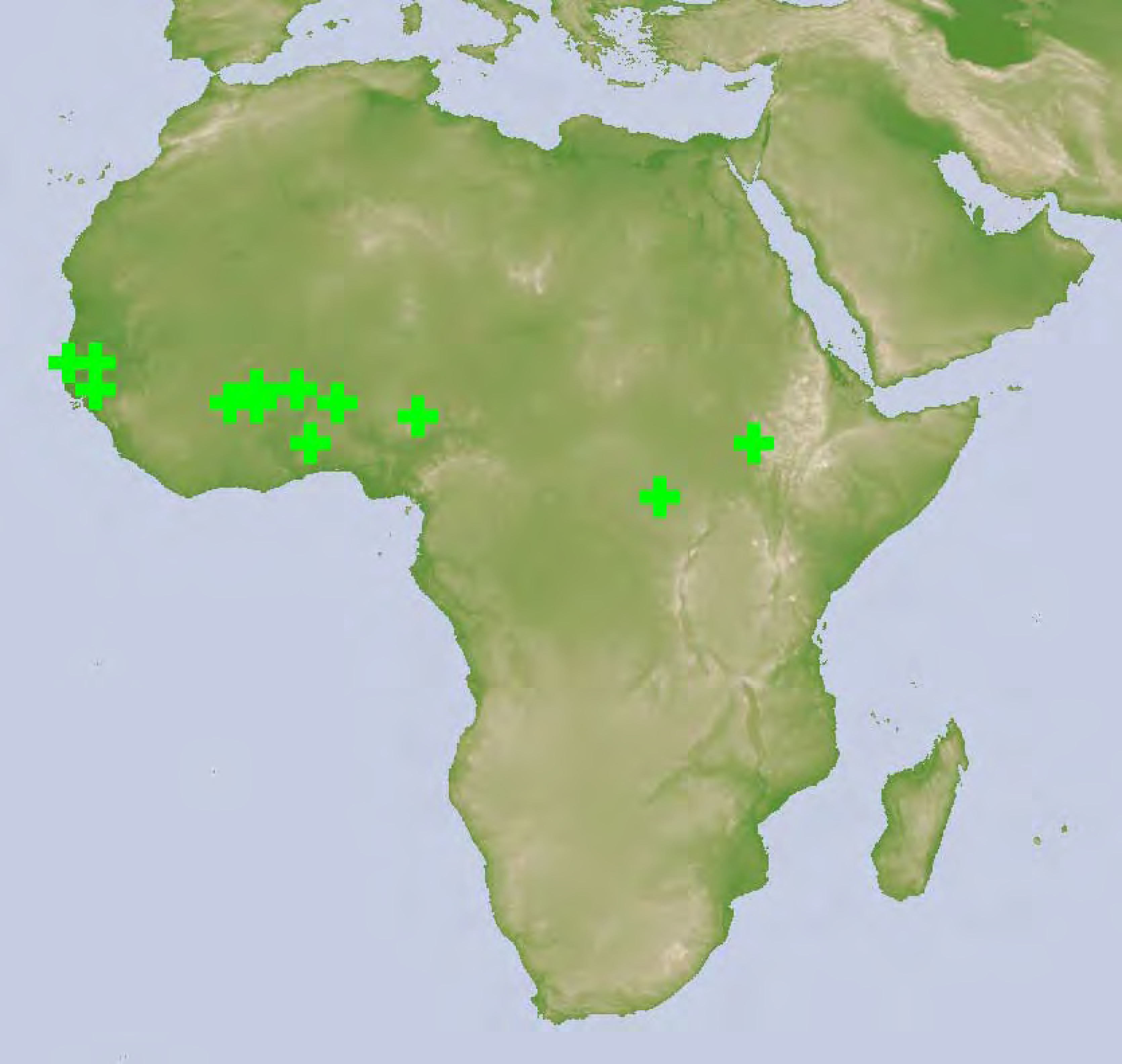

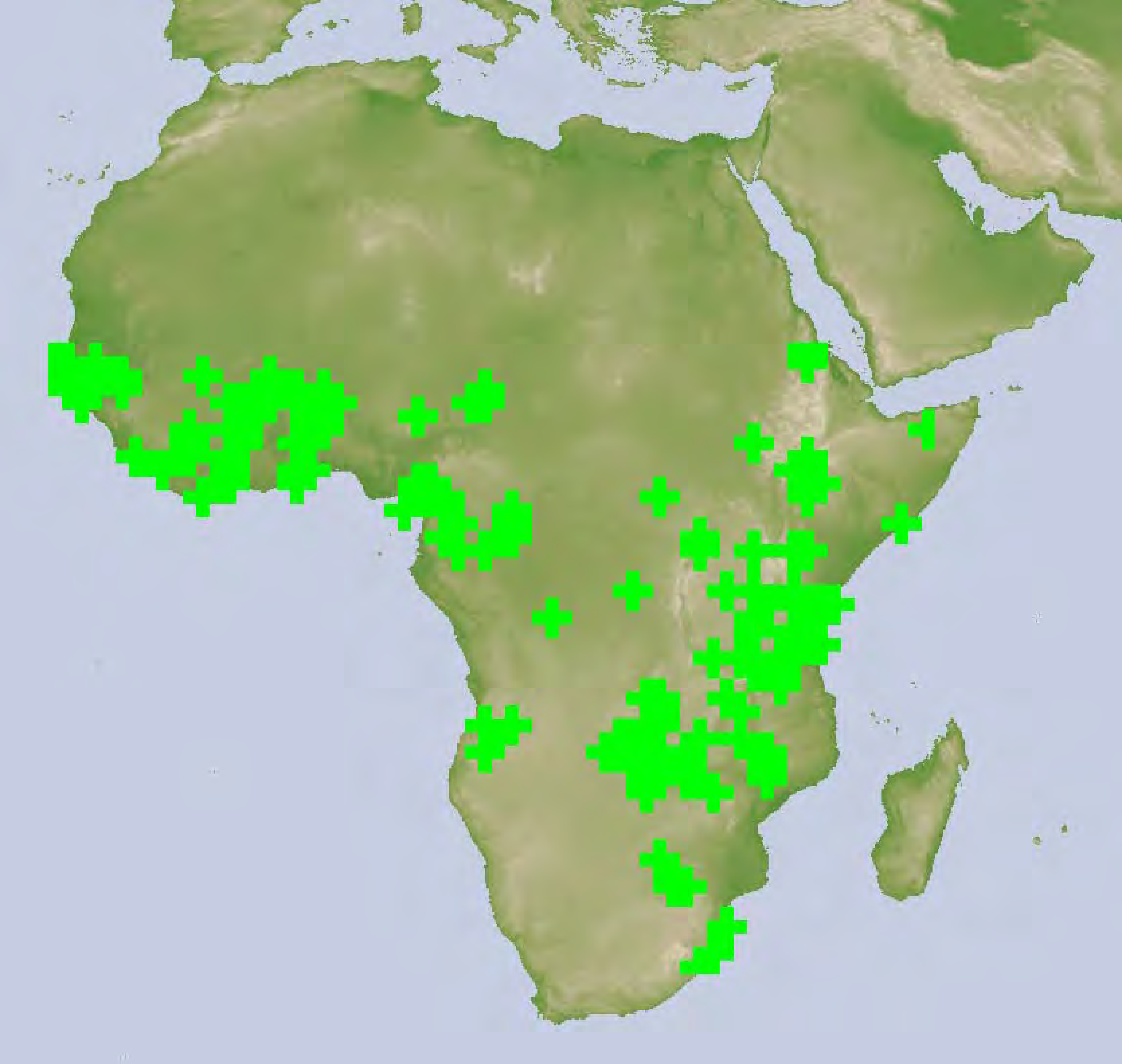

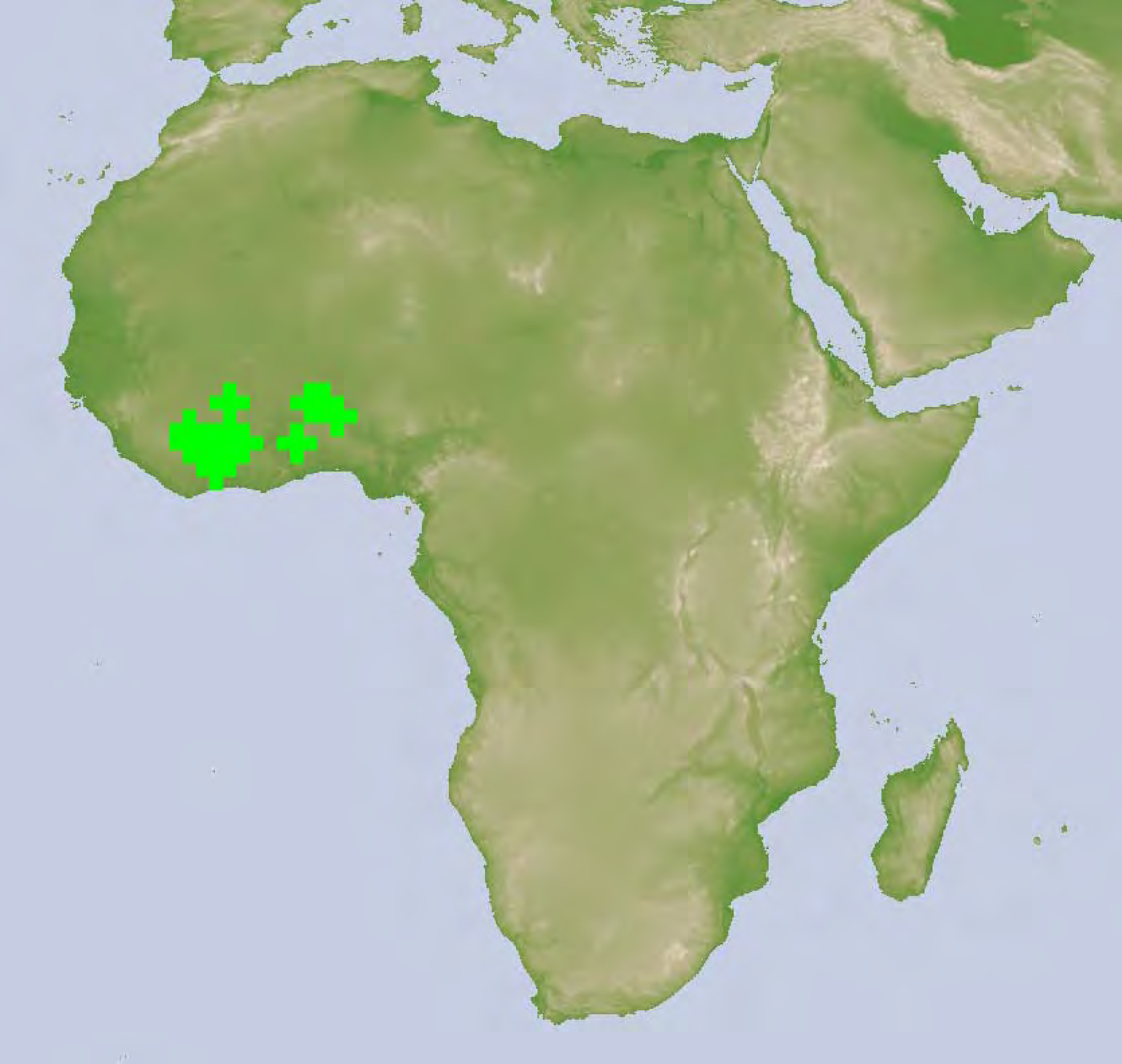

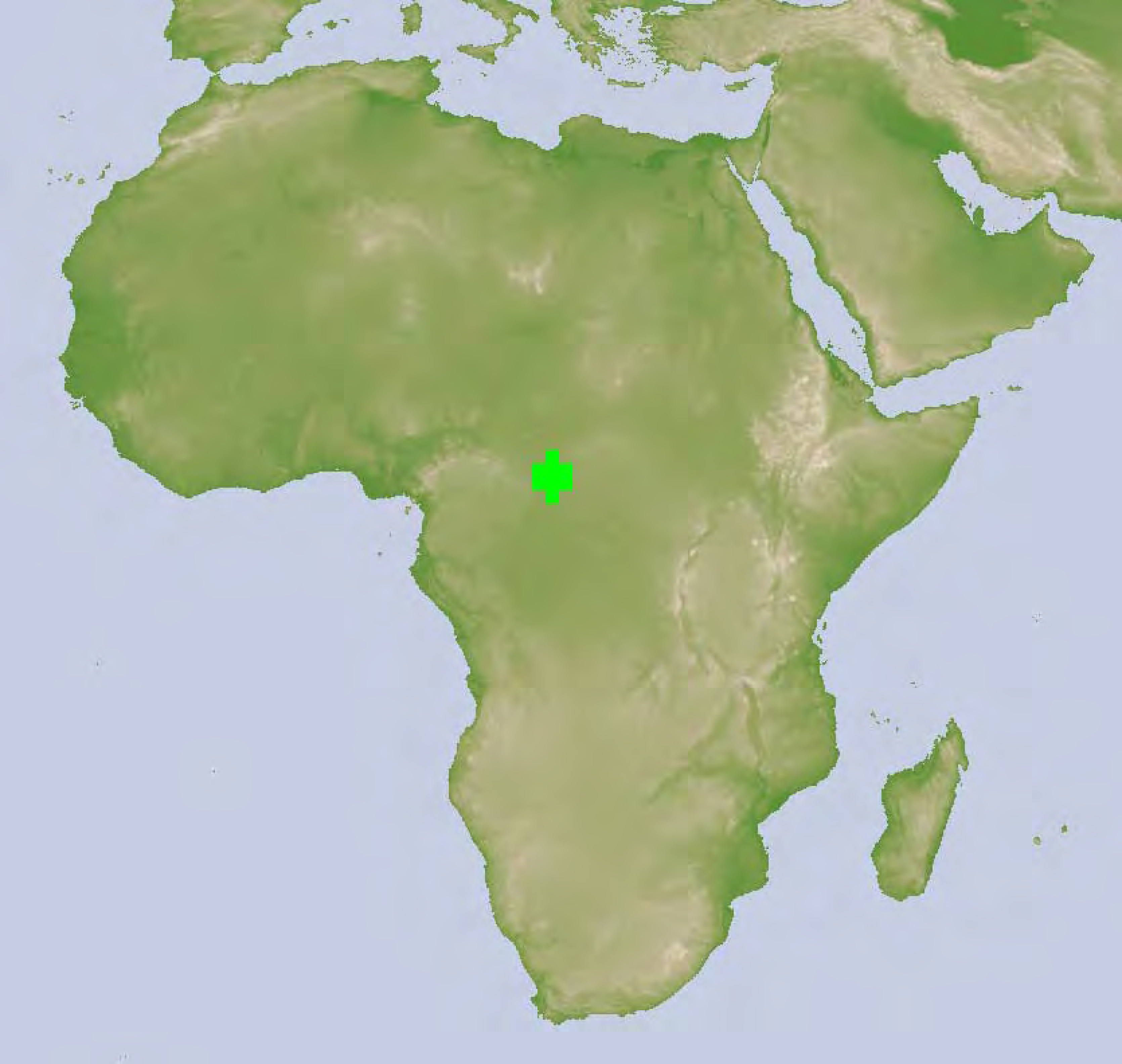

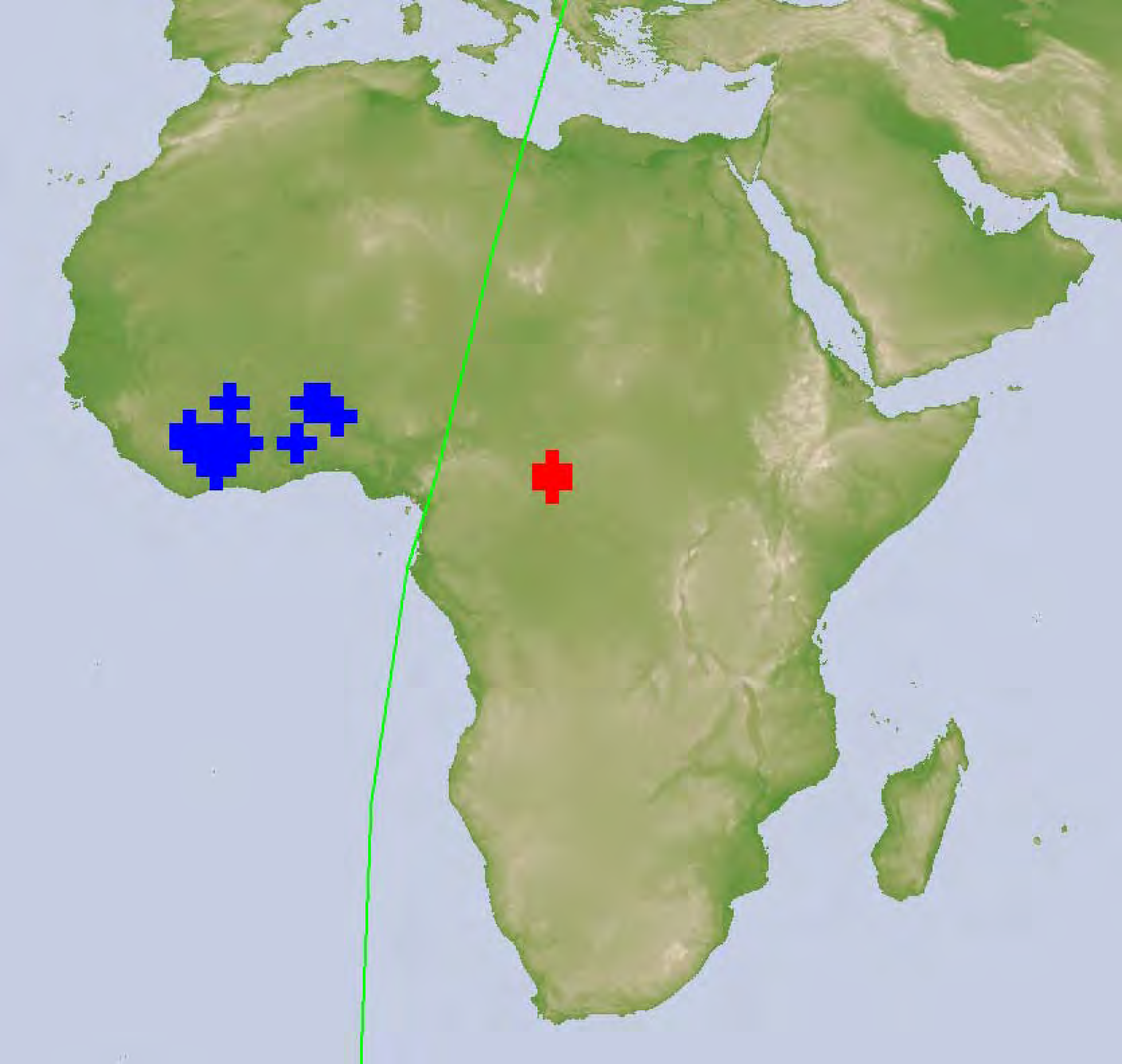

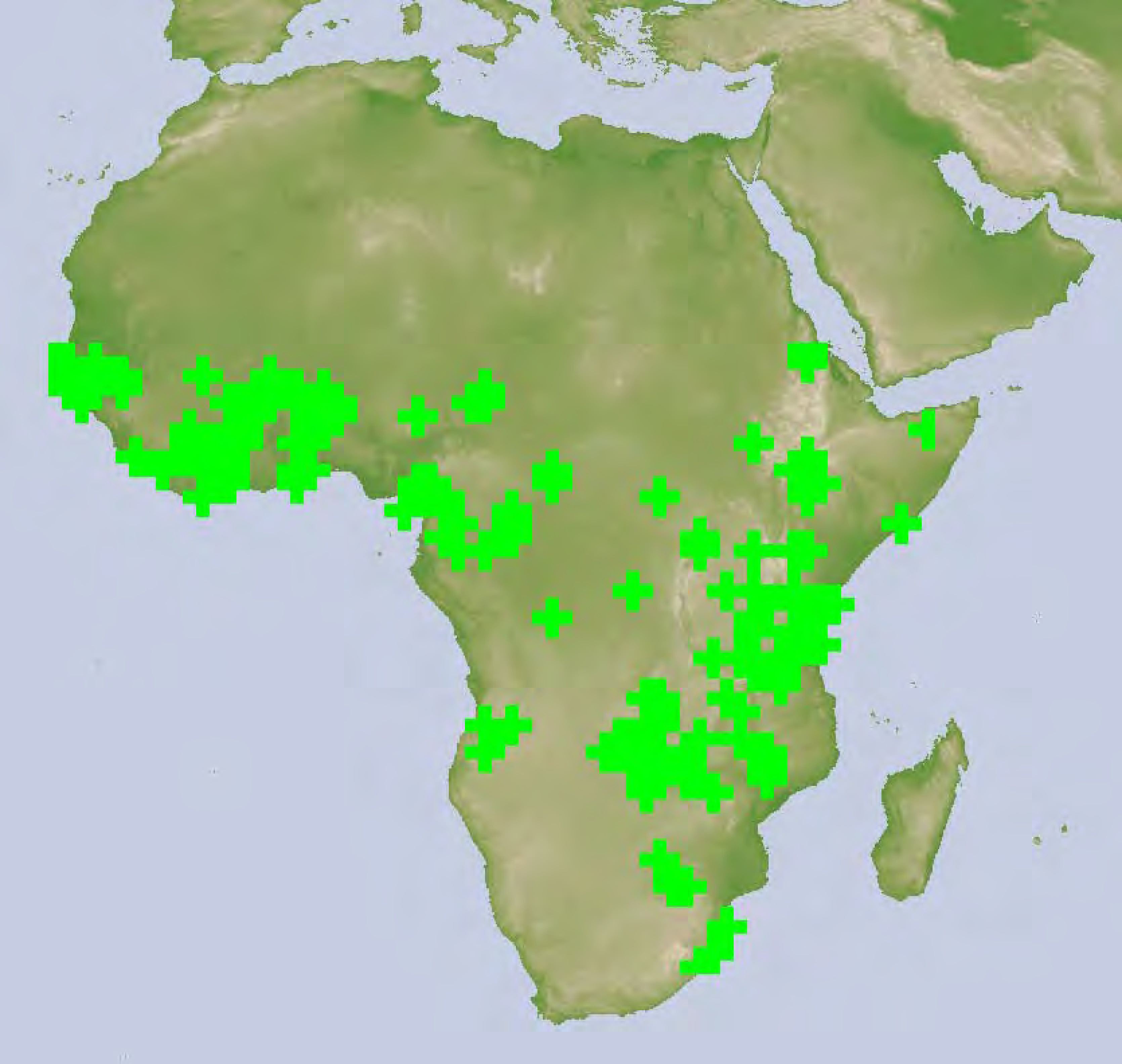

Consensus reconstruction

node_1 [pseudoliberianus]
node_2 [liberianus]
node_3 [macroliberianus]
node_4
node_5
node_6 [rufopygus]
node_7 [jossoi]
node_8 [saadaniensis]
node_9
node_10
node_11
node_12 [schaufussi]
node_13 [rolandoi]
node_14
node_15 [zavattarii]
node_16
node_17 [angolensis]
node_18
node_19 [rufostillans]
node_20
node_21 [aequatus]
node_22
node_23
node_24 [rufobasalis]
node_25 [ebenus]
node_26
node_27 [naevius]
node_28
node_29 [zambesianus]
node_30
node_31
node_32 [delahayei]
node_33
node_34 [katangensis]
node_35
node_36 [pendjarius]
node_37
node_38 [rougonorum]
node_39 [curtipilis]
node_40
node_41 [flexicornis]
node_42
node_43 [viridiaereus]
node_44
node_45
node_46 [chrysoderus]
node_47
node_48 [lamtoensis]
node_49 [hemichlorus]
node_50
node_51

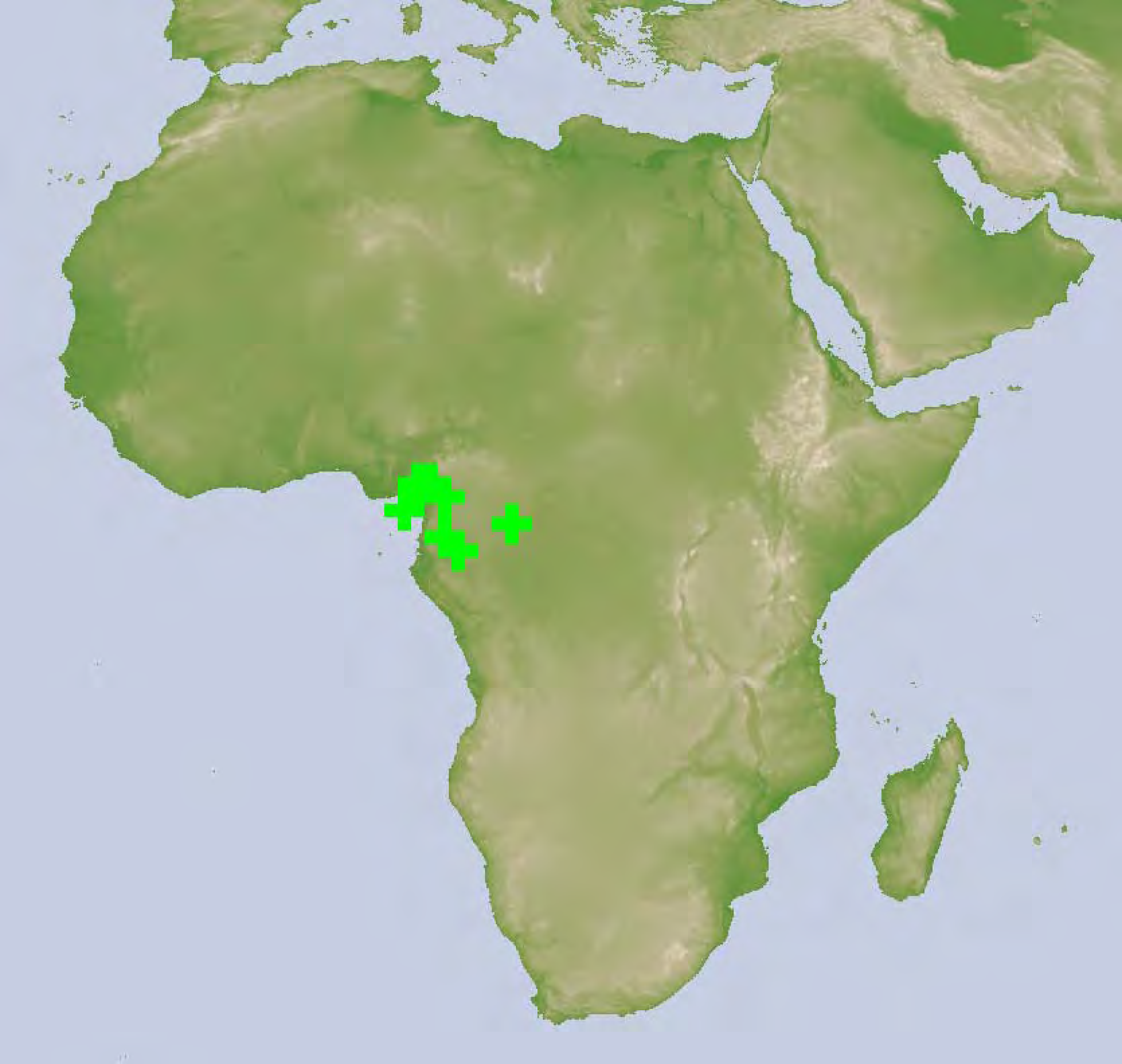

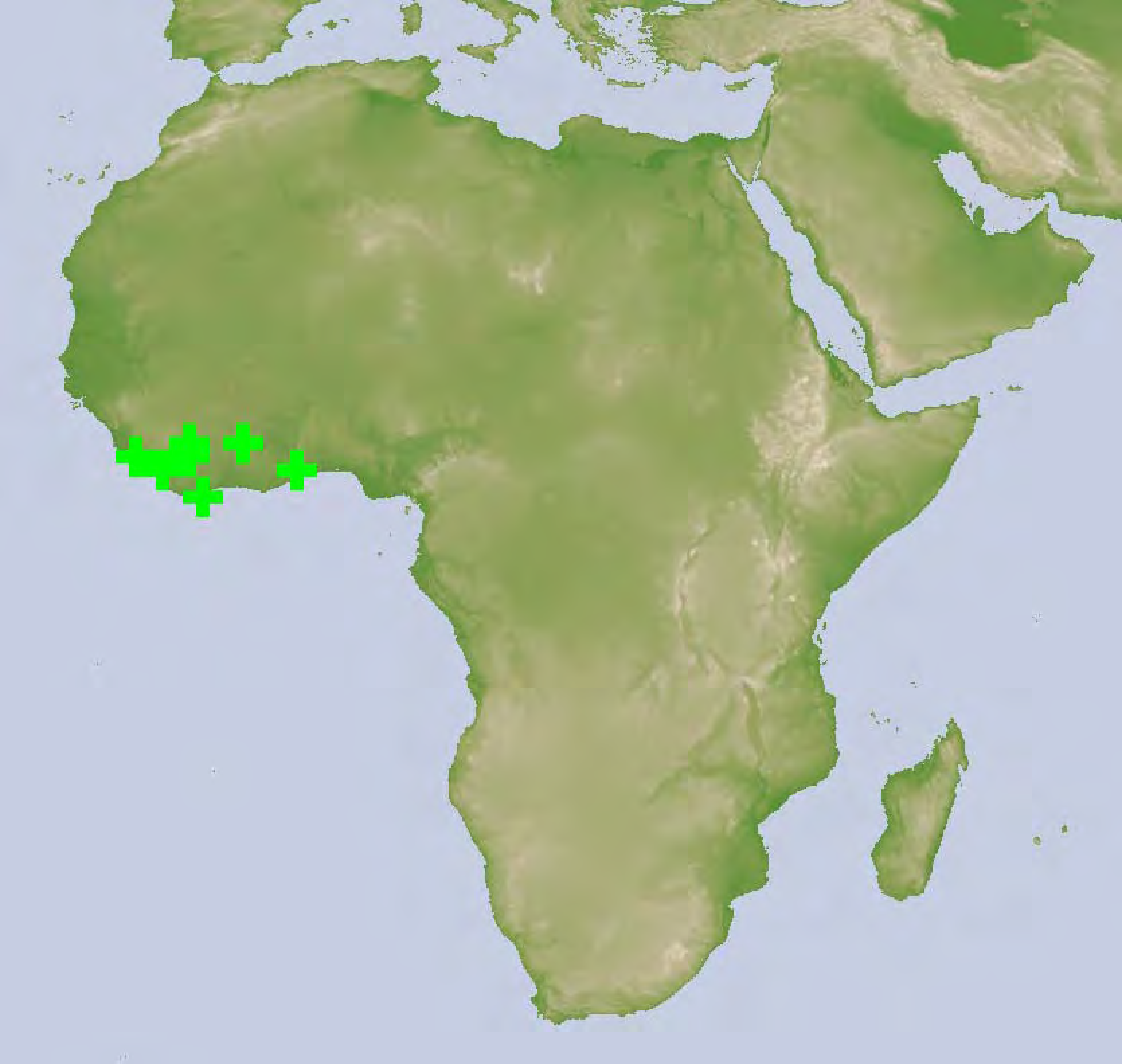

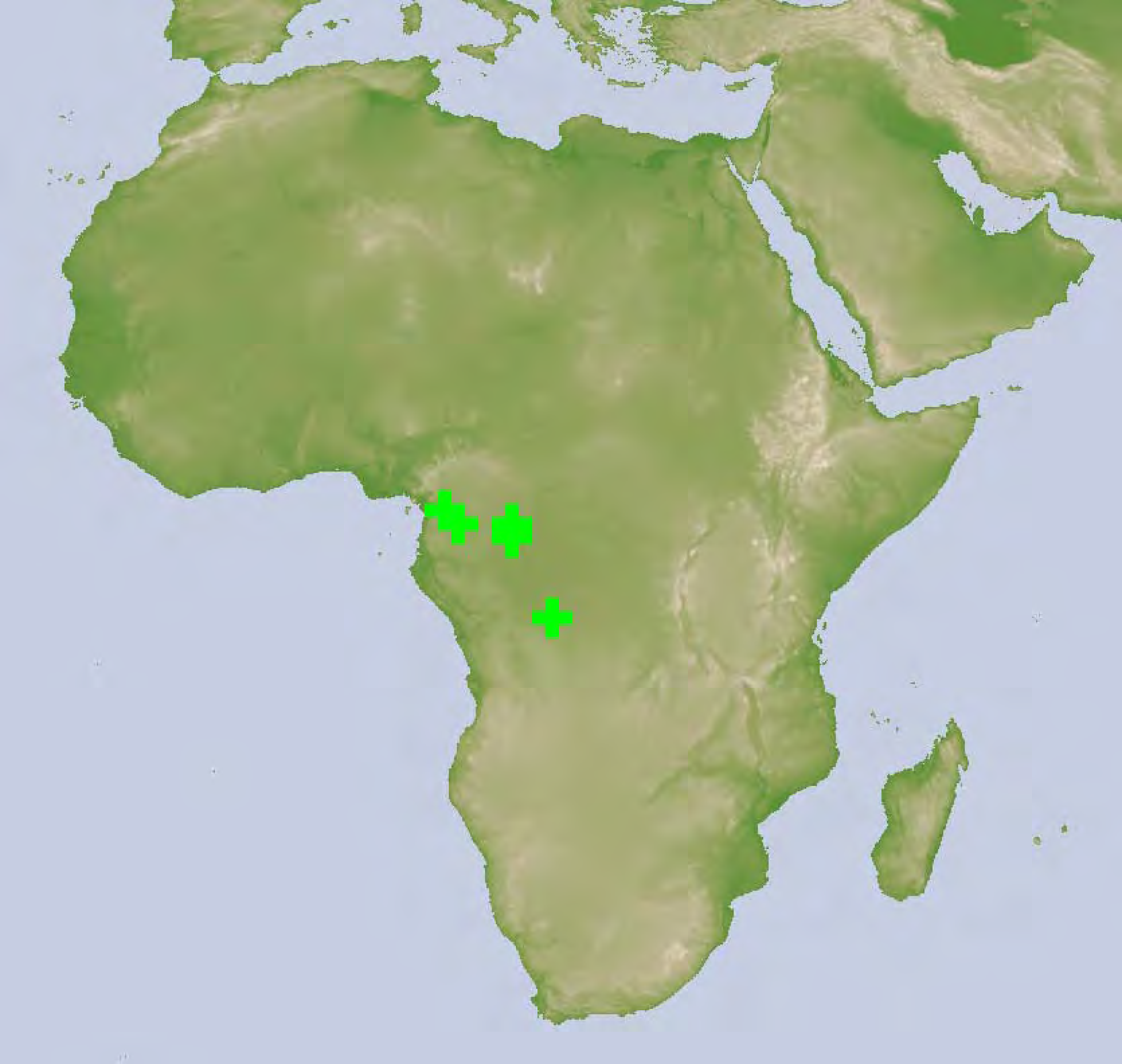

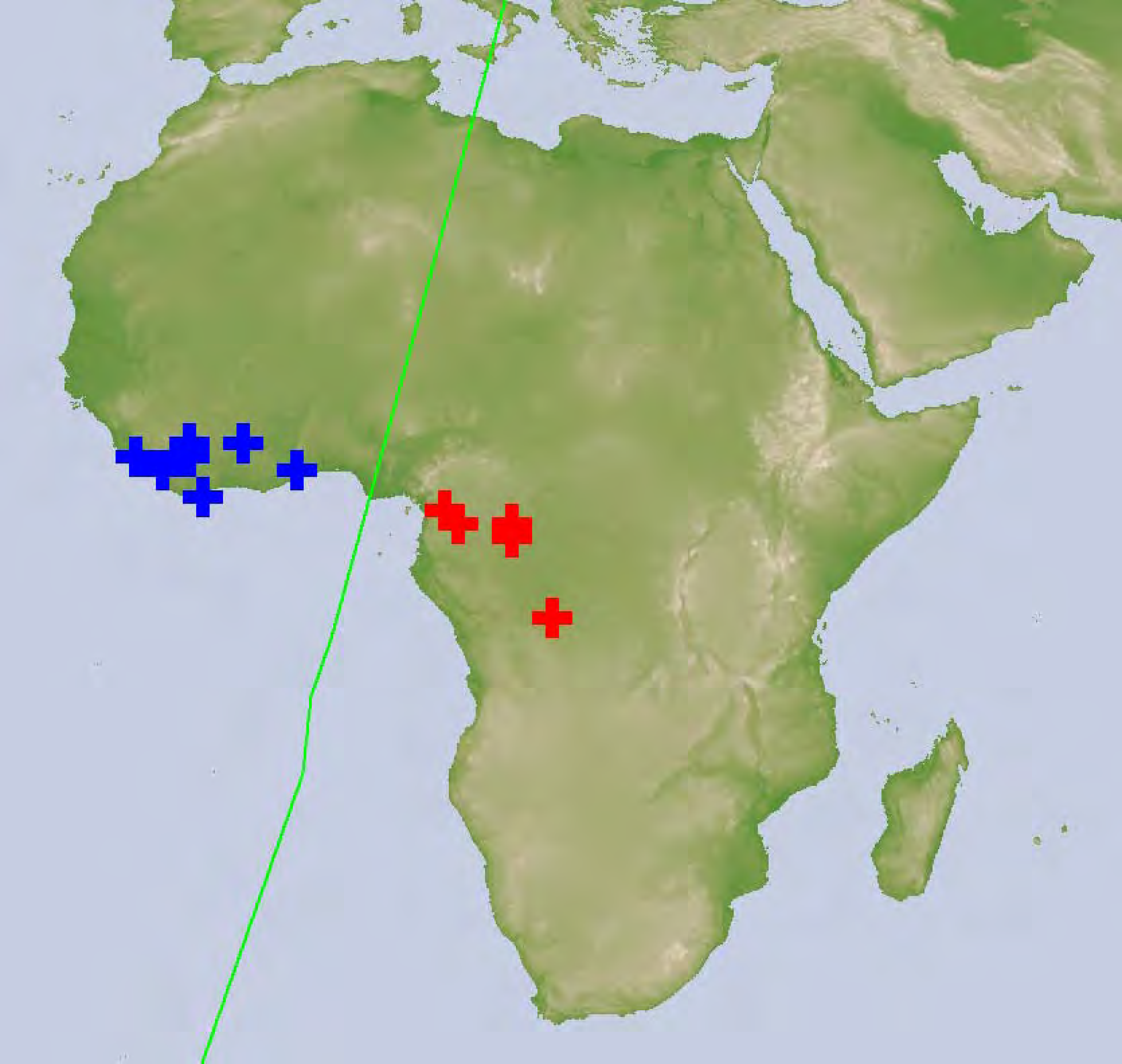

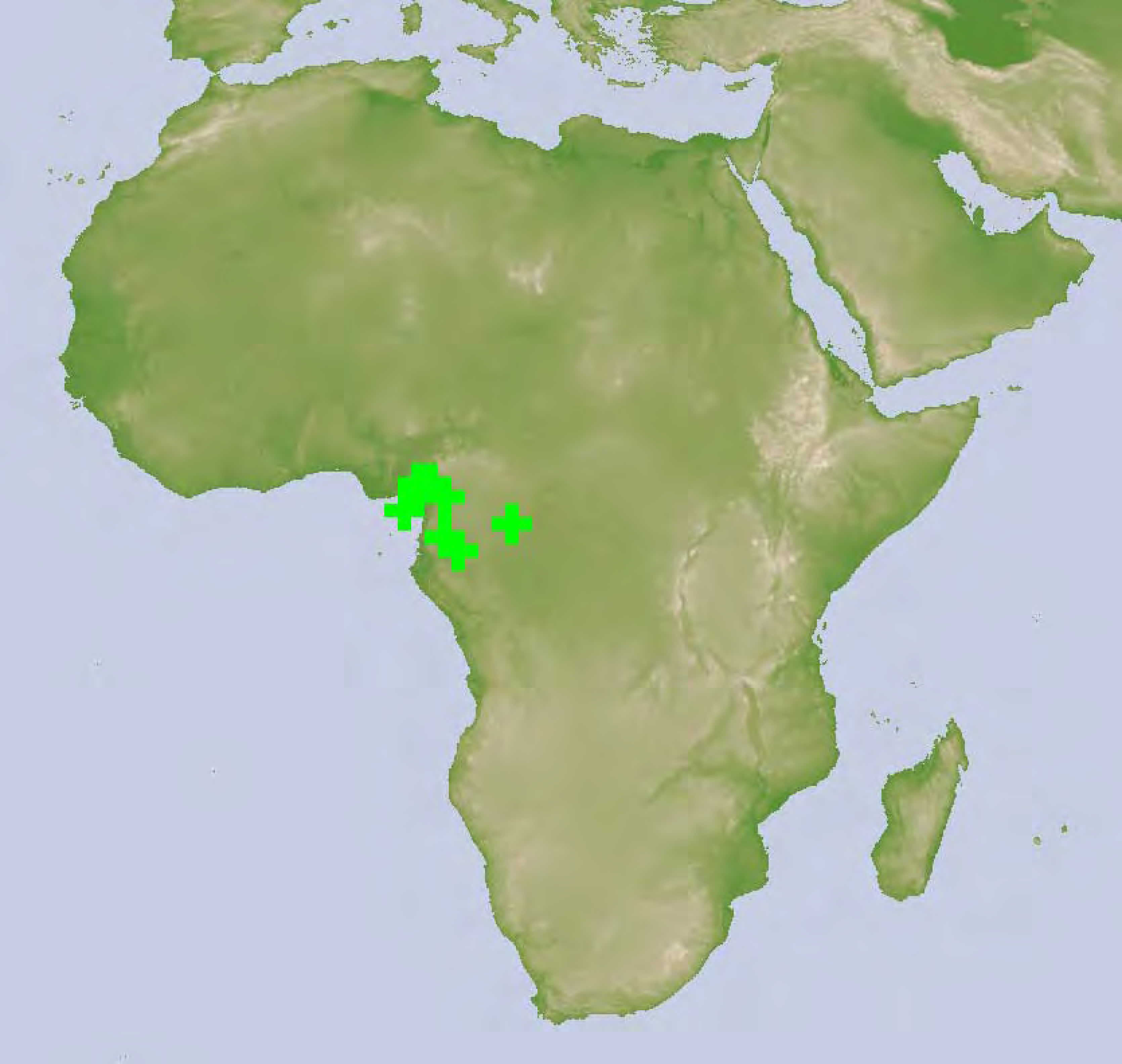

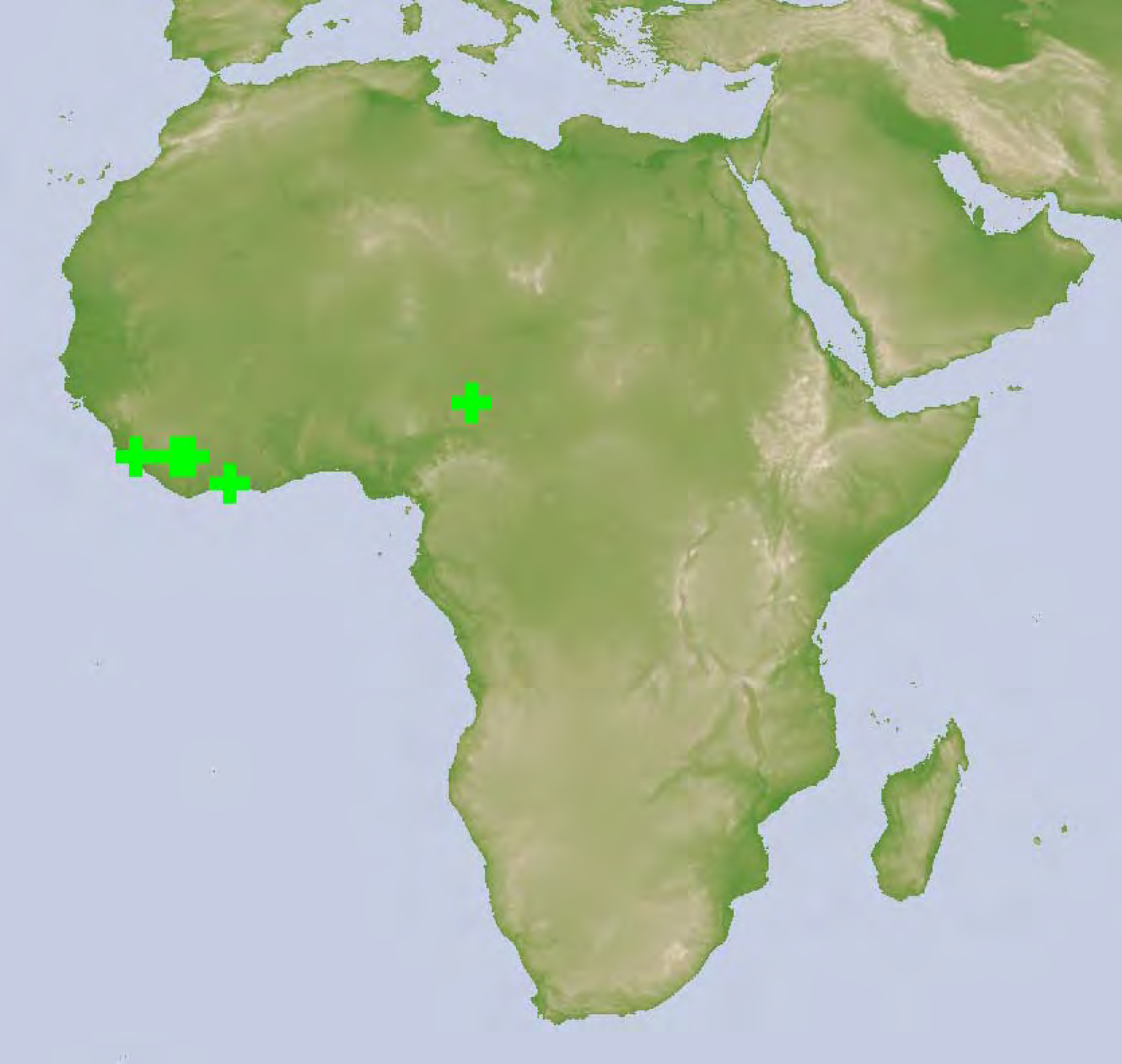

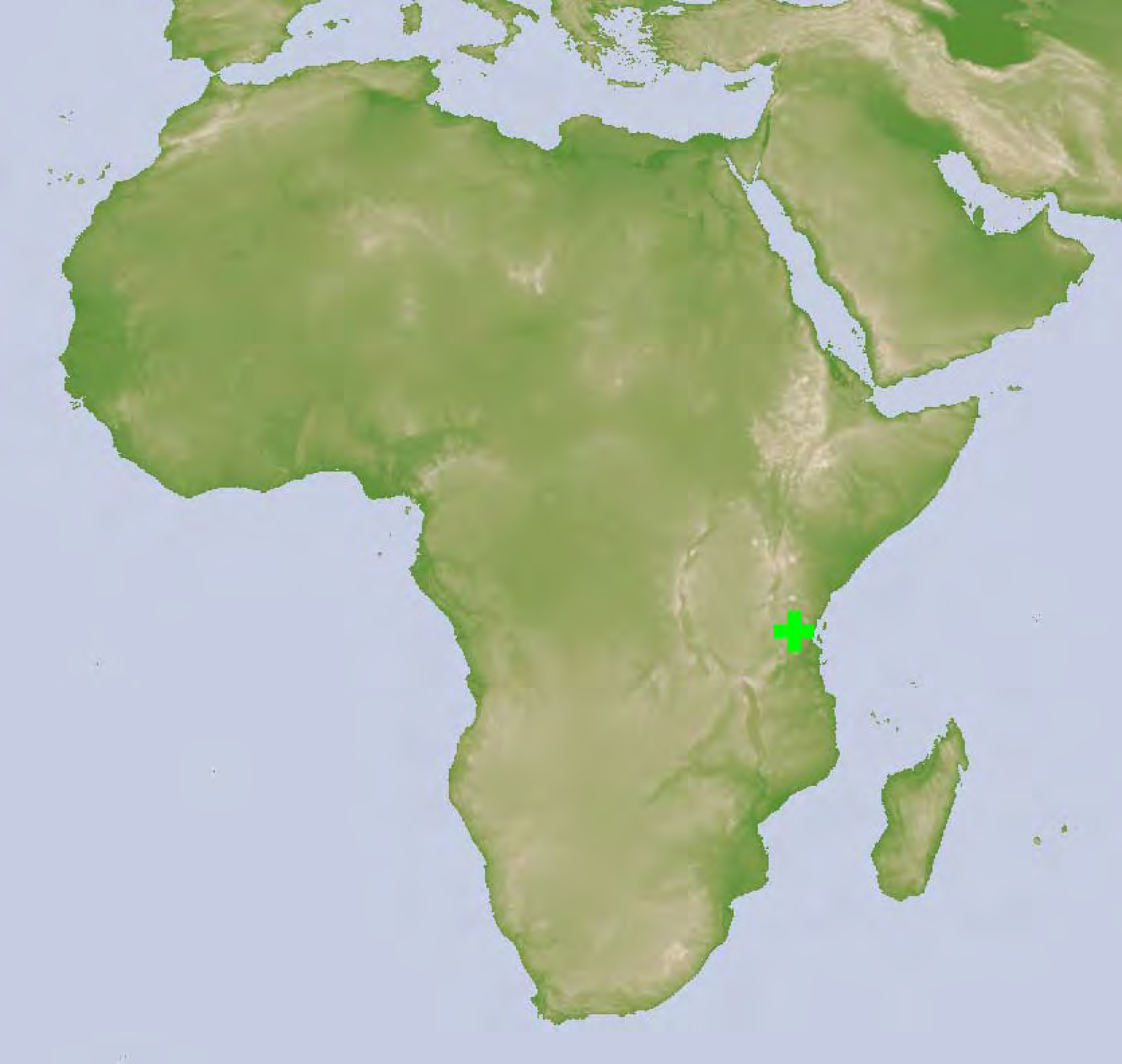

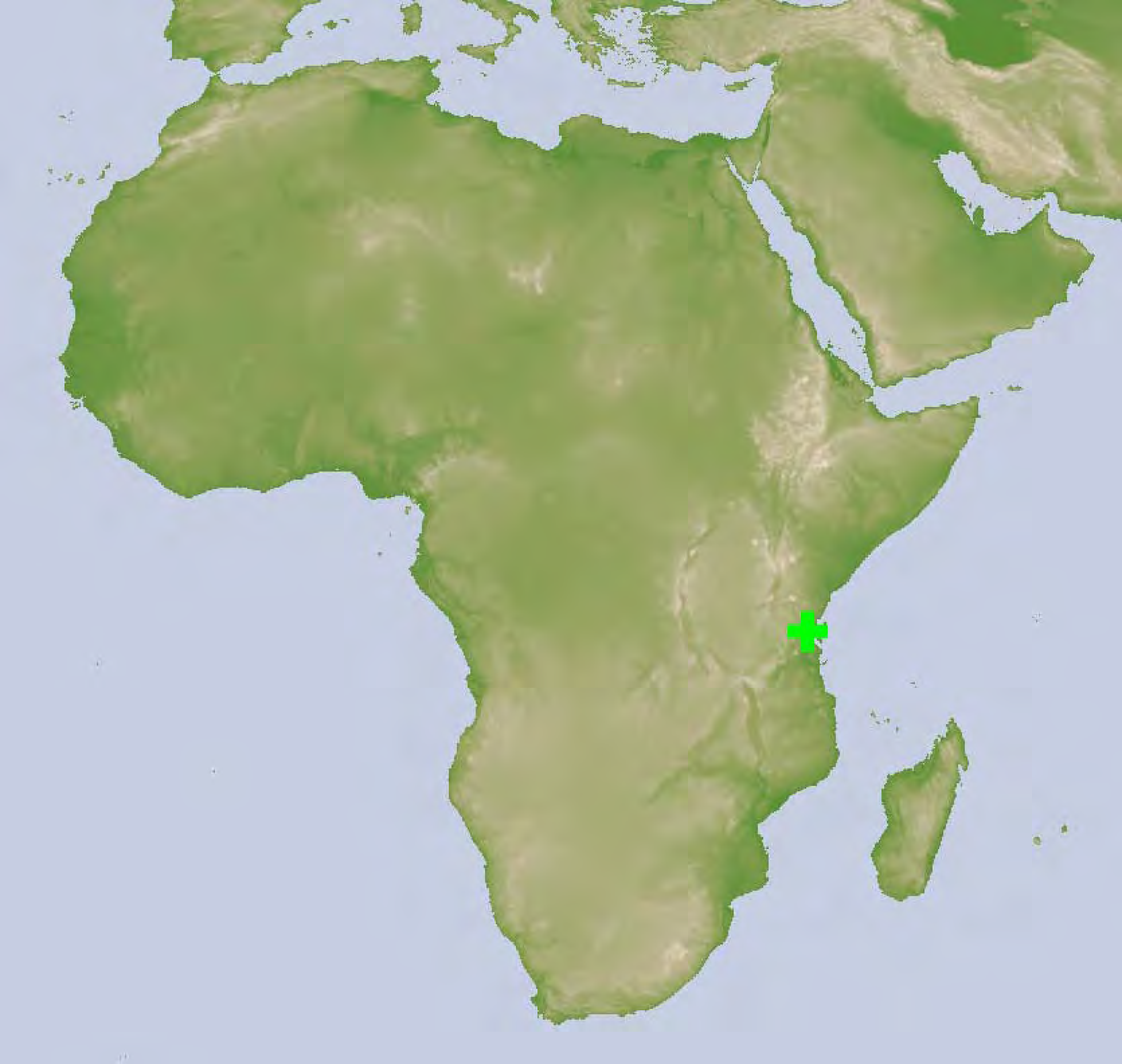

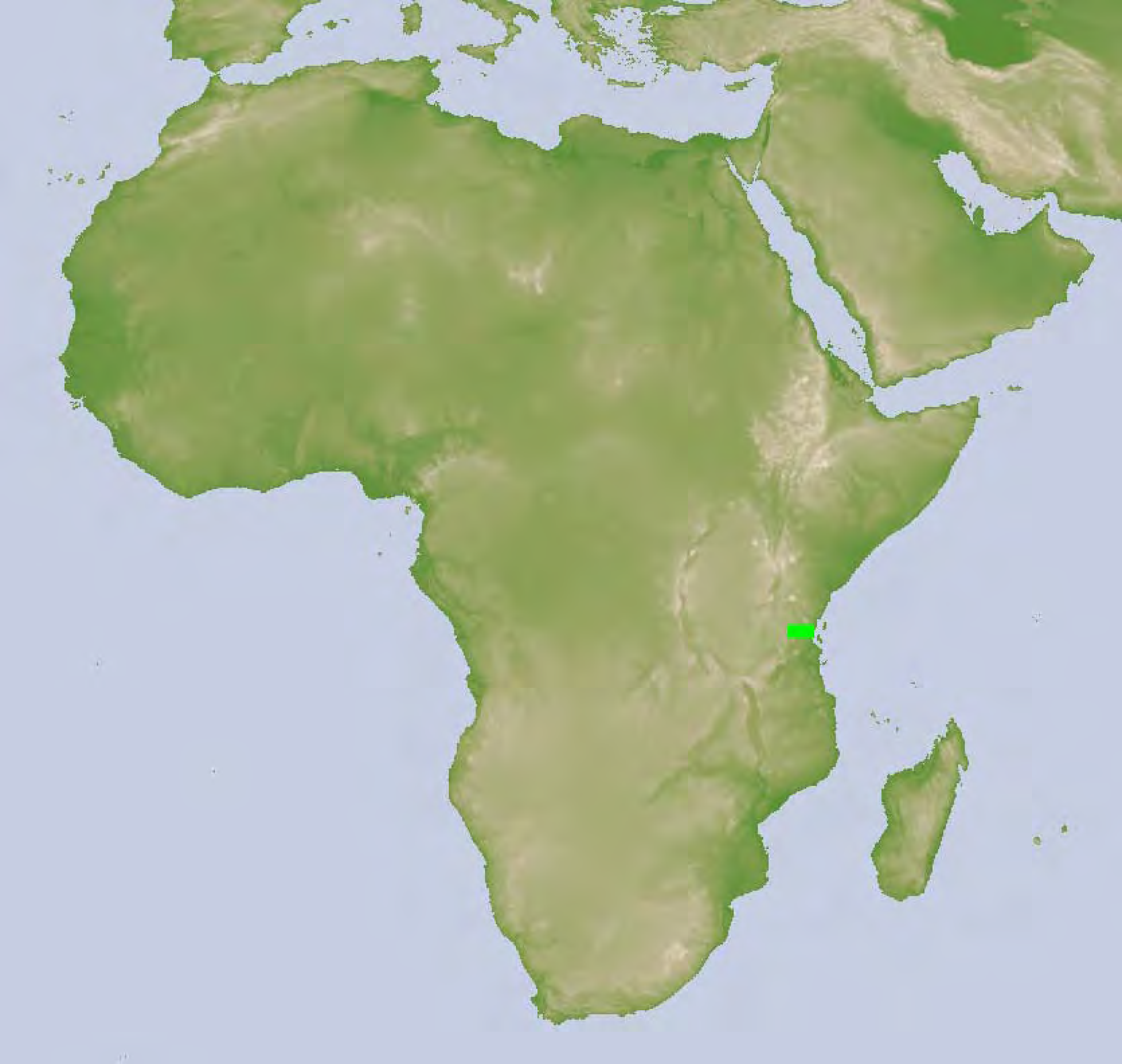

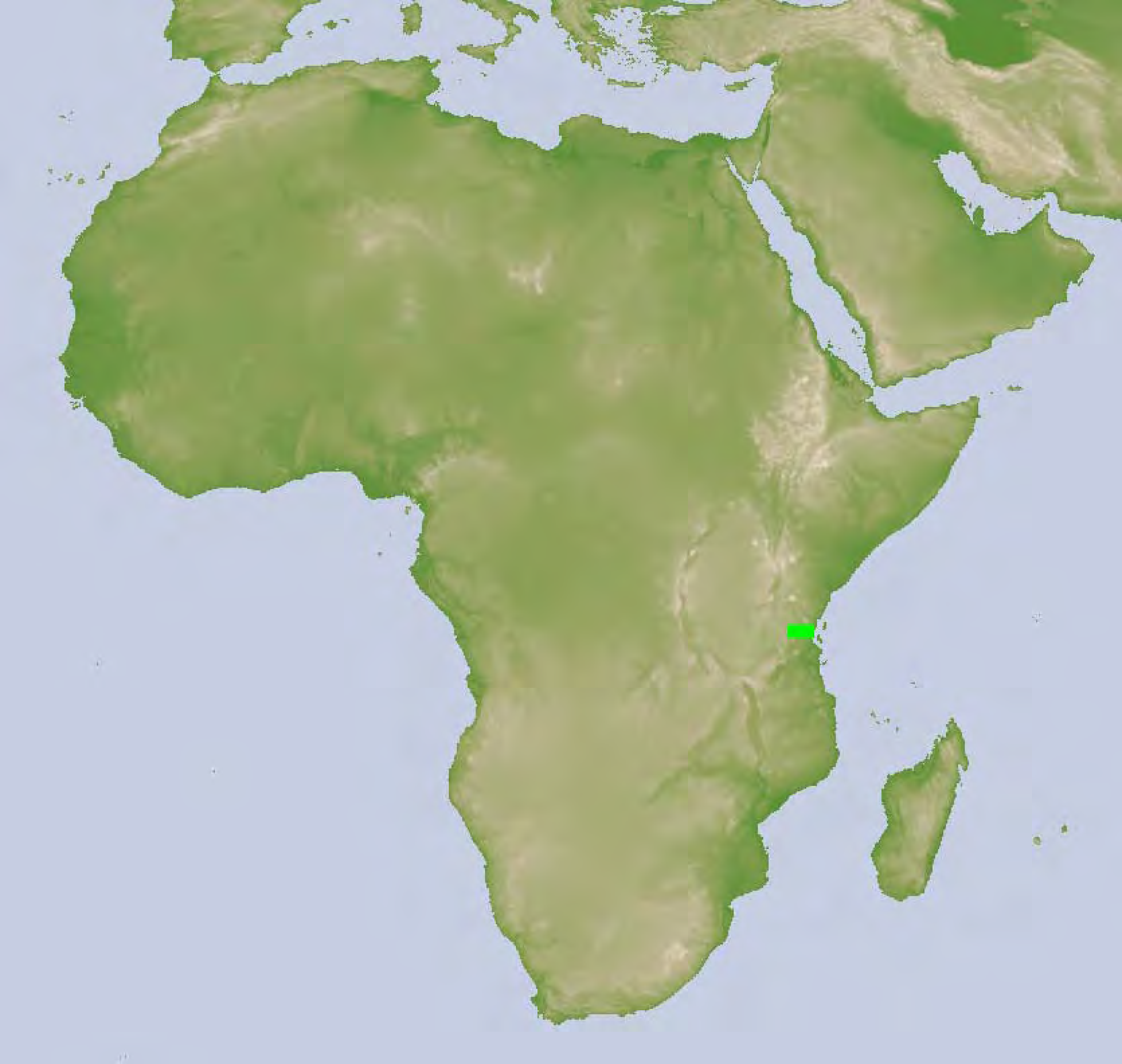

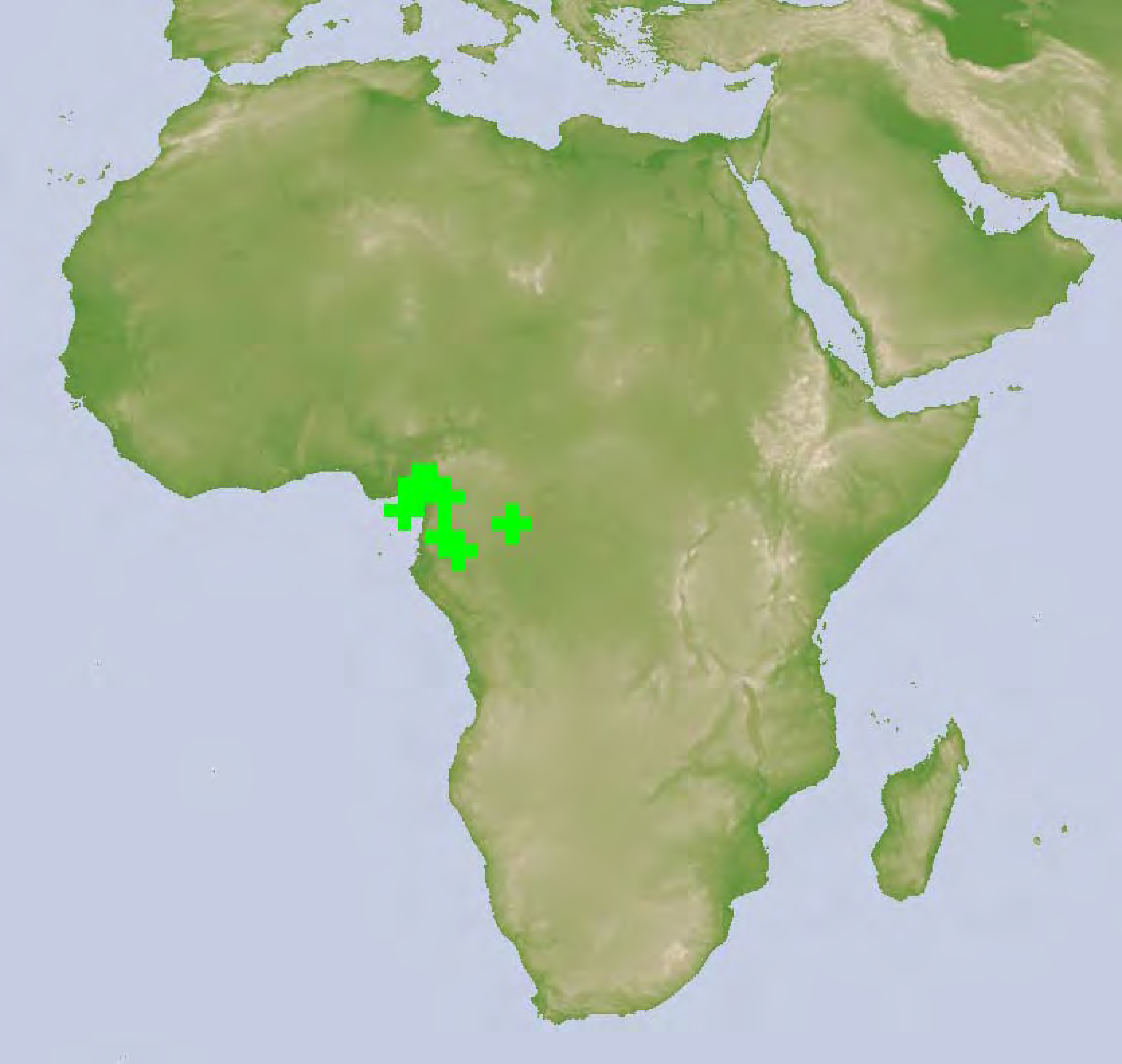

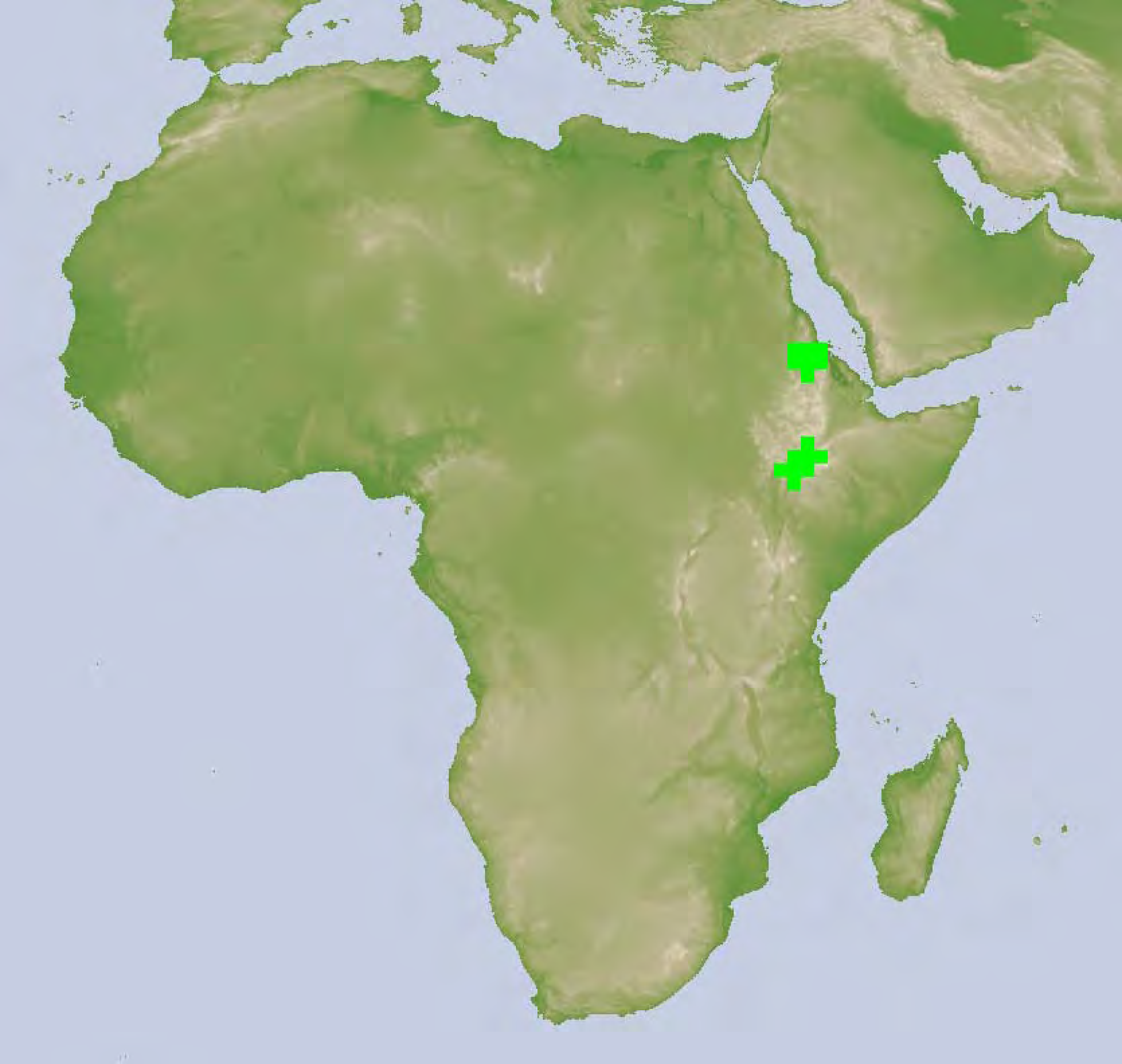

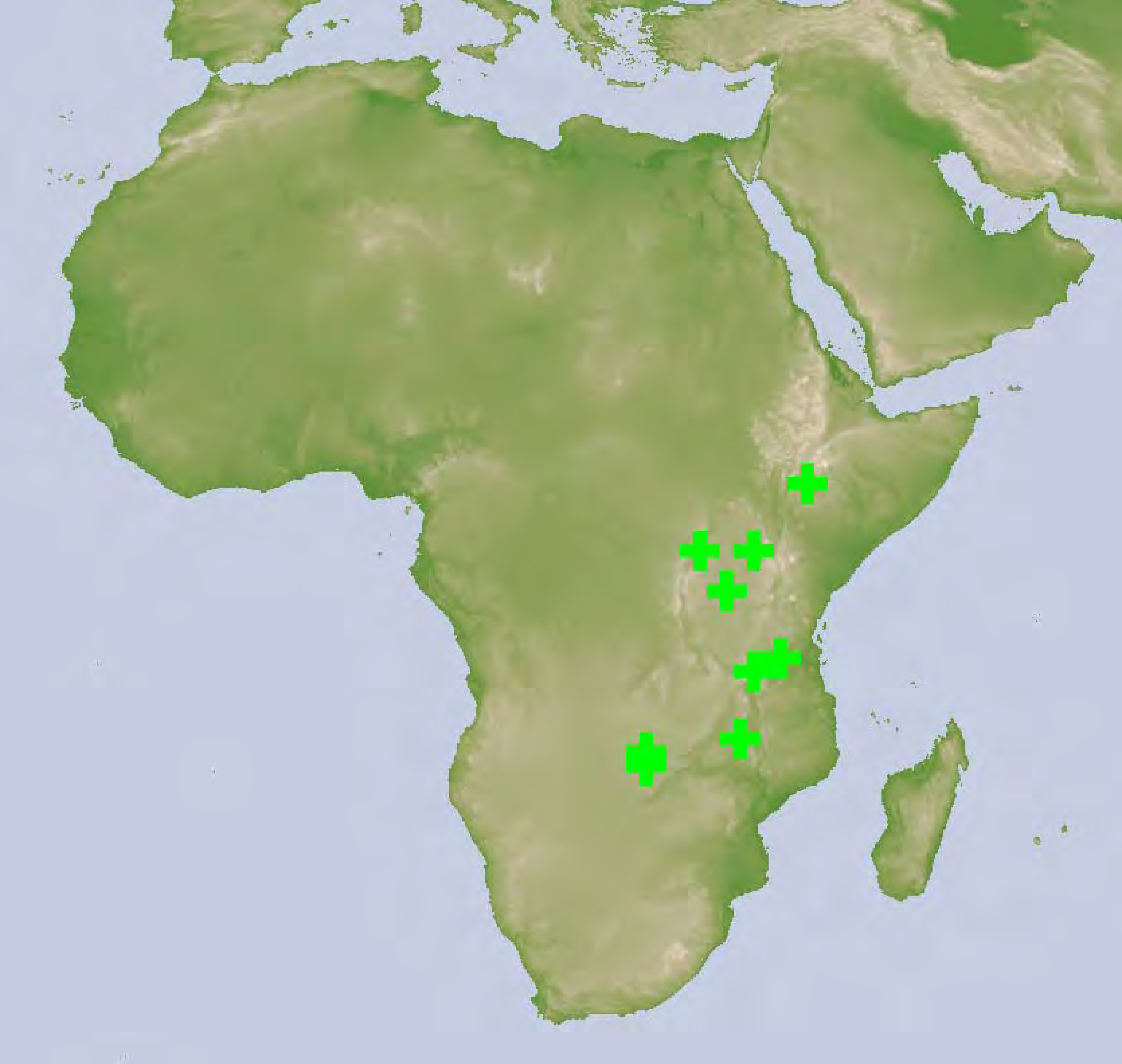

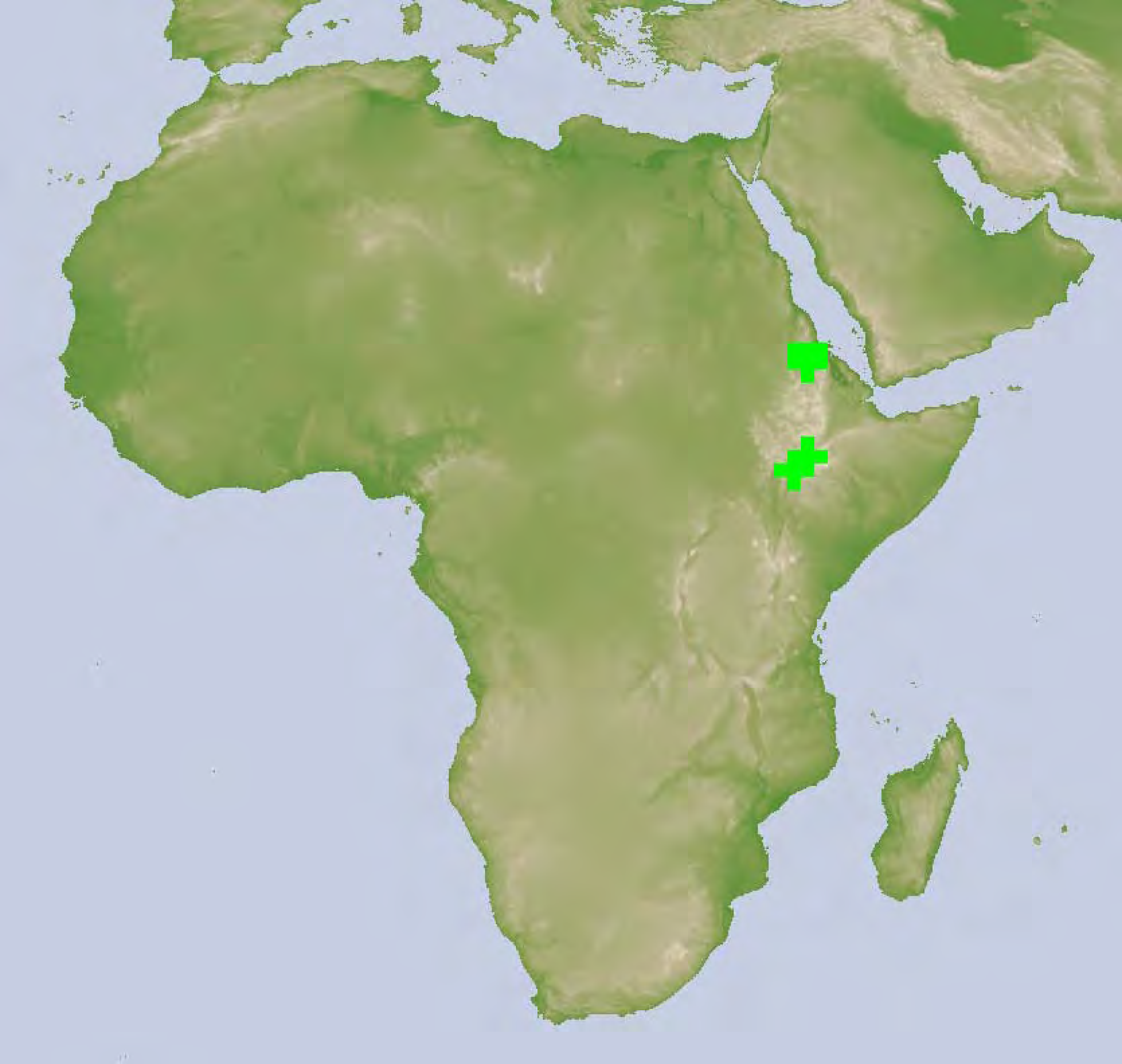

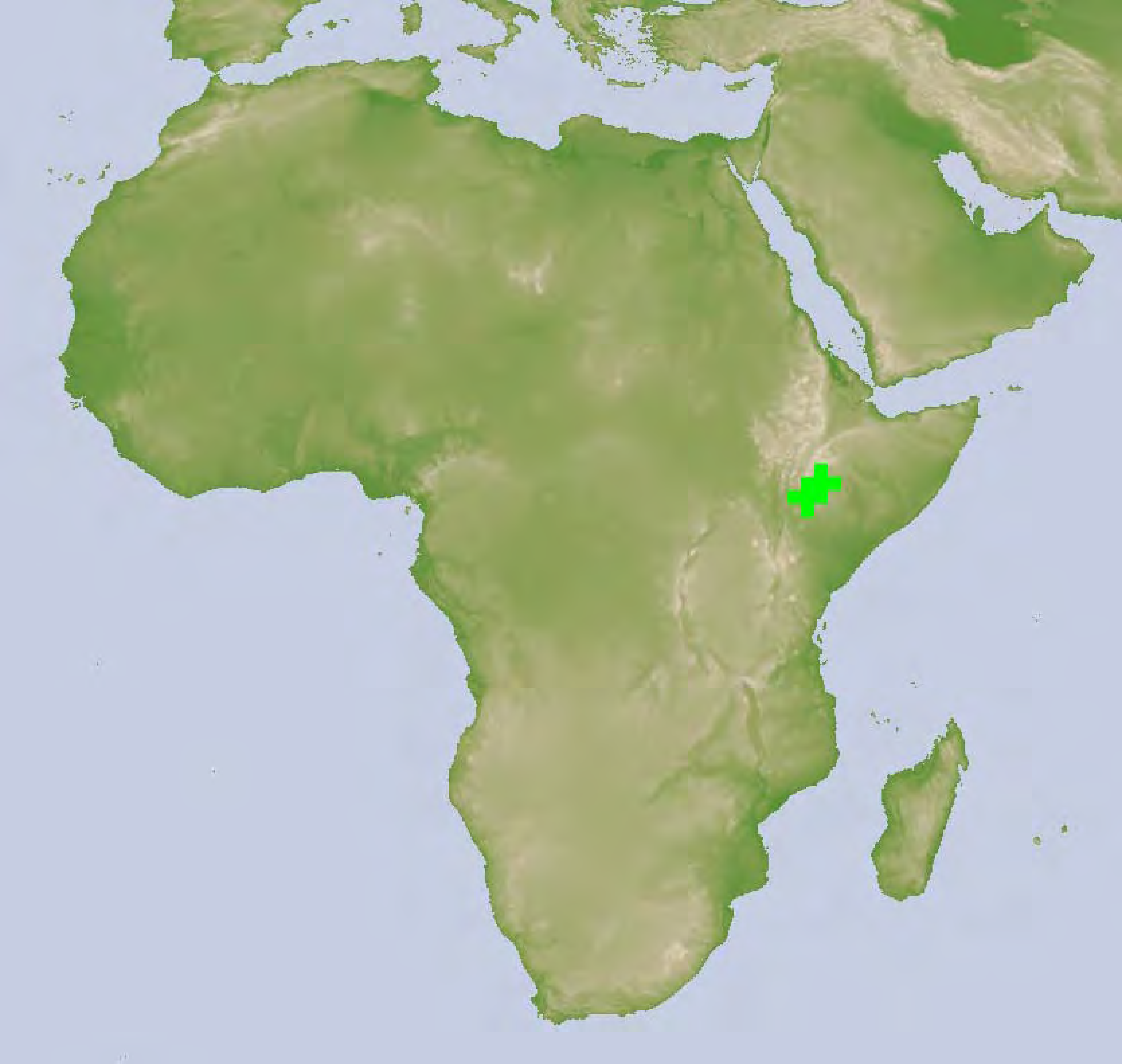

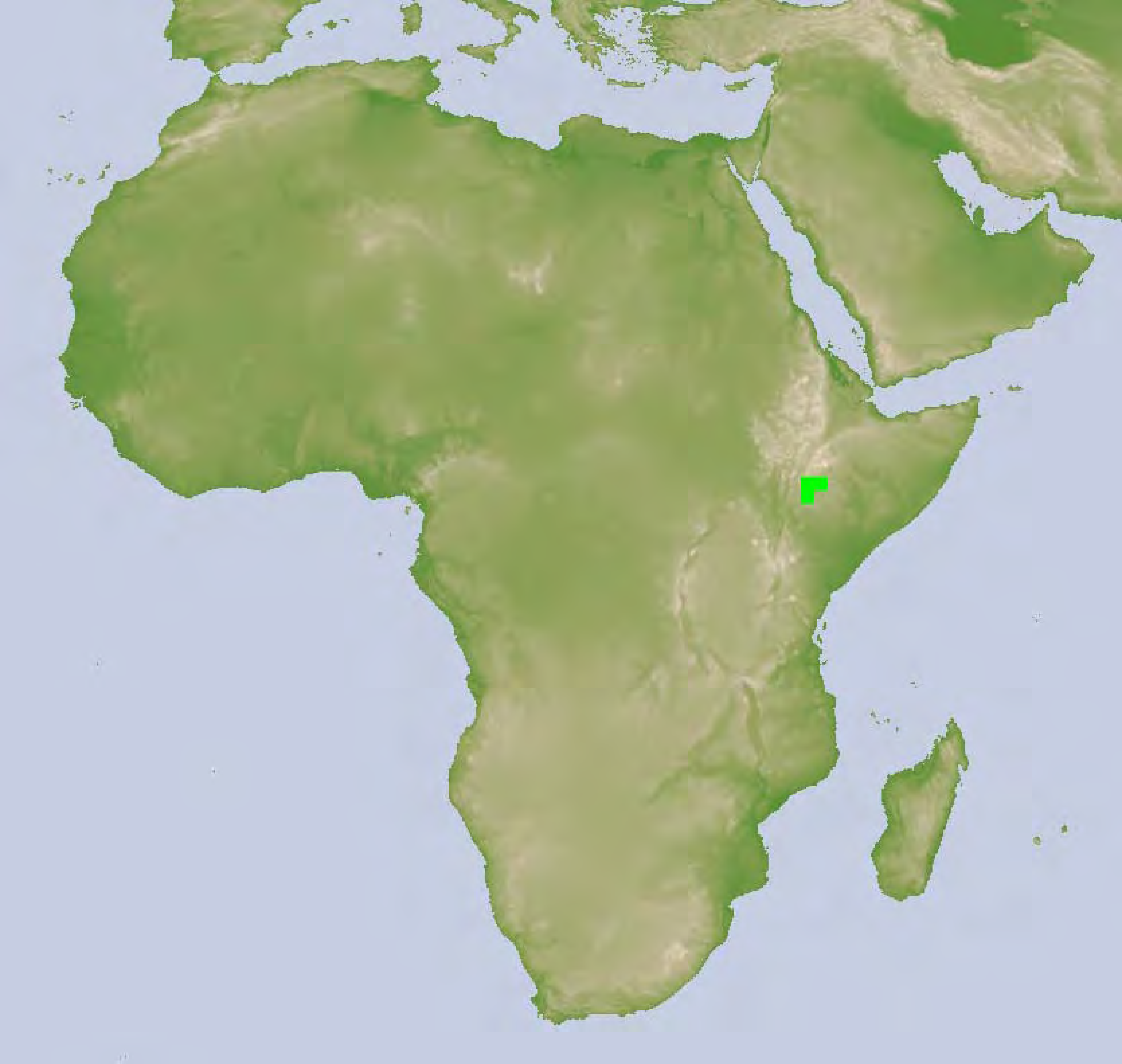

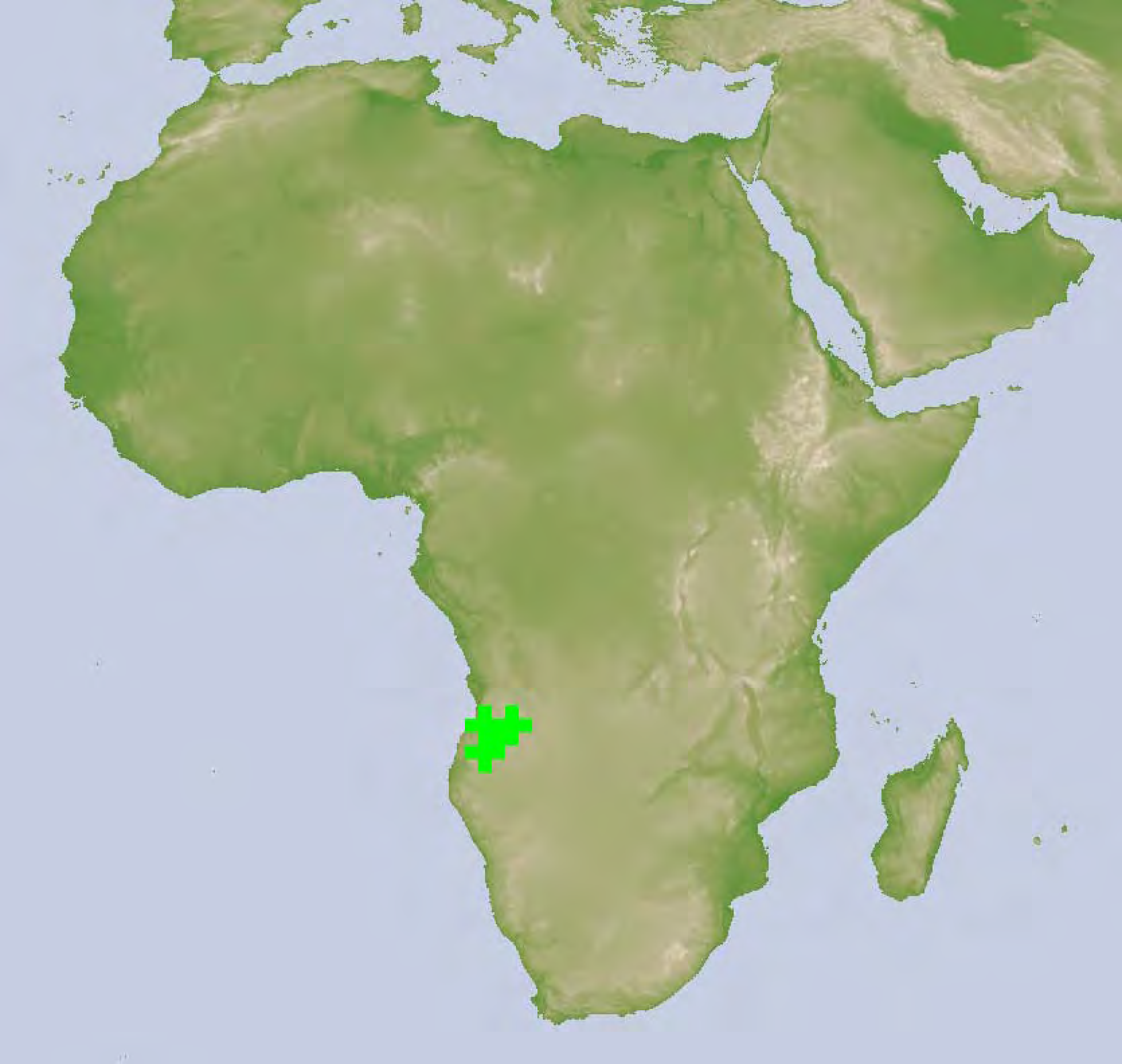

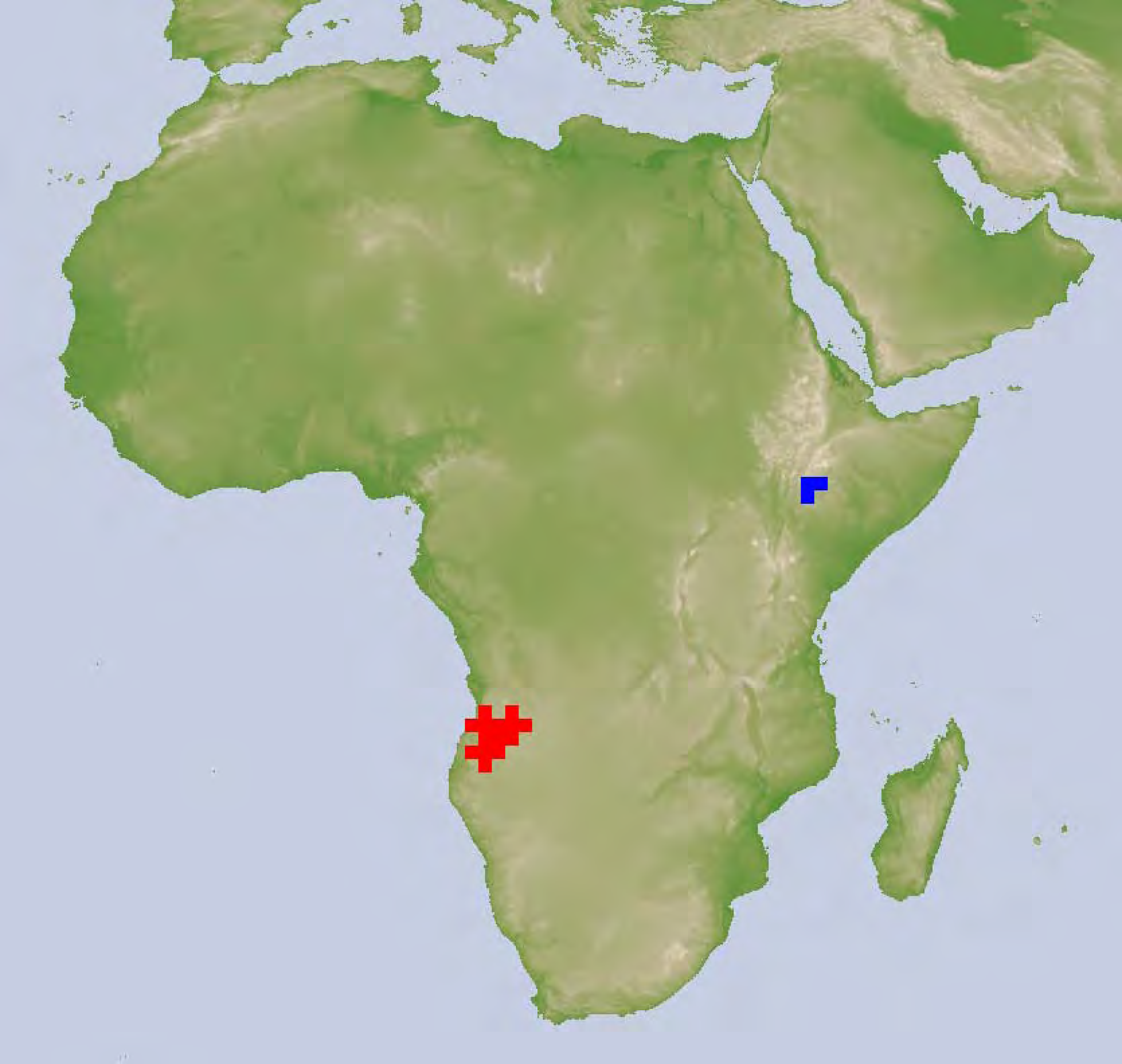

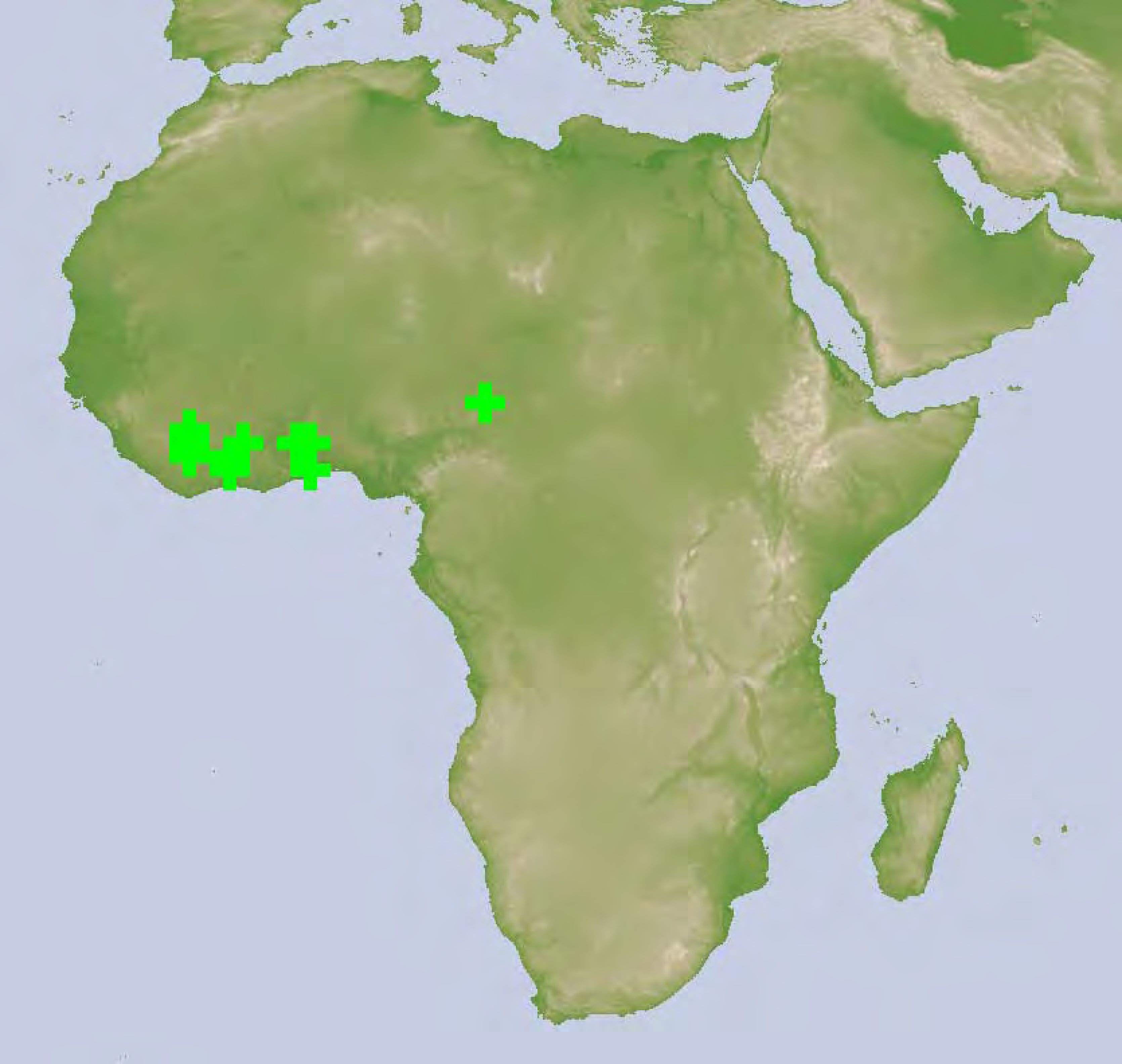

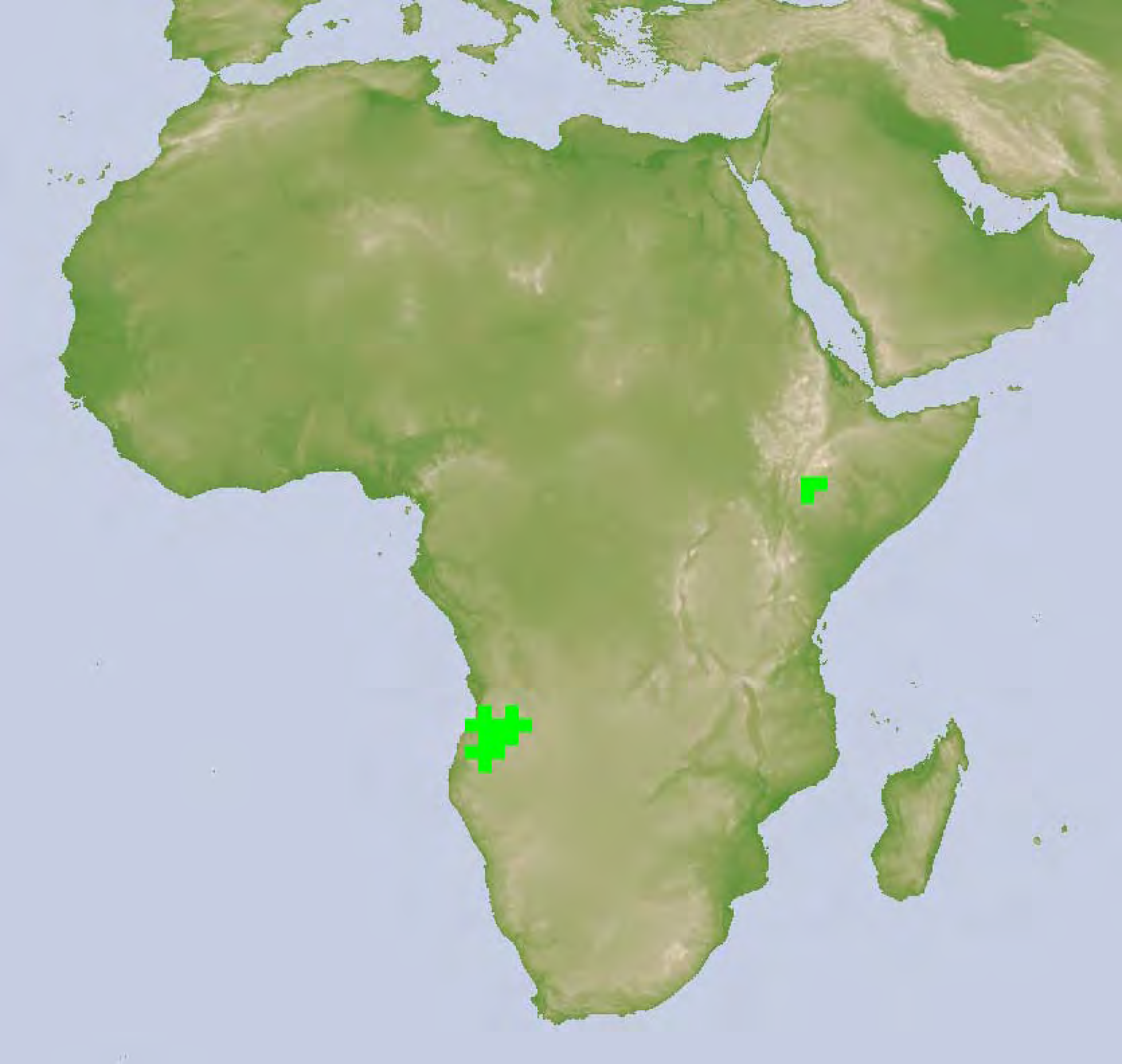

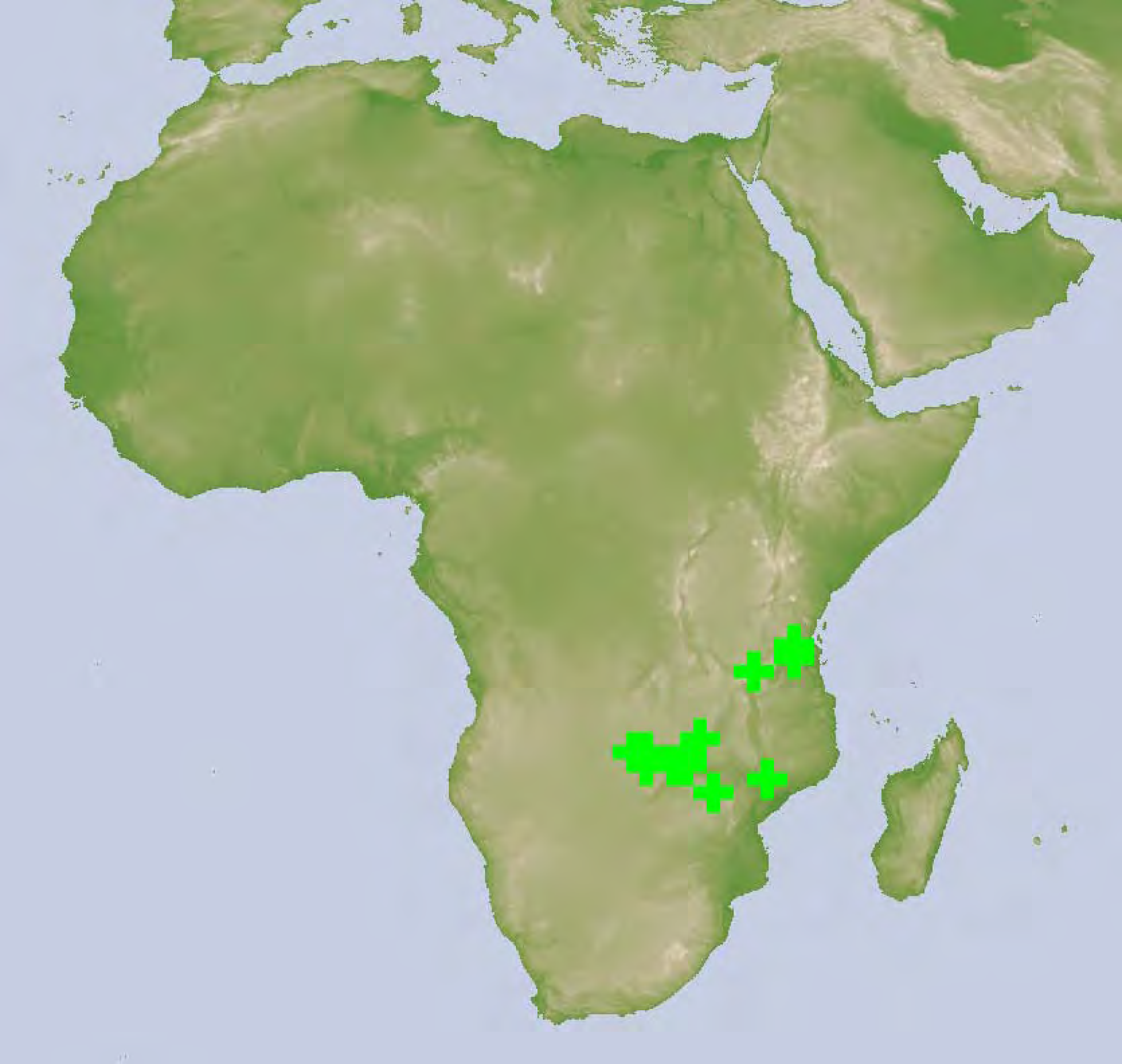

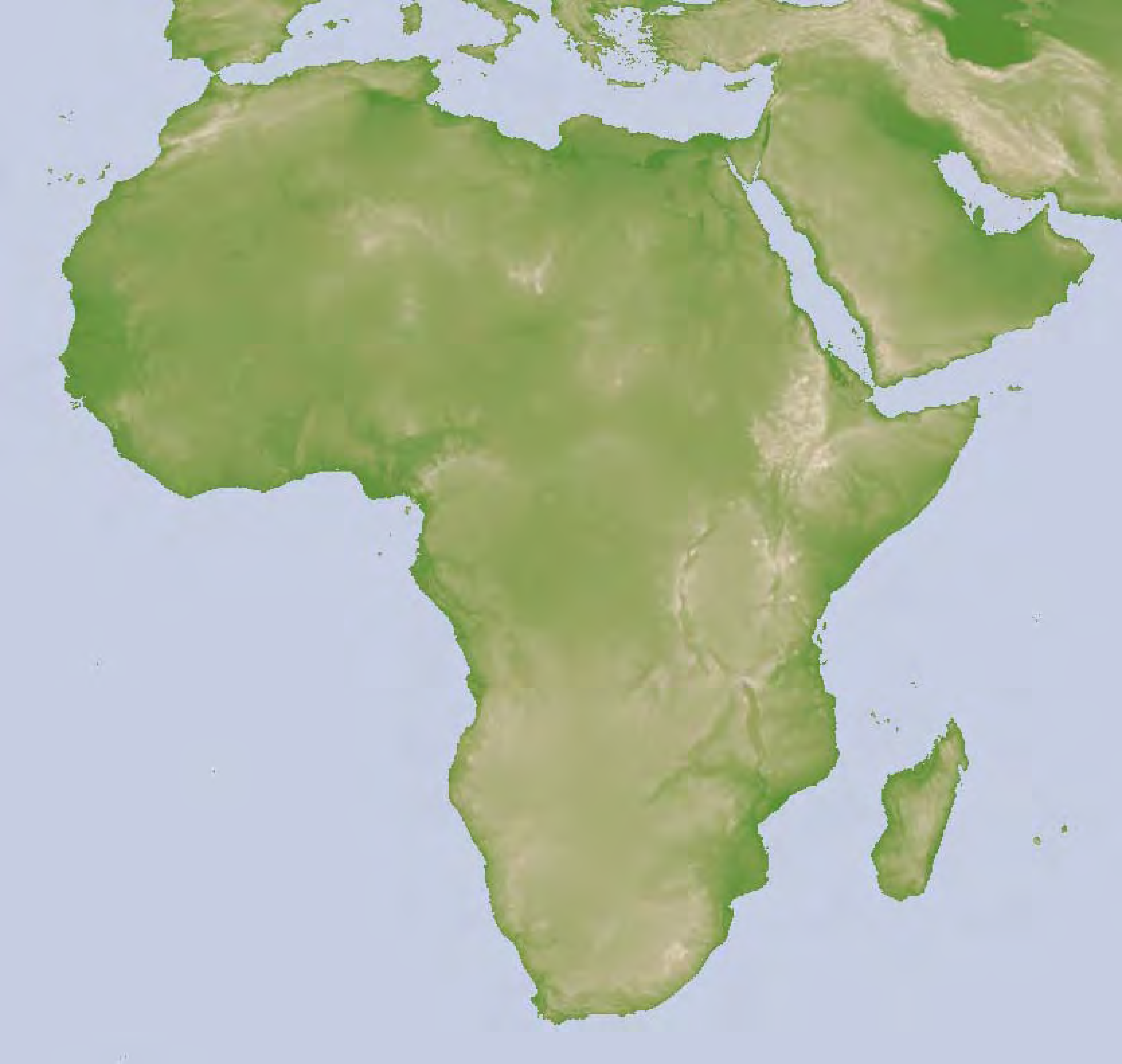

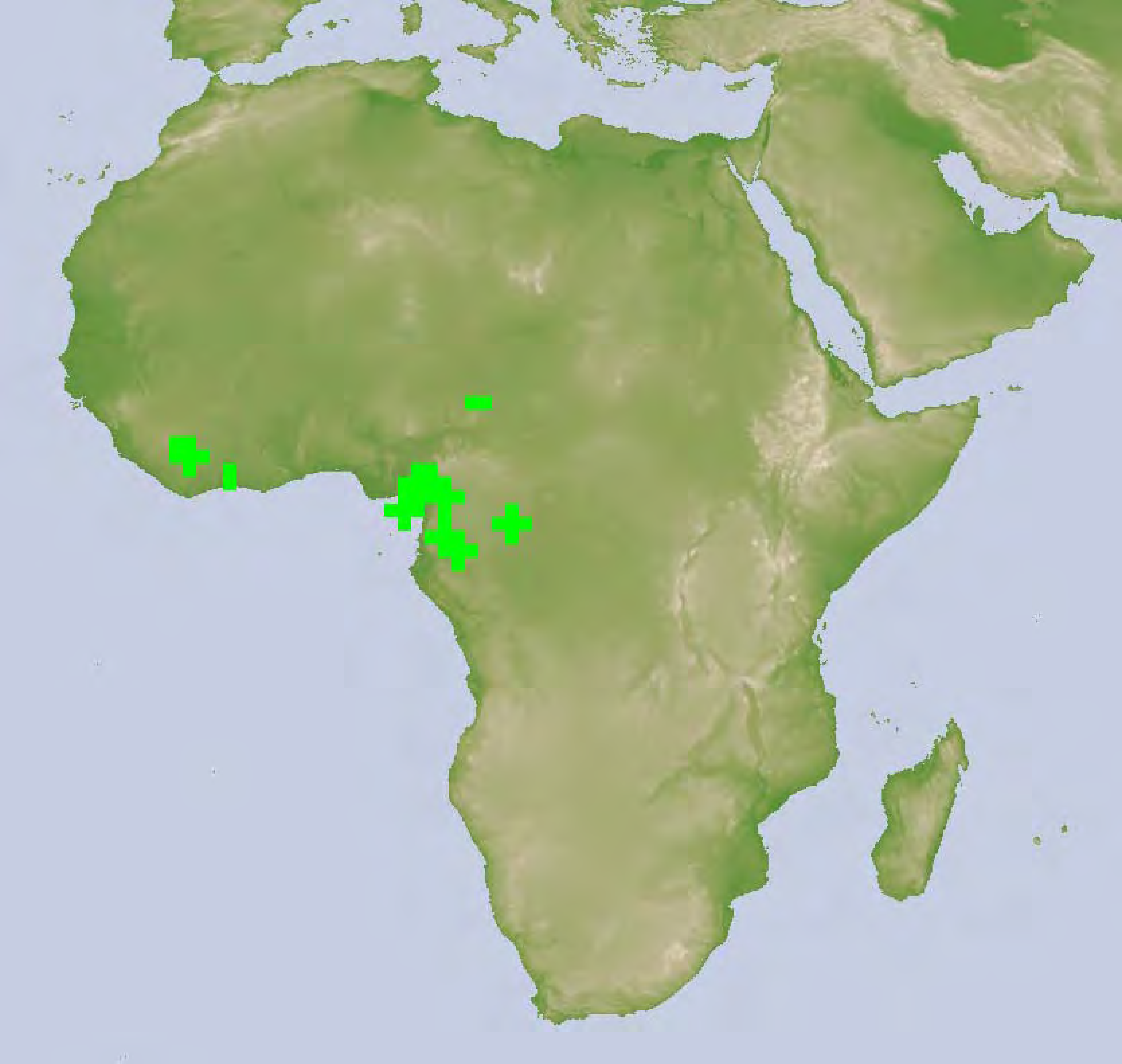

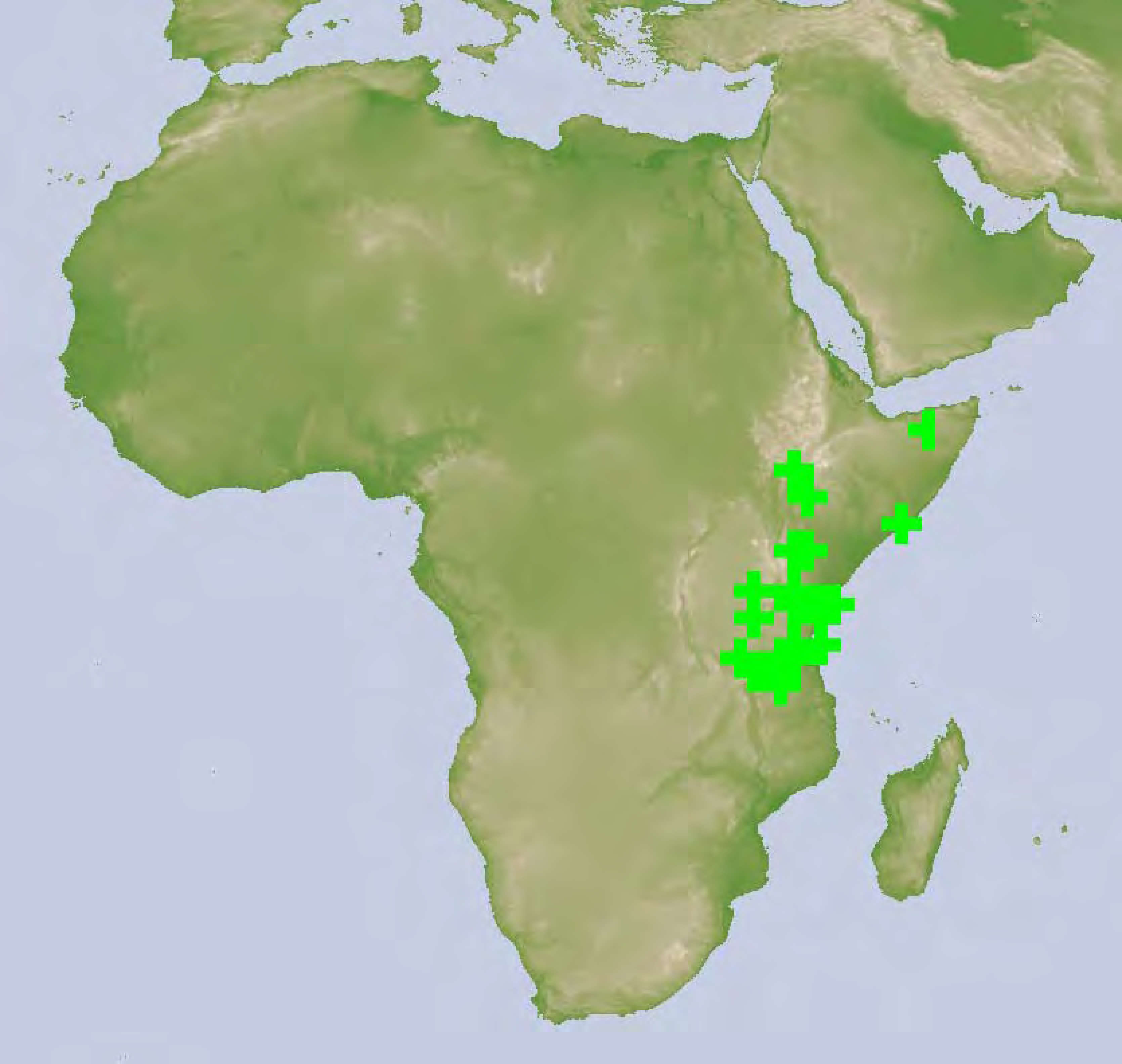

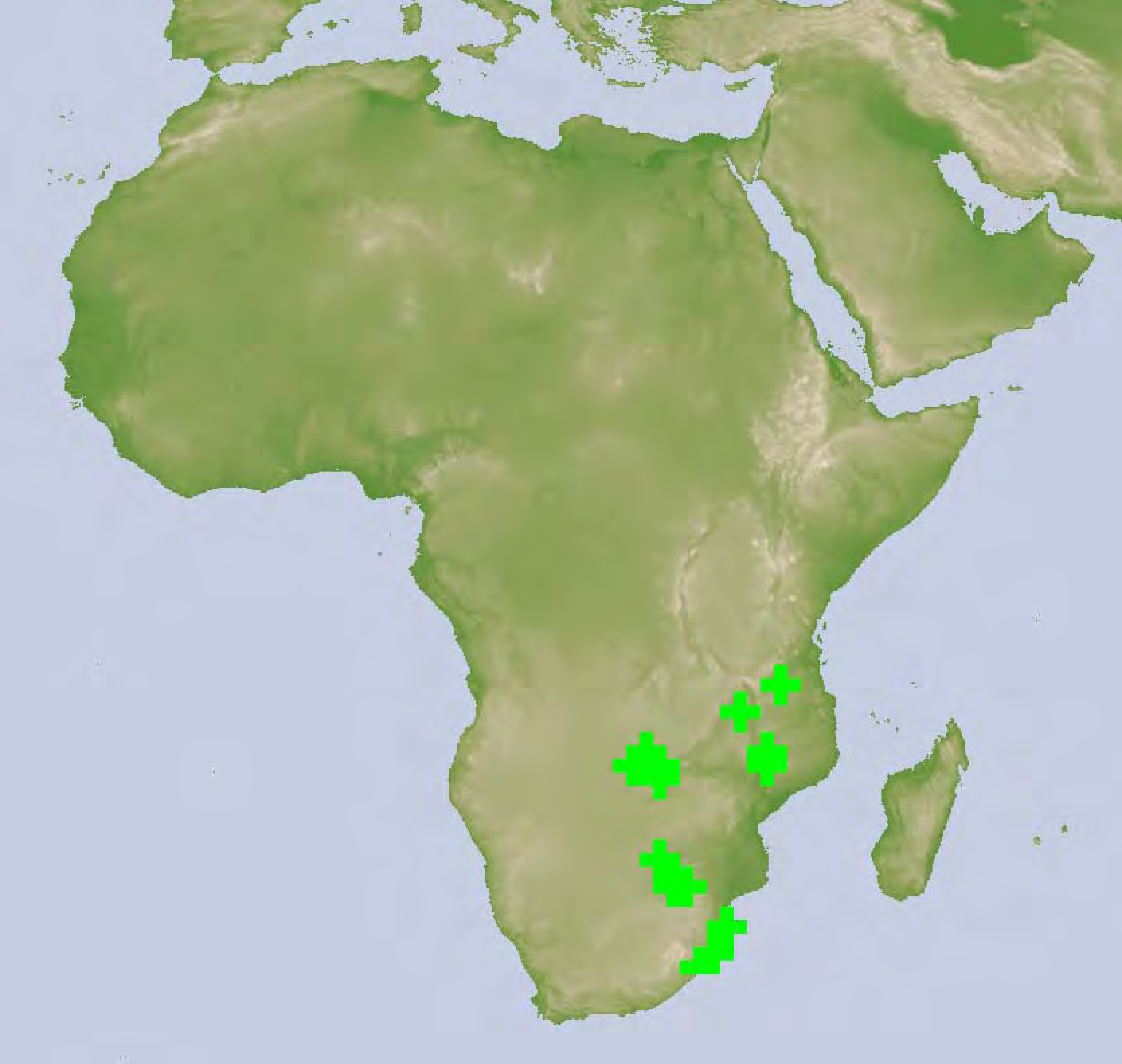

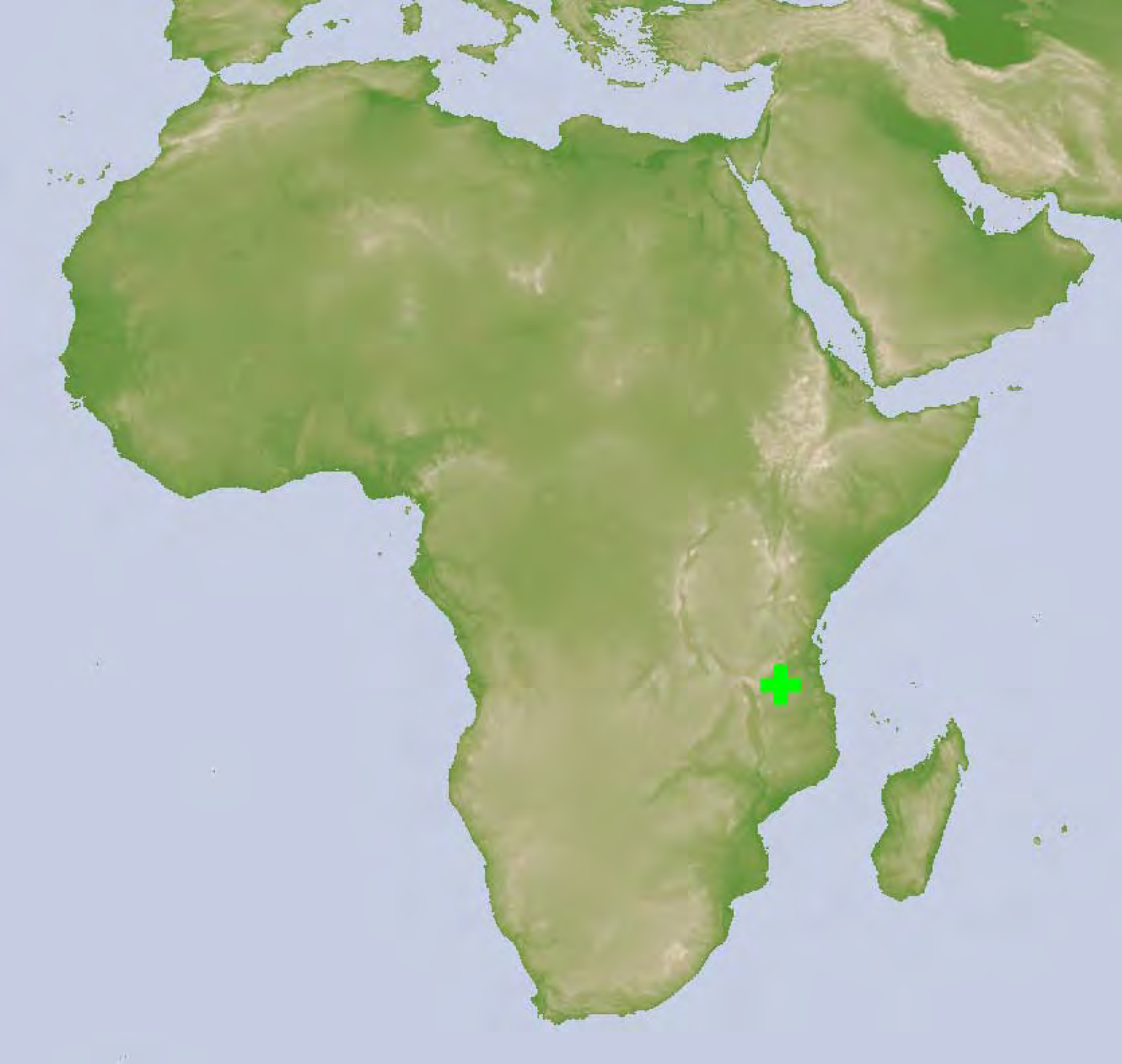

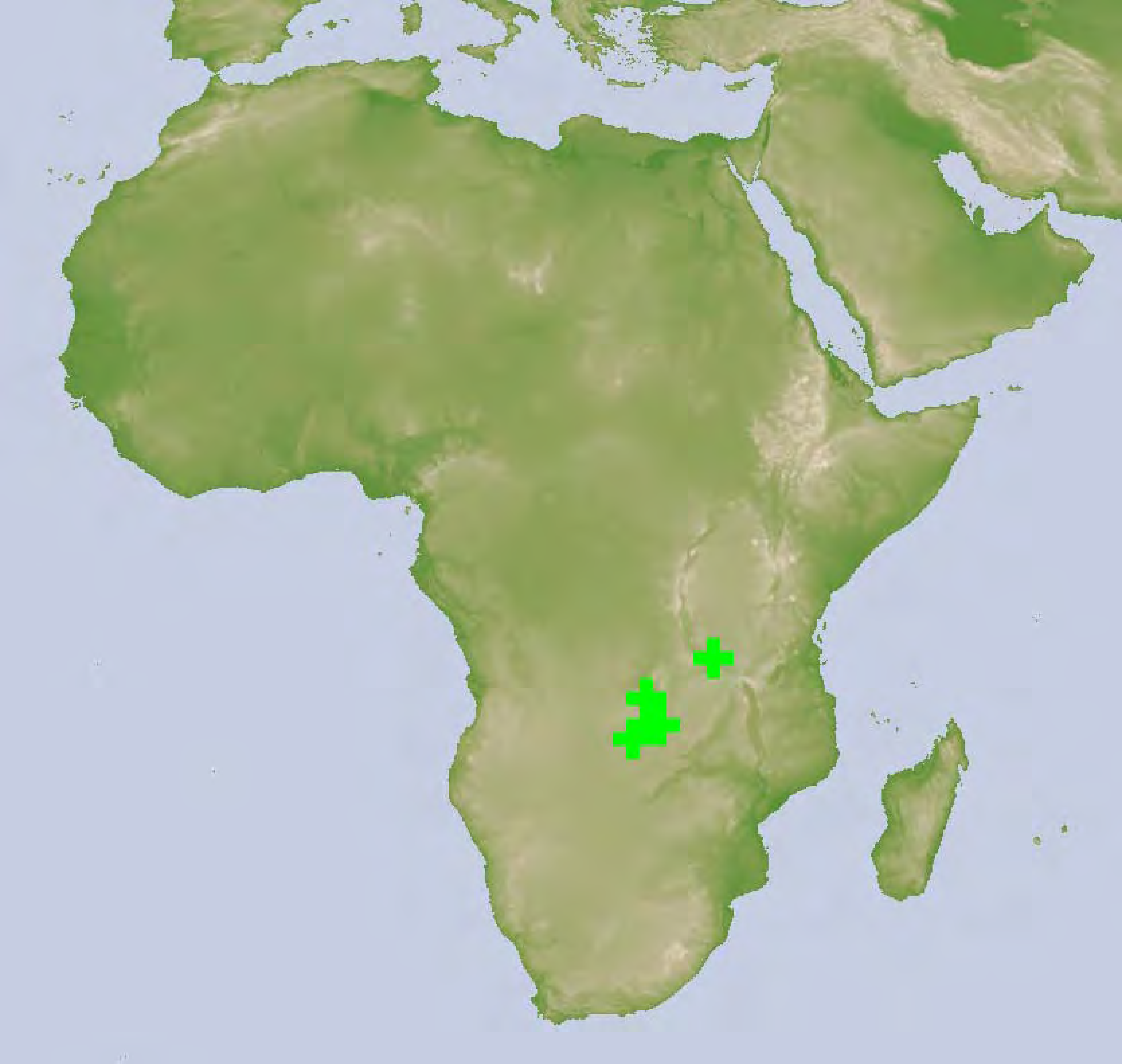

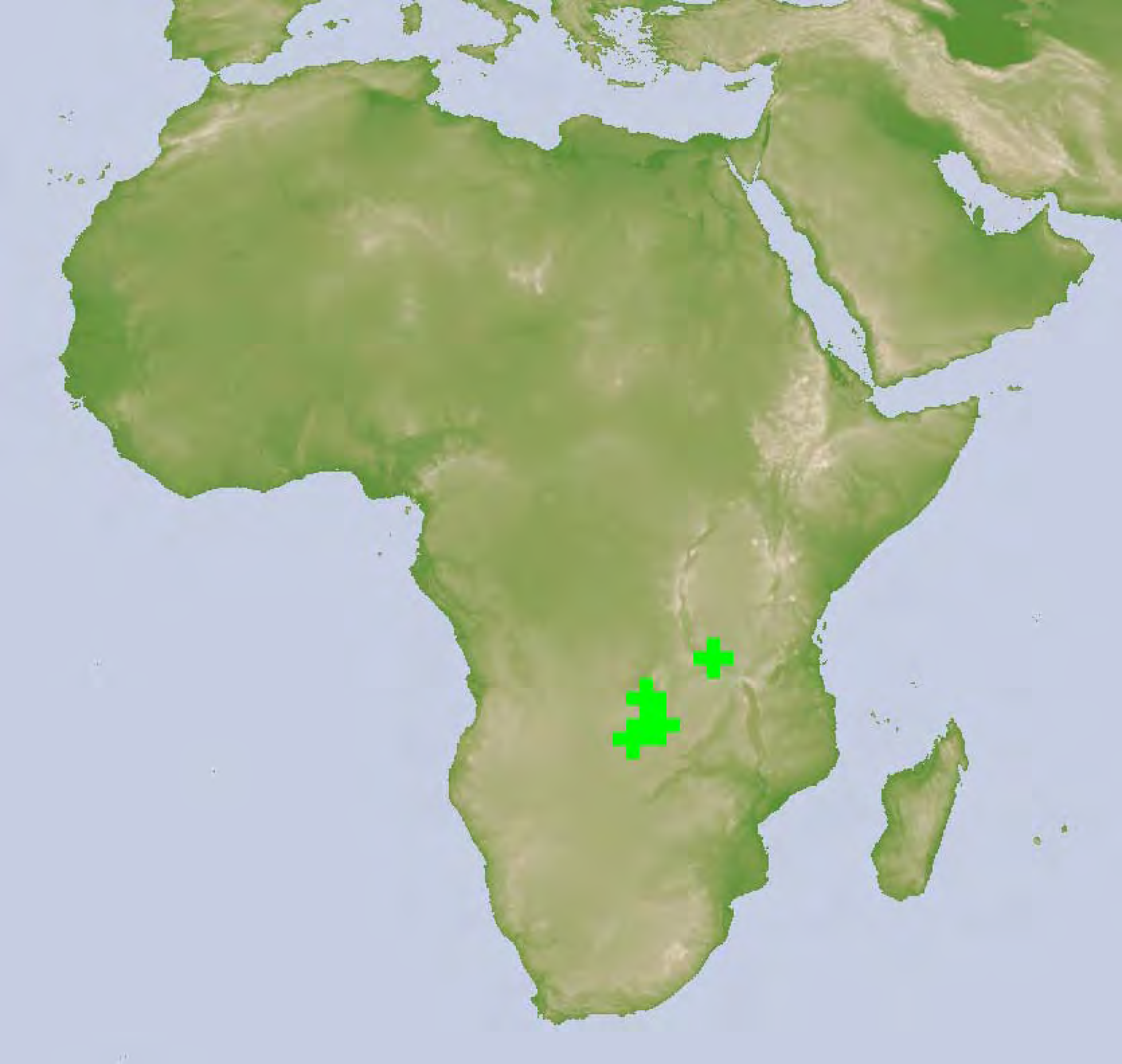

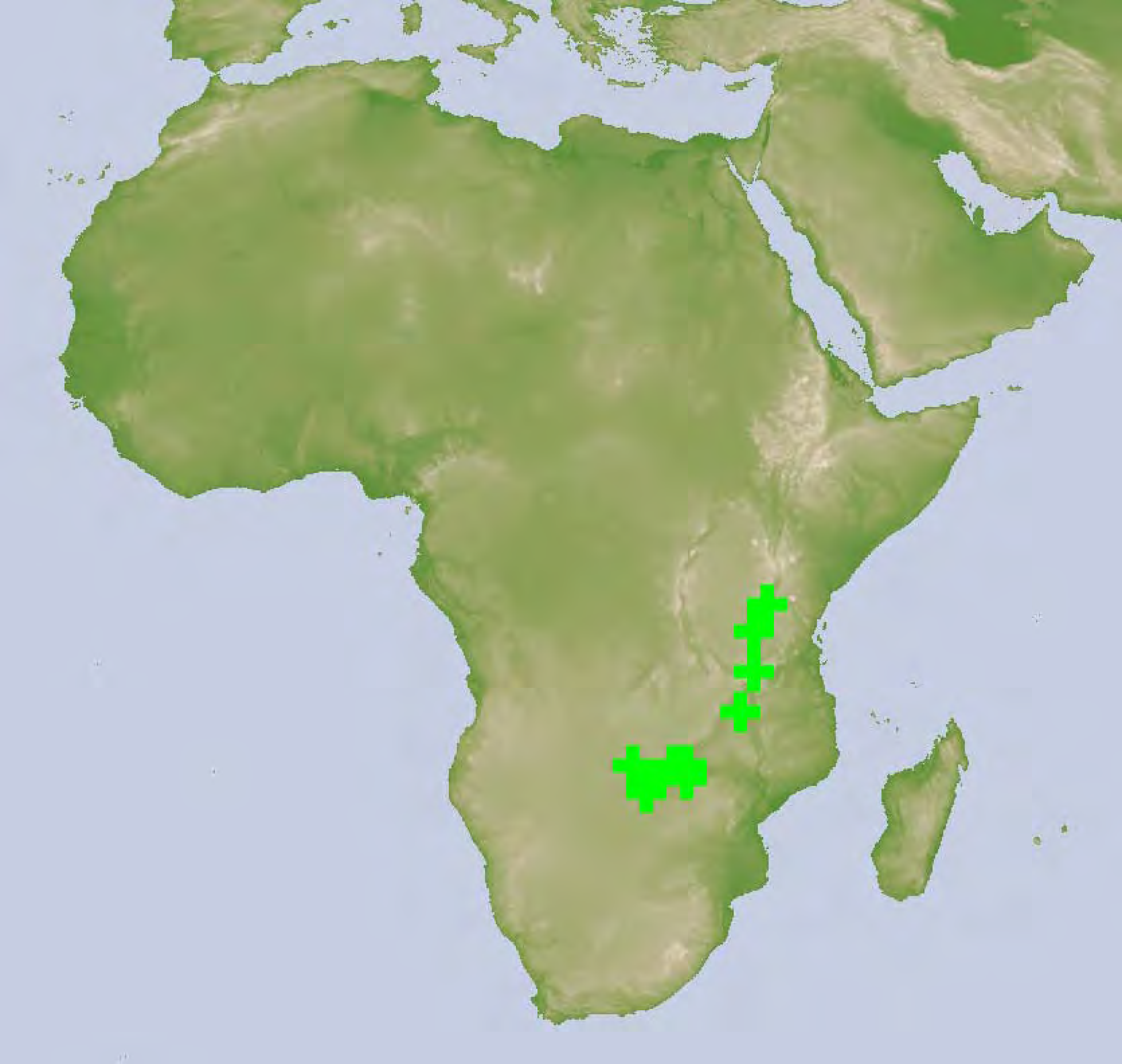

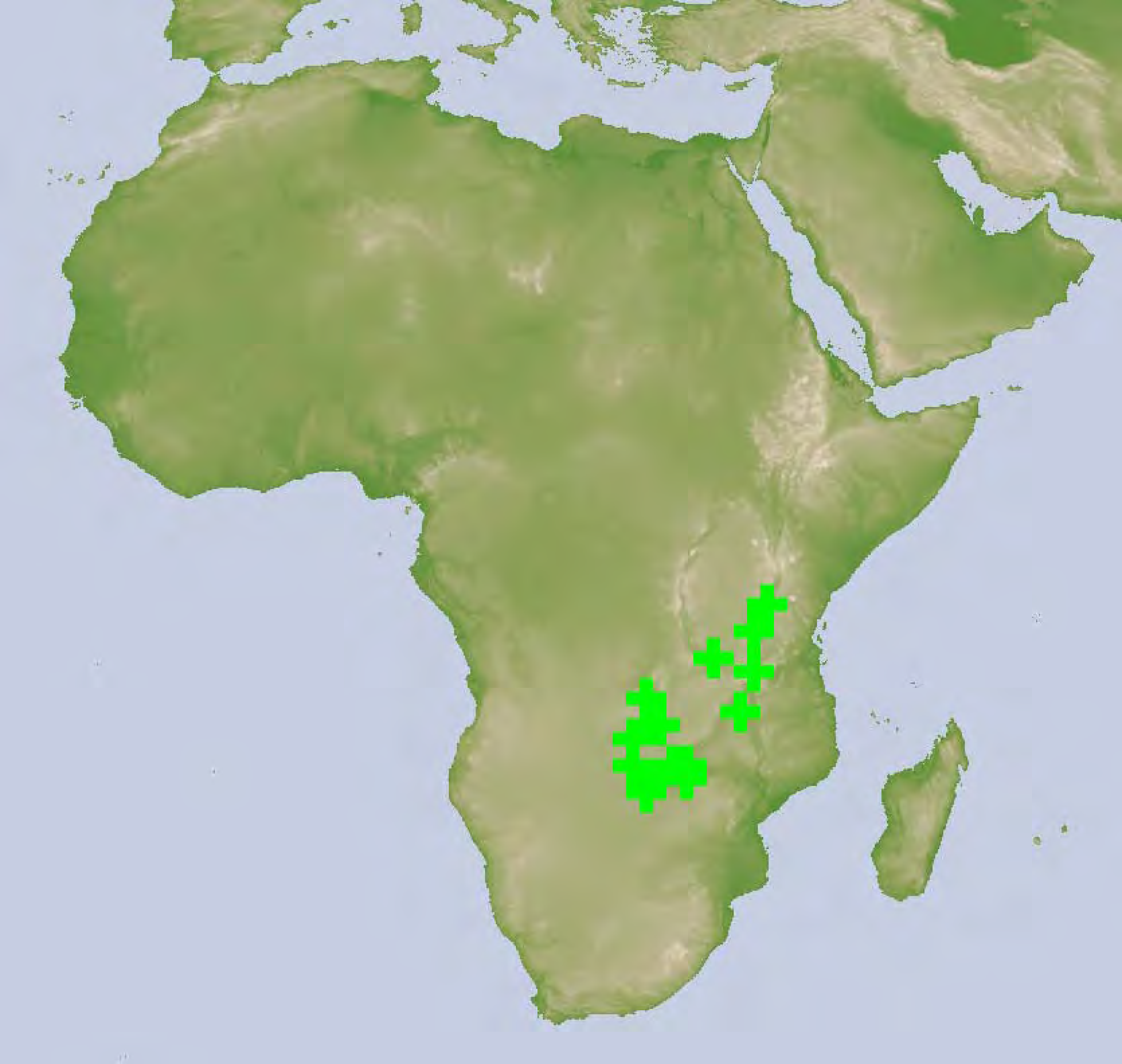

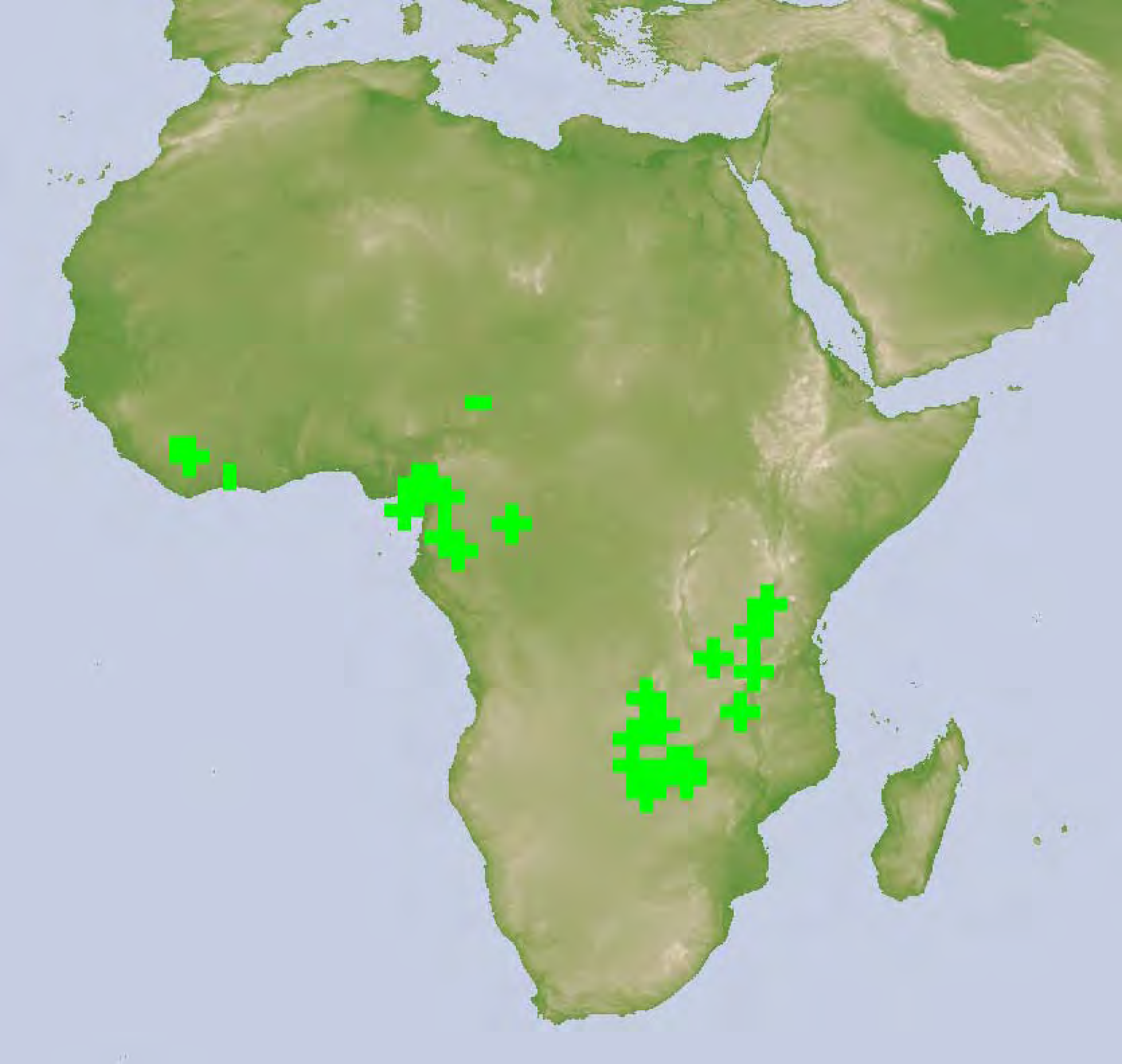

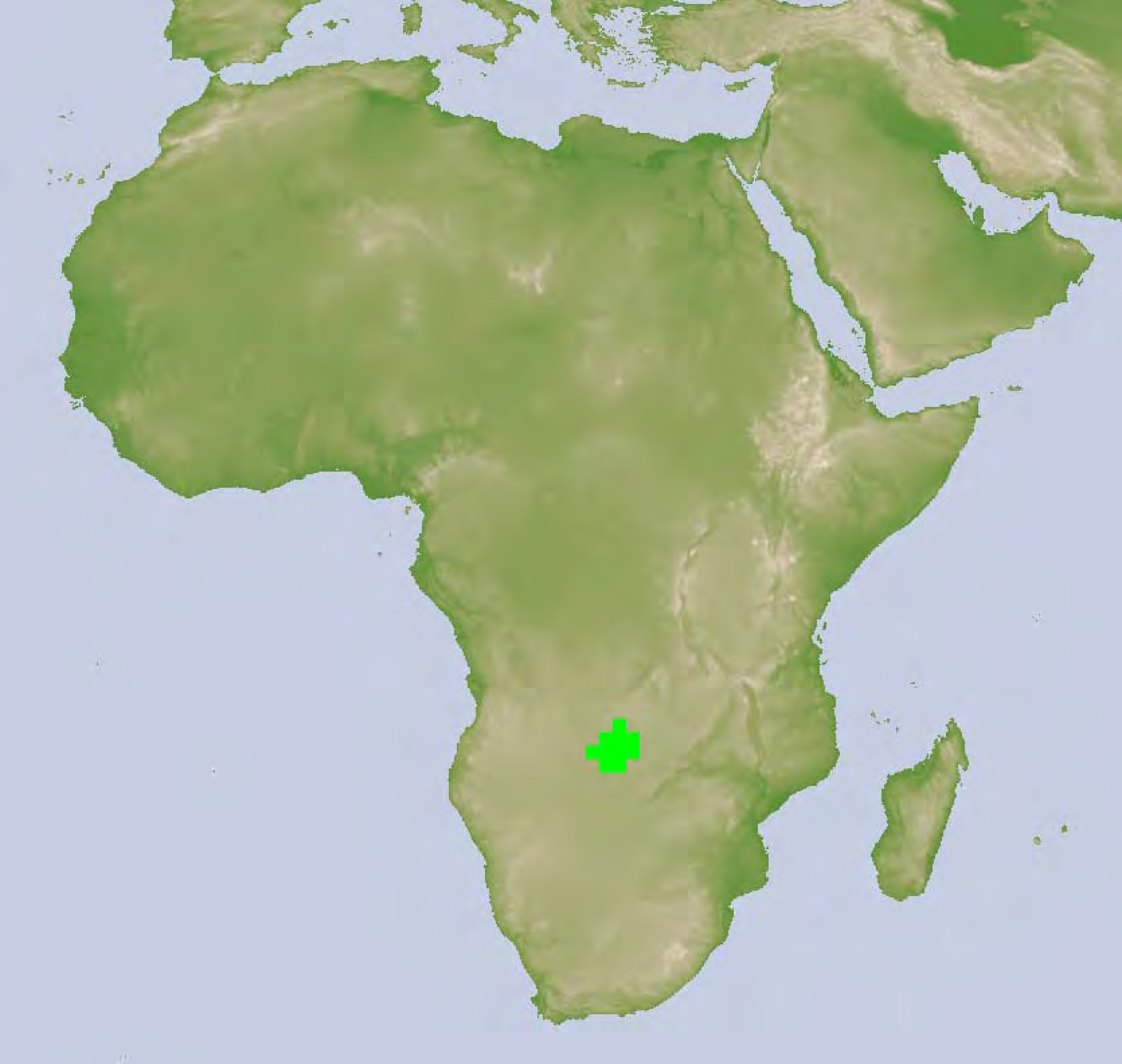

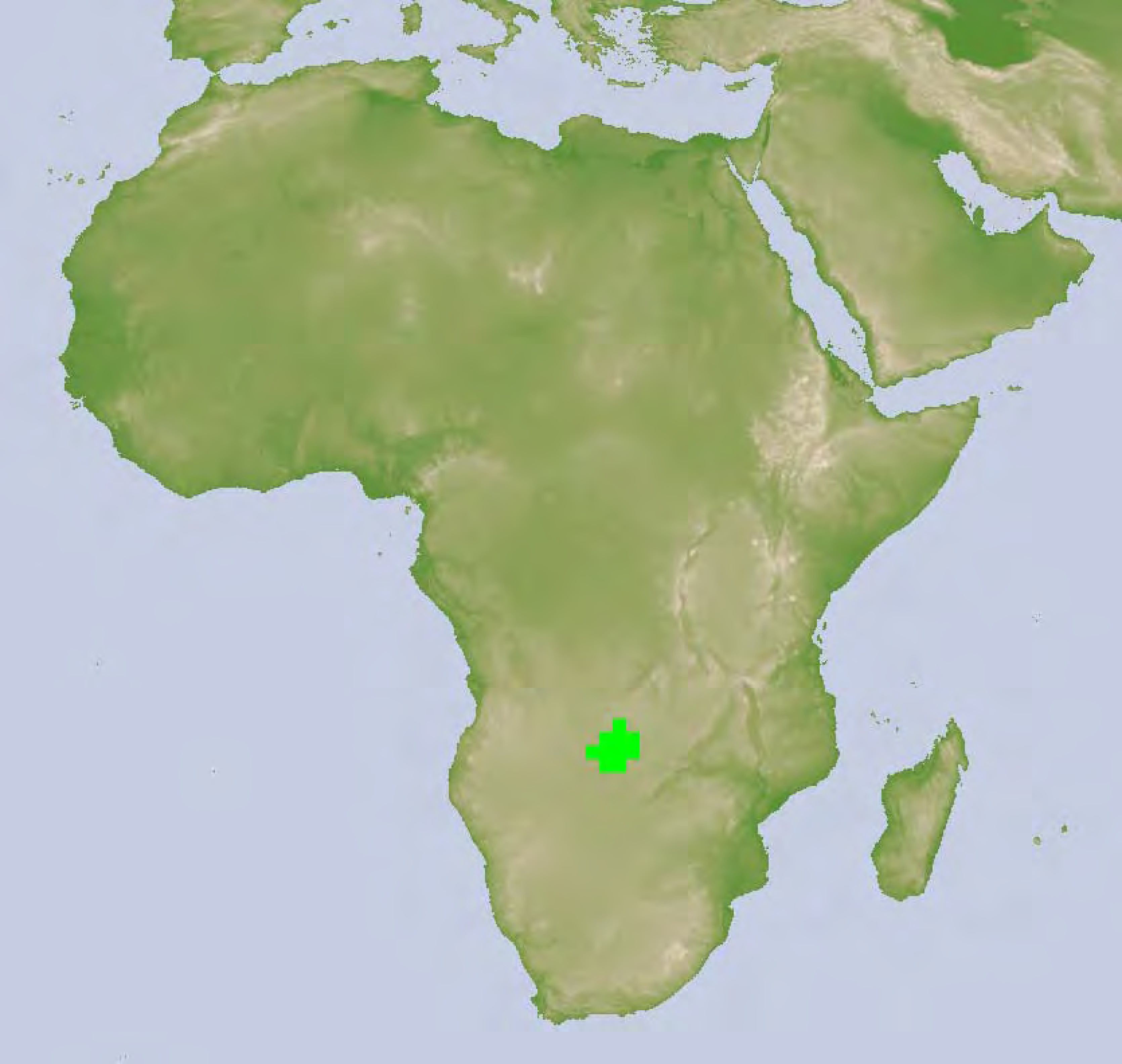

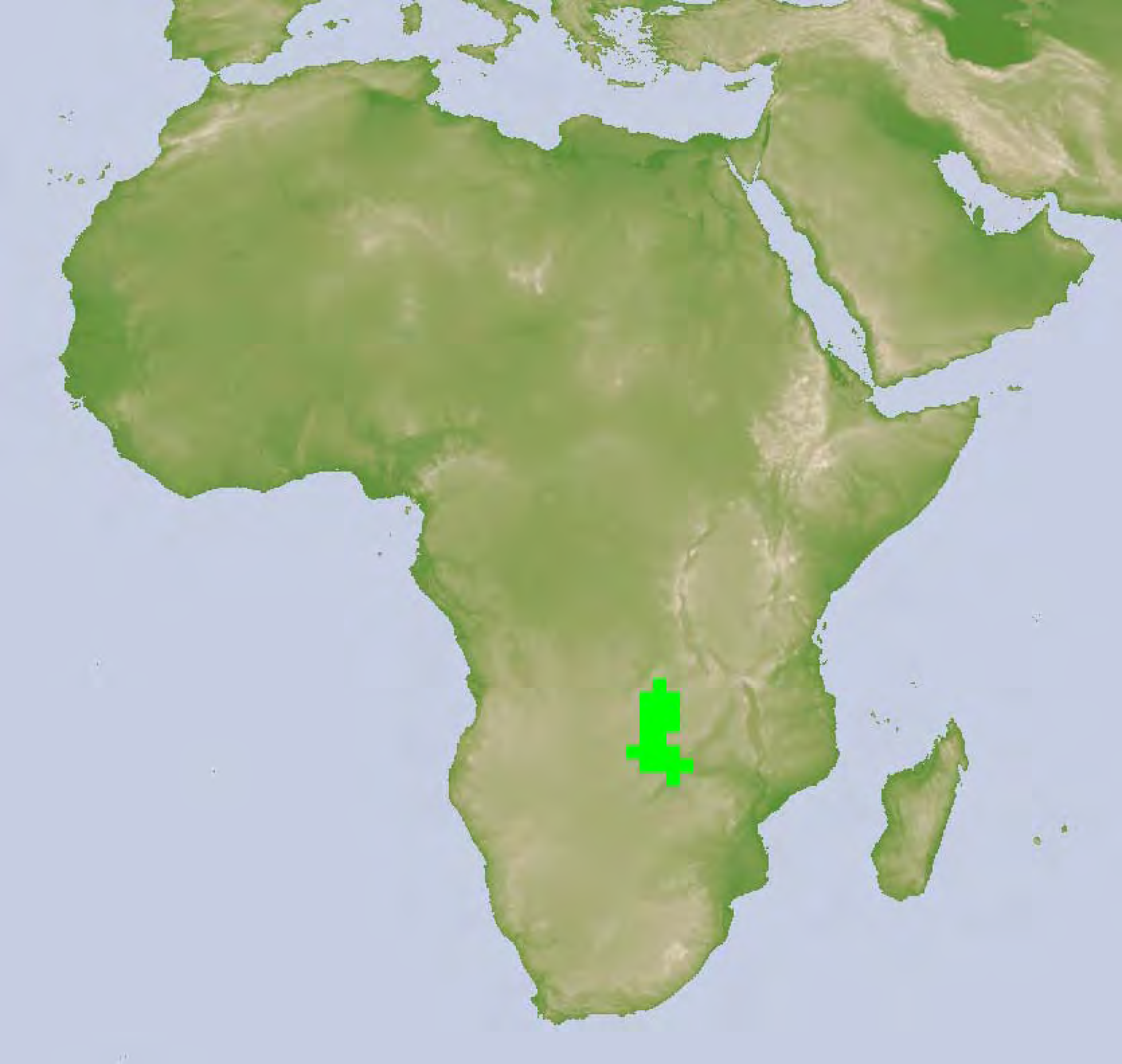

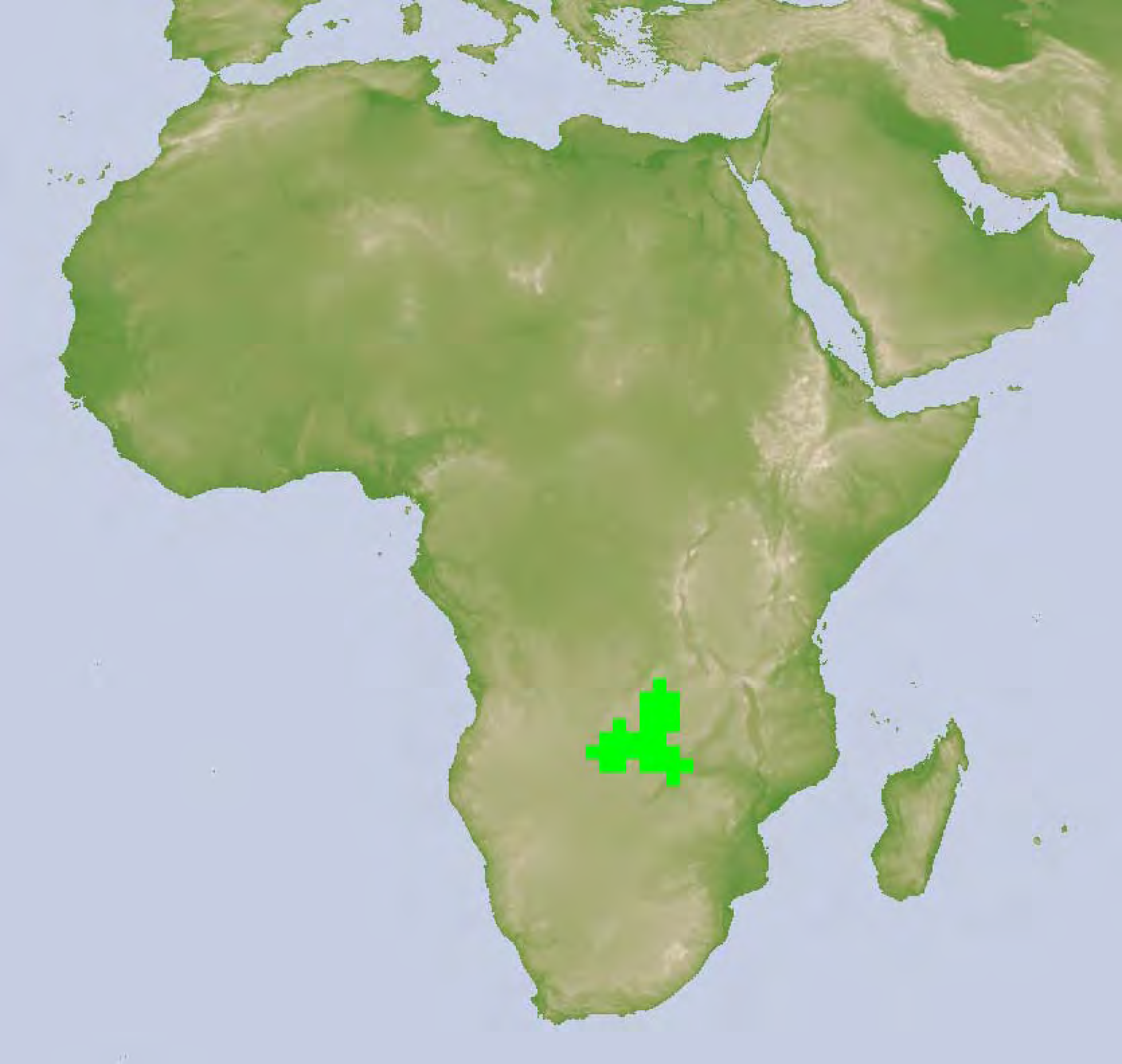

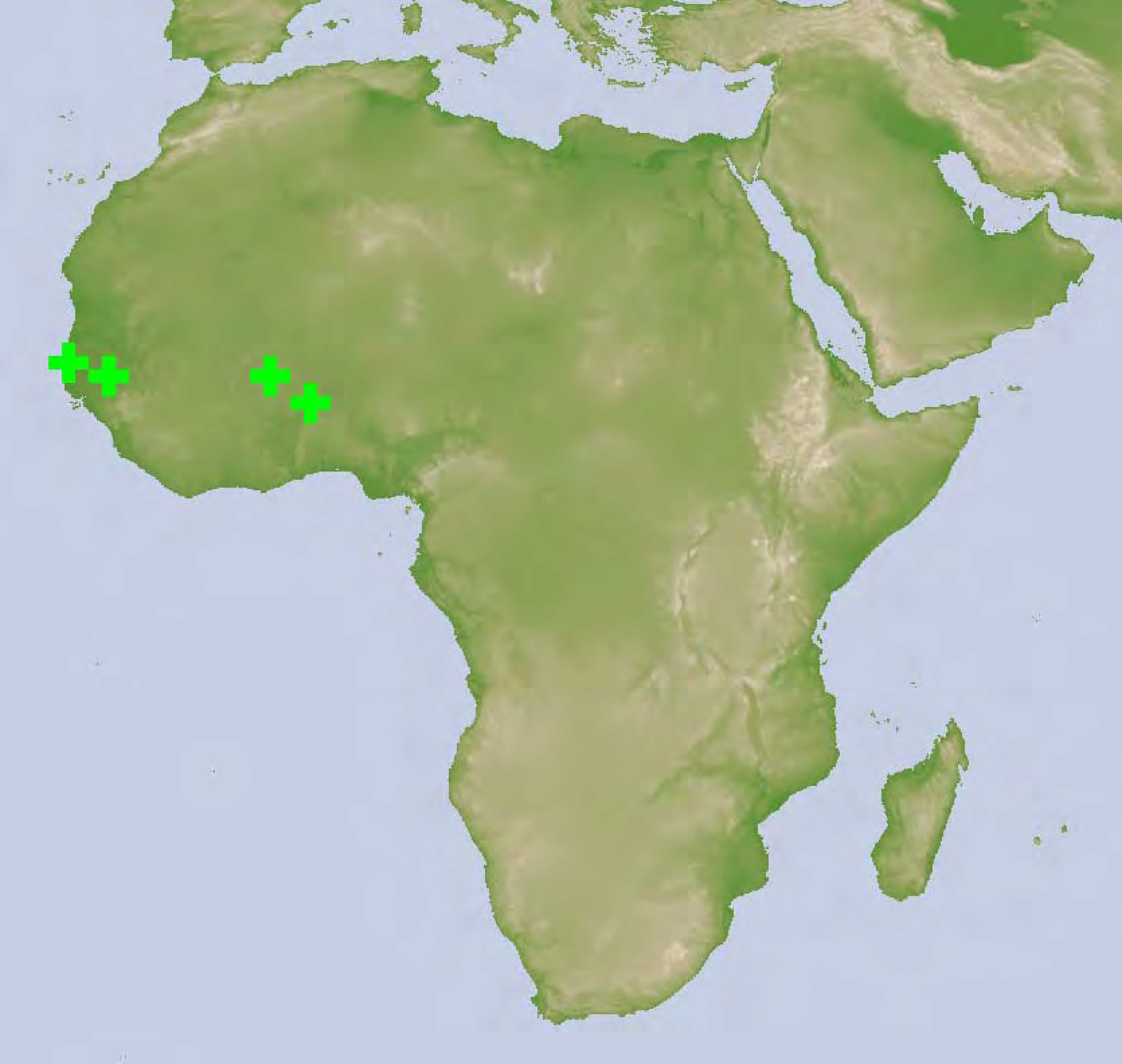

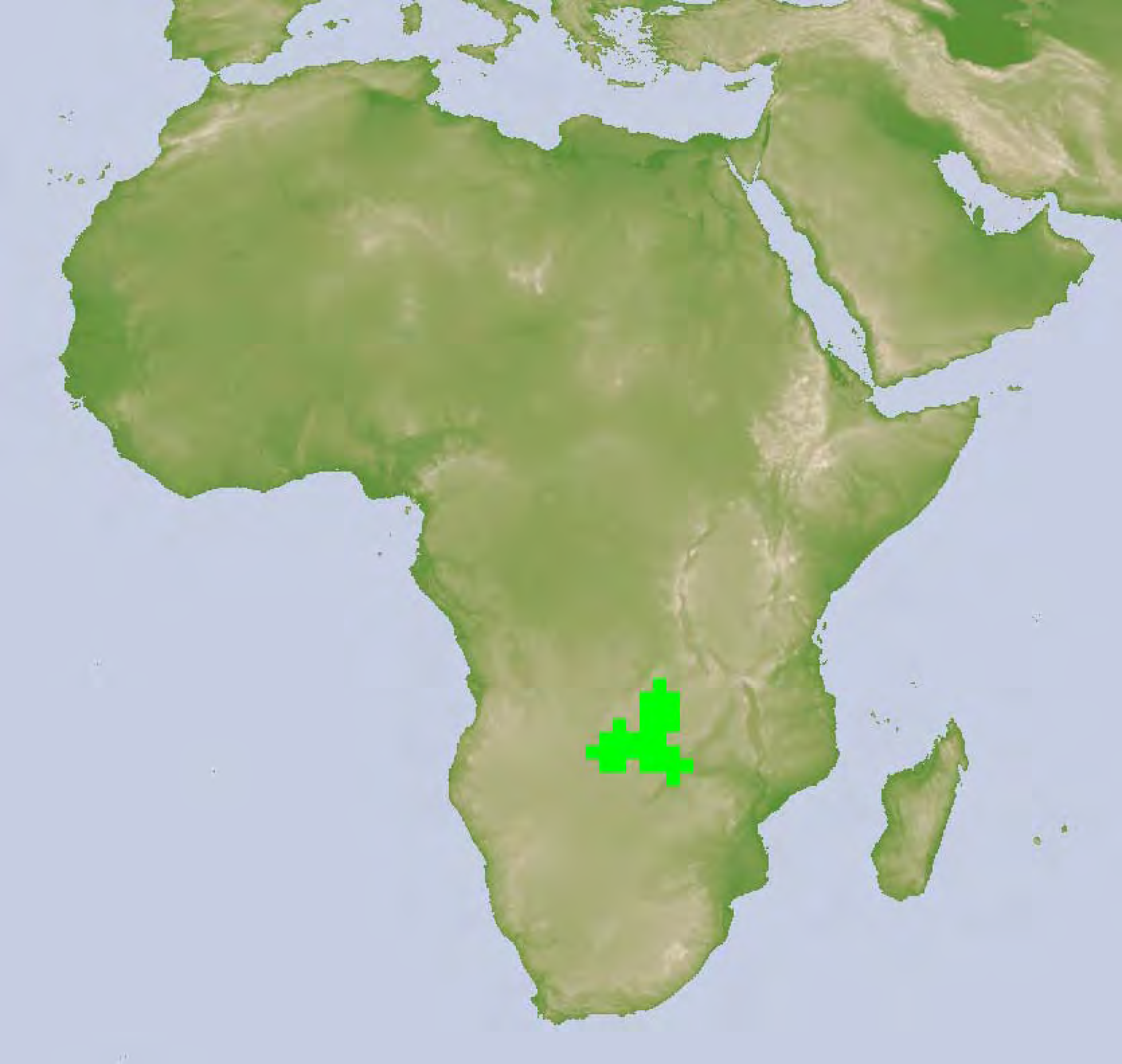

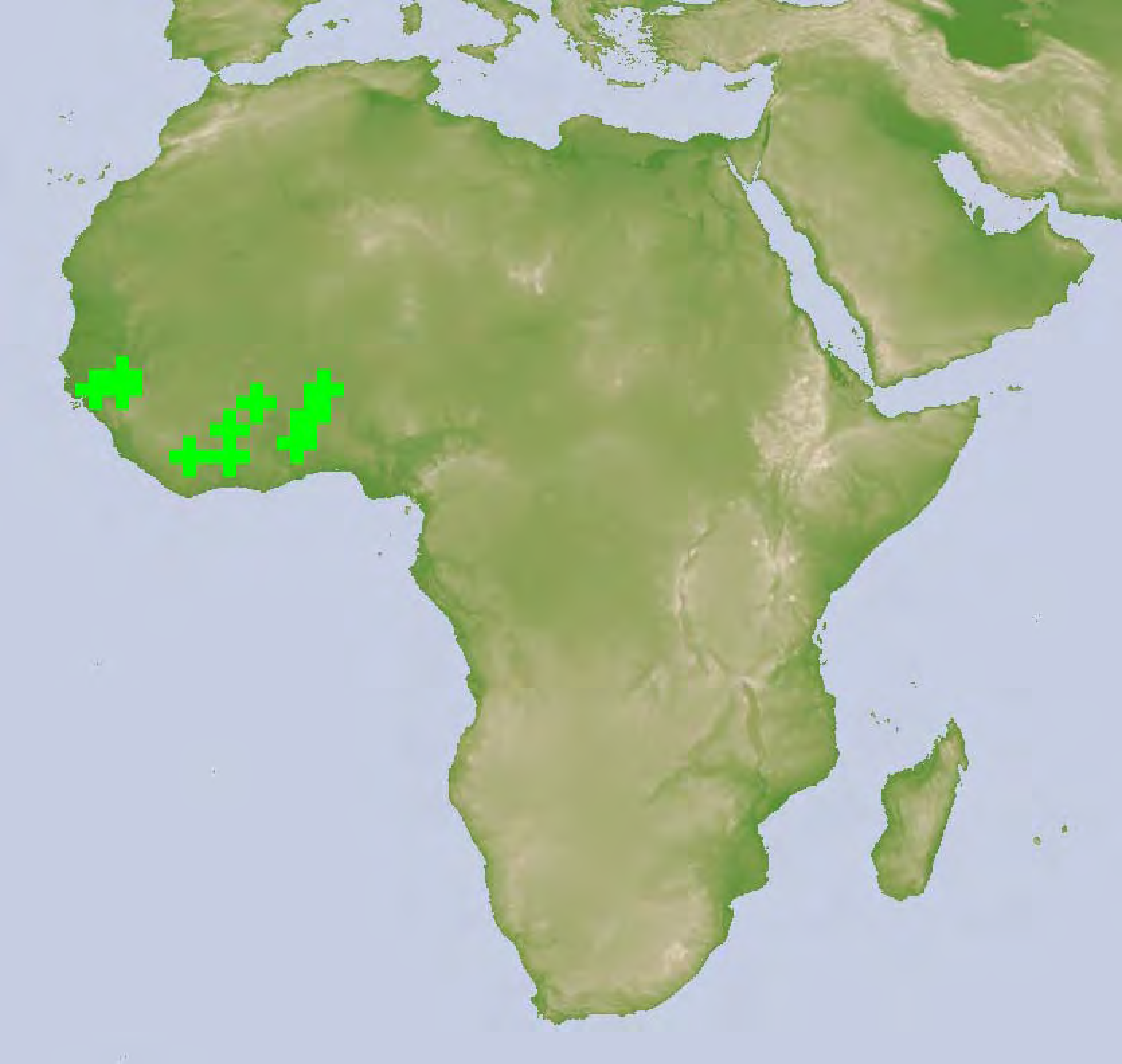

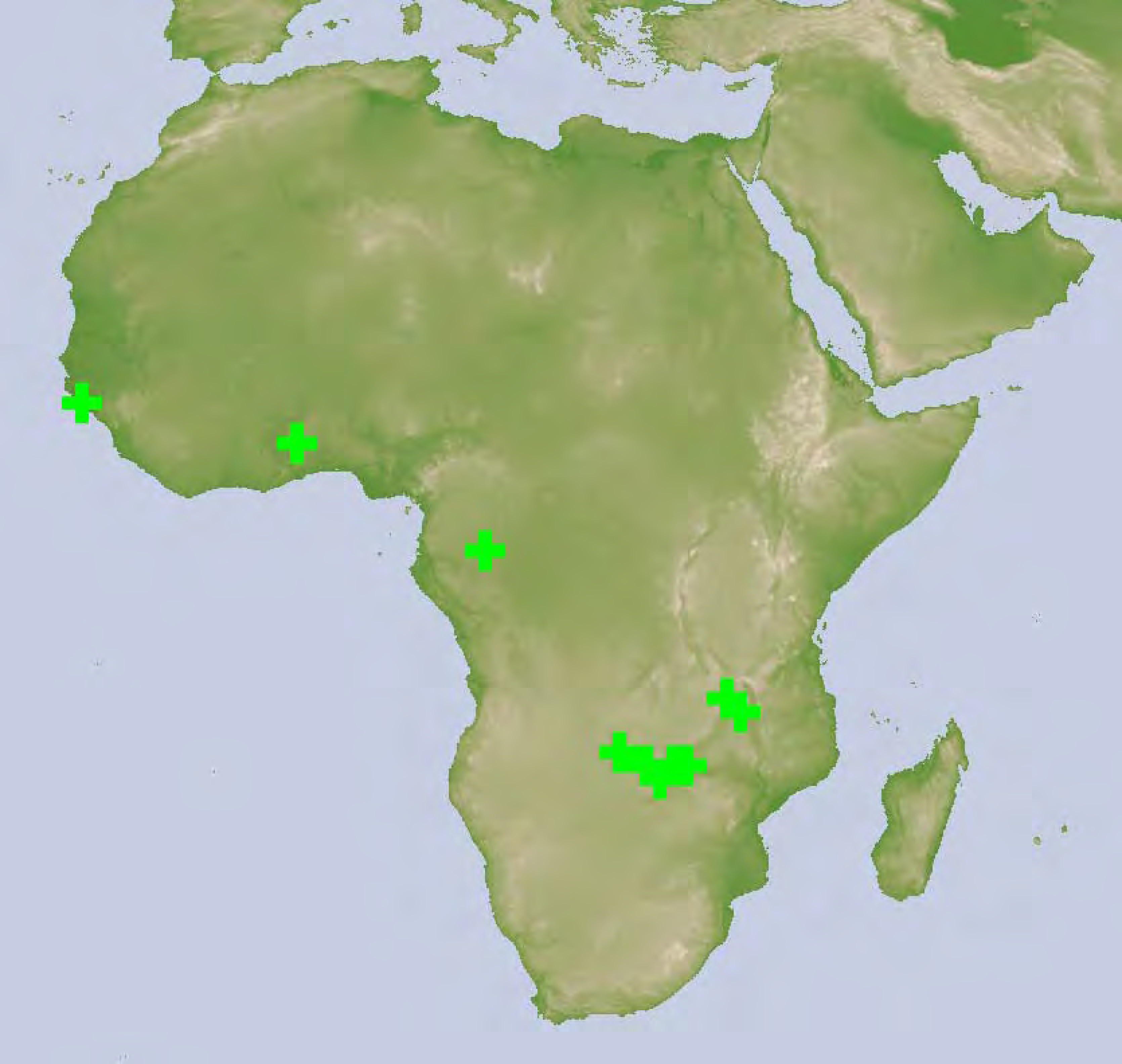

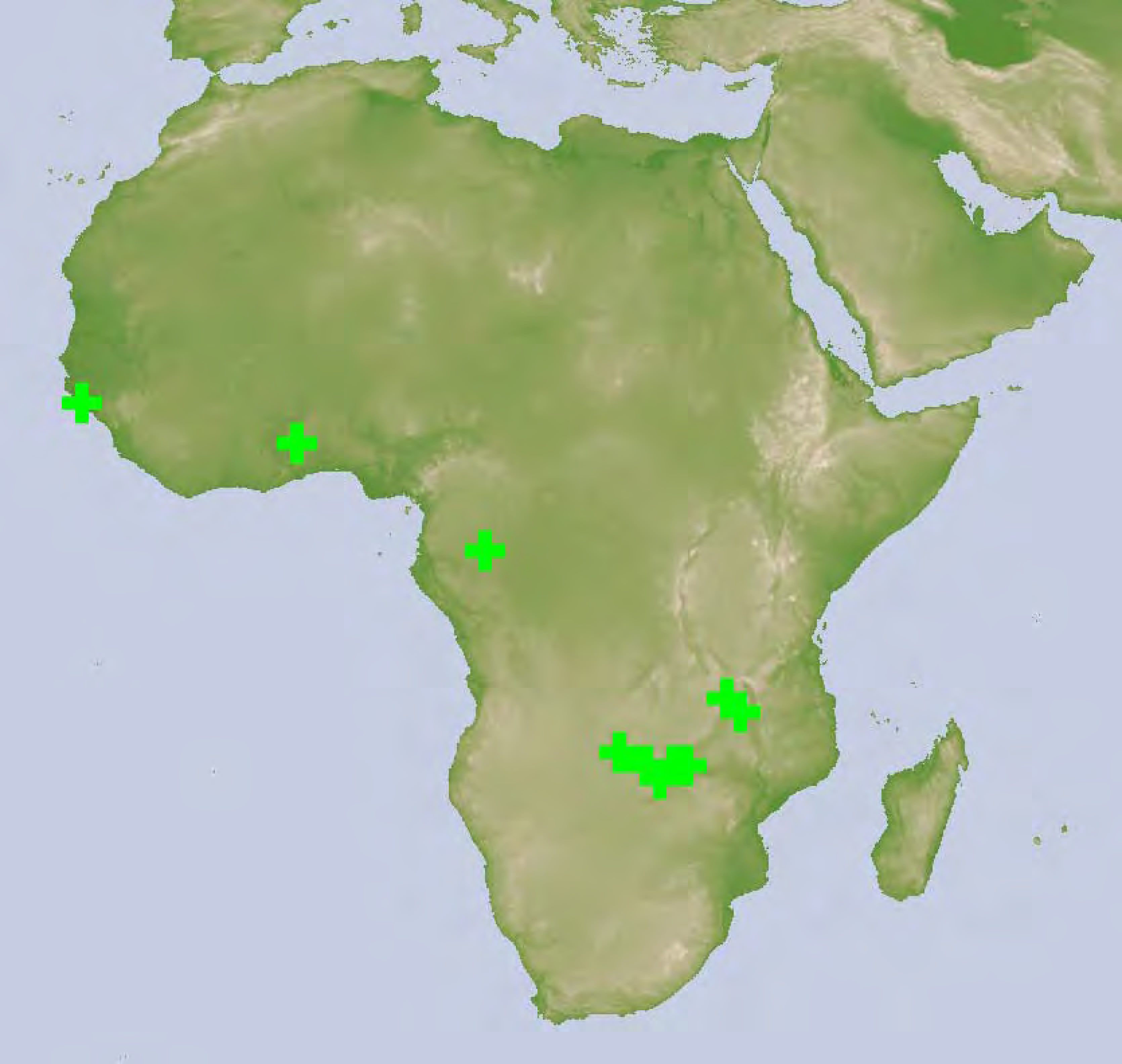

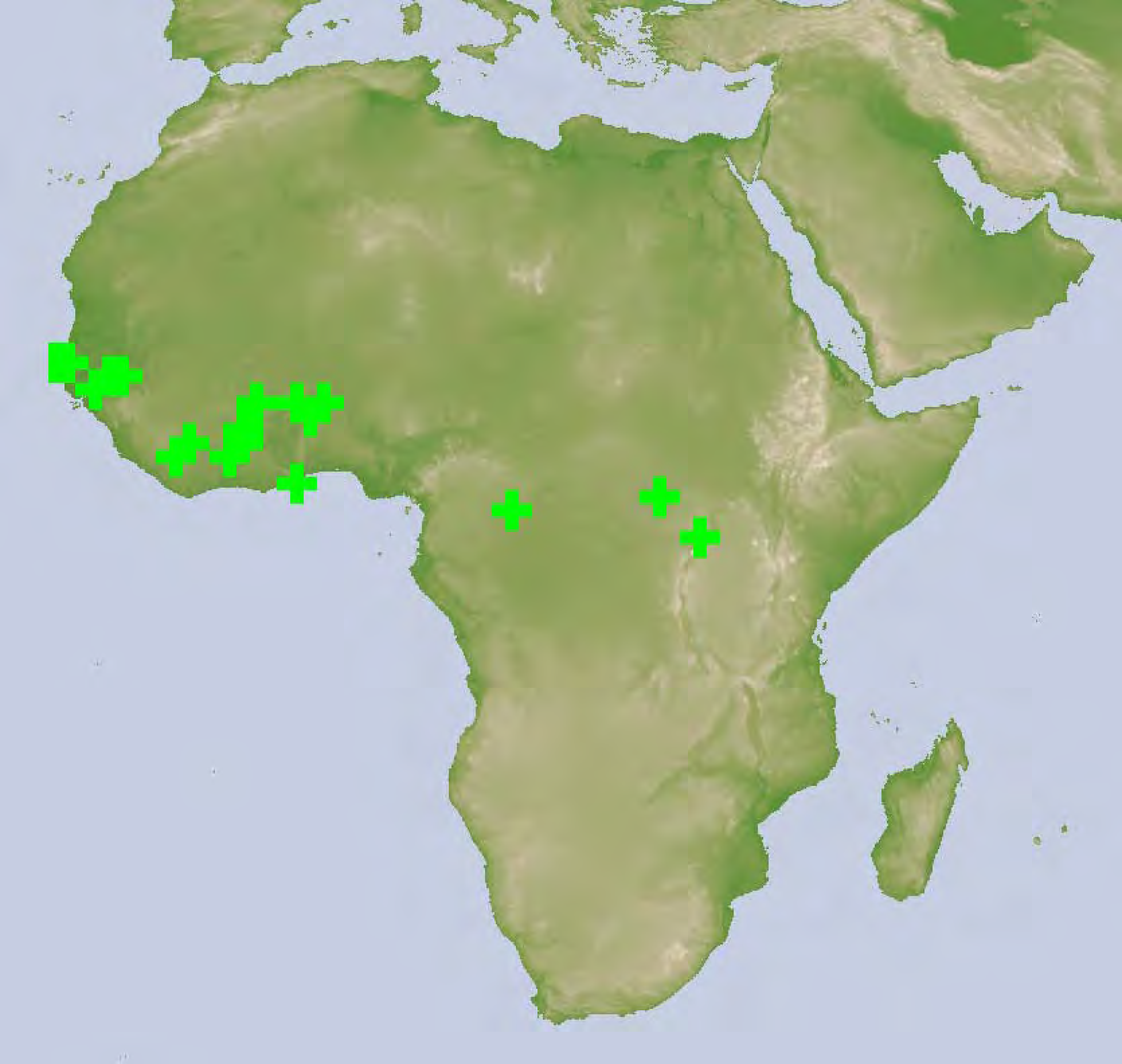

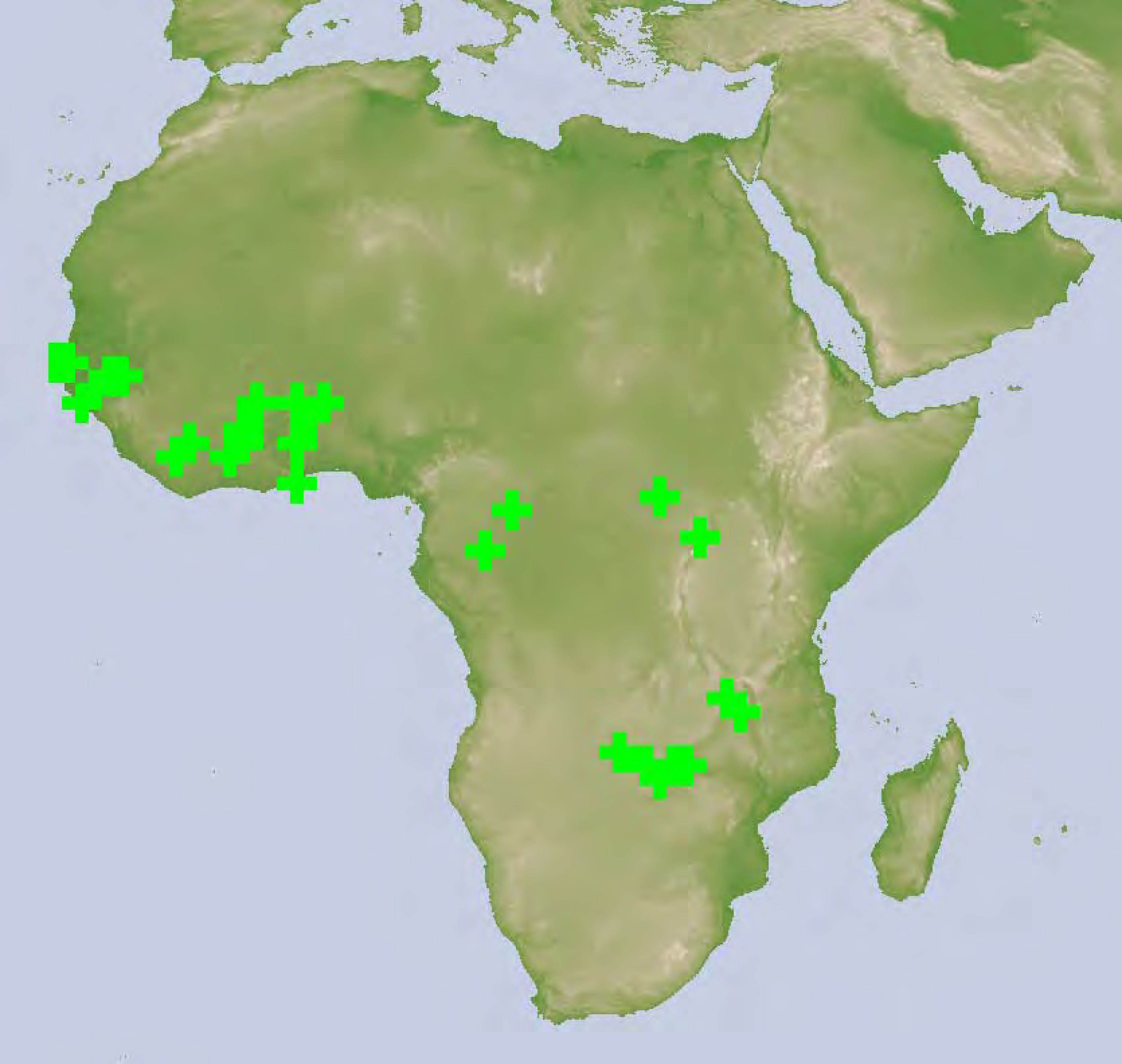

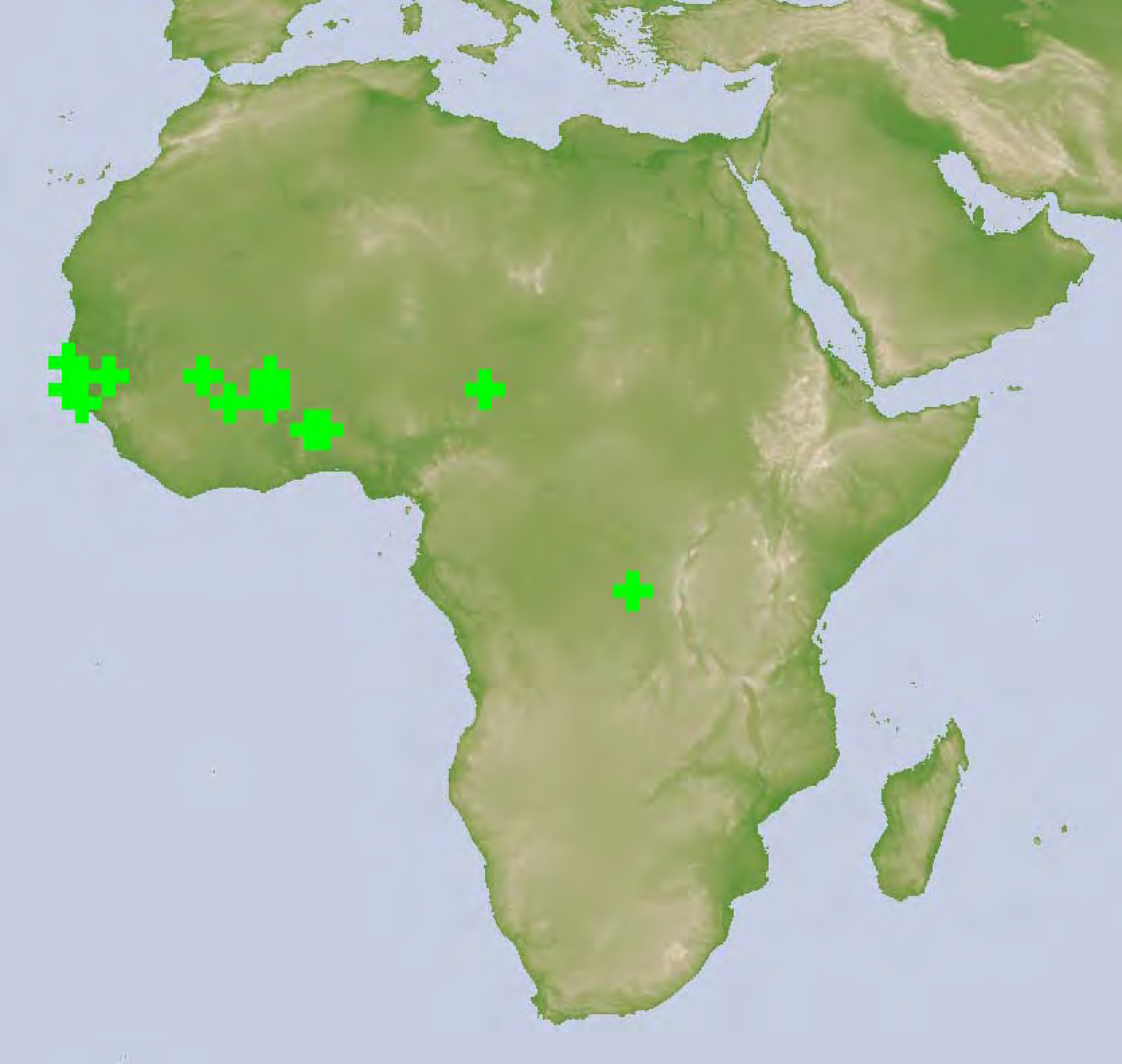

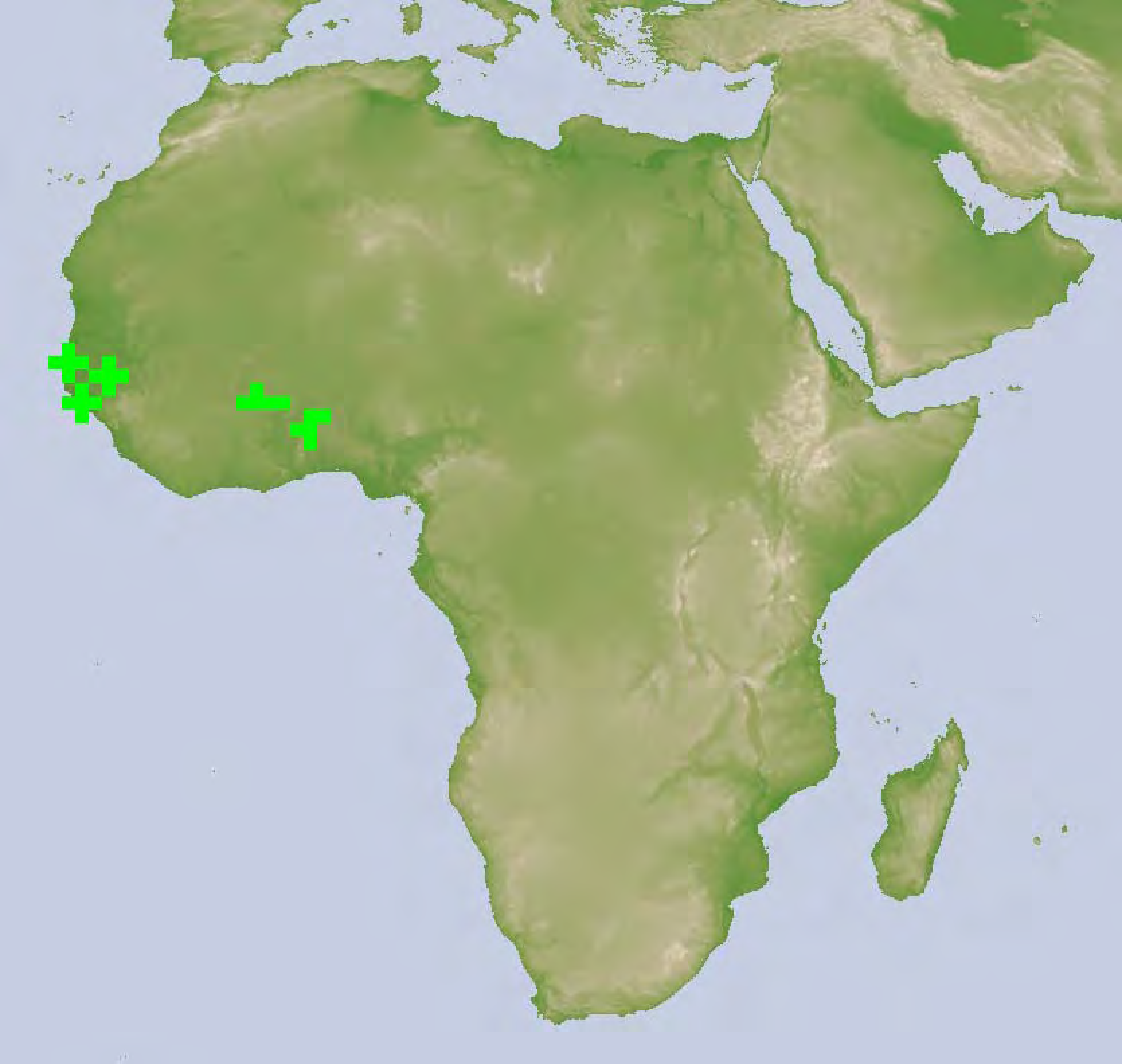

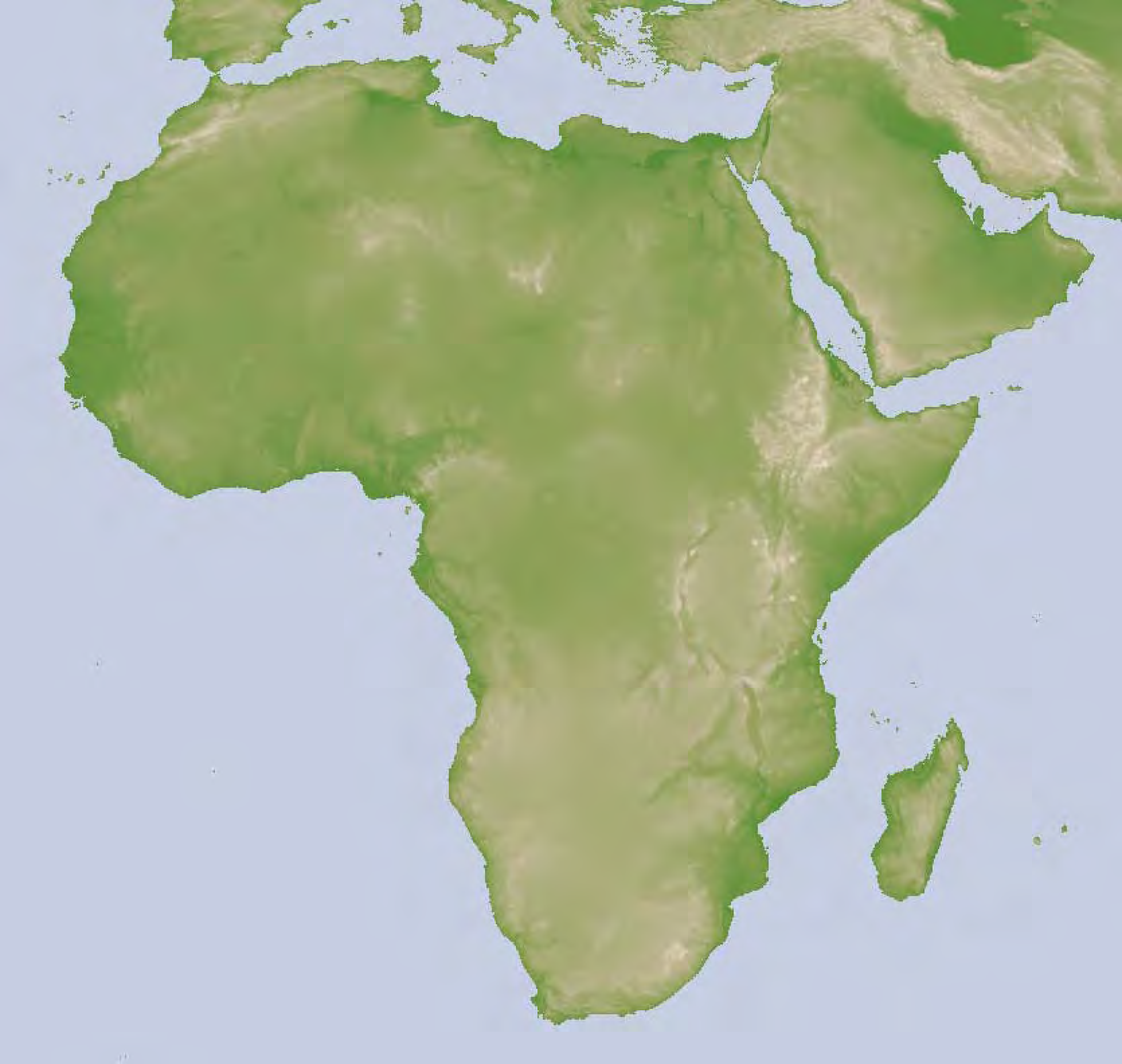

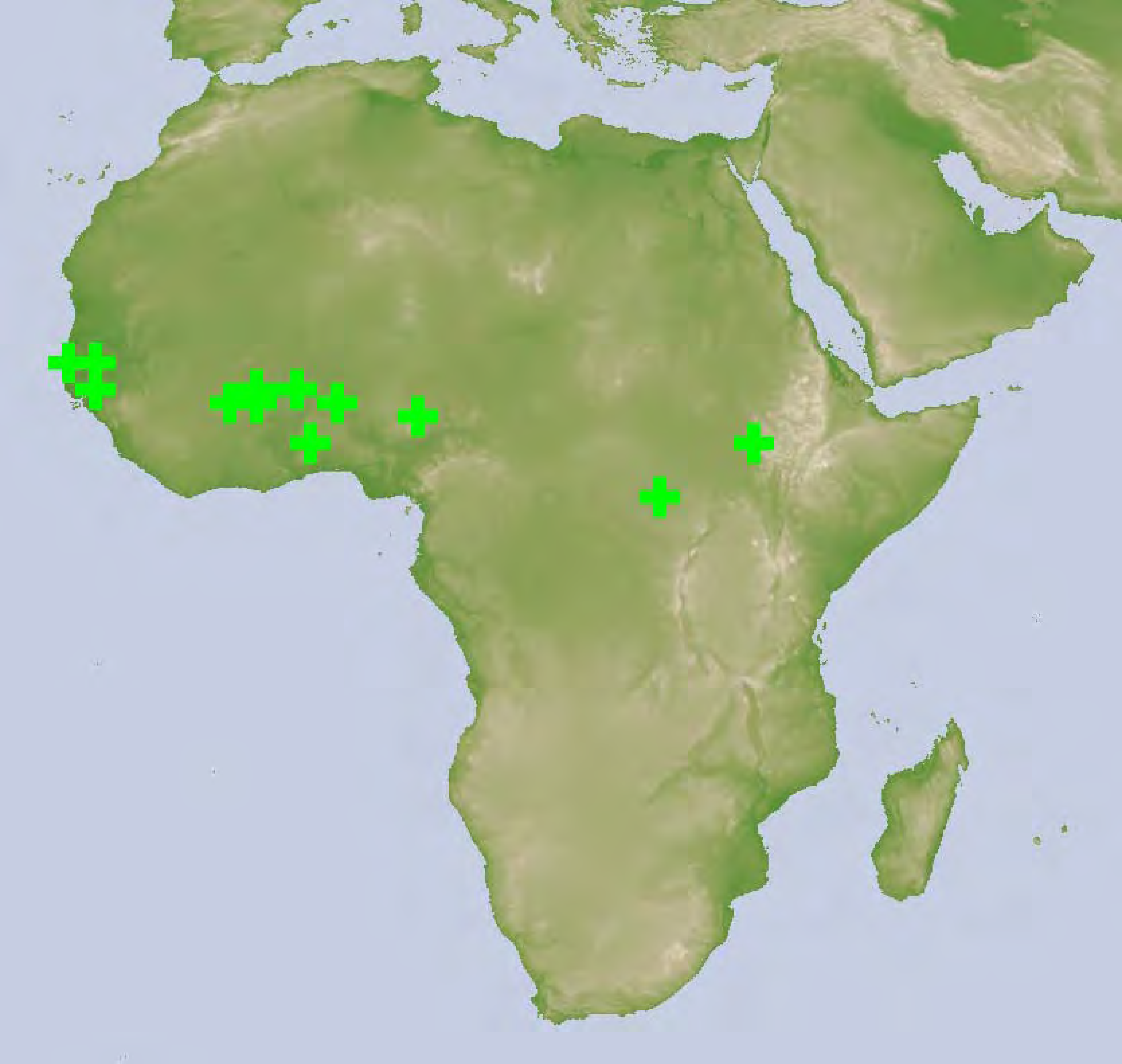

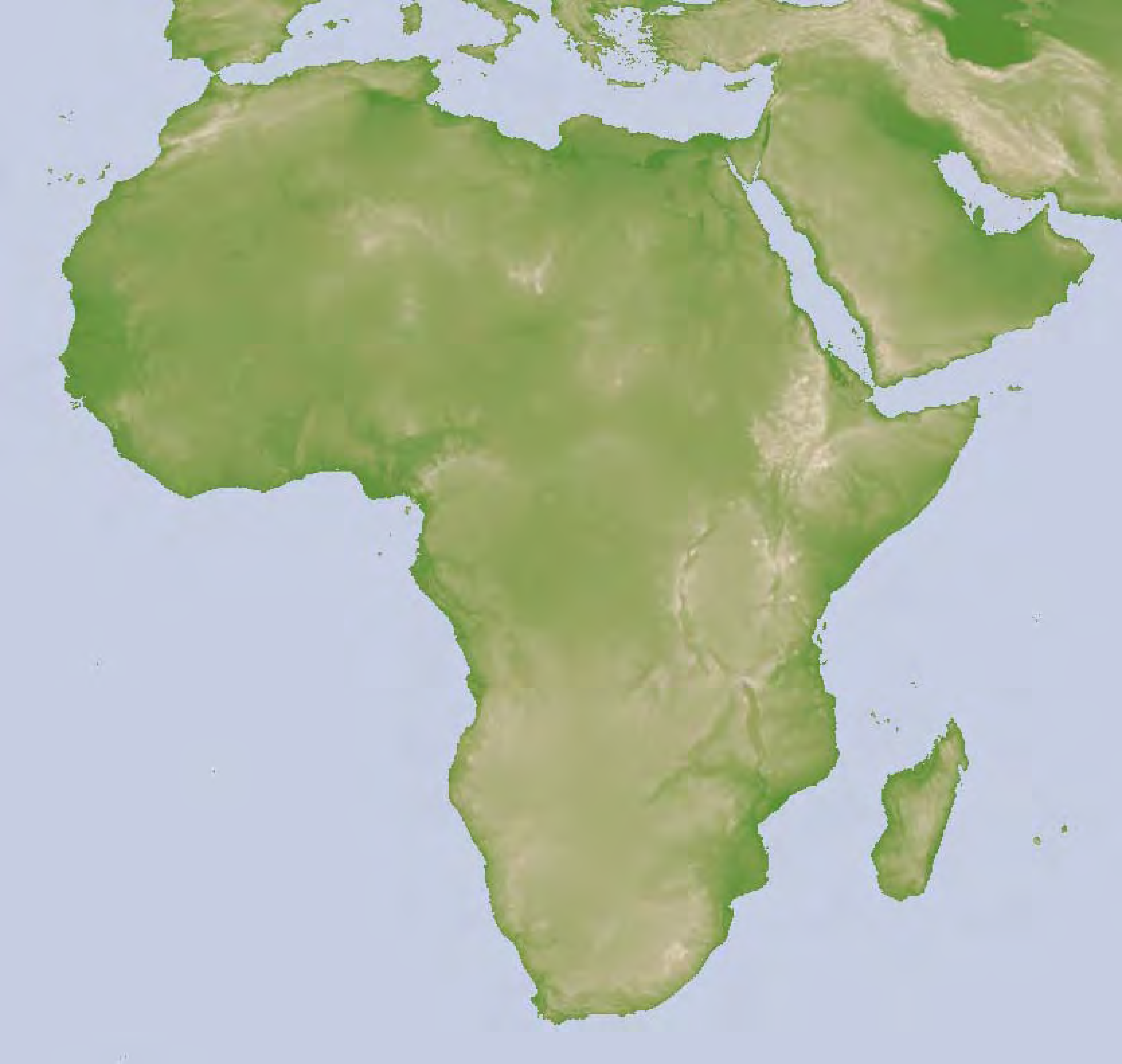

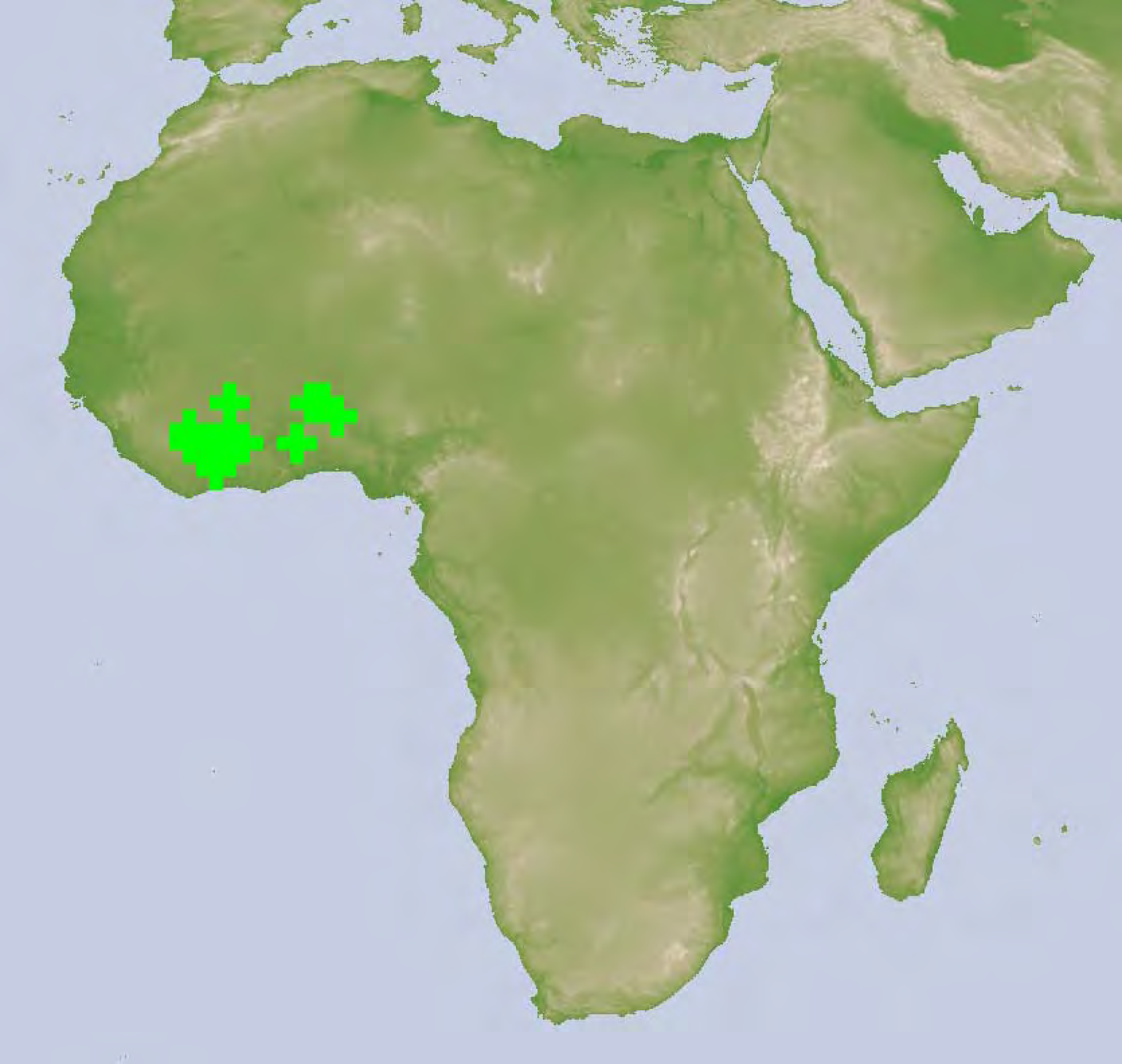

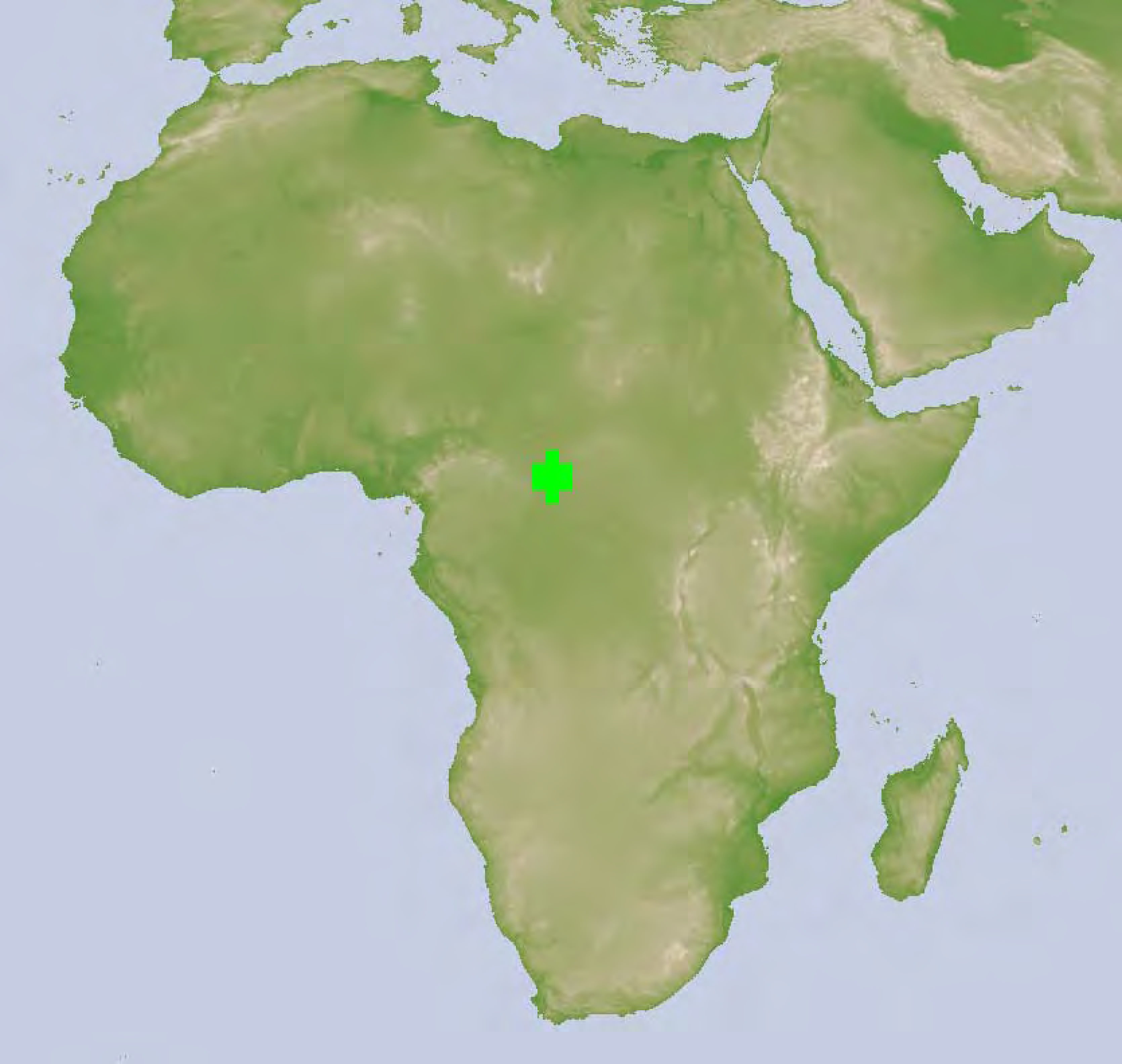

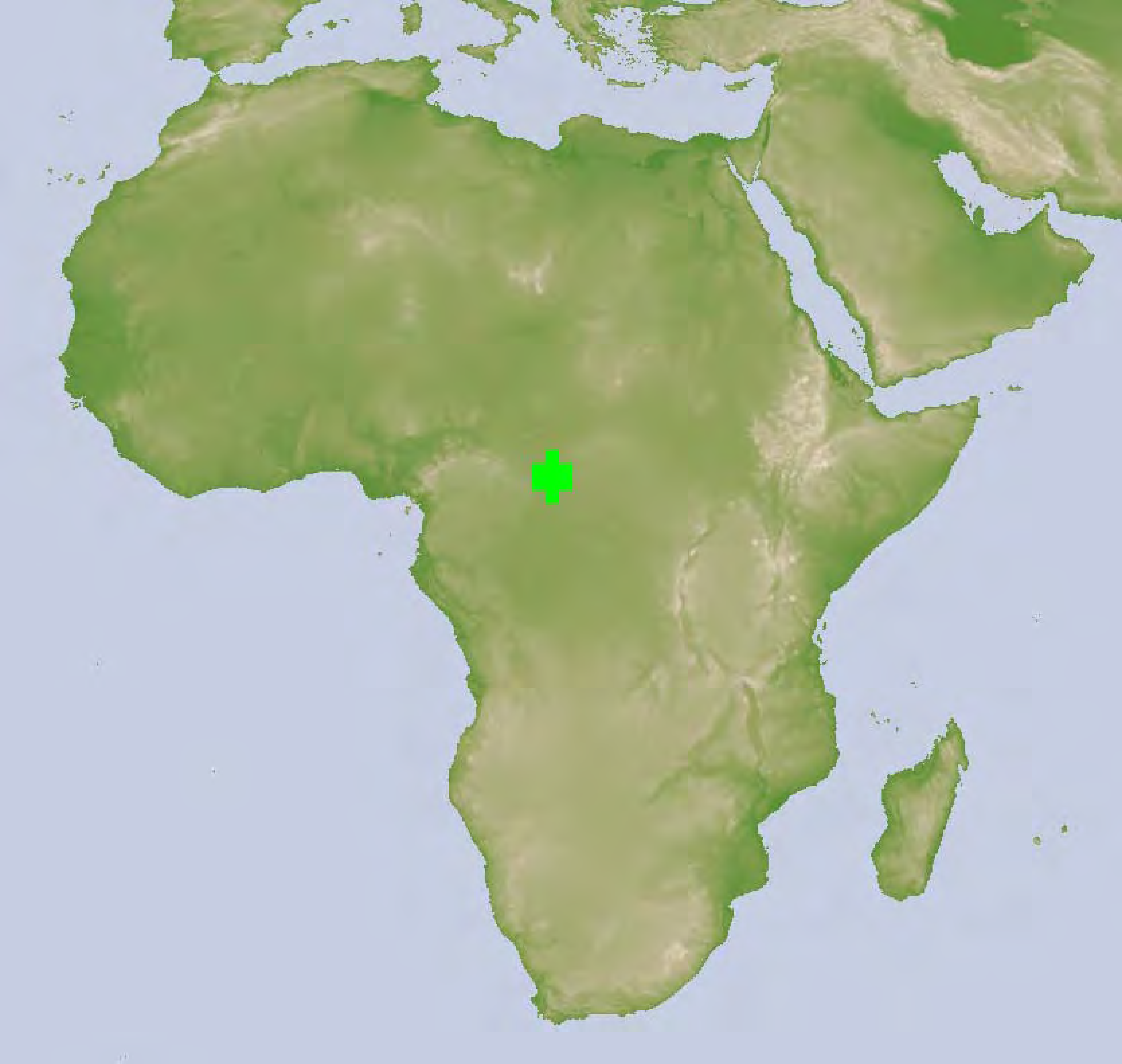

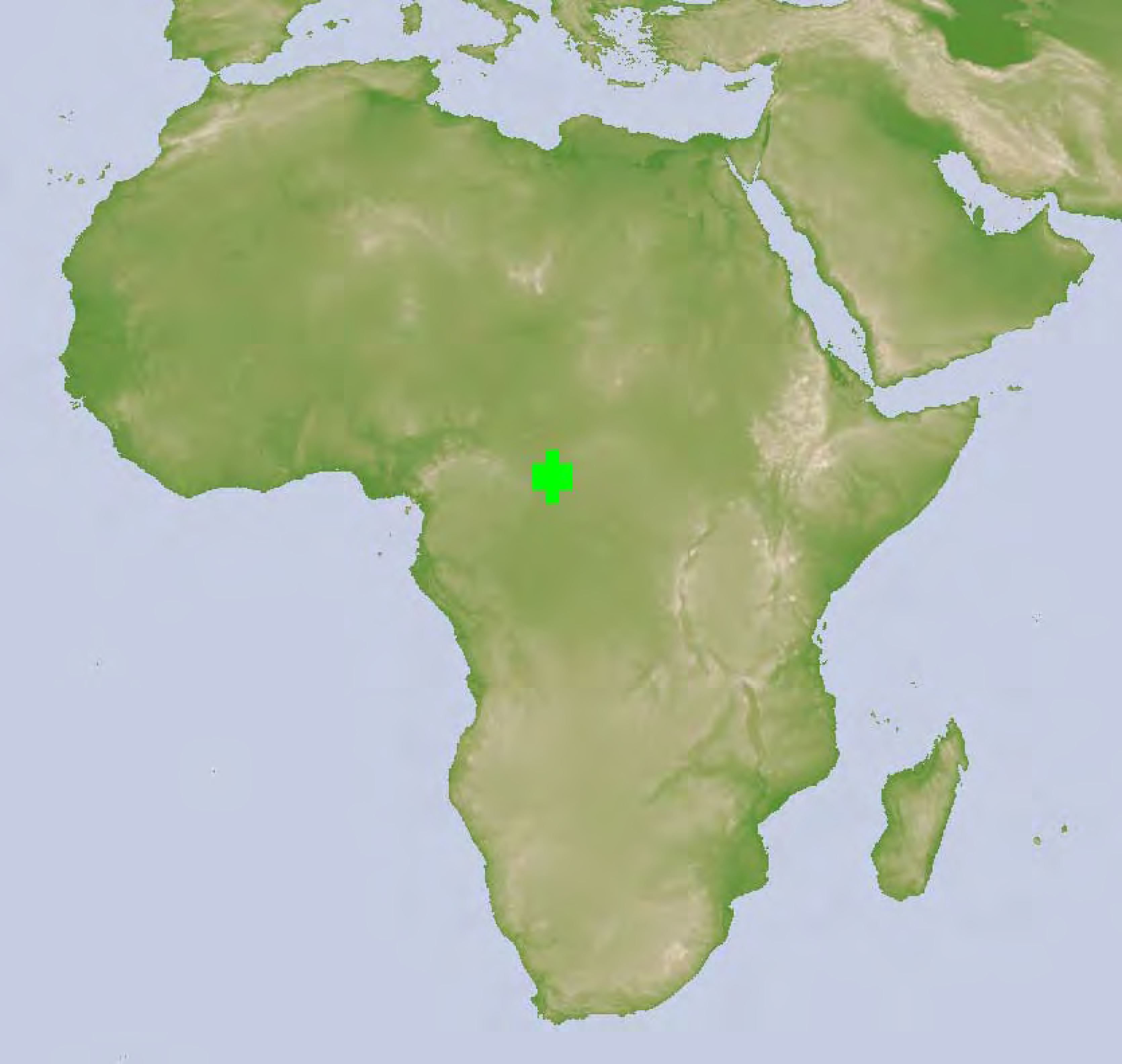

Supplement: Supplementary file 1 [file insects-10-00064-s001.zip › Roggero Data S4 VIP Results.pdf]
