# Supplementary material for: The Phylogenetic Relationships of Tiaronthophagus n.gen. (Coleoptera, Scarabaeidae, Onthophagini) Evaluated by Phenotypic Characters
Source: Insects. 2019 Mar 1;10(3):64. doi: 10.3390/insects10030064 (PMC6468411; doi:10.3390/insects10030064)
Supplement: Supplementary file 1 [file insects-10-00064-s001.zip › Roggero Data S5 List of the morphological features.pdf]

Appendix 5. List of the major differences of the morphological features among the Tiaronthophagus species

---

female clypeus

ogival-shaped, very wrinkled , with more or less evident points

rufopygus; jossoi; macroliberianus; liberianus; pseudoliberianus

rounded with a notch in the middle, the margin up-turned, more or less wrinkled, with large points on sides

viridiaereus; hemichlorus; lamtoensis; chrysoderus; flexicornis; delahayei; rufobasalis; katanganus; aequatus; angolensis; rougonorum; rufostillans; naevius; ebenus; curtipilis; pendjarius; rolandoi; schaufussi; zavattari; zambesianus

slightly triangular-shaped,anteriorly truncated, wrinkled, with some large points on clypeus surface

saadaniensis

male clypeus

largerly ogival, anteriorly margin up-turned , small points on the whole surface, with some larger ones near the genal sutura

angolensis; rufostillans

ogival, the anterior margin up-turned, smooth, with large and larger points mixed

zambesianus

rounded with a notch in the middle, the margin up-turned, smooth, with large points on sides and smaller ones in the middle

viridiaereus; flexicornis

rounded, with some large points mixed to smaller ones, the anterior margin up-turned

schaufussi; zavattari; chrysoderus; rougonorum; lamtoensis; rolandoi; naevius

rounded, the anterior margin up-turned, extremely smooth, some large and many small points

delahayei; aequatus

rounded, the margin slightly notched and up-turned anteriorly, a dense puncture on clypeus

curtipilis

sightly ogival, the anterior margin up-turned and slighly notched with many large and smaller points

pendjarius

slightly ogival, clypeus surface smooth, with some large points mixed to smaller ones, the anterior margin up-turned

katanganus; ebenus; rufobasalis

slightly triangular-shaped,anteriorly truncated, wrinkled, with some large points on clypeus surface

saadaniensis

triangular-shaped, the anterior margin up-turned, with many large and small points mixed

jossoi

triangular-shaped, the anterior margin markedly up-turned, smooth with few large points mixed to smaller ones

macroliberianus; liberianus; pseudoliberianus

triangular-shaped, the anterior margin truncated and up-turned, reletively wrinkled with some large points

rufopygus

male not seen

hemichlorus

vertex carina of female

carina large, reaching the eyes, tricuspidal
liberianus; pseudoliberianus

elongate, triangular-shaped, large, almost reaching the eyes
macroliberianus

lamina very small, trapezoidal straight
lamtoensis

large, reaching the eyes, short, trapezoidal with the base large
pendjarius; rolandoi; schaufussi; zavattari; katanganus

large, rectangular, lowered
jossoi

large, superior magin tricuspidal
delahayei

narrow, small, the superior margin triangular-shaped
curtipilis

narrow, small, the superior margin tricuspidal, straight
rufostillans; angolensis

small, narrow, rounded
zambesianus

short lamina with the base large, almost reaching the eyes
flexicornis

short lamina with the base narrower (never reaching the eyes)
chrysoderus

small , never reaching the eyes, triangular-shaped, short
rufopygus

small pyramidal, placed near the hind margin
saadaniensis

small, narrow, short, trapezoidal-shaped, slightly concave
ebenus, rufobasalis

small, straight triangular-shape, the apex rounded
viridiaereus

small, trapezoidal, relatively narrow
rougonorum

small, tricuspidal, with the central apex higher then the side ones
aequatus

triangular, small up-turned
hemichlorus

tricuspidal, rather large, straight, narrow
naevius

vertex carina of major male

almost ovalar lamina, carrying superiorly an arched cylindrical, short and straight expansion
liberianus; macroliberianus; pseudoliberianus

lamina elongate, trinagular-shaped rectilinear, the apex rounded, never carrying a cylindrical projection
rufopygus

lamina rectangular shaped, very short, then tapering into a triangle and carrying apically a cylindrical long and straight expansion
lamtoensis

lamina trapezoidal inverted , tapering to apex, carrying a long cyliindrical straight projection
rolandoi; schaufussi; zavattari

narrow, very small, never reaching the eyes, straight, elevated, truncated at apex
rougonorum

rectangular lamina but narrowed at the base, carrying superiorly an arched cylindrical expansion anteriorly turned
angolensis; rufostillans; zambesianus

rectangular lamina very narrow, with two pointed expansions on sides and a central straight, short protuberance
jossoi

rectangular lamina, carrying superiorly an arched cylindrical expansion anteriorly turned
chrysoderus

rectangular lamina, carrying superiorly an arched cylindrical, short and straight expansion
flexicornis; katanganus

rectangular, short , tapering to apex and carrying a cylindrical projection
pendjarius; viridiaereus

rectangular, straight, the apex rectilinear
curtipilis

rectangular-shaped, apically tapering and carrying a short rectilinear cylindrical projection
naevius

sinuate rectangular lamina, carrying superiorly an arched cylindrical expansion anteriorly turned
aequatus

sinuate rectangular lamina, in the distal third tapering into a triangle carrying superiorly an arched, small cylindrical expansion anteriorly turned
ebenus; rufobasalis

not seen
delahayei; hemichlorus; saadaniensis

Pronotum disc sculpture

evident, relatively small, thick, ovalar granules
ebenus

evident, rounded, quite large granules, well spaced
aequatus

large ovalar evident granules relatively dense
rufopygus

large very thick granules
saadaniensis

large, evidente ovalar thick granules
zambesianus

large, thick granules
chrysoderus

many large points mixed with very small granules
curtipilis

rounded, evident granules, well spaced
hemichlorus

rounded, relatively dense granules
rolandoi; schaufussi; zavattari

rounded-ovalar, thick granules
jossoi

small, rounded thick granules
naevius; pendjarius; liberianus; macroliberianus; pseudoliberianus

small sparse granules
angolensis; rufostillans

many thick, small granules
rougonorum

thick, relatively large, ovalar granules
rufobasalis

thick, rounded granules
delahayei; flexicornis; katanganus; lamtoensis; viridiaereus

female pronotum tubercles

absent

rufopygus

conical, evident, very close at base, the apices pointed

hemichlorus

conical, relatively large, parallel the apicex pointed

viridiaereus

conical, short, very close along the midline, rectilinear, the apex rounded

flexicornis, lamtoensis pendjarius

conical, straight, rectilinear

delahayei

conical, the apex rounded , parallel rectilinear

rolandoi

conical, the apex rounded , slightly diverging at apex

schaufussi; zavattari

reduced, rounded, very close, sometimes forming a unique line

rougonorum; curtipilis

conical, pointed, converging to the base

ebenus; rufobasalis

small conical, the apices pointed, close along the midline, the apices diverging

zambesianus; angolensis; rufostillans; aequatus

small, conical, rectilinear, the apices slightly diverging

chrysoderus

two conical, pointed, parallel, close to the midline

liberianus; pseudoliberianus; macroliberianus

two conical, small, parallel protuberances

naevius

two flat, rounded, expanded, rectilinear projections

katanganus

two rectilinear expansions, straight, very close, expanded, flat on sides

jossoi

two very expanded parallel laminae encircling a large smooth hollow

saadaniensis

Elytral interstriae

evident, very dense granules, evenly distributed on the whole surface
ebenus; rufobasalis

extremely dense, relatively large granules, the surface quite rough
pendjarius; rougonorum; viridiaereus

extremely dense, small granules, the surface almost rough
jossoi

large, uniform flat granules, disposed in two rows
lamtoensis; chrysoderus; curtipilis

small but evident scattered granules, the first two interstriae almost smooth, the points larger and dense on sides
hemichlorus

small, evident, quite dense granules, evenly distributed on the whole surface
liberianus; macroliberianus; pseudoliberianus

small, uniform granules, disposed in two rows
flexicornis; katanganus

very small and sparse granules, the first interstria smooth, the point thicker on sides
schaufussi; zavattari; aequatus; angolensis; delahayei; naevius; rufostillans; rolandoi

very small granules, evenly distributed on the whole surface
rufopygus

very small, evident, regularly distributed in two rows granules
zambesianus

very thick granules, the surface extremely rough
saadaniensis
