# Supplementary material for: The Phylogenetic Relationships of Tiaronthophagus n.gen. (Coleoptera, Scarabaeidae, Onthophagini) Evaluated by Phenotypic Characters
Source: Insects. 2019 Mar 1;10(3):64. doi: 10.3390/insects10030064 (PMC6468411; doi:10.3390/insects10030064)
Supplement: Supplementary file 1 [file insects-10-00064-s001.zip › Roggero Data S6 Identification Plates.pdf]

Paramere

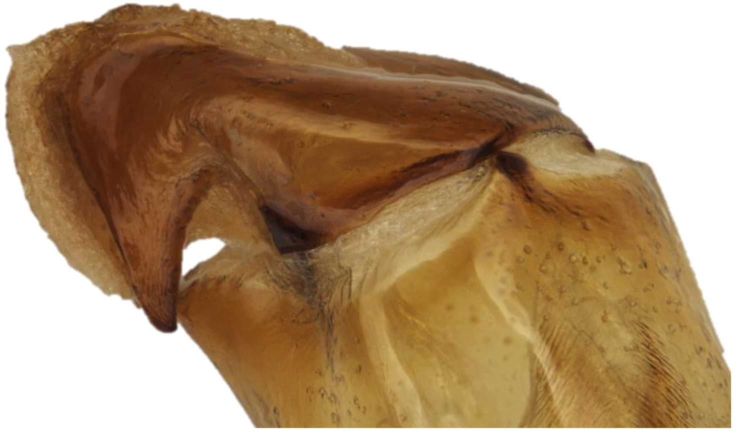

aequatus

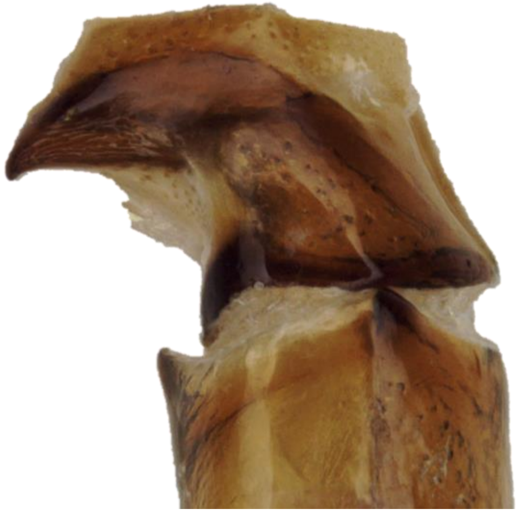

chrysoderus

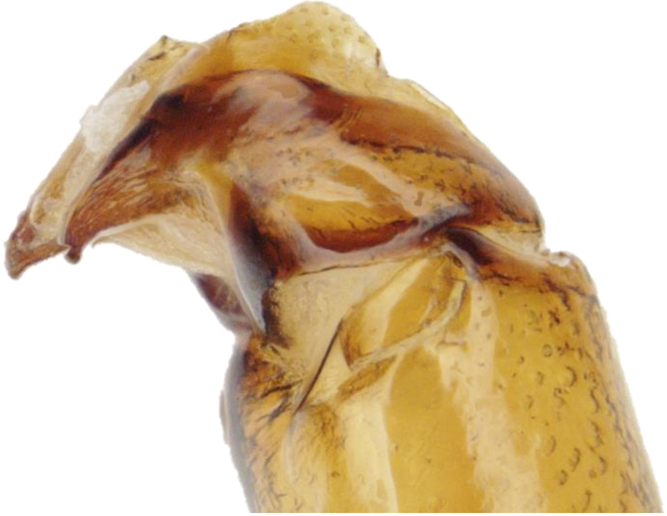

curtipilis

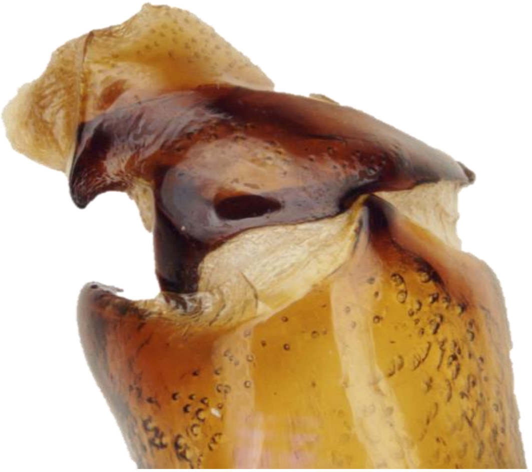

delahayei

Paramere

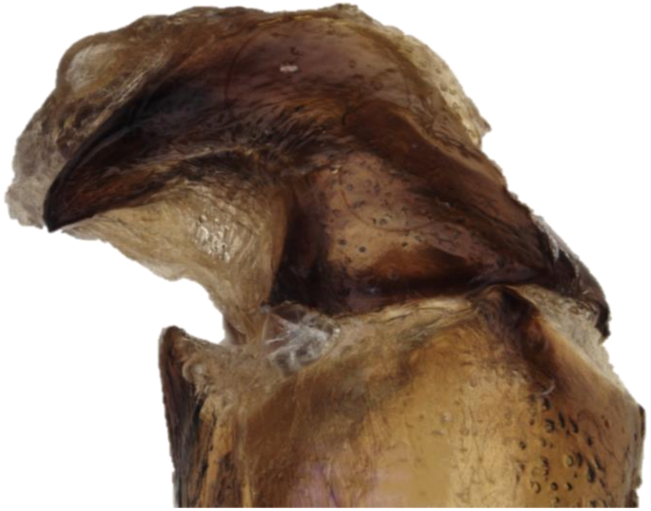

ebenus

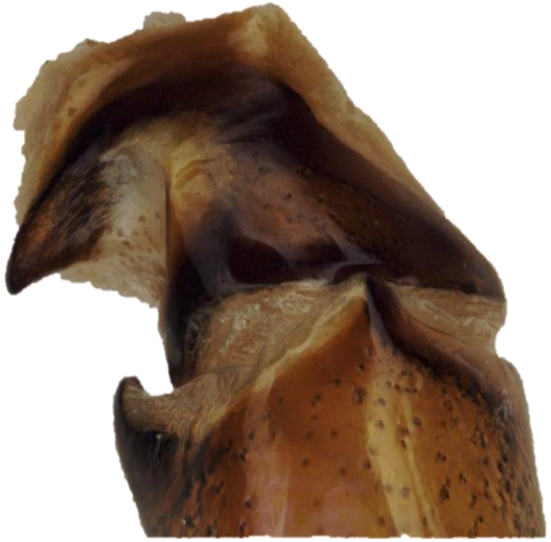

flexicornis

MALE MISSING

hemichlorus

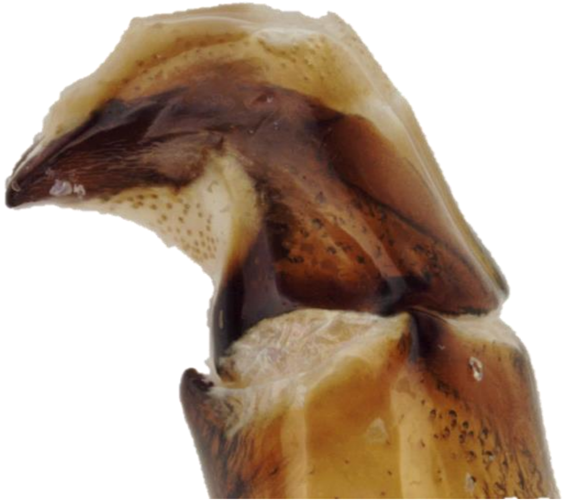

lamtoensis

Paramere

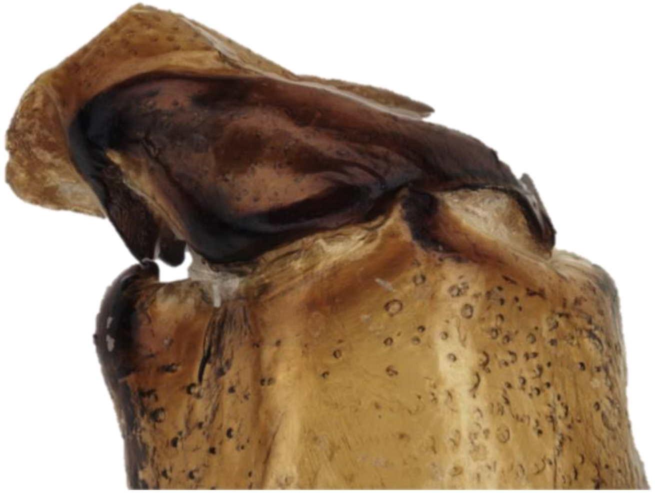

liberianus

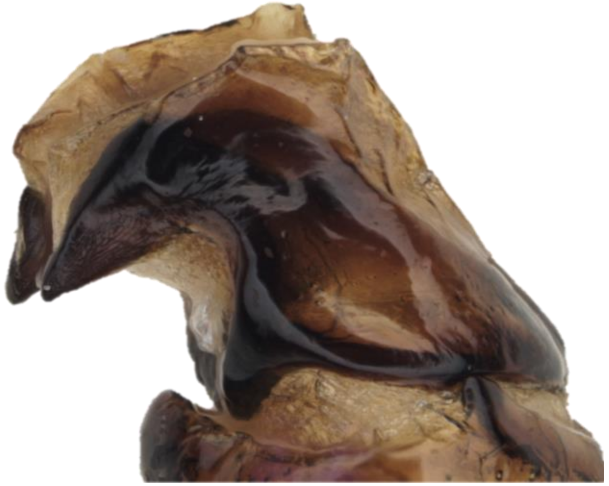

macroliberianus

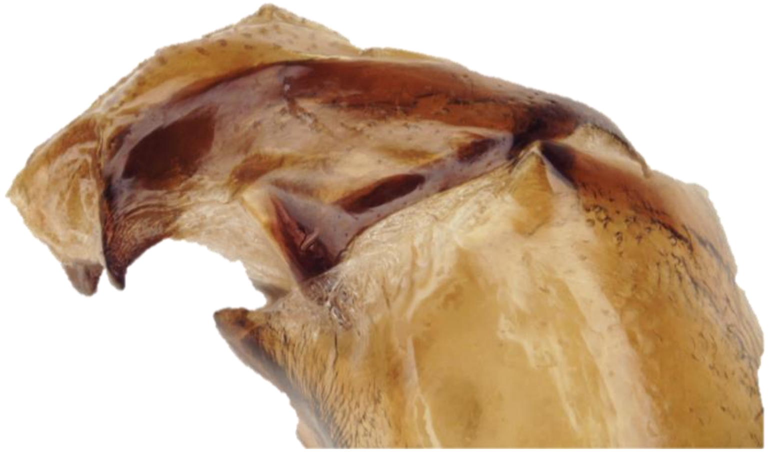

naevius

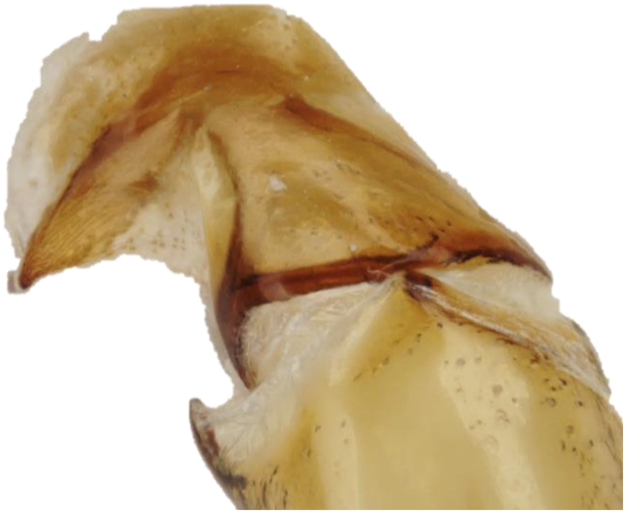

pendjarius

Paramere

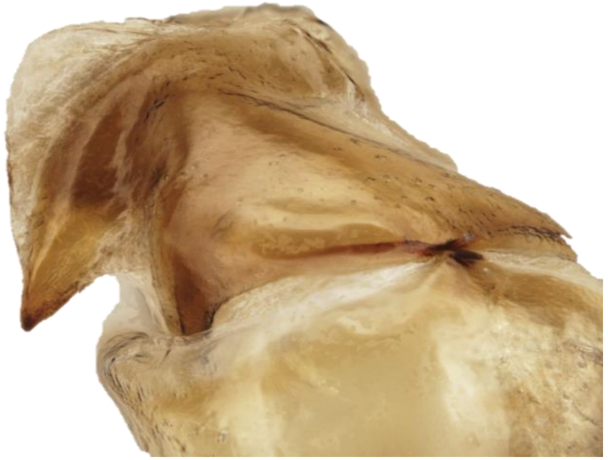

pseudoliberianus

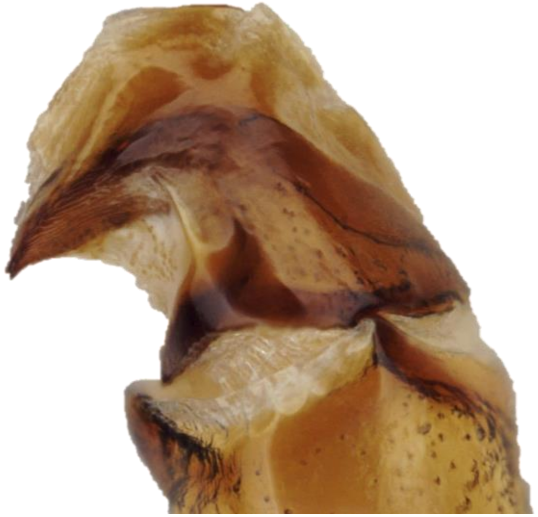

rougonorum

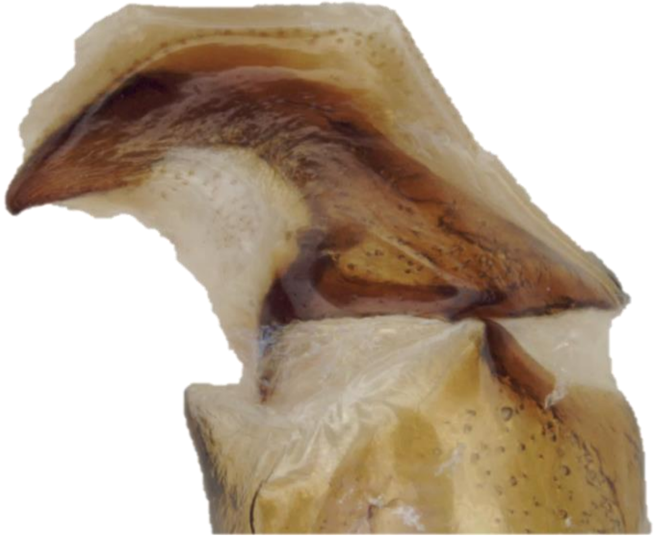

rufobasalis

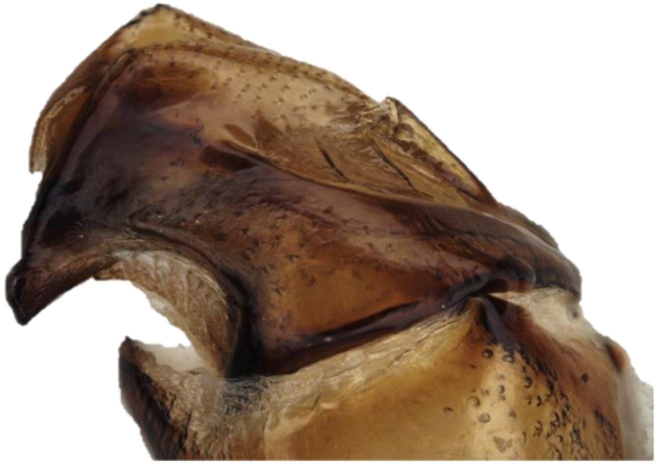

rufopygus

Paramere

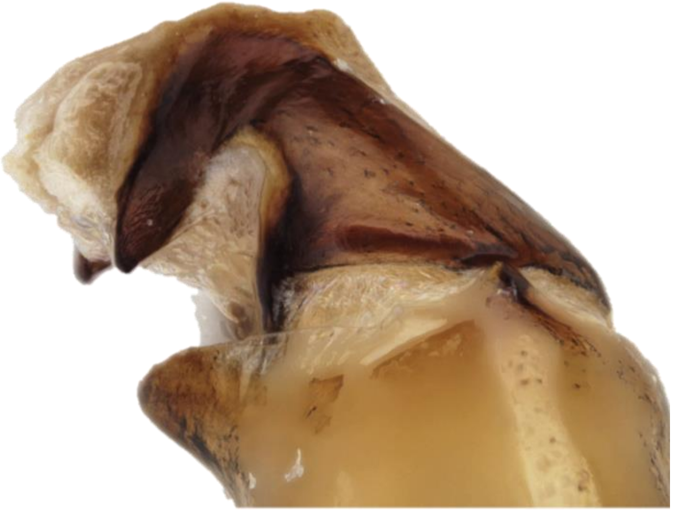

rufostillans

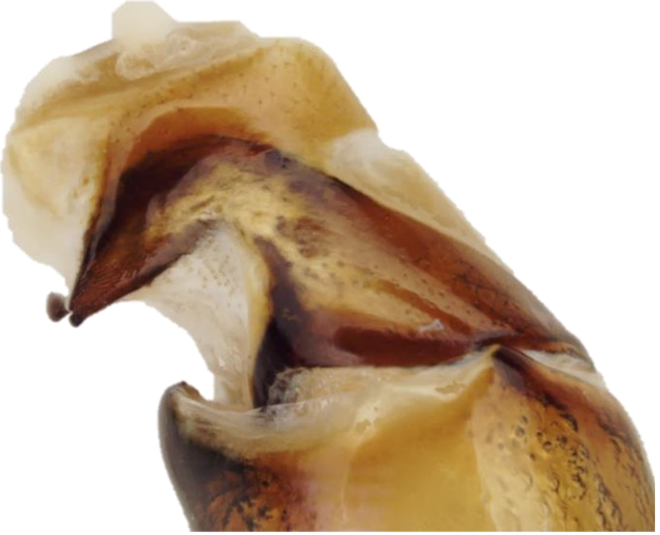

schaufussi

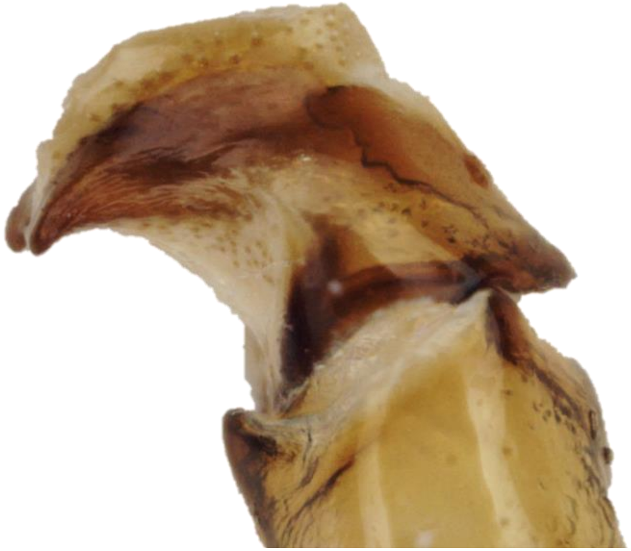

viridiaereus

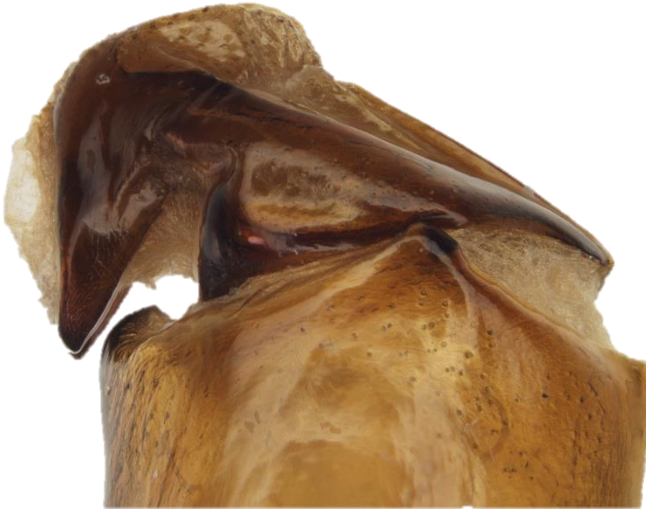

zavattarii

Paramere

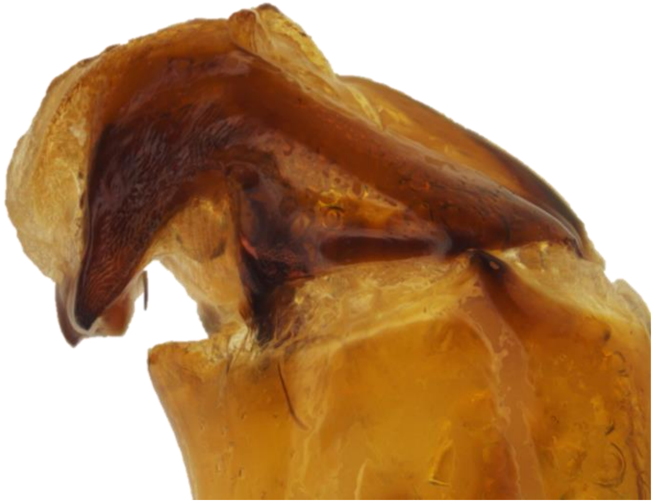

angolensis

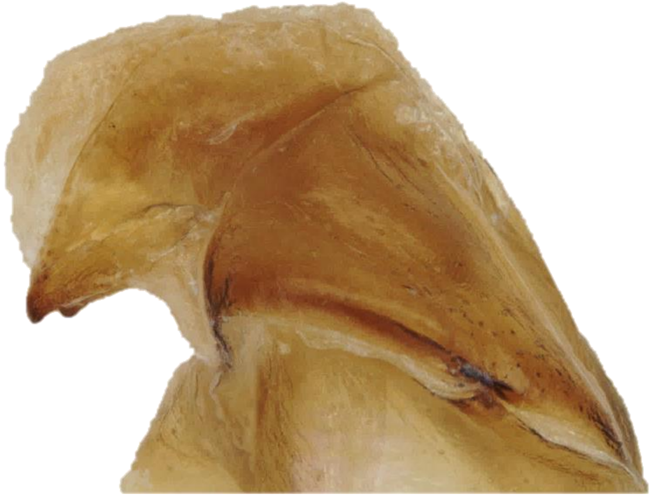

jossoi

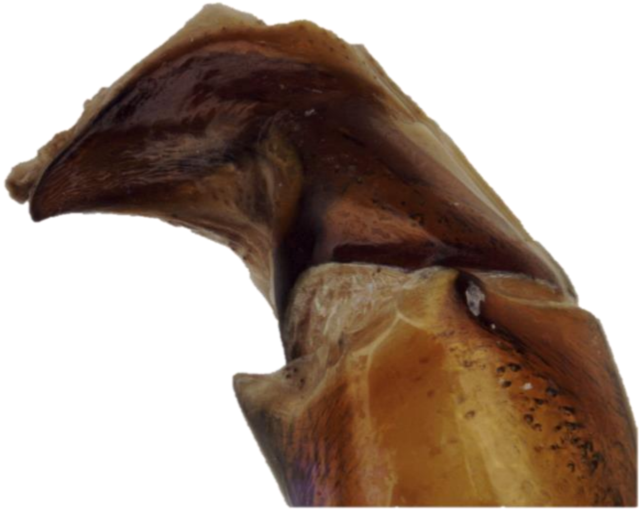

katanganus

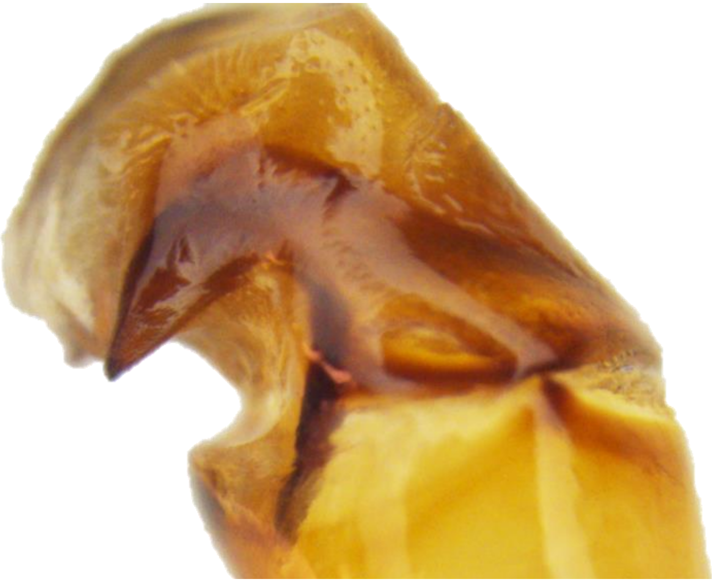

rolandoi

Paramere

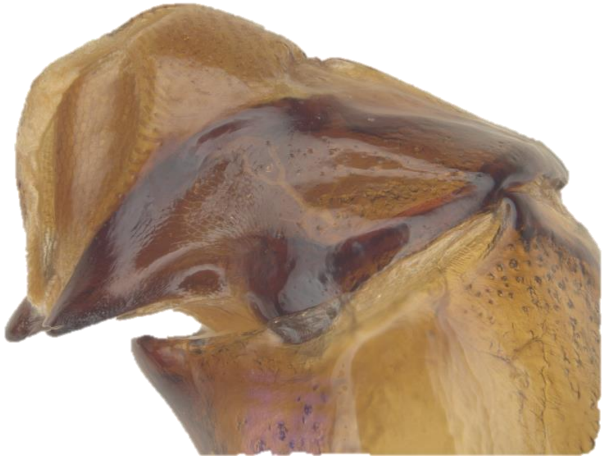

saadaniensis

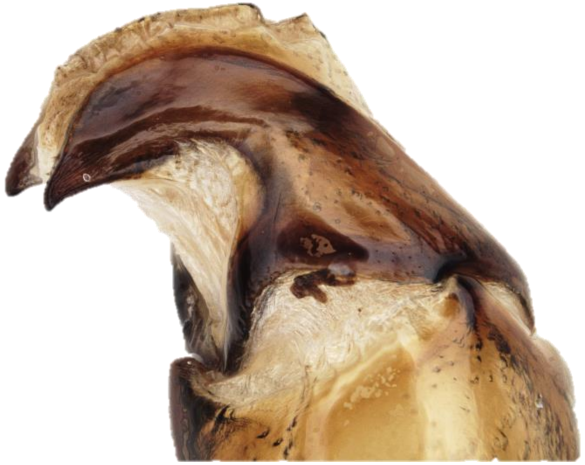

zambesianus

Lamella copulatrix

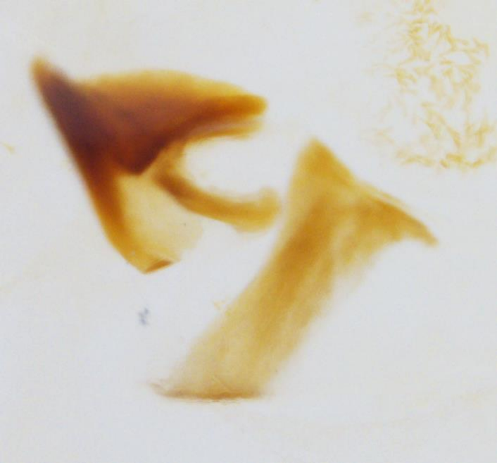

aequatus

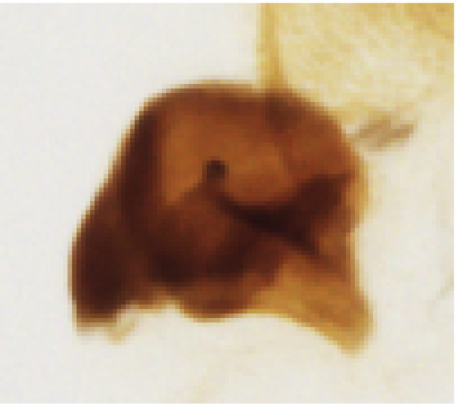

chrysoderus

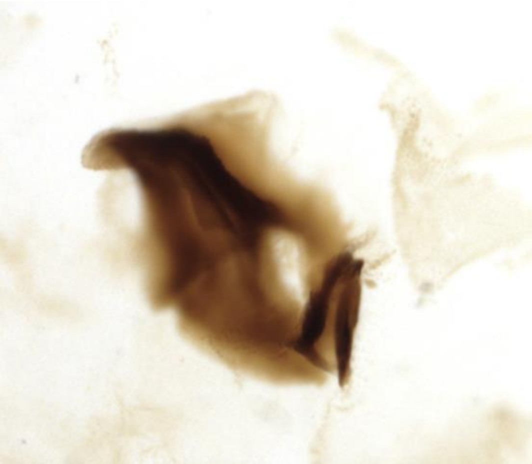

curtipilis

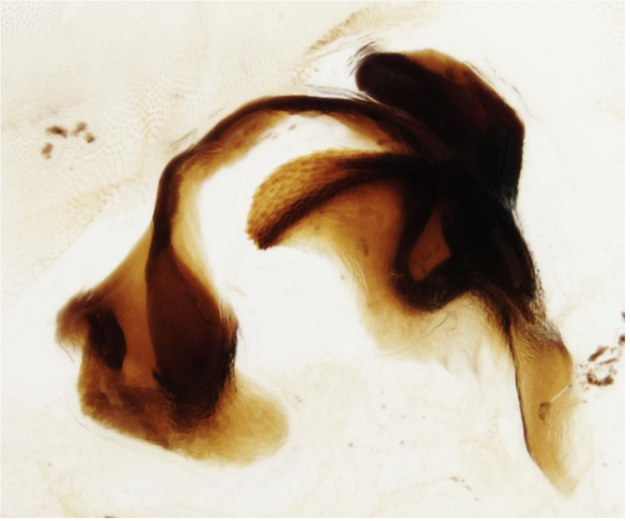

delahayei

Lamella copulatrix

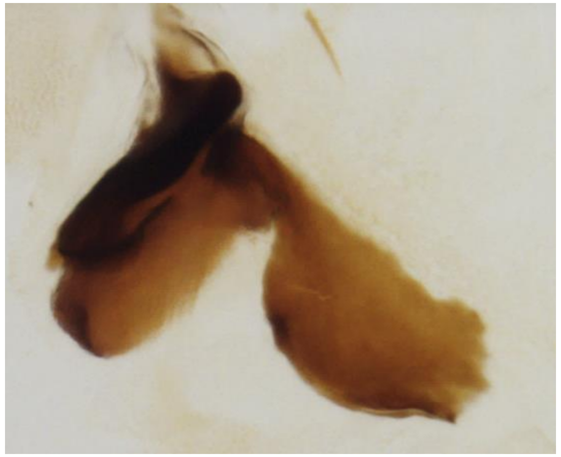

ebenus

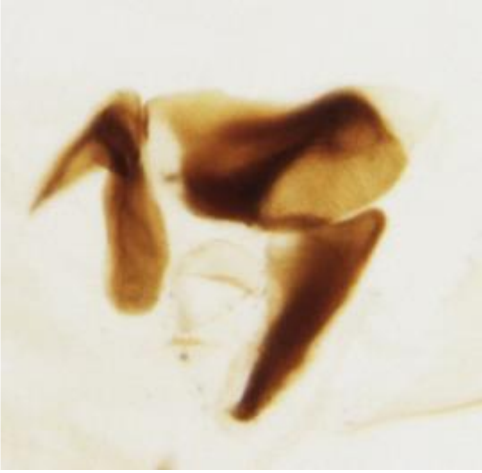

flexicornis

MALE MISSING

hemichlorus

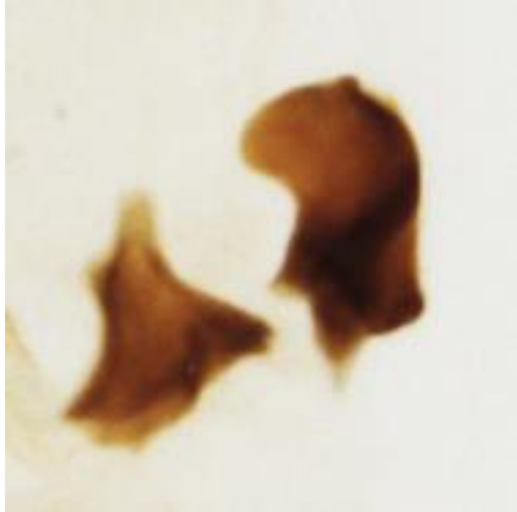

lamtoensis

Lamella copulatrix

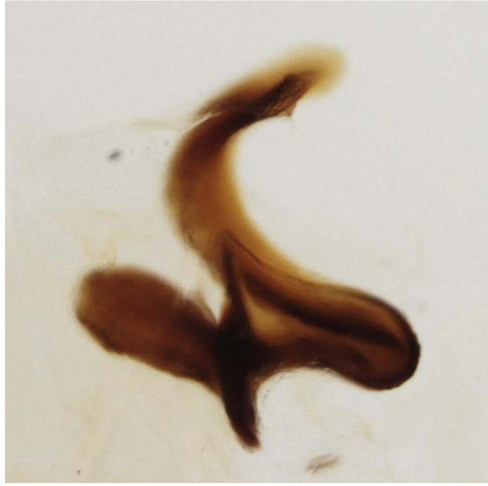

liberianus

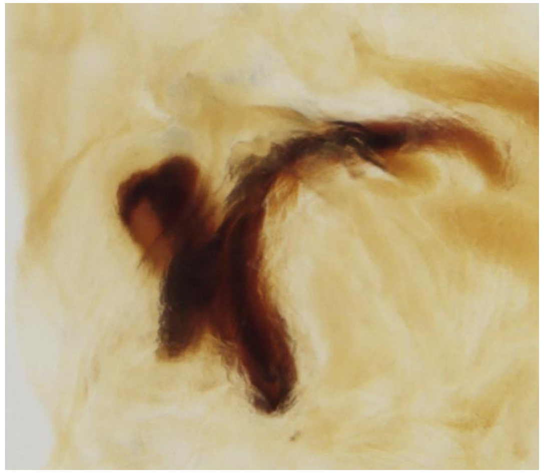

macroliberianus

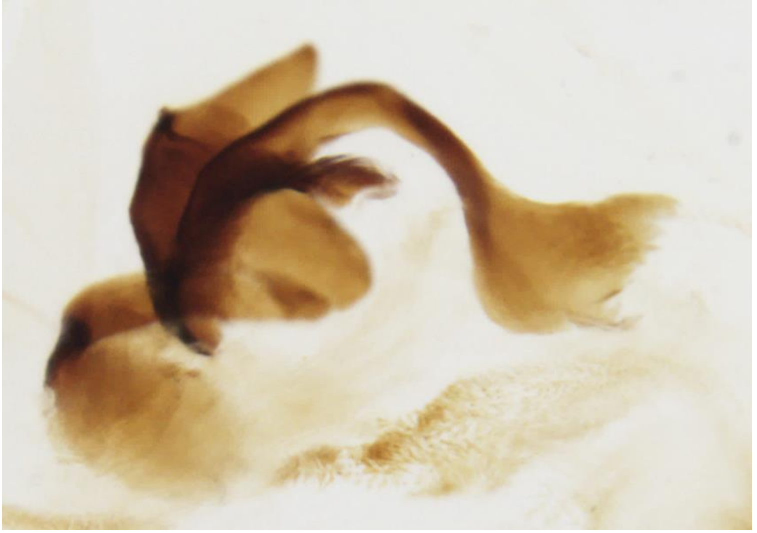

naevius

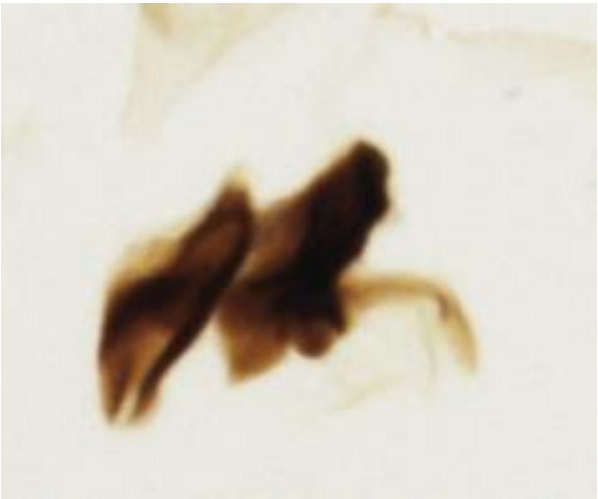

pendjarius

Lamella copulatrix

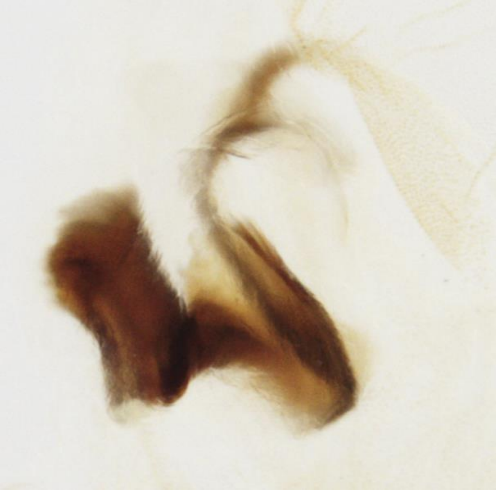

pseudoliberianus

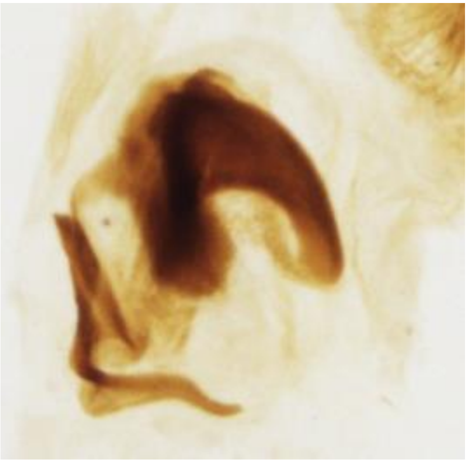

rougonorum

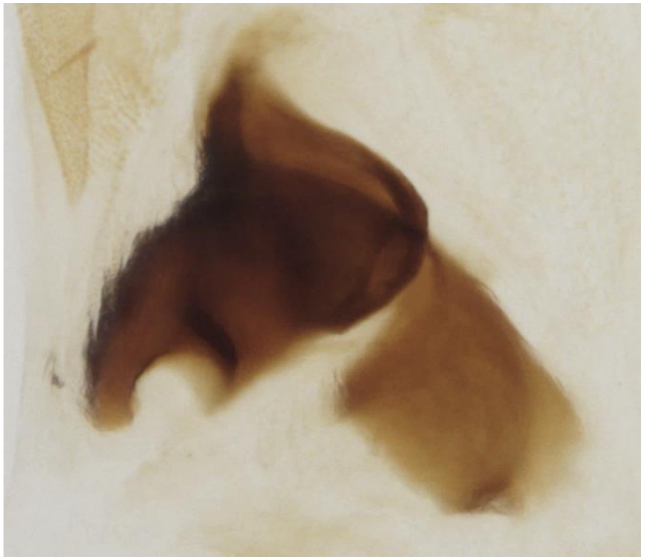

rufobasalis

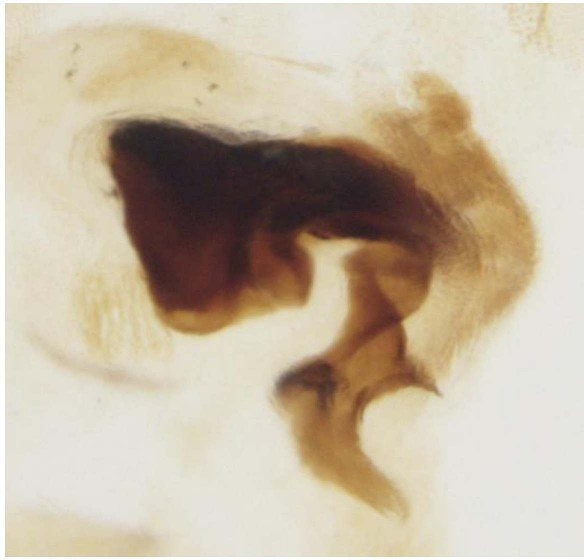

rufopygus

Lamella copulatrix

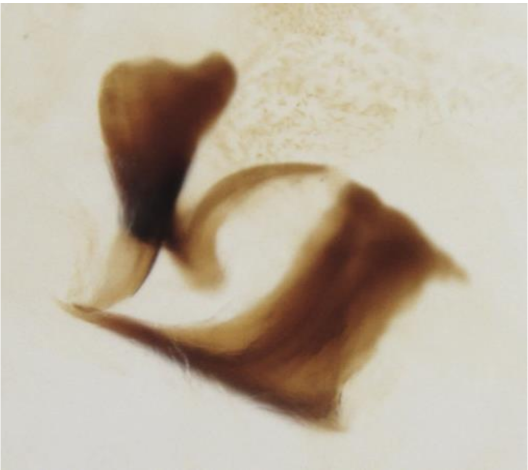

rufostillans

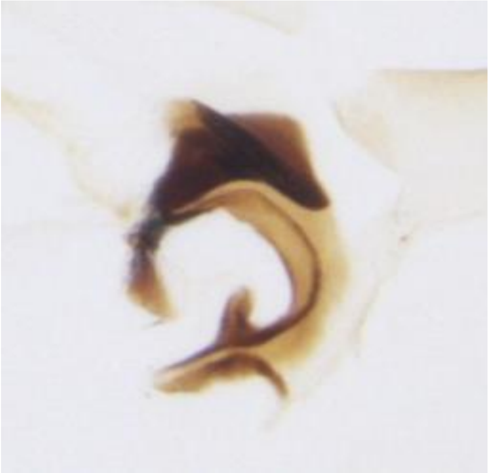

schaufussi

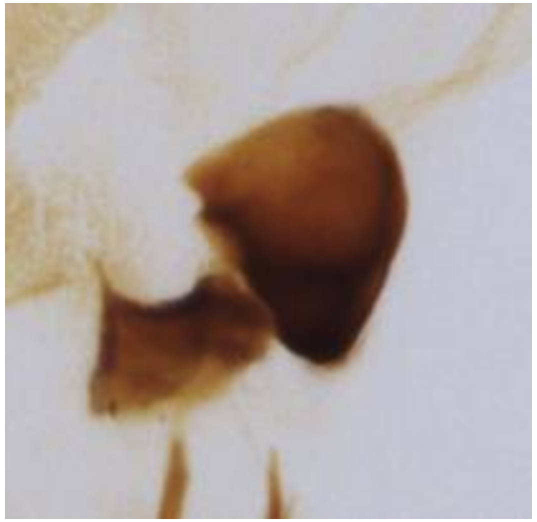

viridiaereus

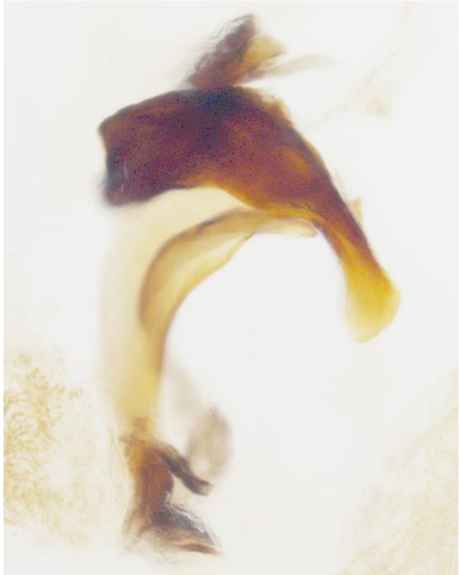

zavattarii

Lamella copulatrix

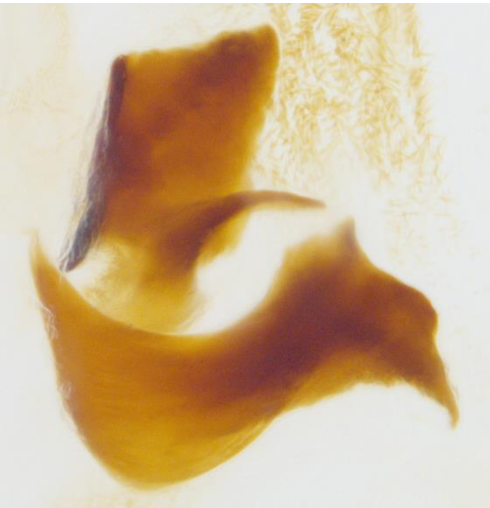

angolensis

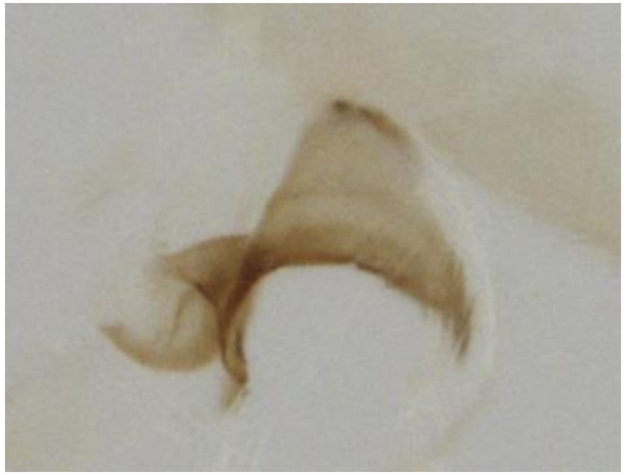

jossoi

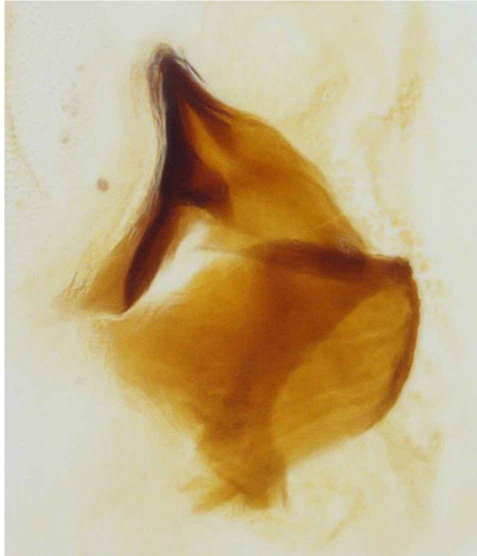

katanganus

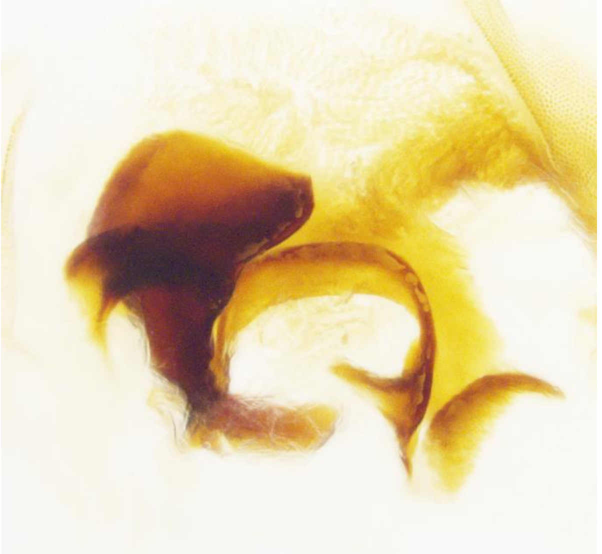

rolandoi

Lamella copulatrix

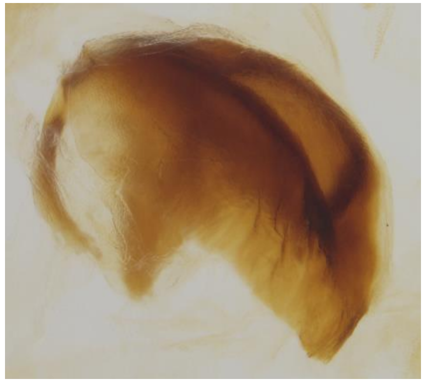

saadaniensis

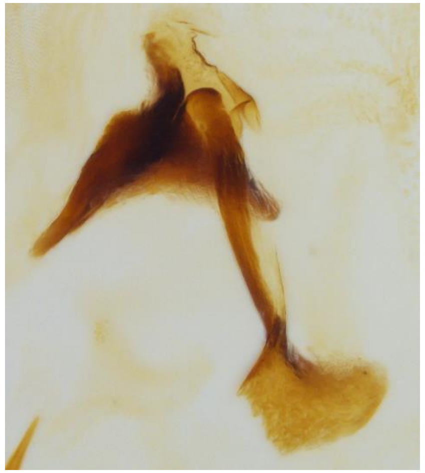

zambesianus

Female genitalia

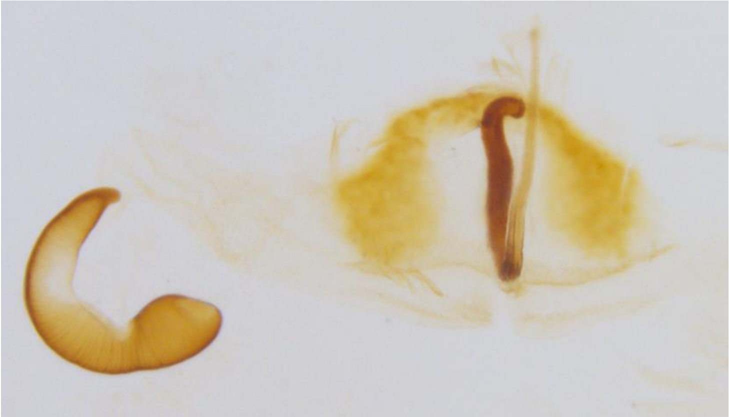

aequatus

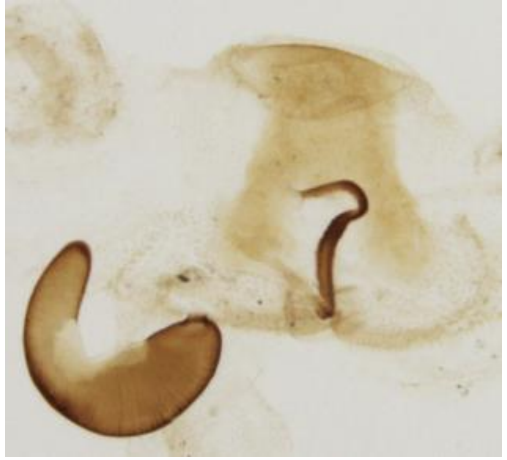

chrysoderus

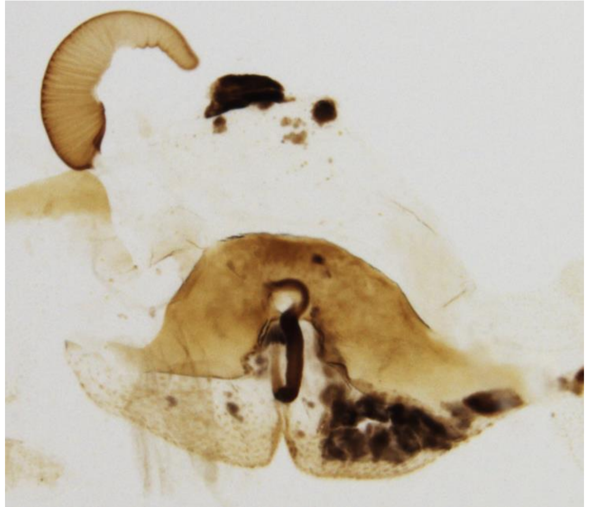

curtipilis

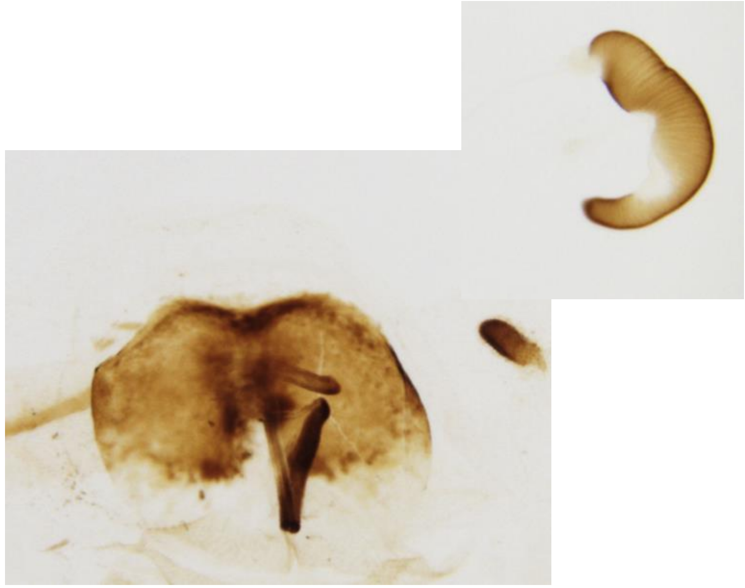

delahayei

Female genitalia

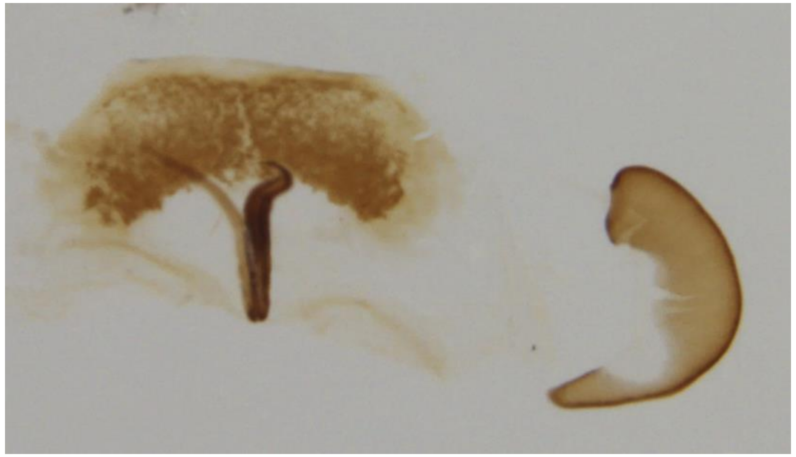

ebenus

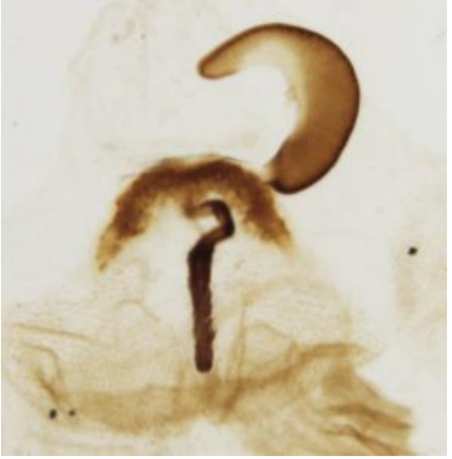

flexicornis

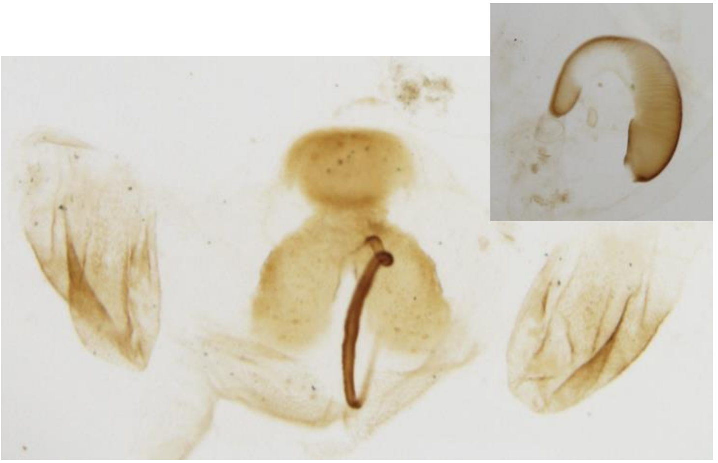

hemichlorus

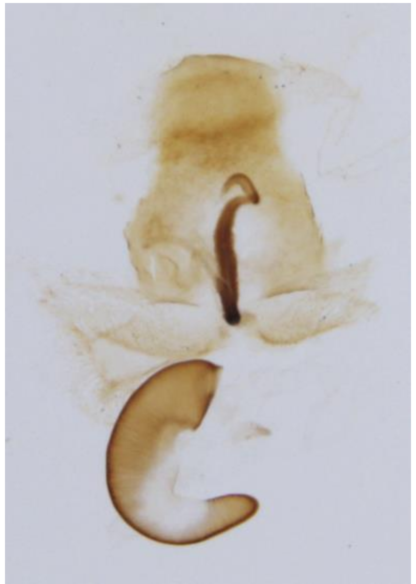

lamtoensis

Female genitalia

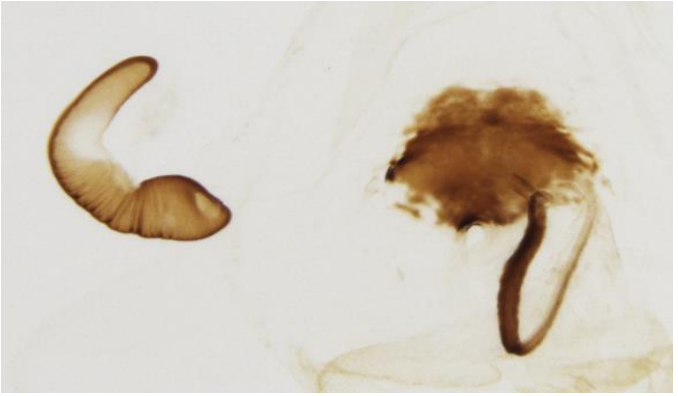

liberianus

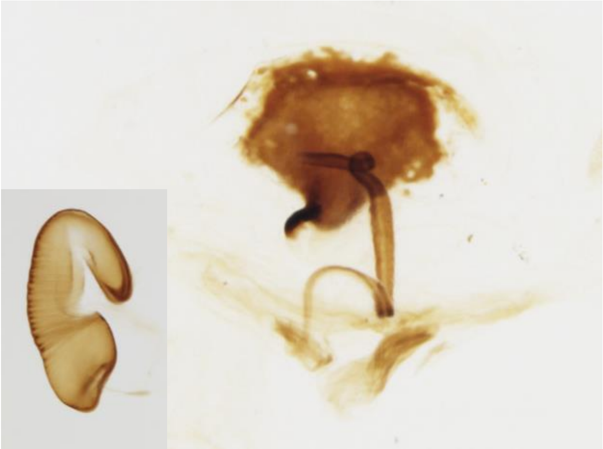

macroliberianus

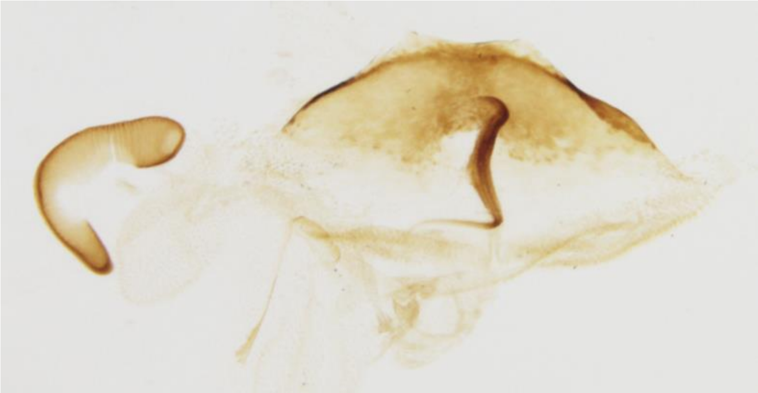

naevius

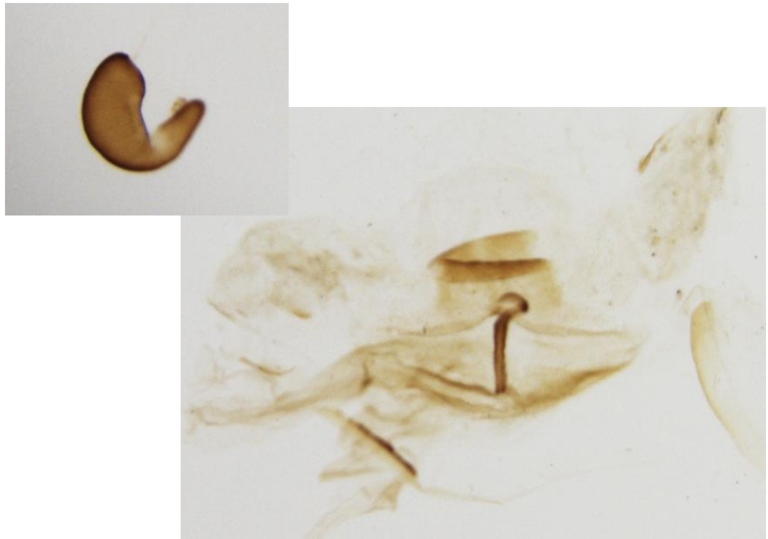

pendjarius

Female genitalia

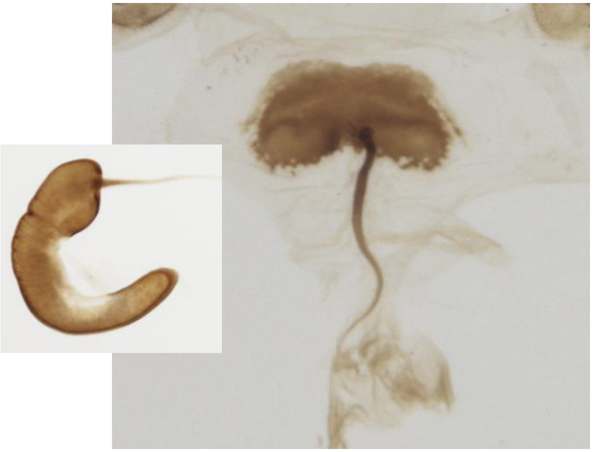

pseudoliberianus

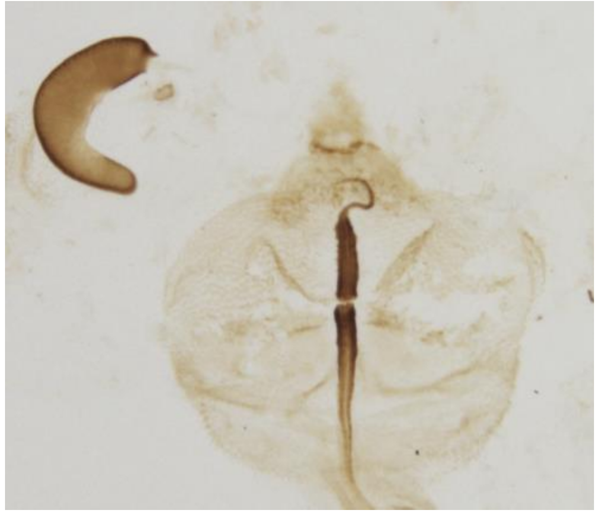

rougonorum

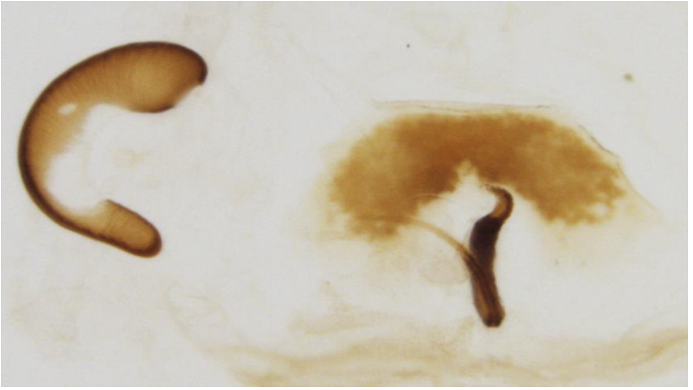

rufobasalis

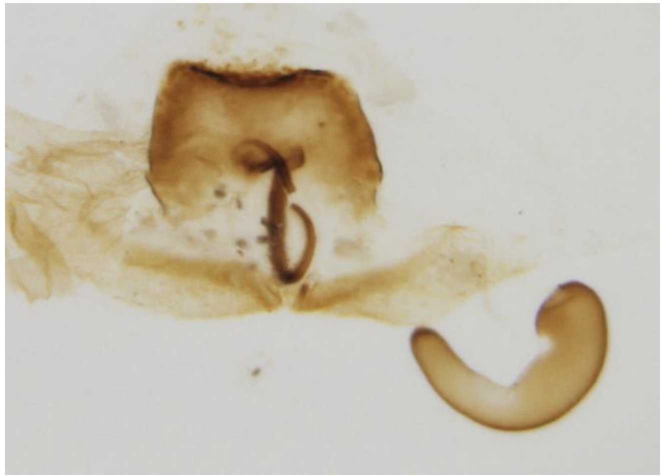

rufopygus

Female genitalia

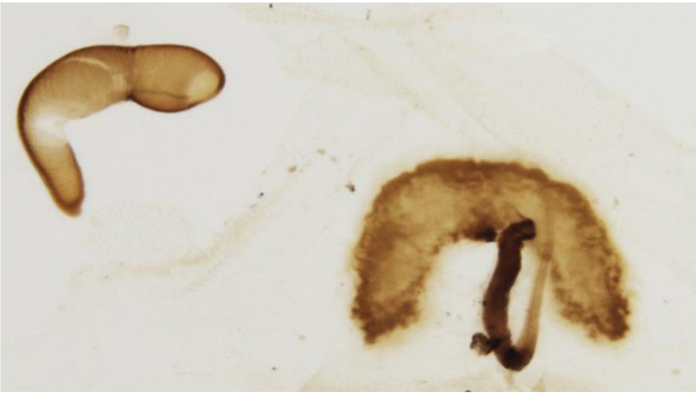

rufostillans

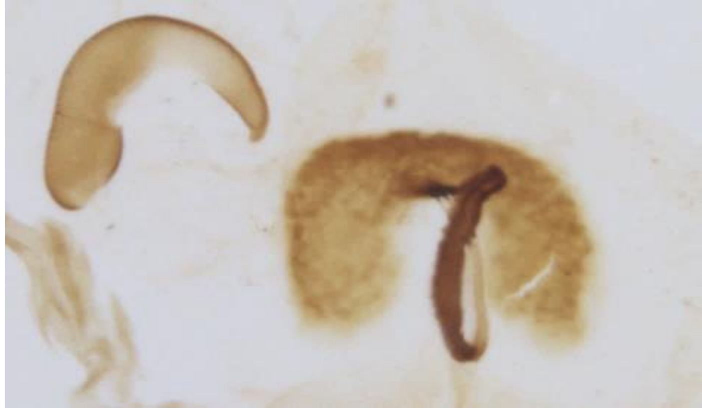

schaufussi

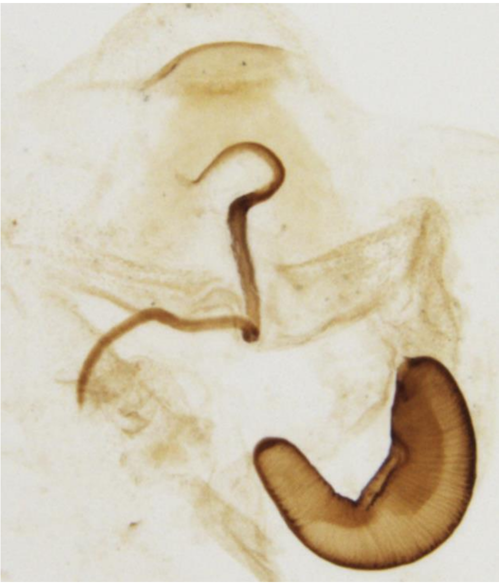

viridiaereus

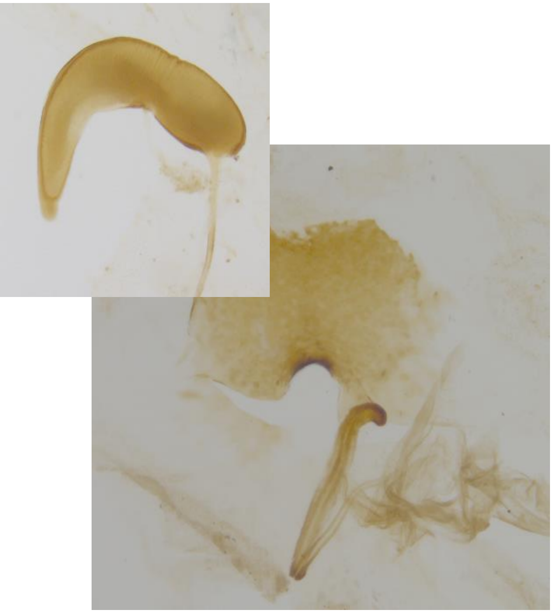

zavattarii

Female genitalia

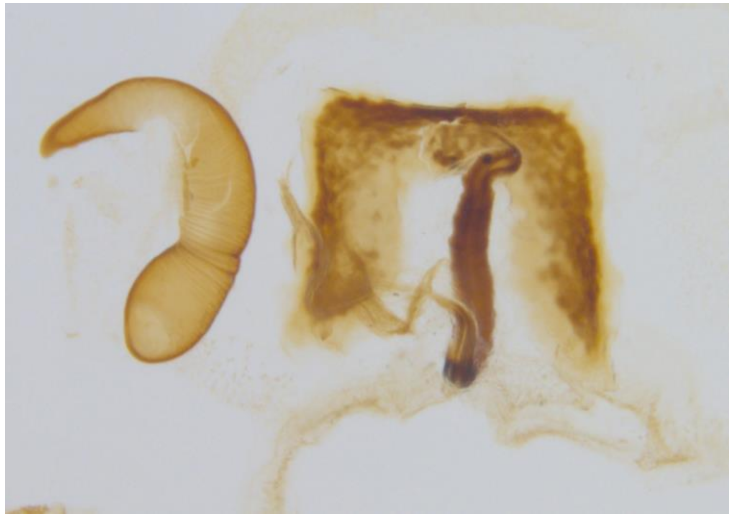

angolensis

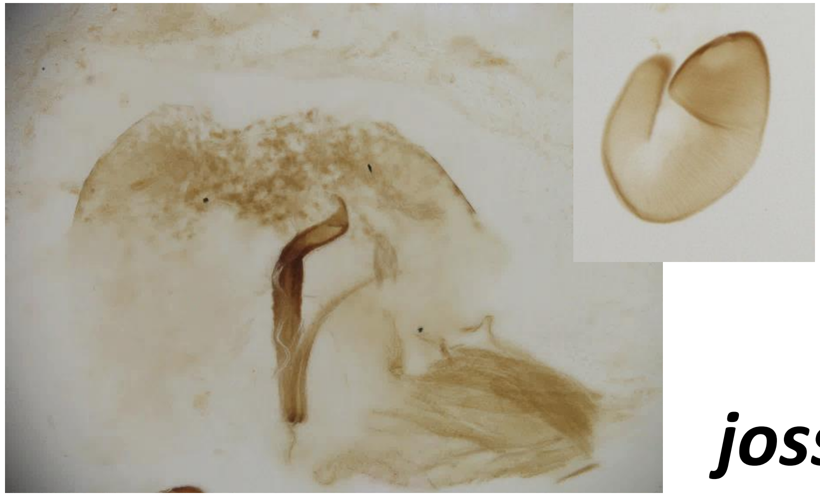

jossoi

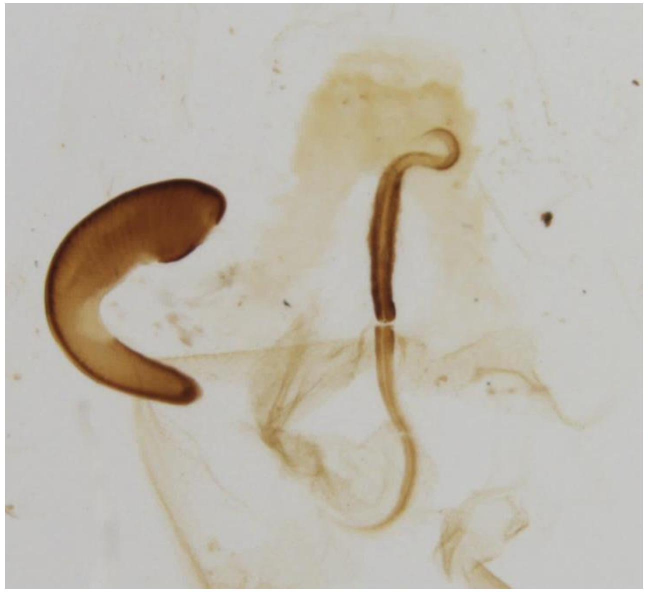

katanganus

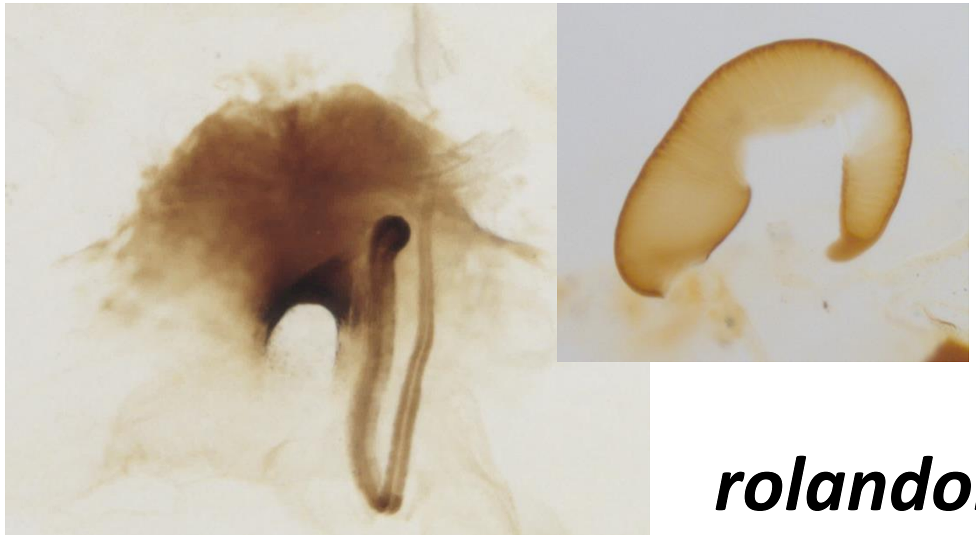

rolandoi

Female genitalia

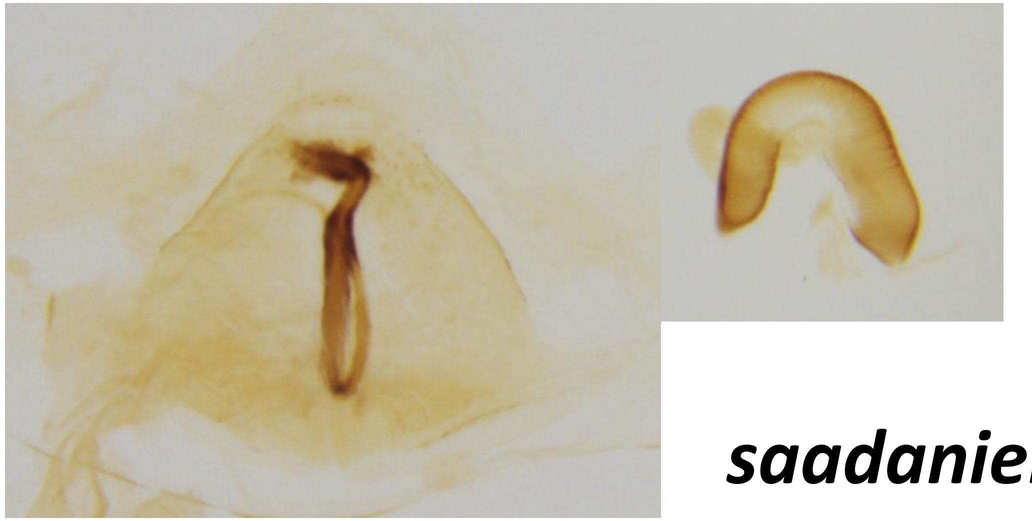

saadaniensis

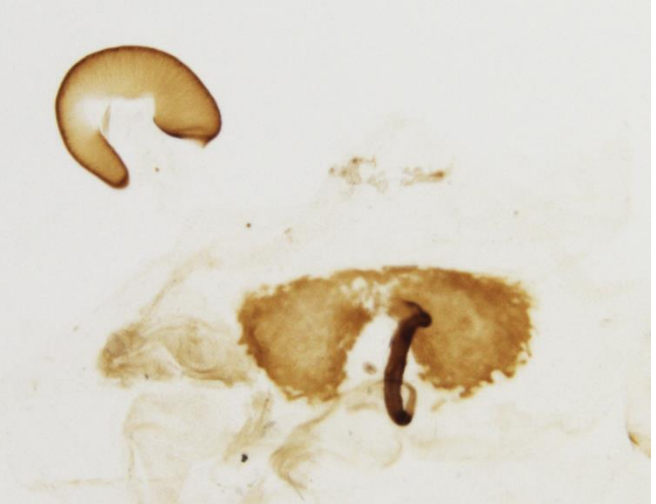

zambesianus
